# Genomic epidemiology reveals multiple introductions of SARS-CoV-2 followed by community and nosocomial spread, Germany, February to May 2020

**DOI:** 10.2807/1560-7917.ES.2021.26.43.2002066

**Published:** 2021-10-28

**Authors:** Maximilian Muenchhoff, Alexander Graf, Stefan Krebs, Caroline Quartucci, Sandra Hasmann, Johannes Hellmuth, Clemens Scherer, Andreas Osterman, Stephan Boehm, Christopher Mandel, Andrea Becker-Pennrich, Michael Zoller, Hans Christian Stubbe, Stefan Munker, Dieter Munker, Katrin Milger-Kneidinger, Madeleine Gapp, Stephanie Schneider, Adrian Ruhle, Linda Jocham, Leo Nicolai, Kami Pekayvaz, Tobias Weinberger, Helga Mairhofer, Elham Khatamzas, Katharina Hofmann, Patricia Spaeth, Sabine Bender, Stefan Kääb, Bernhard Zwißler, Julia Mayerle, Juergen Behr, Michael von Bergwelt-Baildon, Martin Reincke, Beatrice Grabein, Christian Ludwig Hinske, Helmut Blum, Oliver T Keppler

**Affiliations:** 1 Max von Pettenkofer Institute and Gene Center, Virology, National Reference Center for Retroviruses, LMU München, Munich, Germany; 2 German Center for Infection Research (DZIF), partner site Munich, Germany; 3 COVID-19 Registry of the LMU Munich (CORKUM), University Hospital, LMU Munich, Munich, Germany; 4 Laboratory for Functional Genome Analysis, Gene-Center, Ludwig-Maximilians-University, Munich, Germany; 5 Institute and Clinic for Occupational, Social and Environmental Medicine, LMU University Hospital Munich, Munich, Germany; 6 Department of Internal Medicine V, Ludwig Maximilians University of Munich, Comprehensive Pneumology Center Munich (CPC-M), Member of the German Center for Lung Research (DZL), Munich, Germany; 7 Medizinische Klinik und Poliklinik IV, LMU Klinikum, Ludwig-Maximilians-Universität München, Munich, Germany; 8 Medizinische Klinik und Poliklinik III, University Hospital Ludwig-Maximilian University Munich, Germany; 9 German Cancer Consortium (DKTK), Munich, Germany; 10 Medizinische Klinik und Poliklinik I, University Hospital Ludwig-Maximilian University Munich, Germany; 11 DZHK (German Centre for Cardiovascular Research), partner site Munich Heart Alliance, Munich, Germany; 12 Department of Anesthesiology, University Hospital Ludwig-Maximilian University Munich, Munich, Germany; 13 Department of Medicine II, Hospital of the LMU Munich, Munich, Germany; 14 Department of Clinical microbiology and hospital hygiene, University Hospital, LMU Munich, Munich, Germany

**Keywords:** SARS-CoV-2, genomic epidemiology, nosocomial transmission, outbreak control

## Abstract

**Background:**

In the SARS-CoV-2 pandemic, viral genomes are available at unprecedented speed, but spatio-temporal bias in genome sequence sampling precludes phylogeographical inference without additional contextual data.

**Aim:**

We applied genomic epidemiology to trace SARS-CoV-2 spread on an international, national and local level, to illustrate how transmission chains can be resolved to the level of a single event and single person using integrated sequence data and spatio-temporal metadata.

**Methods:**

We investigated 289 COVID-19 cases at a university hospital in Munich, Germany, between 29 February and 27 May 2020. Using the ARTIC protocol, we obtained near full-length viral genomes from 174 SARS-CoV-2-positive respiratory samples. Phylogenetic analyses using the Auspice software were employed in combination with anamnestic reporting of travel history, interpersonal interactions and perceived high-risk exposures among patients and healthcare workers to characterise cluster outbreaks and establish likely scenarios and timelines of transmission.

**Results:**

We identified multiple independent introductions in the Munich Metropolitan Region during the first weeks of the first pandemic wave, mainly by travellers returning from popular skiing areas in the Alps. In these early weeks, the rate of presumable hospital-acquired infections among patients and in particular healthcare workers was high (9.6% and 54%, respectively) and we illustrated how transmission chains can be dissected at high resolution combining virus sequences and spatio-temporal networks of human interactions.

**Conclusions:**

Early spread of SARS-CoV-2 in Europe was catalysed by superspreading events and regional hotspots during the winter holiday season. Genomic epidemiology can be employed to trace viral spread and inform effective containment strategies.

## Introduction

Following the first detection of severe acute respiratory syndrome coronavirus 2 (SARS-CoV-2) in China in December 2019, a global pandemic evolved within months [1]. Modern life and globalisation have resulted in accelerated worldwide dissemination and multiple introductions have been reported from different regions before the implementation of travel restrictions [2-6]. In western Europe, the number of cases peaked in the first wave of the pandemic between March and April 2020 leading to lockdowns to varying degrees in most countries [7,8]. However, to curtail further spread within the communities and within healthcare institutions with vulnerable populations, containment strategies with case isolation, contact tracing and identification of transmission chains are essential.

This is the first pandemic when capacity of next generation sequencing (NGS) is widely available, allowing sharing of sequences at unprecedented speed from various countries with more than 155,000 sequences submitted to GISAID by 21 October 2020 [9]. In a genomic epidemiology approach, viral genomic data can be integrated with spatio-temporal and additional metadata to inform about the origin and transmission networks of disease outbreaks [10]. In this retrospective study of cases from the first wave of the pandemic at the Ludwig-Maximilian University Hospital (LMU Klinikum) in Munich, Germany, we show how genomic epidemiology using a combination of detailed case histories and establishment of human interaction networks together with viral sequence data can be used to trace SARS-CoV-2 spread at an international, national and local level. This information can be used to inform implementation and adjustment of infection prevention and control measures.

## Methods

### Study subjects

Clinical specimens were collected from all coronavirus disease (COVID-19) cases, i.e. PCR-confirmed SARS-CoV-2-infected patients and healthcare workers (HCW) of the LMU Klinikum Munich tested between 29 February and 27 May 2020 at the diagnostic laboratory at the Max von Pettenkofer Institute in Munich, Germany.

### Contact tracing and risk classification

Epidemiological links were analysed by chart reviews and structured interviews. These metadata were collected using a software system developed in-house called SARS-CoV-2 Infection Surveillance (SCoVIS), based on Django (https://www.djangoproject.com) and a PostgreSQL database. Information on every index patient or index HCW was captured by an assigned clinician, recording onset of symptoms, test results, contacts including risk categories, date, place and type of contact. For the investigation of transmission clusters within the clinic, persons who had contact with a COVID-19 case were classified in three risk categories following the definitions from the German public health institute, the Robert Koch Institute, according to duration, closeness and context [11]. Briefly, risk category I indicates contact with a COVID-19 case with high risk, whereas II and III indicate contact with low to very low risk. Detailed definitions of the risk categories of contact persons are summarised in Supplementary Table S1.

### Classification of probable modes of transmission 

We estimated the most likely modes of transmission based on previously reported incubation periods [12] using the following criteria:

- Travel-associated: PCR-confirmed SARS-CoV-2 infection up to 10 days after returning from a COVID-19 risk area as defined by the Robert Koch Institute at that time;
- Hospital-acquired infection in HCW: infection 4–10 days after contact with a SARS-CoV-2-infected person within the hospital and no other reported exposure to SARS-CoV-2;
- Hospital-acquired infection in patients: treatment/admission for a non-COVID-19-related reason and positive SARS-CoV-2 PCR-result at least 4 days after hospitalisation/visit to the clinic;
- Nursing home-acquired infection: confirmed infection at the time of admission to our clinic of a patient from a nursing home;
- Community-acquired: none of the above;
- Unknown: fulfilling more than one of the above criteria or no detailed information available.

### Nucleic acid isolation and RT–qPCR

Viral RNA was extracted from respiratory samples either with the QiaSymphony DSP Virus/Pathogen Kit (Qiagen, Hilden Germany), the Maxwell RSC 48 RNA kit (Promega, Madison, United States (US)) or the EZ1 Virus Mini Kit (Qiagen). For diagnostic testing, the following PCR assays were used: The protocol from the US Centers for Disease Control and Prevention, the Charité protocol, the Seegene Allplex 2019-nCoV Assay or the Cobas SARS-CoV-2 assay (Roche, Mannheim, Germany) as described previously [13].

### Analysis of SARS-CoV-2 amplicon-based sequencing

Amplicon pools spanning the SARS-CoV-2 genome were prepared based on the ARTIC network nCoV-2019 sequencing protocol v2 and analysed using the ARTIC bioinformatics protocol (http://artic.network/ncov-2019) (see the Supplement for methodological details) [14]. The consensus sequences and accompanying metadata for the samples were uploaded to the GISAID repository.

### Ethical statement

Patients were part of the COVID-19 Registry of the LMU Klinikum (CORKUM, World Health Organization (WHO) trial identification number DRKS00021225). Staff members were part of the Care-Corona-Immune Study (CCI). All data were anonymised for analysis and the study was approved by the local ethics committee (CORKUM No: 20–245 and CCI No: 20–247).

## Results

### Multiple independent introductions of SARS-CoV-2 into the Munich Metropolitan Region

We sequenced clinical specimens collected from 289 SARS-CoV-2-infected individuals between 29 February and 27 May 2020 at the LMU Klinikum in Munich, which is the second largest university hospital in Germany. The majority of COVID-19 cases were male (205/289 (70.9%)) with a median age of 55 years (interquartile range (IQR): 37–71). Of note, 61 of these cases (21.1%) were HCW. We obtained 174 near full-length SARS-CoV-2 genomes with a genome coverage of more than 90% that were further used for phylogenetic studies (see Supplementary Table S2 for GISAID accession numbers and metadata). Genome coverage was related to viral load of the original respiratory sample (Spearman rank correlation coefficient r = 0.74; p < 0.0001; data not shown) with a median genome coverage of 99.5% (IQR: 98.8–99.7). Sequenced isolates were obtained directly from nasopharyngeal swabs (n = 144), endotracheal aspirates (n = 23), bronchoalveolar lavage fluid (n = 2) or sputum (n = 5).

**Figure 1:**
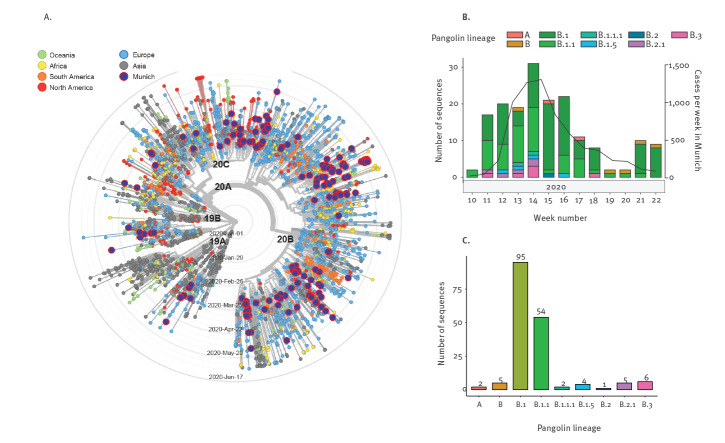
Phylogenetic relationship of SARS-CoV-2 isolates from the Munich Metropolitan Region, February–May 2020 (n = 174) and global strains

We performed a maximum likelihood phylogenetic analysis of the isolates from these 174 individual patients together with a global subsampling of 3,776 sequences deposited at GISAID (accession date: 15 June 2020, Supplementary Table S3). The SARS-CoV-2 sequences from the LMU Klinikum were distributed throughout the global phylogenetic tree, suggesting multiple independent introductions in the Munich Metropolitan Region (Figure 1A). During these first weeks of the pandemic in south-eastern Germany, the predominant subtypes using the Pangolin classification were B.1 and B.1.1 (Figure 1B and C).

SARS-CoV-2: severe acute respiratory syndrome coronavirus 2.

A. Time-resolved maximum likelihood phylogeny of 3,776 global subsampled sequences available from GISAID (accession date: 6 June 2020) and 174 genomes from Munich obtained in this study (dark blue circle with red border). Nextstrain nomenclature clades are indicated above the main branches. Accession numbers are shown in Supplementary Table S3.

B. Breakdown of sequenced cases (n = 174) according to Pangolin nomenclature across calendar weeks of 2020. Total number of cases per week in Munich are plotted on the right y-axis.

C. The total number of sequenced cases is shown for each Pangolin lineage.

### Phylogenetic analysis of early COVID-19 cases from Munich and the neighbouring Alpine region

To explore the source of the early introductions, we combined SARS-CoV-2 sequence data with detailed patient history. Most early COVID-19 cases at the LMU Klinikum had a travel history to either Austria or northern Italy, which prompted us to perform a regional phylogeographical analysis of these 174 isolates together with subsampled sequences at GISAID from Italy (n = 61), Austria (n = 88) and the rest of Germany (n = 92) (accession date: 6 June 2020, Supplementary Table S4).

**Figure 2:**
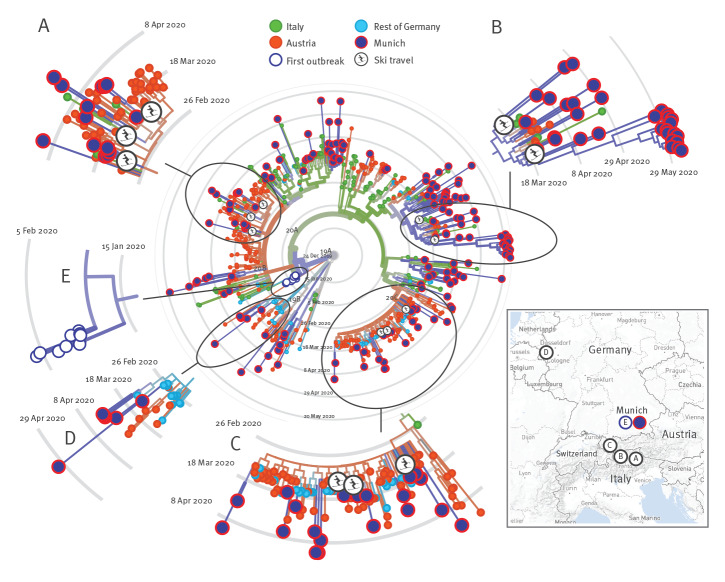
Regional phylogenetic relationship of SARS-CoV-2 isolates, Munich Metropolitan Region, February–May 2020 (n = 174), with strains from Austria, Germany and Italy

The patients’ travel history together with unsupervised clustering of isolates using the maximum likelihood phylogeny revealed multiple introductions from various skiing areas in the Alps with subsequent community transmission for some cases. Of note, three unconnected individuals, including the first COVID-19 case at the LMU Klinikum, returned from a skiing vacation in the same skiing area in the Dolomites in Northern Italy, in the period 22 to 28 February 2020 (Figure 2A, skier symbols). The isolates of these three travellers clustered together, indicating infection with the same strain circulating in that area at that time or possibly even transmission from the same contact. A pattern and geophylogenetic link associated with travel and skiing vacations was also seen in two travellers returning from South Tyrol, Northern Italy (Figure 2B, skier symbol), as well as a couple and another traveller returning from the popular skiing area of Ischgl in Tyrol, Austria (Figure 2C, skier symbols); the latter destination had been implied as a hotspot of the early coronavirus pandemic in Europe [15]. Several other COVID-19 cases without recent travel history presented at a later time carrying identical or highly similar virus variants (see Supplementary Figures S1–S3 for divergence), indicating subsequent local spread in the Greater Munich Area. We also performed an analysis including additional SARS-CoV-2 sequences available from France and Switzerland. However, the cases in Munich did not cluster with sequences from these countries to the same extent as with Italian and Austrian sequences (Supplementary Figure S4).

SARS-CoV-2: severe acute respiratory syndrome coronavirus 2.

The 174 SARS-CoV-2 genomes obtained in this study (dark blue circles with red border) are shown in relation to subsampled sequences deposited at GISAID from Italy (green, n = 61), Austria (red, n = 88) and the rest of Germany (light blue, n = 92) in a time-resolved maximum likelihood phylogenetic tree. Accession numbers are shown in Supplementary Table S4. Sequences generated from cases with a recent travel history to skiing areas are indicated with skier symbols. Genomes of cases from the first outbreak of SARS-CoV-2 infections in the Munich area are shown as white circles with blue borders. The map indicates the geographical locations of the magnified clusters A**–**E**.**

A. Magnified insert of a cluster of sequences from cases including three travellers returning from skiing-holidays in the Dolomites, northern Italy.

B. Cluster of genomes including early cases of patients returning from South Tyrol, northern Italy.

C. Group of sequences with three ski-travellers returning from Ischgl, Tyrol, Austria.

D. Cluster of genomes with early cases from the Heinsberg area, North-Rhein-Westphalia, Germany.

E. Zoomed view of sequences from the first outbreak in the Munich area involving an introduction by a Chinese business traveller at the end of January 2020 (white circles with blue border).

**Figure 3:**
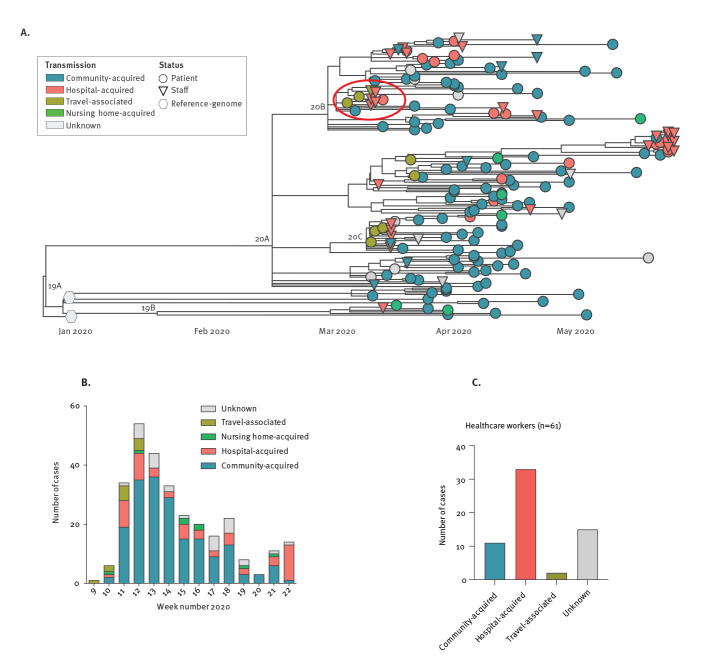
Local phylogenetic relationship of SARS-CoV-2 genomes and transmission dynamics, Munich Metropolitan Region, February–May 2020 (n = 289)

Interestingly, we observed a few cases presenting to the LMU Klinikum at a later time between 11 March and 2 May 2020, that clustered with SARS-CoV-2 isolates sampled from a group of people from Gangelt in the Heinsberg area in North Rhine-Westphalia, ca 650 km north-west of Munich (Figure 3D).

SARS-CoV-2: severe acute respiratory syndrome coronavirus 2.

**Figure 4:**
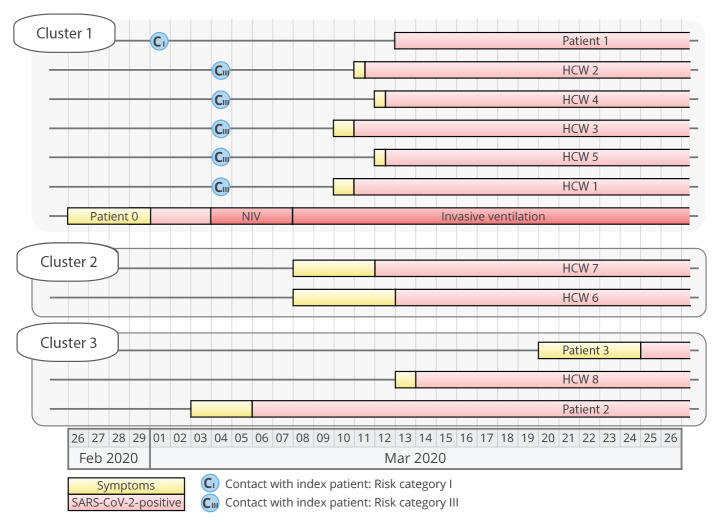
Chronological order of events of the first COVID-19 outbreak at the Ludwig-Maximilian Klinikum, Munich, February–May 2020 (n = 12)

COVID-19: coronavirus disease; HCW: healthcare workers; NIV: Non-invasive ventilation; SARS-CoV-2: severe acute respiratory syndrome coronavirus 2.

**Figure 5:**
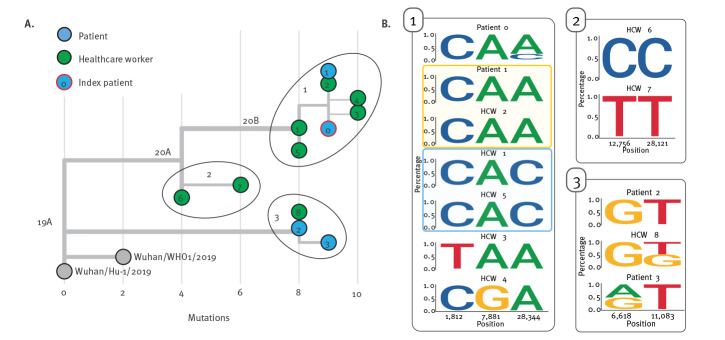
Investigation of nosocomial SARS-CoV-2 transmission clusters using genomic epidemiology Munich, February–May 2020 (n = 12)

A. The phylogenetic relationship of SARS-CoV-2 genomes obtained in this study from HCW (n = 39, circles) and patients (n = 135, triangles) are shown in a maximum likelihood time-resolved phylogenetic tree in relation to two reference genomes from Wuhan, China (Wuhan/WHO1/2019 and Wuhan/Hu-1/2019, hexagons). Presumable transmission modes are indicated by tip colours. Nextstrain nomenclature clades are indicated above the main branches. The red ellipse indicates the sequences of the cluster of cases that is investigated further in Figures 4 and 5.

B. The most likely mode of SARS-CoV-2 transmission is indicated for all cases (n = 289), from the first case on 26 February 2020 (week 9) until 27 May 2020 (week 22).

C. The overall proportion of presumable transmission modes are indicated for patients and HCW.

Of note, the first COVID-19 cases reported in Germany were in the Munich Metropolitan Region and related to an outbreak at a local company involving 16 individuals that resulted from an initial transmission of a Chinese business traveller in late January 2020 [16-18]. These formed a separate phylogenetic cluster with no additional genetically identical cases sampled at LMU Klinikum at later stages of the pandemic (Figure 3E).

To allow better interaction with the sequence data shown in this study, we build a Narrative using Nextstrain that can be accessed here: https://nextstrain.org/community/narratives/axgraf/SARS-CoV-2/Munich.

### Hospital-associated infections among clinical staff

Among all SARS-CoV-2 infections (n = 289) diagnosed in the LMU Klinikum by the Max von Pettenkofer Institute during this early period of the first pandemic wave (weeks 9–22 of 2020), we observed cases both among HCW (n = 61; 21.1%) and patients (n = 228; 78.9%). The 174 SARS-CoV-2 genomic sequences derived from samples from patients and HCW were closely related and clustered together in the phylogeny (Figure 3A). Among the early cases during the first 2 weeks (weeks 9 and 10) of this local epidemic we observed a high proportion of travel-associated cases with subsequent transmission into the community as well as hospitals and nursing homes (Figure 3B). The proportion of presumable hospital-acquired infections (definition see Methods) was 54.1% among HCW (33/61 cases) compared with 9.6% among patients (22/228 cases) (Figure 3C).

### Dissection of COVID-19 clusters using spatio-temporal and interaction metadata as well as genomic tracing to establish transmission trees and risk constellations

To spatio-temporally resolve presumed nosocomial transmission chains, we investigated clusters of infection at the LMU Klinikum, combining detailed case histories based on anamnestic workup and patient files with the interactive phylogenetic viral genome analysis platform using the Auspice software. As an example, we report here the outbreak investigation of the earliest cluster of cases at our hospital.

The chronological order of events is summarised in Figure 4. After returning from a skiing trip to Italy, Patient 0 started feeling unwell on 26 February 2020 and presented 3 days later to the emergency department at the LMU Klinikum with fever (39.3 °C), respiratory distress and dry cough. At that time, Italy was not considered a COVID-19 risk region according to the Robert Koch Institute, and after ruling out influenza A/B and respiratory syncytial virus infection, the patient was transferred to an intermediate care unit over night without further hygiene precautions. On the next day, Patient 0 was tested PCR-positive for SARS-CoV-2. Consequently, the patient was isolated and strict personal protective measures were implemented. 

The timeline summarises the events involving patients and HCW in a presumed nosocomial outbreak between 26 February and 26 March 2020. Yellow boxes: onset of symptoms; red boxes: date of first positive SARS-CoV-2 PCR result, the length of box does not correspond to the duration of PCR-positivity; blue circle: first time point and risk category of the contact to the index person (Patient 0). The risk category of the contact is indicated within the blue circle (C_I_ or C_III_, none of these contacts was defined as risk category II). Cases are shown in three groups (Clusters 1, 2 and 3) based on the findings shown in the subsequent phylogenetic analysis. HCW included five nurses and two doctors.

On 4 March, this patient (Patient 0, Figure 4, Cluster 1) presented with respiratory instability and was transferred to the intensive care unit (ICU) where non-invasive ventilation (NIV) was started. Because of increasing respiratory insufficiency, intubation was necessary. The patient developed severe acute respiratory distress syndrome, and extracorporeal membrane oxygenation (ECMO) had to be started on 8 March. The treatment showed to be effective, and the patient was weaned successfully from ECMO on 23 March. Later tracheotomy was performed, and prolonged ventilator weaning was necessary.

Between 8 and 10 March, five people (one patient and four HCW), who had been in contact with this patient (Patient 0) at some stage, became symptomatic and tested PCR-positive for SARS-CoV-2 (Figure 4, Clusters 1 and 2). Multiple transmissions originating from Patient 0 were suspected, creating anxiety and distress among members of this clinical unit regarding the effectiveness of personal protective equipment and potentially unrecognised routes of transmission of this still poorly characterised coronavirus. To quickly curtail the outbreak, respiratory swabs from a total of 69 HCW and patients from this unit were analysed by RT-PCR in the period from 11 to 14 March 2020, identifying an additional four individuals with COVID-19. To examine the assumption that all these infections originated from Patient 0, we investigated this outbreak scenario using in-depth genomic epidemiology:

On 10 March, 6 days after admission of Patient 0 to the ICU, HCW 1 who had initially started the NIV therapy and subsequently intubated the patient noticed a sore throat during the evening and tested positive for SARS-CoV-2 the following day on 11 March (Figure 4). On the same day, two HCW (HCW 2 and 3) who had spent their entire shift with the patient while on NIV on their first day on the ICU (4 March) complained about sore throat and muscle aches and tested positive. On the same evening, the HCW 4 who had assisted the NIV therapy and the intubation presented with fever and also tested positive. Detailed interviewing revealed that shortly after intubation of Patient 0 on 4 March, a ventilator system disconnection occurred and HCW 4 was temporarily exposed to air exiting the lungs of Patient 0. On the following day (12 March), the HCW 5 treating Patient 0 on the isolation ward also tested positive for SARS-CoV-2. All these HCW reported to have followed infection prevention and control measures (IPC) using personal protective equipment at all times when in contact with the patient, including FFP2 masks; they were therefore classified as risk category III contacts.

These five cases were confirmed to be epidemiologically linked to Patient 0 by NGS analysis (Figure 5A, Cluster 1). Interestingly, HCW 1 and 5 did not share the single nucleotide variant C28344A present in the other related sequences. In the nasopharyngeal swab specimen of Patient 0, we detected the minority wild-type variant 28344C with a frequency of 22.7% suggesting that this minority wild-type variant had probably been transmitted to HCW 1 and 5, while the variant C28344A was transmitted to Patient 1 and HCW 2, 3 and 4 (Figure 5B). Two independent de novo substitutions were detected in the sequences of HCW 3 (C1812T) and HCW 4 (A7881G). Given these sequence similarities and documented risk contacts, it is likely that these HCW were infected directly by Patient 0. However, with an incubation period of 7 days and more after risk contact in these cases, which is slightly longer than the average incubation period of 5 days from other studies, it cannot be excluded that Patient 0 first infected another unidentified intermediate host who subsequently transmitted to these HCW [12].

SARS-CoV-2: severe acute respiratory syndrome coronavirus 2.

A. Maximum likelihood phylogeny of SARS-CoV-2 sequences from HCW (green circles), patients (blue circle) and the index person (Patient 0, blue with red border) involved in this outbreak in relation to two reference genomes from Wuhan, China. The number of mutations in relation to the reference sequence Wuhan/Hu-1/2019 is shown on the x-axis. Sequences are divided in three groups: (i) Sequences from five HCW (green circles, 1–5) and one patient (blue circles, Patient 1) that are similar to the sequence of the index case (blue circle with red border, Patient 0). (ii) Two sequences from HCW 6 and 7 that turned out not to be related to the index case, Patient 0. (iii) A smaller separate cluster was identified consisting of HCW 8 and two patients (Patient 2 and Patient 3) that were infected in a nursing home.

B. For each group, minority allele variants are represented by sequence logos showing the variant frequency calls as proportionally sized nucleotide letters. Nucleotide positions are indicated in relation to the reference genome Wuhan/Hu-1/2019 (GenBank accession number: MN908947.3).

In total, 69 persons with contact to Patient 0 or one of the other COVID-19 cases in this outbreak were tested (32, 29 and eight were category I, II and III contacts, respectively). Among these screened persons, three infected HCW and one patient were identified. Patient 1 was admitted to the LMU Klinikum on 28 February for a COVID-19-unrelated reason and spent one night (1 March) in the same room with Patient 0 before the latter was diagnosed with COVID-19 and protective measures were implemented, thus classifying this as a risk category I contact. Patient 1, while testing PCR-negative on 3 March, tested positive on 13 March, at a point when they were free of any COVID-19-related symptoms. The virus sequence obtained from Patient 1 was identical to the consensus sequence obtained from Patient 0.

On 13 March, HCW 6, who had treated Patient 0 before and after his isolation, became ill with fever and tonsillitis and tested positive for SARS-CoV-2. While a transmission directly from Patient 0 or this cluster seemed likely, sequence analysis revealed that their infection was unrelated and transmission must have occurred from another source (Figure 5, Cluster 2). Another HCW (HCW 7) who was involved in the treatment of Patient 0 had mild symptoms beginning 8 March (fatigue) and tested SARS-CoV-2-positive on 12 March. Also here, NGS analysis demonstrated that this infection was not related to Patient 0. It turned out that HCW 7 had been on a skiing trip to Tyrol, Austria, between 26 February and 1 March, suggesting that this infection was presumably travel-associated.

Another HCW (HCW 8), who was treating Patient 0, tested positive on 14 March, yet with a sequence clearly distinct to that derived from Patient 0 (Figure 5A, Cluster 3). Further investigation showed that Patient 2, who was admitted to the clinic on 6 March and who was also treated by HCW 8, shared this very SARS-CoV-2 sequence. Patient 2 was an elderly person with productive cough who did not tolerate wearing a face mask, so HCW 8 was heavily exposed and transmission from Patient 2 seems likely although HCW 8 was wearing an FFP2 mask at all times. One week later on 20 March, Patient 3 was admitted from the same nursing home as Patient 2 to another ward at the LMU Klinikum, presenting with productive cough and diarrhoea. Patient 3 was also positive for SARS-CoV-2 and the viral sequence was identical to that of Patient 2 with one additional nucleotide substitution, suggesting that both patients had acquired the infection at their nursing home.

## Discussion

This study illustrates how the first wave of the SARS-CoV-2 outbreak in the Munich area between March and June 2020 was probably fuelled by multiple introductions linked to social and cultural events, i.e. skiing holidays in the Alpine region and the carnival season, before travel restrictions were implemented. To unravel the sources of introduction, we combined detailed travel history with genomic data available from different Alpine regions and identified several cases with SARS-CoV-2 isolates related to isolates circulating in those areas at the time of travel. Other studies have also suggested that people travelling to skiing resorts in the Alpine region during the early pandemic phase played a substantial role in early SARS-CoV-2 dissemination in Europe [19,20].

Furthermore, we found that SARS-CoV-2 circulating in the Munich area showed high similarity to virus sequences from the Heinsberg area. Heinsberg has been linked to a superspreading outbreak originating from an infected couple and related to a single carnival session on 15 February 2020 with more than 300 contacts [21], indicating that introduction of these variants into the Munich Metropolitan Region may have occurred from people travelling from the Heinsberg area. However, given the low genetic divergence, incomplete sampling and unrestricted travel at this early stage of the pandemic, introduction of these variants may also have occurred from a different source.

Concerning the well described first cluster of cases associated with a business traveller from China to a local company in the Munich area in late January 2020 [16,17], containment seems to have been successful, since we did not observe a single case between that time until the, genetically distinct, first community-acquired infections in early March. However, further transmissions from that cluster may have gone undiagnosed or subsequent cases could have presented to other healthcare providers.

The rate of probable nosocomial infections among inpatients in our study (9.6%) was in the same range as in other institutions during the early pandemic phase, e.g. 15% in a London teaching hospital, but substantially higher than in some other reports, e.g. < 1.7% at a hospital in the Boston area, indicating that well established IPC, among other factors such as the burden of infectious patients, can have a significant impact on nosocomial spread [22,23]. In our centre, IPC were constantly adapted through the course of this pandemic, resulting in a significant reduction of nosocomial cases among patients. Nevertheless, hospital-acquired infections continued to play a significant role among HCW, with an overall fraction of 54% of cases in this group during this early pandemic phase. However, given the lack of structured data regarding risk constellations outside the hospital, the transmission modes reported here could only be estimated and are subject to error. We chose to use a definition of likely hospital-associated infections for HCW that were tested positive within 4–10 days after contact with a COVID-19 case within the clinic, based on previously reported average incubation periods [12]. Using this cut-off might not reflect all possible scenarios. To further delineate transmission sources, genomic epidemiology was applied for some constellations as exemplified in the case series.

In this analysis, SARS-CoV-2 infection of five HCW was likely to be linked to the index case (Patient 0), while the infections of three other HCW and two other patients were definitely not linked to Patient 0. One likely nosocomial infection (Patient 1) was identified. The analysis of this outbreak exemplifies that the combination of detailed spatio-temporal metadata on interactions and NGS-based virus phylogeny are powerful in establishing likely transmission trees, refuting incorrect assumptions about transmission events and identifying likely risk constellations. It is possible that SARS-CoV-2 transmission may have occurred from a COVID-19 patient to HCW despite the correct use of personal protective equipment, for example during aerosol-generating procedures including NIV. NIV was only later recognised as a potential high-risk procedure in the context of COVID-19, although the true risk level still remains debated [24,25]. Of note, the premises where the aerosol-producing procedures, i.e. NIV therapy and intubation, had been performed were not equipped with negative pressure systems and were poorly ventilated, thus potentially contributing to increased aerosol concentrations. However, the self-reported correct adherence to IPC cannot be confirmed retrospectively, and suboptimal hygiene precautions may also have caused transmissions in the turbulent early days of the pandemic.

NGS has been employed previously to investigate outbreaks of other pathogens such as multidrug-resistant bacteria and recently also for nosocomial SARS-CoV-2 outbreaks [26,27]. NGS using the ARTIC protocol allows rapid sequencing and phylogenetic analysis of isolates from clinical specimens with intermediate to high concentrations of viral RNA [28]. As exemplified here, in addition to comparing the assembled consensus sequence for each case it can be informative to compare the presence of shared minority variants between presumed transmission pairs since either the minority or majority variant can be transmitted to a new host. Of note, we did not observe co-transmission of mixed genotypes as reported by others [29] but in our analysis of transmission pairs, only one of the detected polymorphic variants was detected in the new host. This is consistent with low levels of shared viral diversity observed between transmission pairs in a larger study [30], but may also be due to the use of the amplicon-based sequencing approach applied in our study, which is not the optimal method to adequately reflect intra-host viral diversity.

Our study has a few important limitations. The information we obtained about case and contact histories was extracted semi-automatically as well as manually from medical records and supported by directed interviews. To facilitate future investigations of outbreak scenarios, structured standardised interviews and contact tracing should ideally be performed, enhanced by machine learning methods such as contact prediction via interaction network analysis, tightly integrating documented interactions and genetic evidence. This is currently in development in a nationwide collaborative project as part of the medical informatics initiative. This initiative aims at the development of cross-institutional infrastructure and data-sharing effort by the German Ministry of Research and Education. In addition, spatio-temporal tracing of interactions between HCW and patients could be supported by proximity detection methods using contact tracing apps on mobile phones, although privacy protection remains a major concern for such approaches. Our analysis was limited to cases that presented to our healthcare institution and other sequence data available at GISAID. We realise that the number of available sequences from Germany, especially southern Germany, was low during the investigated time period. We have incorporated all available GISAID sequences from Germany in the analysis during that period. Given the low genetic divergence and incomplete sampling in the early phase of the pandemic, the validity of inferring of transmission and geographical origins of introductions is limited [31]. Interpretation of phylogenies needs to be done carefully in this setting and transmission from other sources cannot be ruled out. Even given the combination of documented high-risk interactions with identical sequences between the index case and infected contact, it cannot be ultimately excluded that transmission occurred from another source.

## Conclusion

We demonstrate how the combination of case histories and genomic data can be integrated to resolve the spread of SARS-CoV-2 on a global, regional and local level. These findings can be used to implement and adjust containment strategies at the population and institutional level.

## Supplementary Data

3295000 Supplement
