
JOURNAL INFORMATION
==============================
NLM Title Abbreviation: Euro Surveill
Journal ID: Euro Surveill
NLM Journal ID: 100887452
Journal ID: eurosurveillance

Eurosurveillance

ISSN: 1025-496X
EISSN: 1560-7917
Publisher: European Centre for Disease Prevention and Control

ARTICLE INFORMATION
==============================
PMCID: PMC8619875
PMID: 34823640
DOI: 10.2807/1560-7917.ES.2021.26.47.211125c
Article ID: 211125c
Article ID: 211125c
Article version: 1
Subjects: Author's Correction

Authors’ correction for Euro Surveill. 2021;26(43)

Eurosurveillance editorial team 1
1 European Centre for Disease Prevention and Control (ECDC), Stockholm, Sweden
Correspondence: Eurosurveillance editorial team (eurosurveillance@ecdc.europa.eu)

Print publication date: 2021 Nov 25
Volume: 26
Issue: 47
Electronic Location ID: 211125c
Copyright: This article is copyright of the authors or their affiliated institutions, 2021.
Copyright year: 2021
Copyright holder: The authors or their affiliated institutions
License: This is an open-access article distributed under the terms of the Creative Commons Attribution (CC BY 4.0) Licence. You may share and adapt the material, but must give appropriate credit to the source, provide a link to the licence, and indicate if changes were made.
License URL: https://creativecommons.org/licenses/by/4.0/
Erratum for: Genomic epidemiology reveals multiple introductions of SARS-CoV-2 followed by community and nosocomial spread, Germany, February to May 2020 26 43 28 10 2021 2002066 Eurosurveillance 10.2807/1560-7917.ES.2021.26.43.2002066 PMC8555370 34713795
PubMedReplaces: True
sequence: 6

==============================
In the article ‘Genomic epidemiology reveals multiple introductions of SARS-CoV-2 followed by community and nosocomial spread, Germany, February to May 2020’ by Muenchhoff et al. published on 28 October 2021, the following changes were made on request of the authors: Information on funding from the Bavarian research network Bay-VOC and LMUexcellent was added; the name of author Katrin Milger-Kneidinger was changed to Katrin Milger; middle names were added for several authors; all affiliations were reformatted to match the institutional standard. These corrections were published on 22 November 2021.

