# Supplementary material for: Genomic epidemiology reveals multiple introductions of SARS-CoV-2 followed by community and nosocomial spread, Germany, February to May 2020
Source: Euro Surveill. 2021 Oct 28;26(43):2002066. doi: 10.2807/1560-7917.ES.2021.26.43.2002066 (PMC8555370; doi:10.2807/1560-7917.ES.2021.26.43.2002066)
Supplement: Supplement [file 20-02066_MUENCHHOFF_Supplement.pdf]

Supplementary Material

This supplementary material is hosted by Eurosurveillance as supporting information alongside the article "Genomic epidemiology reveals multiple introductions followed by community and nosocomial spread of SARS-CoV-2 in Bavaria, February to May 2020", on behalf of the authors, who remain responsible for the accuracy and appropriateness of the content. The same standards for ethics, copyright, attributions and permissions as for the article apply. Supplements are not edited by Eurosurveillance and the journal is not responsible for the maintenance of any links or email addresses provided therein.

Supplementary Figure S1. Divergence of branch of the phylogeny shown in Figure 2 panel A
First travel associated cases are indicated by skiing icons in relation to sequences from Italy, Austria and the rest of Germany (light blue) in a maximum likelihood phylogenetic tree.

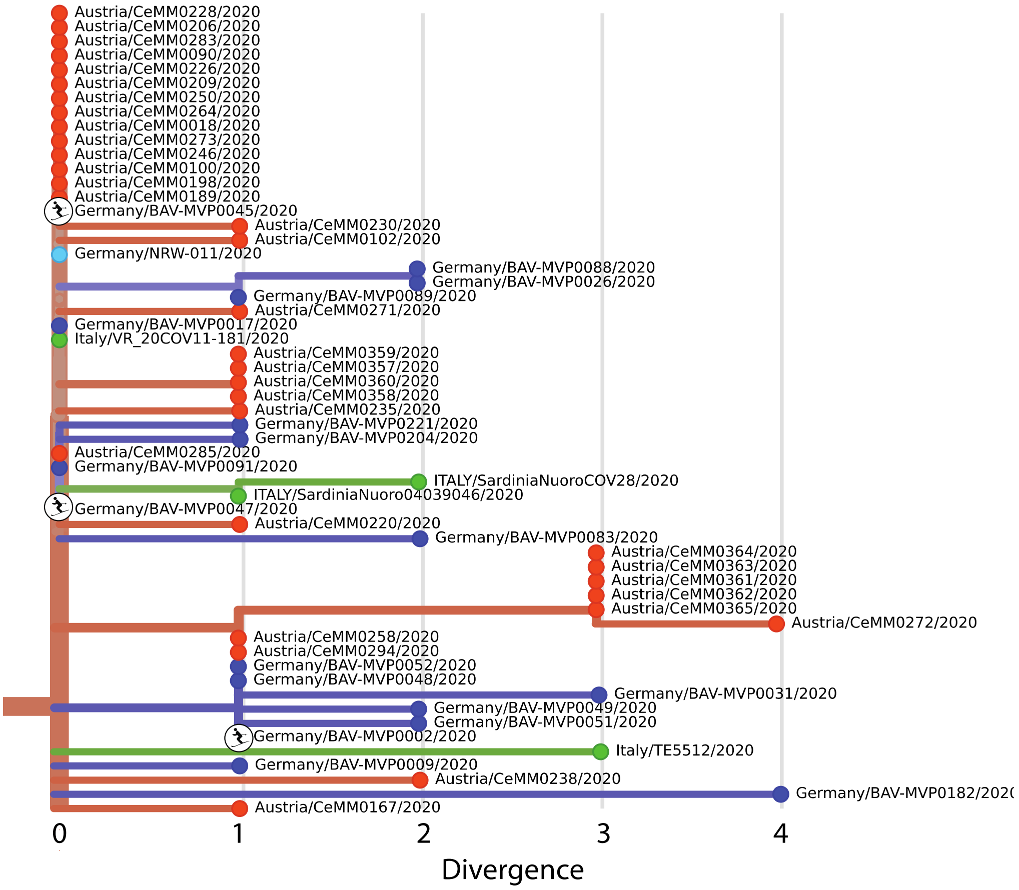

Supplementary Figure S2. Divergence of branch of the phylogeny shown in Figure 2 panel B
First travel associated cases are indicated by skiing icons in relation to sequences from Italy, Austria and the rest of Germany (light blue) in a maximum likelihood phylogenetic tree.

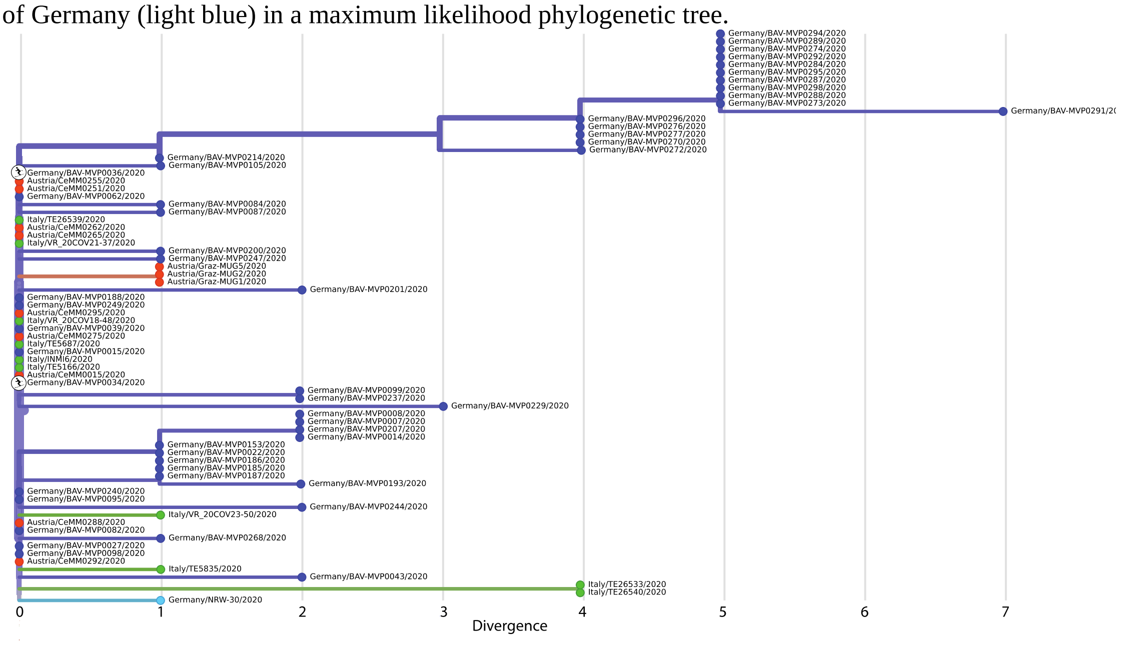

Supplementary Figure S3. Divergence of branch of the phylogeny shown in Figure 2 panel C
First travel associated cases are indicated by skiing icons in relation to sequences from Italy, Austria and the rest of Germany (light blue) in a maximum likelihood phylogenetic tree.

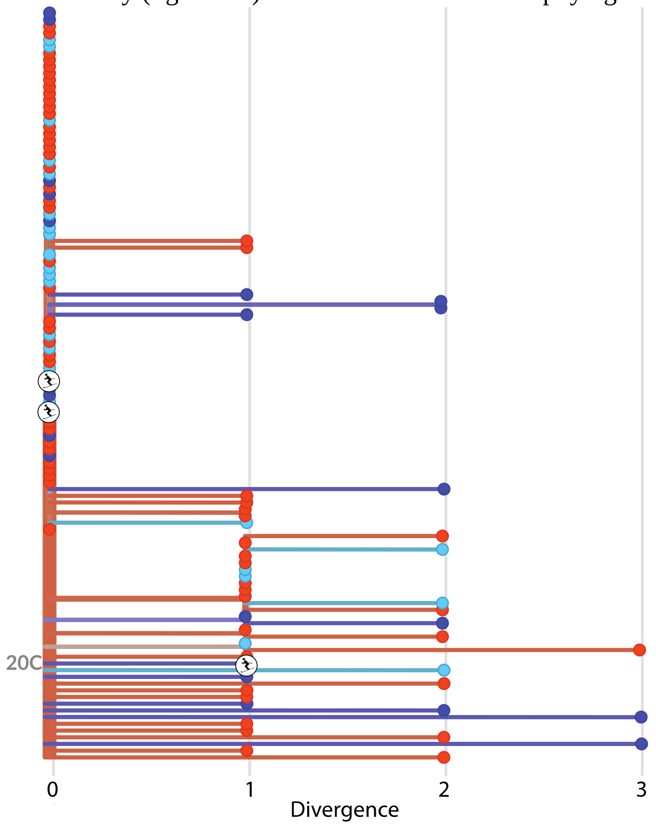

Supplementary Figure S4. Phylogenetic relationship of SARS-CoV-2 genomes from Germany, Austria, Italy, France and Switzerland

The 174 SARS-CoV-2 genomes obtained in this study (dark blue circles with red border) are shown in relation to subsampled sequences deposited at GISAID from Italy (green, n=136), Austria (red, n=250), France (orange, n=297), Switzerland (n=486) and the rest of Germany (light blue, n=78) in a time-resolved maximum likelihood phylogenetic tree.

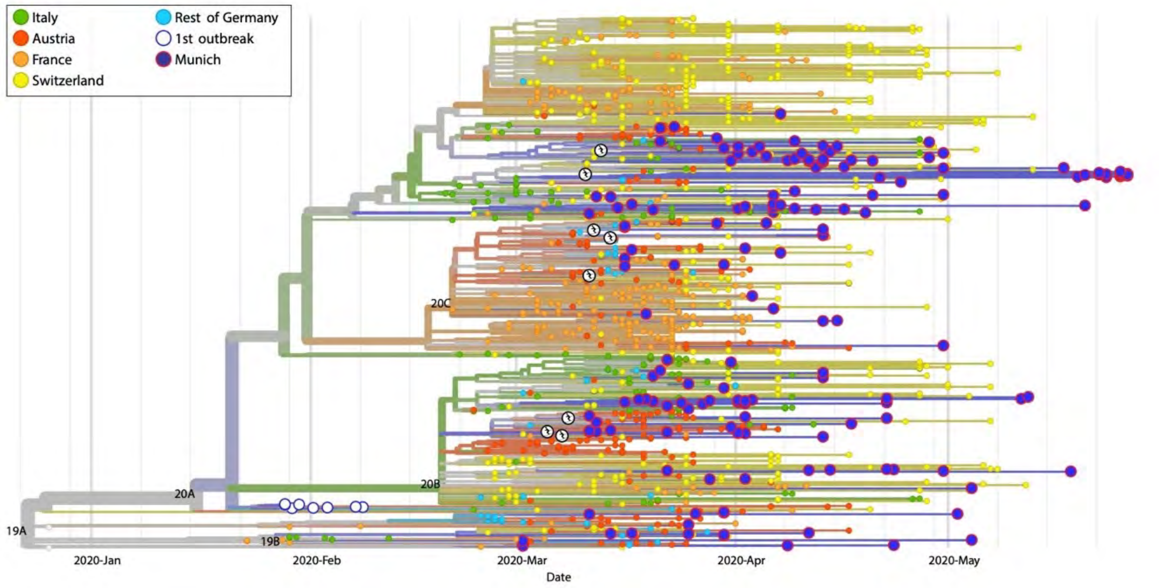

Supplementary Table S1. Synopsis of Risk Category I, II and III contact classification

Category I	Category II	Category III
Person with >= 15 Min. face-to-face contact (<=1,5m)	People with < 15 Min. face-to-face contact (cumulative)	Staff <=1.5 m, with adequate protective clothing
Probably relevant aerosol exposure, even at a greater distance from the index case than 1,5 m	Probably NOT relevant aerosol exposure in the room (>1.5 m from the index case)	Staff >1.5 m, without adequate protective clothing, without direct contact with secretions or excretions of the patient and without aerosol exposure
Direct contact with secretions	Aircraft: within 2 rows in front/behind, but not Kat I	Contact of <=1.5 m when wearing medical MNS in both staff and MNS/MNB in patients without relevant aerosol production

Aircraft: direct seat neighbour	Index case and contact person wear MNS or an MNB consistently and correctly in situations where 1.5 m minimum distance could not be observed	
Medical staff <= 1.5 m, without adequate protective clothing		
Medical staff >1.5 m, without adequate protective clothing with direct contact with secretions or excretions of the patient or in case of possible aerosol exposure		

Supplementary Methods

SARS-CoV-2 whole-genome-sequencing

Amplicon pools from SARS-CoV-2 genomes were generated according to the ARTIC network nCoV-2019 sequencing protocol v2. cDNA was synthesized from isolated total RNA, amplicons were generated by two multiplex PCR reactions, pooled, diluted and quantified by Qubit DNA HS kit.

For nanopore sequencing 50 ng of the amplicon pool was end-repaired, barcoded and ligated to the nanopore sequencing adapter following the ARTIC network nCoV-2019 sequencing protocol v2. Barcoded pools of 24 samples were sequenced on a MinION R9.4.1 flowcell.

For Illumina sequencing amplicon pools were diluted to 0.2 ng/ul and tagmented with nextera XT library prep kit (Illumina, USA). Nextera libraries were dual-barcoded and sequenced on an Illumina Hiseq 1500 instrument.

Sequence analysis using Nanopore sequencing

The Nanopore sequenced amplicons were assembled using the Artic bioinformatics protocol (http://artic.network/ncov-2019). Briefly, the amplicon-based reads were basecalled with the Guppy basecaller (v.3.6.0) provided by ONT using the high-accuracy DNA model and samples were demultiplexed according to the protocol. Per sample the reads were filtered by quality and a read length between 380 and 700 nt. Following the protocol, the consensus sequence for each sample was obtained using the '*minion*' subcommand of the a*rtic* tool with default settings. Shortly, the module aligns the reads to the Sars-Cov-2 reference genome sequence (MN908947.3) with minimap2, removes primer sequences and normalizes coverage for time improvements.[1] Variants were called with Nanopolish which were used to build the consensus sequence.[2]

Sequence analysis using Illumina

Amplicon-based reads sequenced with the Illumina Hiseq 1500 sequencer were demultiplexed for each sample and mapped to the Sars-Cov-2 reference genome (MN908947.3) with *bwa-mem*. Consensus sequences were obtained using the *iVar* package.[3] The *iVar* pipeline first takes the mapped reads and trims the primer sequences using a BED file of primer positions converted from the Artic protocol repository. Further, reads were filtered by base quality >20 and a minimal length of 30nt. Pileup files were generated using *samtools*[4] and supplied to the consensus calling package within *iVar* with default settings.[5]

Variant calling

Based on the sequencing technology SNV calling was achieved in the following way. For reads sequenced on the Illumina Hiseq 1500, SNV were called with *bcftools* included in the *samtools* package. The parameters were modified so that no Indels were called and a ploidy of two was used to distinguish mixed populations. For Nanopore sequenced reads SNVs were called using the variant package from *Nanopolish*.[2] Beside default settings, Indels were discarded, the homopolymer caller was enabled, a ploidy of two was used and the minimal distance from alignment end to calculate variants was set to 10 (--min-flanking-sequence=10).

To distinguish minority variants from sequencing errors, minority variants must have a minimal variant frequency of at least 20% to be considered. Sequence logos were generated from the variant frequency calls with the R package *ggseqlogo*.

Phylogenetic analyses

Phylogenetic analyses were accomplished with a local instance of the web and analysis platform Auspice with the SARS-CoV-2 build (https://github.com/nextstrain/ncov) and the

bioinformatic toolkit *augur* (https://github.com/nextstrain/augur).[6] The consensus sequences as well as the meta-data were downloaded from GISAID (accession date 2020/06/06) with a total of 49.237 sequences obtained world-wide. Our sequenced samples were incorporated and all samples were analysed with the provided Snakefile in the repository using subsequent calls to the *augur* toolkit. Briefly, the steps within the Snakefile consist of filtering samples with a consensus sequence length of less than 90%. Further, the samples were aligned to the reference genome sequence (MN908947.3). For the generation of the global phylogenetic tree (as shown in figure 1) the sample size was reduced by subsampling a maximum of 30 samples for each country, year and month. The European phylogenetic tree (figure 2) was subsampled to a maximum of 400 samples for each country, year and month. In both trees all our samples were excluded from subsampling. GISAID accession numbers of the sequences used in figure 1 and figure 2 are summarized in Supplementary table 3 and 4 (appendix pp 9-99). The maximum likelihood tree was calculated with the tool *augur tree* which calls *IQ-TREE* (v.2.0.3). The resulting tree was refined by the toolkit *augur refine*, which uses internally *TreeTime* to reconstruct ancestral sequences and infer a time resolved phylogeny. Clade annotations were assigned using the *augur clades* command according to the Nextstrain classification consisting of currently five major clades. The Pangolin Tool was used on consensus sequences to assign pangolin lineages.

Supplementary references

1. Li H. Minimap2: pairwise alignment for nucleotide sequences. *Bioinformatics* 2018; **34**(18): 3094-100.

2. Loman NJ, Quick J, Simpson JT. A complete bacterial genome assembled de novo using only nanopore sequencing data. *Nat Methods* 2015; **12**(8): 733-5.

3. Grubaugh ND, Gangavarapu K, Quick J, et al. An amplicon-based sequencing framework for accurately measuring intrahost virus diversity using PrimalSeq and iVar. *Genome Biol* 2019; **20**(1): 8.

4. Li H.*, Handsaker B.*, Wysoker A., Fennell T., Ruan J., Homer N., Marth G., Abecasis G., Durbin R. and 1000 Genome Project Data Processing Subgroup (2009) The Sequence alignment/map (SAM) format and SAMtools. Bioinformatics, 25, 2078-9.
5. Li H. A statistical framework for SNP calling, mutation discovery, association mapping and population genetical parameter estimation from sequencing data. *Bioinformatics* 2011; **27**(21): 2987-93.
6. Hadfield J, Megill C, Bell SM, et al. Nextstrain: real-time tracking of pathogen evolution. *Bioinformatics* 2018; **34**(23): 4121-3.

Supplementary Table S2. Metadata and accession numbers of sequenced isolates used for phylogenetic studies.

Collection dates, gender, age, patient/staff status, genome coverage, sequencing technology, cluster analysis in this study, original sample material, country of likely exposure, probable transmission type and GISAID accession numbers are indicated for the 174 samples used for further phylogenetic studies.

collection_date	gender	age	status	coverage	technology	cluster	material	country_exposure	covid19_infection_type	gisaid_accession_nr
2020-03-17	Male	90	Patient	99.65	Illumina HiSeq1500	Figure 4	Nasopharyngeal Swab	Germany	nursing_home_acquired	EPI_ISL_437254
2020-03-07	Female	19	Patient	99.71	Illumina HiSeq1500		Nasopharyngeal Swab	Germany	community_acquired	EPI_ISL_437210
2020-03-23	Male	46	Patient	99.71	Illumina HiSeq1500		Nasopharyngeal Swab	Germany	community_acquired	EPI_ISL_437237
2020-03-13	Female	30	Staff	99.34	Illumina HiSeq1500	Figure 4	Nasopharyngeal Swab	Germany	hospital_acquired	EPI_ISL_437233
2020-03-11	Female	30	Staff	99.74	Illumina HiSeq1500	Figure 4	Nasopharyngeal Swab	Germany	hospital_acquired	EPI_ISL_437246
2020-03-24	Male	85	Patient	99.65	Illumina HiSeq1500	Figure 4	Nasopharyngeal Swab	Germany	hospital_acquired	EPI_ISL_437263
2020-03-11	Male	47	Patient	99.64	Illumina HiSeq1500		Nasopharyngeal Swab	Austria	travel_associated	EPI_ISL_437228
2020-03-14	Male	56	Patient	99.65	Illumina HiSeq1500		Nasopharyngeal Swab	Austria	travel_associated	EPI_ISL_437229
2020-03-24	Male	86	Patient	99.57	Illumina HiSeq1500		Nasopharyngeal Swab	Germany	community_acquired	EPI_ISL_437238
2020-03-11	Female	31	Staff	99.65	Illumina HiSeq1500	Figure 4	Nasopharyngeal Swab	Germany	hospital_acquired	EPI_ISL_437248
2020-03-12	Female	24	Patient	99.65	Illumina HiSeq1500		Nasopharyngeal Swab	Germany	community_acquired	EPI_ISL_437249
2020-03-12	Male	29	Staff	99.65	Illumina HiSeq1500	Figure 4	Nasopharyngeal Swab	Germany	hospital_acquired	EPI_ISL_437250
2020-03-14	Female	29	Staff	99.6	Nanopore MinION	Figure 4	Nasopharyngeal Swab	Germany	hospital_acquired	EPI_ISL_420903
2020-03-14	Male	82	Patient	99.66	Illumina HiSeq1500	Figure 4	Nasopharyngeal Swab	Germany	hospital_acquired	EPI_ISL_437251

2020-03-16	Female	25	Staff	99.57	Illumina HiSeq1500		Nasopharyngeal Swab	Germany	community_acquired	EPI_ISL_437252
2020-04-22	Female	55	Staff	98.78	Illumina HiSeq1500	Figure 4	Nasopharyngeal Swab	Germany	hospital_acquired	EPI_ISL_466892
2020-03-11	Male	65	Patient	99.51	Illumina HiSeq1500		Nasopharyngeal Swab	Germany	community_acquired	EPI_ISL_437245
2020-03-11	Female	34	Staff	99.65	Illumina HiSeq1500	Figure 4	Nasopharyngeal Swab	Germany	hospital_acquired	EPI_ISL_437247
2020-03-11	Male	69	Patient	96.01	Illumina HiSeq1500		Nasopharyngeal Swab		unknown	EPI_ISL_549025
2020-03-25	Male	77	Patient	99.76	Illumina HiSeq1500		Sputum	Germany	community_acquired	EPI_ISL_437264
2020-03-20	Male	31	Patient	99.09	Illumina HiSeq1500		Nasopharyngeal Swab	Germany	community_acquired	EPI_ISL_437257
2020-03-16	Male	47	Staff	99.02	Nanopore MinION		Nasopharyngeal Swab	Germany	hospital_acquired	EPI_ISL_420906
2020-03-19	Female	35	Staff	97.26	Nanopore MinION		Nasopharyngeal Swab	Germany	hospital_acquired	EPI_ISL_420909
2020-04-13	Female	80	Patient	93.9	Illumina HiSeq1500		Bronchoalveolar Lavage Fluid	Germany	community_acquired	EPI_ISL_548950
2020-03-30	Female	68	Patient	99.6	Illumina HiSeq1500		Endotracheal Aspirate	Germany	community_acquired	EPI_ISL_437276
2020-03-14	Male	38	Patient	89.68	Illumina HiSeq1500		Nasopharyngeal Swab	Germany	community_acquired	EPI_ISL_548951
2020-03-16	Male	33	Staff	99.66	Illumina HiSeq1500		Nasopharyngeal Swab	Germany	hospital_acquired	EPI_ISL_437205
2020-04-02	Male	78	Patient	99.63	Illumina HiSeq1500		Nasopharyngeal Swab	Germany	community_acquired	EPI_ISL_437239
2020-03-19	Female	38	Patient	99.65	Illumina HiSeq1500		Nasopharyngeal Swab	Germany	community_acquired	EPI_ISL_437255
2020-03-17	Male	48	Patient	98.82	Illumina HiSeq1500		Nasopharyngeal Swab		unknown	EPI_ISL_451937
2020-03-16	Female	54	Staff	99.02	Nanopore MinION		Nasopharyngeal Swab	Germany	hospital_acquired	EPI_ISL_420907
2020-04-02	Male	63	Patient	99.59	Illumina HiSeq1500		Endotracheal Aspirate	Switzerland	travel_associated	EPI_ISL_437284
2020-05-04	Male	56	Patient	99.61	Illumina HiSeq1500		Nasopharyngeal Swab	Austria	travel_associated	EPI_ISL_466906
2020-03-20	Male	32	Staff	97.93	Illumina HiSeq1500		Nasopharyngeal Swab	Germany	hospital_acquired	EPI_ISL_451939
2020-04-06	Male	70	Patient	97.66	Illumina HiSeq1500		Endotracheal Aspirate	Germany	community_acquired	EPI_ISL_466874
2020-03-22	Female	35	Patient	99.66	Illumina HiSeq1500		Nasopharyngeal Swab	Germany	community_acquired	EPI_ISL_437212
2020-03-22	Female	20	Patient	99.15	Illumina HiSeq1500		Sputum	Germany	community_acquired	EPI_ISL_437236

2020-03-17	Male	48	Patient	99.65	Illumina HiSeq1500		Nasopharyngeal Swab		unknown	EPI_ISL_437253
2020-04-03	Female	34	Patient	99.65	Illumina HiSeq1500		Nasopharyngeal Swab	Germany	community_acquired	EPI_ISL_437288
2020-03-21	Female	60	Patient	98.98	Illumina HiSeq1500		Nasopharyngeal Swab	Germany	community_acquired	EPI_ISL_437259
2020-04-02	Male	59	Staff	98.6	Illumina HiSeq1500		Sputum		unknown	EPI_ISL_451946
2020-03-23	Male	46	Staff	99.65	Illumina HiSeq1500		Nasopharyngeal Swab	Germany	unknown	EPI_ISL_451941
2020-03-22	Male	46	Patient	99.46	Illumina HiSeq1500		Nasopharyngeal Swab	Germany	community_acquired	EPI_ISL_437262
2020-03-30	Male	80	Patient	96.44	Illumina HiSeq1500		Nasopharyngeal Swab	Germany	community_acquired	EPI_ISL_451943
2020-03-31	Male	63	Patient	99.65	Illumina HiSeq1500		Endotracheal Aspirate	Germany	community_acquired	EPI_ISL_437232
2020-03-28	Male	76	Patient	99.52	Illumina HiSeq1500		Nasopharyngeal Swab	Germany	community_acquired	EPI_ISL_437268
2020-03-29	Male	43	Patient	99.11	Illumina HiSeq1500		Nasopharyngeal Swab	Germany	community_acquired	EPI_ISL_437270
2020-04-02	Male	69	Patient	99.32	Illumina HiSeq1500		Endotracheal Aspirate	Germany	unknown	EPI_ISL_437285
2020-03-30	Male	59	Patient	99.19	Illumina HiSeq1500		Endotracheal Aspirate	Germany	community_acquired	EPI_ISL_437273
2020-03-22	Female	50	Patient	99.72	Illumina HiSeq1500		Nasopharyngeal Swab		unknown	EPI_ISL_437260
2020-03-25	Female	91	Patient	99.65	Illumina HiSeq1500	Figure 4	Nasopharyngeal Swab	Germany	nursing_home_acquired	EPI_ISL_437231
2020-03-25	Female	92	Patient	98.78	Illumina HiSeq1500		Nasopharyngeal Swab	Germany	community_acquired	EPI_ISL_437265
2020-04-05	Male	86	Patient	99.59	Nanopore MinION		Nasopharyngeal Swab	Germany	hospital_acquired	EPI_ISL_437213
2020-03-30	Male	74	Patient	96.45	Illumina HiSeq1500		Nasopharyngeal Swab	Germany	community_acquired	EPI_ISL_451942
2020-03-28	Female	69	Patient	99.13	Illumina HiSeq1500		Nasopharyngeal Swab	Germany	community_acquired	EPI_ISL_437269
2020-04-01	Female	27	Patient	99.65	Illumina HiSeq1500		Nasopharyngeal Swab	Germany	community_acquired	EPI_ISL_437278
2020-04-01	Male	61	Patient	99.54	Illumina HiSeq1500		Nasopharyngeal Swab	Germany	community_acquired	EPI_ISL_437281
2020-04-01	Male	72	Patient	98.69	Illumina HiSeq1500		Nasopharyngeal Swab	Germany	hospital_acquired	EPI_ISL_451945
2020-04-02	Male	59	Patient	99.54	Illumina HiSeq1500		Nasopharyngeal Swab	Germany	community_acquired	EPI_ISL_437283
2020-03-25	Male	32	Patient	99.56	Illumina HiSeq1500		Nasopharyngeal Swab	Germany	community_acquired	EPI_ISL_452103
2020-04-01	Female	28	Patient	99.65	Illumina HiSeq1500		Nasopharyngeal Swab	Germany	community_acquired	EPI_ISL_437225

2020-04-03	Male	51	Staff	99.06	Illumina HiSeq1500		Nasopharyngeal Swab	Germany	hospital_acquired	EPI_ISL_437289
2020-03-23	Male	29	Patient	99.28	Illumina HiSeq1500		Nasopharyngeal Swab	Germany	community_acquired	EPI_ISL_451940
2020-03-31	Male	59	Patient	98.34	Illumina HiSeq1500		Nasopharyngeal Swab	Germany	community_acquired	EPI_ISL_451944
2020-04-04	Male	63	Staff	99.19	Illumina HiSeq1500		Nasopharyngeal Swab	Germany	community_acquired	EPI_ISL_452104
2020-04-13	Male	69	Patient	99.65	Illumina HiSeq1500		Endotracheal Aspirate	Germany	community_acquired	EPI_ISL_437227
2020-04-08	Male	18	Patient	97.42	Illumina HiSeq1500		Nasopharyngeal Swab	Germany	community_acquired	EPI_ISL_451947
2020-04-09	Female	75	Patient	96.18	Illumina HiSeq1500		Endotracheal Aspirate	Germany	community_acquired	EPI_ISL_437293
2020-03-29	Male	43	Staff	99.62	Illumina HiSeq1500		Nasopharyngeal Swab		unknown	EPI_ISL_437224
2020-04-07	Female	75	Patient	99.16	Illumina HiSeq1500		Nasopharyngeal Swab	Germany	community_acquired	EPI_ISL_437292
2020-04-11	Male	63	Patient	99.65	Illumina HiSeq1500		Nasopharyngeal Swab	Germany	community_acquired	EPI_ISL_437243
2020-03-26	Male	74	Patient	99.65	Illumina HiSeq1500		Nasopharyngeal Swab	Germany	community_acquired	EPI_ISL_437266
2020-04-01	Male	56	Patient	99.71	Illumina HiSeq1500	Figure 4	Nasopharyngeal Swab	Germany	hospital_acquired	EPI_ISL_437280
2020-04-01	Male	0	Patient	99.65	Illumina HiSeq1500		Nasopharyngeal Swab	Germany	community_acquired	EPI_ISL_437282
2020-04-05	Male	64	Patient	99.65	Illumina HiSeq1500		Nasopharyngeal Swab	Germany	community_acquired	EPI_ISL_437290
2020-04-03	Male	59	Patient	99.1	Illumina HiSeq1500		Nasopharyngeal Swab	Germany	community_acquired	EPI_ISL_437287
2020-04-20	Male	84	Patient	99.6	Illumina HiSeq1500		Nasopharyngeal Swab	Germany	community_acquired	EPI_ISL_466888
2020-04-12	Male	79	Patient	98.86	Illumina HiSeq1500		Nasopharyngeal Swab	Germany	nursing_home_acquired	EPI_ISL_437295
2020-04-11	Male	54	Patient	99.66	Illumina HiSeq1500		Nasopharyngeal Swab	Germany	hospital_acquired	EPI_ISL_437217
2020-04-11	Female	70	Patient	99.24	Illumina HiSeq1500		Nasopharyngeal Swab	Germany	hospital_acquired	EPI_ISL_437222
2020-04-08	Female	67	Patient	99.63	Illumina HiSeq1500		Nasopharyngeal Swab	Germany	community_acquired	EPI_ISL_437215
2020-04-10	Male	81	Patient	98.73	Illumina HiSeq1500		Nasopharyngeal Swab	Germany	community_acquired	EPI_ISL_437226
2020-04-07	Female	70	Patient	99.64	Illumina HiSeq1500		Nasopharyngeal Swab	Germany	community_acquired	EPI_ISL_437241
2020-04-07	Male	81	Patient	99.65	Illumina HiSeq1500		Nasopharyngeal Swab	Germany	community_acquired	EPI_ISL_437214
2020-04-11	Female	86	Patient	99.6	Nanopore MinION		Nasopharyngeal Swab	Germany	hospital_acquired	EPI_ISL_437208

2020-04-16	Male	71	Patient	99.68	Illumina HiSeq1500		Endotracheal Aspirate	Germany	community_acquired	EPI_ISL_466885
2020-04-13	Male	30	Patient	99.64	Illumina HiSeq1500		Nasopharyngeal Swab	Germany	community_acquired	EPI_ISL_466878
2020-04-14	Female	71	Patient	99.46	Illumina HiSeq1500		Endotracheal Aspirate	Germany	hospital_acquired	EPI_ISL_466881
2020-04-17	Female	48	Patient	98.08	Illumina HiSeq1500		Nasopharyngeal Swab	Germany	community_acquired	EPI_ISL_466886
2020-04-15	Male	88	Patient	99.53	Illumina HiSeq1500		Nasopharyngeal Swab	Germany	community_acquired	EPI_ISL_466883
2020-04-17	Female	70	Patient	97.92	Illumina HiSeq1500		Nasopharyngeal Swab	Germany	community_acquired	EPI_ISL_466887
2020-04-12	Male	55	Patient	94.46	Illumina HiSeq1500		Nasopharyngeal Swab	Germany	community_acquired	EPI_ISL_548952
2020-04-09	Female	37	Patient	99.62	Illumina HiSeq1500		Nasopharyngeal Swab	Germany	community_acquired	EPI_ISL_437207
2020-04-13	Male	23	Patient	99.56	Illumina HiSeq1500		Nasopharyngeal Swab	Germany	community_acquired	EPI_ISL_437297
2020-04-13	Female	77	Patient	96.23	Illumina HiSeq1500		Nasopharyngeal Swab	Germany	nursing_home_acquired	EPI_ISL_466876
2020-04-13	Male	23	Staff	99.49	Illumina HiSeq1500		Nasopharyngeal Swab	Germany	community_acquired	EPI_ISL_466877
2020-04-13	Male	96	Patient	98.59	Illumina HiSeq1500		Nasopharyngeal Swab	Germany	nursing_home_acquired	EPI_ISL_466879
2020-04-15	Female	95	Patient	98.97	Illumina HiSeq1500		Nasopharyngeal Swab	Germany	community_acquired	EPI_ISL_466882
2020-04-16	Female	57	Patient	99.08	Illumina HiSeq1500		Endotracheal Aspirate	Germany	community_acquired	EPI_ISL_466884
2020-04-22	Male	47	Staff	99.65	Illumina HiSeq1500		Nasopharyngeal Swab	Germany	hospital_acquired	EPI_ISL_466891
2020-04-22	Male	79	Patient	97.82	Illumina HiSeq1500		Nasopharyngeal Swab	Germany	community_acquired	EPI_ISL_466894
2020-04-23	Male	27	Patient	99.15	Illumina HiSeq1500		Nasopharyngeal Swab	Germany	community_acquired	EPI_ISL_466895
2020-04-30	Male	79	Patient	99.5	Illumina HiSeq1500		Endotracheal Aspirate	Germany	community_acquired	EPI_ISL_466901
2020-04-14	Male	60	Patient	92.37	Illumina HiSeq1500		Nasopharyngeal Swab	Germany	community_acquired	EPI_ISL_548953
2020-04-30	Male	62	Patient	99.72	Illumina HiSeq1500		Nasopharyngeal Swab	Germany	community_acquired	EPI_ISL_466904
2020-04-13	Male	36	Staff	99.6	Illumina HiSeq1500		Nasopharyngeal Swab	Germany	hospital_acquired	EPI_ISL_466875
2020-04-24	Female	71	Patient	99.29	Illumina HiSeq1500		Nasopharyngeal Swab	Germany	community_acquired	EPI_ISL_466897
2020-04-13	Female	85	Patient	99.65	Illumina HiSeq1500		Nasopharyngeal Swab	Germany	community_acquired	EPI_ISL_437209
2020-05-02	Male	73	Patient	99.63	Illumina HiSeq1500		Nasopharyngeal Swab	Germany	community_acquired	EPI_ISL_466905

2020-04-21	Male	51	Patient	89.98	Illumina HiSeq1500		Nasopharyngeal Swab	Germany	community_acquired	EPI_ISL_548955
2020-04-19	Male	61	Patient	99.39	Illumina HiSeq1500		Nasopharyngeal Swab	Germany	community_acquired	EPI_ISL_466889
2020-04-20	Female	59	Staff	97.19	Illumina HiSeq1500		Nasopharyngeal Swab	Germany	hospital_acquired	EPI_ISL_466890
2020-04-28	Male	56	Patient	98.86	Illumina HiSeq1500		Nasopharyngeal Swab	Germany	community_acquired	EPI_ISL_466899
2020-04-30	Male	72	Patient	98.75	Illumina HiSeq1500		Nasopharyngeal Swab	Germany	community_acquired	EPI_ISL_466900
2020-04-30	Female	26	Staff	99.81	Illumina HiSeq1500		Nasopharyngeal Swab	Germany	unknown	EPI_ISL_466902
2020-05-22	Male	80	Patient	97.14	Nanopore MinION		Nasopharyngeal Swab	Germany	hospital_acquired	EPI_ISL_466915
2020-05-22	Female	89	Patient	99.43	Nanopore MinION		Nasopharyngeal Swab	Germany	hospital_acquired	EPI_ISL_466916
2020-05-22	Female	26	Staff	91.71	Nanopore MinION		Nasopharyngeal Swab	Germany	hospital_acquired	EPI_ISL_548959
2020-04-28	Male	42	Staff	97.54	Illumina HiSeq1500		Nasopharyngeal Swab	Germany	unknown	EPI_ISL_466898
2020-05-04	Female	75	Patient	99.7	Illumina HiSeq1500		Endotracheal Aspirate	Germany	community_acquired	EPI_ISL_466907
2020-05-17	Female	63	Patient	99.01	Nanopore MinION		Endotracheal Aspirate	Germany	community_acquired	EPI_ISL_466910
2020-05-18	Male	65	Patient	98.28	Nanopore MinION		Nasopharyngeal Swab	Germany	nursing_home_acquired	EPI_ISL_466911
2020-05-23	Female	75	Patient	99.01	Nanopore MinION		Nasopharyngeal Swab	Germany	community_acquired	EPI_ISL_466918
2020-05-20	Female	41	Staff	98.09	Nanopore MinION		Nasopharyngeal Swab	Germany	hospital_acquired	EPI_ISL_466913
2020-03-16	Female	42	Staff	98.95	Nanopore MinION		Nasopharyngeal Swab	Germany	hospital_acquired	EPI_ISL_420905
2020-05-26	Male	35	Staff	99.59	Nanopore MinION		Nasopharyngeal Swab	Germany	hospital_acquired	EPI_ISL_466925
2020-05-26	Male	47	Staff	95.3	Nanopore MinION		Nasopharyngeal Swab	Germany	hospital_acquired	EPI_ISL_548958
2020-03-05	Male	64	Patient	99.64	Illumina HiSeq1500	Figure 4	Endotracheal Aspirate	Italy	travel_associated	EPI_ISL_437204
2020-05-20	Male	81	Patient	97.29	Nanopore MinION		Nasopharyngeal Swab	Germany	unknown	EPI_ISL_466914
2020-05-25	Male	57	Patient	99.01	Nanopore		Nasopharyngeal Swab	Germany	community_acquired	EPI_ISL_466921

					MinION					
2020-05-25	Female	35	Staff	99.59	Nanopore MinION		Nasopharyngeal Swab	Germany	hospital_acquired	EPI_ISL_466922
2020-05-25	Female	29	Staff	99.58	Nanopore MinION		Nasopharyngeal Swab	Germany	hospital_acquired	EPI_ISL_466923
2020-05-11	Female	72	Patient	99.01	Nanopore MinION		Endotracheal Aspirate	Germany	community_acquired	EPI_ISL_466909
2020-05-25	Female	28	Staff	94.37	Nanopore MinION		Nasopharyngeal Swab	Germany	hospital_acquired	EPI_ISL_548957
2020-05-12	Male	60	Patient	91.84	Nanopore MinION		Endotracheal Aspirate	Germany	community_acquired	EPI_ISL_548956
2020-05-25	Female	79	Patient	99.01	Nanopore MinION		Nasopharyngeal Swab	Germany	hospital_acquired	EPI_ISL_466920
2020-03-16	Male	54	Staff	89.91	Nanopore MinION		Nasopharyngeal Swab	Germany	hospital_acquired	EPI_ISL_548961
2020-04-09	Male	75	Patient	98.71	Illumina HiSeq1500		Nasopharyngeal Swab	Germany	community_acquired	EPI_ISL_437242
2020-03-22	Male	54	Patient	99.5	Illumina HiSeq1500		Nasopharyngeal Swab	Germany	community_acquired	EPI_ISL_437261
2020-03-30	Male	81	Patient	99.66	Illumina HiSeq1500		Endotracheal Aspirate	Germany	community_acquired	EPI_ISL_437275
2020-03-31	Male	3	Patient	99.65	Illumina HiSeq1500		Nasopharyngeal Swab	Germany	community_acquired	EPI_ISL_437277
2020-04-13	Male	80	Patient	99.65	Illumina HiSeq1500		Endotracheal Aspirate	Germany	community_acquired	EPI_ISL_437296
2020-05-25	Female	70	Patient	99.01	Nanopore MinION		Nasopharyngeal Swab	Germany	hospital_acquired	EPI_ISL_466919
2020-04-02	Female	44	Patient	98.82	Illumina HiSeq1500		Nasopharyngeal Swab	Germany	community_acquired	EPI_ISL_437286
2020-04-17	Male	81	Patient	94.8	Illumina HiSeq1500		Nasopharyngeal Swab	Germany	community_acquired	EPI_ISL_548954
2020-03-22	Male	23	Patient	99.6	Illumina HiSeq1500		Nasopharyngeal Swab	Germany	community_acquired	EPI_ISL_437230
2020-03-20	Female	47	Staff	99.7	Illumina HiSeq1500		Nasopharyngeal Swab	Germany	community_acquired	EPI_ISL_437258
2020-03-30	Male	64	Patient	99.05	Illumina HiSeq1500		Nasopharyngeal Swab	Germany	community_acquired	EPI_ISL_437271
2020-03-30	Male	57	Patient	99.26	Illumina HiSeq1500		Endotracheal Aspirate	Germany	community_acquired	EPI_ISL_437274
2020-05-23	Male	48	Patient	99.01	Nanopore MinION		Nasopharyngeal Swab	Germany	community_acquired	EPI_ISL_466917
2020-03-21	Male	52	Patient	99.6	Nanopore		Endotracheal Aspirate		unknown	EPI_ISL_420912

					MinION					
2020-04-01	Female	73	Patient	99.63	Illumina HiSeq1500		Nasopharyngeal Swab	Germany	community_acquired	EPI_ISL_437279
2020-03-27	Male	77	Patient	99.75	Illumina HiSeq1500		Nasopharyngeal Swab	Germany	hospital_acquired	EPI_ISL_437267
2020-03-12	Male	29	Staff	99.65	Illumina HiSeq1500	Figure 4	Nasopharyngeal Swab	Austria	travel_associated	EPI_ISL_437211
2020-03-21	Male	55	Patient	99.37	Illumina HiSeq1500		Sputum	Italy	community_acquired	EPI_ISL_437235
2020-03-08	Male	56	Patient	99.73	Illumina HiSeq1500		Nasopharyngeal Swab	Italy	travel_associated	EPI_ISL_437244
2020-03-30	Female	33	Patient	99.61	Illumina HiSeq1500		Nasopharyngeal Swab	Germany	community_acquired	EPI_ISL_437272
2020-03-12	Female	22	Staff	99.7	Illumina HiSeq1500	Figure 4	Nasopharyngeal Swab	Germany	hospital_acquired	EPI_ISL_437218
2020-04-06	Male	61	Patient	99.66	Illumina HiSeq1500		Endotracheal Aspirate	Germany	community_acquired	EPI_ISL_437221
2020-03-20	Male	60	Patient	99.55	Illumina HiSeq1500		Nasopharyngeal Swab	Germany	hospital_acquired	EPI_ISL_437256
2020-03-18	Male	54	Staff	99.66	Illumina HiSeq1500		Nasopharyngeal Swab	Germany	community_acquired	EPI_ISL_437206
2020-04-09	Male	65	Patient	99.66	Illumina HiSeq1500		Endotracheal Aspirate	Germany	community_acquired	EPI_ISL_437216
2020-04-06	Female	41	Patient	99.65	Illumina HiSeq1500		Sputum	Germany	community_acquired	EPI_ISL_437220
2020-03-12	Male	49	Patient	99.65	Illumina HiSeq1500		Nasopharyngeal Swab	Austria	travel_associated	EPI_ISL_437223
2020-04-06	Male	72	Patient	97.08	Illumina HiSeq1500		Endotracheal Aspirate	Germany	community_acquired	EPI_ISL_437240
2020-04-23	Female	19	Patient	99.45	Illumina HiSeq1500		Nasopharyngeal Swab	Germany	community_acquired	EPI_ISL_466896
2020-04-30	Male	43	Patient	99.33	Illumina HiSeq1500		Nasopharyngeal Swab	Germany	hospital_acquired	EPI_ISL_466903
2020-05-19	Male	29	Patient	98.01	Nanopore MinION		Nasopharyngeal Swab	Germany	community_acquired	EPI_ISL_466912
2020-05-26	Female	46	Staff	99.59	Nanopore MinION		Nasopharyngeal Swab	Germany	hospital_acquired	EPI_ISL_466924
2020-04-02	Female	60	Patient	99.56	Illumina HiSeq1500		Nasopharyngeal Swab	Germany	community_acquired	EPI_ISL_437219
2020-03-15	Male	63	Patient	99.65	Illumina HiSeq1500		Nasopharyngeal Swab	Germany	community_acquired	EPI_ISL_437234
2020-04-06	Male	63	Patient	99.65	Illumina HiSeq1500		Nasopharyngeal Swab	Germany	community_acquired	EPI_ISL_437291
2020-04-13	Male	62	Patient	99.66	Illumina HiSeq1500		Nasopharyngeal Swab	Germany	hospital_acquired	EPI_ISL_466880
2020-04-22	Male	51	Staff	98.67	Illumina HiSeq1500		Nasopharyngeal Swab	Germany	community_acquired	EPI_ISL_466893

Supplementary Table S3. Accession numbers and further information of sequences used for the phylogeny shown in figure 1a.

Strain	gisaid_epi_isl	Originating Lab	Submitting Lab	Collection Data	Author
Algeria/G0638_2264/2020	EPI_ISL_418241	NIC Viral Respiratory Unit - Institut Pasteur of Algeria	National Reference Center for Viruses of Respiratory Infections, Institut Pasteur, Paris	2020-03-02	Mélanie Albert et al C
Algeria/G0640_2265/2020	EPI_ISL_418242	NIC Viral Respiratory Unit - Institut Pasteur of Algeria	National Reference Center for Viruses of Respiratory Infections, Institut Pasteur, Paris	2020-03-08	Mélanie Albert et al C
Algeria/G0860_2262/2020	EPI_ISL_420037	NIC Viral Respiratory Unit - Institut Pasteur of Algeria	National Reference Center for Viruses of Respiratory Infections, Institut Pasteur, Paris	2020-03-02	Mélanie Albert et al C
Argentina/C121/2020	EPI_ISL_420600	Servicio Virosis Respiratorias-Departamento Virología-INEI	Instituto Nacional Enfermedades Infecciosas C.G.Malbran	2020-03-07	Baumeister E. et al (https://dx.doi.org/10.1101/2020.05.23.20111443)
Argentina/C1374/2020	EPI_ISL_420599	Servicio Virosis Respiratorias-Departamento Virología-INEI	Instituto Nacional Enfermedades Infecciosas C.G.Malbran	2020-03-18	Baumeister E. et al (https://dx.doi.org/10.1101/2020.05.23.20111443)
Argentina/C3013/2020	EPI_ISL_420598	Servicio Virosis Respiratorias-Departamento Virología-INEI	Instituto Nacional Enfermedades Infecciosas C.G.Malbran	2020-03-22	Baumeister E. et al (https://dx.doi.org/10.1101/2020.05.23.20111443)
Argentina/PAIS_A001/2020	EPI_ISL_430793	Laboratorio Análisis Clínicos, Unidad de Servicios Diagnósticos, Swiss Medical Group	Área de Secuenciación del Laboratorio de Virología del Hospital de Niños Dr. Ricardo Gutierrez	2020-03-01	Nabaes Jodar et al A (https://dx.doi.org/10.1101/2020.05.23.20111443)
Argentina/PAIS_A002/2020	EPI_ISL_430794	Laboratorio Análisis Clínicos, Unidad de Servicios Diagnósticos, Swiss Medical Group	Área de Secuenciación del Laboratorio de Virología del Hospital de Niños Dr. Ricardo Gutierrez	2020-03-24	Nabaes Jodar et al B
Argentina/PAIS_A003/2020	EPI_ISL_430795	Laboratorio de Virología del Hospital de Niños Dr. Ricardo Gutierrez	Área de Secuenciación del Laboratorio de Virología del Hospital de Niños Dr. Ricardo Gutierrez	2020-03-26	Nabaes Jodar et al B
Argentina/PAIS_A004/2020	EPI_ISL_430796	Departamento de Biología y genética molecular, IACA Laboratorios.	Área de Secuenciación del Laboratorio de Virología del Hospital de Niños Dr. Ricardo Gutierrez	2020-03-27	Nabaes Jodar et al A (https://dx.doi.org/10.1101/2020.05.23.20111443)
Argentina/PAIS_A005/2020	EPI_ISL_430797	Departamento de Biología y genética molecular, IACA Laboratorios.	Área de Secuenciación del Laboratorio de Virología del Hospital de Niños Dr. Ricardo Gutierrez	2020-03-27	Nabaes Jodar et al A (https://dx.doi.org/10.1101/2020.05.23.20111443)
Argentina/PAIS_A006/2020	EPI_ISL_430798	Departamento de Biología y genética molecular, IACA Laboratorios.	Área de Secuenciación del Laboratorio de Virología del Hospital de Niños Dr. Ricardo Gutierrez	2020-03-28	Nabaes Jodar et al A (https://dx.doi.org/10.1101/2020.05.23.20111443)
Argentina/PAIS_A007/2020	EPI_ISL_430799	Laboratorio de Virología del Hospital de Niños Dr. Ricardo Gutierrez	Área de Secuenciación del Laboratorio de Virología del Hospital de Niños Dr. Ricardo Gutierrez	2020-03-30	Nabaes Jodar et al A (https://dx.doi.org/10.1101/2020.05.23.20111443)
Argentina/PAIS_A008/2020	EPI_ISL_430800	Laboratorio de Virología del Hospital de Niños Dr. Ricardo Gutierrez	Área de Secuenciación del Laboratorio de Virología del Hospital de Niños Dr. Ricardo Gutierrez	2020-03-30	Nabaes Jodar et al B
Argentina/PAIS_A009/2020	EPI_ISL_430801	Laboratorio de Virología del Hospital de Niños Dr. Ricardo Gutierrez	Área de Secuenciación del Laboratorio de Virología del Hospital de Niños Dr. Ricardo Gutierrez	2020-03-31	Nabaes Jodar et al A (https://dx.doi.org/10.1101/2020.05.23.20111443)
Argentina/PAIS_A011/2020	EPI_ISL_430802	Departamento de Biología y genética molecular, IACA Laboratorios.	Área de Secuenciación del Laboratorio de Virología del Hospital de Niños Dr. Ricardo Gutierrez	2020-03-31	Nabaes Jodar et al A (https://dx.doi.org/10.1101/2020.05.23.20111443)
Argentina/PAIS_A012/2020	EPI_ISL_430803	Laboratorio de Virología del Hospital de Niños Dr. Ricardo Gutierrez	Área de Secuenciación del Laboratorio de Virología del Hospital de Niños Dr. Ricardo Gutierrez	2020-04-01	Nabaes Jodar et al A (https://dx.doi.org/10.1101/2020.05.23.20111443)
Argentina/PAIS_A013/2020	EPI_ISL_430804	Laboratorio de Virología del Hospital de Niños Dr. Ricardo Gutierrez	Área de Secuenciación del Laboratorio de Virología del Hospital de Niños Dr. Ricardo Gutierrez	2020-04-01	Nabaes Jodar et al A (https://dx.doi.org/10.1101/2020.05.23.20111443)
Argentina/PAIS_A014/2020	EPI_ISL_430805	Departamento de Biología y genética molecular, IACA Laboratorios.	Área de Secuenciación del Laboratorio de Virología del Hospital de Niños Dr. Ricardo Gutierrez	2020-04-01	Nabaes Jodar et al A (https://dx.doi.org/10.1101/2020.05.23.20111443)
Argentina/PAIS_A015/2020	EPI_ISL_430806	Departamento de Biología y genética molecular, IACA Laboratorios.	Área de Secuenciación del Laboratorio de Virología del Hospital de Niños Dr. Ricardo Gutierrez	2020-04-01	Nabaes Jodar et al B
Argentina/PAIS_A016/2020	EPI_ISL_430807	Laboratorio de Virología del Hospital de Niños Dr. Ricardo Gutierrez	Área de Secuenciación del Laboratorio de Virología del Hospital de Niños Dr. Ricardo Gutierrez	2020-04-02	Nabaes Jodar et al A (https://dx.doi.org/10.1101/2020.05.23.20111443)
Argentina/PAIS_A017/2020	EPI_ISL_430808	Departamento de Biología y genética molecular, IACA Laboratorios.	Área de Secuenciación del Laboratorio de Virología del Hospital de Niños Dr. Ricardo Gutierrez	2020-04-03	Nabaes Jodar et al A (https://dx.doi.org/10.1101/2020.05.23.20111443)
Argentina/PAIS_A018/2020	EPI_ISL_430809	Laboratorio de Virología del Hospital de Niños Dr. Ricardo Gutierrez	Área de Secuenciación del Laboratorio de Virología del Hospital de Niños Dr. Ricardo Gutierrez	2020-04-04	Nabaes Jodar et al A (https://dx.doi.org/10.1101/2020.05.23.20111443)
Argentina/PAIS_A019/2020	EPI_ISL_430810	Laboratorio de Virología del Hospital de Niños Dr. Ricardo Gutierrez	Área de Secuenciación del Laboratorio de Virología del Hospital de Niños Dr. Ricardo Gutierrez	2020-04-07	Nabaes Jodar et al A (https://dx.doi.org/10.1101/2020.05.23.20111443)
Argentina/PAIS_A020/2020	EPI_ISL_430811	Laboratorio de Virología del Hospital de Niños Dr. Ricardo Gutierrez	Área de Secuenciación del Laboratorio de Virología del Hospital de Niños Dr. Ricardo Gutierrez	2020-04-11	Nabaes Jodar et al A (https://dx.doi.org/10.1101/2020.05.23.20111443)
Argentina/PAIS_A021/2020	EPI_ISL_430812	Laboratorio de Virología del Hospital de Niños Dr. Ricardo Gutierrez	Área de Secuenciación del Laboratorio de Virología del Hospital de Niños Dr. Ricardo Gutierrez	2020-04-11	Nabaes Jodar et al A (https://dx.doi.org/10.1101/2020.05.23.20111443)
Argentina/PAIS_A022/2020	EPI_ISL_430813	Laboratorio de Virología del Hospital de Niños Dr. Ricardo Gutierrez	Área de Secuenciación del Laboratorio de Virología del Hospital de Niños Dr. Ricardo Gutierrez	2020-04-11	Nabaes Jodar et al B
Argentina/PAIS_A023/2020	EPI_ISL_430814	Laboratorio de Virología del Hospital de Niños Dr. Ricardo Gutierrez	Área de Secuenciación del Laboratorio de Virología del Hospital de Niños Dr. Ricardo Gutierrez	2020-04-17	Nabaes Jodar et al A (https://dx.doi.org/10.1101/2020.05.23.20111443)
Argentina/PAIS_A024/2020	EPI_ISL_430815	Laboratorio de Virología del Hospital de Niños Dr. Ricardo Gutierrez	Área de Secuenciación del Laboratorio de Virología del Hospital de Niños Dr. Ricardo Gutierrez	2020-04-18	Nabaes Jodar et al A (https://dx.doi.org/10.1101/2020.05.23.20111443)
Argentina/PAIS_A025/2020	EPI_ISL_430816	Laboratorio de Virología del Hospital de Niños Dr. Ricardo Gutierrez	Área de Secuenciación del Laboratorio de Virología del Hospital de Niños Dr. Ricardo Gutierrez	2020-04-18	Nabaes Jodar et al A (https://dx.doi.org/10.1101/2020.05.23.20111443)
Argentina/PAIS_A026/2020	EPI_ISL_430817	Laboratorio de Virología del Hospital de Niños Dr. Ricardo Gutierrez	Área de Secuenciación del Laboratorio de Virología del Hospital de Niños Dr. Ricardo Gutierrez	2020-04-18	Nabaes Jodar et al A (https://dx.doi.org/10.1101/2020.05.23.20111443)
Argentina/PAIS_A027/2020	EPI_ISL_430818	Laboratorio de Virología del Hospital de Niños Dr. Ricardo Gutierrez	Área de Secuenciación del Laboratorio de Virología del Hospital de Niños Dr. Ricardo Gutierrez	2020-04-19	Nabaes Jodar et al A (https://dx.doi.org/10.1101/2020.05.23.20111443)
Australia/NSW01/2020	EPI_ISL_407893	Centre for Infectious Diseases and Microbiology Laboratory Services	NSW Health Pathology - Institute of Clinical Pathology and Medical Research; Westmead Hospital; University of Sydney	2020-01-24	Eden J-S et al (https://dx.doi.org/10.1101/2020.04.19.048751)
Australia/NSW02/2020	EPI_ISL_408976	Centre for Infectious Diseases and Microbiology Laboratory Services	NSW Health Pathology - Institute of Clinical Pathology and Medical Research; Westmead Hospital; University of Sydney	2020-01-22	Rockett R et al (https://dx.doi.org/10.1101/2020.04.19.048751)
Australia/NSW05/2020	EPI_ISL_412975	Centre for Infectious Diseases and Microbiology Laboratory Services	NSW Health Pathology - Institute of Clinical Pathology and Medical Research; Westmead Hospital; University of Sydney	2020-02-28	Eden J-S et al (https://dx.doi.org/10.1101/2020.04.19.048751)
Australia/NSW06/2020	EPI_ISL_413213	Centre for Infectious Diseases and Microbiology Laboratory Services	NSW Health Pathology - Institute of Clinical Pathology and Medical Research; Westmead Hospital; University of Sydney	2020-02-29	Eden J-S et al (https://dx.doi.org/10.1101/2020.04.19.048751)
Australia/NSW07/2020	EPI_ISL_413214	Centre for Infectious Diseases and Microbiology Laboratory Services	NSW Health Pathology - Institute of Clinical Pathology and Medical Research; Westmead Hospital; University of Sydney	2020-02-29	Eden J-S et al (https://dx.doi.org/10.1101/2020.04.19.048751)
Australia/NSW08/2020	EPI_ISL_413594	Centre for Infectious Diseases and Microbiology Laboratory Services	NSW Health Pathology - Institute of Clinical Pathology and Medical Research; Westmead Hospital; University of Sydney	2020-02-28	Rockett R et al (https://dx.doi.org/10.1101/2020.04.19.048751)
Australia/NSW09/2020	EPI_ISL_413595	Centre for Infectious Diseases and Microbiology Laboratory Services	NSW Health Pathology - Institute of Clinical Pathology and Medical Research; Westmead Hospital; University of Sydney	2020-02-28	Rockett R et al (https://dx.doi.org/10.1101/2020.04.19.048751)
Australia/NSW10/2020	EPI_ISL_413596	Centre for Infectious Diseases and Microbiology - Public Health	NSW Health Pathology - Institute of Clinical Pathology and Medical Research; Westmead Hospital; University of Sydney	2020-02-28	Rockett R et al (https://dx.doi.org/10.1101/2020.04.19.048751)
Australia/NSW170/2020	EPI_ISL_427676	Centre for Infectious Diseases and Microbiology Public Health	NSW Health Pathology - Institute of Clinical Pathology and Medical Research; Westmead Hospital; University of Sydney	2020-03-22	Draper J et al (https://dx.doi.org/10.1101/2020.04.19.048751)
Australia/NSW219/2020	EPI_ISL_427643	Centre for Infectious Diseases and Microbiology Public Health	NSW Health Pathology - Institute of Clinical Pathology and Medical Research; Westmead Hospital; University of Sydney	2020-02-28	Timms V et al (https://dx.doi.org/10.1101/2020.04.19.048751)
Australia/NSW276/2020	EPI_ISL_451540	ACT pathology	NSW Health Pathology - Institute of Clinical Pathology and Medical Research; Westmead Hospital; University of Sydney	2020-03-20	CIDM-PH et al. et al
Australia/NSW31/2020	EPI_ISL_417392	Centre for Infectious Diseases and Microbiology Public Health	NSW Health Pathology - Institute of Clinical Pathology and Medical Research; Westmead Hospital; University of Sydney	2020-03-10	Rahman H et al (https://dx.doi.org/10.1101/2020.04.19.048751)
Australia/NSW314/2020	EPI_ISL_451577	Pathology West - NSW Health Pathology	NSW Health Pathology - Institute of Clinical Pathology and Medical Research; Westmead Hospital; University of Sydney	2020-03-31	CIDM-PH et al. et al
Australia/NSW344/2020	EPI_ISL_451605	Childrens Hospital Westmead	NSW Health Pathology - Institute of Clinical Pathology and Medical Research; Westmead Hospital; University of Sydney	2020-04-02	CIDM-PH et al. et al
Australia/NSW362/2020	EPI_ISL_451623	Pathology West - NSW Health Pathology	NSW Health Pathology - Institute of Clinical Pathology and Medical Research; Westmead Hospital; University of Sydney	2020-04-15	CIDM-PH et al. et al
Australia/NSW379/2020	EPI_ISL_451639	Pathology West - NSW Health Pathology	NSW Health Pathology - Institute of Clinical Pathology and Medical Research; Westmead Hospital; University of Sydney	2020-04-13	CIDM-PH et al. et al
Australia/NSW512/2020	EPI_ISL_455052	Douglas Hanly Moir Pathology	NSW Health Pathology - Institute of Clinical Pathology and Medical Research; Westmead Hospital; University of Sydney	2020-03-31	CIDM-PH et al. et al
Australia/NSW517/2020	EPI_ISL_455057	Pathology West - NSW Health Pathology	NSW Health Pathology - Institute of Clinical Pathology and Medical Research; Westmead Hospital; University of Sydney	2020-05-01	CIDM-PH et al. et al
Australia/NSW519/2020	EPI_ISL_455059	Pathology West - NSW Health Pathology	NSW Health Pathology - Institute of Clinical Pathology and Medical Research; Westmead Hospital; University of Sydney	2020-04-17	CIDM-PH et al. et al
Australia/NSW529/2020	EPI_ISL_455068	Childrens Hospital Westmead	NSW Health Pathology - Institute of Clinical Pathology and Medical Research; Westmead Hospital; University of Sydney	2020-05-05	CIDM-PH et al. et al
Australia/NSW533/2020	EPI_ISL_455072	Sullivan Nicolaides Pathology	NSW Health Pathology - Institute of Clinical Pathology and Medical Research; Westmead Hospital; University of Sydney	2020-05-07	CIDM-PH et al. et al
Australia/NSW554/2020	EPI_ISL_455093	South Eastern Area Laboratory Services	NSW Health Pathology - Institute of Clinical Pathology and Medical Research; Westmead Hospital; University of Sydney	2020-05-20	CIDM-PH et al. et al
Australia/NSW72/2020	EPI_ISL_427744	Centre for Infectious Diseases and Microbiology Public Health	NSW Health Pathology - Institute of Clinical Pathology and Medical Research; Westmead Hospital; University of Sydney	2020-03-21	Bachmann N et al (https://dx.doi.org/10.1101/2020.04.19.048751)
Australia/NSW96/2020	EPI_ISL_427678	Centre for Infectious Diseases and Microbiology Public Health	NSW Health Pathology - Institute of Clinical Pathology and Medical Research; Westmead Hospital; University of Sydney	2020-03-23	Timms V et al (https://dx.doi.org/10.1101/2020.04.19.048751)
Australia/NT01/2020	EPI_ISL_419831	Royal Darwin Hospital	Victorian Infectious Diseases Reference Laboratory and Microbiological Diagnostic Unit Public Health Laboratory, Doherty Institute	2020-02-21	Meumann et al
Australia/NT02/2020	EPI_ISL_419832	Royal Darwin Hospital	Victorian Infectious Diseases Reference Laboratory and Microbiological Diagnostic Unit Public Health Laboratory, Doherty Institute	2020-02-21	Meumann et al
Australia/NT04/2020	EPI_ISL_419833	Royal Darwin Hospital	Victorian Infectious Diseases Reference Laboratory and Microbiological Diagnostic Unit Public Health Laboratory, Doherty Institute	2020-02-22	Meumann et al
Australia/NT05/2020	EPI_ISL_419835	Royal Darwin Hospital	Victorian Infectious Diseases Reference Laboratory and Microbiological Diagnostic Unit Public Health Laboratory, Doherty Institute	2020-02-24	Meumann et al
Australia/QLD01/2020	EPI_ISL_407894	Pathology Queensland	Public Health Virology Laboratory	2020-01-28	Ben Huang et al A
Australia/QLD02/2020	EPI_ISL_407896	Pathology Queensland	Public Health Virology Laboratory	2020-01-30	Ben Huang et al A
Australia/QLD03/2020	EPI_ISL_410717	Pathology Queensland	Public Health Virology Laboratory	2020-02-05	Ben Huang et al B (https://dx.doi.org/10.2807/1560-7917.ES.2020.25.13.2000305)
Australia/QLD04/2020	EPI_ISL_410718	Pathology Queensland	Public Health Virology Laboratory	2020-02-05	Ben Huang et al A
Australia/QLD09/2020	EPI_ISL_414414	Pathology Queensland	Public Health Virology Laboratory	2020-02-29	Bixing Huang et al
Australia/QLDID922/2020	EPI_ISL_418799	Mater Pathology	Public Health Virology Laboratory	2020-02-28	Bixing Huang et al
Australia/SAP067/2020	EPI_ISL_451105	SA Pathology	SA Pathology	2020-03-29	Lex Leong et al
Australia/SAP087/2020	EPI_ISL_451122	SA Pathology	SA Pathology	2020-03-31	Lex Leong et al
Australia/SAP089/2020	EPI_ISL_451124	SA Pathology	SA Pathology	2020-04-01	Lex Leong et al
Australia/SAP097/2020	EPI_ISL_468039	SA Pathology	SA Pathology	2020-02-22	Lex Leong et al
Australia/SAP212/2020	EPI_ISL_467989	SA Pathology	SA Pathology	2020-05-05	Lex Leong et al
Australia/SAP214/2020	EPI_ISL_468032	SA Pathology	SA Pathology	2020-01-30	Lex Leong et al
Australia/SAP305/2020	EPI_ISL_468037	SA Pathology	SA Pathology	2020-01-30	Lex Leong et al
Australia/VIC01/2020	EPI_ISL_406844	Monash Medical Centre	Collaboration between the University of Melbourne at The Peter Doherty Institute for Infection and Immunity, and the Victorian Infectious Disease Reference Laboratory	2020-01-25	Caly et al (https://dx.doi.org/10.5694/mja2.50569)
Australia/VIC07/2020	EPI_ISL_416415	Victorian Infectious Diseases Reference Laboratory (VIDRL)	Victorian Infectious Diseases Reference Laboratory and Microbiological Diagnostic Unit Public Health Laboratory, Doherty Institute	2020-02-08	Caly L. et al B (https://dx.doi.org/10.1101/2020.05.12.20099929)
Australia/VIC1018/2020	EPI_ISL_430490	Victorian Infectious Diseases Reference Laboratory (VIDRL)	Microbiological Diagnostic Unit Public Health Laboratory and Victorian Infectious Diseases Reference Laboratory, The Peter Doherty Institute for Infection and Immunity	2020-04-01	Caly L. et al B (https://dx.doi.org/10.1101/2020.05.12.20099929)
Australia/VIC1030/2020	EPI_ISL_430664	Microbiological Diagnostic Unit Public Health Laboratory	Microbiological Diagnostic Unit Public Health Laboratory	2020-04-02	Seemann T. et al B (https://dx.doi.org/10.1101/2020.05.12.20099929)
Australia/VIC1042/2020	EPI_ISL_430538	Victorian Infectious Diseases Reference Laboratory (VIDRL)	Microbiological Diagnostic Unit Public Health Laboratory and Victorian Infectious Diseases Reference Laboratory, The Peter Doherty Institute for Infection and Immunity	2020-04-03	Caly L. et al B (https://dx.doi.org/10.1101/2020.05.12.20099929)
Australia/VIC1081/2020	EPI_ISL_430580	Victorian Infectious Diseases Reference Laboratory (VIDRL)	Microbiological Diagnostic Unit Public Health Laboratory and Victorian Infectious Diseases Reference Laboratory, The Peter Doherty Institute for Infection and Immunity	2020-04-06	Caly L. et al B (https://dx.doi.org/10.1101/2020.05.12.20099929)
Australia/VIC1092/2020	EPI_ISL_430582	Victorian Infectious Diseases Reference Laboratory (VIDRL)	Microbiological Diagnostic Unit Public Health Laboratory and Victorian Infectious Diseases Reference Laboratory, The Peter Doherty Institute for Infection and Immunity	2020-04-07	Caly L. et al B (https://dx.doi.org/10.1101/2020.05.12.20099929)
Australia/VIC11/2020	EPI_ISL_416517	Victorian Infectious Diseases Reference Laboratory (VIDRL)	Victorian Infectious Diseases Reference Laboratory and Microbiological Diagnostic Unit Public Health Laboratory, Doherty Institute	2020-03-16	Caly L. et al B (https://dx.doi.org/10.1101/2020.05.12.20099929)
Australia/VIC1107/2020	EPI_ISL_430590	Victorian Infectious Diseases Reference Laboratory (VIDRL)	Microbiological Diagnostic Unit Public Health Laboratory and Victorian Infectious Diseases Reference Laboratory, The Peter Doherty Institute for Infection and Immunity	2020-04-07	Caly L. et al B (https://dx.doi.org/10.1101/2020.05.12.20099929)
Australia/VIC1157/2020	EPI_ISL_430598	Victorian Infectious Diseases Reference Laboratory (VIDRL)	Microbiological Diagnostic Unit Public Health Laboratory and Victorian Infectious Diseases Reference Laboratory, The Peter Doherty Institute for Infection and Immunity	2020-04-09	Caly L. et al B (https://dx.doi.org/10.1101/2020.05.12.20099929)
Australia/VIC1217/2020	EPI_ISL_430514	Victorian Infectious Diseases Reference Laboratory (VIDRL)	Microbiological Diagnostic Unit Public Health Laboratory and Victorian Infectious Diseases Reference Laboratory, The Peter Doherty Institute for Infection and Immunity	2020-04-01	Caly L. et al B (https://dx.doi.org/10.1101/2020.05.12.20099929)
Australia/VIC1260/2020	EPI_ISL_436123	Victorian Infectious Diseases Reference Laboratory (VIDRL)	Microbiological Diagnostic Unit Public Health Laboratory and Victorian Infectious Diseases Reference Laboratory, Doherty Institute	2020-04-11	Caly L. et al B (https://dx.doi.org/10.1101/2020.05.12.20099929)
Australia/VIC1265/2020	EPI_ISL_436124	Victorian Infectious Diseases Reference Laboratory (VIDRL)	Microbiological Diagnostic Unit Public Health Laboratory and Victorian Infectious Diseases Reference Laboratory, Doherty Institute	2020-04-12	Caly L. et al B (https://dx.doi.org/10.1101/2020.05.12.20099929)

Australia/VIC1274/2020	EPI_ISL_436132	Victorian Infectious Diseases Reference Laboratory (VIDRL)	Microbiological Diagnostic Unit Public Health Laboratory and Victorian Infectious Diseases Reference Laboratory, Doherty Institute	2020-04-14	Caly L. et al B (https://dx.doi.org/10.1101/2020.05.12.20099929)
Australia/VIC1292/2020	EPI_ISL_430711	Victorian Infectious Diseases Reference Laboratory (VIDRL)	Microbiological Diagnostic Unit Public Health Laboratory and Victorian Infectious Diseases Reference Laboratory, The Peter Doherty Institute for Infection and Immunity	2020-04-09	Caly L. et al A
Australia/VIC1300/2020	EPI_ISL_430677	Microbiological Diagnostic Unit Public Health Laboratory	Microbiological Diagnostic Unit Public Health Laboratory	2020-04-10	Seemann T. et al A
Australia/VIC1334/2020	EPI_ISL_430682	Microbiological Diagnostic Unit Public Health Laboratory	Microbiological Diagnostic Unit Public Health Laboratory	2020-04-13	Seemann T. et al A
Australia/VIC1378/2020	EPI_ISL_456417	Victorian Infectious Diseases Reference Laboratory (VIDRL)	Microbiological Diagnostic Unit Public Health Laboratory and Victorian Infectious Diseases Reference Laboratory, Doherty Institute	2020-04-14	Caly L. et al A
Australia/VIC138/2020	EPI_ISL_419834	Victorian Infectious Diseases Reference Laboratory (VIDRL)	Victorian Infectious Diseases Reference Laboratory and Microbiological Diagnostic Unit Public Health Laboratory, Doherty Institute	2020-02-23	Caly L. et al B (https://dx.doi.org/10.1101/2020.05.12.20099929)
Australia/VIC14/2020	EPI_ISL_419734	Victorian Infectious Diseases Reference Laboratory (VIDRL)	Victorian Infectious Diseases Reference Laboratory and Microbiological Diagnostic Unit Public Health Laboratory, Doherty Institute	2020-02-02	Caly L. et al B (https://dx.doi.org/10.1101/2020.05.12.20099929)
Australia/VIC1428/2020	EPI_ISL_456435	Victorian Infectious Diseases Reference Laboratory (VIDRL)	Microbiological Diagnostic Unit Public Health Laboratory and Victorian Infectious Diseases Reference Laboratory, Doherty Institute	2020-04-17	Caly L. et al A
Australia/VIC143/2020	EPI_ISL_419840	Victorian Infectious Diseases Reference Laboratory (VIDRL)	Victorian Infectious Diseases Reference Laboratory and Microbiological Diagnostic Unit Public Health Laboratory, Doherty Institute	2020-03-16	Caly L. et al B (https://dx.doi.org/10.1101/2020.05.12.20099929)
Australia/VIC15/2020	EPI_ISL_419735	Victorian Infectious Diseases Reference Laboratory (VIDRL)	Victorian Infectious Diseases Reference Laboratory and Microbiological Diagnostic Unit Public Health Laboratory, Doherty Institute	2020-02-24	Caly L. et al A
Australia/VIC1533/2020	EPI_ISL_456478	Microbiological Diagnostic Unit Public Health Laboratory	Microbiological Diagnostic Unit Public Health Laboratory, The Peter Doherty Institute for Infection and Immunity	2020-05-06	Seemann T. et al A
Australia/VIC1561/2020	EPI_ISL_456480	Victorian Infectious Diseases Reference Laboratory (VIDRL)	Microbiological Diagnostic Unit Public Health Laboratory and Victorian Infectious Diseases Reference Laboratory, Doherty Institute	2020-05-04	Caly L. et al A
Australia/VIC1566/2020	EPI_ISL_456484	Victorian Infectious Diseases Reference Laboratory (VIDRL)	Microbiological Diagnostic Unit Public Health Laboratory and Victorian Infectious Diseases Reference Laboratory, Doherty Institute	2020-05-06	Caly L. et al A
Australia/VIC1567/2020	EPI_ISL_456487	Victorian Infectious Diseases Reference Laboratory (VIDRL)	Microbiological Diagnostic Unit Public Health Laboratory and Victorian Infectious Diseases Reference Laboratory, Doherty Institute	2020-05-06	Caly L. et al A
Australia/VIC1571/2020	EPI_ISL_456486	Victorian Infectious Diseases Reference Laboratory (VIDRL)	Microbiological Diagnostic Unit Public Health Laboratory and Victorian Infectious Diseases Reference Laboratory, Doherty Institute	2020-05-07	Caly L. et al A
Australia/VIC1606/2020	EPI_ISL_456517	Victorian Infectious Diseases Reference Laboratory (VIDRL)	Microbiological Diagnostic Unit Public Health Laboratory and Victorian Infectious Diseases Reference Laboratory, Doherty Institute	2020-05-12	Caly L. et al A
Australia/VIC1609/2020	EPI_ISL_456520	Victorian Infectious Diseases Reference Laboratory (VIDRL)	Microbiological Diagnostic Unit Public Health Laboratory and Victorian Infectious Diseases Reference Laboratory, Doherty Institute	2020-05-12	Caly L. et al A
Australia/VIC1617/2020	EPI_ISL_456525	Victorian Infectious Diseases Reference Laboratory (VIDRL)	Microbiological Diagnostic Unit Public Health Laboratory and Victorian Infectious Diseases Reference Laboratory, Doherty Institute	2020-05-06	Caly L. et al A
Australia/VIC1625/2020	EPI_ISL_456532	Victorian Infectious Diseases Reference Laboratory (VIDRL)	Microbiological Diagnostic Unit Public Health Laboratory and Victorian Infectious Diseases Reference Laboratory, Doherty Institute	2020-05-13	Caly L. et al A
Australia/VIC1632/2020	EPI_ISL_456537	Victorian Infectious Diseases Reference Laboratory (VIDRL)	Microbiological Diagnostic Unit Public Health Laboratory and Victorian Infectious Diseases Reference Laboratory, Doherty Institute	2020-05-05	Caly L. et al A
Australia/VIC1633/2020	EPI_ISL_456538	Victorian Infectious Diseases Reference Laboratory (VIDRL)	Microbiological Diagnostic Unit Public Health Laboratory and Victorian Infectious Diseases Reference Laboratory, Doherty Institute	2020-05-04	Caly L. et al A
Australia/VIC1638/2020	EPI_ISL_456541	Victorian Infectious Diseases Reference Laboratory (VIDRL)	Microbiological Diagnostic Unit Public Health Laboratory and Victorian Infectious Diseases Reference Laboratory, Doherty Institute	2020-05-02	Caly L. et al A
Australia/VIC1641/2020	EPI_ISL_456544	Victorian Infectious Diseases Reference Laboratory (VIDRL)	Microbiological Diagnostic Unit Public Health Laboratory and Victorian Infectious Diseases Reference Laboratory, Doherty Institute	2020-05-02	Caly L. et al A
Australia/VIC1643/2020	EPI_ISL_456546	Victorian Infectious Diseases Reference Laboratory (VIDRL)	Microbiological Diagnostic Unit Public Health Laboratory and Victorian Infectious Diseases Reference Laboratory, Doherty Institute	2020-05-02	Caly L. et al A
Australia/VIC1652/2020	EPI_ISL_456554	Victorian Infectious Diseases Reference Laboratory (VIDRL)	Microbiological Diagnostic Unit Public Health Laboratory and Victorian Infectious Diseases Reference Laboratory, Doherty Institute	2020-05-14	Caly L. et al A
Australia/VIC1665/2020	EPI_ISL_456566	Victorian Infectious Diseases Reference Laboratory (VIDRL)	Microbiological Diagnostic Unit Public Health Laboratory and Victorian Infectious Diseases Reference Laboratory, Doherty Institute	2020-05-09	Caly L. et al A
Australia/VIC1673/2020	EPI_ISL_456572	Victorian Infectious Diseases Reference Laboratory (VIDRL)	Microbiological Diagnostic Unit Public Health Laboratory and Victorian Infectious Diseases Reference Laboratory, Doherty Institute	2020-05-12	Caly L. et al A
Australia/VIC1697/2020	EPI_ISL_456584	Victorian Infectious Diseases Reference Laboratory (VIDRL)	Microbiological Diagnostic Unit Public Health Laboratory and Victorian Infectious Diseases Reference Laboratory, Doherty Institute	2020-05-17	Caly L. et al A
Australia/VIC1711/2020	EPI_ISL_456616	Victorian Infectious Diseases Reference Laboratory (VIDRL)	Microbiological Diagnostic Unit Public Health Laboratory and Victorian Infectious Diseases Reference Laboratory, Doherty Institute	2020-05-19	Caly L. et al A
Australia/VIC1726/2020	EPI_ISL_456626	Victorian Infectious Diseases Reference Laboratory (VIDRL)	Microbiological Diagnostic Unit Public Health Laboratory and Victorian Infectious Diseases Reference Laboratory, Doherty Institute	2020-05-23	Caly L. et al A
Australia/VIC1730/2020	EPI_ISL_456629	Victorian Infectious Diseases Reference Laboratory (VIDRL)	Microbiological Diagnostic Unit Public Health Laboratory and Victorian Infectious Diseases Reference Laboratory, Doherty Institute	2020-05-01	Caly L. et al A
Australia/VIC1736/2020	EPI_ISL_456633	Victorian Infectious Diseases Reference Laboratory (VIDRL)	Microbiological Diagnostic Unit Public Health Laboratory and Victorian Infectious Diseases Reference Laboratory, Doherty Institute	2020-05-02	Caly L. et al A
Australia/VIC1792/2020	EPI_ISL_456652	Victorian Infectious Diseases Reference Laboratory (VIDRL)	Microbiological Diagnostic Unit Public Health Laboratory and Victorian Infectious Diseases Reference Laboratory, Doherty Institute	2020-05-26	Caly L. et al A
Australia/VIC1793/2020	EPI_ISL_456653	Victorian Infectious Diseases Reference Laboratory (VIDRL)	Microbiological Diagnostic Unit Public Health Laboratory and Victorian Infectious Diseases Reference Laboratory, Doherty Institute	2020-05-26	Caly L. et al A
Australia/VIC1794/2020	EPI_ISL_456654	Victorian Infectious Diseases Reference Laboratory (VIDRL)	Microbiological Diagnostic Unit Public Health Laboratory and Victorian Infectious Diseases Reference Laboratory, Doherty Institute	2020-05-26	Caly L. et al A
Australia/VIC211/2020	EPI_ISL_419906	Victorian Infectious Diseases Reference Laboratory (VIDRL)	Victorian Infectious Diseases Reference Laboratory and Microbiological Diagnostic Unit Public Health Laboratory, Doherty Institute	2020-03-20	Caly L. et al B (https://dx.doi.org/10.1101/2020.05.12.20099929)
Australia/VIC237/2020	EPI_ISL_419932	Victorian Infectious Diseases Reference Laboratory (VIDRL)	Victorian Infectious Diseases Reference Laboratory and Microbiological Diagnostic Unit Public Health Laboratory, Doherty Institute	2020-03-21	Caly L. et al B (https://dx.doi.org/10.1101/2020.05.12.20099929)
Australia/VIC243/2020	EPI_ISL_419938	Victorian Infectious Diseases Reference Laboratory (VIDRL)	Victorian Infectious Diseases Reference Laboratory and Microbiological Diagnostic Unit Public Health Laboratory, Doherty Institute	2020-03-21	Caly L. et al B (https://dx.doi.org/10.1101/2020.05.12.20099929)
Australia/VIC262/2020	EPI_ISL_419955	Victorian Infectious Diseases Reference Laboratory (VIDRL)	Victorian Infectious Diseases Reference Laboratory and Microbiological Diagnostic Unit Public Health Laboratory, Doherty Institute	2020-03-21	Caly L. et al B (https://dx.doi.org/10.1101/2020.05.12.20099929)
Australia/VIC263/2020	EPI_ISL_419956	Victorian Infectious Diseases Reference Laboratory (VIDRL)	Victorian Infectious Diseases Reference Laboratory and Microbiological Diagnostic Unit Public Health Laboratory, Doherty Institute	2020-03-21	Caly L. et al B (https://dx.doi.org/10.1101/2020.05.12.20099929)
Australia/VIC284/2020	EPI_ISL_419975	Victorian Infectious Diseases Reference Laboratory (VIDRL)	Victorian Infectious Diseases Reference Laboratory and Microbiological Diagnostic Unit Public Health Laboratory, Doherty Institute	2020-03-22	Caly L. et al B (https://dx.doi.org/10.1101/2020.05.12.20099929)
Australia/VIC287/2020	EPI_ISL_419978	Victorian Infectious Diseases Reference Laboratory (VIDRL)	Victorian Infectious Diseases Reference Laboratory and Microbiological Diagnostic Unit Public Health Laboratory, Doherty Institute	2020-03-22	Caly L. et al B (https://dx.doi.org/10.1101/2020.05.12.20099929)
Australia/VIC52/2020	EPI_ISL_419768	Victorian Infectious Diseases Reference Laboratory (VIDRL)	Victorian Infectious Diseases Reference Laboratory and Microbiological Diagnostic Unit Public Health Laboratory, Doherty Institute	2020-03-12	Caly L. et al B (https://dx.doi.org/10.1101/2020.05.12.20099929)
Australia/VIC694/2020	EPI_ISL_426986	Victorian Infectious Diseases Reference Laboratory (VIDRL)	Microbiological Diagnostic Unit Public Health Laboratory and Victorian Infectious Diseases Reference Laboratory, Doherty Institute	2020-03-28	Caly L. et al B (https://dx.doi.org/10.1101/2020.05.12.20099929)
Australia/VIC712/2020	EPI_ISL_426999	Victorian Infectious Diseases Reference Laboratory (VIDRL)	Microbiological Diagnostic Unit Public Health Laboratory and Victorian Infectious Diseases Reference Laboratory, Doherty Institute	2020-03-29	Caly L. et al B (https://dx.doi.org/10.1101/2020.05.12.20099929)
Australia/VIC777/2020	EPI_ISL_427052	Victorian Infectious Diseases Reference Laboratory (VIDRL)	Microbiological Diagnostic Unit Public Health Laboratory and Victorian Infectious Diseases Reference Laboratory, Doherty Institute	2020-03-30	Caly L. et al B (https://dx.doi.org/10.1101/2020.05.12.20099929)
Australia/VIC792/2020	EPI_ISL_427067	Microbiological Diagnostic Unit Public Health Laboratory	Microbiological Diagnostic Unit Public Health Laboratory	2020-03-31	Seemann T. et al B (https://dx.doi.org/10.1101/2020.05.12.20099929)
Australia/VIC798/2020	EPI_ISL_427072	Microbiological Diagnostic Unit Public Health Laboratory	Microbiological Diagnostic Unit Public Health Laboratory	2020-04-01	Seemann T. et al B (https://dx.doi.org/10.1101/2020.05.12.20099929)
Australia/VIC804/2020	EPI_ISL_427078	Microbiological Diagnostic Unit Public Health Laboratory	Microbiological Diagnostic Unit Public Health Laboratory	2020-04-01	Seemann T. et al B (https://dx.doi.org/10.1101/2020.05.12.20099929)
Australia/VIC846/2020	EPI_ISL_427106	Victorian Infectious Diseases Reference Laboratory (VIDRL)	Microbiological Diagnostic Unit Public Health Laboratory and Victorian Infectious Diseases Reference Laboratory, Doherty Institute	2020-04-04	Caly L. et al B (https://dx.doi.org/10.1101/2020.05.12.20099929)
Australia/VIC887/2020	EPI_ISL_427140	Victorian Infectious Diseases Reference Laboratory (VIDRL)	Microbiological Diagnostic Unit Public Health Laboratory and Victorian Infectious Diseases Reference Laboratory, Doherty Institute	2020-04-06	Caly L. et al B (https://dx.doi.org/10.1101/2020.05.12.20099929)
Australia/VIC947/2020	EPI_ISL_430644	Microbiological Diagnostic Unit Public Health Laboratory	Microbiological Diagnostic Unit Public Health Laboratory	2020-03-26	Seemann T. et al B (https://dx.doi.org/10.1101/2020.05.12.20099929)
Australia/VIC965/2020	EPI_ISL_430650	Microbiological Diagnostic Unit Public Health Laboratory	Microbiological Diagnostic Unit Public Health Laboratory	2020-03-27	Seemann T. et al B (https://dx.doi.org/10.1101/2020.05.12.20099929)
Australia/VIC991/2020	EPI_ISL_430474	Victorian Infectious Diseases Reference Laboratory (VIDRL)	Microbiological Diagnostic Unit Public Health Laboratory and Victorian Infectious Diseases Reference Laboratory, The Peter Doherty Institute for Infection and Immunity	2020-03-31	Caly L. et al B (https://dx.doi.org/10.1101/2020.05.12.20099929)
Australia/VPRL005/2020	EPI_ISL_455597	SA Pathology	VPRL	2020-04-02	Beard et al
Australia/VPRL014/2020	EPI_ISL_455603	SA Pathology	VPRL	2020-01-30	Beard et al
Australia/WA11/2020	EPI_ISL_420456	PathWest Laboratory Medicine WA	PathWest Laboratory Medicine WA	2020-02-22	Chisha Sikazwe et al
Australia/WA17/2020	EPI_ISL_470835	PathWest Laboratory Medicine WA	PathWest Laboratory Medicine WA	2020-04-08	Chisha Sikazwe et al
Australia/WA21/2020	EPI_ISL_470839	PathWest Laboratory Medicine WA	PathWest Laboratory Medicine WA	2020-04-28	Chisha Sikazwe et al
Australia/WA22/2020	EPI_ISL_470840	PathWest Laboratory Medicine WA	PathWest Laboratory Medicine WA	2020-03-24	Chisha Sikazwe et al
Australia/WA34/2020	EPI_ISL_470851	PathWest Laboratory Medicine WA	PathWest Laboratory Medicine WA	2020-02-27	Chisha Sikazwe et al
Australia/WA37/2020	EPI_ISL_470854	PathWest Laboratory Medicine WA	PathWest Laboratory Medicine WA	2020-04-20	Chisha Sikazwe et al
Austria/CeMM0002/2020	EPI_ISL_419655	Center for Virology, Medical University of Vienna	Bergthaler laboratory, CeMM Research Center for Molecular Medicine of the Austrian Academy of Sciences	2020-02-26	Alexandra Popa et al
Austria/CeMM0003/2020	EPI_ISL_419656	Center for Virology, Medical University of Vienna	Bergthaler laboratory, CeMM Research Center for Molecular Medicine of the Austrian Academy of Sciences	2020-02-26	Alexandra Popa et al
Austria/CeMM0008/2020	EPI_ISL_419661	Center for Virology, Medical University of Vienna	Bergthaler laboratory, CeMM Research Center for Molecular Medicine of the Austrian Academy of Sciences	2020-03-10	Alexandra Popa et al
Austria/CeMM0013/2020	EPI_ISL_419666	Center for Virology, Medical University of Vienna	Bergthaler laboratory, CeMM Research Center for Molecular Medicine of the Austrian Academy of Sciences	2020-03-11	Alexandra Popa et al
Austria/CeMM0016/2020	EPI_ISL_419669	Center for Virology, Medical University of Vienna	Bergthaler laboratory, CeMM Research Center for Molecular Medicine of the Austrian Academy of Sciences	2020-03-16	Alexandra Popa et al
Austria/CeMM0023/2020	EPI_ISL_437913	Institut für Virologie am Department für Hygiene, Mikrobiologie und Public Health	Bergthaler laboratory, CeMM Research Center for Molecular Medicine of the Austrian Academy of Sciences	2020-03-09	Alexandra Popa et al
Austria/CeMM0024/2020	EPI_ISL_437914	Institut für Virologie am Department für Hygiene, Mikrobiologie und Public Health	Bergthaler laboratory, CeMM Research Center for Molecular Medicine of the Austrian Academy of Sciences	2020-03-09	Alexandra Popa et al
Austria/CeMM0031/2020	EPI_ISL_437920	Institut für Virologie am Department für Hygiene, Mikrobiologie und Public Health	Bergthaler laboratory, CeMM Research Center for Molecular Medicine of the Austrian Academy of Sciences	2020-03-09	Alexandra Popa et al
Austria/CeMM0042/2020	EPI_ISL_437930	Institut für Virologie am Department für Hygiene, Mikrobiologie und Public Health	Bergthaler laboratory, CeMM Research Center for Molecular Medicine of the Austrian Academy of Sciences	2020-03-09	Alexandra Popa et al
Austria/CeMM0045/2020	EPI_ISL_437932	Institut für Virologie am Department für Hygiene, Mikrobiologie und Public Health	Bergthaler laboratory, CeMM Research Center for Molecular Medicine of the Austrian Academy of Sciences	2020-02-24	Alexandra Popa et al
Austria/CeMM0055/2020	EPI_ISL_437937	Universitaetsklinik für Innere Medizin II Innsbruck	Bergthaler laboratory, CeMM Research Center for Molecular Medicine of the Austrian Academy of Sciences	2020-03-27	Alexandra Popa et al
Austria/CeMM0058/2020	EPI_ISL_437938	Universitaetsklinik für Innere Medizin II Innsbruck	Bergthaler laboratory, CeMM Research Center for Molecular Medicine of the Austrian Academy of Sciences	2020-04-03	Alexandra Popa et al
Austria/CeMM0067/2020	EPI_ISL_437939	Universitaetsklinik für Innere Medizin II Innsbruck	Bergthaler laboratory, CeMM Research Center for Molecular Medicine of the Austrian Academy of Sciences	2020-04-06	Alexandra Popa et al
Austria/CeMM0080/2020	EPI_ISL_437947	Universitaetsklinik für Innere Medizin II Innsbruck	Bergthaler laboratory, CeMM Research Center for Molecular Medicine of the Austrian Academy of Sciences	2020-03-24	Alexandra Popa et al
Austria/CeMM0106/2020	EPI_ISL_437966	Universitaetsklinik für Innere Medizin II Innsbruck	Bergthaler laboratory, CeMM Research Center for Molecular Medicine of the Austrian Academy of Sciences	2020-03-18	Alexandra Popa et al
Austria/CeMM0109/2020	EPI_ISL_437968	Universitaetsklinik für Innere Medizin II Innsbruck	Bergthaler laboratory, CeMM Research Center for Molecular Medicine of the Austrian Academy of Sciences	2020-03-12	Alexandra Popa et al
Austria/CeMM0111/2020	EPI_ISL_437969	Universitaetsklinik für Innere Medizin II Innsbruck	Bergthaler laboratory, CeMM Research Center for Molecular Medicine of the Austrian Academy of Sciences	2020-03-12	Alexandra Popa et al
Austria/CeMM0116/2020	EPI_ISL_437974	Institut für Virologie am Department für Hygiene, Mikrobiologie und Public Health	Bergthaler laboratory, CeMM Research Center for Molecular Medicine of the Austrian Academy of Sciences	2020-03-10	Alexandra Popa et al

Austria/CeMM0143/2020	EPI_ISL_437993	Center for Virology, Medical University of Vienna	Bergthaler laboratory, CeMM Research Center for Molecular Medicine of the Austrian Academy of Sciences	2020-02-27	Alexandra Popa et al
Austria/CeMM0146/2020	EPI_ISL_437994	Center for Virology, Medical University of Vienna	Bergthaler laboratory, CeMM Research Center for Molecular Medicine of the Austrian Academy of Sciences	2020-02-28	Alexandra Popa et al
Austria/CeMM0147/2020	EPI_ISL_437995	Center for Virology, Medical University of Vienna	Bergthaler laboratory, CeMM Research Center for Molecular Medicine of the Austrian Academy of Sciences	2020-02-28	Alexandra Popa et al
Austria/CeMM0150/2020	EPI_ISL_437998	Center for Virology, Medical University of Vienna	Bergthaler laboratory, CeMM Research Center for Molecular Medicine of the Austrian Academy of Sciences	2020-03-03	Alexandra Popa et al
Austria/CeMM0170/2020	EPI_ISL_438015	Center for Virology, Medical University of Vienna	Bergthaler laboratory, CeMM Research Center for Molecular Medicine of the Austrian Academy of Sciences	2020-03-07	Alexandra Popa et al
Austria/CeMM0184/2020	EPI_ISL_438026	Center for Virology, Medical University of Vienna	Bergthaler laboratory, CeMM Research Center for Molecular Medicine of the Austrian Academy of Sciences	2020-03-11	Alexandra Popa et al
Austria/CeMM0196/2020	EPI_ISL_438037	Center for Virology, Medical University of Vienna	Bergthaler laboratory, CeMM Research Center for Molecular Medicine of the Austrian Academy of Sciences	2020-03-14	Alexandra Popa et al
Austria/CeMM0199/2020	EPI_ISL_438040	Center for Virology, Medical University of Vienna	Bergthaler laboratory, CeMM Research Center for Molecular Medicine of the Austrian Academy of Sciences	2020-03-15	Alexandra Popa et al
Austria/CeMM0200/2020	EPI_ISL_438041	Center for Virology, Medical University of Vienna	Bergthaler laboratory, CeMM Research Center for Molecular Medicine of the Austrian Academy of Sciences	2020-03-15	Alexandra Popa et al
Austria/CeMM0205/2020	EPI_ISL_438044	Center for Virology, Medical University of Vienna	Bergthaler laboratory, CeMM Research Center for Molecular Medicine of the Austrian Academy of Sciences	2020-03-15	Alexandra Popa et al
Austria/CeMM0212/2020	EPI_ISL_438048	Center for Virology, Medical University of Vienna	Bergthaler laboratory, CeMM Research Center for Molecular Medicine of the Austrian Academy of Sciences	2020-03-16	Alexandra Popa et al
Austria/CeMM0221/2020	EPI_ISL_438055	Center for Virology, Medical University of Vienna	Bergthaler laboratory, CeMM Research Center for Molecular Medicine of the Austrian Academy of Sciences	2020-03-16	Alexandra Popa et al
Austria/CeMM0241/2020	EPI_ISL_438071	Center for Virology, Medical University of Vienna	Bergthaler laboratory, CeMM Research Center for Molecular Medicine of the Austrian Academy of Sciences	2020-03-20	Alexandra Popa et al
Austria/CeMM0256/2020	EPI_ISL_438085	Center for Virology, Medical University of Vienna	Bergthaler laboratory, CeMM Research Center for Molecular Medicine of the Austrian Academy of Sciences	2020-03-22	Alexandra Popa et al
Austria/CeMM0259/2020	EPI_ISL_438088	Center for Virology, Medical University of Vienna	Bergthaler laboratory, CeMM Research Center for Molecular Medicine of the Austrian Academy of Sciences	2020-03-23	Alexandra Popa et al
Austria/CeMM0274/2020	EPI_ISL_438101	Center for Virology, Medical University of Vienna	Bergthaler laboratory, CeMM Research Center for Molecular Medicine of the Austrian Academy of Sciences	2020-03-24	Alexandra Popa et al
Austria/CeMM0287/2020	EPI_ISL_438112	Center for Virology, Medical University of Vienna	Bergthaler laboratory, CeMM Research Center for Molecular Medicine of the Austrian Academy of Sciences	2020-03-26	Alexandra Popa et al
Austria/CeMM0290/2020	EPI_ISL_438114	Center for Virology, Medical University of Vienna	Bergthaler laboratory, CeMM Research Center for Molecular Medicine of the Austrian Academy of Sciences	2020-03-27	Alexandra Popa et al
Austria/CeMM0357/2020	EPI_ISL_438120	Center for Virology, Medical University of Vienna	Bergthaler laboratory, CeMM Research Center for Molecular Medicine of the Austrian Academy of Sciences	2020-03-19	Alexandra Popa et al
Austria/CeMM0359/2020	EPI_ISL_438122	Center for Virology, Medical University of Vienna	Bergthaler laboratory, CeMM Research Center for Molecular Medicine of the Austrian Academy of Sciences	2020-04-02	Alexandra Popa et al
Austria/CeMM0360/2020	EPI_ISL_438123	Center for Virology, Medical University of Vienna	Bergthaler laboratory, CeMM Research Center for Molecular Medicine of the Austrian Academy of Sciences	2020-04-05	Alexandra Popa et al
Austria/CeMM0363/2020	EPI_ISL_438126	Center for Virology, Medical University of Vienna	Bergthaler laboratory, CeMM Research Center for Molecular Medicine of the Austrian Academy of Sciences	2020-04-02	Alexandra Popa et al
Austria/CeMM0364/2020	EPI_ISL_438127	Center for Virology, Medical University of Vienna	Bergthaler laboratory, CeMM Research Center for Molecular Medicine of the Austrian Academy of Sciences	2020-04-05	Alexandra Popa et al
Austria/CeMM0365/2020	EPI_ISL_438128	Center for Virology, Medical University of Vienna	Bergthaler laboratory, CeMM Research Center for Molecular Medicine of the Austrian Academy of Sciences	2020-04-07	Alexandra Popa et al
Austria/Graz-MUG1/2020	EPI_ISL_437197	Diagnostic- and Research Institute of Pathology, Medical University of Graz	Diagnostic- and Research Institute of Pathology, Medical University of Graz	2020-03-24	Karl Kashofer et al
Austria/Graz-MUG11/2020	EPI_ISL_437300	Diagnostic- and Research Institute of Pathology, Medical University of Graz	Diagnostic- and Research Institute of Pathology, Medical University of Graz	2020-04-14	Karl Kashofer et al
Austria/Graz-MUG15/2020	EPI_ISL_437301	Diagnostic- and Research Institute of Pathology, Medical University of Graz	Diagnostic- and Research Institute of Pathology, Medical University of Graz	2020-04-17	Karl Kashofer et al
Austria/Graz-MUG16/2020	EPI_ISL_437302	Diagnostic- and Research Institute of Pathology, Medical University of Graz	Diagnostic- and Research Institute of Pathology, Medical University of Graz	2020-04-17	Karl Kashofer et al
Austria/Graz-MUG5/2020	EPI_ISL_437201	Diagnostic- and Research Institute of Pathology, Medical University of Graz	Diagnostic- and Research Institute of Pathology, Medical University of Graz	2020-04-02	Karl Kashofer et al
Austria/Graz-MUG6/2020	EPI_ISL_437202	Diagnostic- and Research Institute of Pathology, Medical University of Graz	Diagnostic- and Research Institute of Pathology, Medical University of Graz	2020-04-03	Karl Kashofer et al
Austria/Graz-MUG7/2020	EPI_ISL_437203	Diagnostic- and Research Institute of Pathology, Medical University of Graz	Diagnostic- and Research Institute of Pathology, Medical University of Graz	2020-04-06	Karl Kashofer et al
Austria/Graz-MUG8/2020	EPI_ISL_437298	Diagnostic- and Research Institute of Pathology, Medical University of Graz	Diagnostic- and Research Institute of Pathology, Medical University of Graz	2020-04-08	Karl Kashofer et al
Austria/Graz-MUG9/2020	EPI_ISL_437299	Diagnostic- and Research Institute of Pathology, Medical University of Graz	Diagnostic- and Research Institute of Pathology, Medical University of Graz	2020-04-09	Karl Kashofer et al
Bangladesh/Akbiomed_01/2020	EPI_ISL_445244	Akbiomed lab	Tejgaon College bmb lab	2020-04-25	Md.Abdul kaium et al (https://dx.doi.org/10.1101/2020.06.07.138800)
Bangladesh/BARJ_CVASU_CTG_502/2020	EPI_ISL_450340	Corona (COVID-19) Testing Laboratory and Bangladesh Institute of Tropical & Infectious Diseases	Basic and Applied Research on Jute Project	2020-05-13	Rasel Ahmed et al (https://dx.doi.org/10.1101/2020.06.07.138800)
Bangladesh/BARJ_CVASU_CTG_506/2020	EPI_ISL_450342	Corona (COVID-19) Testing Laboratory and Bangladesh Institute of Tropical & Infectious Diseases	Basic and Applied Research on Jute Project	2020-05-08	Rasel Ahmed et al (https://dx.doi.org/10.1101/2020.06.07.138800)
Bangladesh/BARJ_CVASU_CTG_517/2020	EPI_ISL_450344	Corona (COVID-19) Testing Laboratory and Bangladesh Institute of Tropical & Infectious Diseases	Basic and Applied Research on Jute Project	2020-05-03	Rasel Ahmed et al (https://dx.doi.org/10.1101/2020.06.07.138800)
Bangladesh/BARJ_CVASU_CTG_518/2020	EPI_ISL_450345	Corona (COVID-19) Testing Laboratory and Bangladesh Institute of Tropical & Infectious Diseases	Basic and Applied Research on Jute Project	2020-05-10	Md. Sabbir Hossain et al (https://dx.doi.org/10.1101/2020.06.07.138800)
Bangladesh/BCSIR_NILMRC_003/2020	EPI_ISL_462092	National Institute of Laboratory Medicine and Referral Center	Genomic Research Lab, BCSIR	2020-05-23	Shahina Akter et al
Bangladesh/BCSIR_NILMRC_004/2020	EPI_ISL_462090	National Institute of Laboratory Medicine and Referral Center	Genomic Research Lab, BCSIR	2020-05-23	Barna Goswami et al
Bangladesh/BCSIR_NILMRC_006/2020	EPI_ISL_455458	National Institute of Laboratory Medicine and Referral Center	Genomic Research Lab, BCSIR	2020-05-21	Abu Sayeed Mohammad Mahmud et al
Bangladesh/BCSIR_NILMRC_008/2020	EPI_ISL_455459	National Institute of Laboratory Medicine and Referral Center	Genomic Research Lab, BCSIR	2020-05-21	Abu Sayeed Mohammad Mahmud et al
Bangladesh/BCSIR-NILMRC_015/2020	EPI_ISL_462096	National Institute of Laboratory Medicine and Referral Center	Genomic Research Lab, BCSIR	2020-05-23	Abu Sayeed Mohammad Mahmud et al
Bangladesh/BCSIR-NILMRC_050/2020	EPI_ISL_462098	National Institute of Laboratory Medicine and Referral Center	Genomic Research Lab, BCSIR	2020-05-21	Abu Sayeed Mohammad Mahmud et al
Bangladesh/BCSIR-NILMRC_052/2020	EPI_ISL_466627	National Institute of Laboratory Medicine and Referral Center	Genomic Research Lab, BCSIR	2020-06-01	Abu Sayeed Mohammad Mahmud et al
Bangladesh/BCSIR-NILMRC_054/2020	EPI_ISL_466628	National Institute of Laboratory Medicine and Referral Center	Genomic Research Lab, BCSIR	2020-06-01	Abu Sayeed Mohammad Mahmud et al
Bangladesh/BCSIR-NILMRC_056/2020	EPI_ISL_466629	National Institute of Laboratory Medicine and Referral Center	Genomic Research Lab, BCSIR	2020-05-10	Abu Sayeed Mohammad Mahmud et al
Bangladesh/BCSIR-NILMRC_057/2020	EPI_ISL_466630	National Institute of Laboratory Medicine and Referral Center	Genomic Research Lab, BCSIR	2020-05-10	Abu Sayeed Mohammad Mahmud et al
Bangladesh/BCSIR-NILMRC_058/2020	EPI_ISL_466636	National Institute of Laboratory Medicine and Referral Center	Genomic Research Lab, BCSIR	2020-05-10	Abu Sayeed Mohammad Mahmud et al
Bangladesh/BCSIR-NILMRC_059/2020	EPI_ISL_465164	National Institute of Laboratory Medicine and Referral Center	Genomic Research Lab, BCSIR	2020-05-10	Iffat Jahan et al
Bangladesh/BCSIR-NILMRC_062/2020	EPI_ISL_464166	National Institute of Laboratory Medicine and Referral Center	Genomic Research Lab, BCSIR	2020-05-31	Barna Goswami et al
Bangladesh/BCSIR-NILMRC_066/2020	EPI_ISL_464164	National Institute of Laboratory Medicine and Referral Center	Genomic Research Lab, BCSIR	2020-05-26	Tanjina Akhter Banu et al
Bangladesh/BCSIR-NILMRC_067/2020	EPI_ISL_466637	National Institute of Laboratory Medicine and Referral Center	Genomic Research Lab, BCSIR	2020-05-26	Abu Sayeed Mohammad Mahmud et al
Bangladesh/BCSIR-NILMRC_070/2020	EPI_ISL_466639	National Institute of Laboratory Medicine and Referral Center	Genomic Research Lab, BCSIR	2020-05-26	Abu Sayeed Mohammad Mahmud et al
Bangladesh/BCSIR-NILMRC_071/2020	EPI_ISL_466692	National Institute of Laboratory Medicine and Referral Center	Genomic Research Lab, BCSIR	2020-05-26	Abu Sayeed Mohammad Mahmud et al
Bangladesh/BCSIR-NILMRC_072/2020	EPI_ISL_466687	National Institute of Laboratory Medicine and Referral Center	Genomic Research Lab, BCSIR	2020-05-26	Abu Sayeed Mohammad Mahmud et al
Bangladesh/BCSIR-NILMRC_073/2020	EPI_ISL_466689	National Institute of Laboratory Medicine and Referral Center	Genomic Research Lab, BCSIR	2020-06-01	Abu Sayeed Mohammad Mahmud et al
Bangladesh/BCSIR-NILMRC_074/2020	EPI_ISL_466690	National Institute of Laboratory Medicine and Referral Center	Genomic Research Lab, BCSIR	2020-06-01	Abu Sayeed Mohammad Mahmud et al
Bangladesh/BCSIR-NILMRC_075/2020	EPI_ISL_466691	National Institute of Laboratory Medicine and Referral Center	Genomic Research Lab, BCSIR	2020-06-01	Abu Sayeed Mohammad Mahmud et al
Bangladesh/BCSIR-NILMRC_076/2020	EPI_ISL_466644	National Institute of Laboratory Medicine and Referral Center	Genomic Research Lab, BCSIR	2020-06-01	Abu Sayeed Mohammad Mahmud et al
Bangladesh/BCSIR-NILMRC_077/2020	EPI_ISL_466645	National Institute of Laboratory Medicine and Referral Center	Genomic Research Lab, BCSIR	2020-06-01	Abu Sayeed Mohammad Mahmud et al
Bangladesh/BCSIR-NILMRC_078/2020	EPI_ISL_466693	National Institute of Laboratory Medicine and Referral Center	Genomic Research Lab, BCSIR	2020-06-01	Abu Sayeed Mohammad Mahmud et al
Bangladesh/BCSIR-NILMRC_080/2020	EPI_ISL_464162	National Institute of Laboratory Medicine and Referral Center	Genomic Research Lab, BCSIR	2020-06-01	Md. Ahasan Habib et al
Bangladesh/BCSIR-NILMRC_081/2020	EPI_ISL_466694	National Institute of Laboratory Medicine and Referral Center	Genomic Research Lab, BCSIR	2020-06-01	Abu Sayeed Mohammad Mahmud et al
Bangladesh/BCSIR-NILMRC_082/2020	EPI_ISL_466649	National Institute of Laboratory Medicine and Referral Center	Genomic Research Lab, BCSIR	2020-06-01	Abu Sayeed Mohammad Mahmud et al
Bangladesh/BCSIR-NILMRC_083/2020	EPI_ISL_464161	National Institute of Laboratory Medicine and Referral Center	Genomic Research Lab, BCSIR	2020-06-01	Md. Ahasan Habib et al
Bangladesh/BCSIR-NILMRC_084/2020	EPI_ISL_464160	National Institute of Laboratory Medicine and Referral Center	Genomic Research Lab, BCSIR	2020-06-01	Shahina Akter et al
Bangladesh/BCSIR-NILMRC_085/2020	EPI_ISL_466650	National Institute of Laboratory Medicine and Referral Center	Genomic Research Lab, BCSIR	2020-06-01	Abu Sayeed Mohammad Mahmud et al
Bangladesh/BCSIR-NILMRC_086/2020	EPI_ISL_466688	National Institute of Laboratory Medicine and Referral Center	Genomic Research Lab, BCSIR	2020-06-01	Abu Sayeed Mohammad Mahmud et al
Bangladesh/BCSIR-NILMRC_087/2020	EPI_ISL_464159	National Institute of Laboratory Medicine and Referral Center	Genomic Research Lab, BCSIR	2020-05-09	Shahina Akter et al
Bangladesh/BCSIR-NILMRC_090/2020	EPI_ISL_469285	National Institute of Laboratory Medicine and Referral Center	Genomic Research Lab, BCSIR	2020-06-02	Shahina Akter et al
Bangladesh/BCSIR-NILMRC_091/2020	EPI_ISL_469300	National Institute of Laboratory Medicine and Referral Center	Genomic Research Lab, BCSIR	2020-06-02	Abu Sayeed Mohammad Mahmud et al
Bangladesh/BCSIR-NILMRC_094/2020	EPI_ISL_469286	National Institute of Laboratory Medicine and Referral Center	Genomic Research Lab, BCSIR	2020-05-23	Tanjina Akhter Banu et al
Bangladesh/CHRF_0001/2020	EPI_ISL_437912	Child Health Research Foundation	Child Health Research Lab	2020-04-18	Senjuti Saha et al B (https://dx.doi.org/10.1101/2020.06.07.138800)
Bangladesh/CHRF_0002/2020	EPI_ISL_468070	Child Health Research Foundation	Child Health Research Foundation	2020-04-20	Senjuti Saha et al A
Bangladesh/CHRF_0003/2020	EPI_ISL_468071	Child Health Research Foundation	Child Health Research Foundation	2020-04-17	Senjuti Saha et al A
Bangladesh/CHRF_0004/2020	EPI_ISL_468072	Child Health Research Foundation	Child Health Research Foundation	2020-04-17	Senjuti Saha et al A
Bangladesh/CHRF_0005/2020	EPI_ISL_468073	Child Health Research Foundation	Child Health Research Foundation	2020-05-10	Senjuti Saha et al A
Bangladesh/CHRF_0006/2020	EPI_ISL_468074	Child Health Research Foundation	Child Health Research Foundation	2020-05-17	Senjuti Saha et al A
Bangladesh/CHRF_0007/2020	EPI_ISL_468075	Child Health Research Foundation	Child Health Research Foundation	2020-05-14	Senjuti Saha et al A
Bangladesh/CHRF_0008/2020	EPI_ISL_468076	Child Health Research Foundation	Child Health Research Foundation	2020-05-16	Senjuti Saha et al A
Bangladesh/CHRF_0009/2020	EPI_ISL_468077	Child Health Research Foundation	Child Health Research Foundation	2020-04-08	Senjuti Saha et al A
Bangladesh/CHRF_0010/2020	EPI_ISL_468078	Child Health Research Foundation	Child Health Research Foundation	2020-05-03	Senjuti Saha et al A
Bangladesh/DNAS_CPH_466/2020	EPI_ISL_445216	DNA Solution Ltd.	DNA Solution Ltd.	2020-04-28	Md. Imran Khan et al (https://dx.doi.org/10.1101/2020.06.07.138800)
Bangladesh/DNAS_CPH_471/2020	EPI_ISL_445214	DNA Solution Ltd.	DNA Solution Ltd.	2020-04-28	Md. Imran Khan et al (https://dx.doi.org/10.1101/2020.06.07.138800)
Bangladesh/DU_50761/2020	EPI_ISL_450842	COVID-19 Laboratory	DNA Solution Ltd.	2020-05-06	Sharif Akhteruzzaman et al
Bangladesh/DU_CARS_5586/2020	EPI_ISL_450841	COVID-19 Laboratory	DNA Solution Ltd	2020-05-13	Sharif Akhteruzzaman et al
Bangladesh/icddrb-2083/2020	EPI_ISL_470801	Virology	icddr, b, 68, Shaheed Tajuddin Ahmed Sarani, Dhaka, Dhaka 1212, Bangladesh	2020-06-07	Hossain et al
Bangladesh/NIB_01/2020	EPI_ISL_447904	National Institute of Biotechnology	National Institute of Biotechnology	2020-05-11	Md. Moniruzzaman et al
Beijing/DT-travelHU01/2020	EPI_ISL_452349	Laboratory of Infectious Diseases Center of Beijing Ditan Hospital	Laboratory of Infectious Diseases Center of Beijing Ditan Hospital	2020-03-19	Siyuan Yang et al
Beijing/DT-travelIT03/2020	EPI_ISL_452354	Laboratory of Infectious Diseases Center of Beijing Ditan Hospital	Laboratory of Infectious Diseases Center of Beijing Ditan Hospital	2020-03-07	Siyuan Yang et al
Beijing/DT-WH03/2020	EPI_ISL_452362	Laboratory of Infectious Diseases Center of Beijing Ditan Hospital	Laboratory of Infectious Diseases Center of Beijing Ditan Hospital	2020-02-01	Siyuan Yang et al
Beijing/IVDC-01-06/2020	EPI_ISL_469254	National Institute for Viral Disease Control and Prevention, China CDC	Institute of Viral Disease Control and Prevention, China CDC	2020-06-11	Wenjie Tan et al B
Beijing/IVDC-02-06/2020	EPI_ISL_469255	National Institute for Viral Disease Control and Prevention, China CDC	Institute of Viral Disease Control and Prevention, China CDC	2020-06-11	Xiang Zhao et al
Belgium/20A38/2020	EPI_ISL_452148	CUB Hopital Erasme Laboratoire d'Anatomie Pathologique	CUB Hopital Erasme Laboratoire d'Anatomie Pathologique	2020-04-01	Isabelle Salmon et al
Belgium/20A42/2020	EPI_ISL_452140	CUB Hopital Erasme Laboratoire d'Anatomie Pathologique	CUB Hopital Erasme Laboratoire d'Anatomie Pathologique	2020-04-03	Isabelle Salmon et al
Belgium/AA-0408412/2020	EPI_ISL_462266	KU Leuven, Rega Institute, Clinical and Epidemiological Virology	KU Leuven, Rega Institute, Clinical and Epidemiological Virology	2020-04-08	Tony Wawina-Bokalanga et al
Belgium/AM-0328342/2020	EPI_ISL_462196	KU Leuven, Rega Institute, Clinical and Epidemiological Virology	KU Leuven, Rega Institute, Clinical and Epidemiological Virology	2020-03-28	Tony Wawina-Bokalanga et al
Belgium/AO-04074040/2020	EPI_ISL_462258	KU Leuven, Rega Institute, Clinical and Epidemiological Virology	KU Leuven, Rega Institute, Clinical and Epidemiological Virology	2020-04-07	Tony Wawina-Bokalanga et al
Belgium/BA-02291/2020	EPI_ISL_415159	KU Leuven, Clinical and Epidemiological Virology	KU Leuven, Clinical and Epidemiological Virology	2020-02-29	Bert Vanmechelen et al (https://dx.doi.org/10.1101/2020.05.05.078758)
Belgium/BCM-0324160/2020	EPI_ISL_420417	KU Leuven, Clinical and Epidemiological Virology	KU Leuven, Clinical and Epidemiological Virology	2020-03-24	Joan Marti-Carreras et al (https://dx.doi.org/10.1101/2020.05.05.078758)
Belgium/DA-0319106/2020	EPI_ISL_420362	KU Leuven, Clinical and Epidemiological Virology	KU Leuven, Clinical and Epidemiological Virology	2020-03-18	Joan Marti-Carreras et al (https://dx.doi.org/10.1101/2020.05.05.078758)
Belgium/DMT-0411408/2020	EPI_ISL_462262	KU Leuven, Rega Institute, Clinical and Epidemiological Virology	KU Leuven, Rega Institute, Clinical and Epidemiological Virology	2020-04-11	Tony Wawina-Bokalanga et al
Belgium/DWAEE-030954/2020	EPI_ISL_418983	KU Leuven, Clinical and Epidemiological Virology	KU Leuven, Clinical and Epidemiological Virology	2020-03-09	Bert Vanmechelen et al (https://dx.doi.org/10.1101/2020.05.05.078758)
Belgium/GHB-03021/2020	EPI_ISL_407976	KU Leuven, Clinical and Epidemiological Virology	KU Leuven, Clinical and Epidemiological Virology	2020-02-03	Bert Vanmechelen et al (https://dx.doi.org/10.1101/2020.05.05.078758)
Belgium/HI-0507421/2020	EPI_ISL_462275	KU Leuven, Rega Institute, Clinical and Epidemiological Virology	KU Leuven, Rega Institute, Clinical and Epidemiological Virology	2020-05-07	Tony Wawina-Bokalanga et al
Belgium/HNF-0507417/2020	EPI_ISL_462271	KU Leuven, Rega Institute, Clinical and Epidemiological Virology	KU Leuven, Rega Institute, Clinical and Epidemiological Virology	2020-05-07	Tony Wawina-Bokalanga et al
Belgium/ITM_C262/2020	EPI_ISL_434369	Hospital AZ Rivierenland	Institute of Tropical Medicine	2020-04-02	Philippe Selhorst et al
Belgium/ITM_C366/2020	EPI_ISL_434374	Hospital AZ Rivierenland	Institute of Tropical Medicine	2020-04-08	Philippe Selhorst et al
Belgium/ITM_C447/2020	EPI_ISL_434385	Hospital AZ Rivierenland	Institute of Tropical Medicine	2020-04-11	Philippe Selhorst et al

Belgium/LJA-0401368/2020	EPI_ISL_462222	KU Leuven, Rega Institute, Clinical and Epidemiological Virology	KU Leuven, Rega Institute, Clinical and Epidemiological Virology	2020-04-01	Tony Wawina-Bokalanga et al
Belgium/LY-030575/2020	EPI_ISL_420331	KU Leuven, Clinical and Epidemiological Virology	KU Leuven, Clinical and Epidemiological Virology	2020-03-05	Joan Marti-Carreras et al (https://dx.doi.org/10.1101/2020.05.05.078758)
Belgium/MC-030689/2020	EPI_ISL_420345	KU Leuven, Clinical and Epidemiological Virology	KU Leuven, Clinical and Epidemiological Virology	2020-03-06	Joan Marti-Carreras et al (https://dx.doi.org/10.1101/2020.05.05.078758)
Belgium/MF-030546/2020	EPI_ISL_420444	KU Leuven, Clinical and Epidemiological Virology	KU Leuven, Clinical and Epidemiological Virology	2020-03-05	Joan Marti-Carreras et al (https://dx.doi.org/10.1101/2020.05.05.078758)
Belgium/MJP-0306100/2020	EPI_ISL_420356	KU Leuven, Clinical and Epidemiological Virology	KU Leuven, Clinical and Epidemiological Virology	2020-03-06	Joan Marti-Carreras et al (https://dx.doi.org/10.1101/2020.05.05.078758)
Belgium/ML-0324164/2020	EPI_ISL_420421	KU Leuven, Clinical and Epidemiological Virology	KU Leuven, Clinical and Epidemiological Virology	2020-03-24	Joan Marti-Carreras et al (https://dx.doi.org/10.1101/2020.05.05.078758)
Belgium/MSE-0324121/2020	EPI_ISL_420378	KU Leuven, Clinical and Epidemiological Virology	KU Leuven, Clinical and Epidemiological Virology	2020-03-24	Joan Marti-Carreras et al (https://dx.doi.org/10.1101/2020.05.05.078758)
Belgium/Rega-0327216/2020	EPI_ISL_458186	KU Leuven, Rega Institute, Clinical and Epidemiological Virology	KU Leuven, Rega Institute, Clinical and Epidemiological Virology	2020-03-27	Tony Wawina-Bokalanga et al
Belgium/Rega-0328191/2020	EPI_ISL_458161	KU Leuven, Rega Institute, Clinical and Epidemiological Virology	KU Leuven, Rega Institute, Clinical and Epidemiological Virology	2020-03-28	Tony Wawina-Bokalanga et al
Belgium/Rega-0329233/2020	EPI_ISL_458203	KU Leuven, Rega Institute, Clinical and Epidemiological Virology	KU Leuven, Rega Institute, Clinical and Epidemiological Virology	2020-03-29	Tony Wawina-Bokalanga et al
Belgium/rega-0407282/2020	EPI_ISL_464076	KU Leuven, Rega Institute, Clinical and Epidemiological Virology	KU Leuven, Rega Institute, Clinical and Epidemiological Virology	2020-04-07	Tony Wawina-Bokalanga et al
Belgium/rega-0423298/2020	EPI_ISL_462152	KU Leuven, Rega Institute, Clinical and Epidemiological Virology	KU Leuven, Rega Institute, Clinical and Epidemiological Virology	2020-04-23	Tony Wawina-Bokalanga et al
Belgium/RJ-0507419/2020	EPI_ISL_462273	KU Leuven, Rega Institute, Clinical and Epidemiological Virology	KU Leuven, Rega Institute, Clinical and Epidemiological Virology	2020-05-07	Tony Wawina-Bokalanga et al
Belgium/SA-0409296/2020	EPI_ISL_464090	KU Leuven, Rega Institute, Clinical and Epidemiological Virology	KU Leuven, Rega Institute, Clinical and Epidemiological Virology	2020-04-09	Tony Wawina-Bokalanga et al
Belgium/SG-0507420/2020	EPI_ISL_462274	KU Leuven, Rega Institute, Clinical and Epidemiological Virology	KU Leuven, Rega Institute, Clinical and Epidemiological Virology	2020-05-07	Tony Wawina-Bokalanga et al
Belgium/UGent-15/2020	EPI_ISL_425056	Lab voor klinische biologie	Onderzoeksgroep Virologie	2020-03-25	Laurens Lambrechts et al A (https://dx.doi.org/10.1101/2020.05.05.078758)
Belgium/UGent-17/2020	EPI_ISL_425057	Lab voor klinische biologie	Onderzoeksgroep Virologie	2020-03-26	Laurens Lambrechts et al A (https://dx.doi.org/10.1101/2020.05.05.078758)
Belgium/UGent-48/2020	EPI_ISL_434364	Lab voor klinische biologie	Onderzoeksgroep Virologie	2020-03-30	Nick Vereecke et al
Belgium/UGent-52/2020	EPI_ISL_451165	Lab voor klinische biologie	Onderzoeksgroep Virologie	2020-03-31	Laurens Lambrechts et al B
Belgium/UGent-61/2020	EPI_ISL_451172	Lab voor klinische biologie	Onderzoeksgroep Virologie	2020-04-08	Laurens Lambrechts et al B
Belgium/UGent-65/2020	EPI_ISL_451176	Lab voor klinische biologie	Onderzoeksgroep Virologie	2020-04-08	Nick Vereecke et al
Belgium/UGent-66/2020	EPI_ISL_451177	Lab voor klinische biologie	Onderzoeksgroep Virologie	2020-04-08	Nick Vereecke et al
Belgium/UGent-82/2020	EPI_ISL_468731	Lab voor klinische biologie	Onderzoeksgroep Virologie	2020-03-11	Laurens Lambrechts et al B
Belgium/UGent-94/2020	EPI_ISL_468742	Lab voor klinische biologie	Onderzoeksgroep Virologie	2020-03-13	Nick Vereecke et al
Belgium/UGent-95/2020	EPI_ISL_468743	Lab voor klinische biologie	Onderzoeksgroep Virologie	2020-03-17	Nick Vereecke et al
Belgium/UGent-98/2020	EPI_ISL_468746	Lab voor klinische biologie	Onderzoeksgroep Virologie	2020-03-17	Nick Vereecke et al
Belgium/ULG-0532/2020	EPI_ISL_447132	Department of Clinical Microbiology	GIGA Medical Genomics	2020-03-24	Keith Durkin et al A
Belgium/ULG-10041/2020	EPI_ISL_427347	Department of Clinical Microbiology	GIGA Medical Genomics	2020-03-31	Keith Durkin et al B (https://dx.doi.org/10.1101/2020.05.05.078758)
Belgium/ULG-10046/2020	EPI_ISL_421213	Department of Clinical Microbiology	GIGA Medical Genomics	2020-03-31	Keith Durkin et al B (https://dx.doi.org/10.1101/2020.05.05.078758)
Belgium/ULG-10063/2020	EPI_ISL_424665	Department of Clinical Microbiology	GIGA Medical Genomics	2020-04-05	Keith Durkin et al B (https://dx.doi.org/10.1101/2020.05.05.078758)
Belgium/ULG-10066/2020	EPI_ISL_424629	Department of Clinical Microbiology	GIGA Medical Genomics	2020-04-03	Keith Durkin et al B (https://dx.doi.org/10.1101/2020.05.05.078758)
Belgium/ULG-10072/2020	EPI_ISL_424631	Department of Clinical Microbiology	GIGA Medical Genomics	2020-04-03	Keith Durkin et al B (https://dx.doi.org/10.1101/2020.05.05.078758)
Belgium/ULG-10074/2020	EPI_ISL_424634	Department of Clinical Microbiology	GIGA Medical Genomics	2020-04-03	Keith Durkin et al B (https://dx.doi.org/10.1101/2020.05.05.078758)
Belgium/ULG-10086/2020	EPI_ISL_424644	Department of Clinical Microbiology	GIGA Medical Genomics	2020-04-04	Keith Durkin et al B (https://dx.doi.org/10.1101/2020.05.05.078758)
Belgium/ULG-10101/2020	EPI_ISL_427352	Department of Clinical Microbiology	GIGA Medical Genomics	2020-04-06	Keith Durkin et al B (https://dx.doi.org/10.1101/2020.05.05.078758)
Belgium/ULG-10104/2020	EPI_ISL_427355	Department of Clinical Microbiology	GIGA Medical Genomics	2020-04-06	Keith Durkin et al B (https://dx.doi.org/10.1101/2020.05.05.078758)
Belgium/ULG-10105/2020	EPI_ISL_427356	Department of Clinical Microbiology	GIGA Medical Genomics	2020-04-06	Keith Durkin et al B (https://dx.doi.org/10.1101/2020.05.05.078758)
Belgium/ULG-10118/2020	EPI_ISL_427366	Department of Clinical Microbiology	GIGA Medical Genomics	2020-04-07	Keith Durkin et al B (https://dx.doi.org/10.1101/2020.05.05.078758)
Belgium/ULG-10121/2020	EPI_ISL_427369	Department of Clinical Microbiology	GIGA Medical Genomics	2020-04-07	Keith Durkin et al B (https://dx.doi.org/10.1101/2020.05.05.078758)
Belgium/ULG-10127/2020	EPI_ISL_427373	Department of Clinical Microbiology	GIGA Medical Genomics	2020-04-07	Keith Durkin et al B (https://dx.doi.org/10.1101/2020.05.05.078758)
Belgium/ULG-10132/2020	EPI_ISL_427377	Department of Clinical Microbiology	GIGA Medical Genomics	2020-04-07	Keith Durkin et al B (https://dx.doi.org/10.1101/2020.05.05.078758)
Belgium/ULG-10145/2020	EPI_ISL_447137	Department of Clinical Microbiology	GIGA Medical Genomics	2020-04-17	Keith Durkin et al A
Belgium/ULG-10161/2020	EPI_ISL_447143	Department of Clinical Microbiology	GIGA Medical Genomics	2020-04-18	Keith Durkin et al A
Belgium/ULG-10170/2020	EPI_ISL_455958	Department of Clinical Microbiology	GIGA Medical Genomics	2020-05-11	Keith Durkin et al A
Belgium/ULG-10171/2020	EPI_ISL_455959	Department of Clinical Microbiology	GIGA Medical Genomics	2020-05-11	Keith Durkin et al A
Belgium/ULG-10172/2020	EPI_ISL_455960	Department of Clinical Microbiology	GIGA Medical Genomics	2020-05-08	Keith Durkin et al A
Belgium/ULG-10173/2020	EPI_ISL_455961	Department of Clinical Microbiology	GIGA Medical Genomics	2020-05-08	Keith Durkin et al A
Belgium/ULG-10174/2020	EPI_ISL_455962	Department of Clinical Microbiology	GIGA Medical Genomics	2020-05-08	Keith Durkin et al A
Belgium/ULG-10175/2020	EPI_ISL_455963	Department of Clinical Microbiology	GIGA Medical Genomics	2020-05-08	Keith Durkin et al A
Belgium/ULG-10176/2020	EPI_ISL_455964	Department of Clinical Microbiology	GIGA Medical Genomics	2020-05-09	Keith Durkin et al A
Belgium/ULG-10177/2020	EPI_ISL_455965	Department of Clinical Microbiology	GIGA Medical Genomics	2020-05-09	Keith Durkin et al A
Belgium/ULG-10178/2020	EPI_ISL_455966	Department of Clinical Microbiology	GIGA Medical Genomics	2020-05-09	Keith Durkin et al A
Belgium/ULG-10179/2020	EPI_ISL_455967	Department of Clinical Microbiology	GIGA Medical Genomics	2020-05-09	Keith Durkin et al A
Belgium/ULG-10180/2020	EPI_ISL_455968	Department of Clinical Microbiology	GIGA Medical Genomics	2020-05-09	Keith Durkin et al A
Belgium/ULG-10181/2020	EPI_ISL_455969	Department of Clinical Microbiology	GIGA Medical Genomics	2020-05-10	Keith Durkin et al A
Belgium/ULG-10182/2020	EPI_ISL_455970	Department of Clinical Microbiology	GIGA Medical Genomics	2020-05-10	Keith Durkin et al A
Belgium/ULG-10183/2020	EPI_ISL_455971	Department of Clinical Microbiology	GIGA Medical Genomics	2020-05-09	Keith Durkin et al A
Belgium/ULG-10184/2020	EPI_ISL_455972	Department of Clinical Microbiology	GIGA Medical Genomics	2020-05-10	Keith Durkin et al A
Belgium/ULG-10185/2020	EPI_ISL_455973	Department of Clinical Microbiology	GIGA Medical Genomics	2020-05-10	Keith Durkin et al A
Belgium/ULG-10186/2020	EPI_ISL_455974	Department of Clinical Microbiology	GIGA Medical Genomics	2020-05-11	Keith Durkin et al A
Belgium/ULG-10187/2020	EPI_ISL_455975	Department of Clinical Microbiology	GIGA Medical Genomics	2020-05-11	Keith Durkin et al A
Belgium/ULG-10188/2020	EPI_ISL_455976	Department of Clinical Microbiology	GIGA Medical Genomics	2020-05-11	Keith Durkin et al A
Belgium/ULG-10189/2020	EPI_ISL_455977	Department of Clinical Microbiology	GIGA Medical Genomics	2020-05-11	Keith Durkin et al A
Belgium/ULG-10190/2020	EPI_ISL_455978	Department of Clinical Microbiology	GIGA Medical Genomics	2020-05-10	Keith Durkin et al A
Belgium/ULG-10191/2020	EPI_ISL_455979	Department of Clinical Microbiology	GIGA Medical Genomics	2020-05-11	Keith Durkin et al A
Belgium/ULG-6649/2020	EPI_ISL_418631	Department of Clinical Microbiology	GIGA Medical Genomics	2020-03-14	Keith Durkin et al B (https://dx.doi.org/10.1101/2020.05.05.078758)
Belgium/ULG-6972/2020	EPI_ISL_418624	Department of Clinical Microbiology	GIGA Medical Genomics	2020-03-15	Keith Durkin et al B (https://dx.doi.org/10.1101/2020.05.05.078758)
Belgium/ULG-9725/2020	EPI_ISL_418659	Department of Clinical Microbiology	GIGA Medical Genomics	2020-03-22	Keith Durkin et al B (https://dx.doi.org/10.1101/2020.05.05.078758)
Belgium/VAM-0506416/2020	EPI_ISL_462270	KU Leuven, Rega Institute, Clinical and Epidemiological Virology	KU Leuven, Rega Institute, Clinical and Epidemiological Virology	2020-05-06	Tony Wawina-Bokalanga et al
Belgium/VLS-0507418/2020	EPI_ISL_462272	KU Leuven, Rega Institute, Clinical and Epidemiological Virology	KU Leuven, Rega Institute, Clinical and Epidemiological Virology	2020-05-07	Tony Wawina-Bokalanga et al
BosniaandHerzegovina/01-Livno/2020	EPI_ISL_462753	University Clinical Hospital of Mostar	University of Sarajevo Veterinary Faculty	2020-05-27	Goletic et al
BosniaandHerzegovina/02-BanjaLuka/2020	EPI_ISL_462990	University Clinical Centre of the Republic of Srpska	University of Sarajevo, Veterinary Faculty	2020-04-29	Teufik et al
BosniaandHerzegovina/04-Sarajevo/2020	EPI_ISL_467300	General Hospital "Abdulah Nakas"	Alea Genetic Center	2020-04-08	Rijad Konjhodzic et al
BosniaandHerzegovina/ChVir7343/2020	EPI_ISL_462451	Clinical Center, University of Sarajevo	Charite Universitatsmedizin Berlin, Institute of Virology	2020-03-19	Victor M Corman et al A
BosniaandHerzegovina/ChVir7344/2020	EPI_ISL_462452	Clinical Center, University of Sarajevo	Charite Universitatsmedizin Berlin, Institute of Virology	2020-03-19	Victor M Corman et al A
BosniaandHerzegovina/ChVir7346/2020	EPI_ISL_462453	Clinical Center, University of Sarajevo	Charite Universitatsmedizin Berlin, Institute of Virology	2020-03-20	Victor M Corman et al A
BosniaandHerzegovina/ChVir7347/2020	EPI_ISL_462454	Clinical Center, University of Sarajevo	Charite Universitatsmedizin Berlin, Institute of Virology	2020-03-20	Victor M Corman et al A
BosniaandHerzegovina/ChVir7348/2020	EPI_ISL_462455	Clinical Center, University of Sarajevo	Charite Universitatsmedizin Berlin, Institute of Virology	2020-03-20	Victor M Corman et al A
BosniaandHerzegovina/ChVir7349/2020	EPI_ISL_462456	Clinical Center, University of Sarajevo	Charite Universitatsmedizin Berlin, Institute of Virology	2020-03-20	Victor M Corman et al A
BosniaandHerzegovina/ChVir7350/2020	EPI_ISL_462457	Clinical Center, University of Sarajevo	Charite Universitatsmedizin Berlin, Institute of Virology	2020-03-20	Victor M Corman et al A
BosniaandHerzegovina/ChVir7352/2020	EPI_ISL_462458	Clinical Center, University of Sarajevo	Charite Universitatsmedizin Berlin, Institute of Virology	2020-03-21	Victor M Corman et al A
BosniaandHerzegovina/ChVir7353/2020	EPI_ISL_462459	Clinical Center, University of Sarajevo	Charite Universitatsmedizin Berlin, Institute of Virology	2020-03-21	Victor M Corman et al A
BosniaandHerzegovina/ChVir7354/2020	EPI_ISL_462460	Clinical Center, University of Sarajevo	Charite Universitatsmedizin Berlin, Institute of Virology	2020-03-22	Victor M Corman et al A
BosniaandHerzegovina/ChVir7357/2020	EPI_ISL_462462	Clinical Center, University of Sarajevo	Charite Universitatsmedizin Berlin, Institute of Virology	2020-03-23	Victor M Corman et al A
BosniaandHerzegovina/ChVir7360/2020	EPI_ISL_462463	Clinical Center, University of Sarajevo	Charite Universitatsmedizin Berlin, Institute of Virology	2020-03-25	Victor M Corman et al A
BosniaandHerzegovina/ChVir7361/2020	EPI_ISL_462464	Clinical Center, University of Sarajevo	Charite Universitatsmedizin Berlin, Institute of Virology	2020-03-25	Victor M Corman et al A
BosniaandHerzegovina/ChVir7362/2020	EPI_ISL_462465	Clinical Center, University of Sarajevo	Charite Universitatsmedizin Berlin, Institute of Virology	2020-03-25	Victor M Corman et al A
BosniaandHerzegovina/ChVir7365/2020	EPI_ISL_462467	Clinical Center, University of Sarajevo	Charite Universitatsmedizin Berlin, Institute of Virology	2020-03-26	Victor M Corman et al A
BosniaandHerzegovina/ChVir7366/2020	EPI_ISL_462468	Clinical Center, University of Sarajevo	Charite Universitatsmedizin Berlin, Institute of Virology	2020-03-27	Victor M Corman et al A
BosniaandHerzegovina/ChVir7367/2020	EPI_ISL_462469	Clinical Center, University of Sarajevo	Charite Universitatsmedizin Berlin, Institute of Virology	2020-03-27	Victor M Corman et al A
BosniaandHerzegovina/ChVir7370/2020	EPI_ISL_462471	Clinical Center, University of Sarajevo	Charite Universitatsmedizin Berlin, Institute of Virology	2020-03-28	Victor M Corman et al A
BosniaandHerzegovina/ChVir7372/2020	EPI_ISL_462473	Clinical Center, University of Sarajevo	Charite Universitatsmedizin Berlin, Institute of Virology	2020-03-29	Victor M Corman et al A
BosniaandHerzegovina/ChVir7375/2020	EPI_ISL_462474	Clinical Center, University of Sarajevo	Charite Universitatsmedizin Berlin, Institute of Virology	2020-03-29	Victor M Corman et al A
BosniaandHerzegovina/ChVir7377/2020	EPI_ISL_462475	Clinical Center, University of Sarajevo	Charite Universitatsmedizin Berlin, Institute of Virology	2020-03-30	Victor M Corman et al A
BosniaandHerzegovina/ChVir7379/2020	EPI_ISL_462476	Clinical Center, University of Sarajevo	Charite Universitatsmedizin Berlin, Institute of Virology	2020-03-29	Victor M Corman et al A
BosniaandHerzegovina/ChVir7382/2020	EPI_ISL_462472	Clinical Center, University of Sarajevo	Charite Universitatsmedizin Berlin, Institute of Virology	2020-04-01	Victor M Corman et al A
BosniaandHerzegovina/ChVir7387/2020	EPI_ISL_462461	Clinical Center, University of Sarajevo	Charite Universitatsmedizin Berlin, Institute of Virology	2020-04-11	Victor M Corman et al A
BosniaandHerzegovina/ChVir7396/2020	EPI_ISL_462466	Clinical Center, University of Sarajevo	Charite Universitatsmedizin Berlin, Institute of Virology	2020-04-21	Victor M Corman et al A
BosniaandHerzegovina/ChVir7402/2020	EPI_ISL_462470	Clinical Center, University of Sarajevo	Charite Universitatsmedizin Berlin, Institute of Virology	2020-04-22	Victor M Corman et al A
Brazil/AM0204/2020	EPI_ISL_470571	Hermes Pardini	Bioinformatics Laboratory / LNCC	2020-04-02	Alexandra Gerber et al
Brazil/AP161167-IEC/2020	EPI_ISL_450873	Evandro Chagas Institute	Evandro Chagas Institute	2020-03-17	Santos et al (https://dx.doi.org/10.1101/2020.06.11.20128249)
Brazil/AP164346-IEC/2020	EPI_ISL_458145	Evandro Chagas Institute	Evandro Chagas Institute	2020-04-20	Santos et al (https://dx.doi.org/10.1101/2020.06.11.20128249)
Brazil/CV26/2020	EPI_ISL_429684	Central Public Health Laboratory/Octávio Magalhães Institute (IOM) from the Ezequiel Dias Foundation (FUNED)	Instituto Octávio Magalhães / Fundação Ezequiel Dias (IOM/Funed)	2020-03-17	Talita Adelino et al B (https://dx.doi.org/10.1101/2020.06.11.20128249)
Brazil/CV4/2020	EPI_ISL_429667	Central Public Health Laboratory/Octávio Magalhães Institute (IOM) from the Ezequiel Dias Foundation (FUNED)	Instituto Octávio Magalhães / Fundação Ezequiel Dias (IOM/Funed)	2020-03-09	Talita Adelino et al A (https://dx.doi.org/10.1101/2020.05.23.20111443)
Brazil/CV42/2020	EPI_ISL_429695	Central Public Health Laboratory/Octávio Magalhães Institute (IOM) from the Ezequiel Dias Foundation (FUNED)	Instituto Octávio Magalhães / Fundação Ezequiel Dias (IOM/Funed)	2020-03-20	Talita Adelino et al A (https://dx.doi.org/10.1101/2020.05.23.20111443)
Brazil/CV43/2020	EPI_ISL_429696	Central Public Health Laboratory/Octávio Magalhães Institute (IOM) from the Ezequiel Dias Foundation (FUNED)	Instituto Octávio Magalhães / Fundação Ezequiel Dias (IOM/Funed)	2020-03-17	Talita Adelino et al C
Brazil/CV8/2020	EPI_ISL_429671	Central Public Health Laboratory/Octávio Magalhães Institute (IOM) from the Ezequiel Dias Foundation (FUNED)	Instituto Octávio Magalhães / Fundação Ezequiel Dias (IOM/Funed)	2020-03-13	Talita Adelino et al B (https://dx.doi.org/10.1101/2020.06.11.20128249)
Brazil/DF-619i/2020	EPI_ISL_427295	Instituto Oswaldo Cruz FIOCRUZ - Laboratory of Respiratory Viruses and Measles (LVRS)	Instituto Oswaldo Cruz FIOCRUZ - Laboratory of Respiratory Viruses and Measles (LVRS)	2020-03-13	Paola Resende et al C (https://dx.doi.org/10.1101/2020.06.11.20128249)
Brazil/GO0210/2020	EPI_ISL_470577	Hermes Pardini	Bioinformatics Laboratory / LNCC	2020-04-04	Alexandra Gerber et al
Brazil/MG0211/2020	EPI_ISL_470578	Hermes Pardini	Bioinformatics Laboratory / LNCC	2020-03-14	Alexandra Gerber et al
Brazil/MG0217/2020	EPI_ISL_470583	Hermes Pardini	Bioinformatics Laboratory / LNCC	2020-03-20	Alexandra Gerber et al
Brazil/MG0221/2020	EPI_ISL_470587	Hermes Pardini	Bioinformatics Laboratory / LNCC	2020-04-05	Alexandra Gerber et al
Brazil/MG0286/2020	EPI_ISL_470591	Simile	Bioinformatics Laboratory / LNCC	2020-04-17	Alexandra Gerber et al
Brazil/MG0289/2020	EPI_ISL_470594	Simile	Bioinformatics Laboratory / LNCC	2020-04-08	Alexandra Gerber et al

Brazil/MG0290/2020	EPI_ISL_470595	Simile	Bioinformatics Laboratory / LNCC	2020-04-03	Alexandra Gerber et al
Brazil/MT0225/2020	EPI_ISL_470599	Hermes Pardini	Bioinformatics Laboratory / LNCC	2020-03-19	Alexandra Gerber et al
Brazil/PA0232/2020	EPI_ISL_470606	Hermes Pardini	Bioinformatics Laboratory / LNCC	2020-04-02	Alexandra Gerber et al
Brazil/PA0235/2020	EPI_ISL_470609	Hermes Pardini	Bioinformatics Laboratory / LNCC	2020-04-03	Alexandra Gerber et al
Brazil/PI0239/2020	EPI_ISL_470613	Hermes Pardini	Bioinformatics Laboratory / LNCC	2020-03-19	Alexandra Gerber et al
Brazil/PR0241/2020	EPI_ISL_470614	Hermes Pardini	Bioinformatics Laboratory / LNCC	2020-04-02	Alexandra Gerber et al
Brazil/RJ-1056/2020	EPI_ISL_456072	Laboratory of Respiratory Viruses and Measles, Oswaldo Cruz Institute, FIOCRUZ	Laboratory of Respiratory Viruses and Measles, Oswaldo Cruz Institute, FIOCRUZ	2020-04-01	Paola Resende et al C (https://dx.doi.org/10.1101/2020.06.11.20128249)
Brazil/RJ-1100/2020	EPI_ISL_456075	Laboratory of Respiratory Viruses and Measles, Oswaldo Cruz Institute, FIOCRUZ	Laboratory of Respiratory Viruses and Measles, Oswaldo Cruz Institute, FIOCRUZ	2020-04-02	Paola Resende et al C (https://dx.doi.org/10.1101/2020.06.11.20128249)
Brazil/RJ-1119/2020	EPI_ISL_456077	LACEN RJ - Laboratório Central de Saúde Pública Noel Nutels	Laboratory of Respiratory Viruses and Measles, Oswaldo Cruz Institute, FIOCRUZ	2020-03-25	Paola Resende et al C (https://dx.doi.org/10.1101/2020.06.11.20128249)
Brazil/RJ-1464/2020	EPI_ISL_456080	Laboratory of Respiratory Viruses and Measles, Oswaldo Cruz Institute, FIOCRUZ	Laboratory of Respiratory Viruses and Measles, Oswaldo Cruz Institute, FIOCRUZ	2020-04-06	Paola Resende et al C (https://dx.doi.org/10.1101/2020.06.11.20128249)
Brazil/RJ-2044/2020	EPI_ISL_456101	Laboratory of Respiratory Viruses and Measles, Oswaldo Cruz Institute, FIOCRUZ	Laboratory of Respiratory Viruses and Measles, Oswaldo Cruz Institute, FIOCRUZ	2020-04-15	Paola Resende et al C (https://dx.doi.org/10.1101/2020.06.11.20128249)
Brazil/RJ-2062/2020	EPI_ISL_456103	Laboratory of Respiratory Viruses and Measles, Oswaldo Cruz Institute, FIOCRUZ	Laboratory of Respiratory Viruses and Measles, Oswaldo Cruz Institute, FIOCRUZ	2020-04-16	Paola Resende et al C (https://dx.doi.org/10.1101/2020.06.11.20128249)
Brazil/RJ-2197/2020	EPI_ISL_467349	Laboratory of Respiratory Viruses and Measles, Oswaldo Cruz Institute, FIOCRUZ	Laboratory of Respiratory Viruses and Measles, Oswaldo Cruz Institute, FIOCRUZ	2020-04-17	Paola Resende et al B
Brazil/RJ-2683/2020	EPI_ISL_467357	Laboratory of Respiratory Viruses and Measles, Oswaldo Cruz Institute, FIOCRUZ	Laboratory of Respiratory Viruses and Measles, Oswaldo Cruz Institute, FIOCRUZ	2020-04-24	Paola Resende et al B
Brazil/RJ-2696/2020	EPI_ISL_467358	Laboratory of Respiratory Viruses and Measles, Oswaldo Cruz Institute, FIOCRUZ	Laboratory of Respiratory Viruses and Measles, Oswaldo Cruz Institute, FIOCRUZ	2020-04-24	Paola Resende et al B
Brazil/RJ-2840/2020	EPI_ISL_467368	Laboratory of Respiratory Viruses and Measles, Oswaldo Cruz Institute, FIOCRUZ	Laboratory of Respiratory Viruses and Measles, Oswaldo Cruz Institute, FIOCRUZ	2020-04-28	Paola Resende et al B
Brazil/RJ-2844/2020	EPI_ISL_467369	Laboratory of Respiratory Viruses and Measles, Oswaldo Cruz Institute, FIOCRUZ	Laboratory of Respiratory Viruses and Measles, Oswaldo Cruz Institute, FIOCRUZ	2020-04-28	Paola Resende et al B
Brazil/RJ-2847/2020	EPI_ISL_467370	Laboratory of Respiratory Viruses and Measles, Oswaldo Cruz Institute, FIOCRUZ	Laboratory of Respiratory Viruses and Measles, Oswaldo Cruz Institute, FIOCRUZ	2020-04-28	Paola Resende et al B
Brazil/RJ-2868/2020	EPI_ISL_467371	Laboratory of Respiratory Viruses and Measles, Oswaldo Cruz Institute, FIOCRUZ	Laboratory of Respiratory Viruses and Measles, Oswaldo Cruz Institute, FIOCRUZ	2020-04-28	Paola Resende et al B
Brazil/RJ-314/2020	EPI_ISL_414045	LACEN RJ - Laboratório Central de Saúde Pública Noel Nutels	Instituto Oswaldo Cruz FIOCRUZ - Laboratory of Respiratory Viruses and Measles (LVRS)	2020-03-04	Paola Resende et al A (https://dx.doi.org/10.1101/2020.05.23.20111443)
Brazil/RJ-763/2020	EPI_ISL_427302	Instituto Oswaldo Cruz FIOCRUZ - Laboratory of Respiratory Viruses and Measles (LVRS)	Instituto Oswaldo Cruz FIOCRUZ - Laboratory of Respiratory Viruses and Measles (LVRS)	2020-03-20	Paola Resende et al C (https://dx.doi.org/10.1101/2020.06.11.20128249)
Brazil/RJ-899/2020	EPI_ISL_456071	Laboratory of Respiratory Viruses and Measles, Oswaldo Cruz Institute, FIOCRUZ	Laboratory of Respiratory Viruses and Measles, Oswaldo Cruz Institute, FIOCRUZ	2020-03-30	Paola Resende et al C (https://dx.doi.org/10.1101/2020.06.11.20128249)
Brazil/RJ0116/2020	EPI_ISL_417982	Universidade Federal do Rio de Janeiro	Bioinformatics Laboratory - LNCC	2020-03-19	Filipe Romero et al
Brazil/RJ0253/2020	EPI_ISL_470621	Laboratorio de Virologia Molecular / UFRJ	Bioinformatics Laboratory / LNCC	2020-03-31	Alexandra Gerber et al
Brazil/RJ0256/2020	EPI_ISL_470624	Laboratorio de Virologia Molecular / UFRJ	Bioinformatics Laboratory / LNCC	2020-04-03	Alexandra Gerber et al
Brazil/RJ0266/2020	EPI_ISL_470632	Laboratorio de Virologia Molecular / UFRJ	Bioinformatics Laboratory / LNCC	2020-04-14	Alexandra Gerber et al
Brazil/RJ0267/2020	EPI_ISL_470633	Laboratorio de Virologia Molecular / UFRJ	Bioinformatics Laboratory / LNCC	2020-04-15	Alexandra Gerber et al
Brazil/RJ0268/2020	EPI_ISL_470634	Laboratorio de Virologia Molecular / UFRJ	Bioinformatics Laboratory / LNCC	2020-04-15	Alexandra Gerber et al
Brazil/RJ0273/2020	EPI_ISL_470639	Laboratorio de Virologia Molecular / UFRJ	Bioinformatics Laboratory / LNCC	2020-04-20	Alexandra Gerber et al
Brazil/RJ0275/2020	EPI_ISL_470641	Laboratorio de Virologia Molecular / UFRJ	Bioinformatics Laboratory / LNCC	2020-04-20	Alexandra Gerber et al
Brazil/RS0242/2020	EPI_ISL_470651	Hermes Pardini	Bioinformatics Laboratory / LNCC	2020-03-20	Alexandra Gerber et al
Brazil/SC-769/2020	EPI_ISL_427306	LACEN-SC - Laboratorio Central de Santa Catarina	Instituto Oswaldo Cruz FIOCRUZ - Laboratory of Respiratory Viruses and Measles (LVRS)	2020-03-10	Paola Resende et al A (https://dx.doi.org/10.1101/2020.05.23.20111443)
Brazil/SPBR-01/2020	EPI_ISL_412964	Hospital Israelita Albert Einstein	Instituto Adolfo Lutz Interdisciplinary Procedures Center Strategic Laboratory	2020-02-25	Jaqueline Goes de Jesus et al (https://virological.org/t/first-cases-of-coronavirus-disease-covid-19-in-brazil-south-america-2-genomes-3rd-march-2020/409)
Brazil/SPBR-03/2020	EPI_ISL_414014	Hospital Israelita Albert Einstein	Instituto Adolfo Lutz, Interdiciplinary Procedures Center, Strategic Laboratory	2020-03-02	Claudio Tavares Sacchi et al B (https://dx.doi.org/10.1590/s1678-9946202062030)
Brazil/SPBR-05/2020	EPI_ISL_414016	Hospital São Joaquim Beneficencia Portuguesa	Instituto Adolfo Lutz, Interdiciplinary Procedures Center, Strategic Laboratory	2020-02-29	Claudio Tavares Sacchi et al B (https://dx.doi.org/10.1590/s1678-9946202062030)
Brazil/SPBR-06/2020	EPI_ISL_414015	Hospital São Joaquim Beneficencia Portuguesa	Instituto Adolfo Lutz, Interdiciplinary Procedures Center, Strategic Laboratory	2020-02-29	Claudio Tavares Sacchi et al B (https://dx.doi.org/10.1590/s1678-9946202062030)
Brazil/SPBR-07/2020	EPI_ISL_416028	National Influenza Center - Instituto Adolfo Lutz	Instituto Adolfo Lutz, Interdiciplinary Procedures Center, Strategic Laboratory	2020-03-03	Claudio Tavares Sacchi et al A (https://dx.doi.org/10.1101/2020.05.23.20111443)
Brazil/SPBR-08/2020	EPI_ISL_416029	Laboiratório Fleury	Instituto Adolfo Lutz, Interdiciplinary Procedures Center, Strategic Laboratory	2020-03-04	Claudio Tavares Sacchi et al A (https://dx.doi.org/10.1101/2020.05.23.20111443)
Brazil/SPBR-11/2020	EPI_ISL_416033	Hospital Israelita Albert Einstein	Instituto Adolfo Lutz, Interdiciplinary Procedures Center, Strategic Laboratory	2020-03-03	Claudio Tavares Sacchi et al A (https://dx.doi.org/10.1101/2020.05.23.20111443)
Brazil/SPBR-12/2020	EPI_ISL_416034	Hospital Israelita Albert Einstein	Instituto Adolfo Lutz, Interdiciplinary Procedures Center, Strategic Laboratory	2020-03-04	Claudio Tavares Sacchi et al A (https://dx.doi.org/10.1101/2020.05.23.20111443)
Brazil/SPBR-13/2020	EPI_ISL_416035	National Influenza Center - Instituto Adolfo Lutz	Instituto Adolfo Lutz, Interdiciplinary Procedures Center, Strategic Laboratory	2020-03-05	Claudio Tavares Sacchi et al A (https://dx.doi.org/10.1101/2020.05.23.20111443)
Brazil/SPBR-140/2020	EPI_ISL_468307	Centro de Vigilancia a Saude de Diadema	Instituto Adolfo Lutz, Interdiciplinary Procedures Center, Strategic Laboratory	2020-04-08	Claudio Tavares Sacchi et al C
Brazil/SPBR-146/2020	EPI_ISL_468311	Hospital Municipal Dr Ignacio Proenca de Gouvea	Instituto Adolfo Lutz, Interdiciplinary Procedures Center, Strategic Laboratory	2020-03-26	Claudio Tavares Sacchi et al C
Brazil/SPBR-147/2020	EPI_ISL_468312	Hospital Municipal Dr Ignacio Proenca de Gouvea	Instituto Adolfo Lutz, Interdiciplinary Procedures Center, Strategic Laboratory	2020-03-26	Claudio Tavares Sacchi et al C
Brazil/SPBR-148/2020	EPI_ISL_468313	Vigilancia Epidemiologica de São Bernardo do Campo	Instituto Adolfo Lutz, Interdiciplinary Procedures Center, Strategic Laboratory	2020-03-23	Claudio Tavares Sacchi et al C
Brazil/SPBR-149/2020	EPI_ISL_468314	CTA Centro de Testagem e Aconselhamento	Instituto Adolfo Lutz, Interdiciplinary Procedures Center, Strategic Laboratory	2020-03-26	Claudio Tavares Sacchi et al C
Brunei/1/2020	EPI_ISL_443187	National Virology Reference Laboratory	National Public Health Laboratory, National Centre for Infectious Diseases	2020-03-11	Mak Tze Minn et al
Brunei/2/2020	EPI_ISL_435674	National Virology Reference Laboratory	National Public Health Laboratory, National Centre for Infectious Diseases	2020-03-11	Mak Tze Minn et al
Brunei/3/2020	EPI_ISL_435675	National Virology Reference Laboratory	National Public Health Laboratory, National Centre for Infectious Diseases	2020-03-11	Mak Tze Minn et al
Brunei/4/2020	EPI_ISL_435676	National Virology Reference Laboratory	National Public Health Laboratory, National Centre for Infectious Diseases	2020-03-21	Mak Tze Minn et al
Brunei/5/2020	EPI_ISL_435677	National Virology Reference Laboratory	National Public Health Laboratory, National Centre for Infectious Diseases	2020-03-21	Mak Tze Minn et al
Bucuresti/ChVir7244/2020	EPI_ISL_467778	National Influenza Centre Romania	Charite Universitatsmedizin Berlin, Institute of Virology	2020-04-27	Victor M Corman et al A
Bucuresti/ChVir7245/2020	EPI_ISL_467779	National Influenza Centre Romania	Charite Universitatsmedizin Berlin, Institute of Virology	2020-04-27	Victor M Corman et al A
Cambodia/0012/2020	EPI_ISL_411902	Virology Unit, Institut Pasteur du Cambodge.	Virology Unit, Institut Pasteur du Cambodge (Sequencing done by: Jessica E Manning/Jennifer A Bohl at Malaria and Vector Research Research Laboratory, National Institute of Allergy and Infectious Diseases and Vida Ahyong from Chan-Zuckerberg Biohub)	2020-01-27	Erik A Karlsson et al (https://dx.doi.org/10.2807/1560-7917.ES.2020.25.13.2000305)
Canada/BC_02421/2020	EPI_ISL_415581	BCCDC Public Health Laboratory	BCCDC Public Health Laboratory	2020-03-01	Harrigan et al A
Canada/BC_13297/2020	EPI_ISL_415578	BCCDC Public Health Laboratory	BCCDC Public Health Laboratory	2020-02-27	Harrigan et al A
Canada/BC_17397/2020	EPI_ISL_415580	BCCDC Public Health Laboratory	BCCDC Public Health Laboratory	2020-02-28	Harrigan et al A
Canada/BC_35720/2020	EPI_ISL_415582	BCCDC Public Health Laboratory	BCCDC Public Health Laboratory	2020-03-04	Harrigan et al A
Canada/BC_37_0-2/2020	EPI_ISL_412965	BCCDC Public Health Laboratory	BCCDC Public Health Laboratory	2020-02-16	Harrigan et al B (https://dx.doi.org/10.1002/jmv.25773)
Canada/BC_3808524/2020	EPI_ISL_418819	BCCDC Public Health Laboratory	BCCDC Public Health Laboratory	2020-03-09	Harrigan et al A
Canada/BC_4540462/2020	EPI_ISL_418829	BCCDC Public Health Laboratory	BCCDC Public Health Laboratory	2020-03-10	Harrigan et al A
Canada/BC_5979789/2020	EPI_ISL_418835	BCCDC Public Health Laboratory	BCCDC Public Health Laboratory	2020-03-11	Harrigan et al A
Canada/BC_64686/2020	EPI_ISL_415586	BCCDC Public Health Laboratory	BCCDC Public Health Laboratory	2020-03-05	Harrigan et al A
Canada/BC_65034/2020	EPI_ISL_415585	BCCDC Public Health Laboratory	BCCDC Public Health Laboratory	2020-03-04	Harrigan et al A
Canada/BC_69243/2020	EPI_ISL_415577	BCCDC Public Health Laboratory	BCCDC Public Health Laboratory	2020-02-20	Harrigan et al A
Canada/BC_6981299/2020	EPI_ISL_418841	BCCDC Public Health Laboratory	BCCDC Public Health Laboratory	2020-03-11	Harrigan et al A
Canada/BC_6997898/2020	EPI_ISL_418842	BCCDC Public Health Laboratory	BCCDC Public Health Laboratory	2020-03-11	Harrigan et al A
Canada/NL_96/2020	EPI_ISL_469235	Public Health Laboratory	National Microbiology Laboratory	2020-03-22	Anna Majer et al
Canada/NS_13/2020	EPI_ISL_418814	Queen Elizabeth II Health Science Centre	National Microbiology Laboratory	2020-03-13	Anna Majer et al
Canada/NS_31/2020	EPI_ISL_429813	Queen Elizabeth II Health Science Centre	National Microbiology Laboratory	2020-03-15	Anna Majer et al
Canada/ON_MU-S350/2020	EPI_ISL_463983	Toronto Invasive Bacterial Diseases Network	McMaster University	2020-03-29	Allison McGeer et al
Canada/ON_PHL0743/2020	EPI_ISL_418346	Public Health Ontario Laboratories	Public Health Ontario Laboratories	2020-03-07	Alireza Eshaghi et al
Canada/ON_PHL0976/2020	EPI_ISL_418378	Public Health Ontario Laboratories	Public Health Ontario Laboratories	2020-03-13	Alireza Eshaghi et al
Canada/ON_PHL2259/2020	EPI_ISL_418344	Public Health Ontario Laboratories	Public Health Ontario Laboratories	2020-02-29	Alireza Eshaghi et al
Canada/ON_PHL2273/2020	EPI_ISL_418383	Public Health Ontario Laboratories	Public Health Ontario Laboratories	2020-02-29	Alireza Eshaghi et al
Canada/ON_PHL3380/2020	EPI_ISL_418350	Public Health Ontario Laboratories	Public Health Ontario Laboratories	2020-03-20	Alireza Eshaghi et al
Canada/ON_PHL3917/2020	EPI_ISL_418322	Public Health Ontario Laboratories	Public Health Ontario Laboratories	2020-03-08	Alireza Eshaghi et al
Canada/ON_PHL5472/2020	EPI_ISL_418325	Public Health Ontario Laboratories	Public Health Ontario Laboratories	2020-02-20	Alireza Eshaghi et al
Canada/ON_PHL5705/2020	EPI_ISL_418336	Public Health Ontario Laboratories	Public Health Ontario Laboratories	2020-03-12	Alireza Eshaghi et al
Canada/ON_PHL5930/2020	EPI_ISL_418382	Public Health Ontario Laboratories	Public Health Ontario Laboratories	2020-02-28	Alireza Eshaghi et al
Canada/ON_PHL8751/2020	EPI_ISL_418345	Public Health Ontario Laboratories	Public Health Ontario Laboratories	2020-02-29	Alireza Eshaghi et al
Canada/ON_QGLO-016/2020	EPI_ISL_459873	Kingston Health Sciences Center	Queen's Genomics Lab at Ongwanada (Q-GLO)	2020-03-26	Sjaarda CP et al
Canada/ON_QGLO-017/2020	EPI_ISL_459874	Kingston Health Sciences Center	Queen's Genomics Lab at Ongwanada (Q-GLO)	2020-03-27	Sjaarda CP et al
Canada/ON_QGLO-034/2020	EPI_ISL_459885	Kingston Health Sciences Center	Queen's Genomics Lab at Ongwanada (Q-GLO)	2020-03-29	Sjaarda CP et al
Canada/ON_QGLO-042/2020	EPI_ISL_459892	Kingston Health Sciences Center	Queen's Genomics Lab at Ongwanada (Q-GLO)	2020-03-30	Sjaarda CP et al
Canada/ON_SHSC-01/2020	EPI_ISL_450747	Sunnybrook Health Sciences Centre	Department of Laboratory Medicine and Molecular Diagnostics, Sunnybrook Health Sciences Centre	2020-02-26	Jalees A. Nasir et al
Canada/ON-UHTC_0005/2020	EPI_ISL_464043	Unity Health Toronto	Ontario Institute for Cancer Research	2020-03-24	Ramzi Fattouh et al
Canada/ON-UHTC_0021/2020	EPI_ISL_464027	Unity Health Toronto	Ontario Institute for Cancer Research	2020-03-30	Ramzi Fattouh et al
Canada/ON-UHTC_0024/2020	EPI_ISL_464035	Unity Health Toronto	Ontario Institute for Cancer Research	2020-04-01	Ramzi Fattouh et al
Canada/ON-UHTC_0029/2020	EPI_ISL_464060	Unity Health Toronto	Ontario Institute for Cancer Research	2020-04-01	Ramzi Fattouh et al
Canada/ON-UHTC_0035/2020	EPI_ISL_464033	Unity Health Toronto	Ontario Institute for Cancer Research	2020-04-07	Ramzi Fattouh et al
Canada/ON-UHTC_0041/2020	EPI_ISL_464037	Unity Health Toronto	Ontario Institute for Cancer Research	2020-04-03	Ramzi Fattouh et al
Canada/ON-UHTC_0049/2020	EPI_ISL_464013	Unity Health Toronto	Ontario Institute for Cancer Research	2020-04-05	Ramzi Fattouh et al
Canada/ON-UHTC_0066/2020	EPI_ISL_464038	Unity Health Toronto	Ontario Institute for Cancer Research	2020-04-24	Ramzi Fattouh et al
Canada/ON-UHTC_0068/2020	EPI_ISL_464008	Unity Health Toronto	Ontario Institute for Cancer Research	2020-04-28	Ramzi Fattouh et al
Canada/ON-UHTC_0070/2020	EPI_ISL_464022	Unity Health Toronto	Ontario Institute for Cancer Research	2020-04-12	Ramzi Fattouh et al
Canada/ON-UHTC_0081/2020	EPI_ISL_464064	Unity Health Toronto	Ontario Institute for Cancer Research	2020-04-29	Ramzi Fattouh et al
Canada/ON-UHTC_0089/2020	EPI_ISL_464057	Unity Health Toronto	Ontario Institute for Cancer Research	2020-04-19	Ramzi Fattouh et al
Canada/QC_AB7/2020	EPI_ISL_444504	Laboratoire de microbiologie, Hopital de Verdun	Smith Laboratory, Centre de Recherche CHU Sainte-Justine	2020-04-05	Martin Smith et al
Canada/QC_AC3/2020	EPI_ISL_444507	Laboratoire de microbiologie, Hopital de Verdun	Smith Laboratory, Centre de Recherche CHU Sainte-Justine	2020-04-06	Martin Smith et al
Canada/QC_AC5/2020	EPI_ISL_444508	Laboratoire de microbiologie, Hopital de Verdun	Smith Laboratory, Centre de Recherche CHU Sainte-Justine	2020-04-04	Martin Smith et al
Canada/QC_AE8/2020	EPI_ISL_463907	Laboratoire de microbiologie, Hopital de Verdun	Smith Laboratory, Centre de Recherche CHU Sainte-Justine	2020-04-07	Martin Smith et al
Canada/QC_AF6/2020	EPI_ISL_463912	Laboratoire de microbiologie, Hopital de Verdun	Smith Laboratory, Centre de Recherche CHU Sainte-Justine	2020-04-06	Martin Smith et al
Canada/QC_AR5/2020	EPI_ISL_463932	Laboratoire de microbiologie, Hopital de Verdun	Smith Laboratory, Centre de Recherche CHU Sainte-Justine	2020-04-04	Martin Smith et al
Canada/QC_AR6/2020	EPI_ISL_463933	Laboratoire de microbiologie, Hopital de Verdun	Smith Laboratory, Centre de Recherche CHU Sainte-Justine	2020-04-01	Martin Smith et al
Canada/QC_AR9/2020	EPI_ISL_463934	Laboratoire de microbiologie, Hopital de Verdun	Smith Laboratory, Centre de Recherche CHU Sainte-Justine	2020-04-05	Martin Smith et al
Canada/QC_AS9/2020	EPI_ISL_463939	Laboratoire de microbiologie, Hopital de Verdun	Smith Laboratory, Centre de Recherche CHU Sainte-Justine	2020-04-03	Martin Smith et al
Canada/QC_AV4/2020	EPI_ISL_463949	Laboratoire de microbiologie, Hopital de Verdun	Smith Laboratory, Centre de Recherche CHU Sainte-Justine	2020-04-04	Martin Smith et al

Canada/QC_AV6/2020	EPI_ISL_463950	Laboratoire de microbiologie, Hopital de Verdun	Smith Laboratory, Centre de Recherche CHU Sainte-Justine	2020-04-04	Martin Smith et al
Canada/QC_AV9/2020	EPI_ISL_463951	Laboratoire de microbiologie, Hopital de Verdun	Smith Laboratory, Centre de Recherche CHU Sainte-Justine	2020-04-04	Martin Smith et al
Canada/QC_AY8/2020	EPI_ISL_463956	Laboratoire de microbiologie, Hopital de Verdun	Smith Laboratory, Centre de Recherche CHU Sainte-Justine	2020-04-12	Martin Smith et al
Canada/QC_AZ1/2020	EPI_ISL_463958	Laboratoire de microbiologie, Hopital de Verdun	Smith Laboratory, Centre de Recherche CHU Sainte-Justine	2020-04-12	Martin Smith et al
Canada/QC_AZ9/2020	EPI_ISL_463961	Laboratoire de microbiologie, Hopital de Verdun	Smith Laboratory, Centre de Recherche CHU Sainte-Justine	2020-04-14	Martin Smith et al
Canada/QC_BC3/2020	EPI_ISL_463967	Laboratoire de microbiologie, Hopital de Verdun	Smith Laboratory, Centre de Recherche CHU Sainte-Justine	2020-04-09	Martin Smith et al
Canada/QC-AJ7/2020	EPI_ISL_450639	Laboratoire de microbiologie, Hopital de Verdun	Smith Laboratory, Centre de Recherche CHU Sainte-Justine	2020-04-11	Martin Smith et al
Canada/QC-AK2/2020	EPI_ISL_450641	Laboratoire de microbiologie, Hopital de Verdun	Smith Laboratory, Centre de Recherche CHU Sainte-Justine	2020-04-07	Martin Smith et al
Canada/QC-AK3/2020	EPI_ISL_450642	Laboratoire de microbiologie, Hopital de Verdun	Smith Laboratory, Centre de Recherche CHU Sainte-Justine	2020-04-08	Martin Smith et al
Canada/QC-AL9/2020	EPI_ISL_450652	Laboratoire de microbiologie, Hopital de Verdun	Smith Laboratory, Centre de Recherche CHU Sainte-Justine	2020-04-10	Martin Smith et al
Canada/Qc-L00240866/2020	EPI_ISL_465690	Centre hospitalier Anna-Laberge	Laboratoire de santé publique du Québec	2020-03-27	Sandrine Moreira et al
Canada/Qc-L00241115/2020	EPI_ISL_465698	Hôpital Charles-LeMoyne	Laboratoire de santé publique du Québec	2020-03-27	Sandrine Moreira et al
Canada/Qc-L00241344/2020	EPI_ISL_450317	Hôpital Pierre-Boucher	Laboratoire de santé publique du Québec	2020-03-27	Sandrine Moreira et al
canine/HongKong/20-03695/2020	EPI_ISL_450403		School of Public Health	2020-03-18	Sit et al
Chile/Antofagasta_1/2020	EPI_ISL_445266	CENTRO ONCOLOGICO DEL NORTE	Instituto de Salud Publica de Chile	2020-03-12	Andrés E Castillo et al
Chile/Chillan_4/2020	EPI_ISL_445378	HOSPITAL DE BULNES	Instituto de Salud Publica de Chile	2020-04-04	Andrés E Castillo et al
Chile/Concepcion_2/2020	EPI_ISL_445333	LABORATORIO CLINICA UNIVERSITARIA DE CONCEPCION	Instituto de Salud Publica de Chile	2020-03-12	Andrés E Castillo et al
Chile/Copiapo-00006/2020	EPI_ISL_468754	Laboratorio de Biologia Molecular, Facultad de Medicina, Universidad de Atacama	Center for Mathematical Modeling and Center for Genome Regulation. Santiago, Chile	2020-04-29	Gaete A et al
Chile/Copiapo-00007/2020	EPI_ISL_468755	Laboratorio de Biologia Molecular, Facultad de Medicina, Universidad de Atacama	Center for Mathematical Modeling and Center for Genome Regulation. Santiago, Chile	2020-04-29	Gaete A et al
Chile/Copiapo-00014/2020	EPI_ISL_468756	Laboratorio de Biologia Molecular, Facultad de Medicina, Universidad de Atacama	Center for Mathematical Modeling and Center for Genome Regulation. Santiago, Chile	2020-04-30	Gaete A et al
Chile/Copiapo-00015/2020	EPI_ISL_468757	Laboratorio de Biologia Molecular, Facultad de Medicina, Universidad de Atacama	Center for Mathematical Modeling and Center for Genome Regulation. Santiago, Chile	2020-04-30	Gaete A et al
Chile/Copiapo-00063/2020	EPI_ISL_468758	Laboratorio de Biologia Molecular, Facultad de Medicina, Universidad de Atacama	Center for Mathematical Modeling and Center for Genome Regulation. Santiago, Chile	2020-05-01	Gaete A et al
Chile/Copiapo-00064/2020	EPI_ISL_468759	Laboratorio de Biologia Molecular, Facultad de Medicina, Universidad de Atacama	Center for Mathematical Modeling and Center for Genome Regulation. Santiago, Chile	2020-05-01	Gaete A et al
Chile/Huasco-00013/2020	EPI_ISL_468753	Laboratorio de Biologia Molecular, Facultad de Medicina, Universidad de Atacama	Center for Mathematical Modeling and Center for Genome Regulation. Santiago, Chile	2020-04-29	Gaete A et al
Chile/Independencia-27991/2020	EPI_ISL_459857	Center for Genome Regulation (CRG)	Center for Mathematical Modeling and Center for Genome Regulation. Santiago, Chile	2020-03-15	Gaete A et al
Chile/Punta_Arenas_8/2020	EPI_ISL_445289	HOSPITAL NAVAL PUERTO WILLIAMS	Instituto de Salud Publica de Chile	2020-03-20	Andrés E Castillo et al
Chile/Rancagua_1/2020	EPI_ISL_445273	LABORATORIO TORRE MEDICA LTDA.	Instituto de Salud Publica de Chile	2020-03-16	Andrés E Castillo et al
Chile/Rancagua_6/2020	EPI_ISL_445278	LABORATORIO TORRE MEDICA LTDA.	Instituto de Salud Publica de Chile	2020-03-16	Andrés E Castillo et al
Chile/Rancagua_7/2020	EPI_ISL_445297	CLINICA INTEGRAL S.A.	Instituto de Salud Publica de Chile	2020-03-21	Andrés E Castillo et al
Chile/Santiago_14/2020	EPI_ISL_445257	CLINICA TABANCURA	Instituto de Salud Publica de Chile	2020-03-10	Andrés E Castillo et al
Chile/Santiago_17/2020	EPI_ISL_445260	CLINICA ALEMANA DE SANTIAGO S.A.	Instituto de Salud Publica de Chile	2020-03-11	Andrés E Castillo et al
Chile/Santiago_18/2020	EPI_ISL_445261	INTEGRAMEDICA LAB. CLINICO LTDA.	Instituto de Salud Publica de Chile	2020-03-11	Andrés E Castillo et al
Chile/Santiago_19/2020	EPI_ISL_445262	CLINICA TABANCURA	Instituto de Salud Publica de Chile	2020-03-11	Andrés E Castillo et al
Chile/Santiago_21/2020	EPI_ISL_445264	CLINICA REDSALUD VITACURA.	Instituto de Salud Publica de Chile	2020-03-12	Andrés E Castillo et al
Chile/Santiago_22/2020	EPI_ISL_445265	PONTIFICIA UNIVERSIDAD CATOLICA DE CHILE	Instituto de Salud Publica de Chile	2020-03-11	Andrés E Castillo et al
Chile/Santiago_27/2020	EPI_ISL_445306	CLINICA UC SAN CARLOS DE APOQUINDO	Instituto de Salud Publica de Chile	2020-03-16	Andrés E Castillo et al
Chile/Santiago_42/2020	EPI_ISL_445321	HOSPITAL FELIX BULNES	Instituto de Salud Publica de Chile	2020-03-17	Andrés E Castillo et al
Chile/Santiago_45/2020	EPI_ISL_445324	INTEGRAMEDICA S.A	Instituto de Salud Publica de Chile	2020-03-19	Andrés E Castillo et al
Chile/Santiago_46/2020	EPI_ISL_445325	HOSPITAL DR.SOTERO DEL RIO	Instituto de Salud Publica de Chile	2020-03-21	Andrés E Castillo et al
Chile/Santiago_5/2020	EPI_ISL_445248	CLINICA ALEMANA DE SANTIAGO S.A.	Instituto de Salud Publica de Chile	2020-03-09	Andrés E Castillo et al
Chile/Santiago_52/2020	EPI_ISL_445349	HOSPITAL SAN JUAN DE DIOS	Instituto de Salud Publica de Chile	2020-04-01	Andrés E Castillo et al
Chile/Santiago_54/2020	EPI_ISL_445350	HOSPITAL SAN JUAN DE DIOS	Instituto de Salud Publica de Chile	2020-04-01	Andrés E Castillo et al
Chile/Santiago_56/2020	EPI_ISL_445352	HOSPITAL DEL PROFESOR	Instituto de Salud Publica de Chile	2020-04-01	Andrés E Castillo et al
Chile/Santiago_57/2020	EPI_ISL_445353	HOSPITAL PADRE HURTADO	Instituto de Salud Publica de Chile	2020-04-02	Andrés E Castillo et al
Chile/Santiago_58/2020	EPI_ISL_445354	HOSPITAL DE CARABINEROS	Instituto de Salud Publica de Chile	2020-04-02	Andrés E Castillo et al
Chile/Santiago_59/2020	EPI_ISL_445355	MUTUAL DE SEGURIDAD C.CH.C.	Instituto de Salud Publica de Chile	2020-03-22	Andrés E Castillo et al
Chile/Santiago_69/2020	EPI_ISL_445365	HOSPITAL DR.SOTERO DEL RIO	Instituto de Salud Publica de Chile	2020-03-20	Andrés E Castillo et al
Chile/Santiago_70/2020	EPI_ISL_445366	HOSPITAL DR.SOTERO DEL RIO	Instituto de Salud Publica de Chile	2020-03-21	Andrés E Castillo et al
Chile/Santiago_73/2020	EPI_ISL_445369	HOSPITAL DE CARABINEROS	Instituto de Salud Publica de Chile	2020-04-06	Andrés E Castillo et al
Chile/Santiago_74/2020	EPI_ISL_445370	HOSPITAL DE CARABINEROS	Instituto de Salud Publica de Chile	2020-04-05	Andrés E Castillo et al
Chile/Santiago_76/2020	EPI_ISL_445373	HOSPITAL SAN JUAN DE DIOS	Instituto de Salud Publica de Chile	2020-04-03	Andrés E Castillo et al
Chile/Santiago_77/2020	EPI_ISL_445374	HOSPITAL SAN JUAN DE DIOS	Instituto de Salud Publica de Chile	2020-04-04	Andrés E Castillo et al
Chile/Santiago_78/2020	EPI_ISL_445375	HOSPITAL SAN JUAN DE DIOS	Instituto de Salud Publica de Chile	2020-04-04	Andrés E Castillo et al
Chile/Santiago_79/2020	EPI_ISL_445376	HOSPITAL SAN JUAN DE DIOS	Instituto de Salud Publica de Chile	2020-04-04	Andrés E Castillo et al
Chile/Santiago_8/2020	EPI_ISL_445251	HOSPITAL DE CARABINEROS	Instituto de Salud Publica de Chile	2020-03-10	Andrés E Castillo et al
Chile/Santiago_80/2020	EPI_ISL_445377	HOSPITAL SAN JUAN DE DIOS	Instituto de Salud Publica de Chile	2020-04-05	Andrés E Castillo et al
Chile/Santiago_81/2020	EPI_ISL_445379	IMALAB- HOSPITAL FACH	Instituto de Salud Publica de Chile	2020-04-06	Andrés E Castillo et al
Chile/Santiago_op2d1/2020	EPI_ISL_415658	Laboratory of Molecular Virology, Pontificia Universidad Católica de Chile	MSHS Pathogen Surveillance Program	2020-03-06	Rafael A. Medina et al (https://dx.doi.org/10.1007/s40475-020-00205-2)
Chile/Santiago-04917/2020	EPI_ISL_468749	Facultad de Medicina UC	Center for Mathematical Modeling and Center for Genome Regulation. Santiago, Chile	2020-04-29	Gaete A et al
Chile/Santiago-05015/2020	EPI_ISL_468751	Facultad de Medicina UC	Center for Mathematical Modeling and Center for Genome Regulation. Santiago, Chile	2020-04-29	Gaete A et al
Chile/Santiago-05207/2020	EPI_ISL_468748	Facultad de Medicina UC	Center for Mathematical Modeling and Center for Genome Regulation. Santiago, Chile	2020-04-30	Gaete A et al
Chile/Santiago-05230/2020	EPI_ISL_468747	Facultad de Medicina UC	Center for Mathematical Modeling and Center for Genome Regulation. Santiago, Chile	2020-04-30	Gaete A et al
Chile/Santiago-05426/2020	EPI_ISL_468750	Facultad de Medicina UC	Center for Mathematical Modeling and Center for Genome Regulation. Santiago, Chile	2020-04-30	Gaete A et al
Chile/Santiago-1/2020	EPI_ISL_414579	Clinica Alemana de Santiago, Chile	Instituto de Salud Publica de Chile	2020-03-03	Andrés E. Castillo et al (https://dx.doi.org/10.1002/jmv.25797)
Chile/Santiago-2/2020	EPI_ISL_414580	Clinica Santa Maria, Santiago, Chile	Instituto de Salud Publica de Chile	2020-03-05	Andrés E. Castillo et al (https://dx.doi.org/10.1002/jmv.25797)
Chile/Talca-25343/2020	EPI_ISL_459863	Center for Genome Regulation (CRG)	Center for Mathematical Modeling and Center for Genome Regulation. Santiago, Chile	2020-03-12	Gaete A et al
Chile/Temuco_11/2020	EPI_ISL_445345	HOSPITAL DR.HERNAN HENRIQUEZ ARAVENA	Instituto de Salud Publica de Chile	2020-03-27	Andrés E Castillo et al
Chile/Temuco_4/2020	EPI_ISL_445339	HOSPITAL DR.HERNAN HENRIQUEZ ARAVENA	Instituto de Salud Publica de Chile	2020-03-25	Andrés E Castillo et al
Chile/Valparaiso_3/2020	EPI_ISL_445286	HOSPITAL HANGA ROA	Instituto de Salud Publica de Chile	2020-03-19	Andrés E Castillo et al
Chongqing/YC01/2020	EPI_ISL_408478	Yongchuan District Center for Disease Control and Prevention	Chongqing Municipal Center for Disease Control and Prevention	2020-01-21	Ye Sheng et al
Chongqing/ZX01/2020	EPI_ISL_408479	Zhongxian Center for Disease Control and Prevention	Chongqing Municipal Center for Disease Control and Prevention	2020-01-23	Ye Sheng et al
Colombia/Bogota78390/2020	EPI_ISL_418262	Instituto Nacional de Salud	Instituto Nacional de Salud Universidad Cooperativa de Colombia Instituto Alexander von Humboldt Imperial College-London London School of Hygiene & Tropical Medicine	2020-03-06	Marcela Mercado-Reyes et al (https://dx.doi.org/10.1101/2020.06.02.20120782)
Colombia/GUR-0072/2020	EPI_ISL_447734	Grupo de Investigaciones Microbiológicas-UR (GIMUR), Departamento de Biología, Facultad de Ciencias Naturales, Universidad del Rosario, Bogotá, Colombia	Grupo de Investigaciones Microbiológicas-UR (GIMUR), Departamento de Biología, Facultad de Ciencias Naturales, Universidad del Rosario, Bogotá, Colombia Instituto Nacional de Salud, Bogotá, Colombia Icahn School of Medicine at Mount Sinai, New York, USA	2020-03-26	Juan David Ramírez et al (https://www.medrxiv.org/content/10.1101/2020.06.11.20125799v1)
Colombia/GUR-0135/2020	EPI_ISL_447737	Grupo de Investigaciones Microbiológicas-UR (GIMUR), Departamento de Biología, Facultad de Ciencias Naturales, Universidad del Rosario, Bogotá, Colombia	Grupo de Investigaciones Microbiológicas-UR (GIMUR), Departamento de Biología, Facultad de Ciencias Naturales, Universidad del Rosario, Bogotá, Colombia Instituto Nacional de Salud, Bogotá, Colombia Icahn School of Medicine at Mount Sinai, New York, USA	2020-03-26	Juan David Ramírez et al (https://www.medrxiv.org/content/10.1101/2020.06.11.20125799v1)
Colombia/GUR-0207/2020	EPI_ISL_447739	Grupo de Investigaciones Microbiológicas-UR (GIMUR), Departamento de Biología, Facultad de Ciencias Naturales, Universidad del Rosario, Bogotá, Colombia	Grupo de Investigaciones Microbiológicas-UR (GIMUR), Departamento de Biología, Facultad de Ciencias Naturales, Universidad del Rosario, Bogotá, Colombia Instituto Nacional de Salud, Bogotá, Colombia Icahn School of Medicine at Mount Sinai, New York, USA	2020-03-27	Juan David Ramírez et al (https://www.medrxiv.org/content/10.1101/2020.06.11.20125799v1)
Colombia/GUR-0209/2020	EPI_ISL_447740	Grupo de Investigaciones Microbiológicas-UR (GIMUR), Departamento de Biología, Facultad de Ciencias Naturales, Universidad del Rosario, Bogotá, Colombia	Grupo de Investigaciones Microbiológicas-UR (GIMUR), Departamento de Biología, Facultad de Ciencias Naturales, Universidad del Rosario, Bogotá, Colombia Instituto Nacional de Salud, Bogotá, Colombia Icahn School of Medicine at Mount Sinai, New York, USA	2020-03-28	Juan David Ramírez et al (https://www.medrxiv.org/content/10.1101/2020.06.11.20125799v1)
Colombia/GUR-0212/2020	EPI_ISL_447742	Grupo de Investigaciones Microbiológicas-UR (GIMUR), Departamento de Biología, Facultad de Ciencias Naturales, Universidad del Rosario, Bogotá, Colombia	Grupo de Investigaciones Microbiológicas-UR (GIMUR), Departamento de Biología, Facultad de Ciencias Naturales, Universidad del Rosario, Bogotá, Colombia Instituto Nacional de Salud, Bogotá, Colombia Icahn School of Medicine at Mount Sinai, New York, USA	2020-03-28	Juan David Ramírez et al (https://www.medrxiv.org/content/10.1101/2020.06.11.20125799v1)
Colombia/GUR-0458/2020	EPI_ISL_447745	Grupo de Investigaciones Microbiológicas-UR (GIMUR), Departamento de Biología, Facultad de Ciencias Naturales, Universidad del Rosario, Bogotá, Colombia	Grupo de Investigaciones Microbiológicas-UR (GIMUR), Departamento de Biología, Facultad de Ciencias Naturales, Universidad del Rosario, Bogotá, Colombia Instituto Nacional de Salud, Bogotá, Colombia Icahn School of Medicine at Mount Sinai, New York, USA	2020-03-28	Juan David Ramírez et al (https://www.medrxiv.org/content/10.1101/2020.06.11.20125799v1)
Colombia/GUR-0459/2020	EPI_ISL_447746	Grupo de Investigaciones Microbiológicas-UR (GIMUR), Departamento de Biología, Facultad de Ciencias Naturales, Universidad del Rosario, Bogotá, Colombia	Grupo de Investigaciones Microbiológicas-UR (GIMUR), Departamento de Biología, Facultad de Ciencias Naturales, Universidad del Rosario, Bogotá, Colombia Instituto Nacional de Salud, Bogotá, Colombia Icahn School of Medicine at Mount Sinai, New York, USA	2020-03-28	Juan David Ramírez et al (https://www.medrxiv.org/content/10.1101/2020.06.11.20125799v1)
Colombia/GUR-0609/2020	EPI_ISL_447748	Grupo de Investigaciones Microbiológicas-UR (GIMUR), Departamento de Biología, Facultad de Ciencias Naturales, Universidad del Rosario, Bogotá, Colombia	Grupo de Investigaciones Microbiológicas-UR (GIMUR), Departamento de Biología, Facultad de Ciencias Naturales, Universidad del Rosario, Bogotá, Colombia Instituto Nacional de Salud, Bogotá, Colombia Icahn School of Medicine at Mount Sinai, New York, USA	2020-03-29	Juan David Ramírez et al (https://www.medrxiv.org/content/10.1101/2020.06.11.20125799v1)
Colombia/GUR-1215/2020	EPI_ISL_447754	Grupo de Investigaciones Microbiológicas-UR (GIMUR), Departamento de Biología, Facultad de Ciencias Naturales, Universidad del Rosario, Bogotá, Colombia	Grupo de Investigaciones Microbiológicas-UR (GIMUR), Departamento de Biología, Facultad de Ciencias Naturales, Universidad del Rosario, Bogotá, Colombia Instituto Nacional de Salud, Bogotá, Colombia Icahn School of Medicine at Mount Sinai, New York, USA	2020-03-31	Juan David Ramírez et al (https://www.medrxiv.org/content/10.1101/2020.06.11.20125799v1)
Colombia/GUV-92034/2020	EPI_ISL_447755	Instituto Nacional de Salud, Bogotá, Colombia	Grupo de Investigaciones Microbiológicas-UR (GIMUR), Departamento de Biología, Facultad de Ciencias Naturales, Universidad del Rosario, Bogotá, Colombia Instituto Nacional de Salud, Bogotá, Colombia Icahn School of Medicine at Mount Sinai, New York, USA	2020-03-31	Juan David Ramírez et al (https://www.medrxiv.org/content/10.1101/2020.06.11.20125799v1)
Colombia/GUV-92042/2020	EPI_ISL_447756	Instituto Nacional de Salud, Bogotá, Colombia	Grupo de Investigaciones Microbiológicas-UR (GIMUR), Departamento de Biología, Facultad de Ciencias Naturales, Universidad del Rosario, Bogotá, Colombia Instituto Nacional de Salud, Bogotá, Colombia Icahn School of Medicine at Mount Sinai, New York, USA	2020-03-31	Juan David Ramírez et al (https://www.medrxiv.org/content/10.1101/2020.06.11.20125799v1)
Colombia/GUV-92087/2020	EPI_ISL_447760	Instituto Nacional de Salud, Bogotá, Colombia	Grupo de Investigaciones Microbiológicas-UR (GIMUR), Departamento de Biología, Facultad de Ciencias Naturales, Universidad del Rosario, Bogotá, Colombia Instituto Nacional de Salud, Bogotá, Colombia Icahn School of Medicine at Mount Sinai, New York, USA	2020-03-31	Juan David Ramírez et al (https://www.medrxiv.org/content/10.1101/2020.06.11.20125799v1)
Colombia/GUV-92146/2020	EPI_ISL_447761	Instituto Nacional de Salud, Bogotá, Colombia	Grupo de Investigaciones Microbiológicas-UR (GIMUR), Departamento de Biología, Facultad de Ciencias Naturales, Universidad del Rosario, Bogotá, Colombia Instituto Nacional de Salud, Bogotá, Colombia Icahn School of Medicine at Mount Sinai, New York, USA	2020-03-31	Juan David Ramírez et al (https://www.medrxiv.org/content/10.1101/2020.06.11.20125799v1)
Colombia/GUV-92218/2020	EPI_ISL_447765	Instituto Nacional de Salud, Bogotá, Colombia	Grupo de Investigaciones Microbiológicas-UR (GIMUR), Departamento de Biología, Facultad de Ciencias Naturales, Universidad del Rosario, Bogotá, Colombia Instituto Nacional de Salud, Bogotá, Colombia Icahn School of Medicine at Mount Sinai, New York, USA	2020-03-31	Juan David Ramírez et al (https://www.medrxiv.org/content/10.1101/2020.06.11.20125799v1)

Colombia/GUV-92250/2020	EPI_ISL_447766	Instituto Nacional de Salud, Bogotá, Colombia	Grupo de Investigaciones Microbiológicas-UR (GIMUR), Departamento de Biología, Facultad de Ciencias Naturales, Universidad del Rosario, Bogotá, Colombia Instituto Nacional de Salud, Bogotá, Colombia Icahn School of Medicine at Mount Sinai, New York, USA	2020-04-01	Juan David Ramírez et al (https://www.medrxiv.org/content/10.1101/2020.06.11.20125799v1)
Colombia/GUV-92252/2020	EPI_ISL_447767	Instituto Nacional de Salud, Bogotá, Colombia	Grupo de Investigaciones Microbiológicas-UR (GIMUR), Departamento de Biología, Facultad de Ciencias Naturales, Universidad del Rosario, Bogotá, Colombia Instituto Nacional de Salud, Bogotá, Colombia Icahn School of Medicine at Mount Sinai, New York, USA	2020-04-01	Juan David Ramírez et al (https://www.medrxiv.org/content/10.1101/2020.06.11.20125799v1)
Colombia/GVI-93301/2020	EPI_ISL_447769	Instituto Nacional de Salud, Bogotá, Colombia	Grupo de Investigaciones Microbiológicas-UR (GIMUR), Departamento de Biología, Facultad de Ciencias Naturales, Universidad del Rosario, Bogotá, Colombia Instituto Nacional de Salud, Bogotá, Colombia Icahn School of Medicine at Mount Sinai, New York, USA	2020-04-01	Juan David Ramírez et al (https://www.medrxiv.org/content/10.1101/2020.06.11.20125799v1)
Colombia/GVI-93352/2020	EPI_ISL_447771	Instituto Nacional de Salud, Bogotá, Colombia	Grupo de Investigaciones Microbiológicas-UR (GIMUR), Departamento de Biología, Facultad de Ciencias Naturales, Universidad del Rosario, Bogotá, Colombia Instituto Nacional de Salud, Bogotá, Colombia Icahn School of Medicine at Mount Sinai, New York, USA	2020-04-01	Juan David Ramírez et al (https://www.medrxiv.org/content/10.1101/2020.06.11.20125799v1)
Colombia/GVI-93366/2020	EPI_ISL_447774	Instituto Nacional de Salud, Bogotá, Colombia	Grupo de Investigaciones Microbiológicas-UR (GIMUR), Departamento de Biología, Facultad de Ciencias Naturales, Universidad del Rosario, Bogotá, Colombia Instituto Nacional de Salud, Bogotá, Colombia Icahn School of Medicine at Mount Sinai, New York, USA	2020-04-01	Juan David Ramírez et al (https://www.medrxiv.org/content/10.1101/2020.06.11.20125799v1)
Colombia/GVI-93381/2020	EPI_ISL_447775	Instituto Nacional de Salud, Bogotá, Colombia	Grupo de Investigaciones Microbiológicas-UR (GIMUR), Departamento de Biología, Facultad de Ciencias Naturales, Universidad del Rosario, Bogotá, Colombia Instituto Nacional de Salud, Bogotá, Colombia Icahn School of Medicine at Mount Sinai, New York, USA	2020-04-01	Juan David Ramírez et al (https://www.medrxiv.org/content/10.1101/2020.06.11.20125799v1)
Colombia/GVI-93473/2020	EPI_ISL_447781	Instituto Nacional de Salud, Bogotá, Colombia	Grupo de Investigaciones Microbiológicas-UR (GIMUR), Departamento de Biología, Facultad de Ciencias Naturales, Universidad del Rosario, Bogotá, Colombia Instituto Nacional de Salud, Bogotá, Colombia Icahn School of Medicine at Mount Sinai, New York, USA	2020-04-01	Juan David Ramírez et al (https://www.medrxiv.org/content/10.1101/2020.06.11.20125799v1)
Colombia/GVI-93476/2020	EPI_ISL_447782	Instituto Nacional de Salud, Bogotá, Colombia	Grupo de Investigaciones Microbiológicas-UR (GIMUR), Departamento de Biología, Facultad de Ciencias Naturales, Universidad del Rosario, Bogotá, Colombia Instituto Nacional de Salud, Bogotá, Colombia Icahn School of Medicine at Mount Sinai, New York, USA	2020-04-02	Juan David Ramírez et al (https://www.medrxiv.org/content/10.1101/2020.06.11.20125799v1)
Colombia/GVI-93496/2020	EPI_ISL_447784	Instituto Nacional de Salud, Bogotá, Colombia	Grupo de Investigaciones Microbiológicas-UR (GIMUR), Departamento de Biología, Facultad de Ciencias Naturales, Universidad del Rosario, Bogotá, Colombia Instituto Nacional de Salud, Bogotá, Colombia Icahn School of Medicine at Mount Sinai, New York, USA	2020-04-02	Juan David Ramírez et al (https://www.medrxiv.org/content/10.1101/2020.06.11.20125799v1)
Colombia/GVI-93511/2020	EPI_ISL_447785	Instituto Nacional de Salud, Bogotá, Colombia	Grupo de Investigaciones Microbiológicas-UR (GIMUR), Departamento de Biología, Facultad de Ciencias Naturales, Universidad del Rosario, Bogotá, Colombia Instituto Nacional de Salud, Bogotá, Colombia Icahn School of Medicine at Mount Sinai, New York, USA	2020-04-02	Juan David Ramírez et al (https://www.medrxiv.org/content/10.1101/2020.06.11.20125799v1)
Colombia/GVI-93534/2020	EPI_ISL_447786	Instituto Nacional de Salud, Bogotá, Colombia	Grupo de Investigaciones Microbiológicas-UR (GIMUR), Departamento de Biología, Facultad de Ciencias Naturales, Universidad del Rosario, Bogotá, Colombia Instituto Nacional de Salud, Bogotá, Colombia Icahn School of Medicine at Mount Sinai, New York, USA	2020-04-03	Juan David Ramírez et al (https://www.medrxiv.org/content/10.1101/2020.06.11.20125799v1)
Colombia/GVI-93538/2020	EPI_ISL_447790	Instituto Nacional de Salud, Bogotá, Colombia	Grupo de Investigaciones Microbiológicas-UR (GIMUR), Departamento de Biología, Facultad de Ciencias Naturales, Universidad del Rosario, Bogotá, Colombia Instituto Nacional de Salud, Bogotá, Colombia Icahn School of Medicine at Mount Sinai, New York, USA	2020-04-03	Juan David Ramírez et al (https://www.medrxiv.org/content/10.1101/2020.06.11.20125799v1)
Colombia/GVI-97161/2020	EPI_ISL_447791	Instituto Nacional de Salud, Bogotá, Colombia	Grupo de Investigaciones Microbiológicas-UR (GIMUR), Departamento de Biología, Facultad de Ciencias Naturales, Universidad del Rosario, Bogotá, Colombia Instituto Nacional de Salud, Bogotá, Colombia Icahn School of Medicine at Mount Sinai, New York, USA	2020-04-03	Juan David Ramírez et al (https://www.medrxiv.org/content/10.1101/2020.06.11.20125799v1)
Colombia/GVI-97203/2020	EPI_ISL_447793	Instituto Nacional de Salud, Bogotá, Colombia	Grupo de Investigaciones Microbiológicas-UR (GIMUR), Departamento de Biología, Facultad de Ciencias Naturales, Universidad del Rosario, Bogotá, Colombia Instituto Nacional de Salud, Bogotá, Colombia Icahn School of Medicine at Mount Sinai, New York, USA	2020-04-03	Juan David Ramírez et al (https://www.medrxiv.org/content/10.1101/2020.06.11.20125799v1)
Colombia/GVI-97321/2020	EPI_ISL_447796	Instituto Nacional de Salud, Bogotá, Colombia	Grupo de Investigaciones Microbiológicas-UR (GIMUR), Departamento de Biología, Facultad de Ciencias Naturales, Universidad del Rosario, Bogotá, Colombia Instituto Nacional de Salud, Bogotá, Colombia Icahn School of Medicine at Mount Sinai, New York, USA	2020-04-04	Juan David Ramírez et al (https://www.medrxiv.org/content/10.1101/2020.06.11.20125799v1)
Colombia/GVI-97349/2020	EPI_ISL_447797	Instituto Nacional de Salud, Bogotá, Colombia	Grupo de Investigaciones Microbiológicas-UR (GIMUR), Departamento de Biología, Facultad de Ciencias Naturales, Universidad del Rosario, Bogotá, Colombia Instituto Nacional de Salud, Bogotá, Colombia Icahn School of Medicine at Mount Sinai, New York, USA	2020-04-04	Juan David Ramírez et al (https://www.medrxiv.org/content/10.1101/2020.06.11.20125799v1)
Colombia/GVI-97417/2020	EPI_ISL_447798	Instituto Nacional de Salud, Bogotá, Colombia	Grupo de Investigaciones Microbiológicas-UR (GIMUR), Departamento de Biología, Facultad de Ciencias Naturales, Universidad del Rosario, Bogotá, Colombia Instituto Nacional de Salud, Bogotá, Colombia Icahn School of Medicine at Mount Sinai, New York, USA	2020-04-04	Juan David Ramírez et al (https://www.medrxiv.org/content/10.1101/2020.06.11.20125799v1)
Colombia/GVI-97588/2020	EPI_ISL_447799	Instituto Nacional de Salud, Bogotá, Colombia	Grupo de Investigaciones Microbiológicas-UR (GIMUR), Departamento de Biología, Facultad de Ciencias Naturales, Universidad del Rosario, Bogotá, Colombia Instituto Nacional de Salud, Bogotá, Colombia Icahn School of Medicine at Mount Sinai, New York, USA	2020-04-04	Juan David Ramírez et al (https://www.medrxiv.org/content/10.1101/2020.06.11.20125799v1)
Colombia/GVI-97795/2020	EPI_ISL_447805	Instituto Nacional de Salud, Bogotá, Colombia	Grupo de Investigaciones Microbiológicas-UR (GIMUR), Departamento de Biología, Facultad de Ciencias Naturales, Universidad del Rosario, Bogotá, Colombia Instituto Nacional de Salud, Bogotá, Colombia Icahn School of Medicine at Mount Sinai, New York, USA	2020-04-04	Juan David Ramírez et al (https://www.medrxiv.org/content/10.1101/2020.06.11.20125799v1)
Colombia/GVI-97855/2020	EPI_ISL_447806	Instituto Nacional de Salud, Bogotá, Colombia	Grupo de Investigaciones Microbiológicas-UR (GIMUR), Departamento de Biología, Facultad de Ciencias Naturales, Universidad del Rosario, Bogotá, Colombia Instituto Nacional de Salud, Bogotá, Colombia Icahn School of Medicine at Mount Sinai, New York, USA	2020-04-04	Juan David Ramírez et al (https://www.medrxiv.org/content/10.1101/2020.06.11.20125799v1)
Colombia/GVI-97875/2020	EPI_ISL_447807	Instituto Nacional de Salud, Bogotá, Colombia	Grupo de Investigaciones Microbiológicas-UR (GIMUR), Departamento de Biología, Facultad de Ciencias Naturales, Universidad del Rosario, Bogotá, Colombia Instituto Nacional de Salud, Bogotá, Colombia Icahn School of Medicine at Mount Sinai, New York, USA	2020-04-04	Juan David Ramírez et al (https://www.medrxiv.org/content/10.1101/2020.06.11.20125799v1)
Colombia/GVI-97921/2020	EPI_ISL_447808	Instituto Nacional de Salud, Bogotá, Colombia	Grupo de Investigaciones Microbiológicas-UR (GIMUR), Departamento de Biología, Facultad de Ciencias Naturales, Universidad del Rosario, Bogotá, Colombia Instituto Nacional de Salud, Bogotá, Colombia Icahn School of Medicine at Mount Sinai, New York, USA	2020-04-04	Juan David Ramírez et al (https://www.medrxiv.org/content/10.1101/2020.06.11.20125799v1)
Colombia/GVI-98011/2020	EPI_ISL_447810	Instituto Nacional de Salud, Bogotá, Colombia	Grupo de Investigaciones Microbiológicas-UR (GIMUR), Departamento de Biología, Facultad de Ciencias Naturales, Universidad del Rosario, Bogotá, Colombia Instituto Nacional de Salud, Bogotá, Colombia Icahn School of Medicine at Mount Sinai, New York, USA	2020-04-05	Juan David Ramírez et al (https://www.medrxiv.org/content/10.1101/2020.06.11.20125799v1)
Colombia/GVI-98032/2020	EPI_ISL_447811	Instituto Nacional de Salud, Bogotá, Colombia	Grupo de Investigaciones Microbiológicas-UR (GIMUR), Departamento de Biología, Facultad de Ciencias Naturales, Universidad del Rosario, Bogotá, Colombia Instituto Nacional de Salud, Bogotá, Colombia Icahn School of Medicine at Mount Sinai, New York, USA	2020-04-05	Juan David Ramírez et al (https://www.medrxiv.org/content/10.1101/2020.06.11.20125799v1)
Colombia/GVI-98290/2020	EPI_ISL_447813	Instituto Nacional de Salud, Bogotá, Colombia	Grupo de Investigaciones Microbiológicas-UR (GIMUR), Departamento de Biología, Facultad de Ciencias Naturales, Universidad del Rosario, Bogotá, Colombia Instituto Nacional de Salud, Bogotá, Colombia Icahn School of Medicine at Mount Sinai, New York, USA	2020-04-05	Juan David Ramírez et al (https://www.medrxiv.org/content/10.1101/2020.06.11.20125799v1)
Colombia/GVI97223/2020	EPI_ISL_447814	Instituto Nacional de Salud, Bogotá, Colombia	Grupo de Investigaciones Microbiológicas-UR (GIMUR), Departamento de Biología, Facultad de Ciencias Naturales, Universidad del Rosario, Bogotá, Colombia Instituto Nacional de Salud, Bogotá, Colombia Icahn School of Medicine at Mount Sinai, New York, USA	2020-04-06	Juan David Ramírez et al (https://www.medrxiv.org/content/10.1101/2020.06.11.20125799v1)
Colombia/GVI97447/2020	EPI_ISL_447815	Instituto Nacional de Salud, Bogotá, Colombia	Grupo de Investigaciones Microbiológicas-UR (GIMUR), Departamento de Biología, Facultad de Ciencias Naturales, Universidad del Rosario, Bogotá, Colombia Instituto Nacional de Salud, Bogotá, Colombia Icahn School of Medicine at Mount Sinai, New York, USA	2020-04-06	Juan David Ramírez et al (https://www.medrxiv.org/content/10.1101/2020.06.11.20125799v1)
Colombia/INS-103303/2020	EPI_ISL_456154	Instituto Nacional de Salud - Unidad de Secuenciación y Análisis Genómico	Instituto Nacional de Salud, Universidad Cooperativa de Colombia, Instituto Alexander von Humboldt, Imperial College-London, London School of Hygiene & Tropical Medicine	2020-04-26	Katherine Laiton-Donato et al A (https://dx.doi.org/10.1101/2020.06.02.20120782)
Colombia/INS-79253/2020	EPI_ISL_456117	Instituto Nacional de Salud - Unidad de Secuenciación y Análisis Genómico	Instituto Nacional de Salud, Universidad Cooperativa de Colombia, Instituto Alexander von Humboldt, Imperial College-London, London School of Hygiene & Tropical Medicine	2020-03-10	Katherine Laiton-Donato et al A (https://dx.doi.org/10.1101/2020.06.02.20120782)
Colombia/INS-79504/2020	EPI_ISL_456130	Instituto Nacional de Salud - Unidad de Secuenciación y Análisis Genómico	Instituto Nacional de Salud, Universidad Cooperativa de Colombia, Instituto Alexander von Humboldt, Imperial College-London, London School of Hygiene & Tropical Medicine	2020-03-13	Katherine Laiton-Donato et al A (https://dx.doi.org/10.1101/2020.06.02.20120782)
Colombia/INS-79773/2020	EPI_ISL_456134	Instituto Nacional de Salud - Unidad de Secuenciación y Análisis Genómico	Instituto Nacional de Salud, Universidad Cooperativa de Colombia, Instituto Alexander von Humboldt, Imperial College-London, London School of Hygiene & Tropical Medicine	2020-03-14	Katherine Laiton-Donato et al A (https://dx.doi.org/10.1101/2020.06.02.20120782)
Colombia/INS-79965/2020	EPI_ISL_456150	Instituto Nacional de Salud - Unidad de Secuenciación y Análisis Genómico	Instituto Nacional de Salud, Universidad Cooperativa de Colombia, Instituto Alexander von Humboldt, Imperial College-London, London School of Hygiene & Tropical Medicine	2020-03-15	Katherine Laiton-Donato et al A (https://dx.doi.org/10.1101/2020.06.02.20120782)
Colombia/INS-80191/2020	EPI_ISL_456122	Instituto Nacional de Salud - Unidad de Secuenciación y Análisis Genómico	Instituto Nacional de Salud, Universidad Cooperativa de Colombia, Instituto Alexander von Humboldt, Imperial College-London, London School of Hygiene & Tropical Medicine	2020-03-16	Katherine Laiton-Donato et al B
Colombia/INS-80245/2020	EPI_ISL_456155	Instituto Nacional de Salud - Unidad de Secuenciación y Análisis Genómico	Instituto Nacional de Salud, Universidad Cooperativa de Colombia, Instituto Alexander von Humboldt, Imperial College-London, London School of Hygiene & Tropical Medicine	2020-03-16	Katherine Laiton-Donato et al A (https://dx.doi.org/10.1101/2020.06.02.20120782)
Colombia/INS-80656/2020	EPI_ISL_456126	Instituto Nacional de Salud - Unidad de Secuenciación y Análisis Genómico	Instituto Nacional de Salud, Universidad Cooperativa de Colombia, Instituto Alexander von Humboldt, Imperial College-London, London School of Hygiene & Tropical Medicine	2020-03-18	Katherine Laiton-Donato et al A (https://dx.doi.org/10.1101/2020.06.02.20120782)
Colombia/INS-81035/2020	EPI_ISL_456125	Instituto Nacional de Salud - Unidad de Secuenciación y Análisis Genómico	Instituto Nacional de Salud, Universidad Cooperativa de Colombia, Instituto Alexander von Humboldt, Imperial College-London, London School of Hygiene & Tropical Medicine	2020-03-20	Katherine Laiton-Donato et al A (https://dx.doi.org/10.1101/2020.06.02.20120782)
Colombia/INS-81279/2020	EPI_ISL_456142	Instituto Nacional de Salud - Unidad de Secuenciación y Análisis Genómico	Instituto Nacional de Salud, Universidad Cooperativa de Colombia, Instituto Alexander von Humboldt, Imperial College-London, London School of Hygiene & Tropical Medicine	2020-03-18	Katherine Laiton-Donato et al A (https://dx.doi.org/10.1101/2020.06.02.20120782)
Colombia/INS-82099/2020	EPI_ISL_456129	Instituto Nacional de Salud - Unidad de Secuenciación y Análisis Genómico	Instituto Nacional de Salud, Universidad Cooperativa de Colombia, Instituto Alexander von Humboldt, Imperial College-London, London School of Hygiene & Tropical Medicine	2020-03-19	Katherine Laiton-Donato et al A (https://dx.doi.org/10.1101/2020.06.02.20120782)
Colombia/INS-82457/2020	EPI_ISL_456146	Instituto Nacional de Salud - Unidad de Secuenciación y Análisis Genómico	Instituto Nacional de Salud, Universidad Cooperativa de Colombia, Instituto Alexander von Humboldt, Imperial College-London, London School of Hygiene & Tropical Medicine	2020-03-20	Katherine Laiton-Donato et al A (https://dx.doi.org/10.1101/2020.06.02.20120782)
Colombia/INS-84861/2020	EPI_ISL_456116	Instituto Nacional de Salud - Unidad de Secuenciación y Análisis Genómico	Instituto Nacional de Salud, Universidad Cooperativa de Colombia, Instituto Alexander von Humboldt, Imperial College-London, London School of Hygiene & Tropical Medicine	2020-03-22	Katherine Laiton-Donato et al A (https://dx.doi.org/10.1101/2020.06.02.20120782)
Colombia/INS-94514/2020	EPI_ISL_456156	Instituto Nacional de Salud - Unidad de Secuenciación y Análisis Genómico	Instituto Nacional de Salud, Universidad Cooperativa de Colombia, Instituto Alexander von Humboldt, Imperial College-London, London School of Hygiene & Tropical Medicine	2020-04-06	Katherine Laiton-Donato et al A (https://dx.doi.org/10.1101/2020.06.02.20120782)
CostaRica/01/2020	EPI_ISL_434533	Area de Salud Alajuela Sur	Inciensa, Instituto Costarricense de Investigación y Enseñanza en Nutrición y Salud	2020-03-16	Duarte et al
CostaRica/02/2020	EPI_ISL_434535	Area de Salud Alajuela Sur	Inciensa, Instituto Costarricense de Investigación y Enseñanza en Nutrición y Salud	2020-03-16	Duarte et al
CostaRica/03/2020	EPI_ISL_434536	Hospital San Vicente de Paul	Inciensa, Instituto Costarricense de Investigación y Enseñanza en Nutrición y Salud	2020-03-17	Duarte et al
CostaRica/04/2020	EPI_ISL_434540	EBAIS Concepción Norte	Inciensa, Instituto Costarricense de Investigación y Enseñanza en Nutrición y Salud	2020-03-18	Duarte et al
CostaRica/05/2020	EPI_ISL_434538	COOPESAIN	Inciensa, Instituto Costarricense de Investigación y Enseñanza en Nutrición y Salud	2020-03-20	Duarte et al
CostaRica/06/2020	EPI_ISL_434539	Area de Salud Orotina	Inciensa, Instituto Costarricense de Investigación y Enseñanza en Nutrición y Salud	2020-03-25	Duarte et al
CostaRica/CV-0007/2020	EPI_ISL_458236	Hospital Mexico	Charité Virology-University of Costa Rica	2020-03-06	Andres Moreira-Soto et al
Croatia/1146_Split/2020	EPI_ISL_454581	University Hospital for Infectious Diseases "Dr. Fran Mihaljević", Research Unit	University of Zagreb, Centre for research and knowledge transfer in biotechnology	2020-03-18	Ivan-Christian Kurolt et al
Croatia/1560_Split/2020	EPI_ISL_454583	University Hospital for Infectious Diseases "Dr. Fran Mihaljević", Research Unit	University of Zagreb, Centre for research and knowledge transfer in biotechnology	2020-03-18	Ivan-Christian Kurolt et al
Croatia/17-S7new/2020	EPI_ISL_455566	Institute for Public Health	Laboratory for advanced genomics	2020-03-30	Filip Rokić et al
Croatia/1761_Dubrovnik/2020	EPI_ISL_454588	University Hospital for Infectious Diseases "Dr. Fran Mihaljević", Research Unit	University of Zagreb, Centre for research and knowledge transfer in biotechnology	2020-03-20	Ivan-Christian Kurolt et al
Croatia/297_Varazdin/2020	EPI_ISL_454592	University Hospital for Infectious Diseases "Dr. Fran Mihaljević", Research Unit	University of Zagreb, Centre for research and knowledge transfer in biotechnology	2020-03-05	Ivan-Christian Kurolt et al

Croatia/592_Osijek/2020	EPI_ISL_454578	University Hospital for Infectious Diseases "Dr. Fran Mihaljević", Research Unit	University of Zagreb, Centre for research and knowledge transfer in biotechnology	2020-03-13	Ivan-Christian Kurolt et al
Croatia/7R-S8new/2020	EPI_ISL_455567	Institute for Public Health	Laboratory for advanced genomics	2020-03-29	Filip Rokić et al
Croatia/8U-S17new/2020	EPI_ISL_454605	Institute for Public Health	Laboratory for advanced genomics	2020-03-31	Filip Rokić et al
Croatia/AU-S10new/2020	EPI_ISL_468656	Institute for Public Health	Laboratory for advanced genomics	2020-04-09	Filip Rokić et al
Croatia/I7-S21new/2020	EPI_ISL_454606	Institute for Public Health	Laboratory for advanced genomics	2020-03-30	Filip Rokić et al
Croatia/LG-S2new/2020	EPI_ISL_468591	Institute for Public Health	Laboratory for advanced genomics	2020-04-10	Filip Rokić et al
Croatia/OY-S1new/2020	EPI_ISL_454574	nstitute for Public Health	Laboratory for advanced genomics	2020-04-09	Filip Rokić et al
Croatia/ZG-297-20/2020	EPI_ISL_451934	Research Unit, University Hospital for Infectious Diseases "Dr. Fran Mihaljević"	Cicin Sain lab, Helmholtz Centre for Infection Research	2020-03-05	Zeeshan Chaudhry et al
Cyprus/001/2020	EPI_ISL_463741	Department of Molecular Virology, Cyprus Institute of Neurology and Genetics	Department of Molecular Virology, Cyprus Institute of Neurology and Genetics	2020-03-11	Jan Richter et al
Cyprus/002/2020	EPI_ISL_463742	Department of Molecular Virology, Cyprus Institute of Neurology and Genetics	Department of Molecular Virology, Cyprus Institute of Neurology and Genetics	2020-03-22	Jan Richter et al
Cyprus/003/2020	EPI_ISL_463743	Department of Molecular Virology, Cyprus Institute of Neurology and Genetics	Department of Molecular Virology, Cyprus Institute of Neurology and Genetics	2020-03-25	Jan Richter et al
Cyprus/004/2020	EPI_ISL_463744	Department of Molecular Virology, Cyprus Institute of Neurology and Genetics	Department of Molecular Virology, Cyprus Institute of Neurology and Genetics	2020-03-23	Jan Richter et al
Cyprus/005/2020	EPI_ISL_463745	Department of Molecular Virology, Cyprus Institute of Neurology and Genetics	Department of Molecular Virology, Cyprus Institute of Neurology and Genetics	2020-04-01	Jan Richter et al
Cyprus/006/2020	EPI_ISL_463746	Department of Molecular Virology, Cyprus Institute of Neurology and Genetics	Department of Molecular Virology, Cyprus Institute of Neurology and Genetics	2020-04-11	Jan Richter et al
Cyprus/007/2020	EPI_ISL_463747	Department of Molecular Virology, Cyprus Institute of Neurology and Genetics	Department of Molecular Virology, Cyprus Institute of Neurology and Genetics	2020-04-17	Jan Richter et al
Cyprus/008/2020	EPI_ISL_463748	Department of Molecular Virology, Cyprus Institute of Neurology and Genetics	Department of Molecular Virology, Cyprus Institute of Neurology and Genetics	2020-04-27	Jan Richter et al
CzechRepublic/2122/2020	EPI_ISL_425229	Laboratory of Molecular Genetics, 2nd Faculty of Medicine, Charles University in Prague, Prague, Czech Republic	Laboratory of Molecular Genetics, 2nd Faculty of Medicine, Charles University in Prague, Prague, Czech Republic	2020-03-19	Lenka Kramná et al
CzechRepublic/2282/2020	EPI_ISL_426051	Laboratory of Molecular Genetics, 2nd Faculty of Medicine, Charles University in Prague, Prague, Czech Republic	Laboratory of Molecular Genetics, 2nd Faculty of Medicine, Charles University in Prague, Prague, Czech Republic	2020-03-20	Lenka Kramná et al
CzechRepublic/2308/2020	EPI_ISL_434572	The National Institute of Public Health Center for Epidemiology and Microbiology	The National Institute of Public Health Center for Epidemiology and Microbiology	2020-04-14	Alexander Nagy et al
CzechRepublic/2556/2020	EPI_ISL_425228	Laboratory of Molecular Genetics, 2nd Faculty of Medicine, Charles University in Prague, Prague, Czech Republic	Laboratory of Molecular Genetics, 2nd Faculty of Medicine, Charles University in Prague, Prague, Czech Republic	2020-03-22	Lenka Kramná et al
CzechRepublic/2741/2020	EPI_ISL_422636	The National Institute of Public Health Center for Epidemiology and Microbiology	State Veterinary Institute Prague	2020-03-08	Alexander Nagy et al
CzechRepublic/951/2020	EPI_ISL_414477	The National Institute of Public Health Center for Epidemiology and Microbiology	State Veterinary Institute Prague	2020-03-01	Alexander Nagy et al
CzechRepublic/ChVir1630/2020	EPI_ISL_416742	NRL for Influenza, Centrum Epidemiology and Microbiology of National Institute of Public Health, Czech Republic	Charite Universitaetsmedizin Berlin, Institute of Virology	2020-02-29	Victor M Corman et al A
CzechRepublic/ChVir1912/2020	EPI_ISL_416743	NRL for Influenza, Centrum Epidemiology and Microbiology of National Institute of Public Health, Czech Republic	Charite Universitaetsmedizin Berlin, Institute of Virology	2020-03-05	Victor M Corman et al A
CzechRepublic/IAB_1/2020	EPI_ISL_426883	Motol University Hospital	Institute of Applied Biotechnologies a.s.	2020-03-27	Petr Brož et al
CzechRepublic/IAB_10/2020	EPI_ISL_426887	Motol University Hospital	Institute of Applied Biotechnologies a.s.	2020-03-31	Petr Brož et al
CzechRepublic/IAB_12/2020	EPI_ISL_426888	Motol University Hospital	Institute of Applied Biotechnologies a.s.	2020-03-31	Petr Brož et al
CzechRepublic/IAB_14/2020	EPI_ISL_426889	Motol University Hospital	Institute of Applied Biotechnologies a.s.	2020-04-02	Petr Brož et al
CzechRepublic/IAB_15/2020	EPI_ISL_426890	Motol University Hospital	Institute of Applied Biotechnologies a.s.	2020-03-26	Petr Brož et al
CzechRepublic/IAB_16/2020	EPI_ISL_426891	Motol University Hospital	Institute of Applied Biotechnologies a.s.	2020-03-26	Petr Brož et al
CzechRepublic/IAB_17/2020	EPI_ISL_426892	Motol University Hospital	Institute of Applied Biotechnologies a.s.	2020-03-27	Petr Brož et al
CzechRepublic/IAB_18/2020	EPI_ISL_426893	Motol University Hospital	Institute of Applied Biotechnologies a.s.	2020-03-27	Petr Brož et al
CzechRepublic/IAB_20/2020	EPI_ISL_426894	Motol University Hospital	Institute of Applied Biotechnologies a.s.	2020-03-27	Petr Brož et al
CzechRepublic/IAB_21/2020	EPI_ISL_426895	Motol University Hospital	Institute of Applied Biotechnologies a.s.	2020-03-27	Petr Brož et al
CzechRepublic/IAB_22/2020	EPI_ISL_426896	Motol University Hospital	Institute of Applied Biotechnologies a.s.	2020-03-28	Petr Brož et al
CzechRepublic/IAB_23/2020	EPI_ISL_426897	Motol University Hospital	Institute of Applied Biotechnologies a.s.	2020-03-29	Petr Brož et al
CzechRepublic/IAB_4/2020	EPI_ISL_426884	Motol University Hospital	Institute of Applied Biotechnologies a.s.	2020-03-27	Petr Brož et al
CzechRepublic/IAB_8/2020	EPI_ISL_426885	Motol University Hospital	Institute of Applied Biotechnologies a.s.	2020-03-27	Petr Brož et al
CzechRepublic/IAB_9/2020	EPI_ISL_426886	Motol University Hospital	Institute of Applied Biotechnologies a.s.	2020-03-28	Petr Brož et al
CzechRepublic/NRL_2615/2020	EPI_ISL_437519	The National Institute of Public Health Center for Epidemiology and Microbiology	The National Institute of Public Health Center for Epidemiology and Microbiology	2020-03-16	Alexander Nagy et al
CzechRepublic/Seq3/2020	EPI_ISL_426356	Laboratory of Molecular Genetics, 2nd Faculty of Medicine, Charles University in Prague, Prague, Czech Republic	Laboratory of Molecular Genetics, 2nd Faculty of Medicine, Charles University in Prague, Prague, Czech Republic	2020-03-21	Lenka Kramna et al
CzechRepublic/Seq4/2020	EPI_ISL_426357	Laboratory of Molecular Genetics, 2nd Faculty of Medicine, Charles University in Prague, Prague, Czech Republic	Laboratory of Molecular Genetics, 2nd Faculty of Medicine, Charles University in Prague, Prague, Czech Republic	2020-03-18	Lenka Kramna et al
CzechRepublic/Seq5/2020	EPI_ISL_426358	Laboratory of Molecular Genetics, 2nd Faculty of Medicine, Charles University in Prague, Prague, Czech Republic	Laboratory of Molecular Genetics, 2nd Faculty of Medicine, Charles University in Prague, Prague, Czech Republic	2020-03-19	Lenka Kramna et al
CzechRepublic/Seq6/2020	EPI_ISL_426359	Laboratory of Molecular Genetics, 2nd Faculty of Medicine, Charles University in Prague, Prague, Czech Republic	Laboratory of Molecular Genetics, 2nd Faculty of Medicine, Charles University in Prague, Prague, Czech Republic	2020-03-17	Lenka Kramna et al
CzechRepublic/Seq7/2020	EPI_ISL_426360	Laboratory of Molecular Genetics, 2nd Faculty of Medicine, Charles University in Prague, Prague, Czech Republic	Laboratory of Molecular Genetics, 2nd Faculty of Medicine, Charles University in Prague, Prague, Czech Republic	2020-03-18	Lenka Kramna et al
Denmark/ALAB-HH-116/2020	EPI_ISL_452000	Department of Clinical Microbiology, Copenhagen University Hospital, Hvidovre, Kettegaard Alle 30, 2650 Hvidovre.	Albertsen lab, Department of Chemistry and Bioscience, Aalborg University, Denmark	2020-03-09	Rasmus Kirkegaard et al
Denmark/ALAB-HH-119/2020	EPI_ISL_452001	Department of Clinical Microbiology, Copenhagen University Hospital, Hvidovre, Kettegaard Alle 30, 2650 Hvidovre.	Albertsen lab, Department of Chemistry and Bioscience, Aalborg University, Denmark	2020-03-11	Rasmus Kirkegaard et al
Denmark/ALAB-HH-137/2020	EPI_ISL_452016	Department of Clinical Microbiology, Copenhagen University Hospital, Hvidovre, Kettegaard Alle 30, 2650 Hvidovre.	Albertsen lab, Department of Chemistry and Bioscience, Aalborg University, Denmark	2020-03-11	Rasmus Kirkegaard et al
Denmark/ALAB-HH-163/2020	EPI_ISL_452035	Department of Clinical Microbiology, Copenhagen University Hospital, Hvidovre, Kettegaard Alle 30, 2650 Hvidovre.	Albertsen lab, Department of Chemistry and Bioscience, Aalborg University, Denmark	2020-04-14	Rasmus Kirkegaard et al
Denmark/ALAB-HH-169/2020	EPI_ISL_452039	Department of Clinical Microbiology, Copenhagen University Hospital, Hvidovre, Kettegaard Alle 30, 2650 Hvidovre.	Albertsen lab, Department of Chemistry and Bioscience, Aalborg University, Denmark	2020-04-14	Rasmus Kirkegaard et al
Denmark/ALAB-HH-178/2020	EPI_ISL_452046	Department of Clinical Microbiology, Copenhagen University Hospital, Hvidovre, Kettegaard Alle 30, 2650 Hvidovre.	Albertsen lab, Department of Chemistry and Bioscience, Aalborg University, Denmark	2020-04-14	Rasmus Kirkegaard et al
Denmark/ALAB-HH-186/2020	EPI_ISL_452050	Department of Clinical Microbiology, Copenhagen University Hospital, Hvidovre, Kettegaard Alle 30, 2650 Hvidovre.	Albertsen lab, Department of Chemistry and Bioscience, Aalborg University, Denmark	2020-04-14	Rasmus Kirkegaard et al
Denmark/ALAB-HH-193/2020	EPI_ISL_452056	Department of Clinical Microbiology, Copenhagen University Hospital, Hvidovre, Kettegaard Alle 30, 2650 Hvidovre.	Albertsen lab, Department of Chemistry and Bioscience, Aalborg University, Denmark	2020-04-15	Rasmus Kirkegaard et al
Denmark/ALAB-HH-211/2020	EPI_ISL_452066	Department of Clinical Microbiology, Copenhagen University Hospital, Hvidovre, Kettegaard Alle 30, 2650 Hvidovre.	Albertsen lab, Department of Chemistry and Bioscience, Aalborg University, Denmark	2020-04-27	Rasmus Kirkegaard et al
Denmark/ALAB-HH-213/2020	EPI_ISL_452068	Department of Clinical Microbiology, Copenhagen University Hospital, Hvidovre, Kettegaard Alle 30, 2650 Hvidovre.	Albertsen lab, Department of Chemistry and Bioscience, Aalborg University, Denmark	2020-04-27	Rasmus Kirkegaard et al
Denmark/ALAB-HH-227/2020	EPI_ISL_452075	Department of Clinical Microbiology, Copenhagen University Hospital, Hvidovre, Kettegaard Alle 30, 2650 Hvidovre.	Albertsen lab, Department of Chemistry and Bioscience, Aalborg University, Denmark	2020-04-28	Rasmus Kirkegaard et al
Denmark/ALAB-HH-228/2020	EPI_ISL_452076	Department of Clinical Microbiology, Copenhagen University Hospital, Hvidovre, Kettegaard Alle 30, 2650 Hvidovre.	Albertsen lab, Department of Chemistry and Bioscience, Aalborg University, Denmark	2020-04-28	Rasmus Kirkegaard et al
Denmark/ALAB-HH-235/2020	EPI_ISL_452078	Department of Clinical Microbiology, Copenhagen University Hospital, Hvidovre, Kettegaard Alle 30, 2650 Hvidovre.	Albertsen lab, Department of Chemistry and Bioscience, Aalborg University, Denmark	2020-04-28	Rasmus Kirkegaard et al
Denmark/ALAB-HH-240/2020	EPI_ISL_452080	Department of Clinical Microbiology, Copenhagen University Hospital, Hvidovre, Kettegaard Alle 30, 2650 Hvidovre.	Albertsen lab, Department of Chemistry and Bioscience, Aalborg University, Denmark	2020-04-16	Rasmus Kirkegaard et al
Denmark/ALAB-HH-241/2020	EPI_ISL_452081	Department of Clinical Microbiology, Copenhagen University Hospital, Hvidovre, Kettegaard Alle 30, 2650 Hvidovre.	Albertsen lab, Department of Chemistry and Bioscience, Aalborg University, Denmark	2020-04-27	Rasmus Kirkegaard et al
Denmark/ALAB-HH-247/2020	EPI_ISL_452085	Department of Clinical Microbiology, Copenhagen University Hospital, Hvidovre, Kettegaard Alle 30, 2650 Hvidovre.	Albertsen lab, Department of Chemistry and Bioscience, Aalborg University, Denmark	2020-04-23	Rasmus Kirkegaard et al
Denmark/ALAB-HH-252/2020	EPI_ISL_452089	Department of Clinical Microbiology, Copenhagen University Hospital, Hvidovre, Kettegaard Alle 30, 2650 Hvidovre.	Albertsen lab, Department of Chemistry and Bioscience, Aalborg University, Denmark	2020-04-26	Rasmus Kirkegaard et al
Denmark/ALAB-HH-263/2020	EPI_ISL_452094	Department of Clinical Microbiology, Copenhagen University Hospital, Hvidovre, Kettegaard Alle 30, 2650 Hvidovre.	Albertsen lab, Department of Chemistry and Bioscience, Aalborg University, Denmark	2020-04-14	Rasmus Kirkegaard et al
Denmark/ALAB-HH-265/2020	EPI_ISL_452096	Department of Clinical Microbiology, Copenhagen University Hospital, Hvidovre, Kettegaard Alle 30, 2650 Hvidovre.	Albertsen lab, Department of Chemistry and Bioscience, Aalborg University, Denmark	2020-04-14	Rasmus Kirkegaard et al
Denmark/ALAB-HH-266/2020	EPI_ISL_452097	Department of Clinical Microbiology, Copenhagen University Hospital, Hvidovre, Kettegaard Alle 30, 2650 Hvidovre.	Albertsen lab, Department of Chemistry and Bioscience, Aalborg University, Denmark	2020-05-09	Rasmus Kirkegaard et al
Denmark/ALAB-HH-267/2020	EPI_ISL_452098	Department of Clinical Microbiology, Copenhagen University Hospital, Hvidovre, Kettegaard Alle 30, 2650 Hvidovre.	Albertsen lab, Department of Chemistry and Bioscience, Aalborg University, Denmark	2020-05-09	Rasmus Kirkegaard et al
Denmark/ALAB-HH14/2020	EPI_ISL_429272	Department of Clinical Microbiology, Copenhagen University Hospital, Hvidovre, Kettegaard Alle 30, 2650 Hvidovre.	Albertsen lab, Department of Chemistry and Bioscience, Aalborg University, Denmark	2020-03-10	Rasmus Kirkegaard et al
Denmark/ALAB-HH21/2020	EPI_ISL_429279	Department of Clinical Microbiology, Copenhagen University Hospital, Hvidovre, Kettegaard Alle 30, 2650 Hvidovre.	Albertsen lab, Department of Chemistry and Bioscience, Aalborg University, Denmark	2020-03-10	Rasmus Kirkegaard et al
Denmark/ALAB-SSI-1046/2020	EPI_ISL_437630	Department of Virus and Microbiological Special Diagnostics, Statens Serum Institut, Copenhagen, Denmark, Artillerivej 5, 2300 Copenahgen S	Albertsen lab, Department of Chemistry and Bioscience, Aalborg University, Denmark	2020-04-01	Rasmus Kirkegaard et al
Denmark/ALAB-SSI-1065/2020	EPI_ISL_437631	Department of Virus and Microbiological Special Diagnostics, Statens Serum Institut, Copenhagen, Denmark, Artillerivej 5, 2300 Copenahgen S	Albertsen lab, Department of Chemistry and Bioscience, Aalborg University, Denmark	2020-04-02	Rasmus Kirkegaard et al
Denmark/ALAB-SSI-1096/2020	EPI_ISL_437633	Department of Virus and Microbiological Special Diagnostics, Statens Serum Institut, Copenhagen, Denmark, Artillerivej 5, 2300 Copenahgen S	Albertsen lab, Department of Chemistry and Bioscience, Aalborg University, Denmark	2020-04-01	Rasmus Kirkegaard et al
Denmark/ALAB-SSI-1230/2020	EPI_ISL_437637	Department of Virus and Microbiological Special Diagnostics, Statens Serum Institut, Copenhagen, Denmark, Artillerivej 5, 2300 Copenahgen S	Albertsen lab, Department of Chemistry and Bioscience, Aalborg University, Denmark	2020-04-02	Rasmus Kirkegaard et al
Denmark/ALAB-SSI-1273/2020	EPI_ISL_444842	Department of Virus and Microbiological Special Diagnostics, Statens Serum Institut, Copenhagen, Denmark, Artillerivej 5, 2300 Copenahgen S	Albertsen lab, Department of Chemistry and Bioscience, Aalborg University, Denmark	2020-03-10	Rasmus Kirkegaard et al
Denmark/ALAB-SSI-1277/2020	EPI_ISL_444843	Department of Virus and Microbiological Special Diagnostics, Statens Serum Institut, Copenhagen, Denmark, Artillerivej 5, 2300 Copenahgen S	Albertsen lab, Department of Chemistry and Bioscience, Aalborg University, Denmark	2020-04-02	Rasmus Kirkegaard et al
Denmark/ALAB-SSI-1280/2020	EPI_ISL_437639	Department of Virus and Microbiological Special Diagnostics, Statens Serum Institut, Copenhagen, Denmark, Artillerivej 5, 2300 Copenahgen S	Albertsen lab, Department of Chemistry and Bioscience, Aalborg University, Denmark	2020-04-02	Rasmus Kirkegaard et al
Denmark/ALAB-SSI-1351/2020	EPI_ISL_437646	Department of Virus and Microbiological Special Diagnostics, Statens Serum Institut, Copenhagen, Denmark, Artillerivej 5, 2300 Copenahgen S	Albertsen lab, Department of Chemistry and Bioscience, Aalborg University, Denmark	2020-04-02	Rasmus Kirkegaard et al
Denmark/ALAB-SSI-1382/2020	EPI_ISL_444850	Department of Virus and Microbiological Special Diagnostics, Statens Serum Institut, Copenhagen, Denmark, Artillerivej 5, 2300 Copenahgen S	Albertsen lab, Department of Chemistry and Bioscience, Aalborg University, Denmark	2020-04-03	Rasmus Kirkegaard et al
Denmark/ALAB-SSI-1383/2020	EPI_ISL_444851	Department of Virus and Microbiological Special Diagnostics, Statens Serum Institut, Copenhagen, Denmark, Artillerivej 5, 2300 Copenahgen S	Albertsen lab, Department of Chemistry and Bioscience, Aalborg University, Denmark	2020-04-03	Rasmus Kirkegaard et al
Denmark/ALAB-SSI-1386/2020	EPI_ISL_444852	Department of Virus and Microbiological Special Diagnostics, Statens Serum Institut, Copenhagen, Denmark, Artillerivej 5, 2300 Copenahgen S	Albertsen lab, Department of Chemistry and Bioscience, Aalborg University, Denmark	2020-04-03	Rasmus Kirkegaard et al
Denmark/ALAB-SSI-1387/2020	EPI_ISL_444853	Department of Virus and Microbiological Special Diagnostics, Statens Serum Institut, Copenhagen, Denmark, Artillerivej 5, 2300 Copenahgen S	Albertsen lab, Department of Chemistry and Bioscience, Aalborg University, Denmark	2020-04-03	Rasmus Kirkegaard et al
Denmark/ALAB-SSI-1388/2020	EPI_ISL_444854	Department of Virus and Microbiological Special Diagnostics, Statens Serum Institut, Copenhagen, Denmark, Artillerivej 5, 2300 Copenahgen S	Albertsen lab, Department of Chemistry and Bioscience, Aalborg University, Denmark	2020-04-03	Rasmus Kirkegaard et al
Denmark/ALAB-SSI-1398/2020	EPI_ISL_444858	Department of Virus and Microbiological Special Diagnostics, Statens Serum Institut, Copenhagen, Denmark, Artillerivej 5, 2300 Copenahgen S	Albertsen lab, Department of Chemistry and Bioscience, Aalborg University, Denmark	2020-04-03	Rasmus Kirkegaard et al
Denmark/ALAB-SSI-571/2020	EPI_ISL_436974	Department of Virus and Microbiological Special Diagnostics, Statens Serum Institut, Copenhagen, Denmark, Artillerivej 5, 2300 Copenahgen S	Albertsen lab, Department of Chemistry and Bioscience, Aalborg University, Denmark	2020-03-28	Rasmus Kirkegaard et al
Denmark/ALAB-SSI-579/2020	EPI_ISL_436978	Department of Virus and Microbiological Special Diagnostics, Statens Serum Institut, Copenhagen, Denmark, Artillerivej 5, 2300 Copenahgen S	Albertsen lab, Department of Chemistry and Bioscience, Aalborg University, Denmark	2020-03-23	Rasmus Kirkegaard et al
Denmark/ALAB-SSI-709/2020	EPI_ISL_444868	Department of Virus and Microbiological Special Diagnostics, Statens Serum Institut, Copenhagen, Denmark, Artillerivej 5, 2300 Copenahgen S	Albertsen lab, Department of Chemistry and Bioscience, Aalborg University, Denmark	2020-03-28	Rasmus Kirkegaard et al
Denmark/ALAB-SSI-718/2020	EPI_ISL_444872	Department of Virus and Microbiological Special Diagnostics, Statens Serum Institut, Copenhagen, Denmark, Artillerivej 5, 2300 Copenahgen S	Albertsen lab, Department of Chemistry and Bioscience, Aalborg University, Denmark	2020-03-27	Rasmus Kirkegaard et al
Denmark/ALAB-SSI-719/2020	EPI_ISL_444873	Department of Virus and Microbiological Special Diagnostics, Statens Serum Institut, Copenhagen, Denmark, Artillerivej 5, 2300 Copenahgen S	Albertsen lab, Department of Chemistry and Bioscience, Aalborg University, Denmark	2020-03-28	Rasmus Kirkegaard et al
Denmark/ALAB-SSI-786/2020	EPI_ISL_437670	Department of Virus and Microbiological Special Diagnostics, Statens Serum Institut, Copenhagen, Denmark, Artillerivej 5, 2300 Copenahgen S	Albertsen lab, Department of Chemistry and Bioscience, Aalborg University, Denmark	2020-03-30	Rasmus Kirkegaard et al
Denmark/ALAB-SSI-787/2020	EPI_ISL_444905	Department of Virus and Microbiological Special Diagnostics, Statens Serum Institut, Copenhagen, Denmark, Artillerivej 5, 2300 Copenahgen S	Albertsen lab, Department of Chemistry and Bioscience, Aalborg University, Denmark	2020-03-30	Rasmus Kirkegaard et al
Denmark/ALAB-SSI-854/2020	EPI_ISL_444942	Department of Virus and Microbiological Special Diagnostics, Statens Serum Institut, Copenhagen, Denmark, Artillerivej 5, 2300 Copenahgen S	Albertsen lab, Department of Chemistry and Bioscience, Aalborg University, Denmark	2020-03-29	Rasmus Kirkegaard et al
Denmark/ALAB-SSI-860/2020	EPI_ISL_444945	Department of Virus and Microbiological Special Diagnostics, Statens Serum Institut, Copenhagen, Denmark, Artillerivej 5, 2300 Copenahgen S	Albertsen lab, Department of Chemistry and Bioscience, Aalborg University, Denmark	2020-03-27	Rasmus Kirkegaard et al

Denmark/ALAB-SSI-883/2020	EPI_ISL_444954	Department of Virus and Microbiological Special Diagnostics, Statens Serum Institut, Copenhagen, Denmark, Artillerivej 5, 2300 Copenahgen S	Albertsen lab, Department of Chemistry and Bioscience, Aalborg University, Denmark	2020-03-31	Rasmus Kirkegaard et al
Denmark/ALAB-SSI-991/2020	EPI_ISL_437683	Department of Virus and Microbiological Special Diagnostics, Statens Serum Institut, Copenhagen, Denmark, Artillerivej 5, 2300 Copenahgen S	Albertsen lab, Department of Chemistry and Bioscience, Aalborg University, Denmark	2020-04-01	Rasmus Kirkegaard et al
Denmark/ALAB-SSI184/2020	EPI_ISL_429389	Department of Virus and Microbiological Special Diagnostics, Statens Serum Institut, Copenhagen, Denmark, Artillerivej 5, 2300 Copenahgen S	Albertsen lab, Department of Chemistry and Bioscience, Aalborg University, Denmark	2020-03-09	Rasmus Kirkegaard et al
Denmark/ALAB-SSI189/2020	EPI_ISL_429392	Department of Virus and Microbiological Special Diagnostics, Statens Serum Institut, Copenhagen, Denmark, Artillerivej 5, 2300 Copenahgen S	Albertsen lab, Department of Chemistry and Bioscience, Aalborg University, Denmark	2020-03-09	Rasmus Kirkegaard et al
Denmark/ALAB-SSI190/2020	EPI_ISL_429393	Department of Virus and Microbiological Special Diagnostics, Statens Serum Institut, Copenhagen, Denmark, Artillerivej 5, 2300 Copenahgen S	Albertsen lab, Department of Chemistry and Bioscience, Aalborg University, Denmark	2020-03-09	Rasmus Kirkegaard et al
Denmark/ALAB-SSI219/2020	EPI_ISL_429409	Department of Virus and Microbiological Special Diagnostics, Statens Serum Institut, Copenhagen, Denmark, Artillerivej 5, 2300 Copenahgen S	Albertsen lab, Department of Chemistry and Bioscience, Aalborg University, Denmark	2020-03-10	Rasmus Kirkegaard et al
Denmark/ALAB-SSI239/2020	EPI_ISL_429416	Department of Virus and Microbiological Special Diagnostics, Statens Serum Institut, Copenhagen, Denmark, Artillerivej 5, 2300 Copenahgen S	Albertsen lab, Department of Chemistry and Bioscience, Aalborg University, Denmark	2020-03-10	Rasmus Kirkegaard et al
Denmark/ALAB-SSI352/2020	EPI_ISL_429465	Department of Virus and Microbiological Special Diagnostics, Statens Serum Institut, Copenhagen, Denmark, Artillerivej 5, 2300 Copenahgen S	Albertsen lab, Department of Chemistry and Bioscience, Aalborg University, Denmark	2020-03-23	Rasmus Kirkegaard et al
Denmark/ALAB-SSI376/2020	EPI_ISL_429481	Department of Virus and Microbiological Special Diagnostics, Statens Serum Institut, Copenhagen, Denmark, Artillerivej 5, 2300 Copenahgen S	Albertsen lab, Department of Chemistry and Bioscience, Aalborg University, Denmark	2020-03-22	Rasmus Kirkegaard et al
Denmark/ALAB-SSI466/2020	EPI_ISL_429546	Department of Virus and Microbiological Special Diagnostics, Statens Serum Institut, Copenhagen, Denmark, Artillerivej 5, 2300 Copenahgen S	Albertsen lab, Department of Chemistry and Bioscience, Aalborg University, Denmark	2020-03-25	Rasmus Kirkegaard et al
Denmark/ALAB-SSI481/2020	EPI_ISL_429560	Department of Virus and Microbiological Special Diagnostics, Statens Serum Institut, Copenhagen, Denmark, Artillerivej 5, 2300 Copenahgen S	Albertsen lab, Department of Chemistry and Bioscience, Aalborg University, Denmark	2020-03-25	Rasmus Kirkegaard et al
Denmark/ALAB-SSI502/2020	EPI_ISL_429573	Department of Virus and Microbiological Special Diagnostics, Statens Serum Institut, Copenhagen, Denmark, Artillerivej 5, 2300 Copenahgen S	Albertsen lab, Department of Chemistry and Bioscience, Aalborg University, Denmark	2020-03-23	Rasmus Kirkegaard et al
Denmark/ALAB-SSI522/2020	EPI_ISL_429587	Department of Virus and Microbiological Special Diagnostics, Statens Serum Institut, Copenhagen, Denmark, Artillerivej 5, 2300 Copenahgen S	Albertsen lab, Department of Chemistry and Bioscience, Aalborg University, Denmark	2020-03-26	Rasmus Kirkegaard et al
Denmark/ALAB-SSI592/2020	EPI_ISL_429589	Department of Virus and Microbiological Special Diagnostics, Statens Serum Institut, Copenhagen, Denmark, Artillerivej 5, 2300 Copenahgen S	Albertsen lab, Department of Chemistry and Bioscience, Aalborg University, Denmark	2020-03-28	Rasmus Kirkegaard et al
Denmark/ALAB-SSI595/2020	EPI_ISL_429590	Department of Virus and Microbiological Special Diagnostics, Statens Serum Institut, Copenhagen, Denmark, Artillerivej 5, 2300 Copenahgen S	Albertsen lab, Department of Chemistry and Bioscience, Aalborg University, Denmark	2020-03-28	Rasmus Kirkegaard et al
Denmark/SSI-01/2020	EPI_ISL_416142	Department of Virus and Microbiological Special diagnostics, Statens Serum Institut, Copenhagen, Denmark.	Statens Serum Institute	2020-02-26	Morten Rasmussen et al
Denmark/SSI-02/2020	EPI_ISL_416143	Department of Virus and Microbiological Special diagnostics, Statens Serum Institut, Copenhagen, Denmark.	ViFU	2020-02-28	Morten Rasmussen et al
Denmark/SSI-03/2020	EPI_ISL_416144	Department of Virus and Microbiological Special diagnostics, Statens Serum Institut, Copenhagen, Denmark.	ViFU	2020-03-01	Morten Rasmussen et al
DRC/108/2020	EPI_ISL_417948	Viral Respiratory Lab, National Institute for Biomedical Research (INRB)	Pathogen Sequencing Lab, National Institute for Biomedical Research (INRB)	2020-03-19	Placide Mbala-Kingebeni et al
DRC/1249/2020	EPI_ISL_429259	Viral Respiratory Lab, National Institute for Biomedical Research (INRB)	Pathogen Sequencing Lab, National Institute for Biomedical Research (INRB)	2020-04-06	Placide Mbala-Kingebeni et al
DRC/1319/2020	EPI_ISL_431011	Viral Respiratory Lab, National Institute for Biomedical Research (INRB)	Pathogen Sequencing Lab, National Institute for Biomedical Research (INRB)	2020-04-07	Placide Mbala-Kingebeni et al
DRC/1326/2020	EPI_ISL_435116	Viral Respiratory Lab, National Institute for Biomedical Research (INRB)	Pathogen Sequencing Lab, National Institute for Biomedical Research (INRB)	2020-04-07	Placide Mbala-Kingebeni et al
DRC/1376/2020	EPI_ISL_431012	Viral Respiratory Lab, National Institute for Biomedical Research (INRB)	Pathogen Sequencing Lab, National Institute for Biomedical Research (INRB)	2020-04-07	Placide Mbala-Kingebeni et al
DRC/1377/2020	EPI_ISL_434678	Viral Respiratory Lab, National Institute for Biomedical Research (INRB)	Pathogen Sequencing Lab, National Institute for Biomedical Research (INRB)	2020-04-07	Placide Mbala-Kingebeni et al
DRC/1398/2020	EPI_ISL_435114	Viral Respiratory Lab, National Institute for Biomedical Research (INRB)	Pathogen Sequencing Lab, National Institute for Biomedical Research (INRB)	2020-04-07	Placide Mbala-Kingebeni et al
DRC/1422/2020	EPI_ISL_434680	Viral Respiratory Lab, National Institute for Biomedical Research (INRB)	Pathogen Sequencing Lab, National Institute for Biomedical Research (INRB)	2020-04-08	Placide Mbala-Kingebeni et al
DRC/1423/2020	EPI_ISL_434710	Viral Respiratory Lab, National Institute for Biomedical Research (INRB)	Pathogen Sequencing Lab, National Institute for Biomedical Research (INRB)	2020-04-08	Placide Mbala-Kingebeni et al
DRC/1516/2020	EPI_ISL_435032	Viral Respiratory Lab, National Institute for Biomedical Research (INRB)	Pathogen Sequencing Lab, National Institute for Biomedical Research (INRB)	2020-04-09	Placide Mbala-Kingebeni et al
DRC/1767/2020	EPI_ISL_435157	Viral Respiratory Lab, National Institute for Biomedical Research (INRB)	Pathogen Sequencing Lab, National Institute for Biomedical Research (INRB)	2020-04-10	Placide Mbala-Kingebeni et al
DRC/191/2020	EPI_ISL_417955	Viral Respiratory Lab, National Institute for Biomedical Research (INRB)	Pathogen Sequencing Lab, National Institute for Biomedical Research (INRB)	2020-03-21	Placide Mbala-Kingebeni et al
DRC/2047/2020	EPI_ISL_435161	Viral Respiratory Lab, National Institute for Biomedical Research (INRB)	Pathogen Sequencing Lab, National Institute for Biomedical Research (INRB)	2020-04-12	Placide Mbala-Kingebeni et al
DRC/2121/2020	EPI_ISL_435164	Viral Respiratory Lab, National Institute for Biomedical Research (INRB)	Pathogen Sequencing Lab, National Institute for Biomedical Research (INRB)	2020-04-12	Placide Mbala-Kingebeni et al
DRC/2133/2020	EPI_ISL_435168	Viral Respiratory Lab, National Institute for Biomedical Research (INRB)	Pathogen Sequencing Lab, National Institute for Biomedical Research (INRB)	2020-04-13	Placide Mbala-Kingebeni et al
DRC/214/2020	EPI_ISL_420030	Viral Respiratory Lab, National Institute for Biomedical Research (INRB)	Pathogen Sequencing Lab, National Institute for Biomedical Research (INRB)	2020-03-21	Placide Mbala-Kingebeni et al
DRC/215/2020	EPI_ISL_420031	Viral Respiratory Lab, National Institute for Biomedical Research (INRB)	Pathogen Sequencing Lab, National Institute for Biomedical Research (INRB)	2020-03-21	Placide Mbala-Kingebeni et al
DRC/2169/2020	EPI_ISL_436194	Viral Respiratory Lab, National Institute for Biomedical Research (INRB)	Pathogen Sequencing Lab, National Institute for Biomedical Research (INRB)	2020-04-13	Placide Mbala-Kingebeni et al
DRC/236/2020	EPI_ISL_420032	Viral Respiratory Lab, National Institute for Biomedical Research (INRB)	Pathogen Sequencing Lab, National Institute for Biomedical Research (INRB)	2020-03-22	Placide Mbala-Kingebeni et al
DRC/2369/2020	EPI_ISL_437195	Viral Respiratory Lab, National Institute for Biomedical Research (INRB)	Pathogen Sequencing Lab, National Institute for Biomedical Research (INRB)	2020-04-14	Placide Mbala-Kingebeni et al
DRC/241/2020	EPI_ISL_420033	Viral Respiratory Lab, National Institute for Biomedical Research (INRB)	Pathogen Sequencing Lab, National Institute for Biomedical Research (INRB)	2020-03-22	Placide Mbala-Kingebeni et al
DRC/243/2020	EPI_ISL_420034	Viral Respiratory Lab, National Institute for Biomedical Research (INRB)	Pathogen Sequencing Lab, National Institute for Biomedical Research (INRB)	2020-03-22	Placide Mbala-Kingebeni et al
DRC/248/2020	EPI_ISL_420035	Viral Respiratory Lab, National Institute for Biomedical Research (INRB)	Pathogen Sequencing Lab, National Institute for Biomedical Research (INRB)	2020-03-22	Placide Mbala-Kingebeni et al
DRC/2529/2020	EPI_ISL_437338	Viral Respiratory Lab, National Institute for Biomedical Research (INRB)	Pathogen Sequencing Lab, National Institute for Biomedical Research (INRB)	2020-04-15	Placide Mbala-Kingebeni et al
DRC/253/2020	EPI_ISL_420838	Viral Respiratory Lab, National Institute for Biomedical Research (INRB)	Pathogen Sequencing Lab, National Institute for Biomedical Research (INRB)	2020-03-22	Placide Mbala-Kingebeni et al
DRC/254/2020	EPI_ISL_420839	Viral Respiratory Lab, National Institute for Biomedical Research (INRB)	Pathogen Sequencing Lab, National Institute for Biomedical Research (INRB)	2020-03-22	Placide Mbala-Kingebeni et al
DRC/2580/2020	EPI_ISL_437341	Viral Respiratory Lab, National Institute for Biomedical Research (INRB)	Pathogen Sequencing Lab, National Institute for Biomedical Research (INRB)	2020-04-15	Placide Mbala-Kingebeni et al
DRC/2670/2020	EPI_ISL_437343	Viral Respiratory Lab, National Institute for Biomedical Research (INRB)	Pathogen Sequencing Lab, National Institute for Biomedical Research (INRB)	2020-04-15	Placide Mbala-Kingebeni et al
DRC/2728/2020	EPI_ISL_437345	Viral Respiratory Lab, National Institute for Biomedical Research (INRB)	Pathogen Sequencing Lab, National Institute for Biomedical Research (INRB)	2020-04-16	Placide Mbala-Kingebeni et al
DRC/2819/2020	EPI_ISL_437347	Viral Respiratory Lab, National Institute for Biomedical Research (INRB)	Pathogen Sequencing Lab, National Institute for Biomedical Research (INRB)	2020-04-16	Placide Mbala-Kingebeni et al
DRC/2942/2020	EPI_ISL_437355	Viral Respiratory Lab, National Institute for Biomedical Research (INRB)	Pathogen Sequencing Lab, National Institute for Biomedical Research (INRB)	2020-04-17	Placide Mbala-Kingebeni et al
DRC/299/2020	EPI_ISL_420840	Viral Respiratory Lab, National Institute for Biomedical Research (INRB)	Pathogen Sequencing Lab, National Institute for Biomedical Research (INRB)	2020-03-20	Placide Mbala-Kingebeni et al
DRC/300/2020	EPI_ISL_420841	Viral Respiratory Lab, National Institute for Biomedical Research (INRB)	Pathogen Sequencing Lab, National Institute for Biomedical Research (INRB)	2020-03-22	Placide Mbala-Kingebeni et al
DRC/3451/2020	EPI_ISL_447598	Viral Respiratory Lab, National Institute for Biomedical Research (INRB)	Pathogen Sequencing Lab, National Institute for Biomedical Research (INRB)	2020-04-22	Placide Mbala-Kingebeni et al
DRC/3482/2020	EPI_ISL_447601	Viral Respiratory Lab, National Institute for Biomedical Research (INRB)	Pathogen Sequencing Lab, National Institute for Biomedical Research (INRB)	2020-04-22	Placide Mbala-Kingebeni et al
DRC/3483/2020	EPI_ISL_447602	Viral Respiratory Lab, National Institute for Biomedical Research (INRB)	Pathogen Sequencing Lab, National Institute for Biomedical Research (INRB)	2020-04-22	Placide Mbala-Kingebeni et al
DRC/3595/2020	EPI_ISL_447606	Viral Respiratory Lab, National Institute for Biomedical Research (INRB)	Pathogen Sequencing Lab, National Institute for Biomedical Research (INRB)	2020-04-23	Placide Mbala-Kingebeni et al
DRC/3662/2020	EPI_ISL_447234	Viral Respiratory Lab, National Institute for Biomedical Research (INRB)	Pathogen Sequencing Lab, National Institute for Biomedical Research (INRB)	2020-04-23	Placide Mbala-Kingebeni et al
DRC/3683/2020	EPI_ISL_447236	Viral Respiratory Lab, National Institute for Biomedical Research (INRB)	Pathogen Sequencing Lab, National Institute for Biomedical Research (INRB)	2020-04-24	Placide Mbala-Kingebeni et al
DRC/376/2020	EPI_ISL_420851	Viral Respiratory Lab, National Institute for Biomedical Research (INRB)	Pathogen Sequencing Lab, National Institute for Biomedical Research (INRB)	2020-03-25	Placide Mbala-Kingebeni et al
DRC/3787/2020	EPI_ISL_447241	Viral Respiratory Lab, National Institute for Biomedical Research (INRB)	Pathogen Sequencing Lab, National Institute for Biomedical Research (INRB)	2020-04-24	Placide Mbala-Kingebeni et al
DRC/3806/2020	EPI_ISL_447244	Viral Respiratory Lab, National Institute for Biomedical Research (INRB)	Pathogen Sequencing Lab, National Institute for Biomedical Research (INRB)	2020-04-24	Placide Mbala-Kingebeni et al
DRC/3829/2020	EPI_ISL_447246	Viral Respiratory Lab, National Institute for Biomedical Research (INRB)	Pathogen Sequencing Lab, National Institute for Biomedical Research (INRB)	2020-04-24	Placide Mbala-Kingebeni et al
DRC/396/2020	EPI_ISL_420852	Viral Respiratory Lab, National Institute for Biomedical Research (INRB)	Pathogen Sequencing Lab, National Institute for Biomedical Research (INRB)	2020-03-26	Placide Mbala-Kingebeni et al
DRC/397/2020	EPI_ISL_420845	Viral Respiratory Lab, National Institute for Biomedical Research (INRB)	Pathogen Sequencing Lab, National Institute for Biomedical Research (INRB)	2020-03-26	Placide Mbala-Kingebeni et al
DRC/402/2020	EPI_ISL_420853	Viral Respiratory Lab, National Institute for Biomedical Research (INRB)	Pathogen Sequencing Lab, National Institute for Biomedical Research (INRB)	2020-03-26	Placide Mbala-Kingebeni et al
DRC/521/2020	EPI_ISL_420854	Viral Respiratory Lab, National Institute for Biomedical Research (INRB)	Pathogen Sequencing Lab, National Institute for Biomedical Research (INRB)	2020-03-25	Placide Mbala-Kingebeni et al
DRC/523/2020	EPI_ISL_420849	Viral Respiratory Lab, National Institute for Biomedical Research (INRB)	Pathogen Sequencing Lab, National Institute for Biomedical Research (INRB)	2020-03-28	Placide Mbala-Kingebeni et al
DRC/73/2020	EPI_ISL_417941	Viral Respiratory Lab, National Institute for Biomedical Research (INRB)	Pathogen Sequencing Lab, National Institute for Biomedical Research (INRB)	2020-03-18	Placide Mbala-Kingebeni et al
DRC/80/2020	EPI_ISL_417942	Viral Respiratory Lab, National Institute for Biomedical Research (INRB)	Pathogen Sequencing Lab, National Institute for Biomedical Research (INRB)	2020-03-18	Placide Mbala-Kingebeni et al
DRC/81/2020	EPI_ISL_417944	Viral Respiratory Lab, National Institute for Biomedical Research (INRB)	Pathogen Sequencing Lab, National Institute for Biomedical Research (INRB)	2020-03-18	Placide Mbala-Kingebeni et al
DRC/82/2020	EPI_ISL_417946	Viral Respiratory Lab, National Institute for Biomedical Research (INRB)	Pathogen Sequencing Lab, National Institute for Biomedical Research (INRB)	2020-03-18	Placide Mbala-Kingebeni et al
DRC/94/2020	EPI_ISL_417947	Viral Respiratory Lab, National Institute for Biomedical Research (INRB)	Pathogen Sequencing Lab, National Institute for Biomedical Research (INRB)	2020-03-19	Placide Mbala-Kingebeni et al
DRC/KN-0038/2020	EPI_ISL_417434	Viral Respiratory Lab, National Institute for Biomedical Research (INRB)	Pathogen Sequencing Lab, National Institute for Biomedical Research (INRB)	2020-03-14	Placide Mbala-Kingebeni et al
DRC/KN-0058/2020	EPI_ISL_417438	Viral Respiratory Lab, National Institute for Biomedical Research (INRB)	Pathogen Sequencing Lab, National Institute for Biomedical Research (INRB)	2020-03-17	Placide Mbala-Kingebeni et al
DRC/KN-0060/2020	EPI_ISL_417440	Viral Respiratory Lab, National Institute for Biomedical Research (INRB)	Pathogen Sequencing Lab, National Institute for Biomedical Research (INRB)	2020-03-17	Placide Mbala-Kingebeni et al
DRC/KN-0070/2020	EPI_ISL_417441	Viral Respiratory Lab, National Institute for Biomedical Research (INRB)	Pathogen Sequencing Lab, National Institute for Biomedical Research (INRB)	2020-03-17	Placide Mbala-Kingebeni et al
DRC/KN-0072/2020	EPI_ISL_417442	Viral Respiratory Lab, National Institute for Biomedical Research (INRB)	Pathogen Sequencing Lab, National Institute for Biomedical Research (INRB)	2020-03-18	Placide Mbala-Kingebeni et al
DRC/KN-13/2020	EPI_ISL_414647	Viral Respiratory Lab, National Institute for Biomedical Research (INRB)	Pathogen Sequencing Lab, National Institute for Biomedical Research (INRB)	2020-03-09	Placide Mbala-Kingebeni et al
Ecuador/HEE-01/2020	EPI_ISL_417482	Institute of Microbiology, Universidad San Francisco de Quito	Institute of Microbiology, Universidad San Francisco de Quito	2020-03-09	Sully Márquez et al
Ecuador/HGSQ-USFQ-018/2020	EPI_ISL_422563	Institute of Microbiology Universidad San Francisco de Quito	Institute of Microbiology Universidad San Francisco de Quito	2020-03-30	Belen Prado-Vivar et al (https://dx.doi.org/10.1101/2020.06.11.20128330)
Egypt/CUNCI-HGC002-2/2020	EPI_ISL_469275	Human Genome Center	Human Genome Center	2020-05-02	Zekri et al
Egypt/CUNCI-HGC002/2020	EPI_ISL_468045		Cancer Biology Department	2020-05-02	Zekri et al
Egypt/CUNCI-HGC003/2020	EPI_ISL_468049		Cancer Biology Department	2020-05-02	Zekri et al
Egypt/CUNCI-HGC004/2020	EPI_ISL_468048		Cancer Biology Department	2020-05-02	Zekri et al
Egypt/CUNCI-HGC005/2020	EPI_ISL_468052		Cancer Biology Department	2020-05-02	Zekri et al
Egypt/CUNCI-HGC006/2020	EPI_ISL_468050		Cancer Biology Department	2020-05-02	Zekri et al
Egypt/CUNCI-HGC007-2/2020	EPI_ISL_468054		Cancer Biology Department	2020-05-02	Zekri et al
Egypt/CUNCI-HGC007/2020	EPI_ISL_468046		Cancer Biology Department	2020-05-02	Zekri et al
Egypt/CUNCI-HGC008/2020	EPI_ISL_468044		Cancer Biology Department	2020-05-02	Zekri et al
Egypt/CUNCI-HGC009/2020	EPI_ISL_468053		Cancer Biology Department	2020-05-02	Zekri et al
Egypt/CUNCI-HGC010/2020	EPI_ISL_468055		Cancer Biology Department	2020-05-02	Zekri et al
Egypt/CUNCI-HGC011/2020	EPI_ISL_468059		Cancer Biology Department	2020-05-02	Zekri et al
Egypt/CUNCI-HGC012/2020	EPI_ISL_468051		Cancer Biology Department	2020-05-02	Zekri et al
Egypt/CUNCI-HGC013/2020	EPI_ISL_468047		Cancer Biology Department	2020-05-02	Zekri et al
Egypt/CUNCI-HGC014/2020	EPI_ISL_468060		Cancer Biology Department	2020-05-02	Zekri et al
Egypt/CUNCI-HGC015/2020	EPI_ISL_468056		Cancer Biology Department	2020-05-02	Zekri et al
Egypt/CUNCI-HGC016/2020	EPI_ISL_468058		Cancer Biology Department	2020-05-02	Zekri et al

Egypt/CUNCI-HGC021/2020	EPI_ISL_468061		Cancer Biology Department	2020-05-02	Zekri et al
Egypt/CUNCI-HGC023/2020	EPI_ISL_468057		Cancer Biology Department	2020-05-02	Zekri et al
Egypt/NRC-01/2020	EPI_ISL_430820	Center of Scientific Excellence for Influenza Viruses, National Research Centre (NRC), Egypt.	Center of Scientific Excellence for Influenza Viruses, National Research Centre (NRC), Egypt.	2020-03-18	Mohamed Ahmed Ali et al
Egypt/NRC-03/2020	EPI_ISL_430819	Center of Scientific Excellence for Influenza Viruses,National Research Centre (NRC), Egypt.	Center of Scientific Excellence for Influenza Viruses,National Research Centre (NRC), Egypt.	2020-03-18	Mohamed Ahmed Ali et al
England/20092000704/2020	EPI_ISL_464181	Respiratory Virus Unit, Microbiology Services Colindale, Public Health England	Respiratory Virus Unit, Microbiology Services Colindale, Public Health England	2020-02-24	PHE Covid Sequencing Team et al
England/20092000804/2020	EPI_ISL_464182	Respiratory Virus Unit, Microbiology Services Colindale, Public Health England	Respiratory Virus Unit, Microbiology Services Colindale, Public Health England	2020-02-24	PHE Covid Sequencing Team et al
England/20094061404/2020	EPI_ISL_464185	Respiratory Virus Unit, Microbiology Services Colindale, Public Health England	Respiratory Virus Unit, Microbiology Services Colindale, Public Health England	2020-02-25	PHE Covid Sequencing Team et al
England/20096051104/2020	EPI_ISL_464188	Respiratory Virus Unit, Microbiology Services Colindale, Public Health England	Respiratory Virus Unit, Microbiology Services Colindale, Public Health England	2020-02-26	PHE Covid Sequencing Team et al
England/20098017604/2020	EPI_ISL_464195	Respiratory Virus Unit, Microbiology Services Colindale, Public Health England	Respiratory Virus Unit, Microbiology Services Colindale, Public Health England	2020-02-26	PHE Covid Sequencing Team et al
England/200981386/2020	EPI_ISL_414010	Respiratory Virus Unit, Microbiology Services Colindale, Public Health England	Respiratory Virus Unit, Microbiology Services Colindale, Public Health England	2020-02-26	Monica Galiano et al (https://virological.org/t/preliminary-analysis-of-sars-cov-2-importation-establishment-of-uk-transmission-lineages/507)
England/200990660/2020	EPI_ISL_414523	Respiratory Virus Unit, Microbiology Services Colindale, Public Health England	Respiratory Virus Unit, Microbiology Services Colindale, Public Health England	2020-02-27	Monica Galiano et al (https://virological.org/t/preliminary-analysis-of-sars-cov-2-importation-establishment-of-uk-transmission-lineages/507)
England/20100005304/2020	EPI_ISL_464251	Respiratory Virus Unit, Microbiology Services Colindale, Public Health England	Respiratory Virus Unit, Microbiology Services Colindale, Public Health England	2020-02-28	PHE Covid Sequencing Team et al
England/20100005406/2020	EPI_ISL_415135	Respiratory Virus Unit, Microbiology Services Colindale, Public Health England	Respiratory Virus Unit, Microbiology Services Colindale, Public Health England	2020-02-28	Monica Galiano et al (https://virological.org/t/preliminary-analysis-of-sars-cov-2-importation-establishment-of-uk-transmission-lineages/507)
England/20100120904/2020	EPI_ISL_464259	Respiratory Virus Unit, Microbiology Services Colindale, Public Health England	Respiratory Virus Unit, Microbiology Services Colindale, Public Health England	2020-02-29	PHE Covid Sequencing Team et al
England/20100121007/2020	EPI_ISL_415141	Respiratory Virus Unit, Microbiology Services Colindale, Public Health England	Respiratory Virus Unit, Microbiology Services Colindale, Public Health England	2020-02-29	Monica Galiano et al (https://virological.org/t/preliminary-analysis-of-sars-cov-2-importation-establishment-of-uk-transmission-lineages/507)
England/201040024/2020	EPI_ISL_464304	Respiratory Virus Unit, Microbiology Services Colindale, Public Health England	Respiratory Virus Unit, Microbiology Services Colindale, Public Health England	2020-02-03	PHE Covid Sequencing Team et al
England/201040081/2020	EPI_ISL_414525	Respiratory Virus Unit, Microbiology Services Colindale, Public Health England	Respiratory Virus Unit, Microbiology Services Colindale, Public Health England	2020-03-02	Monica Galiano et al (https://virological.org/t/preliminary-analysis-of-sars-cov-2-importation-establishment-of-uk-transmission-lineages/507)
England/201080026/2020	EPI_ISL_464431	Respiratory Virus Unit, Microbiology Services Colindale, Public Health England	Respiratory Virus Unit, Microbiology Services Colindale, Public Health England	2020-03-06	PHE Covid Sequencing Team et al
England/20108127304/2020	EPI_ISL_464482	Respiratory Virus Unit, Microbiology Services Colindale, Public Health England	Respiratory Virus Unit, Microbiology Services Colindale, Public Health England	2020-03-05	PHE Covid Sequencing Team et al
England/201140058/2020	EPI_ISL_464710	Respiratory Virus Unit, Microbiology Services Colindale, Public Health England	Respiratory Virus Unit, Microbiology Services Colindale, Public Health England	2020-03-10	PHE Covid Sequencing Team et al
England/201161201/2020	EPI_ISL_464820	Respiratory Virus Unit, Microbiology Services Colindale, Public Health England	Respiratory Virus Unit, Microbiology Services Colindale, Public Health England	2020-03-11	PHE Covid Sequencing Team et al
England/20144075204/2020	EPI_ISL_423567	Respiratory Virus Unit, Microbiology Services Colindale, Public Health England	Respiratory Virus Unit, Microbiology Services Colindale, Public Health England	2020-04-01	Monica Galiano et al (https://virological.org/t/preliminary-analysis-of-sars-cov-2-importation-establishment-of-uk-transmission-lineages/507)
England/20166008804/2020	EPI_ISL_466521	Respiratory Virus Unit, Microbiology Services Colindale, Public Health England	Respiratory Virus Unit, Microbiology Services Colindale, Public Health England	2020-04-15	PHE Covid Sequencing Team et al
England/20166064004/2020	EPI_ISL_466563	Respiratory Virus Unit, Microbiology Services Colindale, Public Health England	Respiratory Virus Unit, Microbiology Services Colindale, Public Health England	2020-04-16	PHE Covid Sequencing Team et al
England/20172002604/2020	EPI_ISL_465321	Respiratory Virus Unit, Microbiology Services Colindale, Public Health England	Respiratory Virus Unit, Microbiology Services Colindale, Public Health England	2020-04-19	PHE Covid Sequencing Team et al
England/20189176801/2020	EPI_ISL_465575	Respiratory Virus Unit, Microbiology Services Colindale, Public Health England	Respiratory Virus Unit, Microbiology Services Colindale, Public Health England	2020-05-01	PHE Covid Sequencing Team et al
England/20204115604/2020	EPI_ISL_465653	Respiratory Virus Unit, Microbiology Services Colindale, Public Health England	Respiratory Virus Unit, Microbiology Services Colindale, Public Health England	2020-05-13	PHE Covid Sequencing Team et al
England/BRIS-124C2F/2020	EPI_ISL_443890	PHE South West Regional Laboratory, National Infection Service	Wellcome Sanger Institute for the COVID-19 Genomics UK Consortium	2020-03-25	Stephanie Hutchings et al A (https://virological.org/t/preliminary-analysis-of-sars-cov-2-importation-establishment-of-uk-transmission-lineages/507)
England/BRIS-124D86/2020	EPI_ISL_443977	PHE South West Regional Laboratory, National Infection Service	Wellcome Sanger Institute for the COVID-19 Genomics UK Consortium	2020-03-26	Stephanie Hutchings et al A (https://virological.org/t/preliminary-analysis-of-sars-cov-2-importation-establishment-of-uk-transmission-lineages/507)
England/BRIS-125101/2020	EPI_ISL_443914	PHE South West Regional Laboratory, National Infection Service	Wellcome Sanger Institute for the COVID-19 Genomics UK Consortium	2020-03-26	Stephanie Hutchings et al A (https://virological.org/t/preliminary-analysis-of-sars-cov-2-importation-establishment-of-uk-transmission-lineages/507)
England/BRIS-12C999/2020	EPI_ISL_469501	PHE South West Regional Laboratory, National Infection Service	Wellcome Sanger Institute for the COVID-19 Genomics UK Consortium	2020-05-14	Stephanie Hutchings et al B
England/CAMB-1AFB7A/2020	EPI_ISL_459194	Department of Pathology, University of Cambridge	Wellcome Sanger Institute for the COVID-19 Genomics UK Consortium	2020-05-02	Luke W Meredith et al B
England/CAMB-1B0217/2020	EPI_ISL_459315	Department of Pathology, University of Cambridge	Wellcome Sanger Institute for the COVID-19 Genomics UK Consortium	2020-05-02	Luke W Meredith et al B
England/CAMB-1B0D34/2020	EPI_ISL_452966	Department of Pathology, University of Cambridge	COVID-19 Genomics UK (COG-UK) Consortium	2020-05-11	Luke W Meredith et al A (https://virological.org/t/preliminary-analysis-of-sars-cov-2-importation-establishment-of-uk-transmission-lineages/507)
England/CAMB-1B25B2/2020	EPI_ISL_456696	Department of Pathology, University of Cambridge	COVID-19 Genomics UK (COG-UK) Consortium	2020-05-15	Luke W Meredith et al A (https://virological.org/t/preliminary-analysis-of-sars-cov-2-importation-establishment-of-uk-transmission-lineages/507)
England/CAMB-1B335D/2020	EPI_ISL_461556	Department of Pathology, University of Cambridge	COVID-19 Genomics UK (COG-UK) Consortium	2020-05-25	Luke W Meredith et al B
England/CAMB-7330F/2020	EPI_ISL_440257	Department of Pathology, University of Cambridge	Wellcome Sanger Institute for the COVID-19 Genomics UK Consortium	2020-03-21	Luke W Meredith et al A (https://virological.org/t/preliminary-analysis-of-sars-cov-2-importation-establishment-of-uk-transmission-lineages/507)
England/CAMB-76FEA/2020	EPI_ISL_425448	Department of Pathology, University of Cambridge	COVID-19 Genomics UK (COG-UK) Consortium	2020-03-31	Luke W Meredith et al C (https://dx.doi.org/10.1101/2020.05.08.20095687)
England/CAMB-776F3/2020	EPI_ISL_438543	Department of Pathology, University of Cambridge	Wellcome Sanger Institute for the COVID-19 Genomics UK Consortium	2020-03-30	Luke W Meredith et al A (https://virological.org/t/preliminary-analysis-of-sars-cov-2-importation-establishment-of-uk-transmission-lineages/507)
England/CAMB-7A2A1/2020	EPI_ISL_440609	Department of Pathology, University of Cambridge	Wellcome Sanger Institute for the COVID-19 Genomics UK Consortium	2020-04-03	Luke W Meredith et al A (https://virological.org/t/preliminary-analysis-of-sars-cov-2-importation-establishment-of-uk-transmission-lineages/507)
England/CAMB-7BD09/2020	EPI_ISL_442063	Department of Pathology, University of Cambridge	Wellcome Sanger Institute for the COVID-19 Genomics UK Consortium	2020-04-04	Luke W Meredith et al A (https://virological.org/t/preliminary-analysis-of-sars-cov-2-importation-establishment-of-uk-transmission-lineages/507)
England/CAMB-7C1A2/2020	EPI_ISL_434005	Department of Pathology, University of Cambridge	COVID-19 Genomics UK (COG-UK) Consortium	2020-04-13	Luke W Meredith et al C (https://dx.doi.org/10.1101/2020.05.08.20095687)
England/CAMB-7C4D6/2020	EPI_ISL_442280	Department of Pathology, University of Cambridge	Wellcome Sanger Institute for the COVID-19 Genomics UK Consortium	2020-04-05	Luke W Meredith et al A (https://virological.org/t/preliminary-analysis-of-sars-cov-2-importation-establishment-of-uk-transmission-lineages/507)
England/CAMB-7D0FF/2020	EPI_ISL_441605	Department of Pathology, University of Cambridge	Wellcome Sanger Institute for the COVID-19 Genomics UK Consortium	2020-04-06	Luke W Meredith et al A (https://virological.org/t/preliminary-analysis-of-sars-cov-2-importation-establishment-of-uk-transmission-lineages/507)
England/CAMB-7E1CE/2020	EPI_ISL_434048	Department of Pathology, University of Cambridge	COVID-19 Genomics UK (COG-UK) Consortium	2020-04-17	Luke W Meredith et al C (https://dx.doi.org/10.1101/2020.05.08.20095687)
England/CAMB-7EC81/2020	EPI_ISL_442826	Department of Pathology, University of Cambridge	Wellcome Sanger Institute for the COVID-19 Genomics UK Consortium	2020-04-06	Luke W Meredith et al A (https://virological.org/t/preliminary-analysis-of-sars-cov-2-importation-establishment-of-uk-transmission-lineages/507)
England/CAMB-81BAC/2020	EPI_ISL_442591	Department of Pathology, University of Cambridge	Wellcome Sanger Institute for the COVID-19 Genomics UK Consortium	2020-04-12	Luke W Meredith et al A (https://virological.org/t/preliminary-analysis-of-sars-cov-2-importation-establishment-of-uk-transmission-lineages/507)
England/CAMB-81C6D/2020	EPI_ISL_442551	Department of Pathology, University of Cambridge	Wellcome Sanger Institute for the COVID-19 Genomics UK Consortium	2020-04-12	Luke W Meredith et al A (https://virological.org/t/preliminary-analysis-of-sars-cov-2-importation-establishment-of-uk-transmission-lineages/507)
England/CAMB-83CF2/2020	EPI_ISL_438682	Department of Pathology, University of Cambridge	COVID-19 Genomics UK (COG-UK) Consortium	2020-04-23	Luke W Meredith et al A (https://virological.org/t/preliminary-analysis-of-sars-cov-2-importation-establishment-of-uk-transmission-lineages/507)
England/LIVE-9BF3A/2020	EPI_ISL_449490	Liverpool Clinical Laboratories	COVID-19 Genomics UK (COG-UK) Consortium	2020-03-26	Sam Haldenby et al (https://virological.org/t/preliminary-analysis-of-sars-cov-2-importation-establishment-of-uk-transmission-lineages/507)
England/LIVE-9D7B8/2020	EPI_ISL_453232	Liverpool Clinical Laboratories	COVID-19 Genomics UK (COG-UK) Consortium	2020-04-15	Sam Haldenby et al (https://virological.org/t/preliminary-analysis-of-sars-cov-2-importation-establishment-of-uk-transmission-lineages/507)
England/LIVE-9EADC/2020	EPI_ISL_453364	Liverpool Clinical Laboratories	COVID-19 Genomics UK (COG-UK) Consortium	2020-04-05	Sam Haldenby et al (https://virological.org/t/preliminary-analysis-of-sars-cov-2-importation-establishment-of-uk-transmission-lineages/507)
England/LOND-D4C3E/2020	EPI_ISL_449666	University College London, Great Ormond Street Hospital for Children NHS Foundation Trust, Imperial College Healthcare NHS Trust	COVID-19 Genomics UK (COG-UK) Consortium	2020-04-14	Sergi Castellano et al (https://virological.org/t/preliminary-analysis-of-sars-cov-2-importation-establishment-of-uk-transmission-lineages/507)
England/NORT-28CA2A/2020	EPI_ISL_469328	NU-OMICS DNA Sequencing research facility, Northumbria University	Wellcome Sanger Institute for the COVID-19 Genomics UK Consortium	2020-05-18	Chris Duncan et al
England/NORW-E7DF9/2020	EPI_ISL_449091	Quadram Institute Bioscience	COVID-19 Genomics UK (COG-UK) Consortium	2020-05-06	Dave J. Baker et al (https://virological.org/t/preliminary-analysis-of-sars-cov-2-importation-establishment-of-uk-transmission-lineages/507)
England/NORW-EABB0/2020	EPI_ISL_457359	Quadram Institute Bioscience	COVID-19 Genomics UK (COG-UK) Consortium	2020-05-11	Dave J. Baker et al (https://virological.org/t/preliminary-analysis-of-sars-cov-2-importation-establishment-of-uk-transmission-lineages/507)
England/NORW-EB2AB/2020	EPI_ISL_457415	Quadram Institute Bioscience	COVID-19 Genomics UK (COG-UK) Consortium	2020-05-03	Dave J. Baker et al (https://virological.org/t/preliminary-analysis-of-sars-cov-2-importation-establishment-of-uk-transmission-lineages/507)
England/NORW-EB33F/2020	EPI_ISL_457419	Quadram Institute Bioscience	COVID-19 Genomics UK (COG-UK) Consortium	2020-05-03	Dave J. Baker et al (https://virological.org/t/preliminary-analysis-of-sars-cov-2-importation-establishment-of-uk-transmission-lineages/507)
England/NORW-EB50C/2020	EPI_ISL_457439	Quadram Institute Bioscience	COVID-19 Genomics UK (COG-UK) Consortium	2020-05-01	Dave J. Baker et al (https://virological.org/t/preliminary-analysis-of-sars-cov-2-importation-establishment-of-uk-transmission-lineages/507)
England/NOTT-110D23/2020	EPI_ISL_448410	Queens Medical Centre, Clinical Microbiology Department / DeepSeq Nottingham	COVID-19 Genomics UK (COG-UK) Consortium	2020-04-29	Gemma Clark et al A (https://virological.org/t/preliminary-analysis-of-sars-cov-2-importation-establishment-of-uk-transmission-lineages/507)
England/NOTT-111108/2020	EPI_ISL_453630	Queens Medical Centre, Clinical Microbiology Department / DeepSeq Nottingham	COVID-19 Genomics UK (COG-UK) Consortium	2020-05-02	Gemma Clark et al A (https://virological.org/t/preliminary-analysis-of-sars-cov-2-importation-establishment-of-uk-transmission-lineages/507)
England/NOTT-111399/2020	EPI_ISL_453654	Queens Medical Centre, Clinical Microbiology Department / DeepSeq Nottingham	COVID-19 Genomics UK (COG-UK) Consortium	2020-05-13	Gemma Clark et al A (https://virological.org/t/preliminary-analysis-of-sars-cov-2-importation-establishment-of-uk-transmission-lineages/507)
England/NOTT-1116DC/2020	EPI_ISL_461909	Queens Medical Centre, Clinical Microbiology Department / DeepSeq Nottingham	COVID-19 Genomics UK (COG-UK) Consortium	2020-05-22	Gemma Clark et al B
England/NOTT-111AC1/2020	EPI_ISL_461959	Queens Medical Centre, Clinical Microbiology Department / DeepSeq Nottingham	COVID-19 Genomics UK (COG-UK) Consortium	2020-05-27	Gemma Clark et al B
England/NOTT-111B28/2020	EPI_ISL_461962	Queens Medical Centre, Clinical Microbiology Department / DeepSeq Nottingham	COVID-19 Genomics UK (COG-UK) Consortium	2020-06-01	Gemma Clark et al B
England/NOTT-111B37/2020	EPI_ISL_461963	Queens Medical Centre, Clinical Microbiology Department / DeepSeq Nottingham	COVID-19 Genomics UK (COG-UK) Consortium	2020-06-01	Gemma Clark et al B
England/NOTT-111B46/2020	EPI_ISL_461964	Queens Medical Centre, Clinical Microbiology Department / DeepSeq Nottingham	COVID-19 Genomics UK (COG-UK) Consortium	2020-06-01	Gemma Clark et al B
England/NOTT-111B64/2020	EPI_ISL_461965	Queens Medical Centre, Clinical Microbiology Department / DeepSeq Nottingham	COVID-19 Genomics UK (COG-UK) Consortium	2020-06-02	Gemma Clark et al B
England/NOTT-111B73/2020	EPI_ISL_461966	Queens Medical Centre, Clinical Microbiology Department / DeepSeq Nottingham	COVID-19 Genomics UK (COG-UK) Consortium	2020-06-02	Gemma Clark et al B
England/NOTT-111B82/2020	EPI_ISL_461967	Queens Medical Centre, Clinical Microbiology Department / DeepSeq Nottingham	COVID-19 Genomics UK (COG-UK) Consortium	2020-06-02	Gemma Clark et al B
England/NOTT-111BBF/2020	EPI_ISL_461969	Queens Medical Centre, Clinical Microbiology Department / DeepSeq Nottingham	COVID-19 Genomics UK (COG-UK) Consortium	2020-06-02	Gemma Clark et al B
England/OXON-AC938/2020	EPI_ISL_448496	Oxford Viromics, NDM, University of Oxford; Oxford University Hospitals; Basingstoke and North Hampshire Hospital	COVID-19 Genomics UK (COG-UK) Consortium	2020-04-06	Tanya Golubchik et al (https://virological.org/t/preliminary-analysis-of-sars-cov-2-importation-establishment-of-uk-transmission-lineages/507)
England/OXON-ACB41/2020	EPI_ISL_448510	Oxford Viromics, NDM, University of Oxford; Oxford University Hospitals; Basingstoke and North Hampshire Hospital	COVID-19 Genomics UK (COG-UK) Consortium	2020-04-06	Tanya Golubchik et al (https://virological.org/t/preliminary-analysis-of-sars-cov-2-importation-establishment-of-uk-transmission-lineages/507)
England/PORT-2CFC9E/2020	EPI_ISL_461984	Centre for Enzyme Innovation, University of Portsmouth / Translational Research Laboratory, Portsmouth Hospitals NHS Trust	COVID-19 Genomics UK (COG-UK) Consortium	2020-05-05	Angela Beckett et al
England/PORT-2CFD13/2020	EPI_ISL_461989	Centre for Enzyme Innovation, University of Portsmouth / Translational Research Laboratory, Portsmouth Hospitals NHS Trust	COVID-19 Genomics UK (COG-UK) Consortium	2020-05-08	Angela Beckett et al
England/SHEF-C67C5/2020	EPI_ISL_432826	Virology Department, Sheffield Teaching Hospitals NHS Foundation Trust / Virology Department, Sheffield Teaching Hospitals NHS Foundation Trust	COVID-19 Genomics UK (COG-UK) Consortium	2020-04-06	Thushan de Silva et al (https://virological.org/t/preliminary-analysis-of-sars-cov-2-importation-establishment-of-uk-transmission-lineages/507)
England/SHEF-CBBA6/2020	EPI_ISL_442399	Virology Department, Sheffield Teaching Hospitals NHS Foundation Trust/Department of Infection, Immunity and Cardiovascular Disease, The Medical School, University of Sheffield	COVID-19 Genomics UK (COG-UK) Consortium	2020-03-24	Thushan de Silva et al (https://virological.org/t/preliminary-analysis-of-sars-cov-2-importation-establishment-of-uk-transmission-lineages/507)
England/SHEF-D0124/2020	EPI_ISL_442466	Virology Department, Sheffield Teaching Hospitals NHS Foundation Trust/Department of Infection, Immunity and Cardiovascular Disease, The Medical School, University of Sheffield	COVID-19 Genomics UK (COG-UK) Consortium	2020-03-26	Thushan de Silva et al (https://virological.org/t/preliminary-analysis-of-sars-cov-2-importation-establishment-of-uk-transmission-lineages/507)
env/Beijing/BJ2452/2020	EPI_ISL_430743	Chinese PLA Institute for Disease Control and Prevention	Chinese PLA Institute for Disease Control and Prevention	2020-03-14	Peng Li et al
env/Beijing/IVDC-03-06/2020	EPI_ISL_469256	National Institute for Viral Disease Control and Prevention, China CDC	National Institute for Viral Disease Control and Prevention, China CDC	2020-06-11	Xiang Zhao et al
env/USA/FL-UF11/2020	EPI_ISL_447900	Lednicky Laboratory at Emerging Pathogens Institute	Environmental and Global Health	2020-02-21	Lednicky et al
Finland/1/2020	EPI_ISL_407079	Lapland Central Hospital	Department of Virology, University of Helsinki and Helsinki University Hospital, Helsinki, Finland	2020-01-29	Teemu Smura et al A
Finland/13M26/2020	EPI_ISL_418386	Department of Virology and Immunology, University of Helsinki and Helsinki University Hospital, Huslab Finland	Department of Virology, Faculty of Medicine, University of Helsinki, Helsinki, Finland	2020-03-13	Teemu Smura et al A
Finland/13M27/2020	EPI_ISL_418387	Department of Virology and Immunology, University of Helsinki and Helsinki University Hospital, Huslab Finland	Department of Virology, Faculty of Medicine, University of Helsinki, Helsinki, Finland	2020-03-13	Teemu Smura et al A
Finland/13M29/2020	EPI_ISL_418388	Department of Virology and Immunology, University of Helsinki and Helsinki University Hospital, Huslab Finland	Department of Virology, Faculty of Medicine, University of Helsinki, Helsinki, Finland	2020-03-13	Teemu Smura et al A
Finland/13M3/2020	EPI_ISL_418389	Department of Virology and Immunology, University of Helsinki and Helsinki University Hospital, Huslab Finland	Department of Virology, Faculty of Medicine, University of Helsinki, Helsinki, Finland	2020-03-13	Teemu Smura et al A
Finland/13M33/2020	EPI_ISL_418390	Department of Virology and Immunology, University of Helsinki and Helsinki University Hospital, Huslab Finland	Department of Virology, Faculty of Medicine, University of Helsinki, Helsinki, Finland	2020-03-13	Teemu Smura et al A
Finland/13M57/2020	EPI_ISL_418391	Department of Virology and Immunology, University of Helsinki and Helsinki University Hospital, Huslab Finland	Department of Virology, Faculty of Medicine, University of Helsinki, Helsinki, Finland	2020-03-13	Teemu Smura et al A
Finland/13M58/2020	EPI_ISL_418392	Department of Virology and Immunology, University of Helsinki and Helsinki University Hospital, Huslab Finland	Department of Virology, Faculty of Medicine, University of Helsinki, Helsinki, Finland	2020-03-13	Teemu Smura et al A
Finland/13M64/2020	EPI_ISL_418394	Department of Virology and Immunology, University of Helsinki and Helsinki University Hospital, Huslab Finland	Department of Virology, Faculty of Medicine, University of Helsinki, Helsinki, Finland	2020-03-13	Teemu Smura et al A
Finland/13M65/2020	EPI_ISL_418395	Department of Virology and Immunology, University of Helsinki and Helsinki University Hospital, Huslab Finland	Department of Virology, Faculty of Medicine, University of Helsinki, Helsinki, Finland	2020-03-13	Teemu Smura et al A
Finland/13M69/2020	EPI_ISL_418396	Department of Virology and Immunology, University of Helsinki and Helsinki University Hospital, Huslab Finland	Department of Virology, Faculty of Medicine, University of Helsinki, Helsinki, Finland	2020-03-13	Teemu Smura et al A
Finland/13M77/2020	EPI_ISL_418397	Department of Virology and Immunology, University of Helsinki and Helsinki University Hospital, Huslab Finland	Department of Virology, Faculty of Medicine, University of Helsinki, Helsinki, Finland	2020-03-13	Teemu Smura et al A
Finland/13M83/2020	EPI_ISL_418400	Department of Virology and Immunology, University of Helsinki and Helsinki University Hospital, Huslab Finland	Department of Virology, Faculty of Medicine, University of Helsinki, Helsinki, Finland	2020-03-13	Teemu Smura et al A
Finland/14M12/2020	EPI_ISL_418401	Department of Virology and Immunology, University of Helsinki and Helsinki University Hospital, Huslab Finland	Department of Virology, Faculty of Medicine, University of Helsinki, Helsinki, Finland	2020-03-14	Teemu Smura et al A
Finland/14M14/2020	EPI_ISL_418403	Department of Virology and Immunology, University of Helsinki and Helsinki University Hospital, Huslab Finland	Department of Virology, Faculty of Medicine, University of Helsinki, Helsinki, Finland	2020-03-14	Teemu Smura et al A
Finland/14M16/2020	EPI_ISL_418404	Department of Virology and Immunology, University of Helsinki and Helsinki University Hospital, Huslab Finland	Department of Virology, Faculty of Medicine, University of Helsinki, Helsinki, Finland	2020-03-14	Teemu Smura et al A
Finland/14M26/2020	EPI_ISL_418406	Department of Virology and Immunology, University of Helsinki and Helsinki University Hospital, Huslab Finland	Department of Virology, Faculty of Medicine, University of Helsinki, Helsinki, Finland	2020-03-14	Teemu Smura et al A
Finland/14M3/2020	EPI_ISL_418407	Department of Virology and Immunology, University of Helsinki and Helsinki University Hospital, Huslab Finland	Department of Virology, Faculty of Medicine, University of Helsinki, Helsinki, Finland	2020-03-14	Teemu Smura et al A
Finland/14M74/2020	EPI_ISL_418409	Department of Virology and Immunology, University of Helsinki and Helsinki University Hospital, Huslab Finland	Department of Virology, Faculty of Medicine, University of Helsinki, Helsinki, Finland	2020-03-14	Teemu Smura et al A

Finland/14M77/2020	EPI_ISL_418410	Department of Virology and Immunology, University of Helsinki and Helsinki University Hospital, Huslab Finland	Department of Virology, Faculty of Medicine, University of Helsinki, Helsinki, Finland	2020-03-14	Teemu Smura et al A
Finland/14M82/2020	EPI_ISL_418411	Department of Virology and Immunology, University of Helsinki and Helsinki University Hospital, Huslab Finland	Department of Virology, Faculty of Medicine, University of Helsinki, Helsinki, Finland	2020-03-14	Teemu Smura et al A
Finland/FIN-114/2020	EPI_ISL_414640	Department of Virology and Immunology, University of Helsinki and Helsinki University Hospital, Huslab Finland	Department of Virology, Faculty of Medicine, University of Helsinki, Helsinki, Finland	2020-03-01	Teemu Smura et al A
Finland/FIN-25/2020	EPI_ISL_412971	HUS Diagnostiikkakeskus, Hallinto	Department of Virology Faculty of Medicine, Medicum University of Helsinki	2020-02-25	Teemu Smura et al B (https://dx.doi.org/10.1002/jmv.25773)
Finland/FIN-266/2020	EPI_ISL_414646	Department of Virology and Immunology, University of Helsinki and Helsinki University Hospital, Huslab Finland	Department of Virology, Faculty of Medicine, University of Helsinki, Helsinki, Finland	2020-03-04	Teemu Smura et al A
Finland/FIN-455/2020	EPI_ISL_414642	Department of Virology and Immunology, University of Helsinki and Helsinki University Hospital, Huslab Finland	Department of Virology, Faculty of Medicine, University of Helsinki, Helsinki, Finland	2020-03-08	Teemu Smura et al A
Finland/FIN-508/2020	EPI_ISL_414643	Department of Virology and Immunology, University of Helsinki and Helsinki University Hospital, Huslab Finland	Department of Virology, Faculty of Medicine, University of Helsinki, Helsinki, Finland	2020-03-07	Teemu Smura et al A
Finland/FIN03032020A/2020	EPI_ISL_413602	Department of Virology and Immunology, University of Helsinki and Helsinki University Hospital, Huslab Finland	Department of Virology, Faculty of Medicine, University of Helsinki, Helsinki, Finland	2020-03-03	Teemu Smura et al A
Finland/FIN03032020C/2020	EPI_ISL_413604	Department of Virology and Immunology, University of Helsinki and Helsinki University Hospital, Huslab Finland	Department of Virology, Faculty of Medicine, University of Helsinki, Helsinki, Finland	2020-03-03	Teemu Smura et al A
France/211_80/2020	EPI_ISL_469282	Service de Virologie Hôpital Saint-Louis	Laboratory of Cell Biology of viral infection, Unit INSERM-U944	2020-03-11	Laurent Meertens et al
France/ARA12222/2020	EPI_ISL_419179	Institut des Agents Infectieux (IAI), Hospices Civils de Lyon	CNR Virus des Infections Respiratoires - France SUD	2020-03-22	Antonin Bal et al
France/ARA12270/2020	EPI_ISL_419188	Centre Hospitalier de Macon	CNR Virus des Infections Respiratoires - France SUD	2020-03-22	Antonin Bal et al
France/B1623/2020	EPI_ISL_418219	CHU - Hôpital Cavale Blanche - Labo. de Virologie	National Reference Center for Viruses of Respiratory Infections, Institut Pasteur, Paris	2020-02-26	Mélanie Albert et al B (https://dx.doi.org/10.1101/2020.04.24.059576)
France/B2330/2020	EPI_ISL_416502	CHRU Pontchaillou - Laboratoire de Virologie	National Reference Center for Viruses of Respiratory Infections, Institut Pasteur, Paris	2020-02-26	Mélnie Albert et al A (https://dx.doi.org/10.1101/2020.04.24.059576)
France/B2336/2020	EPI_ISL_416505	CHRU Pontchaillou - Laboratoire de Virologie	National Reference Center for Viruses of Respiratory Infections, Institut Pasteur, Paris	2020-03-02	Mélnie Albert et al A (https://dx.doi.org/10.1101/2020.04.24.059576)
France/B2337/2020	EPI_ISL_416506	CHRU Pontchaillou - Laboratoire de Virologie	National Reference Center for Viruses of Respiratory Infections, Institut Pasteur, Paris	2020-03-03	Mélnie Albert et al A (https://dx.doi.org/10.1101/2020.04.24.059576)
France/B2344/2020	EPI_ISL_416509	CHRU Pontchaillou - Laboratoire de Virologie	National Reference Center for Viruses of Respiratory Infections, Institut Pasteur, Paris	2020-03-06	Mélnie Albert et al A (https://dx.doi.org/10.1101/2020.04.24.059576)
France/B5310/2020	EPI_ISL_443265	CHU - Hôpital Cavale Blanche - Labo. de Virologie	National Reference Center for Viruses of Respiratory Infections, Institut Pasteur, Paris	2020-04-03	Mélanie Albert et al C
France/B5363/2020	EPI_ISL_443273	CHU - Hôpital Cavale Blanche - Labo. de Virologie	National Reference Center for Viruses of Respiratory Infections, Institut Pasteur, Paris	2020-03-24	Mélanie Albert et al C
France/B5434/2020	EPI_ISL_443279	CHU - Hôpital Cavale Blanche - Labo. de Virologie	National Reference Center for Viruses of Respiratory Infections, Institut Pasteur, Paris	2020-04-01	Mélanie Albert et al C
France/B5447/2020	EPI_ISL_443280	CHU - Hôpital Cavale Blanche - Labo. de Virologie	National Reference Center for Viruses of Respiratory Infections, Institut Pasteur, Paris	2020-04-01	Mélanie Albert et al C
France/B5456/2020	EPI_ISL_443281	CHU - Hôpital Cavale Blanche - Labo. de Virologie	National Reference Center for Viruses of Respiratory Infections, Institut Pasteur, Paris	2020-04-03	Mélanie Albert et al C
France/B5685/2020	EPI_ISL_443291	CHRU Pontchaillou - Laboratoire de Virologie	National Reference Center for Viruses of Respiratory Infections, Institut Pasteur, Paris	2020-04-02	Mélanie Albert et al C
France/B5687/2020	EPI_ISL_443292	CHRU Pontchaillou - Laboratoire de Virologie	National Reference Center for Viruses of Respiratory Infections, Institut Pasteur, Paris	2020-04-03	Mélanie Albert et al C
France/B5688/2020	EPI_ISL_443293	CHRU Pontchaillou - Laboratoire de Virologie	National Reference Center for Viruses of Respiratory Infections, Institut Pasteur, Paris	2020-04-03	Mélanie Albert et al C
France/B5689/2020	EPI_ISL_443294	CHRU Pontchaillou - Laboratoire de Virologie	National Reference Center for Viruses of Respiratory Infections, Institut Pasteur, Paris	2020-04-06	Mélanie Albert et al C
France/BFC5011/2020	EPI_ISL_443261	CHU de Dijon - Laboratoire de Virologie	National Reference Center for Viruses of Respiratory Infections, Institut Pasteur, Paris	2020-04-03	Mélanie Albert et al C
France/BFC5012/2020	EPI_ISL_443262	CHU de Dijon - Laboratoire de Virologie	National Reference Center for Viruses of Respiratory Infections, Institut Pasteur, Paris	2020-04-02	Mélanie Albert et al C
France/BFC5017/2020	EPI_ISL_443263	CHU de Dijon - Laboratoire de Virologie	National Reference Center for Viruses of Respiratory Infections, Institut Pasteur, Paris	2020-04-02	Mélanie Albert et al C
France/BFC5019/2020	EPI_ISL_443264	CHU de Dijon - Laboratoire de Virologie	National Reference Center for Viruses of Respiratory Infections, Institut Pasteur, Paris	2020-04-02	Mélanie Albert et al C
France/BFC6053/2020	EPI_ISL_443307	La Villa Papyri	National Reference Center for Viruses of Respiratory Infections, Institut Pasteur, Paris	2020-04-09	Mélanie Albert et al C
France/CIBU-200634/2020	EPI_ISL_430846	General Intensive Care Unit, Raymond Poincaré Hospital (AP-HP), Lab Inflammation & Infection, U1173 University Paris Saclay-UVSQ/INSERM, Garches, France.	Institut Pasteur, Laboratory for Urgent Response to biological Threats	2020-04-01	Annane Djillali et al
France/CIBU200107/2020	EPI_ISL_437689	Laboratory for Urgent Response to Biological Threats	Institut Pasteur CIBU / ERI	2020-02-27	V. Caro et al
France/CIBU200132/2020	EPI_ISL_437690	Laboratory for Urgent Response to Biological Threats	Institut Pasteur CIBU /ERI	2020-02-29	V. Caro et al
France/GE1583/2020	EPI_ISL_414623	Laboratoire de Virologie Institut de Virologie - INSERM U 1109 Hôpitaux Universitaires de Strasbourg	National Reference Center for Viruses of Respiratory Infections, Institut Pasteur, Paris	2020-02-25	Mélnie Albert et al B
France/GE1973/2020	EPI_ISL_414631	Hôpital Robert Debré Laboratoire de Virologie	National Reference Center for Viruses of Respiratory Infections, Institut Pasteur, Paris	2020-03-04	Mélnie Albert et al A (https://dx.doi.org/10.1101/2020.04.24.059576)
France/GE2720/2020	EPI_ISL_420039	L'Air du Temps	National Reference Center for Viruses of Respiratory Infections, Institut Pasteur, Paris	2020-03-12	Mélanie Albert et al B (https://dx.doi.org/10.1101/2020.04.24.059576)
France/GE6058/2020	EPI_ISL_443308	Plaisance	National Reference Center for Viruses of Respiratory Infections, Institut Pasteur, Paris	2020-04-15	Mélanie Albert et al C
France/HF1463/2020	EPI_ISL_429968	Centre Hospitalier Compiègne Laboratoire de Biologie	National Reference Center for Viruses of Respiratory Infections, Institut Pasteur, Paris	2020-02-21	Mélanie Albert et al B (https://dx.doi.org/10.1101/2020.04.24.059576)
France/HF1465/2020	EPI_ISL_418218	Centre Hospitalier Compiègne Laboratoire de Biologie	National Reference Center for Viruses of Respiratory Infections, Institut Pasteur, Paris	2020-02-21	Mélanie Albert et al C
France/HF1645/2020	EPI_ISL_418220	Centre Hospitalier Compiègne Laboratoire de Biologie	National Reference Center for Viruses of Respiratory Infections, Institut Pasteur, Paris	2020-02-28	Mélanie Albert et al B (https://dx.doi.org/10.1101/2020.04.24.059576)
France/HF1684/2020	EPI_ISL_414626		National Reference Center for Viruses of Respiratory Infections, Institut Pasteur, Paris	2020-02-29	Mélnie Albert et al A (https://dx.doi.org/10.1101/2020.04.24.059576)
France/HF1871/2020	EPI_ISL_414630	Centre Hospitalier Compiègne Laboratoire de Biologie	National Reference Center for Viruses of Respiratory Infections, Institut Pasteur, Paris	2020-03-03	Mélnie Albert et al A (https://dx.doi.org/10.1101/2020.04.24.059576)
France/HF2039/2020	EPI_ISL_415649		National Reference Center for Viruses of Respiratory Infections, Institut Pasteur, Paris	2020-03-05	Mélnie Albert et al A (https://dx.doi.org/10.1101/2020.04.24.059576)
France/HF2196/2020	EPI_ISL_416493	CH Jean de Navarre Laboratoire de Biologie	National Reference Center for Viruses of Respiratory Infections, Institut Pasteur, Paris	2020-03-08	Mélnie Albert et al A (https://dx.doi.org/10.1101/2020.04.24.059576)
France/HF2381/2020	EPI_ISL_418226	EHPAD - Résidences les Cèdres	National Reference Center for Viruses of Respiratory Infections, Institut Pasteur, Paris	2020-03-09	Mélanie Albert et al B (https://dx.doi.org/10.1101/2020.04.24.059576)
France/HF3036/2020	EPI_ISL_420053	CH Jean de Navarre Laboratoire de Biologie	National Reference Center for Viruses of Respiratory Infections, Institut Pasteur, Paris	2020-03-19	Mélanie Albert et al B (https://dx.doi.org/10.1101/2020.04.24.059576)
France/HF3141/2020	EPI_ISL_420057	CH Compiègne Laboratoire de Biologie	National Reference Center for Viruses of Respiratory Infections, Institut Pasteur, Paris	2020-03-22	Mélanie Albert et al B (https://dx.doi.org/10.1101/2020.04.24.059576)
France/HF3534/2020	EPI_ISL_428353	CH Compiègne Laboratoire de Biologie	National Reference Center for Viruses of Respiratory Infections, Institut Pasteur, Paris	2020-03-24	Mélanie Albert et al C
France/HF3598/2020	EPI_ISL_428358	CH Jeanne de Navarre Laboratoire de Biologie	National Reference Center for Viruses of Respiratory Infections, Institut Pasteur, Paris	2020-03-24	Mélanie Albert et al C
France/HF3677/2020	EPI_ISL_428359	CH Compiègne Laboratoire de Biologie	National Reference Center for Viruses of Respiratory Infections, Institut Pasteur, Paris	2020-03-23	Mélanie Albert et al C
France/HF4220/2020	EPI_ISL_428366	CH Jeanne de Navarre Laboratoire de Biologie	National Reference Center for Viruses of Respiratory Infections, Institut Pasteur, Paris	2020-03-30	Mélanie Albert et al C
France/IDF0515/2020	EPI_ISL_408430	Department of Infectious and Tropical Diseases, Bichat Claude Bernard Hospital, Paris	National Reference Center for Viruses of Respiratory Infections, Institut Pasteur, Paris	2020-01-29	Mélanie Albert et al B (https://dx.doi.org/10.1101/2020.04.24.059576)
France/IDF0626/2020	EPI_ISL_408431	Sorbonne Université, Inserm et Assistance Publique-Hôpitaux de Paris (Pitié Salpêtrière)	National Reference Center for Viruses of Respiratory Infections, Institut Pasteur, Paris	2020-01-29	Mélanie Albert et al A (https://dx.doi.org/10.2807/1560-7917.ES.2020.25.13.2000305)
France/IDF2251/2020	EPI_ISL_428355	Institut Médico légal- Hop R. Poincaré	National Reference Center for Viruses of Respiratory Infections, Institut Pasteur, Paris	2020-03-11	Mélanie Albert et al C
France/IDF2253/2020	EPI_ISL_428357	Institut Médico légal- Hop R. Poincaré	National Reference Center for Viruses of Respiratory Infections, Institut Pasteur, Paris	2020-03-11	Mélanie Albert et al C
France/IDF2278/2020	EPI_ISL_416499	LABM GH nord Essonne	National Reference Center for Viruses of Respiratory Infections, Institut Pasteur, Paris	2020-03-11	Mélnie Albert et al A (https://dx.doi.org/10.1101/2020.04.24.059576)
France/IDF2532/2020	EPI_ISL_418232	Service des Urgences	National Reference Center for Viruses of Respiratory Infections, Institut Pasteur, Paris	2020-03-15	Mélanie Albert et al B (https://dx.doi.org/10.1101/2020.04.24.059576)
France/IDF3218/2020	EPI_ISL_420062	Service de Biologie Médicale - BP 125	National Reference Center for Viruses of Respiratory Infections, Institut Pasteur, Paris	2020-03-22	Mélanie Albert et al B (https://dx.doi.org/10.1101/2020.04.24.059576)
France/IDF3509/2020	EPI_ISL_428351	GH Nord Essonne Service de Biologie clinique	National Reference Center for Viruses of Respiratory Infections, Institut Pasteur, Paris	2020-03-24	Mélanie Albert et al C
France/IDF3703/2020	EPI_ISL_428361	LABM GH nord Essonne de Longjumeau - BP 125	National Reference Center for Viruses of Respiratory Infections, Institut Pasteur, Paris	2020-03-25	Mélanie Albert et al C
France/IDF4432/2020	EPI_ISL_443317	Cabinet Médical	National Reference Center for Viruses of Respiratory Infections, Institut Pasteur, Paris	2020-03-23	Mélanie Albert et al C
France/IDF4793/2020	EPI_ISL_443259	Résidence Ornano	National Reference Center for Viruses of Respiratory Infections, Institut Pasteur, Paris	2020-04-02	Mélanie Albert et al C
France/IDF4843/2020	EPI_ISL_443260	LABM GH nord Essonne de Longjumeau - BP 125	National Reference Center for Viruses of Respiratory Infections, Institut Pasteur, Paris	2020-04-02	Mélanie Albert et al C
France/IDF5655/2020	EPI_ISL_443285	Laboratoire de Microbiologie - Bât A - CH René Dubois	National Reference Center for Viruses of Respiratory Infections, Institut Pasteur, Paris	2020-04-02	Mélanie Albert et al C
France/IDF5778/2020	EPI_ISL_443304	Résidence Esterel	National Reference Center for Viruses of Respiratory Infections, Institut Pasteur, Paris	2020-04-01	Mélanie Albert et al C
France/IDF5986/2020	EPI_ISL_443305	LABM GH nord Essonne de Longjumeau - BP 125	National Reference Center for Viruses of Respiratory Infections, Institut Pasteur, Paris	2020-04-13	Mélanie Albert et al C
France/IDF6037/2020	EPI_ISL_443306	Cabinet Médical	National Reference Center for Viruses of Respiratory Infections, Institut Pasteur, Paris	2020-04-02	Mélanie Albert et al C
France/IDF6073/2020	EPI_ISL_443314	LABM GH nord Essonne de Longjumeau - BP 125	National Reference Center for Viruses of Respiratory Infections, Institut Pasteur, Paris	2020-04-14	Mélanie Albert et al C
France/Lyon_0668/2020	EPI_ISL_417336	Institut des Agents Infectieux (IAI), Hospices Civils de Lyon	CNR Virus des Infections Respiratoires - France SUD	2020-03-06	Antonin Bal et al
France/N1620/2020	EPI_ISL_416624	Centre Hositalier Universitaire de Rouen Laboratoire de Virologie	National Reference Center for Viruses of Respiratory Infections, Institut Pasteur, Paris	2020-02-26	Mélnie Albert et al A (https://dx.doi.org/10.1101/2020.04.24.059576)
France/N2223/2020	EPI_ISL_416494	Centre Hositalier Universitaire de Rouen Laboratoire de Virologie	National Reference Center for Viruses of Respiratory Infections, Institut Pasteur, Paris	2020-03-04	Mélnie Albert et al A (https://dx.doi.org/10.1101/2020.04.24.059576)
France/OCC-13/2020	EPI_ISL_434628	CHU Purpan - Laboratoire de Virologie - Institut Fédératif de Biologie	Laboratoire de virologie - École Nationale Vétérinaire de Toulouse	2020-04-07	Guillaume Croville et al
France/OCC-14/2020	EPI_ISL_434629	CHU Purpan - Laboratoire de Virologie - Institut Fédératif de Biologie	Laboratoire de virologie - École Nationale Vétérinaire de Toulouse	2020-04-07	Guillaume Croville et al
France/OCC-15/2020	EPI_ISL_434630	CHU Purpan - Laboratoire de Virologie - Institut Fédératif de Biologie	Laboratoire de virologie - École Nationale Vétérinaire de Toulouse	2020-04-08	Guillaume Croville et al
France/OCC-16/2020	EPI_ISL_434631	CHU Purpan - Laboratoire de Virologie - Institut Fédératif de Biologie	Laboratoire de virologie - École Nationale Vétérinaire de Toulouse	2020-04-08	Guillaume Croville et al
France/OCC-17/2020	EPI_ISL_434632	CHU Purpan - Laboratoire de Virologie - Institut Fédératif de Biologie	Laboratoire de virologie - École Nationale Vétérinaire de Toulouse	2020-04-09	Guillaume Croville et al
France/OCC-18/2020	EPI_ISL_434633	CHU Purpan - Laboratoire de Virologie - Institut Fédératif de Biologie	Laboratoire de virologie - École Nationale Vétérinaire de Toulouse	2020-04-09	Guillaume Croville et al
France/OCC-19/2020	EPI_ISL_434634	CHU Purpan - Laboratoire de Virologie - Institut Fédératif de Biologie	Laboratoire de virologie - École Nationale Vétérinaire de Toulouse	2020-04-11	Guillaume Croville et al
France/OCC-20/2020	EPI_ISL_434635	CHU Purpan - Laboratoire de Virologie - Institut Fédératif de Biologie	Laboratoire de virologie - École Nationale Vétérinaire de Toulouse	2020-04-11	Guillaume Croville et al
France/PL1643/2020	EPI_ISL_414625	Centre Hospitalier Régional Universitaire de Nantes Laboratoire de Virologie	National Reference Center for Viruses of Respiratory Infections, Institut Pasteur, Paris	2020-02-26	Mélnie Albert et al A (https://dx.doi.org/10.1101/2020.04.24.059576)
France/RA739/2020	EPI_ISL_410486	CNR Virus des Infections Respiratoires - France SUD	CNR Virus des Infections Respiratoires - France SUD	2020-02-08	Bal et al (https://dx.doi.org/10.1016/j.cmi.2020.03.020)
Fujian/IM3520001T/2020	EPI_ISL_431118	Fujian Center for Disease Control and Prevention	Fujian Center for Disease Control and Prevention	2020-03-13	Lin Qi et al
Fujian/IM3520010T/2020	EPI_ISL_431780	Fujian Center for Disease Control and Prevention	Fujian Center for Disease Control and Prevention	2020-03-17	Lin Qi et al
Fujian/IM3520013T/2020	EPI_ISL_431781	Fujian Center for Disease Control and Prevention	Fujian Center for Disease Control and Prevention	2020-03-19	Lin Qi et al
Fuzhou/JX2012/2020	EPI_ISL_455462	Jiangxi Province Center for Disease Control and Prevention	Jiangxi Province Center for Disease Control and Prevention	2020-02-05	JianXiong Li et al
Gambia/GC19-015/2020	EPI_ISL_428855	MRCG at LSHTM Geomics lab	MRCG at LSHTM Genomics lab	2020-03-17	Sesay et al et al (https://dx.doi.org/10.1101/2020.04.30.070771)
Gambia/GC19-026/2020	EPI_ISL_428856	MRCG at LSHTM Genomics Lab	MRCG at LSHTM Genomics lab	2020-03-21	Sesay et al et al (https://dx.doi.org/10.1101/2020.04.30.070771)
Gambia/GC19-029/2020	EPI_ISL_428857	MRCG at LSHTM Genomics lab	MRCG at LSHTM Genomics lab	2020-04-20	Sesay et al et al (https://dx.doi.org/10.1101/2020.04.30.070771)
Georgia/Tb-1352/2020	EPI_ISL_420140	Department for Virology, Molecular Biology and Genome Research, R. G. Lugar Center for Public Health Research, National Center for Disease Control and Public Health (NCDC) of Georgia.	Department for Virology, Molecular Biology and Genome Research, R. G. Lugar Center for Public Health Research, National Center for Disease Control and Public Health (NCDC) of Georgia.	2020-03-28	Nato Kotaria et al
Georgia/Tb-1523/2020	EPI_ISL_420142	Department for Virology, Molecular Biology and Genome Research, R. G. Lugar Center for Public Health Research, National Center for Disease Control and Public Health (NCDC) of Georgia.	Department for Virology, Molecular Biology and Genome Research, R. G. Lugar Center for Public Health Research, National Center for Disease Control and Public Health (NCDC) of Georgia.	2020-03-30	Marine Murtskhvaladze et al
Georgia/Tb-1679/2020	EPI_ISL_420144	Department for Virology, Molecular Biology and Genome Research, R. G. Lugar Center for Public Health Research, National Center for Disease Control and Public Health (NCDC) of Georgia.	Department for Virology, Molecular Biology and Genome Research, R. G. Lugar Center for Public Health Research, National Center for Disease Control and Public Health (NCDC) of Georgia.	2020-04-01	Gvantsa Chanturia et al
Georgia/Tb-22208/2020	EPI_ISL_470876	Department for Virology, Molecular Biology and Genome Research, R. G. Lugar Center for Public Health Research, National Center for Disease Control and Public Health (NCDC) of Georgia.	Department for Virology, Molecular Biology and Genome Research, R. G. Lugar Center for Public Health Research, National Center for Disease Control and Public Health (NCDC) of Georgia.	2020-05-29	Giorgi Tomashvili et al
Georgia/Tb-273/2020	EPI_ISL_416479	R. G. Lugar Center for Public Health Research, National Center for Disease Control and Public Health (NCDC) of Georgia.	R. G. Lugar Center for Public Health Research, National Center for Disease Control and Public Health (NCDC) of Georgia.	2020-03-05	Marine Murtskhvaladze et al
Georgia/Tb-3118/2020	EPI_ISL_470877	Department for Virology, Molecular Biology and Genome Research, R. G. Lugar Center for Public Health Research, National Center for Disease Control and Public Health (NCDC) of Georgia.	Department for Virology, Molecular Biology and Genome Research, R. G. Lugar Center for Public Health Research, National Center for Disease Control and Public Health (NCDC) of Georgia.	2020-04-15	Gvantsa Brachveli et al
Georgia/Tb-390/2020	EPI_ISL_416477	R. G. Lugar Center for Public Health Research, National Center for Disease Control and Public Health (NCDC) of Georgia.	R. G. Lugar Center for Public Health Research, National Center for Disease Control and Public Health (NCDC) of Georgia.	2020-03-08	Marine Murtskhvaladze et al
Georgia/Tb-477/2020	EPI_ISL_415642	R. G. Lugar Center for Public Health Research, National Center for Disease Control and Public Health (NCDC) of Georgia.	R. G. Lugar Center for Public Health Research, National Center for Disease Control and Public Health (NCDC) of Georgia.	2020-03-10	Nato Kotaria et al

Georgia/Tb-6572/2020	EPI_ISL_447056	Department for Virology, Molecular Biology and Genome Research, R. G. Lugar Center for Public Health Research, National Center for Disease Control and Public Health (NCDC) of Georgia.	Department for Virology, Molecular Biology and Genome Research, R. G. Lugar Center for Public Health Research, National Center for Disease Control and Public Health (NCDC) of Georgia.	2020-04-29	Gvantsa Brachveli et al
Georgia/Tb-6598/2020	EPI_ISL_447055	Department for Virology, Molecular Biology and Genome Research, R. G. Lugar Center for Public Health Research, National Center for Disease Control and Public Health (NCDC) of Georgia.	Department for Virology, Molecular Biology and Genome Research, R. G. Lugar Center for Public Health Research, National Center for Disease Control and Public Health (NCDC) of Georgia.	2020-04-29	Meri Pantsulaia et al
Georgia/Tb-673/2020	EPI_ISL_416478	R. G. Lugar Center for Public Health Research, National Center for Disease Control and Public Health (NCDC) of Georgia.	R. G. Lugar Center for Public Health Research, National Center for Disease Control and Public Health (NCDC) of Georgia.	2020-03-14	Marine Murtskhvaladze et al
Georgia/Tb-712/2020	EPI_ISL_416481	R. G. Lugar Center for Public Health Research, National Center for Disease Control and Public Health (NCDC) of Georgia.	R. G. Lugar Center for Public Health Research, National Center for Disease Control and Public Health (NCDC) of Georgia.	2020-03-16	Gvantsa Chanturia et al
Georgia/Tb-82/2020	EPI_ISL_415644	R. G. Lugar Center for Public Health Research, National Center for Disease Control and Public Health (NCDC) of Georgia.	R. G. Lugar Center for Public Health Research, National Center for Disease Control and Public Health (NCDC) of Georgia.	2020-02-28	Nato Kotaria et al
Georgia/Tb/2020	EPI_ISL_416482	R. G. Lugar Center for Public Health Research, National Center for Disease Control and Public Health (NCDC) of Georgia.	R. G. Lugar Center for Public Health Research, National Center for Disease Control and Public Health (NCDC) of Georgia.	2020-03-13	Adam Kotorashvili et al
Germany/Baden-Wuerttemberg-1/2020	EPI_ISL_412912	State Health Office Baden-Wuerttemberg	Charité Universitätsmedizin Berlin, Institute of Virology	2020-02-25	Victor M Corman et al B (https://dx.doi.org/10.2807/1560-7917.ES.2020.25.13.2000305)
Germany/BAV-MVP0002/2020	EPI_ISL_437204	Max von Pettenkofer Institute, Virology, National Reference Center for Retroviruses, LMU München	Laboratory for Functional Genome Analysis, Dept. Genomics, Gene Center of the LMU Munich	2020-03-05	Max Muenchhoff et al
Germany/BAV-MVP0003/2020	EPI_ISL_437205	Max von Pettenkofer Institute, Virology, National Reference Center for Retroviruses, LMU München	Laboratory for Functional Genome Analysis, Dept. Genomics, Gene Center of the LMU Munich	2020-03-16	Max Muenchhoff et al
Germany/BAV-MVP0005/2020	EPI_ISL_437206	Max von Pettenkofer Institute, Virology, National Reference Center for Retroviruses, LMU München	Laboratory for Functional Genome Analysis, Dept. Genomics, Gene Center of the LMU Munich	2020-03-18	Max Muenchhoff et al
Germany/BAV-MVP0006/2020	EPI_ISL_437207	Max von Pettenkofer Institute, Virology, National Reference Center for Retroviruses, LMU München	Laboratory for Functional Genome Analysis, Dept. Genomics, Gene Center of the LMU Munich	2020-04-09	Max Muenchhoff et al
Germany/BAV-MVP0007/2020	EPI_ISL_437208	Max von Pettenkofer Institute, Virology, National Reference Center for Retroviruses, LMU München	Laboratory for Functional Genome Analysis, Dept. Genomics, Gene Center of the LMU Munich	2020-04-11	Max Muenchhoff et al
Germany/BAV-MVP0008/2020	EPI_ISL_437209	Max von Pettenkofer Institute, Virology, National Reference Center for Retroviruses, LMU München	Laboratory for Functional Genome Analysis, Dept. Genomics, Gene Center of the LMU Munich	2020-04-13	Max Muenchhoff et al
Germany/BAV-MVP0009/2020	EPI_ISL_437210	Max von Pettenkofer Institute, Virology, National Reference Center for Retroviruses, LMU München	Laboratory for Functional Genome Analysis, Dept. Genomics, Gene Center of the LMU Munich	2020-03-07	Max Muenchhoff et al
Germany/BAV-MVP0010/2020	EPI_ISL_437211	Max von Pettenkofer Institute, Virology, National Reference Center for Retroviruses, LMU München	Laboratory for Functional Genome Analysis, Dept. Genomics, Gene Center of the LMU Munich	2020-03-12	Max Muenchhoff et al
Germany/BAV-MVP0011/2020	EPI_ISL_437212	Max von Pettenkofer Institute, Virology, National Reference Center for Retroviruses, LMU München	Laboratory for Functional Genome Analysis, Dept. Genomics, Gene Center of the LMU Munich	2020-03-22	Max Muenchhoff et al
Germany/BAV-MVP0012/2020	EPI_ISL_437213	Max von Pettenkofer Institute, Virology, National Reference Center for Retroviruses, LMU München	Laboratory for Functional Genome Analysis, Dept. Genomics, Gene Center of the LMU Munich	2020-04-05	Max Muenchhoff et al
Germany/BAV-MVP0013/2020	EPI_ISL_437214	Max von Pettenkofer Institute, Virology, National Reference Center for Retroviruses, LMU München	Laboratory for Functional Genome Analysis, Dept. Genomics, Gene Center of the LMU Munich	2020-04-07	Max Muenchhoff et al
Germany/BAV-MVP0014/2020	EPI_ISL_437215	Max von Pettenkofer Institute, Virology, National Reference Center for Retroviruses, LMU München	Laboratory for Functional Genome Analysis, Dept. Genomics, Gene Center of the LMU Munich	2020-04-08	Max Muenchhoff et al
Germany/BAV-MVP0015/2020	EPI_ISL_437216	Max von Pettenkofer Institute, Virology, National Reference Center for Retroviruses, LMU München	Laboratory for Functional Genome Analysis, Dept. Genomics, Gene Center of the LMU Munich	2020-04-09	Max Muenchhoff et al
Germany/BAV-MVP0016/2020	EPI_ISL_437217	Max von Pettenkofer Institute, Virology, National Reference Center for Retroviruses, LMU München	Laboratory for Functional Genome Analysis, Dept. Genomics, Gene Center of the LMU Munich	2020-04-11	Max Muenchhoff et al
Germany/BAV-MVP0017/2020	EPI_ISL_437218	Max von Pettenkofer Institute, Virology, National Reference Center for Retroviruses, LMU München	Laboratory for Functional Genome Analysis, Dept. Genomics, Gene Center of the LMU Munich	2020-03-12	Max Muenchhoff et al
Germany/BAV-MVP0018/2020	EPI_ISL_452103	Max von Pettenkofer Institute, Virology, National Reference Center for Retroviruses, LMU München	Laboratory for Functional Genome Analysis, Dept. Genomics, Gene Center of the LMU Munich	2020-03-25	Max Muenchhoff et al
Germany/BAV-MVP0019/2020	EPI_ISL_437219	Max von Pettenkofer Institute, Virology, National Reference Center for Retroviruses, LMU München	Laboratory for Functional Genome Analysis, Dept. Genomics, Gene Center of the LMU Munich	2020-04-02	Max Muenchhoff et al
Germany/BAV-MVP0020/2020	EPI_ISL_437220	Max von Pettenkofer Institute, Virology, National Reference Center for Retroviruses, LMU München	Laboratory for Functional Genome Analysis, Dept. Genomics, Gene Center of the LMU Munich	2020-04-06	Max Muenchhoff et al
Germany/BAV-MVP0021/2020	EPI_ISL_437221	Max von Pettenkofer Institute, Virology, National Reference Center for Retroviruses, LMU München	Laboratory for Functional Genome Analysis, Dept. Genomics, Gene Center of the LMU Munich	2020-04-06	Max Muenchhoff et al
Germany/BAV-MVP0022/2020	EPI_ISL_437222	Max von Pettenkofer Institute, Virology, National Reference Center for Retroviruses, LMU München	Laboratory for Functional Genome Analysis, Dept. Genomics, Gene Center of the LMU Munich	2020-04-11	Max Muenchhoff et al
Germany/BAV-MVP0023/2020	EPI_ISL_437223	Max von Pettenkofer Institute, Virology, National Reference Center for Retroviruses, LMU München	Laboratory for Functional Genome Analysis, Dept. Genomics, Gene Center of the LMU Munich	2020-03-12	Max Muenchhoff et al
Germany/BAV-MVP0024/2020				2020-03-27	
Germany/BAV-MVP0025/2020	EPI_ISL_437224	Max von Pettenkofer Institute, Virology, National Reference Center for Retroviruses, LMU München	Laboratory for Functional Genome Analysis, Dept. Genomics, Gene Center of the LMU Munich	2020-03-29	Max Muenchhoff et al
Germany/BAV-MVP0026/2020	EPI_ISL_437225	Max von Pettenkofer Institute, Virology, National Reference Center for Retroviruses, LMU München	Laboratory for Functional Genome Analysis, Dept. Genomics, Gene Center of the LMU Munich	2020-04-01	Max Muenchhoff et al
Germany/BAV-MVP0027/2020	EPI_ISL_437226	Max von Pettenkofer Institute, Virology, National Reference Center for Retroviruses, LMU München	Laboratory for Functional Genome Analysis, Dept. Genomics, Gene Center of the LMU Munich	2020-04-10	Max Muenchhoff et al
Germany/BAV-MVP0028/2020	EPI_ISL_437227	Max von Pettenkofer Institute, Virology, National Reference Center for Retroviruses, LMU München	Laboratory for Functional Genome Analysis, Dept. Genomics, Gene Center of the LMU Munich	2020-04-13	Max Muenchhoff et al
Germany/BAV-MVP0029/2020	EPI_ISL_437228	Max von Pettenkofer Institute, Virology, National Reference Center for Retroviruses, LMU München	Laboratory for Functional Genome Analysis, Dept. Genomics, Gene Center of the LMU Munich	2020-03-11	Max Muenchhoff et al
Germany/BAV-MVP0030/2020	EPI_ISL_437229	Max von Pettenkofer Institute, Virology, National Reference Center for Retroviruses, LMU München	Laboratory for Functional Genome Analysis, Dept. Genomics, Gene Center of the LMU Munich	2020-03-14	Max Muenchhoff et al
Germany/BAV-MVP0031/2020	EPI_ISL_437230	Max von Pettenkofer Institute, Virology, National Reference Center for Retroviruses, LMU München	Laboratory for Functional Genome Analysis, Dept. Genomics, Gene Center of the LMU Munich	2020-03-22	Max Muenchhoff et al
Germany/BAV-MVP0033/2020	EPI_ISL_437232	Max von Pettenkofer Institute, Virology, National Reference Center for Retroviruses, LMU München	Laboratory for Functional Genome Analysis, Dept. Genomics, Gene Center of the LMU Munich	2020-03-31	Max Muenchhoff et al
Germany/BAV-MVP0034/2020	EPI_ISL_437233	Max von Pettenkofer Institute, Virology, National Reference Center for Retroviruses, LMU München	Laboratory for Functional Genome Analysis, Dept. Genomics, Gene Center of the LMU Munich	2020-03-13	Max Muenchhoff et al
Germany/BAV-MVP0035/2020	EPI_ISL_437234	Max von Pettenkofer Institute, Virology, National Reference Center for Retroviruses, LMU München	Laboratory for Functional Genome Analysis, Dept. Genomics, Gene Center of the LMU Munich	2020-03-15	Max Muenchhoff et al
Germany/BAV-MVP0036/2020	EPI_ISL_437235	Max von Pettenkofer Institute, Virology, National Reference Center for Retroviruses, LMU München	Laboratory for Functional Genome Analysis, Dept. Genomics, Gene Center of the LMU Munich	2020-03-21	Max Muenchhoff et al
Germany/BAV-MVP0037/2020	EPI_ISL_437236	Max von Pettenkofer Institute, Virology, National Reference Center for Retroviruses, LMU München	Laboratory for Functional Genome Analysis, Dept. Genomics, Gene Center of the LMU Munich	2020-03-22	Max Muenchhoff et al
Germany/BAV-MVP0038/2020	EPI_ISL_437237	Max von Pettenkofer Institute, Virology, National Reference Center for Retroviruses, LMU München	Laboratory for Functional Genome Analysis, Dept. Genomics, Gene Center of the LMU Munich	2020-03-23	Max Muenchhoff et al
Germany/BAV-MVP0039/2020	EPI_ISL_437238	Max von Pettenkofer Institute, Virology, National Reference Center for Retroviruses, LMU München	Laboratory for Functional Genome Analysis, Dept. Genomics, Gene Center of the LMU Munich	2020-03-24	Max Muenchhoff et al
Germany/BAV-MVP0040/2020	EPI_ISL_437239	Max von Pettenkofer Institute, Virology, National Reference Center for Retroviruses, LMU München	Laboratory for Functional Genome Analysis, Dept. Genomics, Gene Center of the LMU Munich	2020-04-02	Max Muenchhoff et al
Germany/BAV-MVP0041/2020	EPI_ISL_437240	Max von Pettenkofer Institute, Virology, National Reference Center for Retroviruses, LMU München	Laboratory for Functional Genome Analysis, Dept. Genomics, Gene Center of the LMU Munich	2020-04-06	Max Muenchhoff et al
Germany/BAV-MVP0042/2020	EPI_ISL_437241	Max von Pettenkofer Institute, Virology, National Reference Center for Retroviruses, LMU München	Laboratory for Functional Genome Analysis, Dept. Genomics, Gene Center of the LMU Munich	2020-04-07	Max Muenchhoff et al
Germany/BAV-MVP0043/2020	EPI_ISL_437242	Max von Pettenkofer Institute, Virology, National Reference Center for Retroviruses, LMU München	Laboratory for Functional Genome Analysis, Dept. Genomics, Gene Center of the LMU Munich	2020-04-09	Max Muenchhoff et al
Germany/BAV-MVP0045/2020	EPI_ISL_437244	Max von Pettenkofer Institute, Virology, National Reference Center for Retroviruses, LMU München	Laboratory for Functional Genome Analysis, Dept. Genomics, Gene Center of the LMU Munich	2020-03-08	Max Muenchhoff et al
Germany/BAV-MVP0046/2020	EPI_ISL_437245	Max von Pettenkofer Institute, Virology, National Reference Center for Retroviruses, LMU München	Laboratory for Functional Genome Analysis, Dept. Genomics, Gene Center of the LMU Munich	2020-03-11	Max Muenchhoff et al
Germany/BAV-MVP0047/2020	EPI_ISL_437246	Max von Pettenkofer Institute, Virology, National Reference Center for Retroviruses, LMU München	Laboratory for Functional Genome Analysis, Dept. Genomics, Gene Center of the LMU Munich	2020-03-11	Max Muenchhoff et al
Germany/BAV-MVP0048/2020	EPI_ISL_437247	Max von Pettenkofer Institute, Virology, National Reference Center for Retroviruses, LMU München	Laboratory for Functional Genome Analysis, Dept. Genomics, Gene Center of the LMU Munich	2020-03-11	Max Muenchhoff et al
Germany/BAV-MVP0049/2020	EPI_ISL_437248	Max von Pettenkofer Institute, Virology, National Reference Center for Retroviruses, LMU München	Laboratory for Functional Genome Analysis, Dept. Genomics, Gene Center of the LMU Munich	2020-03-11	Max Muenchhoff et al
Germany/BAV-MVP0050/2020	EPI_ISL_437249	Max von Pettenkofer Institute, Virology, National Reference Center for Retroviruses, LMU München	Laboratory for Functional Genome Analysis, Dept. Genomics, Gene Center of the LMU Munich	2020-03-12	Max Muenchhoff et al
Germany/BAV-MVP0051/2020	EPI_ISL_437250	Max von Pettenkofer Institute, Virology, National Reference Center for Retroviruses, LMU München	Laboratory for Functional Genome Analysis, Dept. Genomics, Gene Center of the LMU Munich	2020-03-12	Max Muenchhoff et al
Germany/BAV-MVP0052/2020	EPI_ISL_437251	Max von Pettenkofer Institute, Virology, National Reference Center for Retroviruses, LMU München	Laboratory for Functional Genome Analysis, Dept. Genomics, Gene Center of the LMU Munich	2020-03-14	Max Muenchhoff et al
Germany/BAV-MVP0053/2020	EPI_ISL_437252	Max von Pettenkofer Institute, Virology, National Reference Center for Retroviruses, LMU München	Laboratory for Functional Genome Analysis, Dept. Genomics, Gene Center of the LMU Munich	2020-03-16	Max Muenchhoff et al
Germany/BAV-MVP0054/2020	EPI_ISL_437253	Max von Pettenkofer Institute, Virology, National Reference Center for Retroviruses, LMU München	Laboratory for Functional Genome Analysis, Dept. Genomics, Gene Center of the LMU Munich	2020-03-17	Max Muenchhoff et al
Germany/BAV-MVP0056/2020	EPI_ISL_437255	Max von Pettenkofer Institute, Virology, National Reference Center for Retroviruses, LMU München	Laboratory for Functional Genome Analysis, Dept. Genomics, Gene Center of the LMU Munich	2020-03-19	Max Muenchhoff et al
Germany/BAV-MVP0057/2020	EPI_ISL_437256	Max von Pettenkofer Institute, Virology, National Reference Center for Retroviruses, LMU München	Laboratory for Functional Genome Analysis, Dept. Genomics, Gene Center of the LMU Munich	2020-03-20	Max Muenchhoff et al
Germany/BAV-MVP0058/2020	EPI_ISL_437257	Max von Pettenkofer Institute, Virology, National Reference Center for Retroviruses, LMU München	Laboratory for Functional Genome Analysis, Dept. Genomics, Gene Center of the LMU Munich	2020-03-20	Max Muenchhoff et al
Germany/BAV-MVP0059/2020	EPI_ISL_437258	Max von Pettenkofer Institute, Virology, National Reference Center for Retroviruses, LMU München	Laboratory for Functional Genome Analysis, Dept. Genomics, Gene Center of the LMU Munich	2020-03-20	Max Muenchhoff et al
Germany/BAV-MVP0060/2020	EPI_ISL_437259	Max von Pettenkofer Institute, Virology, National Reference Center for Retroviruses, LMU München	Laboratory for Functional Genome Analysis, Dept. Genomics, Gene Center of the LMU Munich	2020-03-21	Max Muenchhoff et al
Germany/BAV-MVP0061/2020	EPI_ISL_437260	Max von Pettenkofer Institute, Virology, National Reference Center for Retroviruses, LMU München	Laboratory for Functional Genome Analysis, Dept. Genomics, Gene Center of the LMU Munich	2020-03-22	Max Muenchhoff et al
Germany/BAV-MVP0062/2020	EPI_ISL_437261	Max von Pettenkofer Institute, Virology, National Reference Center for Retroviruses, LMU München	Laboratory for Functional Genome Analysis, Dept. Genomics, Gene Center of the LMU Munich	2020-03-22	Max Muenchhoff et al
Germany/BAV-MVP0063/2020	EPI_ISL_437262	Max von Pettenkofer Institute, Virology, National Reference Center for Retroviruses, LMU München	Laboratory for Functional Genome Analysis, Dept. Genomics, Gene Center of the LMU Munich	2020-03-22	Max Muenchhoff et al
Germany/BAV-MVP0064/2020	EPI_ISL_437263	Max von Pettenkofer Institute, Virology, National Reference Center for Retroviruses, LMU München	Laboratory for Functional Genome Analysis, Dept. Genomics, Gene Center of the LMU Munich	2020-03-24	Max Muenchhoff et al
Germany/BAV-MVP0067/2020	EPI_ISL_437264	Max von Pettenkofer Institute, Virology, National Reference Center for Retroviruses, LMU München	Laboratory for Functional Genome Analysis, Dept. Genomics, Gene Center of the LMU Munich	2020-03-25	Max Muenchhoff et al
Germany/BAV-MVP0069/2020	EPI_ISL_437265	Max von Pettenkofer Institute, Virology, National Reference Center for Retroviruses, LMU München	Laboratory for Functional Genome Analysis, Dept. Genomics, Gene Center of the LMU Munich	2020-03-25	Max Muenchhoff et al
Germany/BAV-MVP0070/2020	EPI_ISL_437266	Max von Pettenkofer Institute, Virology, National Reference Center for Retroviruses, LMU München	Laboratory for Functional Genome Analysis, Dept. Genomics, Gene Center of the LMU Munich	2020-03-26	Max Muenchhoff et al
Germany/BAV-MVP0071/2020	EPI_ISL_437267	Max von Pettenkofer Institute, Virology, National Reference Center for Retroviruses, LMU München	Laboratory for Functional Genome Analysis, Dept. Genomics, Gene Center of the LMU Munich	2020-03-27	Max Muenchhoff et al
Germany/BAV-MVP0072/2020	EPI_ISL_437268	Max von Pettenkofer Institute, Virology, National Reference Center for Retroviruses, LMU München	Laboratory for Functional Genome Analysis, Dept. Genomics, Gene Center of the LMU Munich	2020-03-28	Max Muenchhoff et al
Germany/BAV-MVP0073/2020	EPI_ISL_437269	Max von Pettenkofer Institute, Virology, National Reference Center for Retroviruses, LMU München	Laboratory for Functional Genome Analysis, Dept. Genomics, Gene Center of the LMU Munich	2020-03-28	Max Muenchhoff et al
Germany/BAV-MVP0075/2020	EPI_ISL_437270	Max von Pettenkofer Institute, Virology, National Reference Center for Retroviruses, LMU München	Laboratory for Functional Genome Analysis, Dept. Genomics, Gene Center of the LMU Munich	2020-03-29	Max Muenchhoff et al
Germany/BAV-MVP0076/2020	EPI_ISL_437271	Max von Pettenkofer Institute, Virology, National Reference Center for Retroviruses, LMU München	Laboratory for Functional Genome Analysis, Dept. Genomics, Gene Center of the LMU Munich	2020-03-30	Max Muenchhoff et al
Germany/BAV-MVP0079/2020	EPI_ISL_437273	Max von Pettenkofer Institute, Virology, National Reference Center for Retroviruses, LMU München	Laboratory for Functional Genome Analysis, Dept. Genomics, Gene Center of the LMU Munich	2020-03-30	Max Muenchhoff et al
Germany/BAV-MVP0080/2020	EPI_ISL_437274	Max von Pettenkofer Institute, Virology, National Reference Center for Retroviruses, LMU München	Laboratory for Functional Genome Analysis, Dept. Genomics, Gene Center of the LMU Munich	2020-03-30	Max Muenchhoff et al
Germany/BAV-MVP0081/2020	EPI_ISL_437275	Max von Pettenkofer Institute, Virology, National Reference Center for Retroviruses, LMU München	Laboratory for Functional Genome Analysis, Dept. Genomics, Gene Center of the LMU Munich	2020-03-30	Max Muenchhoff et al
Germany/BAV-MVP0082/2020	EPI_ISL_437276	Max von Pettenkofer Institute, Virology, National Reference Center for Retroviruses, LMU München	Laboratory for Functional Genome Analysis, Dept. Genomics, Gene Center of the LMU Munich	2020-03-30	Max Muenchhoff et al
Germany/BAV-MVP0083/2020	EPI_ISL_437277	Max von Pettenkofer Institute, Virology, National Reference Center for Retroviruses, LMU München	Laboratory for Functional Genome Analysis, Dept. Genomics, Gene Center of the LMU Munich	2020-03-31	Max Muenchhoff et al
Germany/BAV-MVP0084/2020	EPI_ISL_437278	Max von Pettenkofer Institute, Virology, National Reference Center for Retroviruses, LMU München	Laboratory for Functional Genome Analysis, Dept. Genomics, Gene Center of the LMU Munich	2020-04-01	Max Muenchhoff et al
Germany/BAV-MVP0085/2020	EPI_ISL_437279	Max von Pettenkofer Institute, Virology, National Reference Center for Retroviruses, LMU München	Laboratory for Functional Genome Analysis, Dept. Genomics, Gene Center of the LMU Munich	2020-04-01	Max Muenchhoff et al
Germany/BAV-MVP0086/2020	EPI_ISL_437280	Max von Pettenkofer Institute, Virology, National Reference Center for Retroviruses, LMU München	Laboratory for Functional Genome Analysis, Dept. Genomics, Gene Center of the LMU Munich	2020-04-01	Max Muenchhoff et al
Germany/BAV-MVP0087/2020	EPI_ISL_437281	Max von Pettenkofer Institute, Virology, National Reference Center for Retroviruses, LMU München	Laboratory for Functional Genome Analysis, Dept. Genomics, Gene Center of the LMU Munich	2020-04-01	Max Muenchhoff et al
Germany/BAV-MVP0088/2020	EPI_ISL_437282	Max von Pettenkofer Institute, Virology, National Reference Center for Retroviruses, LMU München	Laboratory for Functional Genome Analysis, Dept. Genomics, Gene Center of the LMU Munich	2020-04-01	Max Muenchhoff et al
Germany/BAV-MVP0089/2020	EPI_ISL_437283	Max von Pettenkofer Institute, Virology, National Reference Center for Retroviruses, LMU München	Laboratory for Functional Genome Analysis, Dept. Genomics, Gene Center of the LMU Munich	2020-04-02	Max Muenchhoff et al
Germany/BAV-MVP0090/2020	EPI_ISL_437284	Max von Pettenkofer Institute, Virology, National Reference Center for Retroviruses, LMU München	Laboratory for Functional Genome Analysis, Dept. Genomics, Gene Center of the LMU Munich	2020-04-02	Max Muenchhoff et al
Germany/BAV-MVP0091/2020	EPI_ISL_437285	Max von Pettenkofer Institute, Virology, National Reference Center for Retroviruses, LMU München	Laboratory for Functional Genome Analysis, Dept. Genomics, Gene Center of the LMU Munich	2020-04-02	Max Muenchhoff et al
Germany/BAV-MVP0092/2020	EPI_ISL_437286	Max von Pettenkofer Institute, Virology, National Reference Center for Retroviruses, LMU München	Laboratory for Functional Genome Analysis, Dept. Genomics, Gene Center of the LMU Munich	2020-04-02	Max Muenchhoff et al
Germany/BAV-MVP0095/2020	EPI_ISL_437287	Max von Pettenkofer Institute, Virology, National Reference Center for Retroviruses, LMU München	Laboratory for Functional Genome Analysis, Dept. Genomics, Gene Center of the LMU Munich	2020-04-03	Max Muenchhoff et al
Germany/BAV-MVP0096/2020	EPI_ISL_437288	Max von Pettenkofer Institute, Virology, National Reference Center for Retroviruses, LMU München	Laboratory for Functional Genome Analysis, Dept. Genomics, Gene Center of the LMU Munich	2020-04-03	Max Muenchhoff et al
Germany/BAV-MVP0097/2020	EPI_ISL_437289	Max von Pettenkofer Institute, Virology, National Reference Center for Retroviruses, LMU München	Laboratory for Functional Genome Analysis, Dept. Genomics, Gene Center of the LMU Munich	2020-04-03	Max Muenchhoff et al
Germany/BAV-MVP0098/2020	EPI_ISL_452104	Max von Pettenkofer Institute, Virology, National Reference Center for Retroviruses, LMU München	Laboratory for Functional Genome Analysis, Dept. Genomics, Gene Center of the LMU Munich	2020-04-04	Max Muenchhoff et al
Germany/BAV-MVP0099/2020	EPI_ISL_437290	Max von Pettenkofer Institute, Virology, National Reference Center for Retroviruses, LMU München	Laboratory for Functional Genome Analysis, Dept. Genomics, Gene Center of the LMU Munich	2020-04-05	Max Muenchhoff et al
Germany/BAV-MVP0100/2020	EPI_ISL_437291	Max von Pettenkofer Institute, Virology, National Reference Center for Retroviruses, LMU München	Laboratory for Functional Genome Analysis, Dept. Genomics, Gene Center of the LMU Munich	2020-04-06	Max Muenchhoff et al
Germany/BAV-MVP0101/2020	EPI_ISL_437292	Max von Pettenkofer Institute, Virology, National Reference Center for Retroviruses, LMU München	Laboratory for Functional Genome Analysis, Dept. Genomics, Gene Center of the LMU Munich	2020-04-07	Max Muenchhoff et al
Germany/BAV-MVP0103/2020	EPI_ISL_437293	Max von Pettenkofer Institute, Virology, National Reference Center for Retroviruses, LMU München	Laboratory for Functional Genome Analysis, Dept. Genomics, Gene Center of the LMU Munich	2020-04-09	Max Muenchhoff et al
Germany/BAV-MVP0104/2020	EPI_ISL_437294	Max von Pettenkofer Institute, Virology, National Reference Center for Retroviruses, LMU München	Laboratory for Functional Genome Analysis, Dept. Genomics, Gene Center of the LMU Munich	2020-04-10	Max Muenchhoff et al
Germany/BAV-MVP0105/2020	EPI_ISL_437295	Max von Pettenkofer Institute, Virology, National Reference Center for Retroviruses, LMU München	Laboratory for Functional Genome Analysis, Dept. Genomics, Gene Center of the LMU Munich	2020-04-12	Max Muenchhoff et al
Germany/BAV-MVP0106/2020	EPI_ISL_437296	Max von Pettenkofer Institute, Virology, National Reference Center for Retroviruses, LMU München	Laboratory for Functional Genome Analysis, Dept. Genomics, Gene Center of the LMU Munich	2020-04-13	Max Muenchhoff et al

Germany/BAV-MVP0107/2020	EPI_ISL_437297	Max von Pettenkofer Institute, Virology, National Reference Center for Retroviruses, LMU München	Laboratory for Functional Genome Analysis, Dept. Genomics, Gene Center of the LMU Munich	2020-04-13	Max Muenchhoff et al
Germany/BAV-MVP0108/2020				2020-04-13	
Germany/BAV-MVP0109/2020				2020-03-11	
Germany/BAV-MVP0111/2020				2020-03-14	
Germany/BAV-MVP0113/2020	EPI_ISL_451937	Max von Pettenkofer Institute, Virology, National Reference Center for Retroviruses, LMU München	Laboratory for Functional Genome Analysis, Dept. Genomics, Gene Center of the LMU Munich	2020-03-17	Max Muenchhoff et al
Germany/BAV-MVP0118/2020	EPI_ISL_451938	Max von Pettenkofer Institute, Virology, National Reference Center for Retroviruses, LMU München	Laboratory for Functional Genome Analysis, Dept. Genomics, Gene Center of the LMU Munich	2020-03-20	Max Muenchhoff et al
Germany/BAV-MVP0119/2020	EPI_ISL_451939	Max von Pettenkofer Institute, Virology, National Reference Center for Retroviruses, LMU München	Laboratory for Functional Genome Analysis, Dept. Genomics, Gene Center of the LMU Munich	2020-03-20	Max Muenchhoff et al
Germany/BAV-MVP0123/2020	EPI_ISL_451940	Max von Pettenkofer Institute, Virology, National Reference Center for Retroviruses, LMU München	Laboratory for Functional Genome Analysis, Dept. Genomics, Gene Center of the LMU Munich	2020-03-23	Max Muenchhoff et al
Germany/BAV-MVP0124/2020	EPI_ISL_451941	Max von Pettenkofer Institute, Virology, National Reference Center for Retroviruses, LMU München	Laboratory for Functional Genome Analysis, Dept. Genomics, Gene Center of the LMU Munich	2020-03-23	Max Muenchhoff et al
Germany/BAV-MVP0151/2020	EPI_ISL_451943	Max von Pettenkofer Institute, Virology, National Reference Center for Retroviruses, LMU München	Laboratory for Functional Genome Analysis, Dept. Genomics, Gene Center of the LMU Munich	2020-03-30	Max Muenchhoff et al
Germany/BAV-MVP0153/2020	EPI_ISL_451944	Max von Pettenkofer Institute, Virology, National Reference Center for Retroviruses, LMU München	Laboratory for Functional Genome Analysis, Dept. Genomics, Gene Center of the LMU Munich	2020-03-31	Max Muenchhoff et al
Germany/BAV-MVP0155/2020	EPI_ISL_451945	Max von Pettenkofer Institute, Virology, National Reference Center for Retroviruses, LMU München	Laboratory for Functional Genome Analysis, Dept. Genomics, Gene Center of the LMU Munich	2020-04-01	Max Muenchhoff et al
Germany/BAV-MVP0157/2020	EPI_ISL_451946	Max von Pettenkofer Institute, Virology, National Reference Center for Retroviruses, LMU München	Laboratory for Functional Genome Analysis, Dept. Genomics, Gene Center of the LMU Munich	2020-04-02	Max Muenchhoff et al
Germany/BAV-MVP0173/2020	EPI_ISL_451947	Max von Pettenkofer Institute, Virology, National Reference Center for Retroviruses, LMU München	Laboratory for Functional Genome Analysis, Dept. Genomics, Gene Center of the LMU Munich	2020-04-08	Max Muenchhoff et al
Germany/BAV-MVP0179/2020				2020-04-12	
Germany/BAV-MVP0182/2020	EPI_ISL_466875	Max von Pettenkofer Institute, Virology, National Reference Center for Retroviruses, LMU München	Laboratory for Functional Genome Analysis, Dept. Genomics, Gene Center of the LMU Munich	2020-04-13	Max Muenchhoff et al
Germany/BAV-MVP0183/2020	EPI_ISL_466876	Max von Pettenkofer Institute, Virology, National Reference Center for Retroviruses, LMU München	Laboratory for Functional Genome Analysis, Dept. Genomics, Gene Center of the LMU Munich	2020-04-13	Max Muenchhoff et al
Germany/BAV-MVP0184/2020	EPI_ISL_466877	Max von Pettenkofer Institute, Virology, National Reference Center for Retroviruses, LMU München	Laboratory for Functional Genome Analysis, Dept. Genomics, Gene Center of the LMU Munich	2020-04-13	Max Muenchhoff et al
Germany/BAV-MVP0185/2020	EPI_ISL_466878	Max von Pettenkofer Institute, Virology, National Reference Center for Retroviruses, LMU München	Laboratory for Functional Genome Analysis, Dept. Genomics, Gene Center of the LMU Munich	2020-04-13	Max Muenchhoff et al
Germany/BAV-MVP0186/2020	EPI_ISL_466879	Max von Pettenkofer Institute, Virology, National Reference Center for Retroviruses, LMU München	Laboratory for Functional Genome Analysis, Dept. Genomics, Gene Center of the LMU Munich	2020-04-13	Max Muenchhoff et al
Germany/BAV-MVP0187/2020	EPI_ISL_466880	Max von Pettenkofer Institute, Virology, National Reference Center for Retroviruses, LMU München	Laboratory for Functional Genome Analysis, Dept. Genomics, Gene Center of the LMU Munich	2020-04-13	Max Muenchhoff et al
Germany/BAV-MVP0188/2020				2020-04-14	
Germany/BAV-MVP0190/2020	EPI_ISL_466881	Max von Pettenkofer Institute, Virology, National Reference Center for Retroviruses, LMU München	Laboratory for Functional Genome Analysis, Dept. Genomics, Gene Center of the LMU Munich	2020-04-14	Max Muenchhoff et al
Germany/BAV-MVP0192/2020	EPI_ISL_466882	Max von Pettenkofer Institute, Virology, National Reference Center for Retroviruses, LMU München	Laboratory for Functional Genome Analysis, Dept. Genomics, Gene Center of the LMU Munich	2020-04-15	Max Muenchhoff et al
Germany/BAV-MVP0193/2020	EPI_ISL_466883	Max von Pettenkofer Institute, Virology, National Reference Center for Retroviruses, LMU München	Laboratory for Functional Genome Analysis, Dept. Genomics, Gene Center of the LMU Munich	2020-04-15	Max Muenchhoff et al
Germany/BAV-MVP0196/2020	EPI_ISL_466884	Max von Pettenkofer Institute, Virology, National Reference Center for Retroviruses, LMU München	Laboratory for Functional Genome Analysis, Dept. Genomics, Gene Center of the LMU Munich	2020-04-16	Max Muenchhoff et al
Germany/BAV-MVP0200/2020	EPI_ISL_466885	Max von Pettenkofer Institute, Virology, National Reference Center for Retroviruses, LMU München	Laboratory for Functional Genome Analysis, Dept. Genomics, Gene Center of the LMU Munich	2020-04-16	Max Muenchhoff et al
Germany/BAV-MVP0201/2020	EPI_ISL_466886	Max von Pettenkofer Institute, Virology, National Reference Center for Retroviruses, LMU München	Laboratory for Functional Genome Analysis, Dept. Genomics, Gene Center of the LMU Munich	2020-04-17	Max Muenchhoff et al
Germany/BAV-MVP0203/2020	EPI_ISL_466887	Max von Pettenkofer Institute, Virology, National Reference Center for Retroviruses, LMU München	Laboratory for Functional Genome Analysis, Dept. Genomics, Gene Center of the LMU Munich	2020-04-17	Max Muenchhoff et al
Germany/BAV-MVP0204/2020				2020-04-17	
Germany/BAV-MVP0207/2020	EPI_ISL_466888	Max von Pettenkofer Institute, Virology, National Reference Center for Retroviruses, LMU München	Laboratory for Functional Genome Analysis, Dept. Genomics, Gene Center of the LMU Munich	2020-04-20	Max Muenchhoff et al
Germany/BAV-MVP0208/2020	EPI_ISL_466889	Max von Pettenkofer Institute, Virology, National Reference Center for Retroviruses, LMU München	Laboratory for Functional Genome Analysis, Dept. Genomics, Gene Center of the LMU Munich	2020-04-19	Max Muenchhoff et al
Germany/BAV-MVP0210/2020	EPI_ISL_466890	Max von Pettenkofer Institute, Virology, National Reference Center for Retroviruses, LMU München	Laboratory for Functional Genome Analysis, Dept. Genomics, Gene Center of the LMU Munich	2020-04-20	Max Muenchhoff et al
Germany/BAV-MVP0214/2020				2020-04-21	
Germany/BAV-MVP0218/2020	EPI_ISL_466891	Max von Pettenkofer Institute, Virology, National Reference Center for Retroviruses, LMU München	Laboratory for Functional Genome Analysis, Dept. Genomics, Gene Center of the LMU Munich	2020-04-22	Max Muenchhoff et al
Germany/BAV-MVP0219/2020	EPI_ISL_466892	Max von Pettenkofer Institute, Virology, National Reference Center for Retroviruses, LMU München	Laboratory for Functional Genome Analysis, Dept. Genomics, Gene Center of the LMU Munich	2020-04-22	Max Muenchhoff et al
Germany/BAV-MVP0220/2020	EPI_ISL_466893	Max von Pettenkofer Institute, Virology, National Reference Center for Retroviruses, LMU München	Laboratory for Functional Genome Analysis, Dept. Genomics, Gene Center of the LMU Munich	2020-04-22	Max Muenchhoff et al
Germany/BAV-MVP0221/2020	EPI_ISL_466894	Max von Pettenkofer Institute, Virology, National Reference Center for Retroviruses, LMU München	Laboratory for Functional Genome Analysis, Dept. Genomics, Gene Center of the LMU Munich	2020-04-22	Max Muenchhoff et al
Germany/BAV-MVP0225/2020	EPI_ISL_466895	Max von Pettenkofer Institute, Virology, National Reference Center for Retroviruses, LMU München	Laboratory for Functional Genome Analysis, Dept. Genomics, Gene Center of the LMU Munich	2020-04-23	Max Muenchhoff et al
Germany/BAV-MVP0226/2020	EPI_ISL_466896	Max von Pettenkofer Institute, Virology, National Reference Center for Retroviruses, LMU München	Laboratory for Functional Genome Analysis, Dept. Genomics, Gene Center of the LMU Munich	2020-04-23	Max Muenchhoff et al
Germany/BAV-MVP0229/2020	EPI_ISL_466897	Max von Pettenkofer Institute, Virology, National Reference Center for Retroviruses, LMU München	Laboratory for Functional Genome Analysis, Dept. Genomics, Gene Center of the LMU Munich	2020-04-24	Max Muenchhoff et al
Germany/BAV-MVP0237/2020	EPI_ISL_466898	Max von Pettenkofer Institute, Virology, National Reference Center for Retroviruses, LMU München	Laboratory for Functional Genome Analysis, Dept. Genomics, Gene Center of the LMU Munich	2020-04-28	Max Muenchhoff et al
Germany/BAV-MVP0240/2020	EPI_ISL_466899	Max von Pettenkofer Institute, Virology, National Reference Center for Retroviruses, LMU München	Laboratory for Functional Genome Analysis, Dept. Genomics, Gene Center of the LMU Munich	2020-04-28	Max Muenchhoff et al
Germany/BAV-MVP0244/2020	EPI_ISL_466900	Max von Pettenkofer Institute, Virology, National Reference Center for Retroviruses, LMU München	Laboratory for Functional Genome Analysis, Dept. Genomics, Gene Center of the LMU Munich	2020-04-30	Max Muenchhoff et al
Germany/BAV-MVP0246/2020	EPI_ISL_466901	Max von Pettenkofer Institute, Virology, National Reference Center for Retroviruses, LMU München	Laboratory for Functional Genome Analysis, Dept. Genomics, Gene Center of the LMU Munich	2020-04-30	Max Muenchhoff et al
Germany/BAV-MVP0247/2020	EPI_ISL_466902	Max von Pettenkofer Institute, Virology, National Reference Center for Retroviruses, LMU München	Laboratory for Functional Genome Analysis, Dept. Genomics, Gene Center of the LMU Munich	2020-04-30	Max Muenchhoff et al
Germany/BAV-MVP0249/2020	EPI_ISL_466903	Max von Pettenkofer Institute, Virology, National Reference Center for Retroviruses, LMU München	Laboratory for Functional Genome Analysis, Dept. Genomics, Gene Center of the LMU Munich	2020-04-30	Max Muenchhoff et al
Germany/BAV-MVP0250/2020	EPI_ISL_466904	Max von Pettenkofer Institute, Virology, National Reference Center for Retroviruses, LMU München	Laboratory for Functional Genome Analysis, Dept. Genomics, Gene Center of the LMU Munich	2020-04-30	Max Muenchhoff et al
Germany/BAV-MVP0251/2020	EPI_ISL_466905	Max von Pettenkofer Institute, Virology, National Reference Center for Retroviruses, LMU München	Laboratory for Functional Genome Analysis, Dept. Genomics, Gene Center of the LMU Munich	2020-05-02	Max Muenchhoff et al
Germany/BAV-MVP0253/2020	EPI_ISL_466906	Max von Pettenkofer Institute, Virology, National Reference Center for Retroviruses, LMU München	Laboratory for Functional Genome Analysis, Dept. Genomics, Gene Center of the LMU Munich	2020-05-04	Max Muenchhoff et al
Germany/BAV-MVP0259/2020	EPI_ISL_466874	Max von Pettenkofer Institute, Virology, National Reference Center for Retroviruses, LMU München	Laboratory for Functional Genome Analysis, Dept. Genomics, Gene Center of the LMU Munich	2020-04-06	Max Muenchhoff et al
Germany/BAV-MVP0264/2020				2020-05-12	
Germany/BAV-MVP0268/2020	EPI_ISL_466910	Max von Pettenkofer Institute, Virology, National Reference Center for Retroviruses, LMU München	Laboratory for Functional Genome Analysis, Dept. Genomics, Gene Center of the LMU Munich	2020-05-17	Max Muenchhoff et al
Germany/BAV-MVP0269/2020	EPI_ISL_466911	Max von Pettenkofer Institute, Virology, National Reference Center for Retroviruses, LMU München	Laboratory for Functional Genome Analysis, Dept. Genomics, Gene Center of the LMU Munich	2020-05-18	Max Muenchhoff et al
Germany/BAV-MVP0270/2020	EPI_ISL_466912	Max von Pettenkofer Institute, Virology, National Reference Center for Retroviruses, LMU München	Laboratory for Functional Genome Analysis, Dept. Genomics, Gene Center of the LMU Munich	2020-05-19	Max Muenchhoff et al
Germany/BAV-MVP0271/2020	EPI_ISL_466914	Max von Pettenkofer Institute, Virology, National Reference Center for Retroviruses, LMU München	Laboratory for Functional Genome Analysis, Dept. Genomics, Gene Center of the LMU Munich	2020-05-20	Max Muenchhoff et al
Germany/BAV-MVP0272/2020	EPI_ISL_466918	Max von Pettenkofer Institute, Virology, National Reference Center for Retroviruses, LMU München	Laboratory for Functional Genome Analysis, Dept. Genomics, Gene Center of the LMU Munich	2020-05-23	Max Muenchhoff et al
Germany/BAV-MVP0273/2020	EPI_ISL_466913	Max von Pettenkofer Institute, Virology, National Reference Center for Retroviruses, LMU München	Laboratory for Functional Genome Analysis, Dept. Genomics, Gene Center of the LMU Munich	2020-05-20	Max Muenchhoff et al
Germany/BAV-MVP0274/2020	EPI_ISL_466915	Max von Pettenkofer Institute, Virology, National Reference Center for Retroviruses, LMU München	Laboratory for Functional Genome Analysis, Dept. Genomics, Gene Center of the LMU Munich	2020-05-22	Max Muenchhoff et al
Germany/BAV-MVP0276/2020	EPI_ISL_466919	Max von Pettenkofer Institute, Virology, National Reference Center for Retroviruses, LMU München	Laboratory for Functional Genome Analysis, Dept. Genomics, Gene Center of the LMU Munich	2020-05-25	Max Muenchhoff et al
Germany/BAV-MVP0277/2020	EPI_ISL_466920	Max von Pettenkofer Institute, Virology, National Reference Center for Retroviruses, LMU München	Laboratory for Functional Genome Analysis, Dept. Genomics, Gene Center of the LMU Munich	2020-05-25	Max Muenchhoff et al
Germany/BAV-MVP0281/2020	EPI_ISL_466908	Max von Pettenkofer Institute, Virology, National Reference Center for Retroviruses, LMU München	Laboratory for Functional Genome Analysis, Dept. Genomics, Gene Center of the LMU Munich	2020-05-07	Max Muenchhoff et al
Germany/BAV-MVP0283/2020	EPI_ISL_466909	Max von Pettenkofer Institute, Virology, National Reference Center for Retroviruses, LMU München	Laboratory for Functional Genome Analysis, Dept. Genomics, Gene Center of the LMU Munich	2020-05-11	Max Muenchhoff et al
Germany/BAV-MVP0284/2020	EPI_ISL_466917	Max von Pettenkofer Institute, Virology, National Reference Center for Retroviruses, LMU München	Laboratory for Functional Genome Analysis, Dept. Genomics, Gene Center of the LMU Munich	2020-05-23	Max Muenchhoff et al
Germany/BAV-MVP0287/2020	EPI_ISL_466921	Max von Pettenkofer Institute, Virology, National Reference Center for Retroviruses, LMU München	Laboratory for Functional Genome Analysis, Dept. Genomics, Gene Center of the LMU Munich	2020-05-25	Max Muenchhoff et al
Germany/BAV-MVP0288/2020	EPI_ISL_466916	Max von Pettenkofer Institute, Virology, National Reference Center for Retroviruses, LMU München	Laboratory for Functional Genome Analysis, Dept. Genomics, Gene Center of the LMU Munich	2020-05-22	Max Muenchhoff et al
Germany/BAV-MVP0289/2020	EPI_ISL_466922	Max von Pettenkofer Institute, Virology, National Reference Center for Retroviruses, LMU München	Laboratory for Functional Genome Analysis, Dept. Genomics, Gene Center of the LMU Munich	2020-05-25	Max Muenchhoff et al
Germany/BAV-MVP0291/2020	EPI_ISL_466923	Max von Pettenkofer Institute, Virology, National Reference Center for Retroviruses, LMU München	Laboratory for Functional Genome Analysis, Dept. Genomics, Gene Center of the LMU Munich	2020-05-25	Max Muenchhoff et al
Germany/BAV-MVP0292/2020				2020-05-25	
Germany/BAV-MVP0294/2020	EPI_ISL_466924	Max von Pettenkofer Institute, Virology, National Reference Center for Retroviruses, LMU München	Laboratory for Functional Genome Analysis, Dept. Genomics, Gene Center of the LMU Munich	2020-05-26	Max Muenchhoff et al
Germany/BAV-MVP0295/2020				2020-05-26	
Germany/BAV-MVP0296/2020	EPI_ISL_466925	Max von Pettenkofer Institute, Virology, National Reference Center for Retroviruses, LMU München	Laboratory for Functional Genome Analysis, Dept. Genomics, Gene Center of the LMU Munich	2020-05-26	Max Muenchhoff et al
Germany/BAV-MVP0298/2020				2020-05-22	
Germany/BAV-V2010486/2020	EPI_ISL_420898	Max von Pettenkofer Institute, Virology, National Reference Center for Retroviruses, LMU Munich	Laboratory for Functional Genome Analysis, Dept. Genomics, Gene Center of the LMU Munich	2020-03-11	Max Muenchhoff et al
Germany/BAV-V2010492/2020	EPI_ISL_420899	Max von Pettenkofer Institute, Virology, National Reference Center for Retroviruses, LMU Munich	Laboratory for Functional Genome Analysis, Dept. Genomics, Gene Center of the LMU Munich	2020-03-11	Max Muenchhoff et al
Germany/BAV-V2010517/2020				2020-03-11	
Germany/BAV-V2010837/2020	EPI_ISL_420900	Max von Pettenkofer Institute, Virology, National Reference Center for Retroviruses, LMU Munich	Laboratory for Functional Genome Analysis, Dept. Genomics, Gene Center of the LMU Munich	2020-03-12	Max Muenchhoff et al
Germany/BAV-V2010840/2020	EPI_ISL_420901	Max von Pettenkofer Institute, Virology, National Reference Center for Retroviruses, LMU Munich	Laboratory for Functional Genome Analysis, Dept. Genomics, Gene Center of the LMU Munich	2020-03-12	Max Muenchhoff et al
Germany/BAV-V2011376/2020	EPI_ISL_420902	Max von Pettenkofer Institute, Virology, National Reference Center for Retroviruses, LMU Munich	Laboratory for Functional Genome Analysis, Dept. Genomics, Gene Center of the LMU Munich	2020-03-13	Max Muenchhoff et al
Germany/BAV-V2011463/2020	EPI_ISL_420904	Max von Pettenkofer Institute, Virology, National Reference Center for Retroviruses, LMU Munich	Laboratory for Functional Genome Analysis, Dept. Genomics, Gene Center of the LMU Munich	2020-03-15	Max Muenchhoff et al
Germany/BAV-V2011672/2020	EPI_ISL_420905	Max von Pettenkofer Institute, Virology, National Reference Center for Retroviruses, LMU Munich	Laboratory for Functional Genome Analysis, Dept. Genomics, Gene Center of the LMU Munich	2020-03-16	Max Muenchhoff et al
Germany/BAV-V2011673/2020	EPI_ISL_420906	Max von Pettenkofer Institute, Virology, National Reference Center for Retroviruses, LMU Munich	Laboratory for Functional Genome Analysis, Dept. Genomics, Gene Center of the LMU Munich	2020-03-16	Max Muenchhoff et al
Germany/BAV-V2011674/2020				2020-03-16	
Germany/BAV-V2011675/2020	EPI_ISL_420907	Max von Pettenkofer Institute, Virology, National Reference Center for Retroviruses, LMU Munich	Laboratory for Functional Genome Analysis, Dept. Genomics, Gene Center of the LMU Munich	2020-03-16	Max Muenchhoff et al
Germany/BAV-V2012612/2020	EPI_ISL_420908	Max von Pettenkofer Institute, Virology, National Reference Center for Retroviruses, LMU Munich	Laboratory for Functional Genome Analysis, Dept. Genomics, Gene Center of the LMU Munich	2020-03-19	Max Muenchhoff et al
Germany/BAV-V2012620/2020	EPI_ISL_420909	Max von Pettenkofer Institute, Virology, National Reference Center for Retroviruses, LMU Munich	Laboratory for Functional Genome Analysis, Dept. Genomics, Gene Center of the LMU Munich	2020-03-19	Max Muenchhoff et al
Germany/BAV-V2012622/2020	EPI_ISL_420911	Max von Pettenkofer Institute, Virology, National Reference Center for Retroviruses, LMU Munich	Laboratory for Functional Genome Analysis, Dept. Genomics, Gene Center of the LMU Munich	2020-03-19	Max Muenchhoff et al
Germany/BAV-V2012856/2020	EPI_ISL_420912	Max von Pettenkofer Institute, Virology, National Reference Center for Retroviruses, LMU Munich	Laboratory for Functional Genome Analysis, Dept. Genomics, Gene Center of the LMU Munich	2020-03-21	Max Muenchhoff et al
Germany/BavPat1-ChVir929/2020	EPI_ISL_450198		Department of Virology	2020-01-28	Boehmer et al
Germany/BavPat1/2020	EPI_ISL_406862	Charité Universitätsmedizin Berlin, Institute of Virology; Institut für Mikrobiologie der Bundeswehr, Munich	Charité Universitätsmedizin Berlin, Institute of Virology	2020-01-28	Victor M Corman et al B (https://dx.doi.org/10.2807/1560-7917.ES.2020.25.13.2000305)
Germany/BavPat10-ChVir1482/2020	EPI_ISL_450207		Department of Virology	2020-02-07	Boehmer et al
Germany/BavPat14-ChVir1483/2020	EPI_ISL_450208		Department of Virology	2020-02-08	Boehmer et al
Germany/BavPat2-ChVir984-ChVir1017/2020	EPI_ISL_450209		Department of Virology	2020-01-30	Boehmer et al
Germany/BavPat2/2020	EPI_ISL_414520	Bundeswehr Institute of Microbiology	Bundeswehr Institute of Microbiology	2020-03-02	Mathias C Walter et al
Germany/BavPat3-ChVir1020/2020	EPI_ISL_450200		Department of Virology	2020-01-28	Boehmer et al
Germany/BavPat4-ChVir1019/2020	EPI_ISL_450199		Department of Virology	2020-01-29	Boehmer et al
Germany/BavPat7-ChVir1289/2020	EPI_ISL_450203		Department of Virology	2020-02-01	Boehmer et al
Germany/BavPat8-ChVir1356/2020	EPI_ISL_450204		Department of Virology	2020-02-03	Boehmer et al
Germany/Cento-70001814/2020	EPI_ISL_459962	Centogene AG	Centogene AG	2020-04-01	Prof. Dr. Peter Bauer et al
Germany/Cento-70004280/2020	EPI_ISL_459963	Centogene AG	Centogene AG	2020-04-01	Prof. Dr. Peter Bauer et al
Germany/COV7/2020-03-26	EPI_ISL_457750	Centogene AG	Centogene AG	2020-03-26	Prof. Dr. Peter Bauer et al
Germany/FrankfurtFFM3/2020	EPI_ISL_452219	University Hospital Frankfurt	Widera/Toptan	2020-03-02	Tuna Toptan1 et al
Germany/FrankfurtFFM4/2020	EPI_ISL_452220	University Hospital Frankfurt	Widera/Toptan	2020-03-02	Tuna Toptan1 et al
Germany/FrankfurtFFM5/2020	EPI_ISL_452221	University Hospital Frankfurt	Widera/Toptan	2020-03-02	Tuna Toptan1 et al

Germany/FrankfurtFFM6/2020	EPI_ISL_452222	University Hospital Frankfurt	Widera/Toptan	2020-03-02	Tuna Toptan1 et al
Germany/FrankfurtFFM7/2020	EPI_ISL_452223	University Hospital Frankfurt	Widera/Toptan	2020-03-02	Tuna Toptan1 et al
Germany/HH-1/2020	EPI_ISL_450413		Center for Diagnostics	2020-03-19	Huang et al
Germany/Muenster_FI1103201/2020	EPI_ISL_463008	Inst. of Molecular Virology	Inst. Hygiene	2020-03-11	Angeles Mecate Zambrano et al
Germany/NRW-01/2020	EPI_ISL_413488	Center of Medical Microbiology, Virology, and Hospital Hygiene, University of Duesseldorf	Center of Medical Microbiology, Virology, and Hospital Hygiene, University of Duesseldorf	2020-02-28	Ortwin Adams et al A (https://dx.doi.org/10.1101/2020.04.25.20079517)
Germany/NRW-011/2020	EPI_ISL_414507	Center of Medical Microbiology, Virology, and Hospital Hygiene, University of Duesseldorf	Center of Medical Microbiology, Virology, and Hospital Hygiene, University of Duesseldorf	2020-03-03	Ortwin Adams et al B
Germany/NRW-02-1/2020	EPI_ISL_414497	Center of Medical Microbiology, Virology, and Hospital Hygiene, University of Duesseldorf	Center of Medical Microbiology, Virology, and Hospital Hygiene, University of Duesseldorf	2020-02-25	Ortwin Adams et al A (https://dx.doi.org/10.1101/2020.04.25.20079517)
Germany/NRW-03/2020	EPI_ISL_414498	Center of Medical Microbiology, Virology, and Hospital Hygiene, University of Duesseldorf	Center of Medical Microbiology, Virology, and Hospital Hygiene, University of Duesseldorf	2020-02-26	Ortwin Adams et al A (https://dx.doi.org/10.1101/2020.04.25.20079517)
Germany/NRW-04/2020	EPI_ISL_414499	Center of Medical Microbiology, Virology, and Hospital Hygiene, University of Duesseldorf	Center of Medical Microbiology, Virology, and Hospital Hygiene, University of Duesseldorf	2020-02-26	Ortwin Adams et al A (https://dx.doi.org/10.1101/2020.04.25.20079517)
Germany/NRW-05/2020	EPI_ISL_414504	Center of Medical Microbiology, Virology, and Hospital Hygiene, University of Duesseldorf	Center of Medical Microbiology, Virology, and Hospital Hygiene, University of Duesseldorf	2020-02-26	Ortwin Adams et al A (https://dx.doi.org/10.1101/2020.04.25.20079517)
Germany/NRW-06/2020	EPI_ISL_414505	Center of Medical Microbiology, Virology, and Hospital Hygiene, University of Duesseldorf	Center of Medical Microbiology, Virology, and Hospital Hygiene, University of Duesseldorf	2020-02-27	Ortwin Adams et al A (https://dx.doi.org/10.1101/2020.04.25.20079517)
Germany/NRW-07/2020	EPI_ISL_414506	Center of Medical Microbiology, Virology, and Hospital Hygiene, University of Duesseldorf	Center of Medical Microbiology, Virology, and Hospital Hygiene, University of Duesseldorf	2020-02-27	Ortwin Adams et al A (https://dx.doi.org/10.1101/2020.04.25.20079517)
Germany/NRW-08/2020	EPI_ISL_414508	Center of Medical Microbiology, Virology, and Hospital Hygiene, University of Duesseldorf	Center of Medical Microbiology, Virology, and Hospital Hygiene, University of Duesseldorf	2020-02-27	Ortwin Adams et al A (https://dx.doi.org/10.1101/2020.04.25.20079517)
Germany/NRW-09/2020	EPI_ISL_414509	Center of Medical Microbiology, Virology, and Hospital Hygiene, University of Duesseldorf	Center of Medical Microbiology, Virology, and Hospital Hygiene, University of Duesseldorf	2020-02-28	Ortwin Adams et al A (https://dx.doi.org/10.1101/2020.04.25.20079517)
Germany/NRW-10/2020	EPI_ISL_414574	Center of Medical Microbiology, Virology, and Hospital Hygiene, University of Duesseldorf	Center of Medical Microbiology, Virology, and Hospital Hygiene, University of Duesseldorf	2020-02-28	Ortwin Adams et al A (https://dx.doi.org/10.1101/2020.04.25.20079517)
Germany/NRW-12/2020	EPI_ISL_417457	Center of Medical Microbiology, Virology, and Hospital Hygiene, University of Duesseldorf	Center of Medical Microbiology, Virology, and Hospital Hygiene, University of Duesseldorf	2020-03-10	Ortwin Adams et al A (https://dx.doi.org/10.1101/2020.04.25.20079517)
Germany/NRW-13/2020	EPI_ISL_417458	Center of Medical Microbiology, Virology, and Hospital Hygiene, University of Duesseldorf	Center of Medical Microbiology, Virology, and Hospital Hygiene, University of Duesseldorf	2020-03-11	Ortwin Adams et al A (https://dx.doi.org/10.1101/2020.04.25.20079517)
Germany/NRW-14/2020	EPI_ISL_417459	Center of Medical Microbiology, Virology, and Hospital Hygiene, University of Duesseldorf	Center of Medical Microbiology, Virology, and Hospital Hygiene, University of Duesseldorf	2020-03-11	Ortwin Adams et al A (https://dx.doi.org/10.1101/2020.04.25.20079517)
Germany/NRW-15/2020	EPI_ISL_417460	Center of Medical Microbiology, Virology, and Hospital Hygiene, University of Duesseldorf	Center of Medical Microbiology, Virology, and Hospital Hygiene, University of Duesseldorf	2020-03-11	Ortwin Adams et al A (https://dx.doi.org/10.1101/2020.04.25.20079517)
Germany/NRW-16/2020	EPI_ISL_417461	Center of Medical Microbiology, Virology, and Hospital Hygiene, University of Duesseldorf	Center of Medical Microbiology, Virology, and Hospital Hygiene, University of Duesseldorf	2020-03-11	Ortwin Adams et al A (https://dx.doi.org/10.1101/2020.04.25.20079517)
Germany/NRW-17/2020	EPI_ISL_417462	Center of Medical Microbiology, Virology, and Hospital Hygiene, University of Duesseldorf	Center of Medical Microbiology, Virology, and Hospital Hygiene, University of Duesseldorf	2020-03-11	Ortwin Adams et al A (https://dx.doi.org/10.1101/2020.04.25.20079517)
Germany/NRW-18/2020	EPI_ISL_417463	Center of Medical Microbiology, Virology, and Hospital Hygiene, University of Duesseldorf	Center of Medical Microbiology, Virology, and Hospital Hygiene, University of Duesseldorf	2020-03-13	Ortwin Adams et al A (https://dx.doi.org/10.1101/2020.04.25.20079517)
Germany/NRW-19/2020	EPI_ISL_417464	Center of Medical Microbiology, Virology, and Hospital Hygiene, University of Duesseldorf	Center of Medical Microbiology, Virology, and Hospital Hygiene, University of Duesseldorf	2020-03-14	Ortwin Adams et al A (https://dx.doi.org/10.1101/2020.04.25.20079517)
Germany/NRW-20/2020	EPI_ISL_417465	Center of Medical Microbiology, Virology, and Hospital Hygiene, University of Duesseldorf	Center of Medical Microbiology, Virology, and Hospital Hygiene, University of Duesseldorf	2020-03-14	Ortwin Adams et al A (https://dx.doi.org/10.1101/2020.04.25.20079517)
Germany/NRW-21/2020	EPI_ISL_417466	Center of Medical Microbiology, Virology, and Hospital Hygiene, University of Duesseldorf	Center of Medical Microbiology, Virology, and Hospital Hygiene, University of Duesseldorf	2020-03-14	Ortwin Adams et al A (https://dx.doi.org/10.1101/2020.04.25.20079517)
Germany/NRW-22/2020	EPI_ISL_417467	Center of Medical Microbiology, Virology, and Hospital Hygiene, University of Duesseldorf	Center of Medical Microbiology, Virology, and Hospital Hygiene, University of Duesseldorf	2020-03-15	Ortwin Adams et al A (https://dx.doi.org/10.1101/2020.04.25.20079517)
Germany/NRW-23/2020	EPI_ISL_417468	Center of Medical Microbiology, Virology, and Hospital Hygiene, University of Duesseldorf	Center of Medical Microbiology, Virology, and Hospital Hygiene, University of Duesseldorf	2020-03-16	Ortwin Adams et al A (https://dx.doi.org/10.1101/2020.04.25.20079517)
Germany/NRW-24/2020	EPI_ISL_419541	Center of Medical Microbiology, Virology, and Hospital Hygiene, University of Duesseldorf	Center of Medical Microbiology, Virology, and Hospital Hygiene, University of Duesseldorf	2020-03-14	Ortwin Adams et al A (https://dx.doi.org/10.1101/2020.04.25.20079517)
Germany/NRW-25/2020	EPI_ISL_419542	Center of Medical Microbiology, Virology, and Hospital Hygiene, University of Duesseldorf	Center of Medical Microbiology, Virology, and Hospital Hygiene, University of Duesseldorf	2020-03-15	Ortwin Adams et al A (https://dx.doi.org/10.1101/2020.04.25.20079517)
Germany/NRW-26/2020	EPI_ISL_419543	Center of Medical Microbiology, Virology, and Hospital Hygiene, University of Duesseldorf	Center of Medical Microbiology, Virology, and Hospital Hygiene, University of Duesseldorf	2020-03-15	Ortwin Adams et al A (https://dx.doi.org/10.1101/2020.04.25.20079517)
Germany/NRW-27/2020	EPI_ISL_419544	Center of Medical Microbiology, Virology, and Hospital Hygiene, University of Duesseldorf	Center of Medical Microbiology, Virology, and Hospital Hygiene, University of Duesseldorf	2020-03-15	Ortwin Adams et al A (https://dx.doi.org/10.1101/2020.04.25.20079517)
Germany/NRW-28/2020	EPI_ISL_419545	Center of Medical Microbiology, Virology, and Hospital Hygiene, University of Duesseldorf	Center of Medical Microbiology, Virology, and Hospital Hygiene, University of Duesseldorf	2020-03-15	Ortwin Adams et al A (https://dx.doi.org/10.1101/2020.04.25.20079517)
Germany/NRW-29/2020	EPI_ISL_419546	Center of Medical Microbiology, Virology, and Hospital Hygiene, University of Duesseldorf	Center of Medical Microbiology, Virology, and Hospital Hygiene, University of Duesseldorf	2020-03-15	Ortwin Adams et al A (https://dx.doi.org/10.1101/2020.04.25.20079517)
Germany/NRW-30/2020	EPI_ISL_419547	Center of Medical Microbiology, Virology, and Hospital Hygiene, University of Duesseldorf	Center of Medical Microbiology, Virology, and Hospital Hygiene, University of Duesseldorf	2020-03-15	Ortwin Adams et al A (https://dx.doi.org/10.1101/2020.04.25.20079517)
Germany/NRW-32/2020	EPI_ISL_419549	Center of Medical Microbiology, Virology, and Hospital Hygiene, University of Duesseldorf	Center of Medical Microbiology, Virology, and Hospital Hygiene, University of Duesseldorf	2020-03-15	Ortwin Adams et al A (https://dx.doi.org/10.1101/2020.04.25.20079517)
Germany/NRW-33/2020	EPI_ISL_419550	Center of Medical Microbiology, Virology, and Hospital Hygiene, University of Duesseldorf	Center of Medical Microbiology, Virology, and Hospital Hygiene, University of Duesseldorf	2020-03-16	Ortwin Adams et al A (https://dx.doi.org/10.1101/2020.04.25.20079517)
Germany/NRW-34/2020	EPI_ISL_419551	Center of Medical Microbiology, Virology, and Hospital Hygiene, University of Duesseldorf	Center of Medical Microbiology, Virology, and Hospital Hygiene, University of Duesseldorf	2020-03-16	Ortwin Adams et al A (https://dx.doi.org/10.1101/2020.04.25.20079517)
Germany/NRW-36/2020	EPI_ISL_425120	Center of Medical Microbiology, Virology, and Hospital Hygiene, University of Duesseldorf	Center of Medical Microbiology, Virology, and Hospital Hygiene, University of Duesseldorf	2020-03-16	Ortwin Adams et al A (https://dx.doi.org/10.1101/2020.04.25.20079517)
Germany/NRW-37/2020	EPI_ISL_425121	Center of Medical Microbiology, Virology, and Hospital Hygiene, University of Duesseldorf	Center of Medical Microbiology, Virology, and Hospital Hygiene, University of Duesseldorf	2020-03-16	Ortwin Adams et al A (https://dx.doi.org/10.1101/2020.04.25.20079517)
Germany/NRW-38/2020	EPI_ISL_425122	Center of Medical Microbiology, Virology, and Hospital Hygiene, University of Duesseldorf	Center of Medical Microbiology, Virology, and Hospital Hygiene, University of Duesseldorf	2020-03-16	Ortwin Adams et al A (https://dx.doi.org/10.1101/2020.04.25.20079517)
Germany/NRW-39/2020	EPI_ISL_425123	Center of Medical Microbiology, Virology, and Hospital Hygiene, University of Duesseldorf	Center of Medical Microbiology, Virology, and Hospital Hygiene, University of Duesseldorf	2020-03-17	Ortwin Adams et al A (https://dx.doi.org/10.1101/2020.04.25.20079517)
Germany/NRW-40/2020	EPI_ISL_425124	Center of Medical Microbiology, Virology, and Hospital Hygiene, University of Duesseldorf	Center of Medical Microbiology, Virology, and Hospital Hygiene, University of Duesseldorf	2020-03-17	Ortwin Adams et al A (https://dx.doi.org/10.1101/2020.04.25.20079517)
Germany/NRW-41/2020	EPI_ISL_425125	Center of Medical Microbiology, Virology, and Hospital Hygiene, University of Duesseldorf	Center of Medical Microbiology, Virology, and Hospital Hygiene, University of Duesseldorf	2020-03-18	Ortwin Adams et al A (https://dx.doi.org/10.1101/2020.04.25.20079517)
Germany/NRW-42/2020	EPI_ISL_425126	Center of Medical Microbiology, Virology, and Hospital Hygiene, University of Duesseldorf	Center of Medical Microbiology, Virology, and Hospital Hygiene, University of Duesseldorf	2020-03-18	Ortwin Adams et al A (https://dx.doi.org/10.1101/2020.04.25.20079517)
Germany/NRW-43/2020	EPI_ISL_425138	Center of Medical Microbiology, Virology, and Hospital Hygiene, University of Duesseldorf	Center of Medical Microbiology, Virology, and Hospital Hygiene, University of Duesseldorf	2020-03-19	Ortwin Adams et al A (https://dx.doi.org/10.1101/2020.04.25.20079517)
Germany/NRW-44/2020	EPI_ISL_425139	Center of Medical Microbiology, Virology, and Hospital Hygiene, University of Duesseldorf	Center of Medical Microbiology, Virology, and Hospital Hygiene, University of Duesseldorf	2020-03-19	Ortwin Adams et al A (https://dx.doi.org/10.1101/2020.04.25.20079517)
Germany/NRW-45/2020	EPI_ISL_425129	Center of Medical Microbiology, Virology, and Hospital Hygiene, University of Duesseldorf	Center of Medical Microbiology, Virology, and Hospital Hygiene, University of Duesseldorf	2020-03-20	Ortwin Adams et al A (https://dx.doi.org/10.1101/2020.04.25.20079517)
Germany/NRW-46/2020	EPI_ISL_425130	Center of Medical Microbiology, Virology, and Hospital Hygiene, University of Duesseldorf	Center of Medical Microbiology, Virology, and Hospital Hygiene, University of Duesseldorf	2020-03-21	Ortwin Adams et al A (https://dx.doi.org/10.1101/2020.04.25.20079517)
Germany/NRW-47/2020	EPI_ISL_425131	Center of Medical Microbiology, Virology, and Hospital Hygiene, University of Duesseldorf	Center of Medical Microbiology, Virology, and Hospital Hygiene, University of Duesseldorf	2020-03-20	Ortwin Adams et al A (https://dx.doi.org/10.1101/2020.04.25.20079517)
Germany/NRW-48/2020	EPI_ISL_425132	Center of Medical Microbiology, Virology, and Hospital Hygiene, University of Duesseldorf	Center of Medical Microbiology, Virology, and Hospital Hygiene, University of Duesseldorf	2020-03-23	Ortwin Adams et al A (https://dx.doi.org/10.1101/2020.04.25.20079517)
Germany/NRW-49/2020	EPI_ISL_425133	Center of Medical Microbiology, Virology, and Hospital Hygiene, University of Duesseldorf	Center of Medical Microbiology, Virology, and Hospital Hygiene, University of Duesseldorf	2020-03-10	Ortwin Adams et al A (https://dx.doi.org/10.1101/2020.04.25.20079517)
Germany/NRW-50/2020	EPI_ISL_425134	Center of Medical Microbiology, Virology, and Hospital Hygiene, University of Duesseldorf	Center of Medical Microbiology, Virology, and Hospital Hygiene, University of Duesseldorf	2020-03-11	Ortwin Adams et al A (https://dx.doi.org/10.1101/2020.04.25.20079517)
Germany/NRW-51/2020	EPI_ISL_425135	Center of Medical Microbiology, Virology, and Hospital Hygiene, University of Duesseldorf	Center of Medical Microbiology, Virology, and Hospital Hygiene, University of Duesseldorf	2020-03-13	Ortwin Adams et al A (https://dx.doi.org/10.1101/2020.04.25.20079517)
Germany/NRW-52/2020	EPI_ISL_425136	Center of Medical Microbiology, Virology, and Hospital Hygiene, University of Duesseldorf	Center of Medical Microbiology, Virology, and Hospital Hygiene, University of Duesseldorf	2020-03-14	Ortwin Adams et al A (https://dx.doi.org/10.1101/2020.04.25.20079517)
Germany/NRW-53/2020	EPI_ISL_425137	Center of Medical Microbiology, Virology, and Hospital Hygiene, University of Duesseldorf	Center of Medical Microbiology, Virology, and Hospital Hygiene, University of Duesseldorf	2020-03-14	Ortwin Adams et al A (https://dx.doi.org/10.1101/2020.04.25.20079517)
Ghana/1565_S13/2020	EPI_ISL_422404	NMIMR, Department of Virology	WACCBIP, University of Ghana	2020-03-24	Joyce M. Ngoi et al
Ghana/1622_S2/2020	EPI_ISL_422384	NMIMR, Department of Virology	WACCBIP, University of Ghana	2020-03-24	Joyce M. Ngoi et al
Ghana/1651_S3/2020	EPI_ISL_422387	NMIMR, Department of Virology	WACCBIP, University of Ghana	2020-03-25	Joyce M. Ngoi et al
Ghana/1659_S14/2020	EPI_ISL_422405	NMIMR, Department of Virology	WACCBIP, University of Ghana	2020-03-25	Joyce M. Ngoi et al
Ghana/2333_S5/2020	EPI_ISL_422394	NMIMR, Department of Virology	WACCBIP, University of Ghana	2020-03-27	Joyce M. Ngoi et al
Ghana/2828_S6/2020	EPI_ISL_422397	NMIMR, Department of Virology	WACCBIP, University of Ghana	2020-03-29	Joyce M. Ngoi et al
Ghana/2850_S15/2020	EPI_ISL_422406	NMIMR, Department of Virology	WACCBIP, University of Ghana	2020-03-29	Joyce M. Ngoi et al
Ghana/2853_S7/2020	EPI_ISL_422398	NMIMR, Department of Virology	WACCBIP, University of Ghana	2020-03-29	Joyce M. Ngoi et al
Ghana/2914_S8/2020	EPI_ISL_422399	NMIMR, Department of Virology	WACCBIP, University of Ghana	2020-03-30	Joyce M. Ngoi et al
Ghana/3176_S11/2020	EPI_ISL_422402	NMIMR, Department of Virology	WACCBIP, University of Ghana	2020-03-30	Joyce M. Ngoi et al
Ghana/3177_S12/2020	EPI_ISL_422403	NMIMR, Department of Virology	WACCBIP, University of Ghana	2020-03-30	Joyce M. Ngoi et al
Greece/12/2020	EPI_ISL_418264	Laboratory of Microbiology, Department of Medicine, National and Kapodistrian University of Athens, Greece	Laboratory of Biology, Department of Medicine, Democritus University of Thrace, Greece	2020-03-18	Maria Bampali et al
Greece/127_HPI/2020	EPI_ISL_430469	Hellenic Pasteur Institute, Public Health Laboratories	Hellenic Pasteur Institute, Public Health Laboratories, Unit of Bioinformatics and Applied Genomics	2020-02-29	Vasiliki Pogka et al
Greece/13/2020	EPI_ISL_427043	Laboratory of Microbiology, Medical School, National and Kapodistrian University of Athens	Laboratory of Biology, Department of Medicine, Democritus University of Thrace	2020-03-18	Bampali et al
Greece/136/2020	EPI_ISL_434473	Laboratory of Microbiology, Medical School, National and Kapodistrian University of Athens	Laboratory of Biology, Department of Medicine, Democritus University of Thrace	2020-03-11	Kassela K. et al (https://dx.doi.org/10.1101/2020.06.03.20121236)
Greece/137/2020	EPI_ISL_434474	Laboratory of Microbiology, Medical School, National and Kapodistrian University of Athens	Laboratory of Biology, Department of Medicine, Democritus University of Thrace	2020-03-11	Kassela K. et al (https://dx.doi.org/10.1101/2020.06.03.20121236)
Greece/138/2020	EPI_ISL_434479	Laboratory of Microbiology, Medical School, National and Kapodistrian University of Athens	Laboratory of Biology, Department of Medicine, Democritus University of Thrace	2020-03-11	Kassela K. et al (https://dx.doi.org/10.1101/2020.06.03.20121236)
Greece/143/2020	EPI_ISL_434477	Laboratory of Microbiology, Medical School, National and Kapodistrian University of Athens	Laboratory of Biology, Department of Medicine, Democritus University of Thrace	2020-03-16	Kassela K. et al (https://dx.doi.org/10.1101/2020.06.03.20121236)
Greece/145_34726/2020	EPI_ISL_447836		Department of Medicine	2020-03-16	Kassela et al (https://dx.doi.org/10.1101/2020.06.03.20121236)
Greece/149_32357/2020	EPI_ISL_447635		Department of Medicine	2020-03-12	Kassela et al (https://dx.doi.org/10.1101/2020.06.03.20121236)
Greece/149/2020	EPI_ISL_434471	Laboratory of Microbiology, Medical School, National and Kapodistrian University of Athens	Laboratory of Biology, Department of Medicine, Democritus University of Thrace	2020-03-11	Kassela K. et al (https://dx.doi.org/10.1101/2020.06.03.20121236)
Greece/16/2020	EPI_ISL_418265	Laboratory of Microbiology, Department of Medicine, National and Kapodistrian University of Athens, Greece	Laboratory of Biology, Department of Medicine, Democritus University of Thrace, Greece	2020-03-18	Maria Bampali et al
Greece/203_33026/2020	EPI_ISL_447636		Department of Medicine	2020-03-13	Kassela et al (https://dx.doi.org/10.1101/2020.06.03.20121236)
Greece/204_33112/2020	EPI_ISL_437877	Laboratory of Microbiology, Medical School, National and Kapodistrian University of Athens	Laboratory of Biology, Department of Medicine, Democritus University of Thrace	2020-03-12	Kassela K. et al (https://dx.doi.org/10.1101/2020.06.03.20121236)
Greece/207_33548/2020	EPI_ISL_447639		Department of Medicine	2020-03-14	Kassela et al (https://dx.doi.org/10.1101/2020.06.03.20121236)
Greece/208_33922/2020	EPI_ISL_437878	Laboratory of Microbiology, Medical School, National and Kapodistrian University of Athens	Laboratory of Biology, Department of Medicine, Democritus University of Thrace	2020-03-14	Kassela K. et al (https://dx.doi.org/10.1101/2020.06.03.20121236)
Greece/223_34904/2020	EPI_ISL_437889	Laboratory of Microbiology, Medical School, National and Kapodistrian University of Athens	Laboratory of Biology, Department of Medicine, Democritus University of Thrace	2020-03-17	Kassela K. et al (https://dx.doi.org/10.1101/2020.06.03.20121236)
Greece/227_35969/2020	EPI_ISL_447644		Department of Medicine	2020-03-11	Kassela et al (https://dx.doi.org/10.1101/2020.06.03.20121236)
Greece/239_32178/2020	EPI_ISL_447646		Department of Medicine	2020-03-10	Kassela et al (https://dx.doi.org/10.1101/2020.06.03.20121236)
Greece/246_32206/2020	EPI_ISL_437905	Laboratory of Microbiology, Medical School, National and Kapodistrian University of Athens	Laboratory of Biology, Department of Medicine, Democritus University of Thrace	2020-03-10	Kassela K. et al (https://dx.doi.org/10.1101/2020.06.03.20121236)
Greece/250_32267/2020	EPI_ISL_447648		Department of Medicine	2020-03-10	Kassela et al (https://dx.doi.org/10.1101/2020.06.03.20121236)
Greece/254_32422/2020	EPI_ISL_447649		Department of Medicine	2020-03-11	Kassela et al (https://dx.doi.org/10.1101/2020.06.03.20121236)
Greece/259_31928/2020	EPI_ISL_447651		Department of Medicine	2020-03-09	Kassela et al (https://dx.doi.org/10.1101/2020.06.03.20121236)
Greece/262_32443/2020	EPI_ISL_447652		Department of Medicine	2020-03-11	Kassela et al (https://dx.doi.org/10.1101/2020.06.03.20121236)
Greece/33_36910/2020	EPI_ISL_437893	Laboratory of Microbiology, Medical School, National and Kapodistrian University of Athens	Laboratory of Biology, Department of Medicine, Democritus University of Thrace	2020-03-29	Kassela K. et al (https://dx.doi.org/10.1101/2020.06.03.20121236)
Greece/42_36236/2020	EPI_ISL_437899	Laboratory of Microbiology, Medical School, National and Kapodistrian University of Athens	Laboratory of Biology, Department of Medicine, Democritus University of Thrace	2020-03-23	Kassela K. et al (https://dx.doi.org/10.1101/2020.06.03.20121236)
Greece/44/2020	EPI_ISL_434465	Laboratory of Microbiology, Medical School, National and Kapodistrian University of Athens	Laboratory of Biology, Department of Medicine, Democritus University of Thrace	2020-03-23	Kassela K. et al (https://dx.doi.org/10.1101/2020.06.03.20121236)
Greece/46/2020	EPI_ISL_434463	Laboratory of Microbiology, Medical School, National and Kapodistrian University of Athens	Laboratory of Biology, Department of Medicine, Democritus University of Thrace	2020-03-23	Kassela K. et al (https://dx.doi.org/10.1101/2020.06.03.20121236)
Greece/48/2020	EPI_ISL_434456	Laboratory of Microbiology, Medical School, National and Kapodistrian University of Athens	Laboratory of Biology, Department of Medicine, Democritus University of Thrace	2020-03-22	Kassela K. et al (https://dx.doi.org/10.1101/2020.06.03.20121236)
Greece/50_36277/2020	EPI_ISL_447833		Department of Medicine	2020-03-23	Kassela et al (https://dx.doi.org/10.1101/2020.06.03.20121236)
Greece/51/2020	EPI_ISL_434468	Laboratory of Microbiology, Medical School, National and Kapodistrian University of Athens	Laboratory of Biology, Department of Medicine, Democritus University of Thrace	2020-03-26	Kassela K. et al (https://dx.doi.org/10.1101/2020.06.03.20121236)
Greece/55_36015/2020	EPI_ISL_447834		Department of Medicine	2020-03-23	Kassela et al (https://dx.doi.org/10.1101/2020.06.03.20121236)
Guam/GU_NHG_01/2020	EPI_ISL_444998	Naval Health Research Center	Naval Medical Research Center Biological Defense Research Directorate	2020-03-26	Logan Voegtly et al
Guam/GU_NHG_02/2020	EPI_ISL_444999	Naval Health Research Center	Naval Medical Research Center Biological Defense Research Directorate	2020-03-24	Logan Voegtly et al
Guam/GU_NHG_03/2020	EPI_ISL_445000	Naval Health Research Center	Naval Medical Research Center Biological Defense Research Directorate	2020-03-20	Logan Voegtly et al
Guangdong/20SF040/2020	EPI_ISL_403937	Guangdong Provincial Center for Diseases Control and Prevention; Guangdong Provincial Public Health	Department of Microbiology, Guangdong Provincial Center for Diseases Control and Prevention	2020-01-18	Min Kang et al
Guangdong/20SF2546/2020	EPI_ISL_428458	Guangdong Provincial Center for Diseases Control and Prevention;Guangdong Provincial Institute of Public Health	School of Public Health, The University of Hong Kong	2020-02-06	Bosheng Li et al
Guangdong/DG-S9-P0045/2020	EPI_ISL_413868	Guangdong Provincial Institution of Public Health, Guangdong Provincial Center for Disease Control and Prevention	Guangdong Provincial Institution of Public Health	2020-02-28	Jing Lu et al (https://dx.doi.org/10.1016/j.cell.2020.04.023)
Guangdong/GD2020086-P0021/2020	EPI_ISL_413862	Guangdong Provincial Institution of Public Health, Guangdong Provincial Center for Disease Control and Prevention	Guangdong Provincial Institution of Public Health	2020-02-01	Jing Lu et al (https://dx.doi.org/10.1016/j.cell.2020.04.023)

Guangdong/GD2020134-P0031/2020	EPI_ISL_413883	Guangdong Provincial Institution of Public Health, Guangdong Provincial Center for Disease Control and Prevention	Guangdong Provincial Institution of Public Health	2020-02-02	Jing Lu et al (https://dx.doi.org/10.1016/j.cell.2020.04.023)
Guangdong/SYSU-IHV/2020	EPI_ISL_444969	Guangzhou Eighth People's Hospital (Jiahe Sector)	Institute of Human Virology, Zhongshan School of Medicine, Sun Yat-sen University	2020-04-16	Junsong Zhang et al
Guangdong/SZ-N59-P0049/2020	EPI_ISL_413872	Guangdong Provincial Institution of Public Health, Guangdong Provincial Center for Disease Control and Prevention	Guangdong Provincial Institution of Public Health	2020-02-28	Jing Lu et al (https://dx.doi.org/10.1016/j.cell.2020.04.023)
Guangzhou/GZMU0054/2020	EPI_ISL_429098	The First Affiliated Hospital of Guangzhou Medical University	BGI-shenzhen & The First Affiliated Hospital of Guangzhou Medical University	2020-01-29	Yanqun Wang et al
Guangzhou/GZMU0078/2020	EPI_ISL_457690	The First Affiliated Hospital of Guangzhou Medical University, Guangzhou, China	BGI-shenzhen & The First Affiliated Hospital of Guangzhou Medical University	2020-02-11	Yanqun Wang et al
Guangzhou/GZMU0101/2020	EPI_ISL_457697	The First Affiliated Hospital of Guangzhou Medical University, Guangzhou, China	BGI-shenzhen & The First Affiliated Hospital of Guangzhou Medical University	2020-02-20	Yanqun Wang et al
Guangzhou/GZMU0117/2020	EPI_ISL_457698	The First Affiliated Hospital of Guangzhou Medical University, Guangzhou, China	BGI-shenzhen & The First Affiliated Hospital of Guangzhou Medical University	2020-02-26	Yanqun Wang et al
Hangzhou/HZ162/2020	EPI_ISL_418508	Hangzhou Center for Disease Control and Prevention	Inspection Center of Hangzhou Center for Disease Control and Prevention	2020-01-23	Yu hua et al
Hangzhou/HZ48/2020	EPI_ISL_418441	Hangzhou Center for Disease Control and Prevention	Inspection Center of Hangzhou Center for Disease Control and Prevention	2020-01-21	Yu hua et al
Hangzhou/HZ49/2020	EPI_ISL_418442	Hangzhou Center for Disease Control and Prevention	Inspection Center of Hangzhou Center for Disease Control and Prevention	2020-01-21	Yu hua et al
Hangzhou/HZ638/2020	EPI_ISL_418515	Hangzhou Center for Disease Control and Prevention	Inspection Center of Hangzhou Center for Disease Control and Prevention	2020-01-25	Yu hua et al
Hangzhou/ZJU-08/2020	EPI_ISL_416473	State Key Laboratory for Diagnosis and Treatment of Infectious Diseases, National Clinical Research Center for Infectious Diseases, First Affiliated Hospital, Zhejiang University School of Medicine, Hangzhou, China 310003	State Key Laboratory for Diagnosis and Treatment of Infectious Diseases, National Clinical Research Center for Infectious Diseases, First Affiliated Hospital, Zhejiang University School of Medicine, Hangzhou, China 310003	2020-01-26	Hangping Yao et al (https://dx.doi.org/10.1056/NEJMoa2006100)
Hangzhou/ZJU-09/2020	EPI_ISL_416474	State Key Laboratory for Diagnosis and Treatment of Infectious Diseases, National Clinical Research Center for Infectious Diseases, First Affiliated Hospital, Zhejiang University School of Medicine, Hangzhou, China 310003	State Key Laboratory for Diagnosis and Treatment of Infectious Diseases, National Clinical Research Center for Infectious Diseases, First Affiliated Hospital, Zhejiang University School of Medicine, Hangzhou, China 310003	2020-01-28	Hangping Yao et al (https://dx.doi.org/10.1056/NEJMoa2006100)
Harbin/HRB-26/2020	EPI_ISL_459909	Zoonotic and Exotic infection Diseases Division, Harbin Veterinary Research Institute, CAAS	Zoonotic and Exotic infection Diseases Division, Harbin Veterinary Research Institute, CAAS	2020-03-02	Zhigao Bu et al
HongKong/HKPU102_2802/2020	EPI_ISL_420455	Department of Clinical Pathology, Pamela Youde Nethersole Eastern Hospital	Department of Health Technology and Informatics, Faculty of Health and Social Science, The Hong Kong Polytechnic University	2020-03-04	Kenneth Siu-Sing LEUNG et al (https://dx.doi.org/10.1101/2020.03.30.20045740)
HongKong/HKPU12_2201/2020	EPI_ISL_417179	Department of Pathology, Princess Margaret Hospital	Department of Health Technology and Informatics, Faculty of Health and Social Science, The Hong Kong Polytechnic University	2020-01-30	Kenneth Siu-Sing LEUNG et al (https://dx.doi.org/10.1101/2020.03.30.20045740)
HongKong/HKPU19_0402/2020	EPI_ISL_417181	Department of Pathology, United Christian Hospital	Department of Health Technology and Informatics, Faculty of Health and Social Science, The Hong Kong Polytechnic University	2020-02-05	Kenneth Siu-Sing LEUNG et al (https://dx.doi.org/10.1101/2020.03.30.20045740)
HongKong/HKPU2_1801/2020	EPI_ISL_417177	Department of Pathology, Princess Margaret Hospital	Department of Health Technology and Informatics, Faculty of Health and Social Science, The Hong Kong Polytechnic University	2020-01-23	Kenneth Siu-Sing LEUNG et al (https://dx.doi.org/10.1101/2020.03.30.20045740)
HongKong/HKPU20_3001/2020	EPI_ISL_417182	Department of Pathology, United Christian Hospital	Department of Health Technology and Informatics, Faculty of Health and Social Science, The Hong Kong Polytechnic University	2020-02-05	Kenneth Siu-Sing LEUNG et al (https://dx.doi.org/10.1101/2020.03.30.20045740)
HongKong/HKPU23_2601/2020	EPI_ISL_417183	Department of Clinical Pathology, Pamela Youde Nethersole Eastern Hospital	Department of Health Technology and Informatics, Faculty of Health and Social Science, The Hong Kong Polytechnic University	2020-01-30	Kenneth Siu-Sing LEUNG et al (https://dx.doi.org/10.1101/2020.03.30.20045740)
HongKong/HKPU29_0102/2020	EPI_ISL_417187	Department of Clinical Pathology, Pamela Youde Nethersole Eastern Hospital	Department of Health Technology and Informatics, Faculty of Health and Social Science, The Hong Kong Polytechnic University	2020-02-08	Kenneth Siu-Sing LEUNG et al (https://dx.doi.org/10.1101/2020.03.30.20045740)
HongKong/HKPU30_2901/2020	EPI_ISL_417188	Department of Clinical Pathology, Pamela Youde Nethersole Eastern Hospital	Department of Health Technology and Informatics, Faculty of Health and Social Science, The Hong Kong Polytechnic University	2020-02-08	Kenneth Siu-Sing LEUNG et al (https://dx.doi.org/10.1101/2020.03.30.20045740)
HongKong/HKPU32_0402/2020	EPI_ISL_417193	Department of Clinical Pathology, Pamela Youde Nethersole Eastern Hospital	Department of Health Technology and Informatics, Faculty of Health and Social Science, The Hong Kong Polytechnic University	2020-02-09	Kenneth Siu-Sing LEUNG et al (https://dx.doi.org/10.1101/2020.03.30.20045740)
HongKong/HKPU33_0202/2020	EPI_ISL_417195	Department of Clinical Pathology, Pamela Youde Nethersole Eastern Hospital	Department of Health Technology and Informatics, Faculty of Health and Social Science, The Hong Kong Polytechnic University	2020-02-09	Kenneth Siu-Sing LEUNG et al (https://dx.doi.org/10.1101/2020.03.30.20045740)
HongKong/HKPU34_3001/2020	EPI_ISL_417197	Department of Clinical Pathology, Pamela Youde Nethersole Eastern Hospital	Department of Health Technology and Informatics, Faculty of Health and Social Science, The Hong Kong Polytechnic University	2020-02-09	Kenneth Siu-Sing LEUNG et al (https://dx.doi.org/10.1101/2020.03.30.20045740)
HongKong/HKPU35_0402/2020	EPI_ISL_417199	Department of Clinical Pathology, Pamela Youde Nethersole Eastern Hospital	Department of Health Technology and Informatics, Faculty of Health and Social Science, The Hong Kong Polytechnic University	2020-02-09	Chong-Yee YAU et al (https://dx.doi.org/10.1101/2020.03.30.20045740)
HongKong/HKPU38-3001/2020	EPI_ISL_419213	Department of Clinical Pathology, Pamela Youde Nethersole Eastern Hospital	Department of Health Technology and Informatics, Faculty of Health and Social Science, The Hong Kong Polytechnic University	2020-02-09	Kenneth Siu-Sing LEUNG et al (https://dx.doi.org/10.1101/2020.03.30.20045740)
HongKong/HKPU40-2801/2020	EPI_ISL_419215	Department of Clinical Pathology, Pamela Youde Nethersole Eastern Hospital	Department of Health Technology and Informatics, Faculty of Health and Social Science, The Hong Kong Polytechnic University	2020-02-10	Kenneth Siu-Sing LEUNG et al (https://dx.doi.org/10.1101/2020.03.30.20045740)
HongKong/HKPU42-0302/2020	EPI_ISL_419217	Department of Pathology, Princess Margaret Hospital	Department of Health Technology and Informatics, Faculty of Health and Social Science, The Hong Kong Polytechnic University	2020-02-10	Kenneth Siu-Sing LEUNG et al (https://dx.doi.org/10.1101/2020.03.30.20045740)
HongKong/HKPU54-0302/2020	EPI_ISL_419226	Department of Clinical Pathology, Pamela Youde Nethersole Eastern Hospital	Department of Health Technology and Informatics, Faculty of Health and Social Science, The Hong Kong Polytechnic University	2020-02-13	Kenneth Siu-Sing LEUNG et al (https://dx.doi.org/10.1101/2020.03.30.20045740)
HongKong/HKPU57-0702/2020	EPI_ISL_419227	Department of Clinical Pathology, Pamela Youde Nethersole Eastern Hospital	Department of Health Technology and Informatics, Faculty of Health and Social Science, The Hong Kong Polytechnic University	2020-02-15	Kenneth Siu-Sing LEUNG et al (https://dx.doi.org/10.1101/2020.03.30.20045740)
HongKong/HKPU6_2101/2020	EPI_ISL_417178	Department of Pathology, Princess Margaret Hospital	Department of Health Technology and Informatics, Faculty of Health and Social Science, The Hong Kong Polytechnic University	2020-01-25	Kenneth Siu-Sing LEUNG et al (https://dx.doi.org/10.1101/2020.03.30.20045740)
HongKong/HKPU60-0802/2020	EPI_ISL_419228	Department of Clinical Pathology, Pamela Youde Nethersole Eastern Hospital	Department of Health Technology and Informatics, Faculty of Health and Social Science, The Hong Kong Polytechnic University	2020-02-17	Kenneth Siu-Sing LEUNG et al (https://dx.doi.org/10.1101/2020.03.30.20045740)
HongKong/HKPU61-0202/2020	EPI_ISL_419229	Department of Clinical Pathology, Pamela Youde Nethersole Eastern Hospital	Department of Health Technology and Informatics, Faculty of Health and Social Science, The Hong Kong Polytechnic University	2020-02-17	Kenneth Siu-Sing LEUNG et al (https://dx.doi.org/10.1101/2020.03.30.20045740)
HongKong/HKPU66-2501/2020	EPI_ISL_419242	Department of Clinical Pathology, Tuen Mun Hospital	Department of Health Technology and Informatics, Faculty of Health and Social Science, The Hong Kong Polytechnic University	2020-02-23	Kenneth Siu-Sing LEUNG et al (https://dx.doi.org/10.1101/2020.03.30.20045740)
HongKong/HKPU70-1302/2020	EPI_ISL_419243	Department of Clinical Pathology, Pamela Youde Nethersole Eastern Hospital	Department of Health Technology and Informatics, Faculty of Health and Social Science, The Hong Kong Polytechnic University	2020-02-22	Kenneth Siu-Sing LEUNG et al (https://dx.doi.org/10.1101/2020.03.30.20045740)
HongKong/HKPU73-0802/2020	EPI_ISL_419244	Department of Clinical Pathology, Pamela Youde Nethersole Eastern Hospital	Department of Health Technology and Informatics, Faculty of Health and Social Science, The Hong Kong Polytechnic University	2020-02-22	Kenneth Siu-Sing LEUNG et al (https://dx.doi.org/10.1101/2020.03.30.20045740)
HongKong/HKPU74-1302/2020	EPI_ISL_419245	Department of Clinical Pathology, Pamela Youde Nethersole Eastern Hospital	Department of Health Technology and Informatics, Faculty of Health and Social Science, The Hong Kong Polytechnic University	2020-02-22	Kenneth Siu-Sing LEUNG et al (https://dx.doi.org/10.1101/2020.03.30.20045740)
HongKong/HKPU84-2502/2020	EPI_ISL_419248	Department of Clinical Pathology, Pamela Youde Nethersole Eastern Hospital	Department of Health Technology and Informatics, Faculty of Health and Social Science, The Hong Kong Polytechnic University	2020-02-24	Kenneth Siu-Sing LEUNG et al (https://dx.doi.org/10.1101/2020.03.30.20045740)
HongKong/HKPU86-1802/2020	EPI_ISL_419249	Department of Clinical Pathology, Pamela Youde Nethersole Eastern Hospital	Department of Health Technology and Informatics, Faculty of Health and Social Science, The Hong Kong Polytechnic University	2020-02-25	Kenneth Siu-Sing LEUNG et al (https://dx.doi.org/10.1101/2020.03.30.20045740)
HongKong/HKPU89-2502/2020	EPI_ISL_419250	Department of Clinical Pathology, Pamela Youde Nethersole Eastern Hospital	Department of Health Technology and Informatics, Faculty of Health and Social Science, The Hong Kong Polytechnic University	2020-02-26	Kenneth Siu-Sing LEUNG et al (https://dx.doi.org/10.1101/2020.03.30.20045740)
HongKong/HKPU91-2002/2020	EPI_ISL_419252	Department of Clinical Pathology, Pamela Youde Nethersole Eastern Hospital	Department of Health Technology and Informatics, Faculty of Health and Social Science, The Hong Kong Polytechnic University	2020-02-26	Kenneth Siu-Sing LEUNG et al (https://dx.doi.org/10.1101/2020.03.30.20045740)
HongKong/HKPU92-1302/2020	EPI_ISL_419253	Department of Pathology, United Christian Hospital	Department of Health Technology and Informatics, Faculty of Health and Social Science, The Hong Kong Polytechnic University	2020-02-26	Kenneth Siu-Sing LEUNG et al (https://dx.doi.org/10.1101/2020.03.30.20045740)
HongKong/HKSH0003/2020	EPI_ISL_430018	Molecular Pathology Division, Department of Pathology, Hong Kong Sanatorium & Hospital	Molecular Pathology Division, Department of Pathology, Hong Kong Sanatorium & Hospital	2020-03-14	Chun Hang AU et al
HongKong/HKSH0004/2020	EPI_ISL_430063	Molecular Pathology Division, Department of Pathology, Hong Kong Sanatorium & Hospital	Molecular Pathology Division, Department of Pathology, Hong Kong Sanatorium & Hospital	2020-03-14	Chun Hang AU et al
HongKong/HKSH0007/2020	EPI_ISL_451957	Molecular Pathology Division, Department of Pathology, Hong Kong Sanatorium & Hospital	Molecular Pathology Division, Department of Pathology, Hong Kong Sanatorium & Hospital	2020-04-01	Chun Hang AU et al
HongKong/HKU-902a/2020	EPI_ISL_434563		Microbiology	2020-01-23	To et al
HongKong/HKU-902b/2020	EPI_ISL_434564			2020-01-24	To et al
HongKong/HKU-903a/2020	EPI_ISL_434565		Microbiology	2020-01-24	To et al
HongKong/HKU-903b/2020	EPI_ISL_434566		Microbiology	2020-01-27	To et al
HongKong/HKU-905a/2020	EPI_ISL_450408		Microbiology	2020-01-24	To et al
HongKong/HKU-908a/2020	EPI_ISL_434569		Microbiology	2020-01-27	To et al
HongKong/HKU-908b/2020	EPI_ISL_434570		Microbiology	2020-01-29	To et al
HongKong/HKU-911a/2020	EPI_ISL_450409		Microbiology	2020-01-30	To et al
HongKong/VB20017970-2/2020	EPI_ISL_417064	Prince of Wales Hospital	Hong Kong Department of Health	2020-01-21	Alan K.L. Tsang et al
HongKong/VB20024950-2/2020	EPI_ISL_412029	Hong Kong Department of Health	The University of Hong Kong	2020-01-30	Dominic N.C. Tsang et al
HongKong/VM20002507-2/2020	EPI_ISL_414528	Hong Kong Department of Health	School of Public Health, The University of Hong Kong	2020-02-10	Dominic N.C. Tsang et al
HongKong/VM20002582-2/2020	EPI_ISL_414569	Hong Kong Department of Health	School of Public Health, The University of Hong Kong	2020-02-12	Dominic N.C. Tsang et al
HongKong/VM20002907/2020	EPI_ISL_414517	Hong Kong Department of Health	School of Public Health, The University of Hong Kong	2020-02-25	Dominic N.C. Tsang et al
HongKong/VM20009579/2020	EPI_ISL_450404		School of Public Health	2020-03-16	Sit et al
HongKong/VM2003179/2020	EPI_ISL_450405		School of Public Health	2020-03-08	Sit et al
Hungary/MBL-002/2020	EPI_ISL_435420	Virological Research Group, Szentágothai Research Centre	Bioinformatics Research Group, Szentágothai Research Centre	2020-03-23	Péter Urbán et al
Hungary/MBL-003/2020	EPI_ISL_435421	Virological Research Group, Szentágothai Research Centre	Bioinformatics Research Group, Szentágothai Research Centre	2020-03-25	Péter Urbán et al
Hungary/MBL-464/2020	EPI_ISL_435422	Virological Research Group, Szentágothai Research Centre	Bioinformatics Research Group, Szentágothai Research Centre	2020-03-27	Péter Urbán et al
Hungary/MBL-465/2020	EPI_ISL_435423	Virological Research Group, Szentágothai Research Centre	Bioinformatics Research Group, Szentágothai Research Centre	2020-03-27	Péter Urbán et al
Hungary/MBL-469/2020	EPI_ISL_435424	Virological Research Group, Szentágothai Research Centre	Bioinformatics Research Group, Szentágothai Research Centre	2020-03-27	Péter Urbán et al
Hungary/mbl2/2020	EPI_ISL_418183	Virological Research Group, Szentágothai Research Centre	Bioinformatics Research Group, Szentágothai Research Centre	2020-03-17	Péter Urbán et al
Hungary/mbl49/2020	EPI_ISL_416744	Virological Research Group, Szentágothai Research Centre	Bioinformatics Research Group, Szentágothai Research Centre	2020-03-20	Péter Urbán et al
Hungary/SRC-00055/2020	EPI_ISL_435419	Virological Research Group, Szentágothai Research Centre	Bioinformatics Research Group, Szentágothai Research Centre	2020-03-20	Péter Urbán et al
Hungary/SRC-00066/2020	EPI_ISL_435403	Virological Research Group, Szentágothai Research Centre	Bioinformatics Research Group, Szentágothai Research Centre	2020-03-20	Péter Urbán et al
Hungary/SRC-00067/2020	EPI_ISL_435404	Virological Research Group, Szentágothai Research Centre	Bioinformatics Research Group, Szentágothai Research Centre	2020-03-20	Péter Urbán et al
Hungary/SRC-00105w/2020	EPI_ISL_435426	Virological Research Group, Szentágothai Research Centre	Bioinformatics Research Group, Szentágothai Research Centre	2020-03-21	Péter Urbán et al
Hungary/SRC-00126/2020	EPI_ISL_435405	Virological Research Group, Szentágothai Research Centre	Bioinformatics Research Group, Szentágothai Research Centre	2020-03-22	Péter Urbán et al
Hungary/SRC-00175/2020	EPI_ISL_435406	Virological Research Group, Szentágothai Research Centre	Bioinformatics Research Group, Szentágothai Research Centre	2020-03-22	Péter Urbán et al
Hungary/SRC-00183/2020	EPI_ISL_435407	Virological Research Group, Szentágothai Research Centre	Bioinformatics Research Group, Szentágothai Research Centre	2020-03-22	Péter Urbán et al
Hungary/SRC-00186/2020	EPI_ISL_435408	Virological Research Group, Szentágothai Research Centre	Bioinformatics Research Group, Szentágothai Research Centre	2020-03-22	Péter Urbán et al
Hungary/SRC-00278w/2020	EPI_ISL_435427	Virological Research Group, Szentágothai Research Centre	Bioinformatics Research Group, Szentágothai Research Centre	2020-03-25	Péter Urbán et al
Hungary/SRC-00417/2020	EPI_ISL_435409	Virological Research Group, Szentágothai Research Centre	Bioinformatics Research Group, Szentágothai Research Centre	2020-03-25	Péter Urbán et al
Hungary/SRC-00419/2020	EPI_ISL_435410	Virological Research Group, Szentágothai Research Centre	Bioinformatics Research Group, Szentágothai Research Centre	2020-03-26	Péter Urbán et al
Hungary/SRC-00541/2020	EPI_ISL_435411	Virological Research Group, Szentágothai Research Centre	Bioinformatics Research Group, Szentágothai Research Centre	2020-03-27	Péter Urbán et al
Hungary/SRC-00572w/2020	EPI_ISL_435431	Virological Research Group, Szentágothai Research Centre	Bioinformatics Research Group, Szentágothai Research Centre	2020-03-25	Péter Urbán et al
Hungary/SRC-00620/2020	EPI_ISL_435416	Virological Research Group, Szentágothai Research Centre	Bioinformatics Research Group, Szentágothai Research Centre	2020-03-27	Péter Urbán et al
Hungary/SRC-00777/2020	EPI_ISL_435417	Virological Research Group, Szentágothai Research Centre	Bioinformatics Research Group, Szentágothai Research Centre	2020-03-30	Péter Urbán et al
Hungary/SRC-00792/2020	EPI_ISL_435412	Virological Research Group, Szentágothai Research Centre	Bioinformatics Research Group, Szentágothai Research Centre	2020-03-30	Péter Urbán et al
Hungary/SRC-00817/2020	EPI_ISL_435413	Virological Research Group, Szentágothai Research Centre	Bioinformatics Research Group, Szentágothai Research Centre	2020-03-30	Péter Urbán et al
Hungary/SRC-00827/2020	EPI_ISL_435414	Virological Research Group, Szentágothai Research Centre	Bioinformatics Research Group, Szentágothai Research Centre	2020-03-30	Péter Urbán et al
Hungary/SRC-00836/2020	EPI_ISL_435415	Virological Research Group, Szentágothai Research Centre	Bioinformatics Research Group, Szentágothai Research Centre	2020-03-30	Péter Urbán et al
Hungary/SRC-01136/2020	EPI_ISL_435418	Virological Research Group, Szentágothai Research Centre	Bioinformatics Research Group, Szentágothai Research Centre	2020-04-02	Péter Urbán et al
Hungary/SRC-02801w/2020	EPI_ISL_435428	Virological Research Group, Szentágothai Research Centre	Bioinformatics Research Group, Szentágothai Research Centre	2020-03-25	Péter Urbán et al
Hungary/SRC-03597w/2020	EPI_ISL_435429	Virological Research Group, Szentágothai Research Centre	Bioinformatics Research Group, Szentágothai Research Centre	2020-03-28	Péter Urbán et al

Hungary/SRC-03670w/2020	EPI_ISL_435430	Virological Research Group, Szentágothai Research Centre	Bioinformatics Research Group, Szentágothai Research Centre	2020-03-29	Péter Urbán et al
Hungary/SRC-1788lc/2020	EPI_ISL_435425	Virological Research Group, Szentágothai Research Centre	Bioinformatics Research Group, Szentágothai Research Centre	2020-03-19	Péter Urbán et al
Iceland/138/2020	EPI_ISL_417720	The National University Hospital of Iceland	deCODE genetics	2020-03-12	Daniel F Gudbjartsson et al (https://dx.doi.org/10.1056/NEJMoa2006100)
Iceland/222/2020	EPI_ISL_417837	The National University Hospital of Iceland	deCODE genetics	2020-03-16	Daniel F Gudbjartsson et al (https://dx.doi.org/10.1056/NEJMoa2006100)
Iceland/228/2020	EPI_ISL_417556	The National University Hospital of Iceland	deCODE genetics	2020-03-17	Daniel F Gudbjartsson et al (https://dx.doi.org/10.1056/NEJMoa2006100)
Iceland/291/2020	EPI_ISL_417620	The National University Hospital of Iceland	deCODE genetics	2020-03-18	Daniel F Gudbjartsson et al (https://dx.doi.org/10.1056/NEJMoa2006100)
Iceland/296/2020	EPI_ISL_417625	The National University Hospital of Iceland	deCODE genetics	2020-03-18	Daniel F Gudbjartsson et al (https://dx.doi.org/10.1056/NEJMoa2006100)
Iceland/299/2020	EPI_ISL_417628	The National University Hospital of Iceland	deCODE genetics	2020-03-18	Daniel F Gudbjartsson et al (https://dx.doi.org/10.1056/NEJMoa2006100)
Iceland/320/2020	EPI_ISL_417649	The National University Hospital of Iceland	deCODE genetics	2020-03-18	Daniel F Gudbjartsson et al (https://dx.doi.org/10.1056/NEJMoa2006100)
Iceland/353/2020	EPI_ISL_424377	deCODE genetics	deCODE genetics	2020-03-18	Daniel F Gudbjartsson et al (https://dx.doi.org/10.1056/NEJMoa2006100)
Iceland/380/2020	EPI_ISL_424404	The National University Hospital of Iceland	deCODE genetics	2020-03-19	Daniel F Gudbjartsson et al (https://dx.doi.org/10.1056/NEJMoa2006100)
Iceland/386/2020	EPI_ISL_424410	The National University Hospital of Iceland	deCODE genetics	2020-03-19	Daniel F Gudbjartsson et al (https://dx.doi.org/10.1056/NEJMoa2006100)
Iceland/389/2020	EPI_ISL_424413	The National University Hospital of Iceland	deCODE genetics	2020-03-19	Daniel F Gudbjartsson et al (https://dx.doi.org/10.1056/NEJMoa2006100)
Iceland/418/2020	EPI_ISL_424442	The National University Hospital of Iceland	deCODE genetics	2020-03-19	Daniel F Gudbjartsson et al (https://dx.doi.org/10.1056/NEJMoa2006100)
Iceland/436/2020	EPI_ISL_424460	The National University Hospital of Iceland	deCODE genetics	2020-03-20	Daniel F Gudbjartsson et al (https://dx.doi.org/10.1056/NEJMoa2006100)
Iceland/451/2020	EPI_ISL_424475	The National University Hospital of Iceland	deCODE genetics	2020-03-20	Daniel F Gudbjartsson et al (https://dx.doi.org/10.1056/NEJMoa2006100)
Iceland/47/2020	EPI_ISL_417683	The National University Hospital of Iceland	deCODE genetics	2020-03-05	Daniel F Gudbjartsson et al (https://dx.doi.org/10.1056/NEJMoa2006100)
Iceland/472/2020	EPI_ISL_424495	The National University Hospital of Iceland	deCODE genetics	2020-03-20	Daniel F Gudbjartsson et al (https://dx.doi.org/10.1056/NEJMoa2006100)
Iceland/474/2020	EPI_ISL_424497	The National University Hospital of Iceland	deCODE genetics	2020-03-20	Daniel F Gudbjartsson et al (https://dx.doi.org/10.1056/NEJMoa2006100)
Iceland/485/2020	EPI_ISL_424508	The National University Hospital of Iceland	deCODE genetics	2020-03-20	Daniel F Gudbjartsson et al (https://dx.doi.org/10.1056/NEJMoa2006100)
Iceland/49/2020	EPI_ISL_417718	The National University Hospital of Iceland	deCODE genetics	2020-03-05	Daniel F Gudbjartsson et al (https://dx.doi.org/10.1056/NEJMoa2006100)
Iceland/73/2020	EPI_ISL_417861	The National University Hospital of Iceland	deCODE genetics	2020-03-09	Daniel F Gudbjartsson et al (https://dx.doi.org/10.1056/NEJMoa2006100)
Iceland/82/2020	EPI_ISL_417867	The National University Hospital of Iceland	deCODE genetics	2020-03-09	Daniel F Gudbjartsson et al (https://dx.doi.org/10.1056/NEJMoa2006100)
Iceland/83/2020	EPI_ISL_417868	The National University Hospital of Iceland	deCODE genetics	2020-03-09	Daniel F Gudbjartsson et al (https://dx.doi.org/10.1056/NEJMoa2006100)
Iceland/97/2020	EPI_ISL_417876	The National University Hospital of Iceland	deCODE genetics	2020-03-10	Daniel F Gudbjartsson et al (https://dx.doi.org/10.1056/NEJMoa2006100)
India/1-27/2020	EPI_ISL_413522	Indian Council of Medical Research - National Institute of Virology	National Influenza Center, Indian Council of Medical Research - National Institute of Virology	2020-01-27	Potdar V et al A (https://dx.doi.org/10.4103/ijmr.IJMR_663_20)
India/1073/2020	EPI_ISL_421662	National Influenza Center, Indian Council of Medical Research - National Institute of Virology	Indian Council of Medical Research-National Institute of Virology, Microbial Containment Complex	2020-03-10	Pragya D. Yadav et al A (http://www.ijmr.org.in/article.asp?issn=0971-5916;year=2020;volume=151;issue=2;spage=255;epage=260;aulast=Potdar)
India/1100/2020	EPI_ISL_421664	National Influenza Center, Indian Council of Medical Research - National Institute of Virology	Indian Council of Medical Research-National Institute of Virology, Microbial Containment Complex	2020-03-10	Pragya D. Yadav et al A (http://www.ijmr.org.in/article.asp?issn=0971-5916;year=2020;volume=151;issue=2;spage=255;epage=260;aulast=Potdar)
India/1111/2020	EPI_ISL_421666	National Influenza Center, Indian Council of Medical Research - National Institute of Virology	Indian Council of Medical Research-National Institute of Virology, Microbial Containment Complex	2020-03-10	Pragya D. Yadav et al A (http://www.ijmr.org.in/article.asp?issn=0971-5916;year=2020;volume=151;issue=2;spage=255;epage=260;aulast=Potdar)
India/1135/2020	EPI_ISL_424362	National Influenza Center, Indian Council of Medical Research - National Institute of Virology	Indian Council of Medical Research-National Institute of Virology, Microbial Containment Complex	2020-03-10	Pragya D. Yadav et al B (https://www.biorxiv.org/content/10.1101/2020.05.28.122143v1)
India/CCMB_C4-15/2020	EPI_ISL_447587	Tamil Nadu Veterinary and Animal Sciences University	CSIR-Centre for Cellular and Molecular Biology	2020-04-20	K Kaveri et al (https://dx.doi.org/10.1101/2020.05.31.126136)
India/CCMB_C9/2020	EPI_ISL_458038	King Institute of Preventive Medicine & Research	CSIR-Centre for Cellular and Molecular Biology	2020-04-29	K.Kaveri et al
India/CCMB_J009/2020	EPI_ISL_447557	CSIR-Centre for Cellular and Molecular Biology	CSIR-Centre for Cellular and Molecular Biology	2020-03-30	Payel Mukherjee et al (https://dx.doi.org/10.1101/2020.05.31.126136)
India/CCMB_J057/2020	EPI_ISL_447563	CSIR-Centre for Cellular and Molecular Biology	CSIR-Centre for Cellular and Molecular Biology	2020-03-31	Sakshi Shambhavi et al A (https://dx.doi.org/10.1101/2020.05.31.126136)
India/CCMB_J067/2020	EPI_ISL_447561	CSIR-Centre for Cellular and Molecular Biology	CSIR-Centre for Cellular and Molecular Biology	2020-03-31	Shagufta Khan et al (https://dx.doi.org/10.1101/2020.05.31.126136)
India/CCMB_J207/2020	EPI_ISL_447570	CSIR-Centre for Cellular and Molecular Biology	CSIR-Centre for Cellular and Molecular Biology	2020-04-01	Lamuk Zaveri et al (https://dx.doi.org/10.1101/2020.05.31.126136)
India/CCMB_J220/2020	EPI_ISL_450331	CSIR-Centre for Cellular and Molecular Biology	CSIR-Centre for Cellular and Molecular Biology	2020-04-01	Tulasi Nagabandi et al (https://dx.doi.org/10.1101/2020.05.31.126136)
India/CCMB_J230/2020	EPI_ISL_447572	CSIR-Centre for Cellular and Molecular Biology	CSIR-Centre for Cellular and Molecular Biology	2020-04-01	Lamuk Zaveri et al (https://dx.doi.org/10.1101/2020.05.31.126136)
India/CCMB_J458/2020	EPI_ISL_447575	CSIR-Centre for Cellular and Molecular Biology	CSIR-Centre for Cellular and Molecular Biology	2020-04-02	Sofia Banu et al (https://dx.doi.org/10.1101/2020.05.31.126136)
India/CCMB_K272/2020	EPI_ISL_458070	CSIR-Centre for Cellular and Molecular Biology	CSIR-Centre for Cellular and Molecular Biology	2020-04-12	Sakshi Shambhavi et al B
India/CCMB_L044/2020	EPI_ISL_447864	CSIR-Centre for Cellular and Molecular Biology	CSIR-Centre for Cellular and Molecular Biology	2020-04-16	Payel Mukherjee et al (https://dx.doi.org/10.1101/2020.05.31.126136)
India/CCMB_O2_P1/2020	EPI_ISL_458075	CSIR-Centre for Cellular and Molecular Biology	CSIR-Centre for Cellular and Molecular Biology	2020-04-15	Dhiviya Vedagiri et al
India/GBRC104/2020	EPI_ISL_451149	M.P Shah Government Medocal college Jamnagar	Gujarat Biotechnology Research Centre	2020-05-07	Janvi Raval et al
India/GBRC122/2020	EPI_ISL_455019	Government Medical College, Vadodara	Gujarat Biotechnology Research Centre	2020-05-02	Ramesh Pandit et al
India/GBRC124/2020	EPI_ISL_455021	Government Medical College, Vadodara	Gujarat Biotechnology Research Centre	2020-05-02	Ankit Hinsu et al A
India/GBRC125/2020	EPI_ISL_455022	Government Medical College, Vadodara	Gujarat Biotechnology Research Centre	2020-05-02	Pritesh Sabara et al B
India/GBRC132/2020	EPI_ISL_458087	B.J. Medical College and Civil hospital	Gujarat Biotechnology Research Centre	2020-05-24	Ramesh Patel et al
India/GBRC138/2020	EPI_ISL_458096	B.J. Medical College and Civil hospital	Gujarat Biotechnology Research Centre	2020-05-24	Dinesh Kumar et al
India/GBRC141b/2020	EPI_ISL_458101	B.J. Medical College and Civil hospital	Gujarat Biotechnology Research Centre	2020-05-24	Dhaval Vaghela et al
India/GBRC143/2020	EPI_ISL_458103	Gujarat Biotechnology Research Centre	Gujarat Biotechnology Research Centre	2020-05-05	Ramesh Pandit et al
India/GBRC151b/2020	EPI_ISL_461479	Government Medical College, Vadodara	Gujarat Biotechnology Research Centre	2020-05-02	Neelam Nathani et al
India/GBRC159b/2020	EPI_ISL_461490	B.J. Medical College and Civil hospital	Gujarat Biotechnology Research Centre	2020-05-27	Afzal Ansari et al
India/GBRC162b/2020	EPI_ISL_461496	B.J. Medical College and Civil hospital	Gujarat Biotechnology Research Centre	2020-05-27	Dipeshwari Shewale et al
India/GBRC169a/2020	EPI_ISL_467029	GMERS Medical College and Hospital, Gandhinagar	Gujarat Biotechnology Research Centre	2020-06-05	Seema Bhatt et al
India/GBRC169b/2020	EPI_ISL_467030	GMERS Medical College and Hospital, Gandhinagar	Gujarat Biotechnology Research Centre	2020-06-05	Gaurishankar Shrimali et al
India/GBRC170a/2020	EPI_ISL_467031	GMERS Medical College and Hospital, Gandhinagar	Gujarat Biotechnology Research Centre	2020-06-05	Bhavesh Modi et al
India/GBRC170b/2020	EPI_ISL_467032	GMERS Medical College and Hospital, Gandhinagar	Gujarat Biotechnology Research Centre	2020-06-05	Bharti Rajani et al
India/GBRC171/2020	EPI_ISL_467033	GMERS Medical College and Hospital, Gandhinagar	Gujarat Biotechnology Research Centre	2020-06-05	Tejas Shah et al
India/GBRC173a/2020	EPI_ISL_467035	GMERS Medical College and Hospital, Gandhinagar	Gujarat Biotechnology Research Centre	2020-06-05	Pritesh Sabara et al B
India/GBRC173b/2020	EPI_ISL_467036	GMERS Medical College and Hospital, Gandhinagar	Gujarat Biotechnology Research Centre	2020-06-05	Apurvasinh Puvar et al B
India/GBRC174a/2020	EPI_ISL_467037	GMERS Medical College and Hospital, Gandhinagar	Gujarat Biotechnology Research Centre	2020-06-05	Janvi Raval et al
India/GBRC174b/2020	EPI_ISL_467038	GMERS Medical College and Hospital, Gandhinagar	Gujarat Biotechnology Research Centre	2020-06-05	Zarna Patel et al
India/GBRC177b/2020	EPI_ISL_467042	B.J. Medical College and Civil hospital	Gujarat Biotechnology Research Centre	2020-06-03	Pinal Trivedi et al
India/GBRC178b/2020	EPI_ISL_467044	B.J. Medical College and Civil hospital	Gujarat Biotechnology Research Centre	2020-06-03	Nidhi Patel et al
India/GBRC179a/2020	EPI_ISL_467045	B.J. Medical College and Civil hospital	Gujarat Biotechnology Research Centre	2020-06-03	Nitin Savaliya et al
India/GBRC179b/2020	EPI_ISL_467046	B.J. Medical College and Civil hospital	Gujarat Biotechnology Research Centre	2020-06-03	Raghawendra Kumar et al
India/GBRC180a/2020	EPI_ISL_467047	B.J. Medical College and Civil hospital	Gujarat Biotechnology Research Centre	2020-06-03	Dinesh Kumar et al
India/GBRC180b/2020	EPI_ISL_467048	B.J. Medical College and Civil hospital	Gujarat Biotechnology Research Centre	2020-06-03	Zuber Saiyed et al
India/GBRC181b/2020	EPI_ISL_467050	B.J. Medical College and Civil hospital	Gujarat Biotechnology Research Centre	2020-06-03	Labdhi Pandya et al
India/GBRC182b/2020	EPI_ISL_467052	B.J. Medical College and Civil hospital	Gujarat Biotechnology Research Centre	2020-06-03	Pranay Shah et al
India/GBRC183b/2020	EPI_ISL_467054	B.J. Medical College and Civil hospital	Gujarat Biotechnology Research Centre	2020-06-03	Nirav Mungalpara et al
India/GBRC185a/2020	EPI_ISL_469025	B.J. Medical College and Civil hospital	Gujarat Biotechnology Research Centre	2020-06-03	Ankit Hinsu et al A
India/GBRC185b/2020	EPI_ISL_469026	B.J. Medical College and Civil hospital	Gujarat Biotechnology Research Centre	2020-06-03	Pritesh Sabara et al B
India/GBRC186b/2020	EPI_ISL_469028	B.J. Medical College and Civil hospital	Gujarat Biotechnology Research Centre	2020-06-03	Janvi Raval et al
India/GBRC187a/2020	EPI_ISL_469029	Government Medical College, Vadodara	Gujarat Biotechnology Research Centre	2020-06-05	Zarna Patel et al
India/GBRC187b/2020	EPI_ISL_469030	Government Medical College, Vadodara	Gujarat Biotechnology Research Centre	2020-06-05	Monika Gandhi et al A
India/GBRC19/2020	EPI_ISL_437448	B.J. Medical College and Civil hospital	Gujarat Biotechnology Research Centre	2020-04-26	Geeta Vaghela et al (https://www.biorxiv.org/content/10.1101/2020.05.28.122143v1)
India/GBRC192/2020	EPI_ISL_469036	Government Medical College, Vadodara	Gujarat Biotechnology Research Centre	2020-06-05	Dinesh Kumar et al
India/GBRC195a/2020	EPI_ISL_469039	GMERS Medical College & Hospital	Gujarat Biotechnology Research Centre	2020-06-08	Labdhi Pandya et al
India/GBRC195b/2020	EPI_ISL_469040	GMERS Medical College & Hospital	Gujarat Biotechnology Research Centre	2020-06-08	Snehal Bagatharia et al
India/GBRC197/2020	EPI_ISL_469042	GMERS Medical College & Hospital	Gujarat Biotechnology Research Centre	2020-06-08	Neena Doshi et al
India/GBRC201a/2020	EPI_ISL_469046	Dr. N. D. Desai Medical College & Hospital	Gujarat Biotechnology Research Centre	2020-06-08	Tejas Shah et al
India/GBRC201b/2020	EPI_ISL_469047	Dr. N. D. Desai Medical College & Hospital	Gujarat Biotechnology Research Centre	2020-06-08	Ankit Hinsu et al A
India/GBRC202/2020	EPI_ISL_469048	Banas Medical College and Research Institute	Gujarat Biotechnology Research Centre	2020-06-05	Radhika Khara et al
India/GBRC24a/2020	EPI_ISL_437453	B.J. Medical College and Civil hospital	Gujarat Biotechnology Research Centre	2020-04-26	Nidhi Sood et al (https://www.biorxiv.org/content/10.1101/2020.05.28.122143v1)
India/GBRC30/2020	EPI_ISL_444461	B.J. Medical College and Civil hospital	Gujarat Biotechnology Research Centre	2020-04-29	Ankit Hinsu et al B (https://www.biorxiv.org/content/10.1101/2020.05.28.122143v1)
India/GBRC31/2020	EPI_ISL_444462	B.J. Medical College and Civil hospital	Gujarat Biotechnology Research Centre	2020-04-29	Pritesh Sabara et al A (https://www.biorxiv.org/content/10.1101/2020.05.28.122143v1)
India/GBRC44/2020	EPI_ISL_444475	B.J. Medical College and Civil hospital	Gujarat Biotechnology Research Centre	2020-04-29	Disha Patel et al (https://www.biorxiv.org/content/10.1101/2020.05.28.122143v1)
India/GBRC62/2020	EPI_ISL_447037	B.J. Medical College and Civil hospital	Gujarat Biotechnology Research Centre	2020-05-03	Monika Gandhi et al B (https://www.biorxiv.org/content/10.1101/2020.05.28.122143v1)
India/GBRC64/2020	EPI_ISL_447039	B.J. Medical College and Civil hospital	Gujarat Biotechnology Research Centre	2020-05-03	Maharshi Pandya et al (https://www.biorxiv.org/content/10.1101/2020.05.28.122143v1)
India/GBRC82a/2020	EPI_ISL_447542	Gujarat Biotechnology Research Centre	Gujarat Biotechnology Research Centre	2020-05-05	Ankit Hinsu et al B (https://www.biorxiv.org/content/10.1101/2020.05.28.122143v1)
India/GBRC83/2020	EPI_ISL_447544	Gujarat Biotechnology Research Centre	Gujarat Biotechnology Research Centre	2020-05-05	Apurvasinh Puvar et al A (https://www.biorxiv.org/content/10.1101/2020.05.28.122143v1)
India/GBRC85/2020	EPI_ISL_447546	Gujarat Biotechnology Research Centre	Gujarat Biotechnology Research Centre	2020-05-05	Monika Gandhi et al B (https://www.biorxiv.org/content/10.1101/2020.05.28.122143v1)
India/GMC-KP1125/2020	EPI_ISL_437626	Department of Microbiology,Gandhi Medical College and Hospital	Department of Veterinary Biotechnalogy, College of Veterinary Science, Rajendranagar, PV Narsimha Rao Telengana Veterinary University	2020-03-24	Kalyani Putty et al (https://www.biorxiv.org/content/10.1101/2020.05.28.122143v1)
India/GMC-RK100/2020	EPI_ISL_431101	Department of Microbiology,Gandhi Medical College and Hospital	Virus Research Laboratory, Department of Zoology, Osmania University,Hyderabad,India	2020-03-01	Muttineni Radhakrishna et al A (https://www.biorxiv.org/content/10.1101/2020.05.28.122143v1)
India/GMC-RK1090/2020	EPI_ISL_438139	Department of Microbiology,Gandhi Medical College and Hospital,Hyderabad	Virus Research Laboratory, Department of Zoology, Osmania University, Hyderabad, India	2020-03-25	Muttineni Radhakrishna et al B
India/GMC-RR1191/2020	EPI_ISL_438138	Department of Microbiology,Gandhi Medical College and Hospital	Department of Microbiology, Gandhi Medical College and Hospital Secendrabad, Hyderbad, India	2020-03-25	Raja Rao Mesipogu et al
India/ILSCV14289/2020	EPI_ISL_463030	Institute of Life Sciences, Bhubaneswar	Immunogenomics lab, Institute of Life Sciences, Bhubaneswar	2020-05-11	Sunil Raghav et al
India/ILSCV16495/2020	EPI_ISL_463046	Institute of Life Sciences, Bhubaneswar	Immunogenomics lab, Institute of Life Sciences, Bhubaneswar	2020-05-12	Sunil Raghav et al
India/ILSCV16510/2020	EPI_ISL_463050	Institute of Life Sciences, Bhubaneswar	Immunogenomics lab, Institute of Life Sciences, Bhubaneswar	2020-05-12	Sunil Raghav et al
India/ILSCV17568/2020	EPI_ISL_463056	Institute of Life Sciences, Bhubaneswar	Immunogenomics lab, Institute of Life Sciences, Bhubaneswar	2020-05-12	Sunil Raghav et al
India/ILSCV24917/2020	EPI_ISL_463088	Institute of Life Sciences, Bhubaneswar	Immunogenomics lab, Institute of Life Sciences, Bhubaneswar	2020-05-18	Sunil Raghav et al
India/MaxCov0030_CSIR-IGIB/2020	EPI_ISL_459932	Devki Devi Foundation, a unit of Max Healthcare	CSIR-IGIB/Max	2020-05-13	Rajesh Pandey# et al
India/NCDC-01710/2020	EPI_ISL_435070	National Centre for Disease control (NCDC), CSIR-Institute of Genomics and Integrative Biology (CSIR-IGIB)	NCDC/CSIR-IGIB	2020-03-18	Pramod Kumar et al (https://dx.doi.org/10.1101/2020.06.04.128751)
India/NCDC-01744/2020	EPI_ISL_435069	National Centre for Disease control (NCDC), CSIR-Institute of Genomics and Integrative Biology (CSIR-IGIB)	NCDC/CSIR-IGIB	2020-03-18	Pramod Kumar et al (https://dx.doi.org/10.1101/2020.06.04.128751)
India/NCDC-01757/2020	EPI_ISL_435068	National Centre for Disease control (NCDC), CSIR-Institute of Genomics and Integrative Biology (CSIR-IGIB)	NCDC/CSIR-IGIB	2020-03-18	Pramod Kumar et al (https://dx.doi.org/10.1101/2020.06.04.128751)
India/NCDC-02309/2020	EPI_ISL_435084	National Centre for Disease control (NCDC), CSIR-Institute of Genomics and Integrative Biology (CSIR-IGIB)	NCDC/CSIR-IGIB	2020-03-29	Pramod Kumar et al (https://dx.doi.org/10.1101/2020.06.04.128751)
India/NCDC-02310/2020	EPI_ISL_435085	National Centre for Disease control (NCDC), CSIR-Institute of Genomics and Integrative Biology (CSIR-IGIB)	NCDC/CSIR-IGIB	2020-03-29	Pramod Kumar et al (https://dx.doi.org/10.1101/2020.06.04.128751)
India/NCDC-02312/2020	EPI_ISL_435093	National Centre for Disease control (NCDC), CSIR-Institute of Genomics and Integrative Biology (CSIR-IGIB)	NCDC/CSIR-IGIB	2020-03-29	Pramod Kumar et al (https://dx.doi.org/10.1101/2020.06.04.128751)
India/NCDC-02320/2020	EPI_ISL_435099	National Centre for Disease control (NCDC), CSIR-Institute of Genomics and Integrative Biology (CSIR-IGIB)	NCDC/CSIR-IGIB	2020-03-29	Pramod Kumar et al (https://dx.doi.org/10.1101/2020.06.04.128751)

India/NCDC-02322/2020	EPI_ISL_435082	National Centre for Disease control (NCDC), CSIR-Institute of Genomics and Integrative Biology (CSIR-IGIB)	NCDC/CSIR-IGIB	2020-03-29	Pramod Kumar et al (https://dx.doi.org/10.1101/2020.06.04.128751)
India/NCDC-02328/2020	EPI_ISL_435095	National Centre for Disease control (NCDC), CSIR-Institute of Genomics and Integrative Biology (CSIR-IGIB)	NCDC/CSIR-IGIB	2020-03-29	Pramod Kumar et al (https://dx.doi.org/10.1101/2020.06.04.128751)
India/NCDC-02336/2020	EPI_ISL_435088	National Centre for Disease control (NCDC), CSIR-Institute of Genomics and Integrative Biology (CSIR-IGIB)	NCDC/CSIR-IGIB	2020-03-29	Pramod Kumar et al (https://dx.doi.org/10.1101/2020.06.04.128751)
India/NCDC-02370/2020	EPI_ISL_435107	National Centre for Disease control (NCDC), CSIR-Institute of Genomics and Integrative Biology (CSIR-IGIB)	NCDC/CSIR-IGIB	2020-03-29	Pramod Kumar et al (https://dx.doi.org/10.1101/2020.06.04.128751)
India/NCDC-02415/2020	EPI_ISL_435111	National Centre for Disease control (NCDC), CSIR-Institute of Genomics and Integrative Biology (CSIR-IGIB)	NCDC/CSIR-IGIB	2020-03-30	Pramod Kumar et al (https://dx.doi.org/10.1101/2020.06.04.128751)
India/NCDC-2443/2020	EPI_ISL_436413	National Centre for Disease control (NCDC)	NCDC/CSIR-IGIB	2020-03-30	Pramod Kumar# et al (https://dx.doi.org/10.1101/2020.06.04.128751)
India/NCDC-2514/2020	EPI_ISL_436416	National Centre for Disease control (NCDC)	NCDC/CSIR-IGIB	2020-03-31	Pramod Kumar# et al (https://dx.doi.org/10.1101/2020.06.04.128751)
India/NCDC-2517/2020	EPI_ISL_436418	National Centre for Disease control (NCDC)	NCDC/CSIR-IGIB	2020-03-31	Pramod Kumar# et al (https://dx.doi.org/10.1101/2020.06.04.128751)
India/NCDC-2535/2020	EPI_ISL_436422	National Centre for Disease control (NCDC)	NCDC/CSIR-IGIB	2020-03-31	Pramod Kumar# et al (https://dx.doi.org/10.1101/2020.06.04.128751)
India/NCDC-3175/2020	EPI_ISL_436426	National Centre for Disease control (NCDC)	NCDC/CSIR-IGIB	2020-04-05	Pramod Kumar# et al (https://dx.doi.org/10.1101/2020.06.04.128751)
India/NCDC-3230/2020	EPI_ISL_436431	National Centre for Disease control (NCDC)	NCDC/CSIR-IGIB	2020-04-05	Pramod Kumar# et al (https://dx.doi.org/10.1101/2020.06.04.128751)
India/NCDC-3835/2020	EPI_ISL_436438	National Centre for Disease control (NCDC)	NCDC/CSIR-IGIB	2020-04-07	Pramod Kumar# et al (https://dx.doi.org/10.1101/2020.06.04.128751)
India/NGC-CDFD-03/2020	EPI_ISL_466841	National Genomics Core-Center for DNA Fingerprinting and Diagnostics	National Genomics Core- Center for DNA Fingerprinting and Diagnostics (NGC-CDFD)- DBT's PAN-INDIA-1000 Genome consortium	2020-05-10	Bala Pratyusha et al
India/NGC-CDFD-12/2020	EPI_ISL_466850	National Genomics Core-Center for DNA Fingerprinting and Diagnostics	National Genomics Core- Center for DNA Fingerprinting and Diagnostics (NGC-CDFD)- DBT's PAN-INDIA-1000 Genome consortium	2020-05-26	Bala Pratyusha et al
India/NGC-CDFD-13/2020	EPI_ISL_466851	National Genomics Core-Center for DNA Fingerprinting and Diagnostics	National Genomics Core- Center for DNA Fingerprinting and Diagnostics (NGC-CDFD)- DBT's PAN-INDIA-1000 Genome consortium	2020-05-27	Bala Pratyusha et al
India/NGC-CDFD-21/2020	EPI_ISL_466859	National Genomics Core-Center for DNA Fingerprinting and Diagnostics	National Genomics Core- Center for DNA Fingerprinting and Diagnostics (NGC-CDFD)- DBT's PAN-INDIA-1000 Genome consortium	2020-05-29	Bala Pratyusha et al
India/NIHSAD-2904-107/2020	EPI_ISL_452791	ICAR-National Institute of High Security Animal Diseases	ICAR-National Institute of High Security Animal Diseases	2020-04-27	Anamika Mishra et al
India/nimh-1071/2020	EPI_ISL_428487	District Surveillence Unit	Department of Neurovirology, National Institute of Mental Health and Neuroscience (NIMHANS)	2020-04-14	Chitra Pattabiraman et al (https://www.biorxiv.org/content/10.1101/2020.05.28.122143v1)
India/nimh-1598/2020	EPI_ISL_436157	District Surveillance Unit	Department of Neurovirology, National Institute of Mental Health and Neuroscience (NIMHANS)	2020-04-17	Chitra Pattabiraman et al (https://www.biorxiv.org/content/10.1101/2020.05.28.122143v1)
India/NIV-11855/2020	EPI_ISL_452198	NIV Influenza	NIV Influenza	2020-04-20	Potdar V et al B
India/NIV-12067/2020	EPI_ISL_452201	NIV Influenza	NIV Influenza	2020-04-20	Potdar V et al B
India/NIV-2810/2020	EPI_ISL_452202	NIV Influenza	NIV Influenza	2020-03-16	Potdar V et al B
India/NIV-QA-710/2020	EPI_ISL_454565	NIV Influenza	NIV Influenza	2020-03-31	Potdar V et al B
India/NIV-QC-796/2020	EPI_ISL_454567	NIV Influenza	NIV Influenza	2020-04-22	Potdar V et al B
India/RMRC23/2020	EPI_ISL_455774	REGIONAL VRDL,ICMR-RMRC BBSR	Immunogenomics lab, Institute of Life Sciences, Bhubaneswar	2020-04-04	Sunil Raghav et al
India/RMRC24/2020	EPI_ISL_455775	REGIONAL VRDL,ICMR-RMRC BBSR	Immunogenomics lab, Institute of Life Sciences, Bhubaneswar	2020-04-04	Sunil Raghav et al
India/RMRC30/2020	EPI_ISL_455778	REGIONAL VRDL,ICMR-RMRC BBSR	Immunogenomics lab, Institute of Life Sciences, Bhubaneswar	2020-04-04	Sunil Raghav et al
India/RMRC6/2020	EPI_ISL_455783	REGIONAL VRDL,ICMR-RMRC BBSR	Immunogenomics lab, Institute of Life Sciences, Bhubaneswar	2020-04-02	Sunil Raghav et al
India/RMRC7/2020	EPI_ISL_455784	REGIONAL VRDL,ICMR-RMRC BBSR	Immunogenomics lab, Institute of Life Sciences, Bhubaneswar	2020-04-02	Sunil Raghav et al
India/S31/2020	EPI_ISL_455660	ICMR-National Institute of Cholera and Enteric Diseases	National Institute of Biomedical Genomics	2020-05-01	Arindam Maitra et al
India/S4/2020	EPI_ISL_455640	ICMR-National Institute of Cholera and Enteric Diseases	National Institute of Biomedical Genomics	2020-03-27	Arindam Maitra et al
India/S48/2020	EPI_ISL_455668	ICMR-National Institute of Cholera and Enteric Diseases	National Institute of Biomedical Genomics	2020-05-05	Arindam Maitra et al
India/S49/2020	EPI_ISL_455669	ICMR-National Institute of Cholera and Enteric Diseases	National Institute of Biomedical Genomics	2020-05-03	Arindam Maitra et al
India/S50/2020	EPI_ISL_455670	ICMR-National Institute of Cholera and Enteric Diseases	National Institute of Biomedical Genomics	2020-05-03	Arindam Maitra et al
India/SMSCOV161/2020	EPI_ISL_454832	SMS Medical College, Jaipur	CSIR Institute of Genomics and Integrative Biology	2020-04-21	Sudhir Bhandari et al
India/THSTI-BAL_42/2020	EPI_ISL_454860	Translational Health Science and Technology Institute -ESIC medical college and hospital, Faridabad	THSTI Bioassay laboratory	2020-04-07	Saurabh Kumar et al
Indonesia/EJ-ITD1238Sp/2020	EPI_ISL_458079	Mitra Keluarga Hospital Kenjeran	Institute of Tropical Disease, Universitas Airlangga	2020-03-30	Aldise M Nastri et al
Indonesia/EJ-ITD1273NT/2020	EPI_ISL_458081	RSUD Bangil Pasuruan	Institute of Tropical Disease, Universitas Airlangga	2020-03-30	Jezzy R Dewantari et al
Indonesia/EJ-ITD3101NT/2020	EPI_ISL_458083	Adi Husada Undaan Hospital	Institute of Tropical Disease, Universitas Airlangga	2020-04-11	Rima R Prasetya et al
Indonesia/EJ-ITD3590NT/2020	EPI_ISL_437188	RSUD Dr. Soetomo	Institute of Tropical Disease, Universitas Airlangga	2020-04-14	Krisnoadi Rahardjo et al
Indonesia/EJ-ITD853Sp/2020	EPI_ISL_437187	Siloam Hospitals	Institute of Tropical Disease, Universitas Airlangga	2020-03-25	Kazufumi Shimizu et al
Indonesia/JKT-EIJK01/2020	EPI_ISL_437189	Pusat Pertamina Hospital	Eijkman Institute for Molecular Biology, Ministry of Research and Technology/National Agency for Research and Innovation	2020-03-26	Edison Johar et al
Indonesia/JKT-EIJK0141/2020	EPI_ISL_435281	Medistra Hospital Jakarta	Eijkman Institute for Molecular Biology, Ministry of Research and Technology/National Agency for Research and Innovation	2020-03-17	Edison Johar et al
Indonesia/JKT-EIJK02/2020	EPI_ISL_437190	RS Pondok Indah Hospital – Pondok Indah	Eijkman Institute for Molecular Biology, Ministry of Research and Technology/National Agency for Research and Innovation	2020-03-26	Edison Johar et al
Indonesia/JKT-EIJK03/2020	EPI_ISL_437191	RS Pondok Indah Hospital – Pondok Indah	Eijkman Institute for Molecular Biology, Ministry of Research and Technology/National Agency for Research and Innovation	2020-03-27	Edison Johar et al
Indonesia/JKT-EIJK0317/2020	EPI_ISL_435282	RS Pondok Indah Hospital – Pondok Indah	Eijkman Institute for Molecular Biology, Ministry of Research and Technology/National Agency for Research and Innovation	2020-03-19	Edison Johar et al
Indonesia/JKT-EIJK04/2020	EPI_ISL_437192	Mitra Keluarga Kelapa Gading Hospital	Eijkman Institute for Molecular Biology, Ministry of Research and Technology/National Agency for Research and Innovation	2020-04-01	Edison Johar et al
Indonesia/JKT-EIJK07/2020	EPI_ISL_467376	RSUP Fatmawati	Eijkman Institute for Molecular Biology, Ministry of Research and Technology/National Agency for Research and Innovation	2020-04-24	Edison Johar et al
Indonesia/JKT-EIJK2444/2020	EPI_ISL_435283	RS Pondok Indah Hospital – Pondok Indah	Eijkman Institute for Molecular Biology, Ministry of Research and Technology/National Agency for Research and Innovation	2020-03-30	Edison Johar et al
Indonesia/MND-EIJK06/2020	EPI_ISL_467375	RSUP Prof. Dr. R. Kandou Manado	Eijkman Institute for Molecular Biology, Ministry of Research and Technology/National Agency for Research and Innovation	2020-03-23	Edison Johar et al
Indonesia/SMR-EIJK05/2020	EPI_ISL_467374	Dinkes Samarinda	Eijkman Institute for Molecular Biology, Ministry of Research and Technology/National Agency for Research and Innovation	2020-03-18	Edison Johar et al
Iran/HGRC-01-IPI-8206/2020	EPI_ISL_424349		Human Genetic Research Center	2020-03-09	Zeinali et al
Iran/HGRC-2-2162/2020	EPI_ISL_437512		Human Genetic Research Center	2020-03-26	Khosravi et al
Iran/KHGRC-1.1-IPI-8206/2020	EPI_ISL_442523	Pasteur Institute of Iran	Kawsar Human Genetic Research Company	2020-03-09	Sirous Zeinali et al
Iran/KHGRC-2-2162/2020	EPI_ISL_442044	Kawsar Human Genetic Research Center	Kawsar Human Genetic Research Center	2020-03-26	Mohammad Ali Khosravi et al
Iran/KHGRC-3-2178/2020	EPI_ISL_445088		Human Genetic Research Center	2020-03-26	Abbasalipour Bashash et al
Ireland/21023/2020	EPI_ISL_418516	UCD National Virus Reference Laboratory	UCD National Virus Reference Laboratory	2020-03-06	Michael Carr et al
Ireland/21145/2020	EPI_ISL_418548	UCD National Virus Reference Laboratory	UCD National Virus Reference Laboratory	2020-03-06	Michael Carr et al
Ireland/24052/2020	EPI_ISL_418584	UCD National Virus Reference Laboratory	UCD National Virus Reference Laboratory	2020-03-10	Michael Carr et al
Ireland/Cork2/2020	EPI_ISL_417432	UCD National Virus Reference Laboratory	Public Health Wales Microbiology Cardiff	2020-03-08	Catherine Dempsey et al
Ireland/Dublin-19072/2020	EPI_ISL_414584	UCD National Virus Reference Laboratory	UCD National Virus Reference Laboratory	2020-03-02	Michael Carr et al
Ireland/Dublin-22361/2020	EPI_ISL_418580	UCD National Virus Reference Laboratory	UCD National Virus Reference Laboratory	2020-03-08	Michael Carr et al
Ireland/Dublin-22428/2020	EPI_ISL_418581	UCD National Virus Reference Laboratory	UCD National Virus Reference Laboratory	2020-03-08	Michael Carr et al
Ireland/Limerick-19933/2020	EPI_ISL_414585	UCD National Virus Reference Laboratory	UCD National Virus Reference Laboratory	2020-03-03	Michael Carr et al
Ireland/Limerick-19934/2020	EPI_ISL_414586	UCD National Virus Reference Laboratory	UCD National Virus Reference Laboratory	2020-03-03	Michael Carr et al
Ireland/Limerick-19935/2020	EPI_ISL_414587	UCD National Virus Reference Laboratory	UCD National Virus Reference Laboratory	2020-03-03	Michael Carr et al
Israel/130710067/2020	EPI_ISL_447468	Clinical Microbiology Laboratory, Sheba Medical Center	Stern Lab	2020-04-14	Stern Lab et al (https://dx.doi.org/10.1101/2020.05.21.20104521)
Israel/130710217/2020	EPI_ISL_447462	Clinical Microbiology Laboratory, Sheba Medical Center	Stern Lab	2020-04-16	Stern Lab et al (https://dx.doi.org/10.1101/2020.05.21.20104521)
Israel/130710390/2020	EPI_ISL_447461	Clinical Microbiology Laboratory, Sheba Medical Center	Stern Lab	2020-04-17	Stern Lab et al (https://dx.doi.org/10.1101/2020.05.21.20104521)
Israel/130711082/2020	EPI_ISL_447456	Clinical Microbiology Laboratory, Sheba Medical Center	Stern Lab	2020-04-20	Stern Lab et al (https://dx.doi.org/10.1101/2020.05.21.20104521)
Israel/130711367/2020	EPI_ISL_447452	Clinical Microbiology Laboratory, Sheba Medical Center	Stern Lab	2020-04-22	Stern Lab et al (https://dx.doi.org/10.1101/2020.05.21.20104521)
Israel/13075703/2020	EPI_ISL_447450	Clinical Microbiology Laboratory, Sheba Medical Center	Stern Lab	2020-03-29	Stern Lab et al (https://dx.doi.org/10.1101/2020.05.21.20104521)
Israel/13075782/2020	EPI_ISL_447447	Clinical Microbiology Laboratory, Sheba Medical Center	Stern Lab	2020-03-30	Stern Lab et al (https://dx.doi.org/10.1101/2020.05.21.20104521)
Israel/13075788/2020	EPI_ISL_447446	Clinical Microbiology Laboratory, Sheba Medical Center	Stern Lab	2020-03-30	Stern Lab et al (https://dx.doi.org/10.1101/2020.05.21.20104521)
Israel/13075790/2020	EPI_ISL_447445	Clinical Microbiology Laboratory, Sheba Medical Center	Stern Lab	2020-03-30	Stern Lab et al (https://dx.doi.org/10.1101/2020.05.21.20104521)
Israel/13075882/2020	EPI_ISL_447442	Clinical Microbiology Laboratory, Sheba Medical Center	Stern Lab	2020-03-30	Stern Lab et al (https://dx.doi.org/10.1101/2020.05.21.20104521)
Israel/13077377/2020	EPI_ISL_447440	Clinical Microbiology Laboratory, Sheba Medical Center	Stern Lab	2020-04-02	Stern Lab et al (https://dx.doi.org/10.1101/2020.05.21.20104521)
Israel/13077511/2020	EPI_ISL_447433	Clinical Microbiology Laboratory, Sheba Medical Center	Stern Lab	2020-04-03	Stern Lab et al (https://dx.doi.org/10.1101/2020.05.21.20104521)
Israel/13077726/2020	EPI_ISL_447426	Clinical Microbiology Laboratory, Sheba Medical Center	Stern Lab	2020-04-03	Stern Lab et al (https://dx.doi.org/10.1101/2020.05.21.20104521)
Israel/13077846/2020	EPI_ISL_447422	Clinical Microbiology Laboratory, Sheba Medical Center	Stern Lab	2020-04-04	Stern Lab et al (https://dx.doi.org/10.1101/2020.05.21.20104521)
Israel/13077847/2020	EPI_ISL_447421	Clinical Microbiology Laboratory, Sheba Medical Center	Stern Lab	2020-04-04	Stern Lab et al (https://dx.doi.org/10.1101/2020.05.21.20104521)
Israel/13077882/2020	EPI_ISL_447419	Clinical Microbiology Laboratory, Sheba Medical Center	Stern Lab	2020-04-04	Stern Lab et al (https://dx.doi.org/10.1101/2020.05.21.20104521)
Israel/2020063/2020	EPI_ISL_447414	Clinical Virology Unit, Hadassah Hebrew University Medical Center	Stern Lab	2020-03-17	Stern Lab et al (https://dx.doi.org/10.1101/2020.05.21.20104521)
Israel/2020068/2020	EPI_ISL_447413	Clinical Virology Unit, Hadassah Hebrew University Medical Center	Stern Lab	2020-03-17	Stern Lab et al (https://dx.doi.org/10.1101/2020.05.21.20104521)
Israel/2020069/2020	EPI_ISL_447412	Clinical Virology Unit, Hadassah Hebrew University Medical Center	Stern Lab	2020-03-17	Stern Lab et al (https://dx.doi.org/10.1101/2020.05.21.20104521)
Israel/2020084/2020	EPI_ISL_447410	Clinical Virology Unit, Hadassah Hebrew University Medical Center	Stern Lab	2020-03-17	Stern Lab et al (https://dx.doi.org/10.1101/2020.05.21.20104521)
Israel/2023920/2020	EPI_ISL_447408	Clinical Virology Unit, Hadassah Hebrew University Medical Center	Stern Lab	2020-03-17	Stern Lab et al (https://dx.doi.org/10.1101/2020.05.21.20104521)
Israel/2046171/2020	EPI_ISL_447405	Clinical Microbiology Laboratory, The Baruch Padeh Medical Center, Poriya	Stern Lab	2020-03-31	Stern Lab et al (https://dx.doi.org/10.1101/2020.05.21.20104521)
Israel/2047016/2020	EPI_ISL_447397	Clinical Microbiology Laboratory, The Baruch Padeh Medical Center, Poriya	Stern Lab	2020-03-26	Stern Lab et al (https://dx.doi.org/10.1101/2020.05.21.20104521)
Israel/2047364/2020	EPI_ISL_447393	Clinical Microbiology Laboratory, The Baruch Padeh Medical Center, Poriya	Stern Lab	2020-03-28	Stern Lab et al (https://dx.doi.org/10.1101/2020.05.21.20104521)
Israel/2047392/2020	EPI_ISL_447392	Clinical Microbiology Laboratory, The Baruch Padeh Medical Center, Poriya	Stern Lab	2020-03-29	Stern Lab et al (https://dx.doi.org/10.1101/2020.05.21.20104521)
Israel/2047586/2020	EPI_ISL_447389	Clinical Microbiology Laboratory, The Baruch Padeh Medical Center, Poriya	Stern Lab	2020-03-29	Stern Lab et al (https://dx.doi.org/10.1101/2020.05.21.20104521)
Israel/2047604/2020	EPI_ISL_447388	Clinical Microbiology Laboratory, The Baruch Padeh Medical Center, Poriya	Stern Lab	2020-03-29	Stern Lab et al (https://dx.doi.org/10.1101/2020.05.21.20104521)
Israel/2047772/2020	EPI_ISL_447385	Clinical Microbiology Laboratory, The Baruch Padeh Medical Center, Poriya	Stern Lab	2020-03-30	Stern Lab et al (https://dx.doi.org/10.1101/2020.05.21.20104521)
Israel/2086004/2020	EPI_ISL_447381	Clinical Virology Unit, Hadassah Hebrew University Medical Center	Stern Lab	2020-04-01	Stern Lab et al (https://dx.doi.org/10.1101/2020.05.21.20104521)
Israel/2086008/2020	EPI_ISL_447380	Clinical Virology Unit, Hadassah Hebrew University Medical Center	Stern Lab	2020-04-01	Stern Lab et al (https://dx.doi.org/10.1101/2020.05.21.20104521)
Israel/2086022/2020	EPI_ISL_447378	Clinical Virology Unit, Hadassah Hebrew University Medical Center	Stern Lab	2020-04-01	Stern Lab et al (https://dx.doi.org/10.1101/2020.05.21.20104521)
Israel/2086034/2020	EPI_ISL_447376	Clinical Virology Unit, Hadassah Hebrew University Medical Center	Stern Lab	2020-04-01	Stern Lab et al (https://dx.doi.org/10.1101/2020.05.21.20104521)
Israel/2089380/2020	EPI_ISL_447371	Clinical Virology Unit, Hadassah Hebrew University Medical Center	Stern Lab	2020-04-01	Stern Lab et al (https://dx.doi.org/10.1101/2020.05.21.20104521)
Israel/2089697/2020	EPI_ISL_447369	Clinical Virology Unit, Hadassah Hebrew University Medical Center	Stern Lab	2020-04-01	Stern Lab et al (https://dx.doi.org/10.1101/2020.05.21.20104521)
Israel/2089718/2020	EPI_ISL_447366	Clinical Virology Unit, Hadassah Hebrew University Medical Center	Stern Lab	2020-04-01	Stern Lab et al (https://dx.doi.org/10.1101/2020.05.21.20104521)
Israel/2089723/2020	EPI_ISL_447365	Clinical Virology Unit, Hadassah Hebrew University Medical Center	Stern Lab	2020-04-01	Stern Lab et al (https://dx.doi.org/10.1101/2020.05.21.20104521)
Israel/2089863/2020	EPI_ISL_447360	Clinical Virology Unit, Hadassah Hebrew University Medical Center	Stern Lab	2020-04-01	Stern Lab et al (https://dx.doi.org/10.1101/2020.05.21.20104521)
Israel/2099018/2020	EPI_ISL_447358	Clinical Virology Unit, Hadassah Hebrew University Medical Center	Stern Lab	2020-04-02	Stern Lab et al (https://dx.doi.org/10.1101/2020.05.21.20104521)
Israel/2099019/2020	EPI_ISL_447357	Clinical Virology Unit, Hadassah Hebrew University Medical Center	Stern Lab	2020-04-02	Stern Lab et al (https://dx.doi.org/10.1101/2020.05.21.20104521)
Israel/2099421/2020	EPI_ISL_447353	Clinical Virology Unit, Hadassah Hebrew University Medical Center	Stern Lab	2020-04-02	Stern Lab et al (https://dx.doi.org/10.1101/2020.05.21.20104521)
Israel/2113155/2020	EPI_ISL_447349	Clinical Virology Unit, Hadassah Hebrew University Medical Center	Stern Lab	2020-04-15	Stern Lab et al (https://dx.doi.org/10.1101/2020.05.21.20104521)
Israel/2113161/2020	EPI_ISL_447348	Clinical Virology Unit, Hadassah Hebrew University Medical Center	Stern Lab	2020-04-15	Stern Lab et al (https://dx.doi.org/10.1101/2020.05.21.20104521)
Israel/2113255/2020	EPI_ISL_447344	Clinical Virology Unit, Hadassah Hebrew University Medical Center	Stern Lab	2020-04-14	Stern Lab et al (https://dx.doi.org/10.1101/2020.05.21.20104521)

Israel/2113601/2020	EPI_ISL_447342	Clinical Virology Unit, Hadassah Hebrew University Medical Center	Stern Lab	2020-04-15	Stern Lab et al (https://dx.doi.org/10.1101/2020.05.21.20104521)
Israel/2113678/2020	EPI_ISL_447340	Clinical Virology Unit, Hadassah Hebrew University Medical Center	Stern Lab	2020-04-15	Stern Lab et al (https://dx.doi.org/10.1101/2020.05.21.20104521)
Israel/51145198/2020	EPI_ISL_447317	Clinical Virology Laboratory, Soroka Medical Center and the Faculty of Health Sciences, Ben-Gurion University of the Negev	Stern Lab	2020-04-01	Stern Lab et al (https://dx.doi.org/10.1101/2020.05.21.20104521)
Israel/51145482/2020	EPI_ISL_447316	Clinical Virology Laboratory, Soroka Medical Center and the Faculty of Health Sciences, Ben-Gurion University of the Negev	Stern Lab	2020-04-05	Stern Lab et al (https://dx.doi.org/10.1101/2020.05.21.20104521)
Israel/701002317/2020	EPI_ISL_447308	Microbiology Division, Barzilai University Medical Center	Stern Lab	2020-03-21	Stern Lab et al (https://dx.doi.org/10.1101/2020.05.21.20104521)
Israel/701002431/2020	EPI_ISL_447302	Microbiology Division, Barzilai University Medical Center	Stern Lab	2020-03-24	Stern Lab et al (https://dx.doi.org/10.1101/2020.05.21.20104521)
Israel/701002462/2020	EPI_ISL_447296	Microbiology Division, Barzilai University Medical Center	Stern Lab	2020-03-24	Stern Lab et al (https://dx.doi.org/10.1101/2020.05.21.20104521)
Israel/701002555/2020	EPI_ISL_447290	Microbiology Division, Barzilai University Medical Center	Stern Lab	2020-03-26	Stern Lab et al (https://dx.doi.org/10.1101/2020.05.21.20104521)
Israel/701002556/2020	EPI_ISL_447289	Microbiology Division, Barzilai University Medical Center	Stern Lab	2020-03-26	Stern Lab et al (https://dx.doi.org/10.1101/2020.05.21.20104521)
Israel/701002561/2020	EPI_ISL_447288	Microbiology Division, Barzilai University Medical Center	Stern Lab	2020-03-26	Stern Lab et al (https://dx.doi.org/10.1101/2020.05.21.20104521)
Israel/701002768/2020	EPI_ISL_447283	Microbiology Division, Barzilai University Medical Center	Stern Lab	2020-03-28	Stern Lab et al (https://dx.doi.org/10.1101/2020.05.21.20104521)
Israel/701002792/2020	EPI_ISL_447281	Microbiology Division, Barzilai University Medical Center	Stern Lab	2020-03-29	Stern Lab et al (https://dx.doi.org/10.1101/2020.05.21.20104521)
Israel/990059204/2020	EPI_ISL_447278	Microbiology laboratory, Assuta Ashdod University-Affiliated Hospital	Stern Lab	2020-03-25	Stern Lab et al (https://dx.doi.org/10.1101/2020.05.21.20104521)
Israel/990059217/2020	EPI_ISL_447277	Microbiology laboratory, Assuta Ashdod University-Affiliated Hospital	Stern Lab	2020-03-25	Stern Lab et al (https://dx.doi.org/10.1101/2020.05.21.20104521)
Israel/990059237/2020	EPI_ISL_447272	Microbiology laboratory, Assuta Ashdod University-Affiliated Hospital	Stern Lab	2020-03-25	Stern Lab et al (https://dx.doi.org/10.1101/2020.05.21.20104521)
Israel/990333193/2020	EPI_ISL_447260	Microbiology laboratory, Assuta Ashdod University-Affiliated Hospital	Stern Lab	2020-03-26	Stern Lab et al (https://dx.doi.org/10.1101/2020.05.21.20104521)
Israel/ISR_JP0320/2020	EPI_ISL_419211	Central Virology Laboratory	Israel Institute for Biological Research	2020-02-23	Inbar Cohen-Gihon et al (https://dx.doi.org/10.1101/2020.05.21.20104521)
Italy/206/2020	EPI_ISL_457699	Department of Infectious Diseases, Istituto Superiore di Sanità, Roma , Italy	Army Medical and Veterinary Research Center	2020-02-22	Paola Stefanelli et al B
Italy/2629/2020	EPI_ISL_457700	Department of Infectious Diseases, Istituto Superiore di Sanità, Roma , Italy	Army Medical and Veterinary Research Center	2020-03-06	Paola Stefanelli et al B
Italy/484/2020	EPI_ISL_457749	Department of Infectious Diseases, Istituto Superiore di Sanità, Roma , Italy	Army Medical and Veterinary Research Center	2020-02-27	Paola Stefanelli et al B
Italy/4870/2020	EPI_ISL_457732	Department of Infectious Diseases, Istituto Superiore di Sanità, Roma , Italy	Army Medical and Veterinary Research Center	2020-03-14	Paola Stefanelli et al B
Italy/4875/2020	EPI_ISL_457724	Department of Infectious Diseases, Istituto Superiore di Sanità, Roma , Italy	Army Medical and Veterinary Research Center	2020-03-15	Paola Stefanelli et al B
Italy/AMC1-4634/2020	EPI_ISL_457825	Army Medical Research Center - Scientific Department	Army Medical and Veterinary Research Center	2020-03-25	Silvia Fillo et al
Italy/AMC2-1245/2020	EPI_ISL_457826	Army Medical Center - Scientific Department	Army Medical and Veterinary Research Center	2020-03-08	Silvia Fillo et al
Italy/BG-11639-28Feb/2020	EPI_ISL_460080	Molecular Virology Unit, Fondazione IRCCS Policlinico San Matteo , Pavia	Laboratory of Virology, INMI Lazzaro Spallanzani IRCCS	2020-02-28	Antonio Piralla et al
Italy/BG-5825-22Feb/2020	EPI_ISL_460079	Molecular Virology Unit, Fondazione IRCCS Policlinico San Matteo , Pavia	Laboratory of Virology, INMI Lazzaro Spallanzani IRCCS	2020-02-22	Barbara Bartolini et al
Italy/BG-7158-24Feb/2020	EPI_ISL_460081	Molecular Virology Unit, Fondazione IRCCS Policlinico San Matteo , Pavia	Laboratory of Virology, INMI Lazzaro Spallanzani IRCCS	2020-02-24	Fausto Baldanti et al
Italy/BG-9209-26Feb/2020	EPI_ISL_460082	Molecular Virology Unit, Fondazione IRCCS Policlinico San Matteo , Pavia	Laboratory of Virology, INMI Lazzaro Spallanzani IRCCS	2020-02-26	Martina Rueca et al
Italy/BG-9225-26Feb/2020	EPI_ISL_460083	Molecular Virology Unit, Fondazione IRCCS Policlinico San Matteo , Pavia	Laboratory of Virology, INMI Lazzaro Spallanzani IRCCS	2020-02-26	Martina Rueca et al
Italy/BS-12256-29Feb/2020	EPI_ISL_460084	Molecular Virology Unit, Fondazione IRCCS Policlinico San Matteo , Pavia	Laboratory of Virology, INMI Lazzaro Spallanzani IRCCS	2020-02-29	Fausto Baldanti et al
Italy/CDG1/2020	EPI_ISL_412973	Department of Infectious Diseases, Istituto Superiore di Sanità, Roma , Italy	Virology Laboratory, Scientific Department, Army Medical Center	2020-02-20	Paola Stefanelli et al A (https://dx.doi.org/10.2807/1560-7917.ES.2020.25.13.2000305)
Italy/CR-7070-24Feb/2020	EPI_ISL_460086	Molecular Virology Unit, Fondazione IRCCS Policlinico San Matteo , Pavia	Laboratory of Virology, INMI Lazzaro Spallanzani IRCCS	2020-02-24	Maria R. Capobianchi et al B
Italy/CR-9206-26Feb/2020	EPI_ISL_460087	Molecular Virology Unit, Fondazione IRCCS Policlinico San Matteo , Pavia	Laboratory of Virology, INMI Lazzaro Spallanzani IRCCS	2020-02-26	Cesare E.M. Gruber et al A
Italy/CR-9214-26Feb/2020	EPI_ISL_460088	Molecular Virology Unit, Fondazione IRCCS Policlinico San Matteo , Pavia	Laboratory of Virology, INMI Lazzaro Spallanzani IRCCS	2020-02-26	Martina Rueca et al
Italy/CR-9491-26Feb/2020	EPI_ISL_460089	Molecular Virology Unit, Fondazione IRCCS Policlinico San Matteo , Pavia	Laboratory of Virology, INMI Lazzaro Spallanzani IRCCS	2020-02-26	Antonino Di Caro et al
Italy/FVG-ICGEB_S5/2020	EPI_ISL_417419	Laboratory of Molecular Virology International Center for Genetic Engineering and Biotechnology (ICGEB)	ARGO Open Lab Platform for Genome sequencing	2020-03-01	Licastro D et al (https://dx.doi.org/10.1128/JVI.00543-20)
Italy/INMI11-B/2020	EPI_ISL_451304	Laboratory of Virology, INMI Lazzaro Spallanzani IRCCS	Laboratory of Virology, INMI Lazzaro Spallanzani IRCCS	2020-03-23	Cesare E.M. Gruber et al A
Italy/INMI4/2020	EPI_ISL_417922	INMI Lazzaro Spallanzani IRCCS	Laboratory of Virology, INMI Lazzaro Spallanzani IRCCS	2020-02-28	Cesare E. M. Gruber et al
Italy/INMI9/2020	EPI_ISL_424343	INMI Lazzaro Spallanzani IRCCS	Laboratory of Virology, INMI Lazzaro Spallanzani IRCCS	2020-03-23	Fabrizio Carletti et al
Italy/IZSPB_809/2020	EPI_ISL_469020	Istituto Zooprofilattico Sperimentale Puglia e Basilicata; Dipartimento di Bioscienze, Biotecnologie e Biofarmaceutica dell'Università degli Studi di Bari "A.Moro"; Istituto di Biomembrane. Bioenergetica e Biotecnologie Molecolari del Consiglio Nazionale delle Ricerche di Bari	Beaconlab (Bioinformatics, Evolution and Comparative Genomics lab), Dept of Biosciences, University on Milan	2020-03-19	Parisi A. et al
Italy/IZSPB_CL1/2020	EPI_ISL_451961	Istituto Zooprofilattico Sperimentale Puglia e Basilicata; Dipartimento di Bioscienze, Biotecnologie e Biofarmaceutica dell'Università degli Studi di Bari "A.Moro"; Istituto di Biomembrane. Bioenergetica e Biotecnologie Molecolari del Consiglio Nazionale delle Ricerche di Bari	Beaconlab (Bioinformatics Evolution and Comparative Genomics lab), Dept of Biosciences, University of Milan	2020-03-14	Parisi A. et al
Italy/LO-11550-28Feb/2020	EPI_ISL_460090	Molecular Virology Unit, Fondazione IRCCS Policlinico San Matteo , Pavia	Laboratory of Virology, INMI Lazzaro Spallanzani IRCCS	2020-02-28	Antonio Piralla et al
Italy/LO-13075-N/2020	EPI_ISL_451309	Molecular Virology Unit, Fondazione IRCCS Policlinico San Matteo , Pavia	Laboratory of Virology, INMI Lazzaro Spallanzani IRCCS	2020-03-01	Fausto Baldanti et al
Italy/MI-5925-22Feb/2020	EPI_ISL_460091	Molecular Virology Unit, Fondazione IRCCS Policlinico San Matteo , Pavia	Laboratory of Virology, INMI Lazzaro Spallanzani IRCCS	2020-02-22	Antonino Di Caro et al
Italy/PV-11951-28Feb/2020	EPI_ISL_460092	Molecular Virology Unit, Fondazione IRCCS Policlinico San Matteo , Pavia	Laboratory of Virology, INMI Lazzaro Spallanzani IRCCS	2020-02-28	Cesare E.M. Gruber et al A
Italy/PV-12578-29Feb/2020	EPI_ISL_460093	Molecular Virology Unit, Fondazione IRCCS Policlinico San Matteo , Pavia	Laboratory of Virology, INMI Lazzaro Spallanzani IRCCS	2020-02-29	Maria R. Capobianchi et al B
Italy/PV-5314-B/2020	EPI_ISL_451306	Molecular Virology Unit, Fondazione IRCCS Policlinico San Matteo , Pavia	Laboratory of Virology, INMI Lazzaro Spallanzani IRCCS	2020-02-21	Antonio Piralla et al
Italy/PV-5314-N/2020	EPI_ISL_451307	Molecular Virology Unit, Fondazione IRCCS Policlinico San Matteo , Pavia	Laboratory of Virology, INMI Lazzaro Spallanzani IRCCS	2020-02-21	Fausto Baldanti et al
Italy/PV-8058-25Feb/2020	EPI_ISL_460094	Molecular Virology Unit, Fondazione IRCCS Policlinico San Matteo , Pavia	Laboratory of Virology, INMI Lazzaro Spallanzani IRCCS	2020-02-25	Barbara Bartolini et al
Italy/PV-9675-26Feb/2020	EPI_ISL_460095	Molecular Virology Unit, Fondazione IRCCS Policlinico San Matteo , Pavia	Laboratory of Virology, INMI Lazzaro Spallanzani IRCCS	2020-02-26	Barbara Bartolini et al
ITALY/SardiniaNuoro04039046/2020	EPI_ISL_458084	Laboratorio Biologia Molecolare Sars Cov2 - UOC Laboratorio Analisi - Servizio Medicina di Laboratorio, Ospedale "San Francesco" - ATS-ASSL Nuoro	Laboratorio specialistico UOC Ematologia - Ospedale "San Francesco" - ATS-ASSL Nuoro	2020-04-03	Piras Giovanna et al
ITALY/SardiniaNuoroCOV28/2020	EPI_ISL_458085	Laboratorio Biologia Molecolare Sars Cov2 - UOC Laboratorio Analisi - Servizio Medicina di Laboratorio , Ospedale "San Francesco" - ATS-ASSL Nuoro	Laboratorio specialistico UOC Ematologia - Ospedale "San Francesco"- ATS-ASSL Nuoro	2020-04-12	Piras Giovanna et al
Italy/TE12337/2020	EPI_ISL_435146	Villa Serena del Dr. Leonardo Petruzzi	Istituto Zooprofilattico Sperimentale dell’Abruzzo e Molise “G. Caporale”	2020-04-07	Lorusso A et al
Italy/TE12759/2020	EPI_ISL_435147	Villa Serena del Dr. Leonardo Petruzzi	Istituto Zooprofilattico Sperimentale dell’Abruzzo e Molise “G. Caporale”	2020-04-08	Lorusso A et al
Italy/TE12774/2020	EPI_ISL_435150	Ospedale SS Annunziata	Istituto Zooprofilattico Sperimentale dell’Abruzzo e Molise “G. Caporale”	2020-04-08	Lorusso A et al
Italy/TE13457/2020	EPI_ISL_435149	SERVIZIO DI IGIENE E SANITÀ PUBBLICA ASL Teramo	Istituto Zooprofilattico Sperimentale dell’Abruzzo e Molise “G. Caporale”	2020-04-08	Lorusso A et al
Italy/TE13484/2020	EPI_ISL_435151	Ospedale SS Annunziata	Istituto Zooprofilattico Sperimentale dell’Abruzzo e Molise “G. Caporale”	2020-04-08	Lorusso A et al
Italy/TE13491/2020	EPI_ISL_435148	Ospedale SS Annunziata	Istituto Zooprofilattico Sperimentale dell’Abruzzo e Molise “G. Caporale”	2020-04-08	Lorusso A et al
Italy/TE13858/2020	EPI_ISL_435152	Servizio di Igiene, Epidemiologia e Sanità Pubblica (SIESP) Avezzano	Istituto Zooprofilattico Sperimentale dell’Abruzzo e Molise “G. Caporale”	2020-04-09	Lorusso A et al
Italy/TE14162/2020	EPI_ISL_435154	SERVIZIO DI IGIENE E SANITÀ PUBBLICA ASL Teramo	Istituto Zooprofilattico Sperimentale dell’Abruzzo e Molise “G. Caporale”	2020-04-09	Lorusso A et al
Italy/TE14168/2020	EPI_ISL_435155	SERVIZIO DI IGIENE E SANITÀ PUBBLICA ASL Teramo	Istituto Zooprofilattico Sperimentale dell’Abruzzo e Molise “G. Caporale”	2020-04-09	Lorusso A et al
Italy/TE14297/2020	EPI_ISL_435153	SERVIZIO DI IGIENE E SANITÀ PUBBLICA ASL Teramo	Istituto Zooprofilattico Sperimentale dell’Abruzzo e Molise “G. Caporale”	2020-04-09	Lorusso A et al
Italy/TE26423/2020	EPI_ISL_436731	Ospedale Civile S. Liberatore di Atri	Istituto Zooprofilattico Sperimentale dell’Abruzzo e Molise “G. Caporale”	2020-04-26	Lorusso A et al
Italy/TE26425/2020	EPI_ISL_436732	Ospedale Civile S. Liberatore di Atri	Istituto Zooprofilattico Sperimentale dell’Abruzzo e Molise “G. Caporale”	2020-04-27	Lorusso A et al
Italy/TE26533/2020	EPI_ISL_436726	SERVIZIO DI IGIENE E SANITÀ PUBBLICA ASL Teramo	Istituto Zooprofilattico Sperimentale dell’Abruzzo e Molise “G. Caporale”	2020-04-27	Lorusso A et al
Italy/TE26539/2020	EPI_ISL_436727	SERVIZIO DI IGIENE E SANITÀ PUBBLICA ASL Teramo	Istituto Zooprofilattico Sperimentale dell’Abruzzo e Molise “G. Caporale”	2020-04-27	Lorusso A et al
Italy/TE26540/2020	EPI_ISL_436728	SERVIZIO DI IGIENE E SANITÀ PUBBLICA ASL Teramo	Istituto Zooprofilattico Sperimentale dell’Abruzzo e Molise “G. Caporale”	2020-04-27	Lorusso A et al
Italy/TE26643/2020	EPI_ISL_436729	SERVIZIO DI IGIENE E SANITÀ PUBBLICA ASL Teramo	Istituto Zooprofilattico Sperimentale dell’Abruzzo e Molise “G. Caporale”	2020-04-27	Lorusso A et al
Italy/TE27020/2020	EPI_ISL_436725	RSA/RP Villa San Giovanni - Gruppo Edos	Istituto Zooprofilattico Sperimentale dell’Abruzzo e Molise “G. Caporale”	2020-04-27	Lorusso A et al
Italy/TE4836/2020	EPI_ISL_418260	Ospedale Civile Giuseppe Mazzini	Istituto Zooprofilattico Sperimentale dell'Abruzzo e Molise "G. Caporale"	2020-03-16	Lorusso A et al
Italy/TE4925/2020	EPI_ISL_418255	Presidio Ospedaliero "S. Spirito" - PESCARA	Istituto Zooprofilattico Sperimentale dell'Abruzzo e Molise "G. Caporale"	2020-03-14	Lorusso A et al
Italy/TE4959/2020	EPI_ISL_418259	Presidio ospedaliero "Santo Spirito"	Istituto Zooprofilattico Sperimentale dell'Abruzzo e Molise "G. Caporale"	2020-03-14	Lorusso A et al
Italy/TE5052/2020	EPI_ISL_418261	Ospedale Civile Giuseppe Mazzini	Istituto Zooprofilattico Sperimentale dell'Abruzzo e Molise "G. Caporale"	2020-03-17	Lorusso A et al
Italy/TE5056/2020	EPI_ISL_418257	Ospedale Civile Giuseppe Mazzini, Teramo	Istituto Zooprofilattico Sperimentale dell'Abruzzo e Molise "G.Caporale"	2020-03-17	Lorusso A et al
Italy/TE5166/2020	EPI_ISL_420563	Ospedale Civile Giuseppe Mazzini	Istituto Zooprofilattico Sperimentale dell'Abruzzo e Molise "G. Caporale"	2020-03-18	Lorusso A et al
Italy/TE5473/2020	EPI_ISL_436718	Ospedale Regionale San Salvatore	Istituto Zooprofilattico Sperimentale dell’Abruzzo e Molise “G. Caporale”	2020-03-19	Lorusso A et al
Italy/TE5545/2020	EPI_ISL_436721	Ospedale Civile S. Liberatore di Atri	Istituto Zooprofilattico Sperimentale dell’Abruzzo e Molise “G. Caporale”	2020-03-20	Lorusso A et al
Italy/TE5547/2020	EPI_ISL_436722	Ospedale Civile S. Liberatore di Atri	Istituto Zooprofilattico Sperimentale dell’Abruzzo e Molise “G. Caporale”	2020-03-20	Lorusso A et al
Italy/TE5551/2020	EPI_ISL_429226	Presidio Ospedaliero Santo Spirito	Istituto Zooprofilattico Sperimentale dell'Abruzzo e Molise "G. Caporale"	2020-03-17	Lorusso A et al
Italy/TE5674/2020	EPI_ISL_429228	Ospedale Civile Giuseppe Mazzini	Istituto Zooprofilattico Sperimentale dell'Abruzzo e Molise "G. Caporale"	2020-03-20	Lorusso A et al
Italy/TE5687/2020	EPI_ISL_436723	Ospedale Civile Giuseppe Mazzini	Istituto Zooprofilattico Sperimentale dell’Abruzzo e Molise “G. Caporale”	2020-03-20	Lorusso A et al
Italy/TE6195/2020	EPI_ISL_420569	Ospedale Civile Giuseppe Mazzini	Istituto Zooprofilattico Sperimentale dell’Abruzzo e Molise “G. Caporale”	2020-03-23	Lorusso A et al
Italy/TE6572/2020	EPI_ISL_429232	Ospedale Civile Giuseppe Mazzini	Istituto Zooprofilattico Sperimentale dell'Abruzzo e Molise "G. Caporale"	2020-03-24	Lorusso A et al
Italy/TE6575/2020	EPI_ISL_429233	Ospedale Civile Giuseppe Mazzini	Istituto Zooprofilattico Sperimentale dell'Abruzzo e Molise "G. Caporale"	2020-03-24	Lorusso A et al
Italy/TE6644/2020	EPI_ISL_435145	Ospedale Civile Giuseppe Mazzini	Istituto Zooprofilattico Sperimentale dell’Abruzzo e Molise “G. Caporale”	2020-03-24	Lorusso A et al
Italy/UniMI01/2020	EPI_ISL_417445	Laboratory of Infectious Diseases, Department of Biomedical and Clinical Sciences L. Sacco, University of Milan	Laboratory of Infectious Diseases, Department of Biomedical and Clinical Sciences L. Sacco, University of Milan	2020-02-24	Gianguglielmo Zehender et al
Italy/UniMI03/2020	EPI_ISL_417447	Laboratory of Infectious Diseases, Department of Biomedical and Clinical Sciences L. Sacco, University of Milan	Laboratory of Infectious Diseases, Department of Biomedical and Clinical Sciences L. Sacco, University of Milan	2020-02-24	Gianguglielmo Zehender et al
Italy/UniSR1/2020	EPI_ISL_413489	Laboratorio di Microbiologia e Virologia, Università Vita-Salute San Raffaele, Milano	Laboratorio di Microbiologia e Virologia, Università Vita-Salute San Raffaele, Milano	2020-03-03	R.A Diotti et al
Italy/VR_20COV25-80/2020	EPI_ISL_452186	ULSS9 Distretto di Bussolengo	Istituto Zooprofilattico Sperimentale delle Venezie	2020-03-28	Adelaide Milani et al
Italy/VR_20COV31-19/2020	EPI_ISL_452188	ULSS9 Distretto di Bussolengo	Istituto Zooprofilattico Sperimentale delle Venezie	2020-03-31	Adelaide Milani et al
Italy/VR_20COV33-126/2020	EPI_ISL_452189	ULSS9 Distretto di Bussolengo	Istituto Zooprofilattico Sperimentale delle Venezie	2020-04-01	Adelaide Milani et al
Italy/VR_20COV46-23/2020	EPI_ISL_452191	ULSS9 Distretto di San Bonifacio	Istituto Zooprofilattico Sperimentale delle Venezie	2020-04-03	Adelaide Milani et al
Italy/VR_20COV46-8/2020	EPI_ISL_452190	ULSS9 Distretto di San Bonifacio	Istituto Zooprofilattico Sperimentale delle Venezie	2020-04-03	Adelaide Milani et al
Jamaica/JM-CDC-4376/2020	EPI_ISL_450794	Jamaica Ministry of Health and Wellness	Pathogen Discovery, Respiratory Viruses Branch, Division of Viral Diseases, Centers for Disease Control and Prevention	2020-03-11	Yan Li et al A
Jamaica/JM-CDC-6603/2020	EPI_ISL_450797	Jamaica Ministry of Health and Wellness	Pathogen Discovery, Respiratory Viruses Branch, Division of Viral Diseases, Centers for Disease Control and Prevention	2020-03-16	Yan Li et al A
Jamaica/JM-CDC-6729/2020	EPI_ISL_450798	Jamaica Ministry of Health and Wellness	Pathogen Discovery, Respiratory Viruses Branch, Division of Viral Diseases, Centers for Disease Control and Prevention	2020-03-16	Krista Queen et al A
Jamaica/JM-CDC-7286/2020	EPI_ISL_450799	Jamaica Ministry of Health and Wellness	Pathogen Discovery, Respiratory Viruses Branch, Division of Viral Diseases, Centers for Disease Control and Prevention	2020-03-17	Yan Li et al A
Japan/AI-I-004/2020	EPI_ISL_407084	Department of Virology III, National Institute of Infectious Diseases	Pathogen Genomics Center, National Institute of Infectious Diseases	2020-01-25	Tsuyoshi Sekizuka et al A
Japan/Donner1/2020	EPI_ISL_437601	Keio University School of Medicine	Keio University School of Medicine	2020-04-06	Kenjiro Kosaki et al A (https://dx.doi.org/10.1101/2020.05.21.20107599)
Japan/Donner11/2020	EPI_ISL_438955	Keio University School of Medicine	Keio University School of Medicine	2020-04-08	Kenjiro Kosaki et al A (https://dx.doi.org/10.1101/2020.05.21.20107599)

Japan/Donner12/2020	EPI_ISL_438956	Keio University School of Medicine	Keio University School of Medicine	2020-04-09	Kenjiro Kosaki et al A (https://dx.doi.org/10.1101/2020.05.21.20107599)
Japan/Donner14/2020	EPI_ISL_438958	Keio University School of Medicine	Keio University School of Medicine	2020-04-10	Kenjiro Kosaki et al A (https://dx.doi.org/10.1101/2020.05.21.20107599)
Japan/Donner15/2020	EPI_ISL_438959	Keio University School of Medicine	Keio University School of Medicine	2020-04-10	Kenjiro Kosaki et al A (https://dx.doi.org/10.1101/2020.05.21.20107599)
Japan/Donner17/2020	EPI_ISL_438961	Keio University School of Medicine	Keio University School of Medicine	2020-04-11	Kenjiro Kosaki et al A (https://dx.doi.org/10.1101/2020.05.21.20107599)
Japan/Donner18/2020	EPI_ISL_438962	Keio University School of Medicine	Keio University School of Medicine	2020-04-13	Kenjiro Kosaki et al A (https://dx.doi.org/10.1101/2020.05.21.20107599)
Japan/Donner19/2020	EPI_ISL_438963	Keio University School of Medicine	Keio University School of Medicine	2020-04-13	Kenjiro Kosaki et al A (https://dx.doi.org/10.1101/2020.05.21.20107599)
Japan/Donner2/2020	EPI_ISL_438947	Keio University School of Medicine	Keio University School of Medicine	2020-04-06	Kenjiro Kosaki et al A (https://dx.doi.org/10.1101/2020.05.21.20107599)
Japan/Donner20/2020	EPI_ISL_438964	Keio University School of Medicine	Keio University School of Medicine	2020-04-14	Kenjiro Kosaki et al A (https://dx.doi.org/10.1101/2020.05.21.20107599)
Japan/Donner21/2020	EPI_ISL_438965	Keio University School of Medicine	Keio University School of Medicine	2020-04-14	Kenjiro Kosaki et al A (https://dx.doi.org/10.1101/2020.05.21.20107599)
Japan/Donner22/2020	EPI_ISL_438966	Keio University School of Medicine	Keio University School of Medicine	2020-04-16	Kenjiro Kosaki et al A (https://dx.doi.org/10.1101/2020.05.21.20107599)
Japan/Donner23/2020	EPI_ISL_438967	Keio University School of Medicine	Keio University School of Medicine	2020-04-16	Kenjiro Kosaki et al A (https://dx.doi.org/10.1101/2020.05.21.20107599)
Japan/Donner24/2020	EPI_ISL_438968	Keio University School of Medicine	Keio University School of Medicine	2020-04-20	Kenjiro Kosaki et al A (https://dx.doi.org/10.1101/2020.05.21.20107599)
Japan/Donner25/2020	EPI_ISL_438969	Keio University School of Medicine	Keio University School of Medicine	2020-04-22	Kenjiro Kosaki et al A (https://dx.doi.org/10.1101/2020.05.21.20107599)
Japan/Donner26/2020	EPI_ISL_438970	Keio University School of Medicine	Keio University School of Medicine	2020-03-25	Kenjiro Kosaki et al A (https://dx.doi.org/10.1101/2020.05.21.20107599)
Japan/Donner27/2020	EPI_ISL_438971	Keio University School of Medicine	Keio University School of Medicine	2020-04-24	Kenjiro Kosaki et al A (https://dx.doi.org/10.1101/2020.05.21.20107599)
Japan/Donner28/2020	EPI_ISL_438972	Keio University School of Medicine	Keio University School of Medicine	2020-03-27	Kenjiro Kosaki et al A (https://dx.doi.org/10.1101/2020.05.21.20107599)
Japan/Donner29/2020	EPI_ISL_469287	Keio University Hospital	Keio University Hospital	2020-05-01	Kenjiro Kosaki et al B
Japan/Donner3/2020	EPI_ISL_438948	Keio University School of Medicine	Keio University School of Medicine	2020-04-06	Kenjiro Kosaki et al A (https://dx.doi.org/10.1101/2020.05.21.20107599)
Japan/Donner30/2020	EPI_ISL_469288	Keio University Hospital	Keio University Hospital	2020-05-01	Kenjiro Kosaki et al B
Japan/Donner31/2020	EPI_ISL_469289	Keio University Hospital	Keio University Hospital	2020-04-01	Kenjiro Kosaki et al B
Japan/Donner32/2020	EPI_ISL_469290	Keio University Hospital	Keio University Hospital	2020-05-03	Kenjiro Kosaki et al B
Japan/Donner33/2020	EPI_ISL_469291	Keio University Hospital	Keio University Hospital	2020-05-07	Kenjiro Kosaki et al B
Japan/Donner34/2020	EPI_ISL_469292	Keio University Hospital	Keio University Hospital	2020-05-07	Kenjiro Kosaki et al B
Japan/Donner35/2020	EPI_ISL_469293	Keio University Hospital	Keio University Hospital	2020-04-03	Kenjiro Kosaki et al B
Japan/Donner36/2020	EPI_ISL_469294	Keio University Hospital	Keio University Hospital	2020-05-11	Kenjiro Kosaki et al B
Japan/Donner37/2020	EPI_ISL_469295	Keio University Hospital	Keio University Hospital	2020-05-14	Kenjiro Kosaki et al B
Japan/Donner38/2020	EPI_ISL_469296	Keio University Hospital	Keio University Hospital	2020-04-15	Kenjiro Kosaki et al B
Japan/Donner4/2020	EPI_ISL_438949	Keio University School of Medicine	Keio University School of Medicine	2020-04-06	Kenjiro Kosaki et al A (https://dx.doi.org/10.1101/2020.05.21.20107599)
Japan/Donner6/2020	EPI_ISL_438951	Keio University School of Medicine	Keio University School of Medicine	2020-04-06	Kenjiro Kosaki et al A (https://dx.doi.org/10.1101/2020.05.21.20107599)
Japan/Donner7/2020	EPI_ISL_438952	Keio University School of Medicine	Keio University School of Medicine	2020-04-06	Kenjiro Kosaki et al A (https://dx.doi.org/10.1101/2020.05.21.20107599)
Japan/Donner8/2020	EPI_ISL_438953	Keio University School of Medicine	Keio University School of Medicine	2020-04-06	Kenjiro Kosaki et al A (https://dx.doi.org/10.1101/2020.05.21.20107599)
Japan/Donner9/2020	EPI_ISL_438954	Keio University School of Medicine	Keio University School of Medicine	2020-04-06	Kenjiro Kosaki et al A (https://dx.doi.org/10.1101/2020.05.21.20107599)
Japan/DP0027/2020	EPI_ISL_416566	Japanese Quarantine Stations	Pathogen Genomics Center, National Institute of Infectious Diseases	2020-02-15	Tsuyoshi Sekizuka et al C (https://dx.doi.org/10.1101/2020.03.23.20041970)
Japan/DP0037/2020	EPI_ISL_416567	Japanese Quarantine Stations	Pathogen Genomics Center, National Institute of Infectious Diseases	2020-02-15	Tsuyoshi Sekizuka et al C (https://dx.doi.org/10.1101/2020.03.23.20041970)
Japan/DP0134/2020	EPI_ISL_416577	Japanese Quarantine Stations	Pathogen Genomics Center, National Institute of Infectious Diseases	2020-02-15	Tsuyoshi Sekizuka et al C (https://dx.doi.org/10.1101/2020.03.23.20041970)
Japan/DP0152/2020	EPI_ISL_416578	Japanese Quarantine Stations	Pathogen Genomics Center, National Institute of Infectious Diseases	2020-02-15	Tsuyoshi Sekizuka et al C (https://dx.doi.org/10.1101/2020.03.23.20041970)
Japan/DP0184/2020	EPI_ISL_416580	Japanese Quarantine Stations	Pathogen Genomics Center, National Institute of Infectious Diseases	2020-02-15	Tsuyoshi Sekizuka et al C (https://dx.doi.org/10.1101/2020.03.23.20041970)
Japan/DP0191/2020	EPI_ISL_416582	Japanese Quarantine Stations	Pathogen Genomics Center, National Institute of Infectious Diseases	2020-02-15	Tsuyoshi Sekizuka et al C (https://dx.doi.org/10.1101/2020.03.23.20041970)
Japan/DP0278/2020	EPI_ISL_416587	Japanese Quarantine Stations	Pathogen Genomics Center, National Institute of Infectious Diseases	2020-02-16	Tsuyoshi Sekizuka et al C (https://dx.doi.org/10.1101/2020.03.23.20041970)
Japan/DP0287/2020	EPI_ISL_416589	Japanese Quarantine Stations	Pathogen Genomics Center, National Institute of Infectious Diseases	2020-02-16	Tsuyoshi Sekizuka et al C (https://dx.doi.org/10.1101/2020.03.23.20041970)
Japan/DP0290/2020	EPI_ISL_416591	Japanese Quarantine Stations	Pathogen Genomics Center, National Institute of Infectious Diseases	2020-02-16	Tsuyoshi Sekizuka et al C (https://dx.doi.org/10.1101/2020.03.23.20041970)
Japan/DP0311/2020	EPI_ISL_416593	Japanese Quarantine Stations	Pathogen Genomics Center, National Institute of Infectious Diseases	2020-02-16	Tsuyoshi Sekizuka et al C (https://dx.doi.org/10.1101/2020.03.23.20041970)
Japan/DP0319/2020	EPI_ISL_416594	Japanese Quarantine Stations	Pathogen Genomics Center, National Institute of Infectious Diseases	2020-02-16	Tsuyoshi Sekizuka et al C (https://dx.doi.org/10.1101/2020.03.23.20041970)
Japan/DP0321/2020	EPI_ISL_454749	Japanese Quarantine Stations	Pathogen Genomics Center, National Institute of Infectious Diseases	2020-02-16	Tsuyoshi Sekizuka et al A
Japan/DP0328/2020	EPI_ISL_416595	Japanese Quarantine Stations	Pathogen Genomics Center, National Institute of Infectious Diseases	2020-02-16	Tsuyoshi Sekizuka et al C (https://dx.doi.org/10.1101/2020.03.23.20041970)
Japan/DP0346/2020	EPI_ISL_416597	Japanese Quarantine Stations	Pathogen Genomics Center, National Institute of Infectious Diseases	2020-02-16	Tsuyoshi Sekizuka et al C (https://dx.doi.org/10.1101/2020.03.23.20041970)
Japan/DP0357/2020	EPI_ISL_416598	Japanese Quarantine Stations	Pathogen Genomics Center, National Institute of Infectious Diseases	2020-02-16	Tsuyoshi Sekizuka et al C (https://dx.doi.org/10.1101/2020.03.23.20041970)
Japan/DP0462/2020	EPI_ISL_416602	Japanese Quarantine Stations	Pathogen Genomics Center, National Institute of Infectious Diseases	2020-02-16	Tsuyoshi Sekizuka et al C (https://dx.doi.org/10.1101/2020.03.23.20041970)
Japan/DP0588/2020	EPI_ISL_416610	Japanese Quarantine Stations	Pathogen Genomics Center, National Institute of Infectious Diseases	2020-02-17	Tsuyoshi Sekizuka et al C (https://dx.doi.org/10.1101/2020.03.23.20041970)
Japan/DP0644/2020	EPI_ISL_416611	Japanese Quarantine Stations	Pathogen Genomics Center, National Institute of Infectious Diseases	2020-02-17	Tsuyoshi Sekizuka et al C (https://dx.doi.org/10.1101/2020.03.23.20041970)
Japan/DP0645/2020	EPI_ISL_416612	Japanese Quarantine Stations	Pathogen Genomics Center, National Institute of Infectious Diseases	2020-02-17	Tsuyoshi Sekizuka et al C (https://dx.doi.org/10.1101/2020.03.23.20041970)
Japan/DP0654/2020	EPI_ISL_416613	Japanese Quarantine Stations	Pathogen Genomics Center, National Institute of Infectious Diseases	2020-02-17	Tsuyoshi Sekizuka et al C (https://dx.doi.org/10.1101/2020.03.23.20041970)
Japan/DP0743/2020	EPI_ISL_416621	Japanese Quarantine Stations	Pathogen Genomics Center, National Institute of Infectious Diseases	2020-02-17	Tsuyoshi Sekizuka et al C (https://dx.doi.org/10.1101/2020.03.23.20041970)
Japan/DP0763/2020	EPI_ISL_416623	Japanese Quarantine Stations	Pathogen Genomics Center, National Institute of Infectious Diseases	2020-02-17	Tsuyoshi Sekizuka et al C (https://dx.doi.org/10.1101/2020.03.23.20041970)
Japan/DP0764/2020	EPI_ISL_416624	Japanese Quarantine Stations	Pathogen Genomics Center, National Institute of Infectious Diseases	2020-02-17	Tsuyoshi Sekizuka et al C (https://dx.doi.org/10.1101/2020.03.23.20041970)
Japan/DP0785/2020	EPI_ISL_416627	Japanese Quarantine Stations	Pathogen Genomics Center, National Institute of Infectious Diseases	2020-02-17	Tsuyoshi Sekizuka et al C (https://dx.doi.org/10.1101/2020.03.23.20041970)
Japan/DP0802/2020	EPI_ISL_416629	Japanese Quarantine Stations	Pathogen Genomics Center, National Institute of Infectious Diseases	2020-02-17	Tsuyoshi Sekizuka et al C (https://dx.doi.org/10.1101/2020.03.23.20041970)
Japan/DP0803/2020	EPI_ISL_416630	Japanese Quarantine Stations	Pathogen Genomics Center, National Institute of Infectious Diseases	2020-02-17	Tsuyoshi Sekizuka et al C (https://dx.doi.org/10.1101/2020.03.23.20041970)
Japan/DP0804/2020	EPI_ISL_416631	Japanese Quarantine Stations	Pathogen Genomics Center, National Institute of Infectious Diseases	2020-02-17	Tsuyoshi Sekizuka et al C (https://dx.doi.org/10.1101/2020.03.23.20041970)
Japan/DP0827/2020	EPI_ISL_416632	Japanese Quarantine Stations	Pathogen Genomics Center, National Institute of Infectious Diseases	2020-02-17	Tsuyoshi Sekizuka et al C (https://dx.doi.org/10.1101/2020.03.23.20041970)
Japan/Hu_DP_Kng_19-027/2020	EPI_ISL_412969		Takayuki Hishiki Kanagawa Prefectural Institute of Public Health, Department of Microbiology	2020-02-10	Hishiki et al B (https://dx.doi.org/10.1002/jmv.25773)
Japan/Hu_Kng_19-437/2020	EPI_ISL_420890		Takayuki Hishiki Kanagawa Prefectural Institute of Public Health	2020-03-09	Hishiki et al A
Japan/Hu_Kng_19-865/2020	EPI_ISL_445183		Takayuki Hishiki Kanagawa Prefectural Institute of Public Health	2020-03-23	Hishiki et al A
Japan/KY-V-029/2020	EPI_ISL_408669	Dept. of Virology III, National Institute of Infectious Diseases	Pathogen Genomics Center, National Institute of Infectious Diseases	2020-01-29	Tsuyoshi Sekizuka et al A
Japan/NA-20-05-1/2020	EPI_ISL_410531	Dept. of Pathology, National Institute of Infectious Diseases	Pathogen Genomics Center, National Institute of Infectious Diseases	2020-01-25	Tsuyoshi Sekizuka et al A
Japan/OS-20-07-1/2020	EPI_ISL_410532	Dept. of Pathology, National Institute of Infectious Diseases	Pathogen Genomics Center, National Institute of Infectious Diseases	2020-01-23	Tsuyoshi Sekizuka et al B (https://dx.doi.org/10.2807/1560-7917.ES.2020.25.13.2000305)
Japan/P1/2020	EPI_ISL_419296	Kochi Prefectural Institute of Public Health	Pathogen Genomics Center, National Institute of Infectious Diseases	2020-03-08	Tsuyoshi Sekizuka et al A
Japan/P2-1/2020	EPI_ISL_419297	Chiba Prefectural Institute of Public Health	Pathogen Genomics Center, National Institute of Infectious Diseases	2020-03-10	Tsuyoshi Sekizuka et al A
Japan/P2-2/2020	EPI_ISL_419298	Chiba Prefectural Institute of Public Health	Pathogen Genomics Center, National Institute of Infectious Diseases	2020-03-10	Tsuyoshi Sekizuka et al A
Japan/P3-1/2020	EPI_ISL_419299	Ishikawa Prefectural Institute of Public Health and Environmental Science	Pathogen Genomics Center, National Institute of Infectious Diseases	2020-03-09	Tsuyoshi Sekizuka et al A
Japan/P3-2/2020	EPI_ISL_419300	Ishikawa Prefectural Institute of Public Health and Environmental Science	Pathogen Genomics Center, National Institute of Infectious Diseases	2020-03-20	Tsuyoshi Sekizuka et al A
Japan/P4-1/2020	EPI_ISL_419301	Saitama Prefectural Institute of Public Health	Pathogen Genomics Center, National Institute of Infectious Diseases	2020-03-11	Tsuyoshi Sekizuka et al A
Japan/P4-2/2020	EPI_ISL_419302	Saitama Prefectural Institute of Public Health	Pathogen Genomics Center, National Institute of Infectious Diseases	2020-03-12	Tsuyoshi Sekizuka et al A
Japan/P4-3/2020	EPI_ISL_419303	Saitama Prefectural Institute of Public Health	Pathogen Genomics Center, National Institute of Infectious Diseases	2020-03-12	Tsuyoshi Sekizuka et al A
Japan/P4-4/2020	EPI_ISL_419304	Saitama Prefectural Institute of Public Health	Pathogen Genomics Center, National Institute of Infectious Diseases	2020-03-17	Tsuyoshi Sekizuka et al A
Japan/P4-5/2020	EPI_ISL_419305	Saitama Prefectural Institute of Public Health	Pathogen Genomics Center, National Institute of Infectious Diseases	2020-03-18	Tsuyoshi Sekizuka et al A
Japan/P4-6/2020	EPI_ISL_419306	Saitama Prefectural Institute of Public Health	Pathogen Genomics Center, National Institute of Infectious Diseases	2020-03-20	Tsuyoshi Sekizuka et al A
Japan/P4-7/2020	EPI_ISL_419307	Saitama Prefectural Institute of Public Health	Pathogen Genomics Center, National Institute of Infectious Diseases	2020-03-20	Tsuyoshi Sekizuka et al A
Japan/P4-8/2020	EPI_ISL_419308	Saitama Prefectural Institute of Public Health	Pathogen Genomics Center, National Institute of Infectious Diseases	2020-03-19	Tsuyoshi Sekizuka et al A
Japan/P5-1/2020	EPI_ISL_419309	Chiba Prefectural Institute of Public Health	Pathogen Genomics Center, National Institute of Infectious Diseases	2020-03-12	Tsuyoshi Sekizuka et al A
Japan/P5-2/2020	EPI_ISL_419310	Chiba Prefectural Institute of Public Health	Pathogen Genomics Center, National Institute of Infectious Diseases	2020-03-13	Tsuyoshi Sekizuka et al A
Japan/P5-3/2020	EPI_ISL_419311	Chiba Prefectural Institute of Public Health	Pathogen Genomics Center, National Institute of Infectious Diseases	2020-03-13	Tsuyoshi Sekizuka et al A
Japan/SMU-0311S2/2020	EPI_ISL_416524	Saitama Medical University Hospital	Saitama Medical University	2020-03-11	Kazuo Imai et al
Japan/SMU-0311S3/2020	EPI_ISL_416525	Saitama Medical University	Saitama Medical University	2020-03-11	Kazuo Imai et al
Japan/TK-20-31-3/2020	EPI_ISL_413459	Department of Pathology, Toshima Hospital	Pathogen Genomics Center, National Institute of Infectious Diseases	2020-02-20	Tsuyoshi Sekizuka et al A
Jiangsu/JS03/2020	EPI_ISL_411953	NHC Key laboratory of Enteric Pathogenic Microbiology, Institute of Pathogenic Microbiology	Jiangsu Provincial Center for Disease Control & Prevention	2020-01-24	Kangchen Zhao et al
Jordan/SR-032/2020	EPI_ISL_429992	Biolab Diagnostic Laboratories	Andersen lab at Scripps Research	2020-03-22	Issa Abu-Dayyeh et al
Jordan/SR-033/2020	EPI_ISL_429993	Biolab Diagnostic Laboratories	Andersen lab at Scripps Research	2020-03-16	Issa Abu-Dayyeh et al
Jordan/SR-0335/2020	EPI_ISL_450187	Biolab Diagnostic Laboratories	Andersen lab at Scripps Research	2020-04-08	Issa Abu-Dayyeh et al
Jordan/SR-0336/2020	EPI_ISL_450188	Biolab Diagnostic Laboratories	Andersen lab at Scripps Research	2020-04-06	Issa Abu-Dayyeh et al
Jordan/SR-034/2020	EPI_ISL_429994	Biolab Diagnostic Laboratories	Andersen lab at Scripps Research	2020-03-17	Issa Abu-Dayyeh et al
Jordan/SR-036/2020	EPI_ISL_429996	Biolab Diagnostic Laboratories	Andersen lab at Scripps Research	2020-03-23	Issa Abu-Dayyeh et al
Jordan/SR-039/2020	EPI_ISL_429998	Biolab Diagnostic Laboratories	Andersen lab at Scripps Research	2020-03-28	Issa Abu-Dayyeh et al
Jordan/SR-041/2020	EPI_ISL_429999	Biolab Diagnostic Laboratories	Andersen lab at Scripps Research	2020-03-17	Issa Abu-Dayyeh et al
Jordan/SR-043/2020	EPI_ISL_430001	Biolab Diagnostic Laboratories	Andersen lab at Scripps Research	2020-03-17	Issa Abu-Dayyeh et al
Jordan/SR-044/2020	EPI_ISL_430002	Biolab Diagnostic Laboratories	Andersen lab at Scripps Research	2020-03-16	Issa Abu-Dayyeh et al
Jordan/SR-045/2020	EPI_ISL_430003	Biolab Diagnostic Laboratories	Andersen lab at Scripps Research	2020-03-16	Issa Abu-Dayyeh et al
Jordan/SR-046/2020	EPI_ISL_430004	Biolab Diagnostic Laboratories	Andersen lab at Scripps Research	2020-03-17	Issa Abu-Dayyeh et al
Jordan/SR-047/2020	EPI_ISL_430005	Biolab Diagnostic Laboratories	Andersen lab at Scripps Research	2020-03-26	Issa Abu-Dayyeh et al
Jordan/SR-048/2020	EPI_ISL_430006	Biolab Diagnostic Laboratories	Andersen lab at Scripps Research	2020-03-23	Issa Abu-Dayyeh et al
Jordan/SR-049/2020	EPI_ISL_430007	Biolab Diagnostic Laboratories	Andersen lab at Scripps Research	2020-03-19	Issa Abu-Dayyeh et al
Jordan/SR-052/2020	EPI_ISL_430009	Biolab Diagnostic Laboratories	Andersen lab at Scripps Research	2020-03-30	Issa Abu-Dayyeh et al
Jordan/SR-054/2020	EPI_ISL_430012	Biolab Diagnostic Laboratories	Andersen lab at Scripps Research	2020-03-30	Issa Abu-Dayyeh et al
Jordan/SR-055/2020	EPI_ISL_430013	Biolab Diagnostic Laboratories	Andersen lab at Scripps Research	2020-04-02	Issa Abu-Dayyeh et al
Kazakhstan/16173/2020	EPI_ISL_454497	RSE "National Center for Biotechnology"	RSE "National Center for Biotechnology"	2020-05-14	Alexandr Shevtsov et al
Kazakhstan/16183/2020	EPI_ISL_454576	Laboratory of virology, National Center of Expertise	Laboratory of molecular-genetic research, National Center of Expertise, Kazakhstan National Center for Biotechnology, Kazakhstan	2020-04-14	Abdaliyev Askar et al
Kazakhstan/16537/2020	EPI_ISL_454499	RSE "National Center for Biotechnology"	RSE "National Center for Biotechnology"	2020-04-16	Alexandr Shevtsov et al

Kazakhstan/18148/2020	EPI_ISL_454579	Laboratory of virology, National Center of Expertise	Laboratory of molecular-genetic research, National Center of Expertise, Kazakhstan National Center for Biotechnology, Kazakhstan	2020-04-16	Abdaliyev Askar et al
Kazakhstan/18287/2020	EPI_ISL_454580	Laboratory of virology, National Center of Expertise	Laboratory of molecular-genetic research, National Center of Expertise, Kazakhstan National Center for Biotechnology, Kazakhstan	2020-04-16	Abdaliyev Askar et al
Kazakhstan/21399/2020	EPI_ISL_454502	RSE "National Center for Biotechnology"	RSE "National Center for Biotechnology"	2020-04-20	Alexandr Shevtsov et al
Kazakhstan/22044/2020	EPI_ISL_454584	Laboratory of virology, National Center of Expertise	Laboratory of molecular-genetic research, National Center for Expertise, Kazakhstan National Center for Biotechnology, Kazakhstan	2020-04-21	Abdaliyev Askar et al
Kazakhstan/22517/2020	EPI_ISL_454586	Laboratory of virology, National Center of Expertise	Laboratory of molecular-genetic research, National Center for Expertise, Kazakhstan National Center for Biotechnology, Kazakhstan	2020-04-21	Abdaliyev Askar et al
Kazakhstan/26473/2020	EPI_ISL_454504	RSE "National Center for Biotechnology"	RSE "National Center for Biotechnology"	2020-04-26	Alexandr Shevtsov et al
Kazakhstan/26474/2020	EPI_ISL_454505	RSE "National Center for Biotechnology"	RSE "National Center for Biotechnology"	2020-04-26	Alexandr Shevtsov et al
Kazakhstan/26489/2020	EPI_ISL_454507	RSE "National Center for Biotechnology"	RSE "National Center for Biotechnology"	2020-04-26	Alexandr Shevtsov et al
Kazakhstan/26491/2020	EPI_ISL_454508	RSE "National Center for Biotechnology"	RSE "National Center for Biotechnology"	2020-04-19	Alexandr Shevtsov et al
Kazakhstan/26497/2020	EPI_ISL_454509	RSE "National Center for Biotechnology"	RSE "National Center for Biotechnology"	2020-04-26	Alexandr Shevtsov et al
Kazakhstan/26501/2020	EPI_ISL_454510	RSE "National Center for Biotechnology"	RSE "National Center for Biotechnology"	2020-04-26	Alexandr Shevtsov et al
Kazakhstan/26506/2020	EPI_ISL_454511	RSE "National Center for Biotechnology"	RSE "National Center for Biotechnology"	2020-04-26	Alexandr Shevtsov et al
Kazakhstan/26508/2020	EPI_ISL_454589	Laboratory of virology, National Center of Expertise	Laboratory of molecular-genetic research, National Center for Expertise, Kazakhstan National Center for Biotechnology, Kazakhstan	2020-04-26	Abdaliyev Askar et al
Kazakhstan/26530/2020	EPI_ISL_454590	Laboratory of virology, National Center of Expertise	Laboratory of molecular-genetic research, National Center of Expertise, Kazakhstan National Center for Biotechnology, Kazakhstan	2020-04-26	Abdaliyev Askar et al
Kazakhstan/26545/2020	EPI_ISL_454591	Laboratory of virology, National Center of Expertise	Laboratory of molecular-genetic research, National Center of Expertise, Kazakhstan National Center for Biotechnology, Kazakhstan	2020-04-26	Abdaliyev Askar et al
Kazakhstan/26548/2020	EPI_ISL_454512	RSE "National Center for Biotechnology"	RSE "National Center for Biotechnology"	2020-04-26	Alexandr Shevtsov et al
Kazakhstan/26549/2020	EPI_ISL_454513	RSE "National Center for Biotechnology"	RSE "National Center for Biotechnology"	2020-04-26	Alexandr Shevtsov et al
Kazakhstan/26568/2020	EPI_ISL_454601	Laboratory of virology, National Center of Expertise	Laboratory of molecular-genetic research, National Center of Expertise, Kazakhstan National Center for Biotechnology, Kazakhstan	2020-04-26	Abdaliyev Askar et al
Kazakhstan/26574/2020	EPI_ISL_454593	Laboratory of virology, National Center of Expertise	Laboratory of molecular-genetic research, National Center of Expertise, Kazakhstan National Center for Biotechnology, Kazakhstan	2020-04-26	Abdaliyev Askar et al
Kazakhstan/26579/2020	EPI_ISL_454603	Laboratory of virology, National Center of Expertise	Laboratory of molecular-genetic research, National Center of Expertise, Kazakhstan National Center for Biotechnology, Kazakhstan	2020-04-26	Abdaliyev Askar et al
Kazakhstan/26580/2020	EPI_ISL_454604	Laboratory of virology, National Center of Expertise	Laboratory of molecular-genetic research, National Center for Expertise, Kazakhstan National Center for Biotechnology, Kazakhstan	2020-04-26	Abdaliyev Askar et al
Kazakhstan/26584/2020	EPI_ISL_454598	Laboratory of virology, National Center of Expertise	Laboratory of molecular-genetic research, National Center of Expertise, Kazakhstan National Center for Biotechnology, Kazakhstan	2020-04-26	Abdaliyev Askar et al
Kazakhstan/26585/2020	EPI_ISL_454514	RSE "National Center for Biotechnology"	RSE "National Center for Biotechnology"	2020-04-26	Alexandr Shevtsov et al
Kazakhstan/26617/2020	EPI_ISL_454515	RSE "National Center for Biotechnology"	RSE "National Center for Biotechnology"	2020-04-26	Alexandr Shevtsov et al
Kazakhstan/26827/2020	EPI_ISL_454516	RSE "National Center for Biotechnology"	RSE "National Center for Biotechnology"	2020-04-26	Alexandr Shevtsov et al
Kazakhstan/26829/2020	EPI_ISL_454518	RSE "National Center for Biotechnology"	RSE "National Center for Biotechnology"	2020-04-26	Alexandr Shevtsov et al
Kazakhstan/33496/2020	EPI_ISL_454585	Laboratory of virology, National Center of Expertise	Laboratory of molecular-genetic research, National Center for Expertise, Kazakhstan National Center for Biotechnology, Kazakhstan	2020-05-04	Abdaliyev Askar et al
Kazakhstan/38716/2020	EPI_ISL_454575	Laboratory of virology, National Center of Expertise	Laboratory of molecular-genetic research, National Center for Expertise, Kazakhstan National Center for Biotechnology, Kazakhstan	2020-05-09	Abdaliyev Askar et al
Kazakhstan/7263/2020	EPI_ISL_454571	National Center of Expertise	National Center for Expertise, National Center for Biotechnology, Kazakhstan	2020-03-25	Abdaliyev Askar et al
Kazakhstan/7341/2020	EPI_ISL_454572	National Center of Expertise	National Center for Expertise, Kazakhstan National Center for Biotechnology, Kazakhstan	2020-03-25	Abdaliyev Askar et al
Kazakhstan/NCB-1/2020	EPI_ISL_435045	Laboratory of Applied Genetics	RSE "National Center for Biotechnology"	2020-03-22	Shevtsov et al
Kazakhstan/NCB-2/2020	EPI_ISL_435046	Laboratory of Applied Genetics	RSE "National Center for Biotechnology"	2020-03-25	Shevtsov et al
Kazakhstan/NCB-3/2020	EPI_ISL_435047	Laboratory of Applied Genetics	RSE "National Center for Biotechnology"	2020-03-25	Shevtsov et al
Kazakhstan/NCB-5/2020	EPI_ISL_435048	Laboratory of Applied Genetics	RSE "National Center for Biotechnology"	2020-04-21	Shevtsov et al
Kenya/D5/2020	EPI_ISL_457999		Centre For Biotechnology Research and Development	2020-05-22	Matoke-Muhia et al
Korea/KCDC2004/2020	EPI_ISL_426164	Division of Viral Diseases, Center for Laboratory Control of Infectious Diseases, Korea Centers for Diseases Control and Prevention	Division of Viral Diseases, Center for Laboratory Control of Infectious Diseases, Korea Centers for Diseases Control and Prevention	2020-02-02	Jeong-Min Kim et al A
Korea/KCDC2005/2020	EPI_ISL_426166	Division of Viral Diseases, Center for Laboratory Control of Infectious Diseases, Korea Centers for Diseases Control and Prevention	Division of Viral Diseases, Center for Laboratory Control of Infectious Diseases, Korea Centers for Diseases Control and Prevention	2020-02-04	Jeong-Min Kim et al A
Korea/KCDC2007/2020	EPI_ISL_426169	Division of Viral Diseases, Center for Laboratory Control of Infectious Diseases, Korea Centers for Diseases Control and Prevention	Division of Viral Diseases, Center for Laboratory Control of Infectious Diseases, Korea Centers for Diseases Control and Prevention	2020-02-05	Jeong-Min Kim et al A
Kuwait/KU001/2020	EPI_ISL_421652	Dasman Diabetes Institute	Dasman Diabetes Institute	2020-03-16	Fahd Al-Mulla et al
Kuwait/KU005/2020	EPI_ISL_422426	JABER AL AHMAD AL SABAH HOSPITAL – KUWAIT CITY	Dasman Diabetes Institute	2020-03-16	Fahd Al-Mulla et al
Kuwait/KU006/2020	EPI_ISL_422424	Jaber Al Ahmad Al Sabah Hospital	Dasman diabetes Institute	2020-03-15	Fahd Al-Mulla et al
Kuwait/KU008/2020	EPI_ISL_422427	JABER AL AHMAD AL SABAH HOSPITAL – KUWAIT CITY	Dasman Diabetes Institute	2020-03-16	Fahd Al-Mulla et al
Kuwait/KU09/2020	EPI_ISL_416541	Dasman Diabetes Institute and Virology Laboratory Ministry of Health	Dasman Diabetes Institute	2020-03-02	Fahd Al-Mulla et al
Kuwait/KU12/2020	EPI_ISL_416458	Virology laboratory Ministry of Health Kuwait sequenced at Dasman Diabetes Institute	Dasman Diabetes Institute	2020-03-02	Fahd Al-Mulla et al
Kuwait/KU17/2020	EPI_ISL_416542	Dasman Diabetes Institute	Dasman Diabetes Institute	2020-03-02	Fahd Al-Mulla et al
Kuwait/KU18/2020	EPI_ISL_416543	Dasman Diabetes Institute	Dasman Diabetes Institute	2020-03-02	Fahd Al-Mulla et al
Latvia/01/2020	EPI_ISL_421653	E. Gulbja Laboratorija	Latvian Biomedical Research and Study Centre	2020-03-25	Ivars Silamiķelis et al
Latvia/010/2020	EPI_ISL_437089	Latvijas Infektoloģijas centrs	Latvian Biomedical Research and Study Centre	2020-03-23	Ivars Silamiķelis et al
Latvia/011/2020	EPI_ISL_437090	Latvijas Infektoloģijas centrs	Latvian Biomedical Research and Study Centre	2020-03-24	Ivars Silamiķelis et al
Latvia/012/2020	EPI_ISL_437091	Latvijas Infektoloģijas centrs	Latvian Biomedical Research and Study Centre	2020-03-23	Ivars Silamiķelis et al
Latvia/013/2020	EPI_ISL_437092	Latvijas Infektoloģijas centrs	Latvian Biomedical Research and Study Centre	2020-03-20	Ivars Silamiķelis et al
Latvia/014/2020	EPI_ISL_437093	Latvijas Infektoloģijas centrs	Latvian Biomedical Research and Study Centre	2020-03-22	Ivars Silamiķelis et al
Latvia/015/2020	EPI_ISL_437094	Latvijas Infektoloģijas centrs	Latvian Biomedical Research and Study Centre	2020-03-23	Ivars Silamiķelis et al
Latvia/016/2020	EPI_ISL_437095	Latvijas Infektoloģijas centrs	Latvian Biomedical Research and Study Centre	2020-03-23	Ivars Silamiķelis et al
Latvia/017/2020	EPI_ISL_437096	Latvijas Infektoloģijas centrs	Latvian Biomedical Research and Study Centre	2020-03-23	Ivars Silamiķelis et al
Latvia/018/2020	EPI_ISL_450518	E. Gulbja Laboratorija	Latvian Biomedical Research and Study Centre	2020-04-18	Ivars Silamiķelis et al
Latvia/019/2020	EPI_ISL_450519	E. Gulbja Laboratorija	Latvian Biomedical Research and Study Centre	2020-04-14	Ivars Silamiķelis et al
Latvia/02/2020	EPI_ISL_421654	E. Gulbja Laboratorija	Latvian Biomedical Research and Study Centre	2020-03-25	Ivars Silamiķelis et al
Latvia/020/2020	EPI_ISL_450520	Centrālā Laboratorija	Latvian Biomedical Research and Study Centre	2020-04-28	Ivars Silamiķelis et al
Latvia/021/2020	EPI_ISL_450521	Centrālā Laboratorija	Latvian Biomedical Research and Study Centre	2020-04-28	Ivars Silamiķelis et al
Latvia/022/2020	EPI_ISL_450522	Centrālā Laboratorija	Latvian Biomedical Research and Study Centre	2020-04-28	Ivars Silamiķelis et al
Latvia/023/2020	EPI_ISL_450523	Centrālā Laboratorija	Latvian Biomedical Research and Study Centre	2020-04-28	Ivars Silamiķelis et al
Latvia/024/2020	EPI_ISL_450524	Centrālā Laboratorija	Latvian Biomedical Research and Study Centre	2020-04-19	Ivars Silamiķelis et al
Latvia/03/2020	EPI_ISL_421655	E. Gulbja Laboratorija	Latvian Biomedical Research and Study Centre	2020-03-25	Ivars Silamiķelis et al
Latvia/04/2020	EPI_ISL_421656	E. Gulbja Laboratorija	Latvian Biomedical Research and Study Centre	2020-03-25	Ivars Silamiķelis et al
Latvia/05/2020	EPI_ISL_426285	E. Gulbja Laboratorija	Latvian Biomedical Research and Study Centre	2020-03-30	Ivars Silamiķelis et al
Latvia/06/2020	EPI_ISL_426286	E. Gulbja Laboratorija	Latvian Biomedical Research and Study Centre	2020-03-30	Ivars Silamiķelis et al
Latvia/07/2020	EPI_ISL_426287	E. Gulbja Laboratorija	Latvian Biomedical Research and Study Centre	2020-03-30	Ivars Silamiķelis et al
Latvia/08/2020	EPI_ISL_426288	E. Gulbja Laboratorija	Latvian Biomedical Research and Study Centre	2020-03-30	Ivars Silamiķelis et al
Latvia/09/2020	EPI_ISL_426289	E. Gulbja Laboratorija	Latvian Biomedical Research and Study Centre	2020-03-30	Ivars Silamiķelis et al
Lebanon/S1_758/2020	EPI_ISL_450508	Rafik Hariri University Hospital	Rafik Hariri University Hospital	2020-03-04	Rita Feghali et al
Lebanon/S10_768/2020	EPI_ISL_450516	Rafik Hariri University Hospital	Rafik Hariri University Hospital	2020-03-15	Rita Feghali et al
Lebanon/S2_759/2020	EPI_ISL_450509	Rafik Hariri University Hospital	Rafik Hariri University Hospital	2020-03-15	Rita Feghali et al
Lebanon/S4_761/2020	EPI_ISL_450511	Rafik Hariri University Hospital	Rafik Hariri University Hospital	2020-03-04	Rita Feghali et al
Lebanon/S5_762/2020	EPI_ISL_450512	Rafik Hariri University Hospital	Rafik Hariri University Hospital	2020-02-27	Rita Feghali et al
Lebanon/S7_763/2020	EPI_ISL_454420	Rafik Hariri University Hospital	Rafik Hariri University Hospital	2020-02-21	Rita Feghali et al
Lebanon/S8_767/2020	EPI_ISL_450514	Rafik Hariri University Hospital	Rafik Hariri University Hospital	2020-03-13	Rita Feghali et al
Lebanon/S9_764/2020	EPI_ISL_450515	Rafik Hariri University Hospital	Rafik Hariri University Hospital	2020-03-11	Rita Feghali et al
Luxembourg/LNS0000001/2020	EPI_ISL_419562	Laboratoire National de Santé, Microbiology, Virology	Laboratoire National de Santé, Microbiology, Epidemiology and Microbial Genomics	2020-02-29	Anke Wienecke-Baldacchino et al
Luxembourg/LNS0102413/2020	EPI_ISL_428955	Laboratoire National de Sante, Microbiology, Virology	Laboratoire National de Sante, Microbiology, Epidemiology and Microbial Genomics	2020-04-01	Anke Wienecke-Baldacchino et al
Luxembourg/LNS0113568/2020	EPI_ISL_428942	Laboratoire National de Sante, Microbiology, Virology	Laboratoire National de Sante, Microbiology, Epidemiology and Microbial Genomics	2020-04-04	Anke Wienecke-Baldacchino et al
Luxembourg/LNS0156959/2020	EPI_ISL_419563	Laboratoire National de Santé, Microbiology, Virology	Laboratoire National de Santé, Microbiology, Epidemiology and Microbial Genomics	2020-03-12	Anke Wienecke-Baldacchino et al
Luxembourg/LNS0188413/2020	EPI_ISL_434495	Laboratoire National de Sante, Microbiology, Virology	Laboratoire National de Sante, Microbiology, Epidemiology and Microbial Genomics	2020-04-12	Anke Wienecke-Baldacchino et al
Luxembourg/LNS0364174/2020	EPI_ISL_434503	Laboratoire National de Sante, Microbiology, Virology	Laboratoire National de Sante, Microbiology, Epidemiology and Microbial Genomics	2020-04-12	Anke Wienecke-Baldacchino et al
Luxembourg/LNS0516520/2020	EPI_ISL_428952	Laboratoire National de Sante, Microbiology, Virology	Laboratoire National de Sante, Microbiology, Epidemiology and Microbial Genomics	2020-04-01	Anke Wienecke-Baldacchino et al
Luxembourg/LNS0681882/2020	EPI_ISL_459893	Laboratoire National de Sante, Microbiology, Virology	Laboratoire National de Sante, Microbiology, Epidemiology and Microbial Genomics	2020-05-15	Anke Wienecke-Baldacchino et al
Luxembourg/LNS0687874/2020	EPI_ISL_429797	Laboratoire National de Sante, Microbiology, Virology	Laboratoire National de Sante, Microbiology, Epidemiology and Microbial Genomics	2020-03-30	Anke Wienecke-Baldacchino et al
Luxembourg/LNS0716877/2020	EPI_ISL_419567	Laboratoire National de Santé, Microbiology, Virology	Laboratoire National de Santé, Microbiology, Epidemiology and Microbial Genomics	2020-03-15	Anke Wienecke-Baldacchino et al
Luxembourg/LNS0928727/2020	EPI_ISL_434494	Laboratoire National de Sante, Microbiology, Virology	Laboratoire National de Sante, Microbiology, Epidemiology and Microbial Genomics	2020-04-12	Anke Wienecke-Baldacchino et al
Luxembourg/LNS0972189/2020	EPI_ISL_434487	Laboratoire National de Sante, Microbiology, Virology	Laboratoire National de Sante, Microbiology, Epidemiology and Microbial Genomics	2020-04-13	Anke Wienecke-Baldacchino et al
Luxembourg/LNS1234709/2020	EPI_ISL_419570	Laboratoire National de Santé, Microbiology, Virology	Laboratoire National de Santé, Microbiology, Epidemiology and Microbial Genomics	2020-03-12	Anke Wienecke-Baldacchino et al
Luxembourg/LNS1586475/2020	EPI_ISL_445075	Laboratoire National de Sante, Microbiology, Virology	Laboratoire National de Sante, Microbiology, Epidemiology and Microbial Genomics	2020-05-08	Anke Wienecke-Baldacchino et al
Luxembourg/LNS1644489/2020	EPI_ISL_421740	Laboratoire National de Sante, Microbiology, Virology	Laboratoire National de Sante, Microbiology, Epidemiology and Microbial Genomics	2020-03-17	Anke Wienecke-Baldacchino et al
Luxembourg/LNS1646752/2020	EPI_ISL_459894	Laboratoire National de Sante, Microbiology, Virology	Laboratoire National de Sante, Microbiology, Epidemiology and Microbial Genomics	2020-05-12	Anke Wienecke-Baldacchino et al
Luxembourg/LNS1890202/2020	EPI_ISL_445068	Laboratoire National de Sante, Microbiology, Virology	Laboratoire National de Sante, Microbiology, Epidemiology and Microbial Genomics	2020-04-28	Anke Wienecke-Baldacchino et al
Luxembourg/LNS1918658/2020	EPI_ISL_419575	Laboratoire National de Santé, Microbiology, Virology	Laboratoire National de Santé, Microbiology, Epidemiology and Microbial Genomics	2020-03-14	Anke Wienecke-Baldacchino et al
Luxembourg/LNS2129030/2020	EPI_ISL_445073	Laboratoire National de Sante, Microbiology, Virology	Laboratoire National de Sante, Microbiology, Epidemiology and Microbial Genomics	2020-04-23	Anke Wienecke-Baldacchino et al
Luxembourg/LNS2151006/2020	EPI_ISL_419576	Laboratoire National de Santé, Microbiology, Virology	Laboratoire National de Santé, Microbiology, Epidemiology and Microbial Genomics	2020-03-17	Anke Wienecke-Baldacchino et al
Luxembourg/LNS2435702/2020	EPI_ISL_434511	Laboratoire National de Sante, Microbiology, Virology	Laboratoire National de Sante, Microbiology, Epidemiology and Microbial Genomics	2020-04-21	Anke Wienecke-Baldacchino et al
Luxembourg/LNS2458393/2020	EPI_ISL_434504	Laboratoire National de Sante, Microbiology, Virology	Laboratoire National de Sante, Microbiology, Epidemiology and Microbial Genomics	2020-04-14	Anke Wienecke-Baldacchino et al

Luxembourg/LNS2614631/2020	EPI_ISL_419578	Laboratoire National de Santé, Microbiology, Virology	Laboratoire National de Santé, Microbiology, Epidemiology and Microbial Genomics	2020-03-08	Anke Wienecke-Baldacchino et al
Luxembourg/LNS2629386/2020	EPI_ISL_434514	Laboratoire National de Sante, Microbiology, Virology	Laboratoire National de Sante, Microbiology, Epidemiology and Microbial Genomics	2020-04-16	Anke Wienecke-Baldacchino et al
Luxembourg/LNS2818424/2020	EPI_ISL_434500	Laboratoire National de Sante, Microbiology, Virology	Laboratoire National de Sante, Microbiology, Epidemiology and Microbial Genomics	2020-04-15	Anke Wienecke-Baldacchino et al
Luxembourg/LNS3029697/2020	EPI_ISL_445069	Laboratoire National de Sante, Microbiology, Virology	Laboratoire National de Sante, Microbiology, Epidemiology and Microbial Genomics	2020-04-26	Anke Wienecke-Baldacchino et al
Luxembourg/LNS3035957/2020	EPI_ISL_421750	Laboratoire National de Sante, Microbiology, Virology	Laboratoire National de Sante, Microbiology, Epidemiology and Microbial Genomics	2020-03-17	Anke Wienecke-Baldacchino et al
Luxembourg/LNS3059908/2020	EPI_ISL_459895	Laboratoire National de Sante, Microbiology, Virology	Laboratoire National de Sante, Microbiology, Epidemiology and Microbial Genomics	2020-05-18	Anke Wienecke-Baldacchino et al
Luxembourg/LNS3283768/2020	EPI_ISL_429787	Laboratoire National de Sante, Microbiology, Virology	Laboratoire National de Sante, Microbiology, Epidemiology and Microbial Genomics	2020-03-22	Anke Wienecke-Baldacchino et al
Luxembourg/LNS3660996/2020	EPI_ISL_459896	Laboratoire National de Sante, Microbiology, Virology	Laboratoire National de Sante, Microbiology, Epidemiology and Microbial Genomics	2020-05-14	Anke Wienecke-Baldacchino et al
Luxembourg/LNS3702558/2020	EPI_ISL_428945	Laboratoire National de Sante, Microbiology, Virology	Laboratoire National de Sante, Microbiology, Epidemiology and Microbial Genomics	2020-04-03	Anke Wienecke-Baldacchino et al
Luxembourg/LNS3770609/2020	EPI_ISL_421747	Laboratoire National de Sante, Microbiology, Virology	Laboratoire National de Sante, Microbiology, Epidemiology and Microbial Genomics	2020-03-17	Anke Wienecke-Baldacchino et al
Luxembourg/LNS3923316/2020	EPI_ISL_459898	Laboratoire National de Sante, Microbiology, Virology	Laboratoire National de Sante, Microbiology, Epidemiology and Microbial Genomics	2020-05-13	Anke Wienecke-Baldacchino et al
Luxembourg/LNS4028260/2020	EPI_ISL_434510	Laboratoire National de Sante, Microbiology, Virology	Laboratoire National de Sante, Microbiology, Epidemiology and Microbial Genomics	2020-04-13	Anke Wienecke-Baldacchino et al
Luxembourg/LNS4134806/2020	EPI_ISL_419586	Laboratoire National de Santé, Microbiology, Virology	Laboratoire National de Santé, Microbiology, Epidemiology and Microbial Genomics	2020-03-14	Anke Wienecke-Baldacchino et al
Luxembourg/LNS4423730/2020	EPI_ISL_429733	Laboratoire National de Sante, Microbiology, Virology	Laboratoire National de Sante, Microbiology, Epidemiology and Microbial Genomics	2020-03-22	Anke Wienecke-Baldacchino et al
Luxembourg/LNS4535024/2020	EPI_ISL_445063	Laboratoire National de Sante, Microbiology, Virology	Laboratoire National de Sante, Microbiology, Epidemiology and Microbial Genomics	2020-04-26	Anke Wienecke-Baldacchino et al
Luxembourg/LNS4719278/2020	EPI_ISL_429790	Laboratoire National de Sante, Microbiology, Virology	Laboratoire National de Sante, Microbiology, Epidemiology and Microbial Genomics	2020-03-27	Anke Wienecke-Baldacchino et al
Luxembourg/LNS4757989/2020	EPI_ISL_434498	Laboratoire National de Sante, Microbiology, Virology	Laboratoire National de Sante, Microbiology, Epidemiology and Microbial Genomics	2020-04-15	Anke Wienecke-Baldacchino et al
Luxembourg/LNS4845603/2020	EPI_ISL_419589	Laboratoire National de Santé, Microbiology, Virology	Laboratoire National de Santé, Microbiology, Epidemiology and Microbial Genomics	2020-03-15	Anke Wienecke-Baldacchino et al
Luxembourg/LNS4885848/2020	EPI_ISL_428941	Laboratoire National de Sante, Microbiology, Virology	Laboratoire National de Sante, Microbiology, Epidemiology and Microbial Genomics	2020-04-03	Anke Wienecke-Baldacchino et al
Luxembourg/LNS5032431/2020	EPI_ISL_419590	Laboratoire National de Santé, Microbiology, Virology	Laboratoire National de Santé, Microbiology, Epidemiology and Microbial Genomics	2020-03-11	Anke Wienecke-Baldacchino et al
Luxembourg/LNS5041225/2020	EPI_ISL_429710	Laboratoire National de Sante, Microbiology, Virology	Laboratoire National de Sante, Microbiology, Epidemiology and Microbial Genomics	2020-03-28	Anke Wienecke-Baldacchino et al
Luxembourg/LNS5444595/2020	EPI_ISL_428957	Laboratoire National de Sante, Microbiology, Virology	Laboratoire National de Sante, Microbiology, Epidemiology and Microbial Genomics	2020-04-07	Anke Wienecke-Baldacchino et al
Luxembourg/LNS5507695/2020	EPI_ISL_459899	Laboratoire National de Sante, Microbiology, Virology	Laboratoire National de Sante, Microbiology, Epidemiology and Microbial Genomics	2020-05-15	Anke Wienecke-Baldacchino et al
Luxembourg/LNS5549490/2020	EPI_ISL_429740	Laboratoire National de Sante, Microbiology, Virology	Laboratoire National de Sante, Microbiology, Epidemiology and Microbial Genomics	2020-03-25	Anke Wienecke-Baldacchino et al
Luxembourg/LNS5677421/2020	EPI_ISL_429784	Laboratoire National de Sante, Microbiology, Virology	Laboratoire National de Sante, Microbiology, Epidemiology and Microbial Genomics	2020-03-27	Anke Wienecke-Baldacchino et al
Luxembourg/LNS5742995/2020	EPI_ISL_445055	Laboratoire National de Sante, Microbiology, Virology	Laboratoire National de Sante, Microbiology, Epidemiology and Microbial Genomics	2020-04-22	Anke Wienecke-Baldacchino et al
Luxembourg/LNS6282845/2020	EPI_ISL_417533	Laboratoire Nationale de Santé, Microbiology, Virology	Laboratoire Nationale de Santé, Microbiology, Epidemiology and Microbial Genomics	2020-03-18	Anke Wienecke-Baldacchino et al
Luxembourg/LNS6326231/2020	EPI_ISL_428960	Laboratoire National de Sante, Microbiology, Virology	Laboratoire National de Sante, Microbiology, Epidemiology and Microbial Genomics	2020-04-02	Anke Wienecke-Baldacchino et al
Luxembourg/LNS6387933/2020	EPI_ISL_459900	Laboratoire National de Sante, Microbiology, Virology	Laboratoire National de Sante, Microbiology, Epidemiology and Microbial Genomics	2020-05-15	Anke Wienecke-Baldacchino et al
Luxembourg/LNS6469209/2020	EPI_ISL_459901	Laboratoire National de Sante, Microbiology, Virology	Laboratoire National de Sante, Microbiology, Epidemiology and Microbial Genomics	2020-05-19	Anke Wienecke-Baldacchino et al
Luxembourg/LNS6546078/2020	EPI_ISL_445053	Laboratoire National de Sante, Microbiology, Virology	Laboratoire National de Sante, Microbiology, Epidemiology and Microbial Genomics	2020-04-30	Anke Wienecke-Baldacchino et al
Luxembourg/LNS6733162/2020	EPI_ISL_459902	Laboratoire National de Sante, Microbiology, Virology	Laboratoire National de Sante, Microbiology, Epidemiology and Microbial Genomics	2020-05-19	Anke Wienecke-Baldacchino et al
Luxembourg/LNS6783622/2020	EPI_ISL_429734	Laboratoire National de Sante, Microbiology, Virology	Laboratoire National de Sante, Microbiology, Epidemiology and Microbial Genomics	2020-03-20	Anke Wienecke-Baldacchino et al
Luxembourg/LNS6854244/2020	EPI_ISL_428956	Laboratoire National de Sante, Microbiology, Virology	Laboratoire National de Sante, Microbiology, Epidemiology and Microbial Genomics	2020-04-01	Anke Wienecke-Baldacchino et al
Luxembourg/LNS6862754/2020	EPI_ISL_459903	Laboratoire National de Sante, Microbiology, Virology	Laboratoire National de Sante, Microbiology, Epidemiology and Microbial Genomics	2020-05-19	Anke Wienecke-Baldacchino et al
Luxembourg/LNS7188652/2020	EPI_ISL_445071	Laboratoire National de Sante, Microbiology, Virology	Laboratoire National de Sante, Microbiology, Epidemiology and Microbial Genomics	2020-04-23	Anke Wienecke-Baldacchino et al
Luxembourg/LNS7342327/2020	EPI_ISL_459905	Laboratoire National de Sante, Microbiology, Virology	Laboratoire National de Sante, Microbiology, Epidemiology and Microbial Genomics	2020-05-12	Anke Wienecke-Baldacchino et al
Luxembourg/LNS7721101/2020	EPI_ISL_429769	Laboratoire National de Sante, Microbiology, Virology	Laboratoire National de Sante, Microbiology, Epidemiology and Microbial Genomics	2020-03-28	Anke Wienecke-Baldacchino et al
Luxembourg/LNS7820945/2020	EPI_ISL_459906	Laboratoire National de Sante, Microbiology, Virology	Laboratoire National de Sante, Microbiology, Epidemiology and Microbial Genomics	2020-05-18	Anke Wienecke-Baldacchino et al
Luxembourg/LNS8167314/2020	EPI_ISL_429758	Laboratoire National de Sante, Microbiology, Virology	Laboratoire National de Sante, Microbiology, Epidemiology and Microbial Genomics	2020-03-24	Anke Wienecke-Baldacchino et al
Luxembourg/LNS8258882/2020	EPI_ISL_434491	Laboratoire National de Sante, Microbiology, Virology	Laboratoire National de Sante, Microbiology, Epidemiology and Microbial Genomics	2020-04-14	Anke Wienecke-Baldacchino et al
Luxembourg/LNS8545083/2020	EPI_ISL_429709	Laboratoire National de Sante, Microbiology, Virology	Laboratoire National de Sante, Microbiology, Epidemiology and Microbial Genomics	2020-03-19	Anke Wienecke-Baldacchino et al
Luxembourg/LNS8655659/2020	EPI_ISL_434492	Laboratoire National de Sante, Microbiology, Virology	Laboratoire National de Sante, Microbiology, Epidemiology and Microbial Genomics	2020-04-12	Anke Wienecke-Baldacchino et al
Luxembourg/LNS8705424/2020	EPI_ISL_445057	Laboratoire National de Sante, Microbiology, Virology	Laboratoire National de Sante, Microbiology, Epidemiology and Microbial Genomics	2020-04-23	Anke Wienecke-Baldacchino et al
Luxembourg/LNS8772889/2020	EPI_ISL_429755	Laboratoire National de Sante, Microbiology, Virology	Laboratoire National de Sante, Microbiology, Epidemiology and Microbial Genomics	2020-03-29	Anke Wienecke-Baldacchino et al
Luxembourg/LNS9063347/2020	EPI_ISL_445067	Laboratoire National de Sante, Microbiology, Virology	Laboratoire National de Sante, Microbiology, Epidemiology and Microbial Genomics	2020-04-21	Anke Wienecke-Baldacchino et al
Luxembourg/LNS9086704/2020	EPI_ISL_419601	Laboratoire National de Santé, Microbiology, Virology	Laboratoire National de Santé, Microbiology, Epidemiology and Microbial Genomics	2020-03-15	Anke Wienecke-Baldacchino et al
Luxembourg/LNS9102295/2020	EPI_ISL_429727	Laboratoire National de Sante, Microbiology, Virology	Laboratoire National de Sante, Microbiology, Epidemiology and Microbial Genomics	2020-03-19	Anke Wienecke-Baldacchino et al
Luxembourg/LNS9579356/2020	EPI_ISL_429708	Laboratoire National de Sante, Microbiology, Virology	Laboratoire National de Sante, Microbiology, Epidemiology and Microbial Genomics	2020-03-30	Anke Wienecke-Baldacchino et al
Luxembourg/LNS9982497/2020	EPI_ISL_419607	Laboratoire National de Santé, Microbiology, Virology	Laboratoire National de Santé, Microbiology, Epidemiology and Microbial Genomics	2020-03-14	Anke Wienecke-Baldacchino et al
Luxembourg/LNS9984061/2020	EPI_ISL_429724	Laboratoire National de Sante, Microbiology, Virology	Laboratoire National de Sante, Microbiology, Epidemiology and Microbial Genomics	2020-03-19	Anke Wienecke-Baldacchino et al
Luxembourg/Lux1/2020	EPI_ISL_413593	Laboratoire National de Santé	Erasmus Medical Center	2020-02-29	David Nieuwenhuijse et al B
Malaysia/188407/2020	EPI_ISL_417918	Department of Medical Microbiology, University Malaya Medical Centre	Department of Medical Microbiology, Faculty of Medicine, University of Malaya	2020-03-18	Yoong Min CHONG et al (https://dx.doi.org/10.1128/MRA.00383-20)
Malaysia/189332/2020	EPI_ISL_417917	Department of Medical Microbiology, University Malaya Medical Centre	Department of Medical Microbiology	2020-03-20	Yoong Min CHONG et al (https://dx.doi.org/10.1128/MRA.00383-20)
Malaysia/IIUM316/2020	EPI_ISL_455312	Microbiology Unit, Department of Pathology & Laboratory Medicine, IIUM Medical Centre	SEA Microbiome Unit, Faculty of Industrial Sciences & Technology, Universiti Malaysia Pahang	2020-04-09	Norhidayah Binti Kamarudin et al
Malaysia/IIUM91/2020	EPI_ISL_455313	Microbiology Unit, Department of Pathology & Laboratory Medicine, IIUM Medical Centre	SEA Microbiome Unit, Faculty of Industrial Sciences & Technology, Universiti Malaysia Pahang	2020-04-02	Hajar Fauzan Ahmad et al
Malaysia/IMR_WC085/2020	EPI_ISL_430443	Institute for Medical Research, Infectious Disease Research Centre, National Institutes of Health, Ministry of Health Malaysia	Institute for Medical Research, Infectious Disease Research Centre, National Institutes of Health, Ministry of Health Malaysia	2020-01-28	Suppiah.J et al
Malaysia/IMR_WC10180/2020	EPI_ISL_459957	Institute for Medical Research, Infectious Disease Research Centre, National Institutes of Health, Minis	Institute for Medical Research, Infectious Disease Research Centre, National Institutes of Health, Minis	2020-04-01	Suppiah J et al
Malaysia/IMR_WC1097/2020	EPI_ISL_430441	Institute for Medical Research, Infectious Disease Research Centre, National Institutes of Health, Ministry of Health Malaysia	Institute for Medical Research, Infectious Disease Research Centre, National Institutes of Health, Ministry of Health Malaysia	2020-02-29	Suppiah.J et al
Malaysia/IMR_WC1098/2020	EPI_ISL_430442	Institute for Medical Research, Infectious Disease Research Centre, National Institutes of Health, Ministry of Health Malaysia	Institute for Medical Research, Infectious Disease Research Centre, National Institutes of Health, Ministry of Health Malaysia	2020-02-29	Suppiah.J et al
Malaysia/IMR_WC1114/2020	EPI_ISL_455793	Institute for Medical Research, Infectious Disease Research Centre, National Institutes of Health, Ministry of Health Malaysia	Malaysia Genome Institute	2020-02-29	Mohd Noor Mat Isa et al
Malaysia/IMR_WC1170/2020	EPI_ISL_430440	Institute for Medical Research, Infectious Disease Research Centre, National Institutes of Health, Ministry of Health Malaysia	Institute for Medical Research, Infectious Disease Research Centre, National Institutes of Health, Ministry of Health Malaysia	2020-03-05	Suppiah.J et al
Malaysia/IMR_WC1177/2020	EPI_ISL_430439	Institute for Medical Research, Infectious Disease Research Centre, National Institutes of Health, Ministry of Health Malaysia	Institute for Medical Research Infectious Disease Research Centre, National Institutes of Health, Ministry of Health Malaysia	2020-03-05	Suppiah.J et al
Malaysia/IMR_WC2423/2020	EPI_ISL_459953	Institute for Medical Research, Infectious Disease Research Centre, National Institutes of Health, Ministry of Health Malaysia	Institute for Medical Research Infectious Disease Research Centre, National Institutes of Health, Ministry of Health Malaysia	2020-03-16	Suppiah J et al
Malaysia/IMR_WC2453/2020	EPI_ISL_459954	Institute for Medical Research, Infectious Disease Research Centre, National Institutes of Health, Ministry of Health Malaysia	Institute for Medical Research, Infectious Disease Research Centre, National Institutes of Health, Ministry of Health Malaysia	2020-03-16	Suppiah J et al
Malaysia/IMR_WC2665/2020	EPI_ISL_459955	Institute for Medical Research, Infectious Disease Research Centre, National Institutes of Health, Ministry of Health Malaysia	Institute for Medical Research, Infectious Disease Research Centre, National Institutes of Health, Ministry of Health Malaysia	2020-03-18	Suppiah J et al
Malaysia/IMR_WC413/2020	EPI_ISL_455791	Institute for Medical Research, Infectious Disease Research Centre, National Institutes of Health, Ministry of Health Malaysia	Malaysia Genome Institute	2020-02-08	Mohd Noor Mat Isa et al
Malaysia/IMR_WC458/2020	EPI_ISL_455792	Institute for Medical Research, Infectious Disease Research Centre, National Institutes of Health, Ministry of Health Malaysia	Malaysia Genome Institute	2020-02-09	Mohd Noor Mat Isa et al
Malaysia/IMR_WC627/2020	EPI_ISL_430444	Institute for Medical Research, Infectious Disease Research Centre, National Institutes of Health, Ministry of Health Malaysia	Institute for Medical Research, Infectious Disease Research Centre, National Institutes of Health, Ministry of Health Malaysia	2020-02-12	Suppiah.J et al
Malaysia/IMR_WC9127/2020	EPI_ISL_459956	Institute for Medical Research, Infectious Disease Research Centre, National Institutes of Health, Ministry of Health Malaysia	Institute for Medical Research, Infectious Disease Research Centre, National Institutes of Health, Ministry of Health Malaysia	2020-03-30	Suppiah J et al
Malaysia/MKAK-CL-2020-5045/2020	EPI_ISL_416829	National Public Health Laboratory	Malaysia Genome Institute	2020-01-24	Mohd Noor Mat Isa et al
Malaysia/MKAK-CL-2020-5047/2020	EPI_ISL_416866	National Public Health Laboratory	Malaysia Genome Institute	2020-01-24	Mohd Noor Mat Isa et al
Malaysia/MKAK-CL-2020-5049/2020	EPI_ISL_416884	National Public Health Laboratory	Malaysia Genome Institute	2020-01-24	Mohd Noor Mat Isa et al
Malaysia/MKAK-CL-2020-6430/2020	EPI_ISL_416886	National Public Health Laboratory	Malaysia Genome Institute	2020-02-04	Mohd Noor Mat Isa et al
Malaysia/MKAK-CL-2020-7554/2020	EPI_ISL_416907	National Public Health Laboratory	Malaysia Genome Institute	2020-02-06	Mohd Noor Mat Isa et al
Mexico/CDMX-INCMNSZ_01/2020	EPI_ISL_426361	Instituto Nacional de Ciencias Medicas y Nutricion Salvador Zubiran	Instituto Nacional de Ciencias Medicas y Nutricion Salvador Zubiran	2020-03-12	Guillermo M. Ruiz-Palacios et al (https://dx.doi.org/10.1101/2020.05.27.120402)
Mexico/CDMX-INCMNSZ_02/2020	EPI_ISL_426362	Instituto Nacional de Ciencias Medicas y Nutricion Salvador Zubiran	Instituto Nacional de Ciencias Medicas y Nutricion Salvador Zubiran	2020-03-10	Guillermo M. Ruiz-Palacios et al (https://dx.doi.org/10.1101/2020.05.27.120402)
Mexico/CDMX-INCMNSZ_03/2020	EPI_ISL_426363	Instituto Nacional de Ciencias Medicas y Nutricion Salvador Zubiran	Instituto Nacional de Ciencias Medicas y Nutricion Salvador Zubiran	2020-03-12	Guillermo M. Ruiz-Palacios et al (https://dx.doi.org/10.1101/2020.05.27.120402)
Mexico/CDMX-INCMNSZ_04/2020	EPI_ISL_426364	Instituto Nacional de Ciencias Medicas y Nutricion Salvador Zubiran	Instituto Nacional de Ciencias Medicas y Nutricion	2020-03-12	Guillermo M. Ruiz-Palacios et al (https://dx.doi.org/10.1101/2020.05.27.120402)
Mexico/CDMX-INCMNSZ_05/2020	EPI_ISL_426365	Instituto Nacional de Ciencias Medicas y Nutricion Salvador Zubiran	Instituto Nacional de Ciencias Medicas y Nutricion Salvador Zubiran	2020-03-12	Guillermo M. Ruiz-Palacios et al (https://dx.doi.org/10.1101/2020.05.27.120402)
Mexico/CDMX-InDRE_01/2020	EPI_ISL_412972	Instituto Nacional de Enfermedades Respiratorias	Instituto de Diagnostico y Referencia Epidemiologicos (INDRE)	2020-02-27	Ramirez-Gonzalez Ernesto et al B (https://dx.doi.org/10.1101/2020.05.27.120402)
Mexico/CDMX-InDRE_06/2020	EPI_ISL_424673	Instituto de Diagnostico y Referencia Epidemiologicos	Instituto de Diagnostico y Referencia Epidemiologicos	2020-03-12	Adnan Araiza Rodríguez et al (https://dx.doi.org/10.1101/2020.05.27.120402)
Mexico/CDMX-InDRE_07/2020	EPI_ISL_452139	Instituto de Diagnostico y Referencia Epidemiologicos (INDRE)	Instituto de diagnóstico y Referencia Epidemiologicos (INDRE)	2020-02-28	Ramirez-Gonzalez Ernesto et al A
Mexico/CDMX-InDRE_08/2020	EPI_ISL_452141	Instituto de Diagnostico y Referencia Epidemiologicos (INDRE)	Instituto de diagnóstico y Referencia Epidemiologicos (INDRE)	2020-03-11	Ramirez-Gonzalez Ernesto et al A
Mexico/CDMX-InDRE_09/2020	EPI_ISL_455432	Instituto de Diagnostico y Referencia Epidemiologicos (INDRE)	Instituto de Diagnostico y Referencia Epidemiologicos (INDRE)	2020-03-11	Taboada Ramírez Blanca. Ramirez-Gonzalez Ernesto et al
Mexico/CDMX-InDRE_10/2020	EPI_ISL_455434	Instituto de Diagnostico y Referencia Epidemiologicos (INDRE)	Instituto de Diagnostico y Referencia Epidemiologicos (INDRE)	2020-03-13	Taboada Ramírez Blanca. Ramirez-Gonzalez Ernesto et al
Mexico/CDMX-InDRE_11/2020	EPI_ISL_455435	Instituto de Diagnostico y Referencia Epidemiologicos (INDRE)	Instituto de Diagnostico y Referencia Epidemiologicos (INDRE)	2020-03-20	Garces-Ayala Fabiola. Taboada Ramírez Blanca. Ramirez-Gonzalez Ernesto et al
Mexico/CDMX-InDRE_12/2020	EPI_ISL_455436	Instituto de Diagnostico y Referencia Epidemiologicos (INDRE)	Instituto de Diagnostico y Referencia Epidemiologicos (INDRE)	2020-03-12	Garces-Ayala Fabiola. Taboada Ramírez Blanca. Ramirez-Gonzalez Ernesto et al
Mexico/CDMX-InDRE_13/2020	EPI_ISL_455437	Instituto de Diagnostico y Referencia Epidemiologicos (INDRE)	Instituto de Diagnostico y Referencia Epidemiologicos (INDRE)	2020-03-21	Araiza-Rodriguez Adnan et al
Mexico/CDMX-InDRE_14/2020	EPI_ISL_455438	Instituto de Diagnostico y Referencia Epidemiologicos (INDRE)	Instituto de Diagnostico y Referencia Epidemiologicos (INDRE)	2020-03-14	Araiza-Rodriguez Adnan et al
Mexico/CDMX-InDRE_15/2020	EPI_ISL_455439	Instituto de Diagnostico y Referencia Epidemiologicos (INDRE)	Instituto de Diagnostico y Referencia Epidemiologicos (INDRE)	2020-03-11	Mendieta-Condado Edgar et al
Mexico/CDMX-InDRE_16/2020	EPI_ISL_455454	Instituto de Diagnostico y Referencia Epidemiologicos (INDRE)	Instituto de Diagnostico y Referencia Epidemiologicos (INDRE)	2020-03-18	Mendieta-Condado Edgar et al
Mexico/CDMX-InDRE_17/2020	EPI_ISL_455455	Instituto de Diagnostico y Referencia Epidemiologicos (INDRE)	Instituto de Diagnostico y Referencia Epidemiologicos (INDRE)	2020-03-14	Rodriguez-Maldonado Abril et al
Mexico/CDMX-InDRE_18/2020	EPI_ISL_455456	Instituto de Diagnostico y Referencia Epidemiologicos (INDRE)	Instituto de Diagnostico y Referencia Epidemiologicos (INDRE)	2020-03-12	Rodriguez-Maldonado Abril et al
Mexico/CDMX-INER_01/2020	EPI_ISL_424345	Instituto Nacional de Enfermedades Respiratorias	Instituto Nacional de Enfermedades Respiratorias	2020-03-12	Joel Armando Vázquez Pérez et al (https://dx.doi.org/10.1101/2020.05.27.120402)
Mexico/CDMX-INER_02/2020	EPI_ISL_424348	Instituto Nacional de Enfermedades Respiratorias	Instituto Nacional de Enfermedades Respiratorias	2020-03-13	Joel Armando Vázquez Pérez et al (https://dx.doi.org/10.1101/2020.05.27.120402)
Mexico/CDMX-INER_04/2020	EPI_ISL_424626	Instituto Nacional de Enfermedades Respiratorias	Instituto Nacional de Enfermedades Respiratorias	2020-03-15	Joel Armando Vázquez Pérez et al (https://dx.doi.org/10.1101/2020.05.27.120402)
Mexico/CDMX-INER_05/2020	EPI_ISL_424627	Instituto Nacional de Enfermedades Respiratorias	Instituto Nacional de Enfermedades Respiratorias	2020-03-16	Joel Armando Vázquez Pérez et al (https://dx.doi.org/10.1101/2020.05.27.120402)
Mexico/Chihuahua-IMSS_01/2020	EPI_ISL_424731	Hospital General Regional No.66, Ciudad Juárez, Chihuahua.	Laboratorio Central de Epidemiología-DLVIE / Laboratorio de Secuenciación-Centro de Instrumentos. Instituto Mexicano del Seguro Social	2020-03-13	Muñoz-Medina JE et al (https://dx.doi.org/10.1101/2020.05.27.120402)
Mexico/EdoMex-InDRE_03/2020	EPI_ISL_424667	Laboratorio Estatal de Salud Publica del Estado de México	Instituto de Diagnóstico y Referencia Epidemiológicos	2020-03-04	Irma López Martínez et al (https://dx.doi.org/10.1101/2020.05.27.120402)
Mexico/Puebla-InDRE_05/2020	EPI_ISL_424672	Laboratorio Estatal de Salud Publica del Estado de Puebla	Instituto de Diagnostico y Referencia Epidemiologicos	2020-03-11	Fabiola Garcés Ayala et al (https://dx.doi.org/10.1101/2020.05.27.120402)
Mexico/Queretaro-InDRE_04/2020	EPI_ISL_424670	Laboratorio Estatal de Salud Publica del Estado de Queretaro	Instituto de Diagnóstico y Referencia Epidemiologicos	2020-03-10	Gisela Barrera Badillo et al (https://dx.doi.org/10.1101/2020.05.27.120402)
mink/Netherlands/NB04_index/2020	EPI_ISL_447634		Virology	2020-05-06	Oreshkova et al

Morocco/15N/2020	EPI_ISL_458150	ANOUAL	ANOUAL	2020-05-15	Jouali Farah et al
Morocco/6887/2020	EPI_ISL_459965	Institut Pasteur du Maroc	Institut Pasteur du Maroc	2020-03-03	Marion Barbet et al
Morocco/6888/2020	EPI_ISL_459966	Institut Pasteur du Maroc	Institut Pasteur du Maroc	2020-03-15	Marion Barbet et al
Morocco/6889/2020	EPI_ISL_459967	Institut Pasteur du Maroc	Institut Pasteur du Maroc	2020-03-15	Marion Barbet et al
Morocco/6890/2020	EPI_ISL_459968	Institut Pasteur du Maroc	Institut Pasteur du Maroc	2020-03-17	Marion Barbet et al
Morocco/6891/2020	EPI_ISL_459969	Institut Pasteur du Maroc	Institut Pasteur du Maroc	2020-03-20	Marion Barbet et al
Morocco/6892/2020	EPI_ISL_459970	Institut Pasteur du Maroc	Institut Pasteur du Maroc	2020-03-17	Marion Barbet et al
Morocco/6893/2020	EPI_ISL_459971	Institut Pasteur du Maroc	Institut Pasteur du Maroc	2020-03-18	Marion Barbet et al
Morocco/6894/2020	EPI_ISL_459972	Institut Pasteur du Maroc	Institut Pasteur du Maroc	2020-03-20	Marion Barbet et al
Morocco/6895/2020	EPI_ISL_459973	Institut Pasteur du Maroc	Institut Pasteur du Maroc	2020-03-20	Marion Barbet et al
Morocco/6896/2020	EPI_ISL_459974	Institut Pasteur du Maroc	Institut Pasteur du Maroc	2020-03-20	Marion Barbet et al
Morocco/6897/2020	EPI_ISL_459975	Institut Pasteur du Maroc	Institut Pasteur du Maroc	2020-03-21	Marion Barbet et al
Morocco/6898/2020	EPI_ISL_459976	Institut Pasteur du Maroc	Institut Pasteur du Maroc	2020-03-16	Marion Barbet et al
Morocco/6899/2020	EPI_ISL_459977	Institut Pasteur du Maroc	Institut Pasteur du Maroc	2020-04-21	Marion Barbet et al
Morocco/6900/2020	EPI_ISL_459978	Institut Pasteur du Maroc	Institut Pasteur du Maroc	2020-04-20	Marion Barbet et al
Morocco/6901/2020	EPI_ISL_459979	Institut Pasteur du Maroc	Institut Pasteur du Maroc	2020-04-19	Marion Barbet et al
Morocco/6902/2020	EPI_ISL_459980	Institut Pasteur du Maroc	Institut Pasteur du Maroc	2020-04-19	Marion Barbet et al
Morocco/6903/2020	EPI_ISL_459981	Institut Pasteur du Maroc	Institut Pasteur du Maroc	2020-04-19	Marion Barbet et al
Morocco/6904/2020	EPI_ISL_459982	Institut Pasteur du Maroc	Institut Pasteur du Maroc	2020-04-18	Marion Barbet et al
Morocco/6905/2020	EPI_ISL_459983	Institut Pasteur du Maroc	Institut Pasteur du Maroc	2020-04-21	Marion Barbet et al
Morocco/6906/2020	EPI_ISL_459984	Institut Pasteur du Maroc	Institut Pasteur du Maroc	2020-04-06	Marion Barbet et al
Morocco/OUA677-19/2020	EPI_ISL_451400	Laboratoire de Recherche et d'Analyse Médicale de la Gendarmerie Royale	Laboratoire de Recherche et d'Analyse Médicale de la Gendarmerie Royale	2020-04-23	Sanaâ LEMRISS et al
Morocco/RMPS-01/2020	EPI_ISL_469017	LNR National Reference Laboratory, Mohammed VI University of Health Sciences	Medical Biotechnology Laboratory, Rabat Medical and Pharmacy School, Mohammed The Vth University in Rabat	2020-04-13	Meriem LAAMARTI et al
Morocco/RMPS-02/2020	EPI_ISL_469049	LNR National Reference Laboratory, Mohammed VI University of Health Sciences	Medical Biotechnology Laboratory, Rabat Medical and Pharmacy School, Mohammed The Vth University in Rabat	2020-03-30	Meriem LAAMARTI et al
Morocco/RMPS-03/2020	EPI_ISL_469051	LNR National Reference Laboratory, Mohammed VI University of Health Sciences	Medical Biotechnology Laboratory, Rabat Medical and Pharmacy School, Mohammed The Vth University in Rabat	2020-04-03	Meriem LAAMARTI et al
Morocco/RMPS-04/2020	EPI_ISL_469052	LNR National Reference Laboratory, Mohammed VI University of Health Sciences	Medical Biotechnology Laboratory, Rabat Medical and Pharmacy School, Mohammed The Vth University in Rabat	2020-03-30	Meriem LAAMARTI et al
Morocco/RMPS-05/2020	EPI_ISL_469053	LNR National Reference Laboratory, Mohammed VI University of Health Sciences	Medical Biotechnology Laboratory, Rabat Medical and Pharmacy School, Mohammed The Vth University in Rabat	2020-03-30	Meriem LAAMARTI et al
Morocco/RMPS-06/2020	EPI_ISL_469054	LNR National Reference Laboratory, Mohammed VI University of Health Sciences	Medical Biotechnology Laboratory, Rabat Medical and Pharmacy School, Mohammed The Vth University in Rabat	2020-04-01	Meriem LAAMARTI et al
Nanchang/JX155/2020	EPI_ISL_421262	Jiangxi Province Center for Disease Control and Prevention	Jiangxi Province Center for Disease Control and Prevention	2020-01-29	JianXiong Li et al
Nepal/61/2020	EPI_ISL_410301	National Influenza Centre, National Public Health Laboratory, Kathmandu, Nepal	The University of Hong Kong	2020-01-13	Ranjit Sah et al (http://biorxiv.org/lookup/doi/10.1101/2020.04.01.019463)
Netherlands/Berlicum_1363564/2020	EPI_ISL_413565	Foundation Pamm	Erasmus Medical Center	2020-02-24	David Nieuwenhuijse et al A (https://dx.doi.org/10.1101/2020.04.21.050633)
Netherlands/Delft_1363424/2020	EPI_ISL_413569	RIVM	Erasmus Medical Center	2020-02-28	David Nieuwenhuijse et al A (https://dx.doi.org/10.1101/2020.04.21.050633)
Netherlands/Diemen_1363454/2020	EPI_ISL_413570	RIVM	Erasmus Medical Center	2020-02-28	David Nieuwenhuijse et al A (https://dx.doi.org/10.1101/2020.04.21.050633)
Netherlands/Flevoland_1/2020	EPI_ISL_415460	Dutch COVID-19 response team	Erasmus Medical Center	2020-03-09	David Nieuwenhuijse et al A (https://dx.doi.org/10.1101/2020.04.21.050633)
Netherlands/Flevoland_16/2020	EPI_ISL_460839	Dutch COVID-19 response team	Erasmus Medical Center	2020-05-20	Bas Oude Munnink et al
Netherlands/Flevoland_6/2020	EPI_ISL_460639	Dutch COVID-19 response team	Erasmus Medical Center	2020-04-15	Bas Oude Munnink et al
Netherlands/Flevoland_7/2020	EPI_ISL_460640	Dutch COVID-19 response team	Erasmus Medical Center	2020-04-15	Bas Oude Munnink et al
Netherlands/Friesland_15/2020	EPI_ISL_455118	Dutch COVID-19 response team	Erasmus Medical Center	2020-03-31	Bas Oude Munnink et al
Netherlands/Friesland_26/2020	EPI_ISL_460648	Dutch COVID-19 response team	Erasmus Medical Center	2020-04-08	Bas Oude Munnink et al
Netherlands/Friesland_40/2020	EPI_ISL_460855	Dutch COVID-19 response team	Erasmus Medical Center	2020-05-01	Bas Oude Munnink et al
Netherlands/Friesland_6/2020	EPI_ISL_422571	Dutch COVID-19 response team	Erasmus Medical Center	2020-03-28	Bas Oude Munnink et al
Netherlands/Gelderland_101/2020	EPI_ISL_460862	Dutch COVID-19 response team	Erasmus Medical Center	2020-05-21	Bas Oude Munnink et al
Netherlands/Gelderland_102/2020	EPI_ISL_460863	Dutch COVID-19 response team	Erasmus Medical Center	2020-05-21	Bas Oude Munnink et al
Netherlands/Gelderland_104/2020	EPI_ISL_460865	Dutch COVID-19 response team	Erasmus Medical Center	2020-05-21	Bas Oude Munnink et al
Netherlands/Gelderland_110/2020	EPI_ISL_460871	Dutch COVID-19 response team	Erasmus Medical Center	2020-05-22	Bas Oude Munnink et al
Netherlands/Gelderland_114/2020	EPI_ISL_460875	Dutch COVID-19 response team	Erasmus Medical Center	2020-05-22	Bas Oude Munnink et al
Netherlands/Gelderland_122/2020	EPI_ISL_460883	Dutch COVID-19 response team	Erasmus Medical Center	2020-05-22	Bas Oude Munnink et al
Netherlands/Gelderland_123/2020	EPI_ISL_460884	Dutch COVID-19 response team	Erasmus Medical Center	2020-05-22	Bas Oude Munnink et al
Netherlands/Gelderland_129/2020	EPI_ISL_460890	Dutch COVID-19 response team	Erasmus Medical Center	2020-05-22	Bas Oude Munnink et al
Netherlands/Gelderland_151/2020	EPI_ISL_460911	Dutch COVID-19 response team	Erasmus Medical Center	2020-05-22	Bas Oude Munnink et al
Netherlands/Gelderland_154/2020	EPI_ISL_460914	Dutch COVID-19 response team	Erasmus Medical Center	2020-05-22	Bas Oude Munnink et al
Netherlands/Gelderland_42/2020	EPI_ISL_460942	Dutch COVID-19 response team	Erasmus Medical Center	2020-04-28	Bas Oude Munnink et al
Netherlands/Gelderland_57/2020	EPI_ISL_460956	Dutch COVID-19 response team	Erasmus Medical Center	2020-05-18	Bas Oude Munnink et al
Netherlands/Gelderland_76/2020	EPI_ISL_460975	Dutch COVID-19 response team	Erasmus Medical Center	2020-05-19	Bas Oude Munnink et al
Netherlands/Gelderland_79/2020	EPI_ISL_460978	Dutch COVID-19 response team	Erasmus Medical Center	2020-05-19	Bas Oude Munnink et al
Netherlands/Gelderland_84/2020	EPI_ISL_460983	Dutch COVID-19 response team	Erasmus Medical Center	2020-05-19	Bas Oude Munnink et al
Netherlands/Gelderland_92/2020	EPI_ISL_460991	Dutch COVID-19 response team	Erasmus Medical Center	2020-05-23	Bas Oude Munnink et al
Netherlands/Helmond_1363548/2020	EPI_ISL_413574	MHC West-Brabant	Erasmus Medical Center	2020-02-29	David Nieuwenhuijse et al A (https://dx.doi.org/10.1101/2020.04.21.050633)
Netherlands/Houten_1363498/2020	EPI_ISL_413575	RIVM	Erasmus Medical Center	2020-02-29	David Nieuwenhuijse et al A (https://dx.doi.org/10.1101/2020.04.21.050633)
Netherlands/Limburg_19/2020	EPI_ISL_461007	Dutch COVID-19 response team	Erasmus Medical Center	2020-05-14	Bas Oude Munnink et al
Netherlands/Limburg_39/2020	EPI_ISL_461027	Dutch COVID-19 response team	Erasmus Medical Center	2020-04-19	Bas Oude Munnink et al
Netherlands/Limburg_59/2020	EPI_ISL_461045	Dutch COVID-19 response team	Erasmus Medical Center	2020-04-24	Bas Oude Munnink et al
Netherlands/Limburg_62/2020	EPI_ISL_461048	Dutch COVID-19 response team	Erasmus Medical Center	2020-04-19	Bas Oude Munnink et al
Netherlands/Loon_op_zand_1363512/2020	EPI_ISL_413576	RIVM	Erasmus Medical Center	2020-02-29	David Nieuwenhuijse et al A (https://dx.doi.org/10.1101/2020.04.21.050633)
Netherlands/NA_106/2020	EPI_ISL_422655	Dutch COVID-19 response team	Erasmus Medical Center	2020-03-18	Bas Oude Munnink et al
Netherlands/NA_168/2020	EPI_ISL_422703	Dutch COVID-19 response team	Erasmus Medical Center	2020-03-16	Bas Oude Munnink et al
Netherlands/NA_22/2020	EPI_ISL_415479	Dutch COVID-19 response team	Erasmus Medical Center	2020-03-08	David Nieuwenhuijse et al A (https://dx.doi.org/10.1101/2020.04.21.050633)
Netherlands/NA_286/2020	EPI_ISL_422595	Dutch COVID-19 response team	Erasmus Medical Center	2020-03-31	Bas Oude Munnink et al
Netherlands/NA_300/2020	EPI_ISL_422609	Dutch COVID-19 response team	Erasmus Medical Center	2020-04-01	Bas Oude Munnink et al
Netherlands/NA_354/2020	EPI_ISL_455158	Dutch COVID-19 response team	Erasmus Medical Center	2020-04-07	Bas Oude Munnink et al
Netherlands/NA_357/2020	EPI_ISL_455161	Dutch COVID-19 response team	Erasmus Medical Center	2020-04-07	Bas Oude Munnink et al
Netherlands/NA_367/2020	EPI_ISL_455171	Dutch COVID-19 response team	Erasmus Medical Center	2020-04-08	Bas Oude Munnink et al
Netherlands/NA_39/2020	EPI_ISL_422800	Dutch COVID-19 response team	Erasmus Medical Center	2020-03-14	Bas Oude Munnink et al
Netherlands/NA_397/2020	EPI_ISL_455199	Dutch COVID-19 response team	Erasmus Medical Center	2020-04-03	Bas Oude Munnink et al
Netherlands/NA_402/2020	EPI_ISL_455204	Dutch COVID-19 response team	Erasmus Medical Center	2020-04-01	Bas Oude Munnink et al
Netherlands/NA_434/2020	EPI_ISL_455236	Dutch COVID-19 response team	Erasmus Medical Center	2020-04-07	Bas Oude Munnink et al
Netherlands/NA_436/2020	EPI_ISL_455238	Dutch COVID-19 response team	Erasmus Medical Center	2020-04-07	Bas Oude Munnink et al
Netherlands/NA_461/2020	EPI_ISL_455263	Dutch COVID-19 response team	Erasmus Medical Center	2020-04-08	Bas Oude Munnink et al
Netherlands/NA_479/2020	EPI_ISL_455281	Dutch COVID-19 response team	Erasmus Medical Center	2020-04-09	Bas Oude Munnink et al
Netherlands/NA_493/2020	EPI_ISL_460666	Dutch COVID-19 response team	Erasmus Medical Center	2020-03-17	Bas Oude Munnink et al
Netherlands/NA_528/2020	EPI_ISL_460701	Dutch COVID-19 response team	Erasmus Medical Center	2020-03-25	Bas Oude Munnink et al
Netherlands/NA_571/2020	EPI_ISL_460735	Dutch COVID-19 response team	Erasmus Medical Center	2020-04-13	Bas Oude Munnink et al
Netherlands/NA_613/2020	EPI_ISL_460776	Dutch COVID-19 response team	Erasmus Medical Center	2020-04-21	Bas Oude Munnink et al
Netherlands/NA_62/2020	EPI_ISL_422823	Dutch COVID-19 response team	Erasmus Medical Center	2020-03-09	Bas Oude Munnink et al
Netherlands/NA_650/2020	EPI_ISL_461085	Dutch COVID-19 response team	Erasmus Medical Center	2020-05-07	Bas Oude Munnink et al
Netherlands/NA_652/2020	EPI_ISL_461087	Dutch COVID-19 response team	Erasmus Medical Center	2020-05-08	Bas Oude Munnink et al
Netherlands/NA_666/2020	EPI_ISL_461101	Dutch COVID-19 response team	Erasmus Medical Center	2020-02-29	Bas Oude Munnink et al
Netherlands/NA_667/2020	EPI_ISL_461102	Dutch COVID-19 response team	Erasmus Medical Center	2020-03-08	Bas Oude Munnink et al
Netherlands/NA_682/2020	EPI_ISL_461117	Dutch COVID-19 response team	Erasmus Medical Center	2020-03-24	Bas Oude Munnink et al
Netherlands/NA_702/2020	EPI_ISL_461137	Dutch COVID-19 response team	Erasmus Medical Center	2020-04-19	Bas Oude Munnink et al
Netherlands/NA_710/2020	EPI_ISL_461145	Dutch COVID-19 response team	Erasmus Medical Center	2020-05-13	Bas Oude Munnink et al
Netherlands/NA_84/2020	EPI_ISL_422844	Dutch COVID-19 response team	Erasmus Medical Center	2020-03-12	Bas Oude Munnink et al
Netherlands/NoordBrabant_10003/2020	EPI_ISL_454752	Dutch COVID-19 response team	National Institute for Public Health and the Environment (RIVM)	2020-02-28	Adam Meijer et al
Netherlands/NoordBrabant_10039/2020	EPI_ISL_454788	Dutch COVID-19 response team	National Institute for Public Health and the Environment (RIVM)	2020-03-16	Adam Meijer et al
Netherlands/NoordBrabant_106/2020	EPI_ISL_455290	Dutch COVID-19 response team	Erasmus Medical Center	2020-04-06	Bas Oude Munnink et al
Netherlands/NoordBrabant_60/2020	EPI_ISL_415516	Dutch COVID-19 response team	Erasmus Medical Center	2020-03-11	David Nieuwenhuijse et al A (https://dx.doi.org/10.1101/2020.04.21.050633)
Netherlands/NoordBrabant_97/2020	EPI_ISL_422891	Dutch COVID-19 response team	Erasmus Medical Center	2020-03-19	Bas Oude Munnink et al
Netherlands/NoordHolland_10001/2020	EPI_ISL_454750	Dutch COVID-19 response team	National Institute for Public Health and the Environment (RIVM)	2020-02-27	Adam Meijer et al
Netherlands/NoordHolland_10002/2020	EPI_ISL_454751	Dutch COVID-19 response team	National Institute for Public Health and the Environment (RIVM)	2020-02-28	Adam Meijer et al
Netherlands/NoordHolland_10011/2020	EPI_ISL_454760	Dutch COVID-19 response team	National Institute for Public Health and the Environment (RIVM)	2020-03-14	Adam Meijer et al
Netherlands/Oss_1363500/2020	EPI_ISL_413581	RIVM	Erasmus Medical Center	2020-02-29	David Nieuwenhuijse et al A (https://dx.doi.org/10.1101/2020.04.21.050633)
Netherlands/Overijssel_10/2020	EPI_ISL_460799	Dutch COVID-19 response team	Erasmus Medical Center	2020-04-08	Bas Oude Munnink et al
Netherlands/Overijssel_35/2020	EPI_ISL_461227	Dutch COVID-19 response team	Erasmus Medical Center	2020-05-26	Bas Oude Munnink et al
Netherlands/Overijssel_6/2020	EPI_ISL_460825	Dutch COVID-19 response team	Erasmus Medical Center	2020-04-06	Bas Oude Munnink et al
Netherlands/Tilburg_1363354/2020	EPI_ISL_413586	Foundation Elisabeth-Tweesteden Ziekenhuis	Erasmus Medical Center	2020-02-27	David Nieuwenhuijse et al A (https://dx.doi.org/10.1101/2020.04.21.050633)
Netherlands/Utrecht_27/2020	EPI_ISL_460830	Dutch COVID-19 response team	Erasmus Medical Center	2020-04-02	Bas Oude Munnink et al
Netherlands/Utrecht_5/2020	EPI_ISL_414439	Dutch COVID-19 response team	Erasmus Medical Center	2020-03-02	David Nieuwenhuijse et al A (https://dx.doi.org/10.1101/2020.04.21.050633)
Netherlands/Utrecht_7/2020	EPI_ISL_414440	Dutch COVID-19 response team	Erasmus Medical Center	2020-03-03	David Nieuwenhuijse et al A (https://dx.doi.org/10.1101/2020.04.21.050633)
Netherlands/Zeeland_16/2020	EPI_ISL_461254	Dutch COVID-19 response team	Erasmus Medical Center	2020-03-28	Bas Oude Munnink et al
Netherlands/Zeeland_5/2020	EPI_ISL_461287	Dutch COVID-19 response team	Erasmus Medical Center	2020-03-18	Bas Oude Munnink et al
Netherlands/ZuidHolland_10004/2020	EPI_ISL_454753	Dutch COVID-19 response team	National Institute for Public Health and the Environment (RIVM)	2020-02-28	Adam Meijer et al
Netherlands/ZuidHolland_10005/2020	EPI_ISL_454754	Dutch COVID-19 response team	National Institute for Public Health and the Environment (RIVM)	2020-02-28	Adam Meijer et al
Netherlands/ZuidHolland_104/2020	EPI_ISL_422903	Dutch COVID-19 response team	Erasmus Medical Center	2020-03-29	Bas Oude Munnink et al

Netherlands/ZuidHolland_106/2020	EPI_ISL_455295	Dutch COVID-19 response team	Erasmus Medical Center	2020-04-04	Bas Oude Munnink et al
Netherlands/ZuidHolland_130/2020	EPI_ISL_461303	Dutch COVID-19 response team	Erasmus Medical Center	2020-04-27	Bas Oude Munnink et al
Netherlands/ZuidHolland_135/2020	EPI_ISL_461308	Dutch COVID-19 response team	Erasmus Medical Center	2020-05-07	Bas Oude Munnink et al
Netherlands/ZuidHolland_137/2020	EPI_ISL_461310	Dutch COVID-19 response team	Erasmus Medical Center	2020-05-04	Bas Oude Munnink et al
Netherlands/ZuidHolland_146/2020	EPI_ISL_461319	Dutch COVID-19 response team	Erasmus Medical Center	2020-04-19	Bas Oude Munnink et al
Netherlands/ZuidHolland_15/2020	EPI_ISL_414557	Dutch COVID-19 response team	Erasmus Medical Center	2020-03-08	David Nieuwenhuijse et al A (https://dx.doi.org/10.1101/2020.04.21.050633)
Netherlands/ZuidHolland_166/2020	EPI_ISL_461338	Dutch COVID-19 response team	Erasmus Medical Center	2020-04-16	Bas Oude Munnink et al
Netherlands/ZuidHolland_18/2020	EPI_ISL_414560	Dutch COVID-19 response team	Erasmus Medical Center	2020-03-03	David Nieuwenhuijse et al A (https://dx.doi.org/10.1101/2020.04.21.050633)
Netherlands/ZuidHolland_180/2020	EPI_ISL_461352	Dutch COVID-19 response team	Erasmus Medical Center	2020-04-22	Bas Oude Munnink et al
Netherlands/ZuidHolland_182/2020	EPI_ISL_461354	Dutch COVID-19 response team	Erasmus Medical Center	2020-04-28	Bas Oude Munnink et al
Netherlands/ZuidHolland_19/2020	EPI_ISL_414561	Dutch COVID-19 response team	Erasmus Medical Center	2020-03-05	David Nieuwenhuijse et al A (https://dx.doi.org/10.1101/2020.04.21.050633)
Netherlands/ZuidHolland_196/2020	EPI_ISL_461368	Dutch COVID-19 response team	Erasmus Medical Center	2020-05-02	Bas Oude Munnink et al
Netherlands/ZuidHolland_203/2020	EPI_ISL_461375	Dutch COVID-19 response team	Erasmus Medical Center	2020-05-03	Bas Oude Munnink et al
Netherlands/ZuidHolland_207/2020	EPI_ISL_461379	Dutch COVID-19 response team	Erasmus Medical Center	2020-05-06	Bas Oude Munnink et al
Netherlands/ZuidHolland_21/2020	EPI_ISL_414563	Dutch COVID-19 response team	Erasmus Medical Center	2020-03-03	David Nieuwenhuijse et al A (https://dx.doi.org/10.1101/2020.04.21.050633)
Netherlands/ZuidHolland_211/2020	EPI_ISL_461383	Dutch COVID-19 response team	Erasmus Medical Center	2020-05-07	Bas Oude Munnink et al
Netherlands/ZuidHolland_214/2020	EPI_ISL_461386	Dutch COVID-19 response team	Erasmus Medical Center	2020-05-08	Bas Oude Munnink et al
Netherlands/ZuidHolland_86/2020	EPI_ISL_422954	Dutch COVID-19 response team	Erasmus Medical Center	2020-03-27	Bas Oude Munnink et al
NewZealand/01/2020	EPI_ISL_413490	Auckland Hospital	Institute of Environmental Science and Research (ESR)	2020-02-27	Matt Storey et al B (https://dx.doi.org/10.1101/2020.03.15.992818)
NewZealand/20VR0461/2020	EPI_ISL_456158	Southern Community Labs Dunedin	Institute of Environmental Science and Research (ESR)	2020-03-13	Matt Storey et al A
NewZealand/20VR0995/2020	EPI_ISL_456178	LabPLUS	Institute of Environmental Science and Research (ESR)	2020-03-17	Matt Storey et al A
NewZealand/20VR0999/2020	EPI_ISL_456180	LabPLUS	Institute of Environmental Science and Research (ESR)	2020-03-16	Matt Storey et al A
NewZealand/20VR1016/2020	EPI_ISL_456182	Wellington SCL	Institute of Environmental Science and Research (ESR)	2020-03-20	Matt Storey et al A
NewZealand/20VR1018/2020	EPI_ISL_456183	Wellington SCL	Institute of Environmental Science and Research (ESR)	2020-03-20	Matt Storey et al A
NewZealand/20VR1019/2020	EPI_ISL_456184	Wellington SCL	Institute of Environmental Science and Research (ESR)	2020-03-20	Matt Storey et al A
NewZealand/20VR1020/2020	EPI_ISL_456185	Wellington SCL	Institute of Environmental Science and Research (ESR)	2020-03-20	Matt Storey et al A
NewZealand/20VR1022/2020	EPI_ISL_456186	Wellington SCL	Institute of Environmental Science and Research (ESR)	2020-03-20	Matt Storey et al A
NewZealand/20VR1076/2020	EPI_ISL_456187	Wellington SCL	Institute of Environmental Science and Research (ESR)	2020-03-22	Matt Storey et al A
NewZealand/20VR1110/2020	EPI_ISL_456191	Waikato Hospital	Institute of Environmental Science and Research (ESR)	2020-03-19	Matt Storey et al A
NewZealand/20VR1114/2020	EPI_ISL_456192	Waikato Hospital	Institute of Environmental Science and Research (ESR)	2020-03-20	Matt Storey et al A
NewZealand/20VR1273/2020	EPI_ISL_456198	LabPLUS	Institute of Environmental Science and Research (ESR)	2020-03-21	Matt Storey et al A
NewZealand/20VR1282/2020	EPI_ISL_456207	LabPLUS	Institute of Environmental Science and Research (ESR)	2020-03-20	Matt Storey et al A
NewZealand/20VR1452/2020	EPI_ISL_456219	Southern Community Labs Dunedin	Institute of Environmental Science and Research (ESR)	2020-03-21	Matt Storey et al A
NewZealand/20VR1615/2020	EPI_ISL_456225	Southern Community Labs Dunedin	Institute of Environmental Science and Research (ESR)	2020-03-24	Matt Storey et al A
NewZealand/20VR1620/2020	EPI_ISL_456230	Southern Community Labs Dunedin	Institute of Environmental Science and Research (ESR)	2020-03-24	Matt Storey et al A
NewZealand/20VR1621/2020	EPI_ISL_456231	Southern Community Labs Dunedin	Institute of Environmental Science and Research (ESR)	2020-03-24	Matt Storey et al A
NewZealand/20VR1624/2020	EPI_ISL_456234	Southern Community Labs Dunedin	Institute of Environmental Science and Research (ESR)	2020-03-27	Matt Storey et al A
NewZealand/20VR1778/2020	EPI_ISL_456247	Southern Community Labs Dunedin	Institute of Environmental Science and Research (ESR)	2020-03-25	Matt Storey et al A
NewZealand/20VR1782/2020	EPI_ISL_456251	Southern Community Labs Dunedin	Institute of Environmental Science and Research (ESR)	2020-03-26	Matt Storey et al A
NewZealand/20VR1784/2020	EPI_ISL_456253	Southern Community Labs Dunedin	Institute of Environmental Science and Research (ESR)	2020-03-26	Matt Storey et al A
NewZealand/20VR1785/2020	EPI_ISL_456254	Southern Community Labs Dunedin	Institute of Environmental Science and Research (ESR)	2020-03-26	Matt Storey et al A
NewZealand/20VR1825/2020	EPI_ISL_456270	Southern Community Labs Dunedin	Institute of Environmental Science and Research (ESR)	2020-03-27	Matt Storey et al A
NewZealand/20VR1843/2020	EPI_ISL_456281	Southern Community Labs Dunedin	Institute of Environmental Science and Research (ESR)	2020-03-28	Matt Storey et al A
NewZealand/20VR1867/2020	EPI_ISL_456292	Southern Community Labs Dunedin	Institute of Environmental Science and Research (ESR)	2020-03-31	Matt Storey et al A
NewZealand/20VR1900/2020	EPI_ISL_456301	PathLab Bay of Plenty	Institute of Environmental Science and Research (ESR)	2020-04-04	Matt Storey et al A
NewZealand/20VR1911/2020	EPI_ISL_456306	Southern Community Labs Dunedin	Institute of Environmental Science and Research (ESR)	2020-04-02	Matt Storey et al A
NewZealand/20VR1924/2020	EPI_ISL_456309	Southern Community Labs Dunedin	Institute of Environmental Science and Research (ESR)	2020-04-03	Matt Storey et al A
NewZealand/20VR1933/2020	EPI_ISL_456314	Southern Community Labs Dunedin	Institute of Environmental Science and Research (ESR)	2020-04-03	Matt Storey et al A
NewZealand/20VR1937/2020	EPI_ISL_456316	Southern Community Labs Dunedin	Institute of Environmental Science and Research (ESR)	2020-04-01	Matt Storey et al A
NewZealand/20VR1941/2020	EPI_ISL_456317	Southern Community Labs Dunedin	Institute of Environmental Science and Research (ESR)	2020-04-04	Matt Storey et al A
NewZealand/20VR1947/2020	EPI_ISL_456318	Southern Community Labs Dunedin	Institute of Environmental Science and Research (ESR)	2020-04-03	Matt Storey et al A
NewZealand/20VR1995/2020	EPI_ISL_456325	Wellington SCL	Institute of Environmental Science and Research (ESR)	2020-04-01	Matt Storey et al A
NewZealand/20VR2006/2020	EPI_ISL_456331	Wellington SCL	Institute of Environmental Science and Research (ESR)	2020-04-01	Matt Storey et al A
NewZealand/20VR2011/2020	EPI_ISL_456332	Wellington SCL	Institute of Environmental Science and Research (ESR)	2020-04-01	Matt Storey et al A
NewZealand/20VR2045/2020	EPI_ISL_456344	Southern Community Labs Dunedin	Institute of Environmental Science and Research (ESR)	2020-04-10	Matt Storey et al A
NewZealand/20VR2054/2020	EPI_ISL_456349	Canterbury Health Laboratories	Institute of Environmental Science and Research (ESR)	2020-04-03	Matt Storey et al A
NewZealand/20VR2055/2020	EPI_ISL_456350	Canterbury Health Laboratories	Institute of Environmental Science and Research (ESR)	2020-04-03	Matt Storey et al A
NewZealand/20VR2065/2020	EPI_ISL_456356	Canterbury Health Laboratories	Institute of Environmental Science and Research (ESR)	2020-04-06	Matt Storey et al A
NewZealand/20VR2066/2020	EPI_ISL_456357	Canterbury Health Laboratories	Institute of Environmental Science and Research (ESR)	2020-04-06	Matt Storey et al A
NewZealand/20VR2073/2020	EPI_ISL_456360	Canterbury Health Laboratories	Institute of Environmental Science and Research (ESR)	2020-04-08	Matt Storey et al A
NewZealand/20VR2074/2020	EPI_ISL_456361	Canterbury Health Laboratories	Institute of Environmental Science and Research (ESR)	2020-04-08	Matt Storey et al A
NewZealand/20VR2075/2020	EPI_ISL_456362	Canterbury Health Laboratories	Institute of Environmental Science and Research (ESR)	2020-04-09	Matt Storey et al A
NewZealand/20VR2078/2020	EPI_ISL_456365	Canterbury Health Laboratories	Institute of Environmental Science and Research (ESR)	2020-04-09	Matt Storey et al A
NewZealand/20VR2085/2020	EPI_ISL_456369	Canterbury Health Laboratories	Institute of Environmental Science and Research (ESR)	2020-04-12	Matt Storey et al A
NewZealand/20VR2089/2020	EPI_ISL_456372	Canterbury Health Laboratories	Institute of Environmental Science and Research (ESR)	2020-04-13	Matt Storey et al A
NewZealand/20VR2475/2020	EPI_ISL_456379	LabPLUS	Institute of Environmental Science and Research (ESR)	2020-04-15	Matt Storey et al A
NewZealand/20VR2554/2020	EPI_ISL_456385	Middlemore Hospital	Institute of Environmental Science and Research (ESR)	2020-04-15	Matt Storey et al A
NewZealand/20VR2562/2020	EPI_ISL_456387	Middlemore Hospital	Institute of Environmental Science and Research (ESR)	2020-04-15	Matt Storey et al A
NewZealand/20VR2922/2020	EPI_ISL_456391	LabPLUS	Institute of Environmental Science and Research (ESR)	2020-04-25	Matt Storey et al A
NewZealand/20VR2932/2020	EPI_ISL_456393	LabPLUS	Institute of Environmental Science and Research (ESR)	2020-04-25	Matt Storey et al A
NewZealand/20VR3004/2020	EPI_ISL_456396	North Shore Hospital	Institute of Environmental Science and Research (ESR)	2020-04-25	Matt Storey et al A
NewZealand/20VR3038/2020	EPI_ISL_456400	Wellington SCL	Institute of Environmental Science and Research (ESR)	2020-04-25	Matt Storey et al A
NewZealand/20VR3045/2020	EPI_ISL_456402	Wellington SCL	Institute of Environmental Science and Research (ESR)	2020-04-25	Matt Storey et al A
NewZealand/CoV001/2020	EPI_ISL_417211	Dunedin Hospital	University of Otago	2020-03-11	M.E. Quiñones-Mateu et al
Nigeria/KW017-CV24/2020	EPI_ISL_455362	Nigeria Centre for Disease Control (NCDC)	African Centre of Excellence for Genomics of Infectious Diseases (ACEGID), Redeemer's University, Ede, Osun State, Nigeria	2020-04-10	Oluniyi P.E. et al A (https://virological.org/t/sars-cov-2-genomes-from-nigeria-reveal-community-transmission-multiple-virus-lineages-and-spike-protein-mutation-associated-with-higher-transmission-and-pathogenicity/494)
Nigeria/Lagos01/2020	EPI_ISL_413550	Centre for Human and Zoonotic Virology (CHAZVY), College of Medicine University of Lagos/Lagos University Teaching Hospital (LUTH), part of the Laboratory Network of the Nigeria Centre for Disease Control (NCDC)	African Centre of Excellence for Genomics of Infectious Diseases (ACEGID), Redeemer's University, Ede, Osun State, Nigeria	2020-02-27	Oluniyi P.E. et al B (https://virological.org/t/first-african-sars-cov-2-genome-sequence-from-nigerian-covid-19-case/421)
Nigeria/OS030-CV5/2020	EPI_ISL_455415	Nigeria Centre for Disease Control (NCDC)	African Centre of Excellence for Genomics of Infectious Diseases (ACEGID), Redeemer's University, Ede, Osun State, Nigeria	2020-03-29	Oluniyi P.E. et al A (https://virological.org/t/sars-cov-2-genomes-from-nigeria-reveal-community-transmission-multiple-virus-lineages-and-spike-protein-mutation-associated-with-higher-transmission-and-pathogenicity/494)
Nigeria/OS055-CV8/2020	EPI_ISL_455418	Nigeria Centre for Disease Control (NCDC)	African Centre of Excellence for Genomics of Infectious Diseases (ACEGID), Redeemer's University, Ede, Osun State, Nigeria	2020-03-29	Oluniyi P.E. et al A (https://virological.org/t/sars-cov-2-genomes-from-nigeria-reveal-community-transmission-multiple-virus-lineages-and-spike-protein-mutation-associated-with-higher-transmission-and-pathogenicity/494)
Nigeria/OS060-CV9/2020	EPI_ISL_455419	Nigeria Centre for Disease Control (NCDC)	African Centre of Excellence for Genomics of Infectious Diseases (ACEGID), Redeemer's University, Ede, Osun State, Nigeria	2020-03-29	Oluniyi P.E. et al A (https://virological.org/t/sars-cov-2-genomes-from-nigeria-reveal-community-transmission-multiple-virus-lineages-and-spike-protein-mutation-associated-with-higher-transmission-and-pathogenicity/494)
Nigeria/OS070-CV11/2020	EPI_ISL_455422	Nigeria Centre for Disease Control	African Centre of Excellence for Genomics of Infectious Diseases (ACEGID), Redeemer's University, Ede, Osun State, Nigeria	2020-03-29	Oluniyi P.E. et al A (https://virological.org/t/sars-cov-2-genomes-from-nigeria-reveal-community-transmission-multiple-virus-lineages-and-spike-protein-mutation-associated-with-higher-transmission-and-pathogenicity/494)
Nigeria/OS075-CV12/2020	EPI_ISL_455423	Nigeria Centre for Disease Control (NCDC)	African Centre of Excellence for Genomics of Infectious Diseases (ACEGID), Redeemer's University, Ede, Osun State, Nigeria	2020-03-29	Oluniyi P.E. et al A (https://virological.org/t/sars-cov-2-genomes-from-nigeria-reveal-community-transmission-multiple-virus-lineages-and-spike-protein-mutation-associated-with-higher-transmission-and-pathogenicity/494)
Nigeria/OS085-CV14/2020	EPI_ISL_455424	Nigeria Centre for Disease Control (NCDC)	African Centre of Excellence for Genomics of Infectious Diseases (ACEGID), Redeemer's University, Ede, Osun State, Nigeria	2020-03-29	Oluniyi P.E. et al A (https://virological.org/t/sars-cov-2-genomes-from-nigeria-reveal-community-transmission-multiple-virus-lineages-and-spike-protein-mutation-associated-with-higher-transmission-and-pathogenicity/494)
Nigeria/OS116-CV17/2020	EPI_ISL_455425	Nigeria Centre for Disease Control (NCDC)	African Centre of Excellence for Genomics of Infectious Diseases (ACEGID), Redeemer's University, Ede, Osun State, Nigeria	2020-03-29	Oluniyi P.E. et al A (https://virological.org/t/sars-cov-2-genomes-from-nigeria-reveal-community-transmission-multiple-virus-lineages-and-spike-protein-mutation-associated-with-higher-transmission-and-pathogenicity/494)
Nigeria/OS122-CV18/2020	EPI_ISL_455426	Nigeria Centre for Disease Control	African Centre of Excellence for Genomics of Infectious Diseases (ACEGID), Redeemer's University, Ede, Osun State, Nigeria	2020-03-29	Oluniyi P.E. et al A (https://virological.org/t/sars-cov-2-genomes-from-nigeria-reveal-community-transmission-multiple-virus-lineages-and-spike-protein-mutation-associated-with-higher-transmission-and-pathogenicity/494)
Nigeria/OS125-CV20/2020	EPI_ISL_455427	Nigeria Centre for Disease Control (NCDC)	African Centre of Excellence for Genomics of Infectious Diseases (ACEGID), Redeemer's University, Ede, Osun State, Nigeria	2020-03-29	Oluniyi P.E. et al A (https://virological.org/t/sars-cov-2-genomes-from-nigeria-reveal-community-transmission-multiple-virus-lineages-and-spike-protein-mutation-associated-with-higher-transmission-and-pathogenicity/494)
Nigeria/OY008-CV29/2020	EPI_ISL_455429	Nigeria Centre for Disease Control (NCDC)	African Centre of Excellence for Genomics of Infectious Diseases (ACEGID), Redeemer's University, Ede, Osun State, Nigeria	2020-03-27	Oluniyi P.E. et al A (https://virological.org/t/sars-cov-2-genomes-from-nigeria-reveal-community-transmission-multiple-virus-lineages-and-spike-protein-mutation-associated-with-higher-transmission-and-pathogenicity/494)
NorthernIreland/HSCNI01/2020	EPI_ISL_414949	Regional Virus Laboratory, Belfast	Public Health Wales Microbiology Cardiff	2020-02-26	Tanya Curran et al (https://virological.org/t/preliminary-analysis-of-sars-cov-2-importation-establishment-of-uk-transmission-lineages/507)
NorthernIreland/NIRE-FA6AD/2020	EPI_ISL_459393	Regional Virus Laboratory, Belfast Health and Social Care Trust	Wellcome Sanger Institute for the COVID-19 Genomics UK Consortium	2020-04-06	Conall McCaughey et al A
NorthernIreland/NIRE-FAE4B/2020	EPI_ISL_448928	Regional Virus Laboratory, Belfast Health and Social Care Trust	COVID-19 Genomics UK (COG-UK) Consortium	2020-03-26	Conall McCaughey et al B (https://virological.org/t/preliminary-analysis-of-sars-cov-2-importation-establishment-of-uk-transmission-lineages/507)
NorthernIreland/NIRE-FB105/2020	EPI_ISL_470026	Regional Virus Laboratory, Belfast Health and Social Care Trust	Wellcome Sanger Institute for the COVID-19 Genomics UK Consortium	2020-04-07	Conall McCaughey et al A
NorthernIreland/NIRE-FB77C/2020	EPI_ISL_448976	Regional Virus Laboratory, Belfast Health and Social Care Trust	COVID-19 Genomics UK (COG-UK) Consortium	2020-03-25	Conall McCaughey et al B (https://virological.org/t/preliminary-analysis-of-sars-cov-2-importation-establishment-of-uk-transmission-lineages/507)
Norway/1380/2020	EPI_ISL_417484	Oslo University Hospital, Department of Medical Microbiology	Norwegian Institute of Public Health, Department of Virology	2020-02-26	Kathrine Stene-Johansen et al
Norway/1495/2020	EPI_ISL_420134	Akershus University Hospital, Department for Microbiology and Infectious Disease Control	Norwegian Institute of Public Health, Department of Virology	2020-02-28	Kathrine Stene-Johansen et al
Norway/1526/2020	EPI_ISL_417485	University Hospital of Northern Norway, Department for Microbiology and Infectious Disease Control	Norwegian Institute of Public Health, Department of Virology	2020-03-02	Kathrine Stene-Johansen et al
Norway/1781/2020	EPI_ISL_420138	University Hospital of Northern Norway, Department for Microbiology and Infectious Disease Control	Norwegian Institute of Public Health, Department of Virology	2020-03-05	Kathrine Stene-Johansen et al
Norway/1867/2020	EPI_ISL_420141	Furst Medical Laboratory	Norwegian Institute of Public Health, Department of Virology	2020-03-10	Kathrine Stene-Johansen et al
Norway/1917/2020	EPI_ISL_420143	Unilabs Laboratory Medicine	Norwegian Institute of Public Health, Department of Virology	2020-03-10	Kathrine Stene-Johansen et al
Norway/1949/2020	EPI_ISL_470882	Foerde Hospital, Department of Microbiology	Norwegian Institute of Public Health, Department of Virology	2020-03-09	Kathrine Stene-Johansen et al

Norway/1951/2020	EPI_ISL_420145	Forde Hospital Department of Microbiology	Norwegian Institute of Public Health, Department of Virology	2020-03-09	Kathrine Stene-Johansen et al
Norway/1953/2020	EPI_ISL_420313	Furst Medical Laboratory	Norwegian Institute of Public Health, Department of Virology	2020-03-10	Kathrine Stene-Johansen et al
Norway/1989/2020	EPI_ISL_420147	Hospital of Southern Norway - Kristiansand, Department of Medical Microbiology	Norwegian Institute of Public Health, Department of Virology	2020-03-10	Kathrine Stene-Johansen et al
Norway/2065/2020	EPI_ISL_420149	Stavanger University Hospital, Department of Medical Microbiology	Norwegian Institute of Public Health, Department of Virology	2020-03-09	Kathrine Stene-Johansen et al
Norway/2084/2020	EPI_ISL_420150	Oslo University Hospital, Department of Medical Microbiology	Norwegian Institute of Public Health, Department of Virology	2020-03-09	Kathrine Stene-Johansen et al
Norway/2087/2020	EPI_ISL_420151	Nordland Hospital - Bodo, Laboratory Department, Molecular Biology Unit	Norwegian Institute of Public Health, Department of Virology	2020-03-17	Kathrine Stene-Johansen et al
Norway/2088/2020	EPI_ISL_420152	University Hospital of Northern Norway, Department for Microbiology and Infectious Disease Control	Norwegian Institute of Public Health, Department of Virology	2020-03-17	Kathrine Stene-Johansen et al
Norway/2090/2020	EPI_ISL_420153	University Hospital of Northern Norway, Department for Microbiology and Infectious Disease Control	Norwegian Institute of Public Health, Department of Virology	2020-03-16	Kathrine Stene-Johansen et al
Norway/2093/2020	EPI_ISL_420310	University Hospital of Northern Norway, Department for Microbiology and Infectious Disease Control	Norwegian Institute of Public Health, Department of Virology	2020-03-16	Kathrine Stene-Johansen et al
Norway/2113/2020	EPI_ISL_420311	Akershus University Hospital, Department for Microbiology and Infectious Disease Control	Norwegian Institute of Public Health, Department of Virology	2020-03-18	Kathrine Stene-Johansen et al
Norway/2114/2020	EPI_ISL_420312	Akershus University Hospital, Department for Microbiology and Infectious Disease Control	Norwegian Institute of Public Health, Department of Virology	2020-03-18	Kathrine Stene-Johansen et al
Norway/2200/2020	EPI_ISL_447837	Dept. of Medical Microbiology, Stavanger University Hospital, Helse Stavanger HF,	Norwegian Institute of Public Health, Department of Virology	2020-03-20	Kathrine Stene-Johansen et al
Norway/2244/2020	EPI_ISL_447838	Medical Microbiology Unit, Department for Laboratory Medicine, Drammen Hospital, Vestre Viken Health Trust,	Norwegian Institute of Public Health, Department of Virology	2020-03-23	Kathrine Stene-Johansen et al
Norway/2246/2020	EPI_ISL_447839	Medical Microbiology Unit, Department for Laboratory Medicine, Drammen Hospital, Vestre Viken Health Trust,	Norwegian Institute of Public Health, Department of Virology	2020-03-24	Kathrine Stene-Johansen et al
Norway/2356-2/2020	EPI_ISL_449786	Ostfold Hospital Trust - Kalnes, Centre for Laboratory Medicine, Section for gene technology and infection serology	Norwegian Institute of Public Health, Department of Virology	2020-04-08	Kathrine Stene-Johansen et al
Norway/2380/2020	EPI_ISL_449787	Furst Medical Laboratory	Norwegian Institute of Public Health, Department of Virology	2020-04-12	Kathrine Stene-Johansen et al
Norway/2381/2020	EPI_ISL_449788	Furst Medical Laboratory	Norwegian Institute of Public Health, Department of Virology	2020-04-11	Kathrine Stene-Johansen et al
Norway/2382/2020	EPI_ISL_449789	Dept. of Medical Microbiology, Stavanger University Hospital, Helse Stavanger HF	Norwegian Institute of Public Health, Department of Virology	2020-03-30	Kathrine Stene-Johansen et al
Norway/2383/2020	EPI_ISL_449790	Dept. of Medical Microbiology, Stavanger University Hospital, Helse Stavanger HF	Norwegian Institute of Public Health, Department of Virology	2020-04-01	Kathrine Stene-Johansen et al
Norway/2386/2020	EPI_ISL_449791	Dept. of Medical Microbiology, Stavanger University Hospital, Helse Stavanger HF	Norwegian Institute of Public Health, Department of Virology	2020-04-04	Kathrine Stene-Johansen et al
Norway/2387/2020	EPI_ISL_449792	Dept. of Medical Microbiology, Stavanger University Hospital, Helse Stavanger HF	Norwegian Institute of Public Health, Department of Virology	2020-04-05	Kathrine Stene-Johansen et al
Norway/2388/2020	EPI_ISL_449793	Dept. of Medical Microbiology, Stavanger University Hospital, Helse Stavanger HF	Norwegian Institute of Public Health, Department of Virology	2020-04-02	Kathrine Stene-Johansen et al
Norway/2390/2020	EPI_ISL_449794	Dept. of Medical Microbiology, Stavanger University Hospital, Helse Stavanger HF	Norwegian Institute of Public Health, Department of Virology	2020-04-02	Kathrine Stene-Johansen et al
Norway/Trondheim-E10/2020	EPI_ISL_450352	St.Olavs hospital/NTNU	Institute of Genomics Core Facility, University of Tartu	2020-03-11	Aleksandr Ianevski et al
Norway/Trondheim-E9/2020	EPI_ISL_450351	St.Olavs hospital/NTNU	Institute of Genomics Core Facility, University of Tartu	2020-03-11	Aleksandr Ianevski et al
Norway/Trondheim-S10/2020	EPI_ISL_450348	St.Olavs hospital/NTNU	Institute of Genomics Core Facility, University of Tartu	2020-03-24	Aleksandr Ianevski et al
Norway/Trondheim-S12/2020	EPI_ISL_450349	St.Olavs hospital/NTNU	Institute of Genomics Core Facility, University of Tartu	2020-03-29	Aleksandr Ianevski et al
Norway/Trondheim-S15/2020	EPI_ISL_450350	St.Olavs hospital/NTNU	Institute of Genomics Core Facility, University of Tartu	2020-03-28	Aleksandr Ianevski et al
Norway/Trondheim-S4/2020	EPI_ISL_450346	St.Olavs hospital/NTNU	Institute of Genomics Core Facility, University of Tartu	2020-03-25	Aleksandr Ianevski et al
Norway/Trondheim-S5/2020	EPI_ISL_450347	St.Olavs hospital/NTNU	Institute of Genomics Core Facility, University of Tartu	2020-03-25	Aleksandr Ianevski et al
Oman/205024076/2020	EPI_ISL_457707	Oman-NIC	Department of Microbiology and Immunology- SQUH	2020-03-24	Fahad Zadjali et al
Oman/205024123/2020	EPI_ISL_457703	Oman-NIC	Department of Microbiology and Immunology- SQUH	2020-03-25	Fahad Zadjali et al
Oman/205026358/2020	EPI_ISL_457981	Oman-NIC	Department of Microbiology and Immunology-SQUH	2020-04-03	Fahad Zadjali et al
Oman/205026721/2020	EPI_ISL_458127	Oman National Influenza Centre	Department of Microbiology and Immunology-SQUH	2020-04-05	Fahad Zadjali et al
Oman/205027285/2020	EPI_ISL_458122	Oman National Influenza Centre	Department of Microbiology and Immunology-SQUH	2020-04-07	Fahad Zadjali et al
Oman/205027556/2020	EPI_ISL_458125	Oman National Influenza Centre	Department of Microbiology and Immunology-SQUH	2020-04-08	Fahad Zadjali et al
Oman/205028472/2020	EPI_ISL_458119	Oman National Influenza Centre	Department of Microbiology and Immunology-SQUH	2020-04-11	Fahad Zadjali et al
Oman/205028718/2020	EPI_ISL_457702	Oman-NIC	Microbiology laboratory- Sultan Qaboos University Hospital	2020-04-12	Fahad Zadjali et al
Oman/205028997/2020	EPI_ISL_458120	Oman National Influenza Centre	Department of Microbiology and Immunology-SQUH	2020-04-13	Fahad Zadjali et al
Oman/205029065/2020	EPI_ISL_457982	Oman-NIC	Department of Microbiology and Immunology-SQUH	2020-04-13	Fahad Zadjali et al
Oman/205030448/2020	EPI_ISL_458121	Oman National Influenza Centre	Department of Microbiology and Immunology-SQUH	2020-04-20	Fahad Zadjali et al
Oman/205031572/2020	EPI_ISL_458126	Oman National Influenza Centre	Department of Microbiology and Immunology-SQUH	2020-04-25	Fahad Zadjali et al
Oman/205032386/2020	EPI_ISL_457984	Oman-NIC	Department of Microbiology and Immunology-SQUH	2020-04-28	Fahad Zadjali et al
Oman/205033010/2020	EPI_ISL_458118	Oman National Influenza Centre	Department of Microbiology and Immunology-SQUH	2020-05-01	Fahad Zadjali et al
Oman/205033013/2020	EPI_ISL_458116	Oman National Influenza Centre	Department of Microbiology and Immunology-SQUH	2020-05-01	Fahad Zadjali et al
Oman/205033293/2020	EPI_ISL_457983	Oman-NIC	Department of Microbiology and Immunology-SQUH	2020-05-03	Fahad Zadjali et al
Oman/205034466/2020	EPI_ISL_457705	OMAN-NIC	Department of Microbiology and Immunology- SQUH	2020-05-08	Fahad Zadjali et al
Oman/205034603/2020	EPI_ISL_458117	Oman National Influenza Centre	Department of Microbiology and Immunology-SQUH	2020-05-09	Fahad Zadjali et al
Oman/RESP-20-1189/2020	EPI_ISL_457706	Oman-NIC	Oman-NIC	2020-03-02	Samira Al-Maruqi et al
Oman/RESP-20-1857/2020	EPI_ISL_457937	Oman-NIC	Oman-NIC	2020-03-15	Samira Al-Maruqi et al
Oman/RESP-20-1976/2020	EPI_ISL_457938	Oman-NIC	Oman-NIC	2020-03-15	Samira Al-Maruqi et al
Oman/RESP-20-2181/2020	EPI_ISL_457939	Oman-NIC	Oman-NIC	2020-03-17	Samira Al-Maruqi et al
Oman/RESP-20-2282/2020	EPI_ISL_457974	Oman-NIC	Oman-NIC	2020-03-14	Samira Al-Maruqi et al
Oman/RESP-20-2523/2020	EPI_ISL_457975	Oman-NIC	Oman-NIC	2020-03-17	Samira Al-Maruqi et al
Oman/RESP-20-2631/2020	EPI_ISL_457976	Oman-NIC	Oman-NIC	2020-03-17	Samira Al-Maruqi et al
Oman/RESP-20-2850/2020	EPI_ISL_457977	Oman-NIC	Oman-NIC	2020-03-18	Samira Al-Maruqi et al
Oman/RESP-20-2857/2020	EPI_ISL_457978	Oman-NIC	Oman-NIC	2020-03-18	Samira Al-Maruqi et al
Oman/RESP-20-3080/2020	EPI_ISL_457979	Oman-NIC	Oman-NIC	2020-03-18	Samira Al-Maruqi et al
Oman/RESP-20-3343/2020	EPI_ISL_457985	Oman-NIC	Oman-NIC	2020-03-18	Samira Al-Maruqi et al
Oman/RESP-20-3515/2020	EPI_ISL_457980	Oman-NIC	Oman-NIC	2020-03-18	Samira Al-Maruqi et al
Oman/RESP-20-3700/2020	EPI_ISL_457986	Oman-NIC	Oman-NIC	2020-03-20	Samira Al-Maruqi et al
Oman/RESP-20-4153/2020	EPI_ISL_457988	Oman-NIC	Oman-NIC	2020-03-21	Samira Al-Maruqi et al
Oman/RESP-20-4252/2020	EPI_ISL_457989	Oman-NIC	Oman-NIC	2020-03-21	Samira Al-Maruqi et al
Oman/RESP-20-4736/2020	EPI_ISL_457991	Oman-NIC	Oman-NIC	2020-03-22	Samira Al-Maruqi et al
Oman/RESP-20-4810/2020	EPI_ISL_457992	Oman-NIC	Oman-NIC	2020-03-22	Samira Al-Maruqi et al
Oman/RESP-20-5414/2020	EPI_ISL_457993	Oman-NIC	Oman-NIC	2020-03-24	Samira Al-Maruqi et al
Oman/RESP-20-5505/2020	EPI_ISL_457994	Oman-NIC	Oman-NIC	2020-03-25	Samira Al-Maruqi et al
Oman/RESP-20-6417/2020	EPI_ISL_457996	Oman-NIC	Oman-NIC	2020-03-29	Samira Al-Maruqi et al
Oman/RESP-20-6560/2020	EPI_ISL_457997	Oman-NIC	Oman-NIC	2020-03-28	Samira Al-Maruqi et al
Oman/RESP-20-6701/2020	EPI_ISL_457998	Oman-NIC	Oman-NIC	2020-03-28	Samira Al-Maruqi et al
Oman/RESP-20-797/2020	EPI_ISL_457701	Oman-NIC	Oman-NIC	2020-02-23	Samira Al-Maruqi et al
Oman/RESP-20-837/2020	EPI_ISL_457704	Oman-NIC	Oman-NIC	2020-02-24	Samira Al-Maruqi et al
Pakistan/Gilgit1/2020	EPI_ISL_417444		Department of Healthcare Biotechnology	2020-03-04	Javed et al (http://biorxiv.org/lookup/doi/10.1101/2020.04.01.019463)
Pakistan/KHI1/2020	EPI_ISL_451958		Jamil-ur-Rahman Center for Genome Research	2020-03-16	Shakeel et al
Pakistan/NIH-44905/2020	EPI_ISL_468159		Department of Virology, Public Health Laboratories Division, National Institute of Health	2020-06-02	Massab Umair et al
Pakistan/NIH-45090/2020	EPI_ISL_468161	Department of Virology, Public Health Laboratories Division, National Institute of Health	Department of Virology, Public Health Laboratories Division, National Institute of Health	2020-06-02	Massab Umair et al
Pakistan/NIH-45143/2020	EPI_ISL_468160		Department of Virology, Public Health Laboratories Division, National Institute of Health	2020-06-02	Massab Umair et al
Pakistan/NIH-45579/2020	EPI_ISL_468162		Department of Virology, Public Health Laboratories Division, National Institute of Health	2020-06-02	Massab Umair et al
Pakistan/NIH-HAS001/2020	EPI_ISL_468163	Department of Virology, Public Health Laboratories Division, National Institute of Health	Department of Virology, Public Health Laboratories Division, National Institute of Health	2020-06-02	Massab Umair et al
Panama/328677/2020	EPI_ISL_415152	Gorgas Memorial Institute for Health Studies	Gorgas Memorial Institute for Health Studies	2020-03-06	Danilo Franco et al
Peru/010/2020	EPI_ISL_415787	Laboratorio de Referencia Nacional de Virus Respiratorio. Instituto Nacional de Salud. Peru	Laboratorio de Referencia Nacional de Biotecnologia y Biologia Molecular.Instituto Nacional de Salud.Peru	2020-03-10	Carlos Padilla Rojas et al (https://dx.doi.org/10.1101/2020.05.23.20111443)
Philippines/PGC01/2020	EPI_ISL_431833	National Institutes of Health, University of the Philippines Manila	Philippine Genome Center, University of the Philippines System	2020-03-22	Carlo M. Lapid et al
Philippines/PGC02/2020	EPI_ISL_434554	National Institutes of Health, University of the Philippines Manila	Philippine Genome Center	2020-03-26	Carlo M. Lapid et al
Philippines/PGC03/2020	EPI_ISL_434555	National Institutes of Health, University of the Philippines Manila	Philippine Genome Center	2020-03-26	Carlo M. Lapid et al
Philippines/PGC04/2020	EPI_ISL_434556	National Institutes of Health, University of the Philippines Manila	Philippine Genome Center	2020-03-26	Carlo M. Lapid et al
Philippines/PGC05/2020	EPI_ISL_434557	National Institutes of Health, University of the Philippines Manila	Philippine Genome Center	2020-03-27	Carlo M. Lapid et al
Philippines/PGC06/2020	EPI_ISL_434558	National Institutes of Health, University of the Philippines Manila	Philippine Genome Center	2020-03-28	Carlo M. Lapid et al
Philippines/RITM-03/2020	EPI_ISL_430456	Rizal Medical Center	Research Institute for Tropical Medicine	2020-03-21	Medado et al
Philippines/RITM-04/2020	EPI_ISL_430840	Veterans Memorial Medical Center	Research Institute for Tropical Medicine	2020-03-08	Medado et al
Philippines/RITM-07/2020	EPI_ISL_430845	Pasig City General Hospital	Research Institute for Tropical Medicine	2020-03-23	Medado et al
Poland/1104795/2020	EPI_ISL_428232	Hematology Laboratory, Section of Molecular Diagnostics, University Clinical Centre, Medical University of Gdansk	Department of Virology, Faculty of Medicine, University of Helsinki, Helsinki, Finland	2020-03-18	Marlena Robakowska et al
Poland/1105644/2020	EPI_ISL_428233	Hematology Laboratory, Section of Molecular Diagnostics, University Clinical Centre, Medical University of Gdansk	Department of Virology, Faculty of Medicine, University of Helsinki, Helsinki, Finland	2020-03-18	Marlena Robakowska et al
Poland/1105973/2020	EPI_ISL_428234	Hematology Laboratory, Section of Molecular Diagnostics, University Clinical Centre, Medical University of Gdansk	Department of Virology, Faculty of Medicine, University of Helsinki, Helsinki, Finland	2020-03-19	Marlena Robakowska et al
Poland/1107355/2020	EPI_ISL_428235	Hematology Laboratory, Section of Molecular Diagnostics, University Clinical Centre, Medical University of Gdansk	Department of Virology, Faculty of Medicine, University of Helsinki, Helsinki, Finland	2020-03-23	Marlena Robakowska et al
Poland/1109171/2020	EPI_ISL_428236	Hematology Laboratory, Section of Molecular Diagnostics, University Clinical Centre, Medical University of Gdansk	Department of Virology, Faculty of Medicine, University of Helsinki, Helsinki, Finland	2020-03-27	Marlena Robakowska et al
Poland/1109201/2020	EPI_ISL_450525	Hematology Laboratory, Section of Molecular Diagnostics, University Clinical Centre, Medical University of Gdansk	Department of Virology, Faculty of Medicine, University of Helsinki, Helsinki, Finland	2020-03-28	Maciej Grzybek et al
Poland/1110540/2020	EPI_ISL_450528	Hematology Laboratory, Section of Molecular Diagnostics, University Clinical Centre, Medical University of Gdansk	Department of Virology, Faculty of Medicine, University of Helsinki, Helsinki, Finland	2020-03-30	Maciej Grzybek et al
Poland/1110551/2020	EPI_ISL_450527	Hematology Laboratory, Section of Molecular Diagnostics, University Clinical Centre, Medical University of Gdansk	Department of Virology, Faculty of Medicine, University of Helsinki, Helsinki, Finland	2020-03-29	Maciej Grzybek et al
Poland/1111628/2020	EPI_ISL_450530	Hematology Laboratory, Section of Molecular Diagnostics, University Clinical Centre, Medical University of Gdansk	Department of Virology, Faculty of Medicine, University of Helsinki, Helsinki, Finland	2020-04-01	Maciej Grzybek et al
Poland/IHG_PAS_1_69/2020	EPI_ISL_450294	Institute of Human Genetics, Polish Academy of Sciences Sanitary and Epidemiological Station in Poznań	Institute of Human Genetics, Polish Academy of Sciences	2020-04-11	Szymon Hryhorowicz et al
Poland/IHG_PAS_1_73/2020	EPI_ISL_450295	Institute of Human Genetics, Polish Academy of Sciences; Sanitary and Epidemiological Station in Poznań	Institute of Human Genetics, Polish Academy of Sciences	2020-04-11	Szymon Hryhorowicz et al
Poland/IHG_PAS_1_77/2020	EPI_ISL_450338	Institute of Human Genetics, Polish Academy of Sciences; Sanitary and Epidemiological Station in Poznań	Institute of Human Genetics, Polish Academy of Sciences	2020-04-11	Szymon Hryhorowicz et al
Poland/IHG_PAS_3_57/2020	EPI_ISL_462480	Institute of Human Genetics, Polish Academy of Sciences	Institute of Human Genetics, Polish Academy of Sciences	2020-05-13	Szymon Hryhorowicz et al
Poland/PL_P1/2020	EPI_ISL_416488	ViroGenetics - BSL3 Laboratory of Virology; Human Genome Variation Research Group & Genomics Centre MCB; Bioinformatics Research Group Department of Virology	ViroGenetics - BSL3 Laboratory of Virology; Human Genome Variation Research Group & Genomics Centre MCB; Bioinformatics Research Group Department of Virology	2020-03-03	Aleksandra Milewska et al
Poland/PL_P10/2020	EPI_ISL_451971	1. ViroGenetics - BSL3 Laboratory of Virology, Małopolska Centre of Biotechnology, Jagiellonian University; 2. II Department of Internal Medicine, Faculty of Medicine, Jagiellonian University Medical College; 3. DIAGNOSTYKA Ltd.	1. ViroGenetics - BSL3 Laboratory of Virology, Małopolska Centre of Biotechnology, Jagiellonian University; 2. II Department of Internal Medicine, Faculty of Medicine, Jagiellonian University Medical College.	2020-04-01	Marek Sanak et al
Poland/PL_P11/2020	EPI_ISL_451972	1. ViroGenetics - BSL3 Laboratory of Virology, Małopolska Centre of Biotechnology, Jagiellonian University; 2. II Department of Internal Medicine, Faculty of Medicine, Jagiellonian University Medical College; 3. DIAGNOSTYKA Ltd.	1. ViroGenetics - BSL3 Laboratory of Virology, Małopolska Centre of Biotechnology, Jagiellonian University; 2. II Department of Internal Medicine, Faculty of Medicine, Jagiellonian University Medical College.	2020-04-02	Marek Sanak et al
Poland/PL_P12/2020	EPI_ISL_451973	1. ViroGenetics - BSL3 Laboratory of Virology, Małopolska Centre of Biotechnology, Jagiellonian University; 2. II Department of Internal	1. ViroGenetics - BSL3 Laboratory of Virology, Małopolska Centre of Biotechnology, Jagiellonian University; 2. II Department	2020-03-31	Marek Sanak et al

		Medicine, Faculty of Medicine, Jagiellonian University Medical College; 3. DIAGNOSTYKA Ltd.	of Internal Medicine, Faculty of Medicine, Jagiellonian University Medical College.		
Poland/PL_P13/2020	EPI_ISL_451974	1. ViroGenetics - BSL3 Laboratory of Virology, Małopolska Centre of Biotechnology, Jagiellonian University; 2. II Department of Internal Medicine, Faculty of Medicine, Jagiellonian University Medical College; 3. DIAGNOSTYKA Ltd.	1. ViroGenetics - BSL3 Laboratory of Virology, Małopolska Centre of Biotechnology, Jagiellonian University; 2. II Department of Internal Medicine, Faculty of Medicine, Jagiellonian University Medical College.	2020-04-03	Marek Sanak et al
Poland/PL_P14/2020	EPI_ISL_451975	1. ViroGenetics - BSL3 Laboratory of Virology, Małopolska Centre of Biotechnology, Jagiellonian University; 2. II Department of Internal Medicine, Faculty of Medicine, Jagiellonian University Medical College; 3. DIAGNOSTYKA Ltd.	1. ViroGenetics - BSL3 Laboratory of Virology, Małopolska Centre of Biotechnology, Jagiellonian University; 2. II Department of Internal Medicine, Faculty of Medicine, Jagiellonian University Medical College.	2020-03-31	Marek Sanak et al
Poland/PL_P15/2020	EPI_ISL_451976	1. ViroGenetics - BSL3 Laboratory of Virology, Małopolska Centre of Biotechnology, Jagiellonian University; 2. II Department of Internal Medicine, Faculty of Medicine, Jagiellonian University Medical College; 3. DIAGNOSTYKA Ltd.	1. ViroGenetics - BSL3 Laboratory of Virology, Małopolska Centre of Biotechnology, Jagiellonian University; 2. II Department of Internal Medicine, Faculty of Medicine, Jagiellonian University Medical College.	2020-04-07	Marek Sanak et al
Poland/PL_P16/2020	EPI_ISL_451977	1. ViroGenetics - BSL3 Laboratory of Virology, Małopolska Centre of Biotechnology, Jagiellonian University; 2. II Department of Internal Medicine, Faculty of Medicine, Jagiellonian University Medical College; 3. DIAGNOSTYKA Ltd.	1. ViroGenetics - BSL3 Laboratory of Virology, Małopolska Centre of Biotechnology, Jagiellonian University; 2. II Department of Internal Medicine, Faculty of Medicine, Jagiellonian University Medical College.	2020-04-03	Marek Sanak et al
Poland/PL_P17/2020	EPI_ISL_451978	1. ViroGenetics - BSL3 Laboratory of Virology, Małopolska Centre of Biotechnology, Jagiellonian University; 2. II Department of Internal Medicine, Faculty of Medicine, Jagiellonian University Medical College; 3. DIAGNOSTYKA Ltd.	1. ViroGenetics - BSL3 Laboratory of Virology, Małopolska Centre of Biotechnology, Jagiellonian University; 2. II Department of Internal Medicine, Faculty of Medicine, Jagiellonian University Medical College.	2020-04-01	Marek Sanak et al
Poland/PL_P18/2020	EPI_ISL_451979	1. ViroGenetics - BSL3 Laboratory of Virology, Małopolska Centre of Biotechnology, Jagiellonian University; 2. II Department of Internal Medicine, Faculty of Medicine, Jagiellonian University Medical College; 3. DIAGNOSTYKA Ltd.	1. ViroGenetics - BSL3 Laboratory of Virology, Małopolska Centre of Biotechnology, Jagiellonian University; 2. II Department of Internal Medicine, Faculty of Medicine, Jagiellonian University Medical College.	2020-03-31	Marek Sanak et al
Poland/PL_P19/2020	EPI_ISL_451980	1. ViroGenetics - BSL3 Laboratory of Virology, Małopolska Centre of Biotechnology, Jagiellonian University; 2. II Department of Internal Medicine, Faculty of Medicine, Jagiellonian University Medical College; 3. DIAGNOSTYKA Ltd.	1. ViroGenetics - BSL3 Laboratory of Virology, Małopolska Centre of Biotechnology, Jagiellonian University; 2. II Department of Internal Medicine, Faculty of Medicine, Jagiellonian University Medical College.	2020-04-02	Marek Sanak et al
Poland/PL_P2/2020	EPI_ISL_428925	ViroGenetics - BSL3 Laboratory of Virology; Human Genome Variation Research Group & Genomics Centre MCB; Bioinformatics Research Group; Wojewódzka Stacja Sanitarno-Epidemiologiczna w Krakowie	ViroGenetics - BSL3 Laboratory of Virology; Human Genome Variation Research Group & Genomics Centre MCB; Bioinformatics Research Group	2020-03-28	Wojciech Branicki et al
Poland/PL_P20/2020	EPI_ISL_451981	1. ViroGenetics - BSL3 Laboratory of Virology, Małopolska Centre of Biotechnology, Jagiellonian University; 2. II Department of Internal Medicine, Faculty of Medicine, Jagiellonian University Medical College; 3. DIAGNOSTYKA Ltd.	1. ViroGenetics - BSL3 Laboratory of Virology, Małopolska Centre of Biotechnology, Jagiellonian University; 2. II Department of Internal Medicine, Faculty of Medicine, Jagiellonian University Medical College.	2020-04-07	Marek Sanak et al
Poland/PL_P22/2020	EPI_ISL_451983	1. ViroGenetics - BSL3 Laboratory of Virology, Małopolska Centre of Biotechnology, Jagiellonian University; 2. II Department of Internal Medicine, Faculty of Medicine, Jagiellonian University Medical College; 3. DIAGNOSTYKA Ltd.	1. ViroGenetics - BSL3 Laboratory of Virology, Małopolska Centre of Biotechnology, Jagiellonian University; 2. II Department of Internal Medicine, Faculty of Medicine, Jagiellonian University Medical College.	2020-04-02	Marek Sanak et al
Poland/PL_P23/2020	EPI_ISL_451984	1. ViroGenetics - BSL3 Laboratory of Virology, Małopolska Centre of Biotechnology, Jagiellonian University; 2. II Department of Internal Medicine, Faculty of Medicine, Jagiellonian University Medical College; 3. DIAGNOSTYKA Ltd.	1. ViroGenetics - BSL3 Laboratory of Virology, Małopolska Centre of Biotechnology, Jagiellonian University; 2. II Department of Internal Medicine, Faculty of Medicine, Jagiellonian University Medical College.	2020-03-29	Marek Sanak et al
Poland/PL_P25/2020	EPI_ISL_451986	1. ViroGenetics - BSL3 Laboratory of Virology, Małopolska Centre of Biotechnology, Jagiellonian University; 2. II Department of Internal Medicine, Faculty of Medicine, Jagiellonian University Medical College; 3. DIAGNOSTYKA Ltd.	1. ViroGenetics - BSL3 Laboratory of Virology, Małopolska Centre of Biotechnology, Jagiellonian University; 2. II Department of Internal Medicine, Faculty of Medicine, Jagiellonian University Medical College.	2020-04-07	Marek Sanak et al
Poland/PL_P26/2020	EPI_ISL_451987	1. ViroGenetics - BSL3 Laboratory of Virology, Małopolska Centre of Biotechnology, Jagiellonian University; 2. II Department of Internal Medicine, Faculty of Medicine, Jagiellonian University Medical College; 3. DIAGNOSTYKA Ltd.	1. ViroGenetics - BSL3 Laboratory of Virology, Małopolska Centre of Biotechnology, Jagiellonian University; 2. II Department of Internal Medicine, Faculty of Medicine, Jagiellonian University Medical College.	2020-04-01	Marek Sanak et al
Poland/PL_P27/2020	EPI_ISL_455440	1. ViroGenetics - BSL3 Laboratory of Virology, Małopolska Centre of Biotechnology, Jagiellonian University; 2. II Department of Internal Medicine, Faculty of Medicine, Jagiellonian University Medical College; 3. Narodowy Instytut Zdrowia Publicznego – Państwowy Zakład Higieny (NIZP-PZH)	1. ViroGenetics - BSL3 Laboratory of Virology, Małopolska Centre of Biotechnology, Jagiellonian University; 2. II Department of Internal Medicine, Faculty of Medicine, Jagiellonian University Medical College; 3. Narodowy Instytut Zdrowia Publicznego – Państwowy Zakład Higieny (NIZP-PZH).	2020-03-27	Katarzyna Pancer et al
Poland/PL_P28/2020	EPI_ISL_455441	1. ViroGenetics - BSL3 Laboratory of Virology, Małopolska Centre of Biotechnology, Jagiellonian University; 2. II Department of Internal Medicine, Faculty of Medicine, Jagiellonian University Medical College; 3. Narodowy Instytut Zdrowia Publicznego – Państwowy Zakład Higieny (NIZP-PZH)	1. ViroGenetics - BSL3 Laboratory of Virology, Małopolska Centre of Biotechnology, Jagiellonian University; 2. II Department of Internal Medicine, Faculty of Medicine, Jagiellonian University Medical College; 3. Narodowy Instytut Zdrowia Publicznego – Państwowy Zakład Higieny (NIZP-PZH).	2020-04-01	Katarzyna Pancer et al
Poland/PL_P29/2020	EPI_ISL_455442	1. ViroGenetics - BSL3 Laboratory of Virology, Małopolska Centre of Biotechnology, Jagiellonian University; 2. II Department of Internal Medicine, Faculty of Medicine, Jagiellonian University Medical College; 3. Narodowy Instytut Zdrowia Publicznego – Państwowy Zakład Higieny (NIZP-PZH)	1. ViroGenetics - BSL3 Laboratory of Virology, Małopolska Centre of Biotechnology, Jagiellonian University; 2. II Department of Internal Medicine, Faculty of Medicine, Jagiellonian University Medical College; 3. Narodowy Instytut Zdrowia Publicznego – Państwowy Zakład Higieny (NIZP-PZH).	2020-03-18	Katarzyna Pancer et al
Poland/PL_P3/2020	EPI_ISL_428926	ViroGenetics - BSL3 Laboratory of Virology; Human Genome Variation Research Group & Genomics Centre MCB; Bioinformatics Research Group; Wojewódzka Stacja Sanitarno-Epidemiologiczna w Krakowie	ViroGenetics - BSL3 Laboratory of Virology; Human Genome Variation Research Group & Genomics Centre MCB; Bioinformatics Research Group	2020-03-28	Wojciech Branicki et al
Poland/PL_P30/2020	EPI_ISL_455443	1. ViroGenetics - BSL3 Laboratory of Virology, Małopolska Centre of Biotechnology, Jagiellonian University; 2. II Department of Internal Medicine, Faculty of Medicine, Jagiellonian University Medical College; 3. Narodowy Instytut Zdrowia Publicznego – Państwowy Zakład Higieny (NIZP-PZH)	1. ViroGenetics - BSL3 Laboratory of Virology, Małopolska Centre of Biotechnology, Jagiellonian University; 2. II Department of Internal Medicine, Faculty of Medicine, Jagiellonian University Medical College; 3. Narodowy Instytut Zdrowia Publicznego – Państwowy Zakład Higieny (NIZP-PZH).	2020-04-01	Katarzyna Pancer et al
Poland/PL_P31/2020	EPI_ISL_455444	1. ViroGenetics - BSL3 Laboratory of Virology, Małopolska Centre of Biotechnology, Jagiellonian University; 2. II Department of Internal Medicine, Faculty of Medicine, Jagiellonian University Medical College; 3. Narodowy Instytut Zdrowia Publicznego – Państwowy Zakład Higieny (NIZP-PZH)	1. ViroGenetics - BSL3 Laboratory of Virology, Małopolska Centre of Biotechnology, Jagiellonian University; 2. II Department of Internal Medicine, Faculty of Medicine, Jagiellonian University Medical College; 3. Narodowy Instytut Zdrowia Publicznego – Państwowy Zakład Higieny (NIZP-PZH).	2020-03-05	Katarzyna Pancer et al
Poland/PL_P32/2020	EPI_ISL_455445	1. ViroGenetics - BSL3 Laboratory of Virology, Małopolska Centre of Biotechnology, Jagiellonian University; 2. II Department of Internal Medicine, Faculty of Medicine, Jagiellonian University Medical College; 3. Narodowy Instytut Zdrowia Publicznego – Państwowy Zakład Higieny (NIZP-PZH)	1. ViroGenetics - BSL3 Laboratory of Virology, Małopolska Centre of Biotechnology, Jagiellonian University; 2. II Department of Internal Medicine, Faculty of Medicine, Jagiellonian University Medical College; 3. Narodowy Instytut Zdrowia Publicznego – Państwowy Zakład Higieny (NIZP-PZH).	2020-03-13	Katarzyna Pancer et al
Poland/PL_P33/2020	EPI_ISL_455446	1. ViroGenetics - BSL3 Laboratory of Virology, Małopolska Centre of Biotechnology, Jagiellonian University; 2. II Department of Internal Medicine, Faculty of Medicine, Jagiellonian University Medical College; 3. Narodowy Instytut Zdrowia Publicznego – Państwowy Zakład Higieny (NIZP-PZH)	1. ViroGenetics - BSL3 Laboratory of Virology, Małopolska Centre of Biotechnology, Jagiellonian University; 2. II Department of Internal Medicine, Faculty of Medicine, Jagiellonian University Medical College; 3. Narodowy Instytut Zdrowia Publicznego – Państwowy Zakład Higieny (NIZP-PZH).	2020-03-13	Katarzyna Pancer et al
Poland/PL_P35/2020	EPI_ISL_455448	1. ViroGenetics - BSL3 Laboratory of Virology, Małopolska Centre of Biotechnology, Jagiellonian University; 2. II Department of Internal Medicine, Faculty of Medicine, Jagiellonian University Medical College; 3. Narodowy Instytut Zdrowia Publicznego – Państwowy Zakład Higieny (NIZP-PZH)	1. ViroGenetics - BSL3 Laboratory of Virology, Małopolska Centre of Biotechnology, Jagiellonian University; 2. II Department of Internal Medicine, Faculty of Medicine, Jagiellonian University Medical College; 3. Narodowy Instytut Zdrowia Publicznego – Państwowy Zakład Higieny (NIZP-PZH).	2020-04-01	Katarzyna Pancer et al
Poland/PL_P36/2020	EPI_ISL_455449	1. ViroGenetics - BSL3 Laboratory of Virology, Małopolska Centre of Biotechnology, Jagiellonian University; 2. II Department of Internal Medicine, Faculty of Medicine, Jagiellonian University Medical College; 3. Narodowy Instytut Zdrowia Publicznego – Państwowy Zakład Higieny (NIZP-PZH)	1. ViroGenetics - BSL3 Laboratory of Virology, Małopolska Centre of Biotechnology, Jagiellonian University; 2. II Department of Internal Medicine, Faculty of Medicine, Jagiellonian University Medical College; 3. Narodowy Instytut Zdrowia Publicznego – Państwowy Zakład Higieny (NIZP-PZH).	2020-03-15	Katarzyna Pancer et al
Poland/PL_P37/2020	EPI_ISL_455450	1. ViroGenetics - BSL3 Laboratory of Virology, Małopolska Centre of Biotechnology, Jagiellonian University; 2. II Department of Internal Medicine, Faculty of Medicine, Jagiellonian University Medical College; 3. Narodowy Instytut Zdrowia Publicznego – Państwowy Zakład Higieny (NIZP-PZH)	1. ViroGenetics - BSL3 Laboratory of Virology, Małopolska Centre of Biotechnology, Jagiellonian University; 2. II Department of Internal Medicine, Faculty of Medicine, Jagiellonian University Medical College; 3. Narodowy Instytut Zdrowia Publicznego – Państwowy Zakład Higieny (NIZP-PZH).	2020-03-26	Katarzyna Pancer et al
Poland/PL_P38/2020	EPI_ISL_455451	1. ViroGenetics - BSL3 Laboratory of Virology, Małopolska Centre of Biotechnology, Jagiellonian University; 2. II Department of Internal Medicine, Faculty of Medicine, Jagiellonian University Medical College; 3. Narodowy Instytut Zdrowia Publicznego – Państwowy Zakład Higieny (NIZP-PZH)	1. ViroGenetics - BSL3 Laboratory of Virology, Małopolska Centre of Biotechnology, Jagiellonian University; 2. II Department of Internal Medicine, Faculty of Medicine, Jagiellonian University Medical College; 3. Narodowy Instytut Zdrowia Publicznego – Państwowy Zakład Higieny (NIZP-PZH).	2020-03-17	Katarzyna Pancer et al
Poland/PL_P40/2020	EPI_ISL_455453	1. ViroGenetics - BSL3 Laboratory of Virology, Małopolska Centre of Biotechnology, Jagiellonian University; 2. II Department of Internal Medicine, Faculty of Medicine, Jagiellonian University Medical College; 3. Narodowy Instytut Zdrowia Publicznego – Państwowy Zakład Higieny (NIZP-PZH)	1. ViroGenetics - BSL3 Laboratory of Virology, Małopolska Centre of Biotechnology, Jagiellonian University; 2. II Department of Internal Medicine, Faculty of Medicine, Jagiellonian University Medical College; 3. Narodowy Instytut Zdrowia Publicznego – Państwowy Zakład Higieny (NIZP-PZH).	2020-03-05	Katarzyna Pancer et al
Poland/PL_P5/2020	EPI_ISL_428928	ViroGenetics - BSL3 Laboratory of Virology; Human Genome Variation Research Group & Genomics Centre MCB; Bioinformatics Research Group; Wojewódzka Stacja Sanitarno-Epidemiologiczna w Krakowie	ViroGenetics - BSL3 Laboratory of Virology; Human Genome Variation Research Group & Genomics Centre MCB; Bioinformatics Research Group	2020-03-30	Wojciech Branicki et al
Poland/PL_P8/2020	EPI_ISL_428931	ViroGenetics - BSL3 Laboratory of Virology; Human Genome Variation Research Group & Genomics Centre MCB; Bioinformatics Research Group; Wojewódzka Stacja Sanitarno-Epidemiologiczna w Krakowie	ViroGenetics - BSL3 Laboratory of Virology; Human Genome Variation Research Group & Genomics Centre MCB; Bioinformatics Research Group	2020-03-28	Wojciech Branicki et al
Poland/PL_P9/2020	EPI_ISL_428932	ViroGenetics - BSL3 Laboratory of Virology; Human Genome Variation Research Group & Genomics Centre MCB; Bioinformatics Research Group; Wojewódzka Stacja Sanitarno-Epidemiologiczna w Krakowie	ViroGenetics - BSL3 Laboratory of Virology; Human Genome Variation Research Group & Genomics Centre MCB; Bioinformatics Research Group	2020-03-28	Wojciech Branicki et al
Poland/Pom1/2020	EPI_ISL_428209	Laboratory of Molecular Biology, Diagnostyka sp. z o.o.	Laboratory of Recombinant Vaccines	2020-04-06	Lukasz Rabalski et al
Poland/Pom10/2020	EPI_ISL_451651	Hematology Laboratory, Section of Molecular Diagnostics, University Clinical Centre, Medical University of Gdansk	Laboratory of Recombinant Vaccines	2020-04-27	Lukasz Rabalski et al
Poland/Pom11/2020	EPI_ISL_451652	Hematology Laboratory, Section of Molecular Diagnostics, University Clinical Centre, Medical University of Gdansk	Laboratory of Recombinant Vaccines	2020-04-30	Lukasz Rabalski et al
Poland/Pom12/2020	EPI_ISL_451653	Hematology Laboratory, Section of Molecular Diagnostics, University Clinical Centre, Medical University of Gdansk	Laboratory of Recombinant Vaccines	2020-05-04	Lukasz Rabalski et al
Poland/Pom13/2020	EPI_ISL_451654	Hematology Laboratory, Section of Molecular Diagnostics, University Clinical Centre, Medical University of Gdansk	Laboratory of Recombinant Vaccines	2020-05-04	Lukasz Rabalski et al
Poland/Pom2/2020	EPI_ISL_450746	Laboratory of Molecular Biology, Diagnostyka sp. z o.o.	Laboratory of Recombinant Vaccines	2020-05-08	Lukasz Rabalski et al
Poland/Pom3/2020	EPI_ISL_451644	Laboratory of Molecular Biology, Diagnostyka sp. z o.o.	Laboratory of Recombinant Vaccines	2020-05-02	Lukasz Rabalski et al
Poland/Pom4/2020	EPI_ISL_451645	Laboratory of Molecular Biology, Diagnostyka sp. z o.o.	Laboratory of Recombinant Vaccines	2020-05-02	Lukasz Rabalski et al
Poland/Pom5/2020	EPI_ISL_451646	Laboratory of Molecular Biology, Diagnostyka sp. z o.o.	Laboratory of Recombinant Vaccines	2020-04-22	Lukasz Rabalski et al
Poland/Pom6/2020	EPI_ISL_451647	Laboratory of Molecular Biology, Diagnostyka sp. z o.o.	Laboratory of Recombinant Vaccines	2020-04-23	Lukasz Rabalski et al
Poland/Pom7/2020	EPI_ISL_451648	Hematology Laboratory, Section of Molecular Diagnostics, University Clinical Centre, Medical University of Gdansk	Laboratory of Recombinant Vaccines	2020-04-26	Lukasz Rabalski et al
Poland/Pom8/2020	EPI_ISL_451649	Hematology Laboratory, Section of Molecular Diagnostics, University Clinical Centre, Medical University of Gdansk	Laboratory of Recombinant Vaccines	2020-04-27	Lukasz Rabalski et al
Poland/Pom9/2020	EPI_ISL_451650	Hematology Laboratory, Section of Molecular Diagnostics, University Clinical Centre, Medical University of Gdansk	Laboratory of Recombinant Vaccines	2020-04-27	Lukasz Rabalski et al
Poland/Sla1/2020	EPI_ISL_451655	State Sanitary Inspectorate	Laboratory of Recombinant Vaccines	2020-05-05	Lukasz Rabalski et al
Poland/Sla10/2020	EPI_ISL_451664	State Sanitary Inspectorate	Laboratory of Recombinant Vaccines	2020-05-05	Lukasz Rabalski et al
Poland/Sla2/2020	EPI_ISL_451656	State Sanitary Inspectorate	Laboratory of Recombinant Vaccines	2020-05-05	Lukasz Rabalski et al
Poland/Sla3/2020	EPI_ISL_451657	State Sanitary Inspectorate	Laboratory of Recombinant Vaccines	2020-05-05	Lukasz Rabalski et al
Poland/Sla4/2020	EPI_ISL_451658	State Sanitary Inspectorate	Laboratory of Recombinant Vaccines	2020-05-05	Lukasz Rabalski et al
Poland/Sla5/2020	EPI_ISL_451659	State Sanitary Inspectorate	Laboratory of Recombinant Vaccines	2020-05-05	Lukasz Rabalski et al
Poland/Sla6/2020	EPI_ISL_451660	State Sanitary Inspectorate	Laboratory of Recombinant Vaccines	2020-05-05	Lukasz Rabalski et al
Poland/Sla7/2020	EPI_ISL_451661	State Sanitary Inspectorate	Laboratory of Recombinant Vaccines	2020-05-05	Lukasz Rabalski et al
Poland/Sla8/2020	EPI_ISL_451662	State Sanitary Inspectorate	Laboratory of Recombinant Vaccines	2020-05-05	Lukasz Rabalski et al
Poland/Sla9/2020	EPI_ISL_451663	State Sanitary Inspectorate	Laboratory of Recombinant Vaccines	2020-05-05	Lukasz Rabalski et al
Poland/Wro-02/2020	EPI_ISL_437625	Laboratory of Genomics & Bioinformatics, Institute of Immunology and Experimental Therapy, Polish Academy of Sciences Oddzial Mikrobiologii Wojewodzkiej Stacji Sanitarno-Epidemiologicznej.	Laboratory of Genomics & Bioinformatics, Institute of Immunology and Experimental Therapy, Polish Academy of Sciences	2020-03-18	Dorota Kujawa et al
Portugal/PT0001b/2020	EPI_ISL_417986	Centro Hospitalar e Universitario de Sao Joao, Porto	Instituto Nacional de Saude (INSA)	2020-03-03	Guiomar et al et al
Portugal/PT0019/2020	EPI_ISL_418004	ARS Algarve - Laboratório Laura Ayres	Instituto Nacional de Saude (INSA)	2020-03-12	Guiomar et al et al
Portugal/PT0049/2020	EPI_ISL_421452	Instituto Nacional de Saude (INSA)	Instituto Nacional de Saude (INSA)	2020-03-18	Guiomar et al et al
Portugal/PT0050/2020	EPI_ISL_421453	CHTMAD	Instituto Nacional de Saude (INSA)	2020-03-18	Guiomar et al et al
Portugal/PT0051/2020	EPI_ISL_421454	H Beatriz Angelo	Instituto Nacional de Saude (INSA)	2020-03-18	Guiomar et al et al
Portugal/PT0064/2020	EPI_ISL_421467	H Evora	Instituto Nacional de Saude (INSA)	2020-03-20	Guiomar et al et al
Portugal/PT0080/2020	EPI_ISL_421483	CH VN Gaia - Espinho	Instituto Nacional de Saude (INSA)	2020-03-18	Guiomar et al et al
Portugal/PT0082/2020	EPI_ISL_421485	CH Barreiro Montijo	Instituto Nacional de Saude (INSA)	2020-03-22	Guiomar et al et al
Portugal/PT0092/2020	EPI_ISL_421495	H Santarem	Instituto Nacional de Saude (INSA)	2020-03-23	Guiomar et al et al
Portugal/PT0097/2020	EPI_ISL_453813		Instituto Nacional de Saude (INSA)	2020-03-24	Borges et al et al
Portugal/PT0114/2020	EPI_ISL_453830		Instituto Nacional de Saude (INSA)	2020-03-25	Borges et al et al
Portugal/PT0118/2020	EPI_ISL_453834		Instituto Nacional de Saude (INSA)	2020-03-25	Borges et al et al
Portugal/PT0139/2020	EPI_ISL_453855		Instituto Nacional de Saude (INSA)	2020-03-27	Borges et al et al
Portugal/PT0220/2020	EPI_ISL_453934		Instituto Nacional de Saude (INSA)	2020-03-30	Borges et al et al
Portugal/PT0233/2020	EPI_ISL_453947		Instituto Nacional de Saude (INSA)	2020-03-31	Borges et al et al

Portugal/PT0234/2020	EPI_ISL_453948		Instituto Nacional de Saude (INSA)	2020-03-31	Borges et al et al
Portugal/PT0237/2020	EPI_ISL_453951		Instituto Nacional de Saude (INSA)	2020-03-31	Borges et al et al
Portugal/PT0245/2020	EPI_ISL_453959		Instituto Nacional de Saude (INSA)	2020-04-01	Borges et al et al
Portugal/PT0246/2020	EPI_ISL_453960		Instituto Nacional de Saude (INSA)	2020-04-02	Borges et al et al
Portugal/PT0249/2020	EPI_ISL_453963		Instituto Nacional de Saude (INSA)	2020-04-02	Borges et al et al
Portugal/PT0251/2020	EPI_ISL_453965		Instituto Nacional de Saude (INSA)	2020-04-02	Borges et al et al
Portugal/PT0255/2020	EPI_ISL_453969		Instituto Nacional de Saude (INSA)	2020-04-03	Borges et al et al
Portugal/PT0261/2020	EPI_ISL_453975		Instituto Nacional de Saude (INSA)	2020-04-03	Borges et al et al
Portugal/PT0265/2020	EPI_ISL_453979		Instituto Nacional de Saude (INSA)	2020-04-04	Borges et al et al
Portugal/PT0267/2020	EPI_ISL_453981		Instituto Nacional de Saude (INSA)	2020-04-04	Borges et al et al
Portugal/PT0270/2020	EPI_ISL_453984		Instituto Nacional de Saude (INSA)	2020-04-05	Borges et al et al
Portugal/PT0272/2020	EPI_ISL_453986		Instituto Nacional de Saude (INSA)	2020-04-05	Borges et al et al
Portugal/PT0275/2020	EPI_ISL_453989		Instituto Nacional de Saude (INSA)	2020-04-05	Borges et al et al
Portugal/PT0277/2020	EPI_ISL_453991		Instituto Nacional de Saude (INSA)	2020-04-05	Borges et al et al
Portugal/PT0283a/2020	EPI_ISL_453997		Instituto Nacional de Saude (INSA)	2020-04-16	Borges et al et al
Portugal/PT0301/2020	EPI_ISL_454017		Instituto Nacional de Saude (INSA)	2020-03-14	Borges et al et al
Portugal/PT0322/2020	EPI_ISL_454038		Instituto Nacional de Saude (INSA)	2020-03-16	Borges et al et al
Portugal/PT0333/2020	EPI_ISL_454049		Instituto Nacional de Saude (INSA)	2020-03-18	Borges et al et al
Portugal/PT0337/2020	EPI_ISL_454053		Instituto Nacional de Saude (INSA)	2020-03-18	Borges et al et al
Portugal/PT0356/2020	EPI_ISL_454072		Instituto Nacional de Saude (INSA)	2020-03-19	Borges et al et al
Portugal/PT0372/2020	EPI_ISL_454088		Instituto Nacional de Saude (INSA)	2020-03-20	Borges et al et al
Portugal/PT0389c/2020	EPI_ISL_454107		Instituto Nacional de Saude (INSA)	2020-04-23	Borges et al et al
Portugal/PT0430/2020	EPI_ISL_454154		Instituto Nacional de Saude (INSA)	2020-03-31	Borges et al et al
Portugal/PT0440/2020	EPI_ISL_454164		Instituto Nacional de Saude (INSA)	2020-03-23	Borges et al et al
Portugal/PT0459/2020	EPI_ISL_454183		Instituto Nacional de Saude (INSA)	2020-04-17	Borges et al et al
Portugal/PT0465/2020	EPI_ISL_454189		Instituto Nacional de Saude (INSA)	2020-04-18	Borges et al et al
Portugal/PT0467/2020	EPI_ISL_454191		Instituto Nacional de Saude (INSA)	2020-04-18	Borges et al et al
Portugal/PT0474/2020	EPI_ISL_454198		Instituto Nacional de Saude (INSA)	2020-04-24	Borges et al et al
Portugal/PT0476/2020	EPI_ISL_454200		Instituto Nacional de Saude (INSA)	2020-04-24	Borges et al et al
Portugal/PT0487/2020	EPI_ISL_454211		Instituto Nacional de Saude (INSA)	2020-04-01	Borges et al et al
Portugal/PT0495/2020	EPI_ISL_454219		Instituto Nacional de Saude (INSA)	2020-04-30	Borges et al et al
Portugal/PT0497/2020	EPI_ISL_454221		Instituto Nacional de Saude (INSA)	2020-05-01	Borges et al et al
Portugal/PT0500/2020	EPI_ISL_454224		Instituto Nacional de Saude (INSA)	2020-03-14	Borges et al et al
Portugal/PT0508/2020	EPI_ISL_454232		Instituto Nacional de Saude (INSA)	2020-03-19	Borges et al et al
Portugal/PT0531/2020	EPI_ISL_454255		Instituto Nacional de Saude (INSA)	2020-04-07	Borges et al et al
Portugal/PT0536/2020	EPI_ISL_454260		Instituto Nacional de Saude (INSA)	2020-04-07	Borges et al et al
Portugal/PT0548/2020	EPI_ISL_454272		Instituto Nacional de Saude (INSA)	2020-05-02	Borges et al et al
Portugal/PT0552/2020	EPI_ISL_454275		Instituto Nacional de Saude (INSA)	2020-04-07	Borges et al et al
Portugal/PT0561/2020	EPI_ISL_454284		Instituto Nacional de Saude (INSA)	2020-04-27	Borges et al et al
Portugal/PT0563/2020	EPI_ISL_454286		Instituto Nacional de Saude (INSA)	2020-05-02	Borges et al et al
Portugal/PT0575/2020	EPI_ISL_454298		Instituto Nacional de Saude (INSA)	2020-04-11	Borges et al et al
Portugal/PT0593/2020	EPI_ISL_454316		Instituto Nacional de Saude (INSA)	2020-05-04	Borges et al et al
Portugal/PT0594/2020	EPI_ISL_454317		Instituto Nacional de Saude (INSA)	2020-04-03	Borges et al et al
Portugal/PT0601/2020	EPI_ISL_454324		Instituto Nacional de Saude (INSA)	2020-04-06	Borges et al et al
Portugal/PT0605/2020	EPI_ISL_454328		Instituto Nacional de Saude (INSA)	2020-03-14	Borges et al et al
Portugal/PT0628/2020	EPI_ISL_454351		Instituto Nacional de Saude (INSA)	2020-04-06	Borges et al et al
Romania/278406/2020	EPI_ISL_445220	Laborarory for Respiratory Viruses, "Cantacuzino" National Military-Medical Institute for Resararch and Development	Cantacuzino Institute	2020-04-30	M.Lazar et al
Romania/279067/2020	EPI_ISL_445243	Laboratory for Respiratory Viruses, Cantacuzino National Military-Medical Institute for Research and Development	Cantacuzino Institute	2020-04-30	M.Lazar et al
Romania/279068/2020	EPI_ISL_447054	Cantacuzino National Military-Medical Institute for Research and Development	Cantacuzino Institute	2020-04-30	M.Lazar et al
Romania/283584/2020	EPI_ISL_455469	Laboratory for Respiratory Viruses, Cantacuzino National Military-Medical Institute for Research and Development	Cantacuzino Institute	2020-05-12	M.Lazar et al
Romania/284056/2020	EPI_ISL_455473	Laboratory for Respiratory Viruses, Cantacuzino National Military-Medical Institute for Research and Development	Cantacuzino Institute	2020-05-14	M.Lazar et al
Romania/284168/2020	EPI_ISL_455472	Laboratory for Respiratory Viruses, Cantacuzino National Military-Medical Institute for Research and Development	Cantacuzino Institute	2020-05-13	M.Lazar et al
Romania/284213/2020	EPI_ISL_455468	Laboratory for Respiratory Viruses, Cantacuzino National Military-Medical Institute for Research and Development	Cantacuzino Institute	2020-05-13	M.Lazar et al
Romania/284371/2020	EPI_ISL_455477	Laboratory for Respiratory Viruses, Cantacuzino National Military-Medical Institute for Research and Development	Cantacuzino Institute	2020-05-13	M.Lazar et al
Romania/284468/2020	EPI_ISL_455479	Laboratory for Respiratory Viruses, Cantacuzino National Military-Medical Institute for Research and Development	Cantacuzino Institute	2020-05-14	M.Lazar et al
Romania/284489/2020	EPI_ISL_455470	Laboratory for Respiratory Viruses, Cantacuzino National Military-Medical Institute for Research and Development	Cantacuzino Institute	2020-05-14	M.Lazar et al
Romania/284508/2020	EPI_ISL_455471	Laboratory for Respiratory Viruses, Cantacuzino National Military-Medical Institute for Research and Development	Cantacuzino Institute	2020-05-14	M.Lazar et al
Romania/284762/2020	EPI_ISL_455475	Laboratory for Respiratory Viruses, Cantacuzino National Military-Medical Institute for Research and Development	Cantacuzino Institute	2020-05-14	M.Lazar et al
Romania/284783/2020	EPI_ISL_455474	Laboratory for Respiratory Viruses, Cantacuzino National Military-Medical Institute for Research and Development	Cantacuzino Institute	2020-05-14	M.Lazar et al
Romania/285388/2020	EPI_ISL_455476	Laboratory for Respiratory Viruses, Cantacuzino National Military-Medical Institute for Research and Development	Cantacuzino Institute	2020-05-17	M.Lazar et al
Romania/ChVir7246/2020	EPI_ISL_467780	National Influenza Centre Romania	Charite Universitatsmedizin Berlin, Institute of Virology	2020-04-26	Victor M Corman et al A
Romania/ChVir7248/2020	EPI_ISL_467781	National Influenza Centre Romania	Charite Universitatsmedizin Berlin, Institute of Virology	2020-04-28	Victor M Corman et al A
Russia/Buryatia-84506/2020	EPI_ISL_428894	State Research Center of Virology and Biotechnology VECTOR, Department of Collection of Microorganisms	State Research Center of Virology and Biotechnology VECTOR, Department of Collection of Microorganisms	2020-03-27	Oleg V. Pyankov et al
Russia/Buryatia-86204/2020	EPI_ISL_428899	State Research Center of Virology and Biotechnology VECTOR, Department of Collection of Microorganisms	State Research Center of Virology and Biotechnology VECTOR, Department of Collection of Microorganisms	2020-03-29	Sergey A. Bodnev et al
Russia/Buryatia-87106/2020	EPI_ISL_428919	State Research Center of Virology and Biotechnology VECTOR, Department of Collection of Microorganisms	State Research Center of Virology and Biotechnology VECTOR, Department of Collection of Microorganisms	2020-03-30	Sergey A. Bodnev et al
Russia/Chechenya-83801/2020	EPI_ISL_428909	State Research Center of Virology and Biotechnology VECTOR, Department of Collection of Microorganisms	State Research Center of Virology and Biotechnology VECTOR, Department of Collection of Microorganisms	2020-03-26	Oleg V. Pyankov et al
Russia/CRIE130014/2020	EPI_ISL_470539	Molecular diagnostic laboratory of Federal Budget Institution of Science "Central Research Institute of Epidemiology" of The Federal Service on Customers' Rights Protection and Human Well-being Surveillance	Group of Genomics and Postgenomic Technologies of Central Research Institute of Epidemilology	2020-03-27	Speranskaya AS et al
Russia/CRIE140762/2020	EPI_ISL_460605	Molecular diagnostic laboratory of Federal Budget Institution of Science "Central Research Institute of Epidemiology" of The Federal Service on Customers' Rights Protection and Human Well-being Surveillance	Group of Genomics and Postgenomic Technologies of Central Research Institute of Epidemilology	2020-03-29	Speranskaya AS et al
Russia/CRIE160583/2020	EPI_ISL_462149	Molecular diagnostic laboratory of Federal Budget Institution of Science "Central Research Institute of Epidemiology" of The Federal Service on Customers' Rights Protection and Human Well-being Surveillance	Group of Genomics and Postgenomic Technologies of Central Research Institute of Epidemilology	2020-03-30	Speranskaya AS et al
Russia/CRIE162784/2020	EPI_ISL_462150	Molecular diagnostic laboratory of Federal Budget Institution of Science "Central Research Institute of Epidemiology" of The Federal Service on Customers' Rights Protection and Human Well-being Surveillance	Group of Genomics and Postgenomic Technologies of Central Research Institute of Epidemilology	2020-03-31	Speranskaya AS et al
Russia/Kabardino-Balkaria-80503/2020	EPI_ISL_428898	State Research Center of Virology and Biotechnology VECTOR, Department of Collection of Microorganisms	State Research Center of Virology and Biotechnology VECTOR, Department of Collection of Microorganisms	2020-03-23	Sergey A. Bodnev et al
Russia/Lipetsk-62704/2020	EPI_ISL_428865	State Research Center of Virology and Biotechnology VECTOR, Department of Collection of Microorganisms	State Research Center of Virology and Biotechnology VECTOR, Department of Collection of Microorganisms	2020-03-11	Oleg V. Pyankov et al
Russia/Moscow_PMVL-1/2020	EPI_ISL_421275	Russian State Collection of Viruses	Pathogenic Microorganisms Variability Laboratory	2020-03-18	Alexey Shchetinin et al
Russia/Moscow_PMVL-3/2020	EPI_ISL_470897	Pathogenic Microorganisms Variability Laboratory	Pathogenic Microorganisms Variability Laboratory	2020-05-05	Alexey Shchetinin et al
Russia/Moscow_PMVL-5/2020	EPI_ISL_470899	Pathogenic Microorganisms Variability Laboratory	Pathogenic Microorganisms Variability Laboratory	2020-05-05	Alexey Shchetinin et al
Russia/Moscow_PMVL-8/2020	EPI_ISL_470901	Influenza etiology and epidemiology laboratory	Pathogenic Microorganisms Variability Laboratory	2020-04-02	Alexey Shchetinin et al
Russia/Moscow_PMVL-9/2020	EPI_ISL_470902	Influenza etiology and epidemiology laboratory	Pathogenic Microorganisms Variability Laboratory	2020-04-08	Alexey Shchetinin et al
Russia/Moscow-35I/2020	EPI_ISL_428852	FSBSI "Chumakov Federal Scientific Center for Research and Development of Immune-and-Biological Products of Russian Academy of Sciences"	FSBSI "Chumakov Federal Scientific Center for Research and Development of Immune-and-Biological Products of Russian Academy of Sciences" & NRC "Kurchatov institute"	2020-04-02	Liubov Kozlovskaya et al
Russia/Moscow-62501/2020	EPI_ISL_428861	State Research Center of Virology and Biotechnology VECTOR, Department of Collection of Microorganisms	State Research Center of Virology and Biotechnology VECTOR, Department of Collection of Microorganisms	2020-03-11	Oleg V. Pyankov et al
Russia/Moscow-67117/2020	EPI_ISL_428875	State Research Center of Virology and Biotechnology VECTOR, Department of Collection of Microorganisms	State Research Center of Virology and Biotechnology VECTOR, Department of Collection of Microorganisms	2020-03-16	Sergey A. Bodnev et al
Russia/Moscow-71602/2020	EPI_ISL_428877	State Research Center of Virology and Biotechnology VECTOR, Department of Collection of Microorganisms	State Research Center of Virology and Biotechnology VECTOR, Department of Collection of Microorganisms	2020-03-19	Sergey A. Bodnev et al
Russia/Moscow-77610/2020	EPI_ISL_428887	State Research Center of Virology and Biotechnology VECTOR, Department of Collection of Microorganisms	State Research Center of Virology and Biotechnology VECTOR, Department of Collection of Microorganisms	2020-03-22	Oleg V. Pyankov et al
Russia/Moscow-77627/2020	EPI_ISL_428884	State Research Center of Virology and Biotechnology VECTOR, Department of Collection of Microorganisms	State Research Center of Virology and Biotechnology VECTOR, Department of Collection of Microorganisms	2020-03-22	Oleg V. Pyankov et al
Russia/Moscow-80402/2020	EPI_ISL_428905	State Research Center of Virology and Biotechnology VECTOR, Department of Collection of Microorganisms	State Research Center of Virology and Biotechnology VECTOR, Department of Collection of Microorganisms	2020-03-23	Sergey A. Bodnev et al
Russia/Moscow-GCBL3/2020	EPI_ISL_436717	Genomics and Computational Biology Lab, Scientific Research Institute of Physical-Chemical Medicine, FMBA of Russia	Genomics and Computational Biology Lab, Scientific Research Institute of Physical-Chemical Medicine, FMBA of Russia	2020-04-14	A. Pavlenko et al
Russia/Pskov-83602/2020	EPI_ISL_428908	State Research Center of Virology and Biotechnology VECTOR, Department of Collection of Microorganisms	State Research Center of Virology and Biotechnology VECTOR, Department of Collection of Microorganisms	2020-03-25	Oleg V. Pyankov et al
Russia/SCPM-O-01/2020	EPI_ISL_451963	Federal Budget Institution of Science, State Research Center for Applied Microbiology & Biotechnology	Federal Budget Institution of Science, State Research Center for Applied Microbiology & Biotechnology	2020-03-20	Dyatlov I et al
Russia/SCPM-O-03/2020	EPI_ISL_451965	Federal Budget Institution of Science, State Research Center for Applied Microbiology & Biotechnology	Federal Budget Institution of Science, State Research Center for Applied Microbiology & Biotechnology	2020-03-20	Dyatlov I et al
Russia/StPetersburg-64304/2020	EPI_ISL_428868	State Research Center of Virology and Biotechnology VECTOR, Department of Collection of Microorganisms	State Research Center of Virology and Biotechnology VECTOR, Department of Collection of Microorganisms	2020-03-13	Oleg V. Pyankov et al
Russia/StPetersburg-73603/2020	EPI_ISL_428883	State Research Center of Virology and Biotechnology VECTOR, Department of Collection of Microorganisms	State Research Center of Virology and Biotechnology VECTOR, Department of Collection of Microorganisms	2020-03-20	Oleg V. Pyankov et al
Russia/StPetersburg-RII4144S/2020	EPI_ISL_427307	WHO National Influenza Centre Russian Federation	WHO National Influenza Centre Russian Federation	2020-03-20	Andrey Komissarov et al
Russia/StPetersburg-RII4326S/2020	EPI_ISL_427337	WHO National Influenza Centre Russian Federation	WHO National Influenza Centre Russian Federation	2020-03-25	Andrey Komissarov et al
Russia/StPetersburg-RII4328S/2020	EPI_ISL_427338	WHO National Influenza Centre Russian Federation	WHO National Influenza Centre Russian Federation	2020-03-25	Andrey Komissarov et al
Russia/StPetersburg-RII4693S/2020	EPI_ISL_427325	WHO National Influenza Centre Russian Federation	WHO National Influenza Centre Russian Federation	2020-04-07	Andrey Komissarov et al
Russia/StPetersburg-RII4697S/2020	EPI_ISL_427328	WHO National Influenza Centre Russian Federation	WHO National Influenza Centre Russian Federation	2020-04-07	Andrey Komissarov et al
Russia/StPetersburg-RII4707S/2020	EPI_ISL_427329	WHO National Influenza Centre Russian Federation	WHO National Influenza Centre Russian Federation	2020-04-07	Andrey Komissarov et al
Russia/StPetersburg-RII4712V/2020	EPI_ISL_430068	WHO National Influenza Centre Russian Federation	WHO National Influenza Centre Russian Federation	2020-04-07	Andrey Komissarov et al
Russia/StPetersburg-RII4724S/2020	EPI_ISL_427333	WHO National Influenza Centre Russian Federation	WHO National Influenza Centre Russian Federation	2020-04-07	Andrey Komissarov et al
Russia/StPetersburg-RII4726S/2020	EPI_ISL_427334	WHO National Influenza Centre Russian Federation	WHO National Influenza Centre Russian Federation	2020-04-07	Andrey Komissarov et al
Russia/StPetersburg-RII4917S/2020	EPI_ISL_430070	WHO National Influenza Centre Russian Federation	WHO National Influenza Centre Russian Federation	2020-04-07	Andrey Komissarov et al
Russia/StPetersburg-RII4983S/2020	EPI_ISL_430080	WHO National Influenza Centre Russian Federation	WHO National Influenza Centre Russian Federation	2020-04-10	Andrey Komissarov et al
Russia/StPetersburg-RII5169S/2020	EPI_ISL_430088	WHO National Influenza Centre Russian Federation	WHO National Influenza Centre Russian Federation	2020-04-09	Andrey Komissarov et al
Russia/StPetersburg-RII5244S/2020	EPI_ISL_430090	WHO National Influenza Centre Russian Federation	WHO National Influenza Centre Russian Federation	2020-04-10	Andrey Komissarov et al
Russia/StPetersburg-RII5634S/2020	EPI_ISL_430093	WHO National Influenza Centre Russian Federation	WHO National Influenza Centre Russian Federation	2020-04-14	Andrey Komissarov et al
Russia/StPetersburg-RII5635S/2020	EPI_ISL_430094	WHO National Influenza Centre Russian Federation	WHO National Influenza Centre Russian Federation	2020-04-14	Andrey Komissarov et al

Russia/StPetersburg-RII5942S/2020	EPI_ISL_430099	WHO National Influenza Centre Russian Federation	WHO National Influenza Centre Russian Federation	2020-04-13	Andrey Komissarov et al
Russia/StPetersburg-RII6027S/2020	EPI_ISL_430100	WHO National Influenza Centre Russian Federation	WHO National Influenza Centre Russian Federation	2020-04-14	Andrey Komissarov et al
Russia/StPetersburg-RII6061S/2020	EPI_ISL_430108	WHO National Influenza Centre Russian Federation	WHO National Influenza Centre Russian Federation	2020-04-15	Andrey Komissarov et al
Russia/StPetersburg-RII6086S/2020	EPI_ISL_430111	WHO National Influenza Centre Russian Federation	WHO National Influenza Centre Russian Federation	2020-04-15	Andrey Komissarov et al
Russia/StPetersburg-RII7488S/2020	EPI_ISL_450253	WHO National Influenza Centre Russian Federation	WHO National Influenza Centre Russian Federation	2020-04-20	Andrey Komissarov et al
Russia/StPetersburg-RII7545S/2020	EPI_ISL_450257	WHO National Influenza Centre Russian Federation	WHO National Influenza Centre Russian Federation	2020-04-20	Andrey Komissarov et al
Russia/StPetersburg-RII7581S/2020	EPI_ISL_450261	WHO National Influenza Centre Russian Federation	WHO National Influenza Centre Russian Federation	2020-04-21	Andrey Komissarov et al
Russia/StPetersburg-RII7656S/2020	EPI_ISL_450264	WHO National Influenza Centre Russian Federation	WHO National Influenza Centre Russian Federation	2020-04-20	Andrey Komissarov et al
Russia/StPetersburg-RII7880S/2020	EPI_ISL_450266	WHO National Influenza Centre Russian Federation	WHO National Influenza Centre Russian Federation	2020-04-22	Andrey Komissarov et al
Russia/StPetersburg-RII8317S/2020	EPI_ISL_450271	WHO National Influenza Centre Russian Federation	WHO National Influenza Centre Russian Federation	2020-04-21	Andrey Komissarov et al
Russia/StPetersburg-RII8371S/2020	EPI_ISL_450274	WHO National Influenza Centre Russian Federation	WHO National Influenza Centre Russian Federation	2020-04-22	Andrey Komissarov et al
Russia/StPetersburg-RII8418S/2020	EPI_ISL_450284	WHO National Influenza Centre Russian Federation	WHO National Influenza Centre Russian Federation	2020-04-22	Andrey Komissarov et al
Russia/StPetersburg-RII8428S/2020	EPI_ISL_450286	WHO National Influenza Centre Russian Federation	WHO National Influenza Centre Russian Federation	2020-04-22	Andrey Komissarov et al
Russia/StPetersburg-RII8890S/2020	EPI_ISL_450289	WHO National Influenza Centre Russian Federation	WHO National Influenza Centre Russian Federation	2020-04-23	Andrey Komissarov et al
Russia/Sverdlovsk-71903/2020	EPI_ISL_428890	State Research Center of Virology and Biotechnology VECTOR, Department of Collection of Microorganisms	State Research Center of Virology and Biotechnology VECTOR, Department of Collection of Microorganisms	2020-03-19	Oleg V. Pyankov et al
Russia/Sverdlovsk-86704/2020	EPI_ISL_428922	State Research Center of Virology and Biotechnology VECTOR, Department of Collection of Microorganisms	State Research Center of Virology and Biotechnology VECTOR, Department of Collection of Microorganisms	2020-03-30	Sergey A. Bodnev et al
Russia/Tver-72601/2020	EPI_ISL_428921	State Research Center of Virology and Biotechnology VECTOR, Department of Collection of Microorganisms	State Research Center of Virology and Biotechnology VECTOR, Department of Collection of Microorganisms	2020-03-19	Sergey A. Bodnev et al
Russia/Ulan-Ude-RII4562V/2020	EPI_ISL_430112	WHO National Influenza Centre Russian Federation	WHO National Influenza Centre Russian Federation	2020-03-25	Andrey Komissarov et al
Russia/Yakutia-73703/2020	EPI_ISL_428888	State Research Center of Virology and Biotechnology VECTOR, Department of Collection of Microorganisms	State Research Center of Virology and Biotechnology VECTOR, Department of Collection of Microorganisms	2020-03-20	Oleg V. Pyankov et al
SaudiArabia/42952/2020	EPI_ISL_469242	Special Infectious Agents Unit	Special Infectious Agents Unit	2020-03-15	Azhar et al
SaudiArabia/4637/2020	EPI_ISL_469241	Special Infectious Agents Unit	Special Infectious Agents Unit	2020-03-15	Azhar et al
SaudiArabia/85613/2020	EPI_ISL_469243	Special Infectious Agents Unit	Special Infectious Agents Unit	2020-03-15	Azhar et al
SaudiArabia/85790C/2020	EPI_ISL_469251	Special Infectious Agents Unit	Special Infectious Agents Unit	2020-03-15	Azhar et al
SaudiArabia/85791/2020	EPI_ISL_469246	Special Infectious Agents Unit	Special Infectious Agents Unit	2020-03-15	Azhar et al
SaudiArabia/86327/2020	EPI_ISL_469248	Special Infectious Agents Unit	Special Infectious Agents Unit	2020-03-15	Azhar et al
SaudiArabia/86650/2020	EPI_ISL_469249	Special Infectious Agents Unit	Special Infectious Agents Unit	2020-03-15	Azhar et al
SaudiArabia/KAIMRC-Alghoribi/2020	EPI_ISL_416432	Clinical Microbiology Lab	Infectious Disease Research Department, King Abdullah International Medical Research Center (KAIMRC)	2020-03-07	Majed Alghoribi et al
SaudiArabia/KAUST-Jeddah59/2020	EPI_ISL_437496	Pathogen Genomics Lab King Abdullah University of Science and Technology(KAUST)	Pathogen Genomics Lab King Abdullah University of Science and Technology(KAUST)	2020-04-01	Sara Mfarrej et al
SaudiArabia/KAUST-Jeddah61/2020	EPI_ISL_437754	Pathogen Genomics Lab King Abdullah University of Science and Technology(KAUST)	Pathogen Genomics Lab King Abdullah University of Science and Technology(KAUST)	2020-04-01	Sharif Hala et al
SaudiArabia/KAUST-Jeddah64/2020	EPI_ISL_437475	Pathogen Genomics Lab King Abdullah University of Science and Technology(KAUST)	Pathogen Genomics Lab King Abdullah University of Science and Technology(KAUST)	2020-04-01	Sharif Hala et al
SaudiArabia/KAUST-Jeddah73/2020	EPI_ISL_437758	Pathogen Genomics Lab King Abdullah University of Science and Technology(KAUST)	Pathogen Genomics Lab King Abdullah University of Science and Technology(KAUST)	2020-04-01	Sharif Hala et al
SaudiArabia/KAUST-Jeddah75/2020	EPI_ISL_437759	Pathogen Genomics Lab King Abdullah University of Science and Technology(KAUST)	Pathogen Genomics Lab King Abdullah University of Science and Technology(KAUST)	2020-04-01	Sharif Hala et al
SaudiArabia/KAUST-Jeddah76/2020	EPI_ISL_437760	Pathogen Genomics Lab King Abdullah University of Science and Technology(KAUST)	Pathogen Genomics Lab King Abdullah University of Science and Technology(KAUST)	2020-04-01	Sharif Hala et al
SaudiArabia/KAUST-Madinah208/2020	EPI_ISL_437715	Pathogen Genomics Lab King Abdullah University of Science and Technology(KAUST)	Pathogen Genomics Lab King Abdullah University of Science and Technology(KAUST)	2020-04-14	Sharif Hala et al
SaudiArabia/KAUST-Madinah23/2020	EPI_ISL_437482	Pathogen Genomics Lab King Abdullah University of Science and Technology(KAUST)	Pathogen Genomics Lab King Abdullah University of Science and Technology(KAUST)	2020-03-29	Sara Mfarrej et al
SaudiArabia/KAUST-Madinah24/2020	EPI_ISL_437483	Pathogen Genomics Lab King Abdullah University of Science and Technology(KAUST)	Pathogen Genomics Lab King Abdullah University of Science and Technology(KAUST)	2020-03-29	Sara Mfarrej et al
SaudiArabia/KAUST-Madinah248/2020	EPI_ISL_437734	Pathogen Genomics Lab King Abdullah University of Science and Technology(KAUST)	Pathogen Genomics Lab King Abdullah University of Science and Technology(KAUST)	2020-04-20	Sharif Hala et al
SaudiArabia/KAUST-Madinah249/2020	EPI_ISL_437735	Pathogen Genomics Lab King Abdullah University of Science and Technology(KAUST)	Pathogen Genomics Lab King Abdullah University of Science and Technology(KAUST)	2020-04-20	Sharif Hala et al
SaudiArabia/KAUST-Madinah25/2020	EPI_ISL_437469	Pathogen Genomics Lab King Abdullah University of Science and Technology(KAUST)	Pathogen Genomics Lab King Abdullah University of Science and Technology(KAUST)	2020-03-29	Sharif Hala et al
SaudiArabia/KAUST-Madinah252/2020	EPI_ISL_437736	Pathogen Genomics Lab King Abdullah University of Science and Technology(KAUST)	Pathogen Genomics Lab King Abdullah University of Science and Technology(KAUST)	2020-04-20	Sharif Hala et al
SaudiArabia/KAUST-Madinah257/2020	EPI_ISL_437741	Pathogen Genomics Lab King Abdullah University of Science and Technology(KAUST)	Pathogen Genomics Lab King Abdullah University of Science and Technology(KAUST)	2020-04-20	Sharif Hala et al
SaudiArabia/KAUST-Madinah258/2020	EPI_ISL_437742	Pathogen Genomics Lab King Abdullah University of Science and Technology(KAUST)	Pathogen Genomics Lab King Abdullah University of Science and Technology(KAUST)	2020-04-20	Sharif Hala et al
SaudiArabia/KAUST-Madinah261/2020	EPI_ISL_437744	Pathogen Genomics Lab King Abdullah University of Science and Technology(KAUST)	Pathogen Genomics Lab King Abdullah University of Science and Technology(KAUST)	2020-04-20	Sharif Hala et al
SaudiArabia/KAUST-Madinah263/2020	EPI_ISL_437746	Pathogen Genomics Lab King Abdullah University of Science and Technology(KAUST)	Pathogen Genomics Lab King Abdullah University of Science and Technology(KAUST)	2020-04-20	Sharif Hala et al
SaudiArabia/KAUST-Madinah27/2020	EPI_ISL_437484	Pathogen Genomics Lab King Abdullah University of Science and Technology(KAUST)	Pathogen Genomics Lab King Abdullah University of Science and Technology(KAUST)	2020-03-29	Sara Mfarrej et al
SaudiArabia/KAUST-Madinah30/2020	EPI_ISL_437485	Pathogen Genomics Lab King Abdullah University of Science and Technology(KAUST)	Pathogen Genomics Lab King Abdullah University of Science and Technology(KAUST)	2020-03-29	Sara Mfarrej et al
SaudiArabia/KAUST-Madinah34/2020	EPI_ISL_437470	Pathogen Genomics Lab King Abdullah University of Science and Technology(KAUST)	Pathogen Genomics Lab King Abdullah University of Science and Technology(KAUST)	2020-03-29	Sharif Hala et al
SaudiArabia/KAUST-Madinah41/2020	EPI_ISL_437472	Pathogen Genomics Lab King Abdullah University of Science and Technology(KAUST)	Pathogen Genomics Lab King Abdullah University of Science and Technology(KAUST)	2020-03-29	Sharif Hala et al
SaudiArabia/KAUST-Madinah44/2020	EPI_ISL_437473	Pathogen Genomics Lab King Abdullah University of Science and Technology(KAUST)	Pathogen Genomics Lab King Abdullah University of Science and Technology(KAUST)	2020-03-29	Sharif Hala et al
SaudiArabia/KAUST-Madinah45/2020	EPI_ISL_437474	Pathogen Genomics Lab King Abdullah University of Science and Technology(KAUST)	Pathogen Genomics Lab King Abdullah University of Science and Technology(KAUST)	2020-03-29	Sharif Hala et al
SaudiArabia/KAUST-Madinah47/2020	EPI_ISL_437487	Pathogen Genomics Lab King Abdullah University of Science and Technology(KAUST)	Pathogen Genomics Lab King Abdullah University of Science and Technology(KAUST)	2020-03-29	Sara Mfarrej et al
SaudiArabia/KAUST-Madinah48/2020	EPI_ISL_437488	Pathogen Genomics Lab King Abdullah University of Science and Technology(KAUST)	Pathogen Genomics Lab King Abdullah University of Science and Technology(KAUST)	2020-03-29	Sara Mfarrej et al
SaudiArabia/KAUST-Madinah49/2020	EPI_ISL_437489	Pathogen Genomics Lab King Abdullah University of Science and Technology(KAUST)	Pathogen Genomics Lab King Abdullah University of Science and Technology(KAUST)	2020-03-29	Sara Mfarrej et al
SaudiArabia/KAUST-Madinah50/2020	EPI_ISL_437490	Pathogen Genomics Lab King Abdullah University of Science and Technology(KAUST)	Pathogen Genomics Lab King Abdullah University of Science and Technology(KAUST)	2020-03-29	Sara Mfarrej et al
SaudiArabia/KAUST-Madinah53/2020	EPI_ISL_437492	Pathogen Genomics Lab King Abdullah University of Science and Technology(KAUST)	Pathogen Genomics Lab King Abdullah University of Science and Technology(KAUST)	2020-03-29	Sara Mfarrej et al
SaudiArabia/KAUST-Makkah05/2020	EPI_ISL_437461	Pathogen Genomics Lab King Abdullah University of Science and Technology(KAUST)	Pathogen Genomics Lab King Abdullah University of Science and Technology(KAUST)	2020-03-23	Sharif Hala et al
SaudiArabia/KAUST-Makkah06/2020	EPI_ISL_437462	Pathogen Genomics Lab King Abdullah University of Science and Technology(KAUST)	Pathogen Genomics Lab King Abdullah University of Science and Technology(KAUST)	2020-03-23	Sharif Hala et al
SaudiArabia/KAUST-Makkah09/2020	EPI_ISL_437463	Pathogen Genomics Lab King Abdullah University of Science and Technology(KAUST)	Pathogen Genomics Lab King Abdullah University of Science and Technology(KAUST)	2020-03-23	Sharif Hala et al
SaudiArabia/KAUST-Makkah10/2020	EPI_ISL_437464	Pathogen Genomics Lab King Abdullah University of Science and Technology(KAUST)	Pathogen Genomics Lab King Abdullah University of Science and Technology(KAUST)	2020-03-23	Sharif Hala et al
SaudiArabia/KAUST-Makkah13/2020	EPI_ISL_437466	Pathogen Genomics Lab King Abdullah University of Science and Technology(KAUST)	Pathogen Genomics Lab King Abdullah University of Science and Technology(KAUST)	2020-03-23	Sharif Hala et al
SaudiArabia/KAUST-Makkah138/2020	EPI_ISL_437693	Pathogen Genomics Lab King Abdullah University of Science and Technology(KAUST)	Pathogen Genomics Lab King Abdullah University of Science and Technology(KAUST)	2020-04-05	Sharif Hala et al
SaudiArabia/KAUST-Makkah139/2020	EPI_ISL_437694	Pathogen Genomics Lab King Abdullah University of Science and Technology(KAUST)	Pathogen Genomics Lab King Abdullah University of Science and Technology(KAUST)	2020-04-05	Sharif Hala et al
SaudiArabia/KAUST-Makkah156/2020	EPI_ISL_437696	Pathogen Genomics Lab King Abdullah University of Science and Technology(KAUST)	Pathogen Genomics Lab King Abdullah University of Science and Technology(KAUST)	2020-04-09	Sharif Hala et al
SaudiArabia/KAUST-Makkah159/2020	EPI_ISL_437477	Pathogen Genomics Lab King Abdullah University of Science and Technology(KAUST)	Pathogen Genomics Lab King Abdullah University of Science and Technology(KAUST)	2020-04-09	Sharif Hala et al
SaudiArabia/KAUST-Makkah163/2020	EPI_ISL_437697	Pathogen Genomics Lab King Abdullah University of Science and Technology(KAUST)	Pathogen Genomics Lab King Abdullah University of Science and Technology(KAUST)	2020-04-09	Sharif Hala et al
SaudiArabia/KAUST-Makkah165/2020	EPI_ISL_437478	Pathogen Genomics Lab King Abdullah University of Science and Technology(KAUST)	Pathogen Genomics Lab King Abdullah University of Science and Technology(KAUST)	2020-04-09	Sharif Hala et al
SaudiArabia/KAUST-Makkah173/2020	EPI_ISL_437699	Pathogen Genomics Lab King Abdullah University of Science and Technology(KAUST)	Pathogen Genomics Lab King Abdullah University of Science and Technology(KAUST)	2020-04-09	Sharif Hala et al
SaudiArabia/KAUST-Makkah179/2020	EPI_ISL_437702	Pathogen Genomics Lab King Abdullah University of Science and Technology(KAUST)	Pathogen Genomics Lab King Abdullah University of Science and Technology(KAUST)	2020-04-14	Sharif Hala et al
SaudiArabia/KAUST-Makkah18/2020	EPI_ISL_437467	Pathogen Genomics Lab King Abdullah University of Science and Technology(KAUST)	Pathogen Genomics Lab King Abdullah University of Science and Technology(KAUST)	2020-03-23	Sharif Hala et al
SaudiArabia/KAUST-Makkah181/2020	EPI_ISL_437703	Pathogen Genomics Lab King Abdullah University of Science and Technology(KAUST)	Pathogen Genomics Lab King Abdullah University of Science and Technology(KAUST)	2020-04-14	Sharif Hala et al
SaudiArabia/KAUST-Makkah183/2020	EPI_ISL_437704	Pathogen Genomics Lab King Abdullah University of Science and Technology(KAUST)	Pathogen Genomics Lab King Abdullah University of Science and Technology(KAUST)	2020-04-14	Sharif Hala et al
SaudiArabia/KAUST-Makkah189/2020	EPI_ISL_437706	Pathogen Genomics Lab King Abdullah University of Science and Technology(KAUST)	Pathogen Genomics Lab King Abdullah University of Science and Technology(KAUST)	2020-04-14	Sharif Hala et al
SaudiArabia/KAUST-Makkah19/2020	EPI_ISL_437481	Pathogen Genomics Lab King Abdullah University of Science and Technology(KAUST)	Pathogen Genomics Lab King Abdullah University of Science and Technology(KAUST)	2020-03-18	Sara Mfarrej et al
SaudiArabia/KAUST-Makkah193/2020	EPI_ISL_437708	Pathogen Genomics Lab King Abdullah University of Science and Technology(KAUST)	Pathogen Genomics Lab King Abdullah University of Science and Technology(KAUST)	2020-04-14	Sharif Hala et al
SaudiArabia/KAUST-Makkah218/2020	EPI_ISL_437720	Pathogen Genomics Lab King Abdullah University of Science and Technology(KAUST)	Pathogen Genomics Lab King Abdullah University of Science and Technology(KAUST)	2020-04-16	Sharif Hala et al
SaudiArabia/KAUST-Makkah228/2020	EPI_ISL_437724	Pathogen Genomics Lab King Abdullah University of Science and Technology(KAUST)	Pathogen Genomics Lab King Abdullah University of Science and Technology(KAUST)	2020-04-16	Sharif Hala et al
SaudiArabia/KAUST-Makkah229/2020	EPI_ISL_437725	Pathogen Genomics Lab King Abdullah University of Science and Technology(KAUST)	Pathogen Genomics Lab King Abdullah University of Science and Technology(KAUST)	2020-04-16	Sharif Hala et al
SaudiArabia/KAUST-Makkah236/2020	EPI_ISL_437731	Pathogen Genomics Lab King Abdullah University of Science and Technology(KAUST)	Pathogen Genomics Lab King Abdullah University of Science and Technology(KAUST)	2020-04-16	Sharif Hala et al
SaudiArabia/SCDC-3324/2020	EPI_ISL_416522	Public Health Laboratory, Saudi CDC	Public Health Laboratory, Saudi CDC	2020-03-10	Albarrag et al
Scotland/CVR3385/2020	EPI_ISL_456813	West of Scotland Specialist Virology Centre, NHSGGC / MRC-University of Glasgow Centre for Virus Research	COVID-19 Genomics UK (COG-UK) Consortium	2020-05-08	Ana da Silva Filipe et al B (https://virological.org/t/preliminary-analysis-of-sars-cov-2-importation-establishment-of-uk-transmission-lineages/507)
Scotland/CVR3393/2020	EPI_ISL_456820	West of Scotland Specialist Virology Centre, NHSGGC / MRC-University of Glasgow Centre for Virus Research	COVID-19 Genomics UK (COG-UK) Consortium	2020-05-09	Ana da Silva Filipe et al B (https://virological.org/t/preliminary-analysis-of-sars-cov-2-importation-establishment-of-uk-transmission-lineages/507)
Scotland/CVR3414/2020	EPI_ISL_456837	West of Scotland Specialist Virology Centre, NHSGGC / MRC-University of Glasgow Centre for Virus Research	COVID-19 Genomics UK (COG-UK) Consortium	2020-05-06	Ana da Silva Filipe et al B (https://virological.org/t/preliminary-analysis-of-sars-cov-2-importation-establishment-of-uk-transmission-lineages/507)
Scotland/CVR3426/2020	EPI_ISL_456847	West of Scotland Specialist Virology Centre, NHSGGC / MRC-University of Glasgow Centre for Virus Research	COVID-19 Genomics UK (COG-UK) Consortium	2020-05-07	Ana da Silva Filipe et al B (https://virological.org/t/preliminary-analysis-of-sars-cov-2-importation-establishment-of-uk-transmission-lineages/507)
Scotland/CVR3429/2020	EPI_ISL_456850	West of Scotland Specialist Virology Centre, NHSGGC / MRC-University of Glasgow Centre for Virus Research	COVID-19 Genomics UK (COG-UK) Consortium	2020-05-08	Ana da Silva Filipe et al B (https://virological.org/t/preliminary-analysis-of-sars-cov-2-importation-establishment-of-uk-transmission-lineages/507)
Scotland/EDB041/2020	EPI_ISL_425846	Virology Department, Royal Infirmary of Edinburgh, NHS Lothian / School of Biological Sciences, University of Edinburgh / Institute of Genetics and Molecular Medicine, University of Edinburgh	COVID-19 Genomics UK (COG-UK) Consortium	2020-03-12	McHugh M et al A (https://virological.org/t/preliminary-analysis-of-sars-cov-2-importation-establishment-of-uk-transmission-lineages/507)
Scotland/EDB1697/2020	EPI_ISL_433120	Virology Department, Royal Infirmary of Edinburgh, NHS Lothian / School of Biological Sciences, University of Edinburgh / Institute of Genetics and Molecular Medicine, University of Edinburgh	COVID-19 Genomics UK (COG-UK) Consortium	2020-04-15	McHugh M et al A (https://virological.org/t/preliminary-analysis-of-sars-cov-2-importation-establishment-of-uk-transmission-lineages/507)
Scotland/EDB3903/2020	EPI_ISL_439363	Virology Department, Royal Infirmary of Edinburgh, NHS Lothian / School of Biological Sciences, University of Edinburgh / Institute of Genetics and Molecular Medicine, University of Edinburgh	COVID-19 Genomics UK (COG-UK) Consortium	2020-05-01	McHugh M et al A (https://virological.org/t/preliminary-analysis-of-sars-cov-2-importation-establishment-of-uk-transmission-lineages/507)
Scotland/EDB3954/2020	EPI_ISL_449259	Virology Department, Royal Infirmary of Edinburgh, NHS Lothian / School of Biological Sciences, University of Edinburgh / Institute of Genetics and Molecular Medicine, University of Edinburgh	COVID-19 Genomics UK (COG-UK) Consortium	2020-05-03	McHugh M et al A (https://virological.org/t/preliminary-analysis-of-sars-cov-2-importation-establishment-of-uk-transmission-lineages/507)
Scotland/EDB4146/2020	EPI_ISL_456920	Virology Department, Royal Infirmary of Edinburgh, NHS Lothian / School of Biological Sciences, University of Edinburgh / Institute of Genetics and Molecular Medicine, University of Edinburgh	COVID-19 Genomics UK (COG-UK) Consortium	2020-04-18	McHugh M et al A (https://virological.org/t/preliminary-analysis-of-sars-cov-2-importation-establishment-of-uk-transmission-lineages/507)
Scotland/EDB5184/2020	EPI_ISL_456985	Virology Department, Royal Infirmary of Edinburgh, NHS Lothian / School of Biological Sciences, University of Edinburgh / Institute of Genetics and Molecular Medicine, University of Edinburgh	COVID-19 Genomics UK (COG-UK) Consortium	2020-05-19	McHugh M et al A (https://virological.org/t/preliminary-analysis-of-sars-cov-2-importation-establishment-of-uk-transmission-lineages/507)
Scotland/EDB551/2020	EPI_ISL_426015	Virology Department, Royal Infirmary of Edinburgh, NHS Lothian / School of Biological Sciences, University of Edinburgh / Institute of Genetics and Molecular Medicine, University of Edinburgh	COVID-19 Genomics UK (COG-UK) Consortium	2020-03-31	McHugh M et al A (https://virological.org/t/preliminary-analysis-of-sars-cov-2-importation-establishment-of-uk-transmission-lineages/507)
Scotland/EDB5590/2020	EPI_ISL_461759	Virology Department, Royal Infirmary of Edinburgh, NHS Lothian / School of Biological Sciences, University of Edinburgh / Institute of Genetics and Molecular Medicine, University of Edinburgh	COVID-19 Genomics UK (COG-UK) Consortium	2020-05-25	McHugh M et al B
Scotland/GCVR-171388/2020	EPI_ISL_459556	NHSGGC West of Scotland Specialist Virology Centre / MRC-University of Glasgow Centre for Virus Research	Wellcome Sanger Institute for the COVID-19 Genomics UK Consortium	2020-03-26	Ana da Silva Filipe et al A
Senegal/003/2020	EPI_ISL_418206	Institut Pasteur Dakar	Institut Pasteur de Dakar	2020-02-28	Ndongo Dia et al
Senegal/016/2020	EPI_ISL_418207	Institut Pasteur Dakar	Institut Pasteur de Dakar	2020-03-02	Ndongo Dia et al
Senegal/020/2020	EPI_ISL_418208	Institut Pasteur Dakar	Institut Pasteur de Dakar	2020-03-04	Ndongo Dia et al
Senegal/026/2020	EPI_ISL_418209	Institut Pasteur Dakar	Institut Pasteur de Dakar	2020-03-03	Ndongo Dia et al
Senegal/073/2020	EPI_ISL_418210	Institut Pasteur Dakar	Institut Pasteur de Dakar	2020-03-10	Ndongo Dia et al
Senegal/082/2020	EPI_ISL_418211	Institut Pasteur Dakar	Institut Pasteur de Dakar	2020-03-11	Ndongo Dia et al
Senegal/087/2020	EPI_ISL_418212	Institut Pasteur Dakar	Institut Pasteur de Dakar	2020-03-11	Ndongo Dia et al
Senegal/094/2020	EPI_ISL_418213	Institut Pasteur Dakar	Institut Pasteur de Dakar	2020-03-12	Ndongo Dia et al

Senegal/102/2020	EPI_ISL_418214	Institut Pasteur Dakar	Institut Pasteur de Dakar	2020-03-12	Ndongo Dia et al
Senegal/119/2020	EPI_ISL_418215	Instirut Pasteur Dakar	Institut Pasteur de Dakar	2020-03-12	Ndongo Dia et al
Senegal/136/2020	EPI_ISL_418216	Institut Pasteur Dakar	Institut Pasteur de Dakar	2020-03-13	Ndongo Dia et al
Senegal/139/2020	EPI_ISL_418217	Institut Pasteur Dakar	Institut Pasteur de Dakar	2020-03-13	Ndongo Dia et al
Senegal/306/2020	EPI_ISL_420069	Institut Pasteur Dakar	Institut Pasteur de Dakar	2020-03-17	Ndongo Dia et al
Senegal/315/2020	EPI_ISL_420070	Institut Pasteur Dakar	Institut Pasteur de Dakar	2020-03-17	Ndongo Dia et al
Senegal/328/2020	EPI_ISL_420071	Institut Pasteur Dakar	Institut Pasteur de Dakar	2020-03-17	Ndongo Dia et al
Senegal/370/2020	EPI_ISL_420072	Institut Pasteur Dakar	Institut Pasteur de Dakar	2020-03-18	Ndongo Dia et al
Senegal/382/2020	EPI_ISL_420073	Institut Pasteur Dakar	Institut Pasteur de Dakar	2020-03-19	Ndongo Dia et al
Senegal/600/2020	EPI_ISL_420074	Institut Pasteur Dakar	Institut Pasteur de Dakar	2020-03-20	Ndongo Dia et al
Senegal/611/2020	EPI_ISL_420076	Institut Pasteur Dakar	Institut Pasteur de Dakar	2020-03-20	Ndongo Dia et al
Senegal/618/2020	EPI_ISL_420077	Institut Pasteur Dakar	Institut Pasteur de Dakar	2020-03-20	Ndongo Dia et al
Senegal/620/2020	EPI_ISL_420078	Institut Pasteur Dakar	Institut Pasteur de Dakar	2020-03-20	Ndongo Dia et al
Serbia/CA3002-04/2020	EPI_ISL_455480	Veterinary Specialized Institute Kraljevo	Veterinary Specialized Institute Kraljevo	2020-04-07	Vidanovic et al
Serbia/CA3089-04/2020	EPI_ISL_454795	Veterinary Specialized Institute Kraljevo	Veterinary Specialized Institute Kraljevo	2020-04-25	Vidanovic et al
Serbia/KV22-05/2020	EPI_ISL_462435		Laboratory Diagnostic	2020-05-10	Vidanovic et al
Serbia/KV26-04/2020	EPI_ISL_437435	Veterinary Specialized Institue Kraljevo	Veterinary Specialized Institue Kraljevo	2020-04-04	Dejan Vidanovic. Bojana Tesovic et al
Serbia/KV26/2020	EPI_ISL_445086		Laboratory Diagnostic	2020-04-13	Vidanovic et al
Serbia/KV27-04/2020	EPI_ISL_462437		Laboratory Diagnostic	2020-04-23	Vidanovic et al
Serbia/KV77-04/2020	EPI_ISL_462438		Laboratory Diagnostic	2020-04-22	Vidanovic et al
Serbia/NoviPazar-363/2020	EPI_ISL_445087		Laboratory Diagnostic	2020-04-04	Vidanovic et al
Serbia/NP363-04/2020	EPI_ISL_437436	Veterinary Specialized Institue Kraljevo	Veterinary Specialized Institue Kraljevo	2020-04-04	Dejan Vidanovic. Bojana Tesovic et al
Serbia/NS838-04/2020	EPI_ISL_462434		Laboratory Diagnostic	2020-04-01	Vidanovic et al
Serbia/UE38-04/2020	EPI_ISL_462436		Laboratory Diagnostic	2020-04-28	Vidanovic et al
Shandong/IVDC-SD-001/2020	EPI_ISL_408482	National Institute for Viral Disease Control and Prevention, China CDC	National Institute for Viral Disease Control & Prevention, CCDC	2020-01-19	Wenjie Tan et al C (https://dx.doi.org/10.1101/2020.02.29.971101)
Shanghai/SH0002/2020	EPI_ISL_416316	Shanghai Public Health Clinical Center, Shanghai Medical College, Fudan University	National Research Center for Translational Medicine (Shanghai), Ruijin Hospital affiliated to Shanghai Jiao Tong University School of Medicine & Shanghai Public Health Clinical Center	2020-01-25	Shengyue Wang et al
Shanghai/SH0013/2020	EPI_ISL_416326	Shanghai Public Health Clinical Center, Shanghai Medical College, Fudan University	National Research Center for Translational Medicine (Shanghai), Ruijin Hospital affiliated to Shanghai Jiao Tong University School of Medicine & Shanghai Public Health Clinical Center	2020-01-30	Shengyue Wang et al
Shanghai/SH0039/2020	EPI_ISL_416348	Shanghai Public Health Clinical Center, Shanghai Medical College, Fudan University	National Research Center for Translational Medicine (Shanghai), Ruijin Hospital affiliated to Shanghai Jiao Tong University School of Medicine & Shanghai Public Health Clinical Center	2020-02-06	Shengyue Wang et al
Shanghai/SH0053/2020	EPI_ISL_416361	Shanghai Public Health Clinical Center, Shanghai Medical College, Fudan University	National Research Center for Translational Medicine (Shanghai), Ruijin Hospital affiliated to Shanghai Jiao Tong University School of Medicine & Shanghai Public Health Clinical Center	2020-02-01	Shengyue Wang et al
Shanghai/SH0086/2020	EPI_ISL_416386	Shanghai Public Health Clinical Center, Shanghai Medical College, Fudan University	National Research Center for Translational Medicine (Shanghai), Ruijin Hospital affiliated to Shanghai Jiao Tong University School of Medicine & Shanghai Public Health Clinical Center	2020-01-31	Shengyue Wang et al
Shanghai/SH0110/2020	EPI_ISL_416399	Shanghai Public Health Clinical Center, Shanghai Medical College, Fudan University	National Research Center for Translational Medicine (Shanghai), Ruijin Hospital affiliated to Shanghai Jiao Tong University School of Medicine & Shanghai Public Health Clinical Center	2020-02-13	Shengyue Wang et al
Sichuan/SC-GA-062/2020	EPI_ISL_451313	West China Hospital of Sichuan University	State Key Laboratory of Biotherapy of Sichuan University	2020-01-26	Baowen Du et al
Sichuan/SC-MY-055/2020	EPI_ISL_451321	West China Hospital of Sichuan University	State Key Laboratory of Biotherapy of Sichuan University	2020-02-02	Baowen Du et al
Sichuan/SC-WCH-310/2020	EPI_ISL_451380	West China Hospital of Sichuan University	State Key Laboratory of Biotherapy of Sichuan University	2020-01-24	Baowen Du et al
Sichuan/SC-WCH4-286/2020	EPI_ISL_451388	West China Hospital of Sichuan University	State Key Laboratory of Biotherapy of Sichuan University	2020-02-10	Baowen Du et al
Singapore/1/2020	EPI_ISL_406973	Singapore General Hospital	National Public Health Laboratory	2020-01-23	Mak et al (https://dx.doi.org/10.2807/1560-7917.ES.2020.25.13.2000305)
Singapore/10/2020	EPI_ISL_410716	National Public Health Laboratory, National Centre for Infectious Diseases	National Centre for Infectious Diseases, National Centre for Infectious Diseases	2020-02-04	Octavia S et al
Singapore/101/2020	EPI_ISL_443193	National Public Health Laboratory, National Centre for Infectious Diseases	National Public Health Laboratory, National Centre for Infectious Diseases	2020-03-16	Mak Tze Minn et al
Singapore/103/2020	EPI_ISL_443195	National Public Health Laboratory, National Centre for Infectious Diseases	National Public Health Laboratory, National Centre for Infectious Diseases	2020-03-11	Mak Tze Minn et al
Singapore/11/2020	EPI_ISL_410719	National Public Health Laboratory	National Public Health Laboratory	2020-02-02	Octavia S et al
Singapore/113/2020	EPI_ISL_443205	National Public Health Laboratory, National Centre for Infectious Diseases	National Public Health Laboratory, National Centre for Infectious Diseases	2020-03-18	Mak Tze Minn et al
Singapore/12/2020	EPI_ISL_414378	National Centre for Infectious Diseases	Programme in Emerging Infectious Diseases, Duke-NUS Medical School	2020-02-17	Danielle E Anderson et al B (https://dx.doi.org/doi/10.1101/2020.03.11.987222)
Singapore/121/2020	EPI_ISL_443213	National Public Health Laboratory, National Centre for Infectious Diseases	National Public Health Laboratory, National Centre for Infectious Diseases	2020-04-08	Mak Tze Minn et al
Singapore/124/2020	EPI_ISL_443216	National Public Health Laboratory, National Centre for Infectious Diseases	National Public Health Laboratory, National Centre for Infectious Diseases	2020-03-19	Mak Tze Minn et al
Singapore/12Clin/2020	EPI_ISL_418995	National Public Health Laboratory, National Centre for Infectious Diseases	National Public Health Laboratory, National Centre for Infectious Diseases	2020-02-28	Mak TM et al
Singapore/13/2020	EPI_ISL_414379	National Centre for Infectious Diseases	Programme in Emerging Infectious Diseases, Duke-NUS Medical School	2020-02-18	Danielle E Anderson et al B (https://dx.doi.org/doi/10.1101/2020.03.11.987222)
Singapore/14/2020	EPI_ISL_414380	National Centre for Infectious Diseases	Programme in Emerging Infectious Diseases, Duke-NUS Medical School	2020-02-13	Danielle E Anderson et al B (https://dx.doi.org/doi/10.1101/2020.03.11.987222)
Singapore/141/2020	EPI_ISL_443233	National Public Health Laboratory, National Centre for Infectious Diseases	National Public Health Laboratory, National Centre for Infectious Diseases	2020-03-17	Mak Tze Minn et al
Singapore/144/2020	EPI_ISL_443236	National Public Health Laboratory, National Centre for Infectious Diseases	National Public Health Laboratory, National Centre for Infectious Diseases	2020-03-17	Mak Tze Minn et al
Singapore/14Clin/2020	EPI_ISL_418998	National Public Health Laboratory, National Centre for Infectious Diseases	National Public Health Laboratory, National Centre for Infectious Diseases	2020-02-14	Mak TM et al
Singapore/15/2020	EPI_ISL_418996	National Public Health Laboratory, National Centre for Infectious Diseases	National Public Health Laboratory, National Centre for Infectious Diseases	2020-01-27	Mak TM et al
Singapore/158/2020	EPI_ISL_462276	National Public Health Laboratory, National Centre for Infectious Diseases	National Public Health Laboratory, National Centre for Infectious Diseases	2020-04-15	Mak TM et al
Singapore/16/2020	EPI_ISL_418997	National Public Health Laboratory, National Centre for Infectious Diseases	National Public Health Laboratory, National Centre for Infectious Diseases	2020-02-06	Mak TM et al
Singapore/165/2020	EPI_ISL_462283	National Public Health Laboratory, National Centre for Infectious Diseases	National Public Health Laboratory, National Centre for Infectious Diseases	2020-01-27	Mak TM et al
Singapore/169/2020	EPI_ISL_462287	National Public Health Laboratory, National Centre for Infectious Diseases	National Public Health Laboratory, National Centre for Infectious Diseases	2020-03-14	Mak TM et al
Singapore/17/2020	EPI_ISL_418992	National Public Health Laboratory, National Centre for Infectious Diseases	National Public Health Laboratory, National Centre for Infectious Diseases	2020-02-10	Mak TM et al
Singapore/174/2020	EPI_ISL_462292	National Public Health Laboratory, National Centre for Infectious Diseases	National Public Health Laboratory, National Centre for Infectious Diseases	2020-03-17	Mak TM et al
Singapore/175/2020	EPI_ISL_462293	National Public Health Laboratory, National Centre for Infectious Diseases	National Public Health Laboratory, National Centre for Infectious Diseases	2020-02-09	Mak TM et al
Singapore/185/2020	EPI_ISL_462303	National Public Health Laboratory, National Centre for Infectious Diseases	National Public Health Laboratory, National Centre for Infectious Diseases	2020-04-28	Mak TM et al
Singapore/187/2020	EPI_ISL_462305	National Public Health Laboratory, National Centre for Infectious Diseases	National Public Health Laboratory, National Centre for Infectious Diseases	2020-02-27	Mak TM et al
Singapore/188/2020	EPI_ISL_462306	National Public Health Laboratory, National Centre for Infectious Diseases	National Public Health Laboratory, National Centre for Infectious Diseases	2020-02-14	Mak TM et al
Singapore/190/2020	EPI_ISL_462308	National Public Health Laboratory, National Centre for Infectious Diseases	National Public Health Laboratory, National Centre for Infectious Diseases	2020-04-20	Mak TM et al
Singapore/191/2020	EPI_ISL_462309	National Public Health Laboratory, National Centre for Infectious Diseases	National Public Health Laboratory, National Centre for Infectious Diseases	2020-04-20	Mak TM et al
Singapore/194/2020	EPI_ISL_462312	National Public Health Laboratory, National Centre for Infectious Diseases	National Public Health Laboratory, National Centre for Infectious Diseases	2020-04-20	Mak TM et al
Singapore/2/2020	EPI_ISL_407987	Singapore General Hospital	Programme in Emerging Infectious Diseases, Duke-NUS Medical School	2020-01-25	Danielle E Anderson et al B (https://dx.doi.org/doi/10.1101/2020.03.11.987222)
Singapore/20/2020	EPI_ISL_418993	National Public Health Laboratory, National Centre for Infectious Diseases	National Public Health Laboratory, National Centre for Infectious Diseases	2020-01-29	Mak TM et al
Singapore/200/2020	EPI_ISL_462318	National Public Health Laboratory, National Centre for Infectious Diseases	National Public Health Laboratory, National Centre for Infectious Diseases	2020-04-23	Mak TM et al
Singapore/207/2020	EPI_ISL_462325	National Public Health Laboratory, National Centre for Infectious Diseases	National Public Health Laboratory, National Centre for Infectious Diseases	2020-04-29	Mak TM et al
Singapore/209/2020	EPI_ISL_462327	National Public Health Laboratory, National Centre for Infectious Diseases	National Public Health Laboratory, National Centre for Infectious Diseases	2020-05-04	Mak TM et al
Singapore/21/2020	EPI_ISL_419000	National Public Health Laboratory, National Centre for Infectious Diseases	National Public Health Laboratory, National Centre for Infectious Diseases	2020-02-13	Mak TM et al
Singapore/211/2020	EPI_ISL_462329	National Public Health Laboratory, National Centre for Infectious Diseases	National Public Health Laboratory, National Centre for Infectious Diseases	2020-03-02	Mak TM et al
Singapore/215/2020	EPI_ISL_462333	National Public Health Laboratory, National Centre for Infectious Diseases	National Public Health Laboratory, National Centre for Infectious Diseases	2020-03-26	Mak TM et al
Singapore/220/2020	EPI_ISL_462338	National Public Health Laboratory, National Centre for Infectious Diseases	National Public Health Laboratory, National Centre for Infectious Diseases	2020-03-06	Mak TM et al
Singapore/229/2020	EPI_ISL_462347	National Public Health Laboratory, National Centre for Infectious Diseases	National Public Health Laboratory, National Centre for Infectious Diseases	2020-04-14	Mak TM et al
Singapore/230/2020	EPI_ISL_462348	National Public Health Laboratory, National Centre for Infectious Diseases	National Public Health Laboratory, National Centre for Infectious Diseases	2020-04-06	Mak TM et al
Singapore/237/2020	EPI_ISL_462355	National Public Health Laboratory, National Centre for Infectious Diseases	National Public Health Laboratory, National Centre for Infectious Diseases	2020-04-03	Mak TM et al
Singapore/240/2020	EPI_ISL_462358	National Public Health Laboratory, National Centre for Infectious Diseases	National Public Health Laboratory, National Centre for Infectious Diseases	2020-03-24	Mak TM et al
Singapore/244/2020	EPI_ISL_462362	National Public Health Laboratory, National Centre for Infectious Diseases	National Public Health Laboratory, National Centre for Infectious Diseases	2020-05-06	Mak TM et al
Singapore/25/2020	EPI_ISL_420102	National Centre for Infectious Diseases	Programme in Emerging Infectious Diseases, Duke-NUS Medical School	2020-03-05	Danielle E Anderson et al A
Singapore/254/2020	EPI_ISL_462372	National Public Health Laboratory, National Centre for Infectious Diseases	National Public Health Laboratory, National Centre for Infectious Diseases	2020-05-15	Mak TM et al
Singapore/255/2020	EPI_ISL_462373	National Public Health Laboratory, National Centre for Infectious Diseases	National Public Health Laboratory, National Centre for Infectious Diseases	2020-03-10	Mak TM et al
Singapore/258/2020	EPI_ISL_462376	National Public Health Laboratory, National Centre for Infectious Diseases	National Public Health Laboratory, National Centre for Infectious Diseases	2020-03-21	Mak TM et al
Singapore/264/2020	EPI_ISL_462382	National Public Health Laboratory, National Centre for Infectious Diseases	National Public Health Laboratory, National Centre for Infectious Diseases	2020-05-06	Mak TM et al
Singapore/267/2020	EPI_ISL_462385	National Public Health Laboratory, National Centre for Infectious Diseases	National Public Health Laboratory, National Centre for Infectious Diseases	2020-03-21	Mak TM et al
Singapore/273/2020	EPI_ISL_462391	National Public Health Laboratory, National Centre for Infectious Diseases	National Public Health Laboratory, National Centre for Infectious Diseases	2020-03-21	Mak TM et al
Singapore/275/2020	EPI_ISL_462393	National Public Health Laboratory, National Centre for Infectious Diseases	National Public Health Laboratory, National Centre for Infectious Diseases	2020-05-06	Mak TM et al
Singapore/29/2020	EPI_ISL_420106	National Centre for Infectious Diseases	Programme in Emerging Infectious Diseases, Duke-NUS Medical School	2020-03-06	Danielle E Anderson et al A
Singapore/292/2020	EPI_ISL_462410	National Public Health Laboratory, National Centre for Infectious Diseases	National Public Health Laboratory, National Centre for Infectious Diseases	2020-03-20	Mak TM et al
Singapore/294/2020	EPI_ISL_462412	National Public Health Laboratory, National Centre for Infectious Diseases	National Public Health Laboratory, National Centre for Infectious Diseases	2020-05-03	Mak TM et al
Singapore/3/2020	EPI_ISL_407988	National Centre for Infectious Diseases	Programme in Emerging Infectious Diseases, Duke-NUS Medical School	2020-02-01	Danielle E Anderson et al B (https://dx.doi.org/doi/10.1101/2020.03.11.987222)
Singapore/306/2020	EPI_ISL_462424	National Public Health Laboratory, National Centre for Infectious Diseases	National Public Health Laboratory, National Centre for Infectious Diseases	2020-04-14	Mak TM et al
Singapore/309/2020	EPI_ISL_462427	National Public Health Laboratory, National Centre for Infectious Diseases	National Public Health Laboratory, National Centre for Infectious Diseases	2020-05-15	Mak TM et al
Singapore/311/2020	EPI_ISL_462429	National Public Health Laboratory, National Centre for Infectious Diseases	National Public Health Laboratory, National Centre for Infectious Diseases	2020-03-17	Mak TM et al
Singapore/315/2020	EPI_ISL_462433	National Public Health Laboratory, National Centre for Infectious Diseases	National Public Health Laboratory, National Centre for Infectious Diseases	2020-02-19	Mak TM et al
Singapore/323ilmn/2020	EPI_ISL_469100	National Public Health Laboratory, National Centre for Infectious Diseases	National Public Health Laboratory, National Centre for Infectious Diseases	2020-03-26	Mak TM et al
Singapore/325/2020	EPI_ISL_462089	Singapore General Hospital	Department of Microbiology	2020-03-25	Nurdyana Abdul Rahman et al
Singapore/329/2020	EPI_ISL_469083	National Public Health Laboratory, National Centre for Infectious Diseases	National Public Health Laboratory, National Centre for Infectious Diseases	2020-04-17	Mak TM et al
Singapore/335/2020	EPI_ISL_469089	National Public Health Laboratory, National Centre for Infectious Diseases	National Public Health Laboratory, National Centre for Infectious Diseases	2020-04-14	Mak TM et al
Singapore/337/2020	EPI_ISL_469091	National Public Health Laboratory, National Centre for Infectious Diseases	National Public Health Laboratory, National Centre for Infectious Diseases	2020-04-17	Mak TM et al
Singapore/34/2020	EPI_ISL_420111	National Centre for Infectious Diseases	Programme in Emerging Infectious Diseases, Duke-NUS Medical School	2020-03-12	Danielle E Anderson et al A
Singapore/342/2020	EPI_ISL_469096	National Public Health Laboratory, National Centre for Infectious Diseases	National Public Health Laboratory, National Centre for Infectious Diseases	2020-05-05	Mak TM et al
Singapore/343/2020	EPI_ISL_469097	National Public Health Laboratory, National Centre for Infectious Diseases	National Public Health Laboratory, National Centre for Infectious Diseases	2020-04-09	Mak TM et al

Singapore/347/2020	EPI_ISL_469102	National Public Health Laboratory, National Centre for Infectious Diseases	National Public Health Laboratory, National Centre for Infectious Diseases	2020-05-20	Mak TM et al
Singapore/35/2020	EPI_ISL_422428	National Public Health Laboratory, National Centre for Infectious Diseases	National Public Health Laboratory, National Centre for Infectious Diseases	2020-02-06	Mak TM et al
Singapore/352/2020	EPI_ISL_469107	National Public Health Laboratory, National Centre for Infectious Diseases	National Public Health Laboratory, National Centre for Infectious Diseases	2020-05-28	Mak TM et al
Singapore/353/2020	EPI_ISL_469108	National Public Health Laboratory, National Centre for Infectious Diseases	National Public Health Laboratory, National Centre for Infectious Diseases	2020-05-14	Mak TM et al
Singapore/354/2020	EPI_ISL_469109	National Public Health Laboratory, National Centre for Infectious Diseases	National Public Health Laboratory, National Centre for Infectious Diseases	2020-04-07	Mak TM et al
Singapore/356/2020	EPI_ISL_469111	National Public Health Laboratory, National Centre for Infectious Diseases	National Public Health Laboratory, National Centre for Infectious Diseases	2020-04-08	Mak TM et al
Singapore/36/2020	EPI_ISL_422429	National Public Health Laboratory, National Centre for Infectious Diseases	National Public Health Laboratory, National Centre for Infectious Diseases	2020-02-16	Mak TM et al
Singapore/360/2020	EPI_ISL_469115	National Public Health Laboratory, National Centre for Infectious Diseases	National Public Health Laboratory, National Centre for Infectious Diseases	2020-05-20	Mak TM et al
Singapore/362/2020	EPI_ISL_469117	National Public Health Laboratory, National Centre for Infectious Diseases	National Public Health Laboratory, National Centre for Infectious Diseases	2020-04-13	Mak TM et al
Singapore/38/2020	EPI_ISL_422431	National Public Health Laboratory, National Centre for Infectious Diseases	National Public Health Laboratory, National Centre for Infectious Diseases	2020-03-31	Mak TM et al
Singapore/383/2020	EPI_ISL_469132	National Public Health Laboratory, National Centre for Infectious Diseases	National Public Health Laboratory, National Centre for Infectious Diseases	2020-02-15	Mak TM et al
Singapore/386/2020	EPI_ISL_469135	National Public Health Laboratory, National Centre for Infectious Diseases	National Public Health Laboratory, National Centre for Infectious Diseases	2020-04-17	Mak TM et al
Singapore/387/2020	EPI_ISL_469136	National Public Health Laboratory, National Centre for Infectious Diseases	National Public Health Laboratory, National Centre for Infectious Diseases	2020-04-17	Mak TM et al
Singapore/390/2020	EPI_ISL_469139	National Public Health Laboratory, National Centre for Infectious Diseases	National Public Health Laboratory, National Centre for Infectious Diseases	2020-06-06	Mak TM et al
Singapore/397/2020	EPI_ISL_469146	National Public Health Laboratory, National Centre for Infectious Diseases	National Public Health Laboratory, National Centre for Infectious Diseases	2020-06-06	Mak TM et al
Singapore/398/2020	EPI_ISL_469147	National Public Health Laboratory, National Centre for Infectious Diseases	National Public Health Laboratory, National Centre for Infectious Diseases	2020-05-21	Mak TM et al
Singapore/3Clin/2020	EPI_ISL_418994	National Public Health Laboratory, National Centre for Infectious Diseases	National Public Health Laboratory, National Centre for Infectious Diseases	2020-01-29	Mak TM et al
Singapore/4/2020	EPI_ISL_410535	National Centre for Infectious Diseases	Programme in Emerging Infectious Diseases, Duke-NUS Medical School	2020-02-03	Danielle E Anderson et al B (https://dx.doi.org/doi/10.1101/2020.03.11.987222)
Singapore/40/2020	EPI_ISL_422433	National Public Health Laboratory, National Centre for Infectious Diseases	National Public Health Laboratory, National Centre for Infectious Diseases	2020-03-31	Mak TM et al
Singapore/42/2020	EPI_ISL_422435	National Public Health Laboratory, National Centre for Infectious Diseases	National Public Health Laboratory, National Centre for Infectious Diseases	2020-03-31	Mak TM et al
Singapore/43/2020	EPI_ISL_428822	National Public Health Laboratory, National Centre for Infectious Diseases	National Public Health Laboratory, National Centre for Infectious Diseases	2020-02-16	Mak TM et al
Singapore/45/2020	EPI_ISL_428824	National Public Health Laboratory, National Centre for Infectious Diseases	National Public Health Laboratory, National Centre for Infectious Diseases	2020-03-16	Mak TM et al
Singapore/5/2020	EPI_ISL_410536	Singapore General Hospital, Molecular Laboratory, Division of Pathology	Programme in Emerging Infectious Diseases, Duke-NUS Medical School	2020-02-06	Danielle E Anderson et al B (https://dx.doi.org/doi/10.1101/2020.03.11.987222)
Singapore/51/2020	EPI_ISL_428830	National Public Health Laboratory, National Centre for Infectious Diseases	National Public Health Laboratory, National Centre for Infectious Diseases	2020-02-27	Mak TM et al
Singapore/6/2020	EPI_ISL_410537	Singapore General Hospital, Molecular Laboratory, Division of Pathology	Programme in Emerging Infectious Diseases, Duke-NUS Medical School	2020-02-09	Danielle E Anderson et al B (https://dx.doi.org/doi/10.1101/2020.03.11.987222)
Singapore/60/2020	EPI_ISL_428839	National Public Health Laboratory, National Centre for Infectious Diseases	National Public Health Laboratory, National Centre for Infectious Diseases	2020-04-08	Mak TM et al
Singapore/61/2020	EPI_ISL_428840	National Public Health Laboratory, National Centre for Infectious Diseases	National Public Health Laboratory, National Centre for Infectious Diseases	2020-04-07	Mak TM et al
Singapore/66/2020	EPI_ISL_428845	National Public Health Laboratory, National Centre for Infectious Diseases	National Public Health Laboratory, National Centre for Infectious Diseases	2020-04-08	Mak TM et al
Singapore/7/2020	EPI_ISL_410713	National Public Health Laboratory, National Centre for Infectious Diseases	National Public Health Laboratory, National Centre for Infectious Diseases	2020-01-27	Octavia S et al
Singapore/70/2020	EPI_ISL_428849	National Public Health Laboratory, National Centre for Infectious Diseases	National Public Health Laboratory, National Centre for Infectious Diseases	2020-04-15	Mak TM et al
Singapore/71/2020	EPI_ISL_428850	National Public Health Laboratory, National Centre for Infectious Diseases	National Public Health Laboratory, National Centre for Infectious Diseases	2020-04-15	Mak TM et al
Singapore/77/2020	EPI_ISL_435682	National Public Health Laboratory, National Centre for Infectious Diseases	National Public Health Laboratory, National Centre for Infectious Diseases	2020-04-07	Mak Tze Minn et al
Singapore/79/2020	EPI_ISL_435684	National Public Health Laboratory, National Centre for Infectious Diseases	National Public Health Laboratory, National Centre for Infectious Diseases	2020-04-11	Mak Tze Minn et al
Singapore/8/2020	EPI_ISL_410714	National Public Health Laboratory, National Centre for Infectious Diseases	National Public Health Laboratory, National Centre for Infectious Diseases	2020-02-03	Octavia S et al
Singapore/84/2020	EPI_ISL_435689	National Public Health Laboratory, National Centre for Infectious Diseases	National Public Health Laboratory, National Centre for Infectious Diseases	2020-04-18	Mak Tze Minn et al
Singapore/85/2020	EPI_ISL_435690	National Public Health Laboratory, National Centre for Infectious Diseases	National Public Health Laboratory, National Centre for Infectious Diseases	2020-04-19	Mak Tze Minn et al
Singapore/9/2020	EPI_ISL_410715	National Public Health Laboratory, National Centre for Infectious Diseases	National Public Health Laboratory, National Centre for Infectious Diseases	2020-02-04	Octavia S et al
Singapore/95/2020	EPI_ISL_435700	National Public Health Laboratory, National Centre for Infectious Diseases	National Public Health Laboratory, National Centre for Infectious Diseases	2020-03-18	Mak Tze Minn et al
Singapore/96/2020	EPI_ISL_443188	National Public Health Laboratory, National Centre for Infectious Diseases	National Public Health Laboratory, National Centre for Infectious Diseases	2020-03-18	Mak Tze Minn et al
Slovakia/SK-BMC1/2020	EPI_ISL_417877	Institute of Virology, Biomedical Research Center of the Slovak Academy of Sciences, Bratislava; Public Health Authority of the Slovak Republic, Bratislava	Institute of Virology, Biomedical Research Center of the Slovak Academy of Sciences, Bratislava; Comenius University Science Park, Bratislava	2020-03-06	Monika Slávikova et al
Slovakia/SK-BMC2/2020	EPI_ISL_417878	Institute of Virology, Biomedical Research Center of the Slovak Academy of Sciences, Bratislava; Public Health Authority of the Slovak Republic, Bratislava	Institute of Virology, Biomedical Research Center of the Slovak Academy of Sciences, Bratislava; Comenius University Science Park, Bratislava	2020-03-07	Monika Slávikova et al
Slovakia/SK-BMC5/2020	EPI_ISL_417879	Institute of Virology, Biomedical Research Center of the Slovak Academy of Sciences, Bratislava; Public Health Authority of the Slovak Republic, Bratislava	Institute of Virology, Biomedical Research Center of the Slovak Academy of Sciences, Bratislava; Comenius University Science Park, Bratislava	2020-03-06	Monika Slávikova et al
Slovakia/SK-BMC6/2020	EPI_ISL_417880	Institute of Virology, Biomedical Research Center of the Slovak Academy of Sciences, Bratislava; Public Health Authority of the Slovak Republic, Bratislava	Institute of Virology, Biomedical Research Center of the Slovak Academy of Sciences, Bratislava; Comenius University Science Park, Bratislava	2020-03-08	Monika Slávikova et al
Slovenia/12304/2020	EPI_ISL_420295	Institute of Microbiology and Immunology, Faculty of Medicine, University of Ljubljana	Institute of Microbiology and Immunology, Faculty of Medicine, University of Ljubljana	2020-03-24	Samo Zakotnik et al
Slovenia/4584/2020	EPI_ISL_420294	Institute of Microbiology and Immunology, Faculty of Medicine, University of Ljubljana	Institute of Microbiology and Immunology, Faculty of Medicine, University of Ljubljana	2020-03-06	Samo Zakotnik et al
Slovenia/MB0329/2020	EPI_ISL_426379	The National Laboratory of Health, Environment and Food, Maribor, Slovenia	The National Laboratory of Health, Environment and Food, Maribor, Slovenia	2020-03-29	Mahnic A. et al
Slovenia/MB0419/2020	EPI_ISL_449799	National Laboratory for Health, Environment and Food	National Laboratory for Health, Environment and Food	2020-04-19	Mahnic A. et al
SouthAfrica/KRISP-0001/2020	EPI_ISL_467431	Molecular Diagnostics Services (MDS)	KRISP, KZN Research Innovation and Sequencing Platform	2020-03-23	Giandhari J et al B
SouthAfrica/KRISP-0016/2020	EPI_ISL_467432	AMPATH-DBN	KRISP, KZN Research Innovation and Sequencing Platform	2020-03-28	Giandhari J et al B
SouthAfrica/KRISP-0019/2020	EPI_ISL_467434	AMPATH-DBN	KRISP, KZN Research Innovation and Sequencing Platform	2020-04-02	Giandhari J et al B
SouthAfrica/KRISP-0021/2020	EPI_ISL_467435	AMPATH-DBN	KRISP, KZN Research Innovation and Sequencing Platform	2020-04-02	Giandhari J et al B
SouthAfrica/KRISP-0029/2020	EPI_ISL_467439	NHLS-IALCH	KRISP, KZN Research Innovation and Sequencing Platform	2020-04-02	Giandhari J et al B
SouthAfrica/KRISP-0033/2020	EPI_ISL_467440	NHLS-IALCH	KRISP, KZN Research Innovation and Sequencing Platform	2020-04-01	Giandhari J et al B
SouthAfrica/KRISP-0035/2020	EPI_ISL_467441	NHLS-IALCH	KRISP, KZN Research Innovation and Sequencing Platform	2020-03-27	Giandhari J et al B
SouthAfrica/KRISP-0040/2020	EPI_ISL_467442	NHLS-IALCH	KRISP, KZN Research Innovation and Sequencing Platform	2020-03-27	Giandhari J et al B
SouthAfrica/KRISP-0041/2020	EPI_ISL_467443	NHLS-IALCH	KRISP, KZN Research Innovation and Sequencing Platform	2020-03-25	Giandhari J et al B
SouthAfrica/KRISP-0055/2020	EPI_ISL_467444	Molecular Diagnostics Services (MDS)	KRISP, KZN Research Innovation and Sequencing Platform	2020-03-24	Giandhari J et al B
SouthAfrica/KRISP-0056/2020	EPI_ISL_467445	Molecular Diagnostics Services (MDS)	KRISP, KZN Research Innovation and Sequencing Platform	2020-04-04	Giandhari J et al B
SouthAfrica/KRISP-0057/2020	EPI_ISL_467446	Molecular Diagnostics Services (MDS)	KRISP, KZN Research Innovation and Sequencing Platform	2020-03-29	Giandhari J et al B
SouthAfrica/KRISP-0058/2020	EPI_ISL_467447	Molecular Diagnostics Services (MDS)	KRISP, KZN Research Innovation and Sequencing Platform	2020-04-04	Giandhari J et al B
SouthAfrica/KRISP-0059/2020	EPI_ISL_467448	Molecular Diagnostics Services (MDS)	KRISP, KZN Research Innovation and Sequencing Platform	2020-04-01	Giandhari J et al B
SouthAfrica/KRISP-0066/2020	EPI_ISL_467450	AMPATH-DBN	KRISP, KZN Research Innovation and Sequencing Platform	2020-04-03	Giandhari J et al B
SouthAfrica/KRISP-0067/2020	EPI_ISL_467451	AMPATH-DBN	KRISP, KZN Research Innovation and Sequencing Platform	2020-04-03	Giandhari J et al B
SouthAfrica/KRISP-0080/2020	EPI_ISL_467454	AMPATH-DBN	KRISP, KZN Research Innovation and Sequencing Platform	2020-04-01	Giandhari J et al B
SouthAfrica/KRISP-0084/2020	EPI_ISL_467456	AMPATH-DBN	KRISP, KZN Research Innovation and Sequencing Platform	2020-04-05	Giandhari J et al B
SouthAfrica/KRISP-0088/2020	EPI_ISL_467457	AMPATH-DBN	KRISP, KZN Research Innovation and Sequencing Platform	2020-04-07	Giandhari J et al B
SouthAfrica/KRISP-0089/2020	EPI_ISL_467458	AMPATH-DBN	KRISP, KZN Research Innovation and Sequencing Platform	2020-04-05	Giandhari J et al B
SouthAfrica/KRISP-0090/2020	EPI_ISL_467459	AMPATH-DBN	KRISP, KZN Research Innovation and Sequencing Platform	2020-04-03	Giandhari J et al B
SouthAfrica/KRISP-0091/2020	EPI_ISL_467460	AMPATH-DBN	KRISP, KZN Research Innovation and Sequencing Platform	2020-04-05	Giandhari J et al B
SouthAfrica/KRISP-0096/2020	EPI_ISL_467461	AMPATH-DBN	KRISP, KZN Research Innovation and Sequencing Platform	2020-04-01	Giandhari J et al B
SouthAfrica/KRISP-0097/2020	EPI_ISL_467462	AMPATH-DBN	KRISP, KZN Research Innovation and Sequencing Platform	2020-04-01	Giandhari J et al B
SouthAfrica/KRISP-0111/2020	EPI_ISL_467465	AMPATH-DBN	KRISP, KZN Research Innovation and Sequencing Platform	2020-04-01	Giandhari J et al B
SouthAfrica/KRISP-0112/2020	EPI_ISL_467466	AMPATH-DBN	KRISP, KZN Research Innovation and Sequencing Platform	2020-04-05	Giandhari J et al B
SouthAfrica/KRISP-0113/2020	EPI_ISL_467467	AMPATH-DBN	KRISP, KZN Research Innovation and Sequencing Platform	2020-04-05	Giandhari J et al B
SouthAfrica/KRISP-0115/2020	EPI_ISL_467468	AMPATH-DBN	KRISP, KZN Research Innovation and Sequencing Platform	2020-04-04	Giandhari J et al B
SouthAfrica/KRISP-0116/2020	EPI_ISL_467469	AMPATH-DBN	KRISP, KZN Research Innovation and Sequencing Platform	2020-04-03	Giandhari J et al B
SouthAfrica/KRISP-0118/2020	EPI_ISL_467470	AMPATH-DBN	KRISP, KZN Research Innovation and Sequencing Platform	2020-04-01	Giandhari J et al B
SouthAfrica/KRISP-0119/2020	EPI_ISL_467471	AMPATH-DBN	KRISP, KZN Research Innovation and Sequencing Platform	2020-04-01	Giandhari J et al B
SouthAfrica/KRISP-012/2020	EPI_ISL_421576	Molecular Diagnostic Services	KRISP, KZN Research Innovation and Sequencing Platform	2020-04-01	Giandhari J et al A (https://dx.doi.org/10.1101/2020.05.29.20116376)
SouthAfrica/KRISP-0120/2020	EPI_ISL_467472	AMPATH-DBN	KRISP, KZN Research Innovation and Sequencing Platform	2020-04-01	Giandhari J et al B
SouthAfrica/KRISP-0123/2020	EPI_ISL_467475	Molecular Diagnostics Services (MDS)	KRISP, KZN Research Innovation and Sequencing Platform	2020-05-14	Giandhari J et al B
SouthAfrica/KRISP-0125/2020	EPI_ISL_467477	Molecular Diagnostics Services (MDS)	KRISP, KZN Research Innovation and Sequencing Platform	2020-05-13	Giandhari J et al B
SouthAfrica/KRISP-0126/2020	EPI_ISL_467478	Molecular Diagnostics Services (MDS)	KRISP, KZN Research Innovation and Sequencing Platform	2020-05-13	Giandhari J et al B
SouthAfrica/KRISP-0127/2020	EPI_ISL_467479	Molecular Diagnostics Services (MDS)	KRISP, KZN Research Innovation and Sequencing Platform	2020-05-13	Giandhari J et al B
SouthAfrica/KRISP-0128/2020	EPI_ISL_467480	Molecular Diagnostics Services (MDS)	KRISP, KZN Research Innovation and Sequencing Platform	2020-05-13	Giandhari J et al B
SouthAfrica/KRISP-0129/2020	EPI_ISL_467481	Molecular Diagnostics Services (MDS)	KRISP, KZN Research Innovation and Sequencing Platform	2020-05-13	Giandhari J et al B
SouthAfrica/KRISP-0130/2020	EPI_ISL_467482	Molecular Diagnostics Services (MDS)	KRISP, KZN Research Innovation and Sequencing Platform	2020-05-13	Giandhari J et al B
SouthAfrica/KRISP-0131/2020	EPI_ISL_467483	Molecular Diagnostics Services (MDS)	KRISP, KZN Research Innovation and Sequencing Platform	2020-05-13	Giandhari J et al B
SouthAfrica/KRISP-0132/2020	EPI_ISL_467484	Molecular Diagnostics Services (MDS)	KRISP, KZN Research Innovation and Sequencing Platform	2020-05-13	Giandhari J et al B
SouthAfrica/KRISP-0133/2020	EPI_ISL_467485	Molecular Diagnostics Services (MDS)	KRISP, KZN Research Innovation and Sequencing Platform	2020-05-13	Giandhari J et al B
SouthAfrica/KRISP-0134/2020	EPI_ISL_467486	Molecular Diagnostics Services (MDS)	KRISP, KZN Research Innovation and Sequencing Platform	2020-05-14	Giandhari J et al B
SouthAfrica/KRISP-0136/2020	EPI_ISL_467488	Molecular Diagnostics Services (MDS)	KRISP, KZN Research Innovation and Sequencing Platform	2020-05-14	Giandhari J et al B
SouthAfrica/KRISP-0137/2020	EPI_ISL_467489	Molecular Diagnostics Services (MDS)	KRISP, KZN Research Innovation and Sequencing Platform	2020-05-14	Giandhari J et al B
SouthAfrica/KRISP-0138/2020	EPI_ISL_467490	Molecular Diagnostics Services (MDS)	KRISP, KZN Research Innovation and Sequencing Platform	2020-05-14	Giandhari J et al B
SouthAfrica/KRISP-0139/2020	EPI_ISL_467491	Molecular Diagnostics Services (MDS)	KRISP, KZN Research Innovation and Sequencing Platform	2020-05-16	Giandhari J et al B
SouthAfrica/KRISP-0141/2020	EPI_ISL_467492	NHLS-IALCH	KRISP, KZN Research Innovation and Sequencing Platform	2020-05-21	Giandhari J et al B
SouthAfrica/KRISP-0143/2020	EPI_ISL_467493	NHLS-IALCH	KRISP, KZN Research Innovation and Sequencing Platform	2020-05-21	Giandhari J et al B
SouthAfrica/KRISP-0144/2020	EPI_ISL_467494	Molecular Diagnostics Services (MDS)	KRISP, KZN Research Innovation and Sequencing Platform	2020-05-30	Giandhari J et al B
SouthAfrica/KRISP-0145/2020	EPI_ISL_467495	Molecular Diagnostics Services (MDS)	KRISP, KZN Research Innovation and Sequencing Platform	2020-05-30	Giandhari J et al B
SouthAfrica/KRISP-0147/2020	EPI_ISL_467497	Molecular Diagnostics Services (MDS)	KRISP, KZN Research Innovation and Sequencing Platform	2020-05-30	Giandhari J et al B
SouthAfrica/KRISP-0148/2020	EPI_ISL_467498	Molecular Diagnostics Services (MDS)	KRISP, KZN Research Innovation and Sequencing Platform	2020-05-30	Giandhari J et al B
SouthAfrica/KRISP-0150/2020	EPI_ISL_467500	Molecular Diagnostics Services (MDS)	KRISP, KZN Research Innovation and Sequencing Platform	2020-05-30	Giandhari J et al B
SouthAfrica/KRISP-0151/2020	EPI_ISL_467501	Molecular Diagnostics Services (MDS)	KRISP, KZN Research Innovation and Sequencing Platform	2020-06-01	Giandhari J et al B
SouthAfrica/KRISP-0152/2020	EPI_ISL_467502	Molecular Diagnostics Services (MDS)	KRISP, KZN Research Innovation and Sequencing Platform	2020-06-01	Giandhari J et al B
SouthAfrica/KRISP-0153/2020	EPI_ISL_467503	Molecular Diagnostics Services (MDS)	KRISP, KZN Research Innovation and Sequencing Platform	2020-06-01	Giandhari J et al B
SouthAfrica/KRISP-0154/2020	EPI_ISL_467504	Molecular Diagnostics Services (MDS)	KRISP, KZN Research Innovation and Sequencing Platform	2020-06-01	Giandhari J et al B
SouthAfrica/KRISP-0155/2020	EPI_ISL_467505	Molecular Diagnostics Services (MDS)	KRISP, KZN Research Innovation and Sequencing Platform	2020-06-01	Giandhari J et al B

SouthAfrica/KRISP-0156/2020	EPI_ISL_467506	Molecular Diagnostics Services (MDS)	KRISP, KZN Research Innovation and Sequencing Platform	2020-06-01	Giandhari J et al B
SouthAfrica/KRISP-0157/2020	EPI_ISL_467507	NHLS-IALCH	KRISP, KZN Research Innovation and Sequencing Platform	2020-05-25	Giandhari J et al B
SouthAfrica/KRISP-0158/2020	EPI_ISL_467508	NHLS-IALCH	KRISP, KZN Research Innovation and Sequencing Platform	2020-05-28	Giandhari J et al B
SouthAfrica/KRISP-0159/2020	EPI_ISL_467509	NHLS-IALCH	KRISP, KZN Research Innovation and Sequencing Platform	2020-06-01	Giandhari J et al B
SouthAfrica/KRISP-0160/2020	EPI_ISL_467510	NHLS-IALCH	KRISP, KZN Research Innovation and Sequencing Platform	2020-06-01	Giandhari J et al B
SouthAfrica/KRISP-0161/2020	EPI_ISL_467511	NHLS-IALCH	KRISP, KZN Research Innovation and Sequencing Platform	2020-06-01	Giandhari J et al B
SouthAfrica/KRISP-0162/2020	EPI_ISL_467512	NHLS-IALCH	KRISP, KZN Research Innovation and Sequencing Platform	2020-06-01	Giandhari J et al B
SouthAfrica/KRISP-0163/2020	EPI_ISL_467513	NHLS-IALCH	KRISP, KZN Research Innovation and Sequencing Platform	2020-06-01	Giandhari J et al B
SouthAfrica/KRISP-0164/2020	EPI_ISL_467514	NHLS-IALCH	KRISP, KZN Research Innovation and Sequencing Platform	2020-06-01	Giandhari J et al B
SouthAfrica/KRISP-0165/2020	EPI_ISL_467515	NHLS-IALCH	KRISP, KZN Research Innovation and Sequencing Platform	2020-05-29	Giandhari J et al B
SouthAfrica/KRISP-0167/2020	EPI_ISL_467517	NHLS-IALCH	KRISP, KZN Research Innovation and Sequencing Platform	2020-06-02	Giandhari J et al B
SouthAfrica/KRISP-0169/2020	EPI_ISL_467519	NHLS-IALCH	KRISP, KZN Research Innovation and Sequencing Platform	2020-05-31	Giandhari J et al B
SouthAfrica/KRISP-0170/2020	EPI_ISL_467520	NHLS-IALCH	KRISP, KZN Research Innovation and Sequencing Platform	2020-05-31	Giandhari J et al B
SouthAfrica/KRISP-0171/2020	EPI_ISL_467521	NHLS-IALCH	KRISP, KZN Research Innovation and Sequencing Platform	2020-06-02	Giandhari J et al B
SouthAfrica/KRISP-0172/2020	EPI_ISL_467522	NHLS-IALCH	KRISP, KZN Research Innovation and Sequencing Platform	2020-06-02	Giandhari J et al B
SouthAfrica/KRISP-0173/2020	EPI_ISL_467523	NHLS-IALCH	KRISP, KZN Research Innovation and Sequencing Platform	2020-06-02	Giandhari J et al B
SouthAfrica/KRISP-0174/2020	EPI_ISL_467524	NHLS-IALCH	KRISP, KZN Research Innovation and Sequencing Platform	2020-06-04	Giandhari J et al B
SouthAfrica/KRISP-04/2020	EPI_ISL_436684	KRISP, KZN Research Innovation and Sequencing Platform	KRISP, KZN Research Innovation and Sequencing Platform	2020-03-31	Giandhari J et al A (https://dx.doi.org/10.1101/2020.05.29.20116376)
SouthAfrica/KRISP-045/2020	EPI_ISL_436686	KRISP, KZN Research Innovation and Sequencing Platform	KRISP, KZN Research Innovation and Sequencing Platform	2020-03-27	Giandhari J et al A (https://dx.doi.org/10.1101/2020.05.29.20116376)
SouthAfrica/KRISP-051/2020	EPI_ISL_436687	KRISP, KZN Research Innovation and Sequencing Platform	KRISP, KZN Research Innovation and Sequencing Platform	2020-03-28	Giandhari J et al A (https://dx.doi.org/10.1101/2020.05.29.20116376)
SouthAfrica/KRISP-06/2020	EPI_ISL_421573	Molecular Diagnostic Services	KRISP, KZN Research Innovation and Sequencing Platform	2020-03-31	Giandhari J et al A (https://dx.doi.org/10.1101/2020.05.29.20116376)
SouthAfrica/KRISP-07/2020	EPI_ISL_421574	Molecular Diagnostic Services	KRISP, KZN Research Innovation and Sequencing Platform	2020-04-01	Giandhari J et al A (https://dx.doi.org/10.1101/2020.05.29.20116376)
SouthAfrica/KRISP-101/2020	EPI_ISL_455631	KRISP, KZN Research Innovation and Sequencing Platform	KRISP, KZN Research Innovation and Sequencing Platform	2020-04-30	Giandhari J et al A (https://dx.doi.org/10.1101/2020.05.29.20116376)
SouthAfrica/KRISP-102/2020	EPI_ISL_455632	KRISP, KZN Research Innovation and Sequencing Platform	KRISP, KZN Research Innovation and Sequencing Platform	2020-04-30	Giandhari J et al A (https://dx.doi.org/10.1101/2020.05.29.20116376)
SouthAfrica/KRISP-104/2020	EPI_ISL_455634	KRISP, KZN Research Innovation and Sequencing Platform	KRISP, KZN Research Innovation and Sequencing Platform	2020-05-01	Giandhari J et al A (https://dx.doi.org/10.1101/2020.05.29.20116376)
SouthAfrica/KRISP-106/2020	EPI_ISL_455636	KRISP, KZN Research Innovation and Sequencing Platform	KRISP, KZN Research Innovation and Sequencing Platform	2020-03-31	Giandhari J et al A (https://dx.doi.org/10.1101/2020.05.29.20116376)
SouthAfrica/KRISP-107/2020	EPI_ISL_455637	KRISP, KZN Research Innovation and Sequencing Platform	KRISP, KZN Research Innovation and Sequencing Platform	2020-05-04	Giandhari J et al A (https://dx.doi.org/10.1101/2020.05.29.20116376)
SouthAfrica/KRISP-108/2020	EPI_ISL_455638	KRISP, KZN Research Innovation and Sequencing Platform	KRISP, KZN Research Innovation and Sequencing Platform	2020-05-04	Giandhari J et al A (https://dx.doi.org/10.1101/2020.05.29.20116376)
SouthAfrica/KRISP-109/2020	EPI_ISL_455639	KRISP, KZN Research Innovation and Sequencing Platform	KRISP, KZN Research Innovation and Sequencing Platform	2020-03-31	Giandhari J et al A (https://dx.doi.org/10.1101/2020.05.29.20116376)
SouthAfrica/R02827/2020	EPI_ISL_430297	National Institute for Communicable Diseases of the National Health Laboratory Service	National Institute for Communicable Diseases of the National Health Laboratory Service	2020-03-06	Allam M et al A
SouthAfrica/R03006/2020	EPI_ISL_417186	National Institute for Communicable Diseases of the National Health Laboratory Service	National Institute for Communicable Diseases of the National Health Laboratory Service	2020-03-07	Allam M et al B (https://dx.doi.org/10.1101/2020.05.29.20116376)
SouthAfrica/R04827/2020	EPI_ISL_450296	National Institute for Communicable Diseases of the National Health Laboratory Service	National Institute for Communicable Diseases of the National Health Laboratory Service	2020-03-17	Allam M et al A
SouthAfrica/R05204/2020	EPI_ISL_450297	National Institute for Communicable Diseases of the National Health Laboratory Service	National Institute for Communicable Diseases of the National Health Laboratory Service	2020-03-19	Allam M et al A
SouthAfrica/R05360/2020	EPI_ISL_450298	National Institute for Communicable Diseases of the National Health Laboratory Service	National Institute for Communicable Diseases of the National Health Laboratory Service	2020-03-19	Allam M et al A
SouthAfrica/R05929/2020	EPI_ISL_450495	National Institute for Communicable Diseases of the National Health Laboratory Service	National Institute for Communicable Diseases of the National Health Laboratory Service	2020-03-25	Allam M et al A
SouthAfrica/R07552/2020	EPI_ISL_450299	National Institute for Communicable Diseases of the National Health Laboratory Service	National Institute for Communicable Diseases of the National Health Laboratory Service	2020-03-31	Allam M et al A
SouthAfrica/R07601/2020	EPI_ISL_450300	National Institute for Communicable Diseases of the National Health Laboratory Service	National Institute for Communicable Diseases of the National Health Laboratory Service	2020-03-30	Allam M et al A
SouthAfrica/R09126/2020	EPI_ISL_470880	National Institute for Communicable Diseases of the National Health Laboratory Service	National Institute for Communicable Diseases of the National Health Laboratory Service	2020-04-28	Allam M et al A
SouthKorea/KCDC12/2020	EPI_ISL_412872	Division of Viral Diseases, Center for Laboratory Control of Infectious Diseases, Korea Centers for Diseases Control and Prevention	Division of Viral Diseases, Center for Laboratory Control of Infectious Diseases, Korea Centers for Diseases Control and Prevention	2020-02-01	Jeong-Min Kim et al A
SouthKorea/KCDC24/2020	EPI_ISL_412873	Division of Viral Diseases, Center for Laboratory Control of Infectious Diseases, Korea Centers for Diseases Control and Prevention	Division of Viral Diseases, Center for Laboratory Control of Infectious Diseases, Korea Centers for Diseases Control and Prevention	2020-02-06	Jeong-Min Kim et al A
SouthKorea/KUMC01/2020	EPI_ISL_413017	Department of Microbiology, Institute for Viral Diseases, College of Medicine, Korea University	Department of Microbiology, Institute for Viral Diseases, College of Medicine, Korea University	2020-02-06	Changmin Kang et al
SouthKorea/KUMC02/2020	EPI_ISL_413018	Department of Microbiology, Institute for Viral Diseases, College of Medicine, Korea University	Department of Microbiology, Institute for Viral Diseases, College of Medicine, Korea University	2020-02-06	Changmin Kang et al
Spain/100_20/2020	EPI_ISL_463000		Molecular Genetics	2020-05-30	Gomez et al
Spain/67_20/2020	EPI_ISL_462995		Molecular Genetics	2020-05-30	Gomez et al
Spain/68_19/2020	EPI_ISL_462996		Molecular Genetics	2020-05-30	Gomez et al
Spain/80_19/2020	EPI_ISL_462997		Molecular Genetics	2020-05-30	Gomez et al
Spain/86_17/2020	EPI_ISL_462998		Molecular Genetics	2020-05-30	Gomez et al
Spain/96_19/2020	EPI_ISL_462999		Molecular Genetics	2020-05-30	Gomez et al
Spain/ACoruna_001/2020	EPI_ISL_468331	Microbiology Service, University Hospital of A Coruna-Biomedical Research Institute	Genomes & Disease, Center for Research in Molecular Medicine and Chronic Diseases, University of Santiago de Compostela	2020-05-11	Kelly Conde et al
Spain/ACoruna_002/2020	EPI_ISL_468332	Microbiology Service, University Hospital of A Coruna-Biomedical Research Institute	Genomes & Disease, Center for Research in Molecular Medicine and Chronic Diseases, University of Santiago de Compostela	2020-05-11	Kelly Conde et al
Spain/ACoruna_006/2020	EPI_ISL_468336	Microbiology Service, University Hospital of A Coruna-Biomedical Research Institute	Genomes & Disease, Center for Research in Molecular Medicine and Chronic Diseases, University of Santiago de Compostela	2020-05-10	Kelly Conde et al
Spain/ACoruna_008/2020	EPI_ISL_468338	Microbiology Service, University Hospital of A Coruna-Biomedical Research Institute	Genomes & Disease, Center for Research in Molecular Medicine and Chronic Diseases, University of Santiago de Compostela	2020-05-10	Kelly Conde et al
Spain/ACoruna_011/2020	EPI_ISL_468341	Microbiology Service, University Hospital of A Coruna-Biomedical Research Institute	Genomes & Disease, Center for Research in Molecular Medicine and Chronic Diseases, University of Santiago de Compostela	2020-05-12	Kelly Conde et al
Spain/ACoruna_012/2020	EPI_ISL_468342	Microbiology Service, University Hospital of A Coruna-Biomedical Research Institute	Genomes & Disease, Center for Research in Molecular Medicine and Chronic Diseases, University of Santiago de Compostela	2020-05-12	Kelly Conde et al
Spain/ACoruna_013/2020	EPI_ISL_468343	Microbiology Service, University Hospital of A Coruna-Biomedical Research Institute	Genomes & Disease, Center for Research in Molecular Medicine and Chronic Diseases, University of Santiago de Compostela	2020-05-29	Kelly Conde et al
Spain/ACoruna_014/2020	EPI_ISL_468344	Microbiology Service, University Hospital of A Coruna-Biomedical Research Institute	Genomes & Disease, Center for Research in Molecular Medicine and Chronic Diseases, University of Santiago de Compostela	2020-05-12	Kelly Conde et al
Spain/Almassera633/2020	EPI_ISL_455722	Servicio de Microbiologia. Hospital Clinico Universitario de Valencia	Sequencing and Bioinformatics Service and Molecular Epidemiology Research Group. FISABIO-Public Health, and SeqCOVID-Spain Consortium	2020-03-30	Giuseppe 'Auria et al
Spain/Canarias201939/2020	EPI_ISL_455326	Hospital Universitario Insular de Gran Canaria	Instituto de Salud Carlos III	2020-02-29	Iglesias-Caballero et al B
Spain/CastillaLaMancha201328/2020	EPI_ISL_418245	Hospital General y Universitario de Guadalajara	Instituto de Salud Carlos III	2020-03-01	Iglesias-Caballero et al A (https://dx.doi.org/10.1101/2020.04.20.050039)
Spain/CastillayLeon201061/2020	EPI_ISL_418247	HOSPITAL GENERAL DE SEGOVIA	Instituto de Salud Carlos III	2020-02-26	Iglesias-Caballero et al A (https://dx.doi.org/10.1101/2020.04.20.050039)
Spain/CastillayLeon201372/2020	EPI_ISL_418249	COMPLEJO ASISTENCIAL UNIVERSITARIO DE BURGOS	Instituto de Salud Carlos III	2020-03-03	Iglesias-Caballero et al A (https://dx.doi.org/10.1101/2020.04.20.050039)
Spain/COV000692/2020	EPI_ISL_452772	Hospital Universitario Araba. Vitoria-Gasteiz,	SeqCOVID-SPAIN consortium/IBV(CSIC)	2020-02-29	Silvia Hernáez Crespo et al
Spain/COV000716/2020	EPI_ISL_452752	Hospital Universitario Araba. Vitoria-Gasteiz,	SeqCOVID-SPAIN consortium/IBV(CSIC)	2020-03-05	Silvia Hernáez Crespo et al
Spain/COV000758/2020	EPI_ISL_452733	Hospital Universitario Araba. Vitoria-Gasteiz,	SeqCOVID-SPAIN consortium/IBV(CSIC)	2020-03-06	Silvia Hernáez Crespo et al
Spain/COV000822/2020	EPI_ISL_452710	Hospital Universitario Araba. Vitoria-Gasteiz,	SeqCOVID-SPAIN consortium/IBV(CSIC)	2020-03-10	Silvia Hernáez Crespo et al
Spain/COV000860/2020	EPI_ISL_452692	Hospital Universitario Araba. Vitoria-Gasteiz,	SeqCOVID-SPAIN consortium/IBV(CSIC)	2020-02-29	Silvia Hernáez Crespo et al
Spain/COV001056/2020	EPI_ISL_452616	Servicio de Microbiología y Parasitología clínica. UCEIMP. Hospital Universitario Virgen del Rocío/IBIS/CSIC/US.	SeqCOVID-SPAIN consortium/IBV(CSIC)	2020-04-01	Guillermo Martín Gutiérrez et al
Spain/COV001058/2020	EPI_ISL_452614	Servicio de Microbiología y Parasitología clínica. UCEIMP. Hospital Universitario Virgen del Rocío/IBIS/CSIC/US.	SeqCOVID-SPAIN consortium/IBV(CSIC)	2020-04-01	Guillermo Martín Gutiérrez et al
Spain/COV001066/2020	EPI_ISL_452610	Servicio de Microbiología y Parasitología clínica. UCEIMP. Hospital Universitario Virgen del Rocío/IBIS/CSIC/US.	SeqCOVID-SPAIN consortium/IBV(CSIC)	2020-04-01	Guillermo Martín Gutiérrez et al
Spain/COV001384/2020	EPI_ISL_452517	Clínica Universidad de Navarra. Servicio de Enfermedades Infecciosas y Microbiología clínica	SeqCOVID-SPAIN consortium/IBV(CSIC)	2020-03-15	Mirian Fernández-Alonso et al
Spain/COV001477/2020	EPI_ISL_467128	Hospital Universitario Araba. Vitoria-Gasteiz	SeqCOVID-SPAIN consortium/IBV(CSIC)	2020-03-18	Silvia Hernáez Crespo et al
Spain/COV001525/2020	EPI_ISL_467167	Hospital Universitario Araba. Vitoria-Gasteiz	SeqCOVID-SPAIN consortium/IBV(CSIC)	2020-03-17	Silvia Hernáez Crespo et al
Spain/COV001528/2020	EPI_ISL_467159	Hospital Universitario Araba. Vitoria-Gasteiz	SeqCOVID-SPAIN consortium/IBV(CSIC)	2020-03-13	Silvia Hernáez Crespo et al
Spain/COV001544/2020	EPI_ISL_467182	Hospital Universitario Araba. Vitoria-Gasteiz	SeqCOVID-SPAIN consortium/IBV(CSIC)	2020-03-16	Silvia Hernáez Crespo et al
Spain/COV001585/2020	EPI_ISL_467136	Hospital Universitario Araba. Vitoria-Gasteiz	SeqCOVID-SPAIN consortium/IBV(CSIC)	2020-03-13	Silvia Hernáez Crespo et al
Spain/COV001889/2020	EPI_ISL_452459	Hospital Universitario Puerta del Mar de Cádiz - INIBICA	SeqCOVID-SPAIN consortium/IBV(CSIC)	2020-03-13	Salud Rodríguez-Pallares et al
Spain/COV001924/2020	EPI_ISL_452450	Servicio de Microbiología. HRU de Málaga. Servicio Andaluz de Salud	SeqCOVID-SPAIN consortium/IBV(CSIC)	2020-03-06	Inmaculada de Toro Peinado et al
Spain/COV001935/2020	EPI_ISL_452441	Servicio de Microbiología. HRU de Málaga. Servicio Andaluz de Salud	SeqCOVID-SPAIN consortium/IBV(CSIC)	2020-03-12	Inmaculada de Toro Peinado et al
Spain/COV001972/2020	EPI_ISL_452410	Servicio de Microbiología. HRU de Málaga. Servicio Andaluz de Salud	SeqCOVID-SPAIN consortium/IBV(CSIC)	2020-03-14	Inmaculada de Toro Peinado et al
Spain/COV002726/2020	EPI_ISL_467266	Hospital Clínico Universitario de Santiago de Compostela	SeqCOVID-SPAIN consortium/IBV(CSIC)	2020-04-15	José Javier Costa Alcalde et al
Spain/COV002733/2020	EPI_ISL_467289	Hospital Clínico Universitario de Santiago de Compostela	SeqCOVID-SPAIN consortium/IBV(CSIC)	2020-04-13	José Javier Costa Alcalde et al
Spain/COV002738/2020	EPI_ISL_467291	Hospital Clínico Universitario de Santiago de Compostela	SeqCOVID-SPAIN consortium/IBV(CSIC)	2020-04-13	José Javier Costa Alcalde et al
Spain/COV002740/2020	EPI_ISL_467280	Hospital Clínico Universitario de Santiago de Compostela	SeqCOVID-SPAIN consortium/IBV(CSIC)	2020-04-15	José Javier Costa Alcalde et al
Spain/COV002753/2020	EPI_ISL_467277	Hospital Clínico Universitario de Santiago de Compostela	SeqCOVID-SPAIN consortium/IBV(CSIC)	2020-04-13	José Javier Costa Alcalde et al
Spain/COV003647/2020	EPI_ISL_467187	Hospital General Universitario Gregorio Marañón	SeqCOVID-SPAIN consortium/IBV(CSIC)	2020-04-01	Laura Pérez-Lago et al
Spain/COV003660/2020	EPI_ISL_467222	Hospital General Universitario Gregorio Marañón	SeqCOVID-SPAIN consortium/IBV(CSIC)	2020-04-01	Laura Pérez-Lago et al
Spain/COV003665/2020	EPI_ISL_467215	Hospital General Universitario Gregorio Marañón	SeqCOVID-SPAIN consortium/IBV(CSIC)	2020-04-01	Laura Pérez-Lago et al
Spain/COV003666/2020	EPI_ISL_467232	Hospital General Universitario Gregorio Marañón	SeqCOVID-SPAIN consortium/IBV(CSIC)	2020-04-01	Laura Pérez-Lago et al
Spain/COV003680/2020	EPI_ISL_467192	Hospital General Universitario Gregorio Marañón	SeqCOVID-SPAIN consortium/IBV(CSIC)	2020-04-02	Laura Pérez-Lago et al
Spain/COV003692/2020	EPI_ISL_467252	Hospital General Universitario Gregorio Marañón	SeqCOVID-SPAIN consortium/IBV(CSIC)	2020-04-02	Laura Pérez-Lago et al
Spain/COV003699/2020	EPI_ISL_467231	Hospital General Universitario Gregorio Marañón	SeqCOVID-SPAIN consortium/IBV(CSIC)	2020-04-02	Laura Pérez-Lago et al
Spain/COV003712/2020	EPI_ISL_467220	Hospital General Universitario Gregorio Marañón	SeqCOVID-SPAIN consortium/IBV(CSIC)	2020-04-02	Laura Pérez-Lago et al
Spain/COV003722/2020	EPI_ISL_467190	Hospital General Universitario Gregorio Marañón	SeqCOVID-SPAIN consortium/IBV(CSIC)	2020-05-07	Laura Pérez-Lago et al
Spain/COV003723/2020	EPI_ISL_467234	Hospital General Universitario Gregorio Marañón	SeqCOVID-SPAIN consortium/IBV(CSIC)	2020-05-07	Laura Pérez-Lago et al
Spain/COV003724/2020	EPI_ISL_467195	Hospital General Universitario Gregorio Marañón	SeqCOVID-SPAIN consortium/IBV(CSIC)	2020-05-10	Laura Pérez-Lago et al
Spain/COV003725/2020	EPI_ISL_467246	Hospital General Universitario Gregorio Marañón	SeqCOVID-SPAIN consortium/IBV(CSIC)	2020-05-10	Laura Pérez-Lago et al
Spain/Donostia-San_Sebastian2077/2020	EPI_ISL_468872	Servicio de Microbiología. Hospital Universitario Donostia. OSI Donostialdea. Área de Enfermedades Infecciosas, Grupo de Infección Respiratoria y Resistencia Antimicrobiana. Instituto de Investigación Sanitaria Biodonostia.	SeqCOVID-SPAIN consortium/IBV(CSIC)	2020-04-10	Gustavo Cilla et al
Spain/Donostia-San_Sebastian2098/2020	EPI_ISL_468902	Servicio de Microbiología. Hospital Universitario Donostia. OSI Donostialdea. Área de Enfermedades Infecciosas, Grupo de Infección Respiratoria y Resistencia Antimicrobiana. Instituto de Investigación Sanitaria Biodonostia.	SeqCOVID-SPAIN consortium/IBV(CSIC)	2020-04-11	Gustavo Cilla et al
Spain/Donostia-San_Sebastian2101/2020	EPI_ISL_468883	Servicio de Microbiología. Hospital Universitario Donostia. OSI Donostialdea. Área de Enfermedades Infecciosas, Grupo de Infección Respiratoria y Resistencia Antimicrobiana. Instituto de Investigación Sanitaria Biodonostia.	SeqCOVID-SPAIN consortium/IBV(CSIC)	2020-04-11	Gustavo Cilla et al
Spain/Donostia-San_Sebastian2141/2020	EPI_ISL_468866	Servicio de Microbiología. Hospital Universitario Donostia. OSI Donostialdea. Área de Enfermedades Infecciosas, Grupo de Infección Respiratoria y Resistencia Antimicrobiana. Instituto de Investigación Sanitaria Biodonostia.	SeqCOVID-SPAIN consortium/IBV(CSIC)	2020-04-12	Gustavo Cilla et al
Spain/Donostia-San_Sebastian2145/2020	EPI_ISL_468941	Servicio de Microbiología. Hospital Universitario Donostia. OSI Donostialdea. Área de Enfermedades Infecciosas, Grupo de Infección Respiratoria y Resistencia Antimicrobiana. Instituto de Investigación Sanitaria Biodonostia.	SeqCOVID-SPAIN consortium/IBV(CSIC)	2020-04-12	Gustavo Cilla et al
Spain/Donostia-San_Sebastian2146/2020	EPI_ISL_468870	Servicio de Microbiología. Hospital Universitario Donostia. OSI Donostialdea. Área de Enfermedades Infecciosas, Grupo de Infección Respiratoria y Resistencia Antimicrobiana. Instituto de Investigación Sanitaria Biodonostia.	SeqCOVID-SPAIN consortium/IBV(CSIC)	2020-04-12	Gustavo Cilla et al

Spain/Donostia-San_Sebastian2147/2020	EPI_ISL_468928	Servicio de Microbiología. Hospital Universitario Donostia. OSI Donostialdea. Área de Enfermedades Infecciosas, Grupo de Infección Respiratoria y Resistencia Antimicrobiana. Instituto de Investigación Sanitaria Biodonostia.	SeqCOVID-SPAIN consortium/IBV(CSIC)	2020-04-12	Gustavo Cilla et al
Spain/Donostia-San_Sebastian2149/2020	EPI_ISL_468913	Servicio de Microbiología. Hospital Universitario Donostia. OSI Donostialdea. Área de Enfermedades Infecciosas, Grupo de Infección Respiratoria y Resistencia Antimicrobiana. Instituto de Investigación Sanitaria Biodonostia.	SeqCOVID-SPAIN consortium/IBV(CSIC)	2020-04-12	Gustavo Cilla et al
Spain/Donostia-San_Sebastian2152/2020	EPI_ISL_468895	Servicio de Microbiología. Hospital Universitario Donostia. OSI Donostialdea. Área de Enfermedades Infecciosas, Grupo de Infección Respiratoria y Resistencia Antimicrobiana. Instituto de Investigación Sanitaria Biodonostia.	SeqCOVID-SPAIN consortium/IBV(CSIC)	2020-04-12	Gustavo Cilla et al
Spain/Donostia-San_Sebastian2169/2020	EPI_ISL_468875	Servicio de Microbiología. Hospital Universitario Donostia. OSI Donostialdea. Área de Enfermedades Infecciosas, Grupo de Infección Respiratoria y Resistencia Antimicrobiana. Instituto de Investigación Sanitaria Biodonostia.	SeqCOVID-SPAIN consortium/IBV(CSIC)	2020-04-12	Gustavo Cilla et al
Spain/LaRioja201277/2020	EPI_ISL_455336	Hospital San Pedro	Instituto de Salud Carlos III	2020-02-29	Iglesias-Caballero et al B
Spain/Madrid_H12_3203/2020	EPI_ISL_428702	Hospital Universitario 12 de Octubre	Hospital Universitario 12 de Octubre	2020-03-11	Esther Viedma et al
Spain/Madrid201105/2020	EPI_ISL_418251	HOSPITAL UNIVERSITARIO LA PAZ	Instituto de Salud Carlos III	2020-02-25	Iglesias-Caballero et al A (https://dx.doi.org/10.1101/2020.04.20.050039)
Spain/PaisVasco201388/2020	EPI_ISL_455351	Hospital Txagorritxu	Instituto de Salud Carlos III	2020-02-29	Iglesias-Caballero et al B
Spain/Palma2583/2020	EPI_ISL_468957	Servicio de Microbiología, Hospital Universitario Son Espases	SeqCOVID-SPAIN consortium/IBV(CSIC)	2020-04-15	Carla López-Causapé et al
Spain/Palma2588/2020	EPI_ISL_468995	Servicio de Microbiología, Hospital Universitario Son Espases	SeqCOVID-SPAIN consortium/IBV(CSIC)	2020-04-13	Carla López-Causapé et al
Spain/Valencia174/2020	EPI_ISL_436269	Servicio de Microbiologia. Hospital Universitario Doctor Peset	Sequencing and Bioinformatics Service and Molecular Epidemiology Research Group. FISABIO-Public Health	2020-03-20	Juan Alberola Enguídanos et al
Spain/Valencia214/2020	EPI_ISL_436309	Servicio de Microbiologia. Hospital Clinico Universitario de Valencia	Sequencing and Bioinformatics Service and Molecular Epidemiology Research Group. FISABIO-Public Health	2020-03-28	Vicente Soriano Chirona et al
Spain/Valencia23/2020	EPI_ISL_419687	Servicio de Microbiología. Consorcio Hospital General Universitario de Valencia	Sequencing and Bioinformatics Service and Molecular Epidemiology Research Group. FISABIO-Public Health	2020-02-27	Giuseppe D'Auria et al
Spain/Valencia25/2020	EPI_ISL_419689	Servicio de Microbiología. Consorcio Hospital General Universitario de Valencia	Sequencing and Bioinformatics Service and Molecular Epidemiology Research Group. FISABIO-Public Health	2020-02-27	Neris Garcia-Gonzalez et al
Spain/Valencia266/2020	EPI_ISL_436361	Servicio de Microbiología. Consorcio Hospital General Universitario de Valencia	Sequencing and Bioinformatics Service and Molecular Epidemiology Research Group. FISABIO-Public Health	2020-02-29	Neris Garcia-Gonzalez et al
Spain/Valencia269/2020	EPI_ISL_436364	Servicio de Microbiologia. Hospital Clinico Universitario de Valencia	Sequencing and Bioinformatics Service and Molecular Epidemiology Research Group. FISABIO-Public Health	2020-03-21	Beatriz Beamud et al
Spain/Valencia274/2020	EPI_ISL_436369	Servicio de Microbiologia. Hospital Clinico Universitario de Valencia	Sequencing and Bioinformatics Service and Molecular Epidemiology Research Group. FISABIO-Public Health	2020-03-26	Sandra Carbo et al
Spain/Valencia313/2020	EPI_ISL_436408	Servicio de Microbiologia. Hospital Clinico Universitario de Valencia	Sequencing and Bioinformatics Service and Molecular Epidemiology Research Group. FISABIO-Public Health	2020-03-25	Neris Garcia-Gonzalez et al
Spain/Valencia4/2020	EPI_ISL_416483	Servicio de Microbiología. Consorcio Hospital General Universitario de Valencia	Sequencing and Bioinformatics Service and Molecular Epidemiology Research Group. FISABIO-Public Health	2020-02-26	Maria Alma Bracho et al
Spain/Valencia5/2020	EPI_ISL_416484	Servicio de Microbiología. Consorcio Hospital General Universitario de Valencia	Sequencing and Bioinformatics Service and Molecular Epidemiology Research Group. FISABIO-Public Health	2020-02-27	Maria Dolores Ocete et al (https://dx.doi.org/10.1101/2020.04.20.050039)
Spain/Valencia50/2020	EPI_ISL_420131	Servicio de Microbiologia. Hospital Clinico Universitario de Valencia	Sequencing and Bioinformatics Service and Molecular Epidemiology Research Group. FISABIO-Public Health	2020-03-18	Paula Ruiz-Hueso et al
Spain/Valencia54/2020	EPI_ISL_425178	Servicio de Microbiología. Consorcio Hospital General Universitario de Valencia	Sequencing and Bioinformatics Service and Molecular Epidemiology Research Group. FISABIO-Public Health	2020-02-28	David Navarro et al
Spain/Valencia565/2020	EPI_ISL_447497	Servicio de Microbiologia. Hospital Clinico Universitario de Valencia	Sequencing and Bioinformatics Service and Molecular Epidemiology Research Group. FISABIO-Public Health	2020-03-27	Llúcia Martínez-Priego et al
Spain/Valencia597/2020	EPI_ISL_447519	Servicio de Microbiologia. Hospital Clinico Universitario de Valencia	Sequencing and Bioinformatics Service and Molecular Epidemiology Research Group. FISABIO-Public Health	2020-04-01	Sandra Carbo et al
Spain/Valencia6/2020	EPI_ISL_416485	Servicio de Microbiologia. Consorcio Hospital General Universitario de Valencia	Sequencing and Bioinformatics Service and Molecular Epidemiology Research Group. FISABIO-Public Health	2020-02-27	Griselda De Marco et al (https://dx.doi.org/10.1101/2020.04.20.050039)
Spain/Valencia605/2020	EPI_ISL_447524	Servicio de Microbiologia. Hospital Clinico Universitario de Valencia	Sequencing and Bioinformatics Service and Molecular Epidemiology Research Group. FISABIO-Public Health	2020-04-01	Llúcia Martínez-Priego et al
Spain/Valencia66/2020	EPI_ISL_425190	Servicio de Microbiología. Consorcio Hospital General Universitario de Valencia	Sequencing and Bioinformatics Service and Molecular Epidemiology Research Group. FISABIO-Public Health	2020-03-16	Vicente Soriano Chirona et al
Spain/Valencia88/2020	EPI_ISL_425212	Servicio de Microbiología. Consorcio Hospital General Universitario de Valencia	Sequencing and Bioinformatics Service and Molecular Epidemiology Research Group. FISABIO-Public Health	2020-03-11	David Navarro et al
Spain/Zaragoza2406/2020	EPI_ISL_468806	Servicio de Microbiología, Hospital Miguel Servet, Zaragoza	SeqCOVID-SPAIN consortium/IBV(CSIC)	2020-03-21	Antonio Rezusta López et al
SriLanka/COV38/2020	EPI_ISL_428670	Centre for Dengue Research	Centre for Dengue Research	2020-03-16	Chandima Jeewandara et al
SriLanka/COV53/2020	EPI_ISL_428671	Centre for Dengue Research	Centre for Dengue Research	2020-03-10	Chandima Jeewandara et al
SriLanka/COV91/2020	EPI_ISL_428672	Centre for Dengue Research	Centre for Dengue Research	2020-03-19	Chandima Jeewandara et al
Sweden/01/2020	EPI_ISL_411951		Unit for Laboratory Development and Technology Transfer, Public Health Agency of Sweden	2020-02-07	Bengner et al (https://dx.doi.org/10.2807/1560-7917.ES.2020.25.13.2000305)
Sweden/20-04631/2020	EPI_ISL_430847	HS mikrobiologi virus	The Public Health Agency of Sweden	2020-02-27	Zhibing Yun et al
Sweden/20-05444/2020	EPI_ISL_445221	Wasterlakarna	The Public Health Agency of Sweden	2020-03-11	Frida Ahlfors et al
Sweden/20-05448/2020	EPI_ISL_434643	Uppsala Narakut Aleris	The Public Health Agency of Sweden	2020-03-10	Annika Nilsson et al
Sweden/20-05653/2020	EPI_ISL_434646	Ulltuna Vardcentral	The Public Health Agency of Sweden	2020-03-12	Heidi Lindback et al
Sweden/20-05856/2020	EPI_ISL_434649	Svardsjo VC	The Public Health Agency of Sweden	2020-03-17	Tommy Janers et al
Sweden/20-06468/2020	EPI_ISL_434655	Uppsala Narakut Aleris	The Public Health Agency of Sweden	2020-03-25	Annika Nilsson et al
Sweden/20-06665/2020	EPI_ISL_450808	Saroledens Familjelakare	The Public Health Agency of Sweden	2020-03-31	Katarina Jarbur et al
Sweden/20-06695/2020	EPI_ISL_450809	Kungsholmsdoktorn	The Public Health Agency of Sweden	2020-03-31	Linus Hammar et al
Sweden/20-06814/2020	EPI_ISL_445228	Ulltuna Vardcentral	The Public Health Agency of Sweden	2020-04-01	Heidi Lindback et al
Sweden/20-06817/2020	EPI_ISL_445229	Narhalsan Backa vardcentral	The Public Health Agency of Sweden	2020-04-01	Mats Olsson et al
Sweden/20-06909/2020	EPI_ISL_450811	Knivsta VC	The Public Health Agency of Sweden	2020-04-06	Johanna Carlson et al
Sweden/20-06928/2020	EPI_ISL_434660	Narhalsan Backa vardcentral	The Public Health Agency of Sweden	2020-04-06	Mats Olsson et al
Sweden/20-07010/2020	EPI_ISL_434661	Omtanken Grimmered	The Public Health Agency of Sweden	2020-04-07	Bernd Sengpiel et al
Sweden/20-07025/2020	EPI_ISL_434663	Surbrunns VC	The Public Health Agency of Sweden	2020-04-06	Erik Embring et al
Sweden/20-07034/2020	EPI_ISL_434664	Omtanken Grimmered	The Public Health Agency of Sweden	2020-04-06	Bernd Sengpiel et al
Sweden/20-07041/2020	EPI_ISL_450812	Uppsala Narakut Aleris	The Public Health Agency of Sweden	2020-04-05	Annika Nilsson et al
Sweden/20-07042/2020	EPI_ISL_434665	Uppsala Narakut Aleris	The Public Health Agency of Sweden	2020-04-04	Annika Nilsson et al
Sweden/20-07171/2020	EPI_ISL_434666	Hornefors Halsocentral	The Public Health Agency of Sweden	2020-04-08	Camilla Eiback et al
Sweden/20-07298/2020	EPI_ISL_434667	Ulltuna Vardcentral	The Public Health Agency of Sweden	2020-04-14	Heidi Lindback et al
Sweden/20-07377/2020	EPI_ISL_455101	Jourcentralen	The Public Health Agency of Sweden	2020-04-15	Salvatore Ascione et al
Sweden/20-07389/2020	EPI_ISL_434668	Kungsors VC	The Public Health Agency of Sweden	2020-04-15	Jessica Karlsson et al
Sweden/20-07390/2020	EPI_ISL_434669	Lakargruppen	The Public Health Agency of Sweden	2020-04-15	Boris Klanger et al
Sweden/20-07439/2020	EPI_ISL_434670	Narhalsan Molnlycke, Barn och ungdomsmedicin	The Public Health Agency of Sweden	2020-04-17	Mats Reimer et al
Sweden/20-07440/2020	EPI_ISL_434671	Narhalsan Sjobo vardcentral	The Public Health Agency of Sweden	2020-04-16	Lovisa Hjerten et al
Sweden/20-07586/2020	EPI_ISL_445235	Wasterlakarna	The Public Health Agency of Sweden	2020-04-20	Frida Ahlfors et al
Sweden/20-07607/2020	EPI_ISL_434675	Surbrunns VC	The Public Health Agency of Sweden	2020-04-20	Erik Embring et al
Sweden/20-07651/2020	EPI_ISL_445236	Narhalsan Backa vardcentral	The Public Health Agency of Sweden	2020-04-21	Mats Olsson et al
Sweden/20-07653/2020	EPI_ISL_445237	Narhalsan Molnlycke, Barn och ungdomsmedicin	The Public Health Agency of Sweden	2020-04-21	Mats Reimer et al
Sweden/20-07672/2020	EPI_ISL_445239	Uppsala Narakut Aleris	The Public Health Agency of Sweden	2020-04-19	Annika Nilsson et al
Sweden/20-07737/2020	EPI_ISL_450815	Narhalsan Molnlycke, Barn och ungdomsmedicin	The Public Health Agency of Sweden	2020-04-23	Mats Reimer et al
Sweden/20-07739/2020	EPI_ISL_450816	orestadsklinikens VC	The Public Health Agency of Sweden	2020-04-22	Lisa Kjellberg / Laura Plavitu et al
Sweden/20-07741/2020	EPI_ISL_445241	Ã–restadsklinikens VC	The Public Health Agency of Sweden	2020-04-23	Lisa Kjellberg / Laura Plavitu et al
Sweden/20-07768/2020	EPI_ISL_450818	Aneby VC	The Public Health Agency of Sweden	2020-04-23	Ken Granath et al
Sweden/20-07856/2020	EPI_ISL_452237	Huddinge VC	The Public Health Agency of Sweden	2020-04-23	Anders Johansson et al
Sweden/20-07861/2020	EPI_ISL_450822	Jarpens HC	The Public Health Agency of Sweden	2020-04-23	Gunilla Johansson et al
Sweden/20-08085/2020	EPI_ISL_454459	Karolinska Universitetslaboratoriet	The Public Health Agency of Sweden	2020-03-05	Anna-Malin Linde et al
Sweden/20-08096/2020	EPI_ISL_454468	Karolinska Universitetslaboratoriet	The Public Health Agency of Sweden	2020-03-06	Anna-Malin Linde et al
Sweden/20-08107/2020	EPI_ISL_454478	Karolinska Universitetslaboratoriet	The Public Health Agency of Sweden	2020-03-08	Anna-Malin Linde et al
Sweden/20-08108/2020	EPI_ISL_454479	Karolinska Universitetslaboratoriet	The Public Health Agency of Sweden	2020-03-09	Anna-Malin Linde et al
Sweden/20-08109/2020	EPI_ISL_454480	Karolinska Universitetslaboratoriet	The Public Health Agency of Sweden	2020-03-09	Anna-Malin Linde et al
Sweden/20-08121/2020	EPI_ISL_454491	Karolinska Universitetslaboratoriet	The Public Health Agency of Sweden	2020-03-09	Anna-Malin Linde et al
Sweden/20-08128/2020	EPI_ISL_454443	Halmstad klinisk mikrobiologi	The Public Health Agency of Sweden	2020-03-10	Anna-Malin Linde et al
Sweden/20-08149/2020	EPI_ISL_429120	Klinisk mikrobiologi och vardhygien Halmstad	The Public Health Agency of Sweden	2020-03-11	Arne Kotz et al
Sweden/20-08173/2020	EPI_ISL_429125	Klinisk mikrobiologi och vardhygien Halmstad	The Public Health Agency of Sweden	2020-03-12	Arne Kotz et al
Sweden/20-08320/2020	EPI_ISL_429126	Unilabs Skovde	The Public Health Agency of Sweden	2020-03-21	Tobias Kollberg et al
Sweden/20-08686/2020	EPI_ISL_434642	Laboratoriemedicin	The Public Health Agency of Sweden	2020-03-27	Oskar Karlsson Lindsjo et al
Sweden/20-08717/2020	EPI_ISL_430859	Laboratoriemedicin	The Public Health Agency of Sweden	2020-04-02	Oskar Karlsson Lindsjo et al
Sweden/20-14013/2020	EPI_ISL_455103	Huslakarna Varmbadhuset Varberg	The Public Health Agency of Sweden	2020-04-27	Johanna Hilmersson et al
Sweden/20-14087/2020	EPI_ISL_450826	Uppsala Narakut Aleris	The Public Health Agency of Sweden	2020-04-27	Annika Nilsson et al
Sweden/20-14262/2020	EPI_ISL_469055	Jourcentralen	The Public Health Agency of Sweden	2020-05-02	Oskar Karlsson Lindsjo et al
Sweden/20-14317/2020	EPI_ISL_469056	Jourcentralen	The Public Health Agency of Sweden	2020-05-05	Oskar Karlsson Lindsjo et al
Sweden/20-14318/2020	EPI_ISL_469057	Inger Landgren	The Public Health Agency of Sweden	2020-05-05	Oskar Karlsson Lindsjo et al
Sweden/20-14377/2020	EPI_ISL_469058	Narhalsan Sjobo vardcentral	The Public Health Agency of Sweden	2020-05-07	Oskar Karlsson Lindsjo et al
Sweden/20-14647/2020	EPI_ISL_469059	Hovas Askim Familjelakare och BVC	The Public Health Agency of Sweden	2020-05-14	Oskar Karlsson Lindsjo et al
Sweden/20-14696/2020	EPI_ISL_469060	Narhalsan Sjobo vardcentral	The Public Health Agency of Sweden	2020-05-15	Oskar Karlsson Lindsjo et al
Sweden/20-14697/2020	EPI_ISL_469061	Narhalsan Sjobo vardcentral	The Public Health Agency of Sweden	2020-05-15	Oskar Karlsson Lindsjo et al
Sweden/20-14785/2020	EPI_ISL_469062	Huddinge VC	The Public Health Agency of Sweden	2020-05-15	Oskar Karlsson Lindsjo et al
Sweden/20-14827/2020	EPI_ISL_469063	Ulltuna Vardcentral	The Public Health Agency of Sweden	2020-05-19	Oskar Karlsson Lindsjo et al
Sweden/20-19008/2020	EPI_ISL_469064	Huddinge VC	The Public Health Agency of Sweden	2020-05-27	Oskar Karlsson Lindsjo et al
Sweden/20-19192/2020	EPI_ISL_469065	Huddinge VC	The Public Health Agency of Sweden	2020-05-28	Oskar Karlsson Lindsjo et al
Sweden/20-50096/2020	EPI_ISL_454876	Karolinska Universitetslaboratoriet	The Public Health Agency of Sweden	2020-03-04	Anna-Malin Linde et al
Sweden/20-50130/2020	EPI_ISL_429141	Klinisk mikrobiologi Orebro	The Public Health Agency of Sweden	2020-03-06	Martin Sundqvist et al
Sweden/20-50149/2020	EPI_ISL_455859	Karolinska Universitetslaboratoriet	The Public Health Agency of Sweden	2020-03-04	Anna-Malin Linde et al
Sweden/20-50180/2020	EPI_ISL_429153	Klinisk mikrobiologi Orebro	The Public Health Agency of Sweden	2020-03-08	Martin Sundqvist et al
Sweden/20-50202/2020	EPI_ISL_455868	Karolinska Universitetslaboratoriet	The Public Health Agency of Sweden	2020-03-05	Anna-Malin Linde et al
Sweden/20-50245/2020	EPI_ISL_455885	Karolinska Universitetslaboratoriet	The Public Health Agency of Sweden	2020-03-07	Anna-Malin Linde et al
Sweden/20-50258/2020	EPI_ISL_455896	Karolinska Universitetslaboratoriet	The Public Health Agency of Sweden	2020-03-08	Anna-Malin Linde et al
Sweden/20-50266/2020	EPI_ISL_455907	Klinisk mikrobiologi, UAS	The Public Health Agency of Sweden	2020-03-06	Anna-Malin Linde et al
Sweden/20-50272/2020	EPI_ISL_455905	Klinisk mikrobiologi, UAS	The Public Health Agency of Sweden	2020-03-09	Anna-Malin Linde et al
Sweden/20-50294/2020	EPI_ISL_454902	Karolinska Universitetslaboratoriet	The Public Health Agency of Sweden	2020-03-11	Anna-Malin Linde et al
Sweden/20-50357/2020	EPI_ISL_429162	The Public Health Agency of Sweden	The Public Health Agency of Sweden	2020-03-15	Olov Svartstrom et al
Sweden/20-51160/2020	EPI_ISL_452242	Wernstedt Medical AB	The Public Health Agency of Sweden	2020-05-04	Eva Sandberg et al
Sweden/20-51167/2020	EPI_ISL_450832	Byjorden vardcentral	The Public Health Agency of Sweden	2020-05-04	Pernilla Brunman et al
Sweden/20-51264/2020	EPI_ISL_455104	Ulltuna Vardcentral	The Public Health Agency of Sweden	2020-05-06	Heidi Lindback et al
Sweden/20-51311/2020	EPI_ISL_450833	Wetterhalsan	The Public Health Agency of Sweden	2020-05-07	Anders Tengblad et al

Sweden/20-51317/2020	EPI_ISL_455105	Wetterhalsan	The Public Health Agency of Sweden	2020-05-07	Anders Tengblad et al
Sweden/20-51349/2020	EPI_ISL_455106	Surbrunns VC	The Public Health Agency of Sweden	2020-05-11	Erik Embring et al
Sweden/20-51350/2020	EPI_ISL_469066	Surbrunns VC	The Public Health Agency of Sweden	2020-05-11	Oskar Karlsson Lindsjo et al
Sweden/20-51387/2020	EPI_ISL_455107	Narhalsan Mellerud	The Public Health Agency of Sweden	2020-05-12	Maria Nykvist et al
Sweden/20-51391/2020	EPI_ISL_455108	Smedby HC	The Public Health Agency of Sweden	2020-05-13	Susanne Brunby et al
Sweden/20-51426/2020	EPI_ISL_455109	Ulltuna Vardcentral	The Public Health Agency of Sweden	2020-05-13	Heidi Lindback et al
Sweden/20-51445/2020	EPI_ISL_455110	Scania Halsocenter, B288	The Public Health Agency of Sweden	2020-05-14	Christina Lergin et al
Sweden/20-51474/2020	EPI_ISL_455111	Olof Norrby	The Public Health Agency of Sweden	2020-05-14	Bla Kustens halsocentral et al
Sweden/20-51598/2020	EPI_ISL_469067	Kungsholmsdoktorn	The Public Health Agency of Sweden	2020-05-19	Oskar Karlsson Lindsjo et al
Sweden/20-51618/2020	EPI_ISL_469068	Hovas Askim Familjelakare och BVC	The Public Health Agency of Sweden	2020-05-20	Oskar Karlsson Lindsjo et al
Sweden/20-51678/2020	EPI_ISL_469069	Narhalsan Olskroken VC	The Public Health Agency of Sweden	2020-05-25	Oskar Karlsson Lindsjo et al
Sweden/20-51703/2020	EPI_ISL_469070	Surbrunns VC	The Public Health Agency of Sweden	2020-05-26	Oskar Karlsson Lindsjo et al
Sweden/20-51758/2020	EPI_ISL_469071	Wasterlakarna	The Public Health Agency of Sweden	2020-05-27	Oskar Karlsson Lindsjo et al
Sweden/20-51891/2020	EPI_ISL_469072	Ulltuna Vardcentral	The Public Health Agency of Sweden	2020-06-03	Oskar Karlsson Lindsjo et al
Sweden/RV-FOI-1/2020	EPI_ISL_424703	Klinisk mikrobiologi, Region Västerbotten	Unit for Biological Agents, Department for CBRN Defence and Security, Swedish Defence Research Agency	2020-03-15	FOI Bioinformatics team et al
Switzerland/100009/2020	EPI_ISL_466934	Viollier AG	Department of Biosystems Science and Engineering, ETH Zürich	2020-03-05	Christian Beisel et al
Switzerland/100019/2020	EPI_ISL_466943	Viollier AG	Department of Biosystems Science and Engineering, ETH Zürich	2020-03-09	Christian Beisel et al
Switzerland/100028/2020	EPI_ISL_466952	Viollier AG	Department of Biosystems Science and Engineering, ETH Zürich	2020-03-10	Christian Beisel et al
Switzerland/100037/2020	EPI_ISL_466961	Viollier AG	Department of Biosystems Science and Engineering, ETH Zürich	2020-03-11	Christian Beisel et al
Switzerland/100042/2020	EPI_ISL_466965	Viollier AG	Department of Biosystems Science and Engineering, ETH Zürich	2020-03-11	Christian Beisel et al
Switzerland/1000477377/2020	EPI_ISL_413020	Department of Internal Medicine, Triemli Hospital	Institute of Medical Virology, University of Zurich	2020-02-27	Stefan Schmutz et al B (https://virological.org/t/phylodynamic-analyses-based-on-11-genomes-from-the-italian-outbreak/426)
Switzerland/1000477757/2020	EPI_ISL_413021	Klinik Hirslanden Zurich	Institute of Medical Virology, University of Zurich	2020-02-29	Stefan Schmutz et al C (https://dx.doi.org/10.1002/jmv.25773)
Switzerland/1000477796/2020	EPI_ISL_413022	Division of Infectious Diseases, University Hospital Zurich	Institute of Medical Virology, University of Zurich	2020-02-29	Stefan Schmutz et al C (https://dx.doi.org/10.1002/jmv.25773)
Switzerland/1000477797/2020	EPI_ISL_413023	Division of Infectious Diseases, University Hospital Zurich	Institute of Medical Virology, University of Zurich	2020-02-29	Stefan Schmutz et al C (https://dx.doi.org/10.1002/jmv.25773)
Switzerland/1000477806/2020	EPI_ISL_413024	Division of Infectious Diseases, University Hospital Zurich	Institute of Medical Virology, University of Zurich	2020-02-29	Stefan Schmutz et al C (https://dx.doi.org/10.1002/jmv.25773)
Switzerland/100055/2020	EPI_ISL_466977	Viollier AG	Department of Biosystems Science and Engineering, ETH Zürich	2020-03-12	Christian Beisel et al
Switzerland/100063/2020	EPI_ISL_466985	Viollier AG	Department of Biosystems Science and Engineering, ETH Zürich	2020-03-12	Christian Beisel et al
Switzerland/100072/2020	EPI_ISL_466994	Viollier AG	Department of Biosystems Science and Engineering, ETH Zürich	2020-03-12	Christian Beisel et al
Switzerland/100127/2020	EPI_ISL_451711	Viollier AG	Department of Biosystems Science and Engineering, ETH Zürich	2020-03-16	Christian Beisel et al
Switzerland/100798/2020	EPI_ISL_451748	Viollier AG	Department of Biosystems Science and Engineering, ETH Zürich	2020-03-25	Christian Beisel et al
Switzerland/100811/2020	EPI_ISL_451761	Viollier AG	Department of Biosystems Science and Engineering, ETH Zürich	2020-03-26	Christian Beisel et al
Switzerland/100823/2020	EPI_ISL_451772	Viollier AG	Department of Biosystems Science and Engineering, ETH Zürich	2020-03-26	Christian Beisel et al
Switzerland/100830/2020	EPI_ISL_451778	Viollier AG	Department of Biosystems Science and Engineering, ETH Zürich	2020-03-26	Christian Beisel et al
Switzerland/100834/2020	EPI_ISL_451782	Viollier AG	Department of Biosystems Science and Engineering, ETH Zürich	2020-03-25	Christian Beisel et al
Switzerland/100846/2020	EPI_ISL_451793	Viollier AG	Department of Biosystems Science and Engineering, ETH Zürich	2020-03-25	Christian Beisel et al
Switzerland/101140/2020	EPI_ISL_451807	Viollier AG	Department of Biosystems Science and Engineering, ETH Zürich	2020-03-31	Christian Beisel et al
Switzerland/101141/2020	EPI_ISL_451808	Viollier AG	Department of Biosystems Science and Engineering, ETH Zürich	2020-03-31	Christian Beisel et al
Switzerland/101146/2020	EPI_ISL_451813	Viollier AG	Department of Biosystems Science and Engineering, ETH Zürich	2020-03-31	Christian Beisel et al
Switzerland/101160/2020	EPI_ISL_451826	Viollier AG	Department of Biosystems Science and Engineering, ETH Zürich	2020-03-31	Christian Beisel et al
Switzerland/101172/2020	EPI_ISL_451835	Viollier AG	Department of Biosystems Science and Engineering, ETH Zürich	2020-03-31	Christian Beisel et al
Switzerland/101178/2020	EPI_ISL_451840	Viollier AG	Department of Biosystems Science and Engineering, ETH Zürich	2020-03-31	Christian Beisel et al
Switzerland/101190/2020	EPI_ISL_451852	Viollier AG	Department of Biosystems Science and Engineering, ETH Zürich	2020-03-31	Christian Beisel et al
Switzerland/110429/2020	EPI_ISL_451858	Viollier AG	Department of Biosystems Science and Engineering, ETH Zürich	2020-04-07	Christian Beisel et al
Switzerland/110448/2020	EPI_ISL_451869	Viollier AG	Department of Biosystems Science and Engineering, ETH Zürich	2020-04-07	Christian Beisel et al
Switzerland/110450/2020	EPI_ISL_451870	Viollier AG	Department of Biosystems Science and Engineering, ETH Zürich	2020-04-07	Christian Beisel et al
Switzerland/110454/2020	EPI_ISL_451874	Viollier AG	Department of Biosystems Science and Engineering, ETH Zürich	2020-04-07	Christian Beisel et al
Switzerland/110459/2020	EPI_ISL_451878	Viollier AG	Department of Biosystems Science and Engineering, ETH Zürich	2020-04-07	Christian Beisel et al
Switzerland/110460/2020	EPI_ISL_451879	Viollier AG	Department of Biosystems Science and Engineering, ETH Zürich	2020-04-07	Christian Beisel et al
Switzerland/110464/2020	EPI_ISL_451882	Viollier AG	Department of Biosystems Science and Engineering, ETH Zürich	2020-04-07	Christian Beisel et al
Switzerland/110467/2020	EPI_ISL_451885	Viollier AG	Department of Biosystems Science and Engineering, ETH Zürich	2020-04-06	Christian Beisel et al
Switzerland/110470/2020	EPI_ISL_451888	Viollier AG	Department of Biosystems Science and Engineering, ETH Zürich	2020-04-06	Christian Beisel et al
Switzerland/110478/2020	EPI_ISL_451890	Viollier AG	Department of Biosystems Science and Engineering, ETH Zürich	2020-04-07	Christian Beisel et al
Switzerland/110480/2020	EPI_ISL_451892	Viollier AG	Department of Biosystems Science and Engineering, ETH Zürich	2020-04-07	Christian Beisel et al
Switzerland/110486/2020	EPI_ISL_451898	Viollier AG	Department of Biosystems Science and Engineering, ETH Zürich	2020-04-07	Christian Beisel et al
Switzerland/120001/2020	EPI_ISL_468202	Viollier AG	Department of Biosystems Science and Engineering, ETH Zürich	2020-04-17	Christian Beisel et al
Switzerland/120035/2020	EPI_ISL_468225	Viollier AG	Department of Biosystems Science and Engineering, ETH Zürich	2020-04-19	Christian Beisel et al
Switzerland/120039/2020	EPI_ISL_468228	Viollier AG	Department of Biosystems Science and Engineering, ETH Zürich	2020-04-20	Christian Beisel et al
Switzerland/120040/2020	EPI_ISL_468229	Viollier AG	Department of Biosystems Science and Engineering, ETH Zürich	2020-04-20	Christian Beisel et al
Switzerland/120047/2020	EPI_ISL_468234	Viollier AG	Department of Biosystems Science and Engineering, ETH Zürich	2020-04-20	Christian Beisel et al
Switzerland/120166/2020	EPI_ISL_468244	Viollier AG	Department of Biosystems Science and Engineering, ETH Zürich	2020-04-26	Christian Beisel et al
Switzerland/120168/2020	EPI_ISL_468246	Viollier AG	Department of Biosystems Science and Engineering, ETH Zürich	2020-04-26	Christian Beisel et al
Switzerland/120175/2020	EPI_ISL_468252	Viollier AG	Department of Biosystems Science and Engineering, ETH Zürich	2020-04-27	Christian Beisel et al
Switzerland/120179/2020	EPI_ISL_468255	Viollier AG	Department of Biosystems Science and Engineering, ETH Zürich	2020-04-27	Christian Beisel et al
Switzerland/120182/2020	EPI_ISL_468257	Viollier AG	Department of Biosystems Science and Engineering, ETH Zürich	2020-04-27	Christian Beisel et al
Switzerland/120183/2020	EPI_ISL_468258	Viollier AG	Department of Biosystems Science and Engineering, ETH Zürich	2020-04-27	Christian Beisel et al
Switzerland/120188/2020	EPI_ISL_468263	Viollier AG	Department of Biosystems Science and Engineering, ETH Zürich	2020-04-27	Christian Beisel et al
Switzerland/120192/2020	EPI_ISL_468267	Viollier AG	Department of Biosystems Science and Engineering, ETH Zürich	2020-04-27	Christian Beisel et al
Switzerland/120206/2020	EPI_ISL_468279	Viollier AG	Department of Biosystems Science and Engineering, ETH Zürich	2020-04-27	Christian Beisel et al
Switzerland/120207/2020	EPI_ISL_468280	Viollier AG	Department of Biosystems Science and Engineering, ETH Zürich	2020-04-28	Christian Beisel et al
Switzerland/120223/2020	EPI_ISL_468285	Viollier AG	Department of Biosystems Science and Engineering, ETH Zürich	2020-04-30	Christian Beisel et al
Switzerland/120227/2020	EPI_ISL_468288	Viollier AG	Department of Biosystems Science and Engineering, ETH Zürich	2020-05-01	Christian Beisel et al
Switzerland/120228/2020	EPI_ISL_468289	Viollier AG	Department of Biosystems Science and Engineering, ETH Zürich	2020-05-01	Christian Beisel et al
Switzerland/120229/2020	EPI_ISL_468290	Viollier AG	Department of Biosystems Science and Engineering, ETH Zürich	2020-05-01	Christian Beisel et al
Switzerland/120230/2020	EPI_ISL_468291	Viollier AG	Department of Biosystems Science and Engineering, ETH Zürich	2020-05-01	Christian Beisel et al
Switzerland/120232/2020	EPI_ISL_468293	Viollier AG	Department of Biosystems Science and Engineering, ETH Zürich	2020-05-01	Christian Beisel et al
Switzerland/120233/2020	EPI_ISL_468294	Viollier AG	Department of Biosystems Science and Engineering, ETH Zürich	2020-05-01	Christian Beisel et al
Switzerland/120234/2020	EPI_ISL_468295	Viollier AG	Department of Biosystems Science and Engineering, ETH Zürich	2020-05-01	Christian Beisel et al
Switzerland/120235/2020	EPI_ISL_468296	Viollier AG	Department of Biosystems Science and Engineering, ETH Zürich	2020-05-01	Christian Beisel et al
Switzerland/120236/2020	EPI_ISL_468297	Viollier AG	Department of Biosystems Science and Engineering, ETH Zürich	2020-05-01	Christian Beisel et al
Switzerland/120237/2020	EPI_ISL_468298	Viollier AG	Department of Biosystems Science and Engineering, ETH Zürich	2020-05-01	Christian Beisel et al
Switzerland/120238/2020	EPI_ISL_468299	Viollier AG	Department of Biosystems Science and Engineering, ETH Zürich	2020-05-01	Christian Beisel et al
Switzerland/120241/2020	EPI_ISL_468301	Viollier AG	Department of Biosystems Science and Engineering, ETH Zürich	2020-05-04	Christian Beisel et al
Switzerland/120242/2020	EPI_ISL_468302	Viollier AG	Department of Biosystems Science and Engineering, ETH Zürich	2020-05-04	Christian Beisel et al
Switzerland/120244/2020	EPI_ISL_468303	Viollier AG	Department of Biosystems Science and Engineering, ETH Zürich	2020-05-04	Christian Beisel et al
Switzerland/130001/2020	EPI_ISL_466997	Viollier AG	Department of Biosystems Science and Engineering, ETH Zürich	2020-05-05	Christian Beisel et al
Switzerland/130003/2020	EPI_ISL_466999	Viollier AG	Department of Biosystems Science and Engineering, ETH Zürich	2020-05-05	Christian Beisel et al
Switzerland/130004/2020	EPI_ISL_467000	Viollier AG	Department of Biosystems Science and Engineering, ETH Zürich	2020-05-05	Christian Beisel et al
Switzerland/130005/2020	EPI_ISL_467001	Viollier AG	Department of Biosystems Science and Engineering, ETH Zürich	2020-05-06	Christian Beisel et al
Switzerland/130006/2020	EPI_ISL_467002	Viollier AG	Department of Biosystems Science and Engineering, ETH Zürich	2020-05-06	Christian Beisel et al
Switzerland/130007/2020	EPI_ISL_467003	Viollier AG	Department of Biosystems Science and Engineering, ETH Zürich	2020-05-06	Christian Beisel et al
Switzerland/130008/2020	EPI_ISL_467004	Viollier AG	Department of Biosystems Science and Engineering, ETH Zürich	2020-05-06	Christian Beisel et al
Switzerland/130012/2020	EPI_ISL_467006	Viollier AG	Department of Biosystems Science and Engineering, ETH Zürich	2020-05-07	Christian Beisel et al
Switzerland/130013/2020	EPI_ISL_467007	Viollier AG	Department of Biosystems Science and Engineering, ETH Zürich	2020-05-07	Christian Beisel et al
Switzerland/130014/2020	EPI_ISL_467008	Viollier AG	Department of Biosystems Science and Engineering, ETH Zürich	2020-05-07	Christian Beisel et al
Switzerland/130018/2020	EPI_ISL_467010	Viollier AG	Department of Biosystems Science and Engineering, ETH Zürich	2020-05-08	Christian Beisel et al
Switzerland/130020/2020	EPI_ISL_467012	Viollier AG	Department of Biosystems Science and Engineering, ETH Zürich	2020-05-08	Christian Beisel et al
Switzerland/130021/2020	EPI_ISL_467013	Viollier AG	Department of Biosystems Science and Engineering, ETH Zürich	2020-05-11	Christian Beisel et al
Switzerland/130026/2020	EPI_ISL_467014	Viollier AG	Department of Biosystems Science and Engineering, ETH Zürich	2020-05-12	Christian Beisel et al
Switzerland/130028/2020	EPI_ISL_467015	Viollier AG	Department of Biosystems Science and Engineering, ETH Zürich	2020-05-13	Christian Beisel et al
Switzerland/130032/2020	EPI_ISL_467016	Viollier AG	Department of Biosystems Science and Engineering, ETH Zürich	2020-05-13	Christian Beisel et al
Switzerland/42169171/2020	EPI_ISL_418271	University Hospital Basel, Clinical Virology	University Hospital Basel, Labormedizin	2020-02-27	Hirsch et al
Switzerland/42169310/2020	EPI_ISL_418275	University Hospital Basel, Clinical Virology	University Hospital Basel, Clinical Bacteriology	2020-02-27	Hirsch et al
Switzerland/42169471/2020	EPI_ISL_418273	University Hospital Basel, Clinical Virology	University Hospital Basel, Labormedizin	2020-02-28	Hirsch et al
Switzerland/42170345/2020	EPI_ISL_418274	University Hospital Basel, Clinical Virology	University Hospital Basel, Clinical Bacteriology	2020-02-28	Hirsch et al
Switzerland/42174724/2020	EPI_ISL_418277	University Hospital Basel, Clinical Virology	University Hospital Basel, Clinical Bacteriology	2020-03-03	Hirsch et al
Switzerland/42175220/2020	EPI_ISL_418280	University Hospital Basel, Clinical Virology	University Hospital Basel, Clinical Bacteriology	2020-03-03	Hirsch et al
Switzerland/42176560/2020	EPI_ISL_418283	University Hospital Basel, Clinical Virology	University Hospital Basel, Clinical Bacteriology	2020-03-04	Hirsch et al
Switzerland/42176771/2020	EPI_ISL_418285	University Hospital Basel, Clinical Virology	University Hospital Basel, Clinical Bacteriology	2020-03-04	Hirsch et al
Switzerland/42177236/2020	EPI_ISL_418433	University Hospital Basel, Clinical Virology	University Hospital Basel, Clinical Bacteriology	2020-03-05	Hirsch et al
Switzerland/42177472/2020	EPI_ISL_418436	University Hospital Basel, Clinical Virology	University Hospital Basel, Clinical Virology	2020-03-05	Hirsch et al
Switzerland/AG0361/2020	EPI_ISL_413999	Laboratoire de Virologie, HUG	Swiss National Reference Centre for Influenza	2020-02-27	LAUBSCHER Florian et al. et al
Switzerland/AG7120/2020	EPI_ISL_415457	Hôpitaux universitaires de Genève Laboratoire de Virologie	Hôpitaux universitaires de Genève Laboratoire de Virologie	2020-02-29	Laubscher F. et al
Switzerland/BE6651/2020	EPI_ISL_415456	Hôpitaux universitaires de Genève Laboratoire de Virologie	Hôpitaux universitaires de Genève Laboratoire de Virologie	2020-02-29	Laubscher F. et al
Switzerland/BL0902/2020	EPI_ISL_414021	Laboratoire de Virologie, HUG	Swiss National Reference Centre for Influenza	2020-02-27	LAUBSCHER Florian et al. et al
Switzerland/GE0199/2020	EPI_ISL_415455	Hôpitaux universitaires de Genève Laboratoire de Virologie	Hôpitaux universitaires de Genève Laboratoire de Virologie	2020-02-28	Laubscher F. et al
Switzerland/GE0304/2020	EPI_ISL_429218	University Hospitals of Geneva Laboratory of Virology	University Hospitals of Geneva Laboratory of Virology	2020-04-02	Laubscher F. et al

Switzerland/GE1184/2020	EPI_ISL_429207	University Hospitals of Geneva Laboratory of Virology	University Hospitals of Geneva Laboratory of Virology	2020-04-03	Laubscher F. et al
Switzerland/GE1402/2020	EPI_ISL_415700	Hôpitaux universitaires de Genève Laboratoire de Virologie	Hôpitaux universitaires de Genève Laboratoire de Virologie	2020-02-28	Laubscher F. et al
Switzerland/GE1422/2020	EPI_ISL_415454	Hôpitaux universitaires de Genève Laboratoire de Virologie	Hôpitaux universitaires de Genève Laboratoire de Virologie	2020-02-28	Laubscher F. et al
Switzerland/GE3121/2020	EPI_ISL_414019	Laboratoire de Virologie, HUG	Swiss National Reference Centre for Influenza	2020-02-27	LAUBSCHER Florian et al. et al
Switzerland/GE3144/2020	EPI_ISL_429196	University Hospitals of Geneva Laboratory of Virology	University Hospitals of Geneva Laboratory of Virology	2020-03-18	Laubscher F. et al
Switzerland/GE3895/2020	EPI_ISL_413997	Laboratoire de Virologie, HUG	Swiss National Reference Centre for Influenza	2020-02-26	LAUBSCHER Florian et al. et al
Switzerland/GE5373/2020	EPI_ISL_414020	Laboratoire de Virologie, HUG	Swiss National Reference Centre for Influenza	2020-02-27	LAUBSCHER Florian et al. et al
Switzerland/GE5737/2020	EPI_ISL_429200	University Hospitals of Geneva Laboratory of Virology	University Hospitals of Geneva Laboratory of Virology	2020-03-19	Laubscher F. et al
Switzerland/GE9586/2020	EPI_ISL_414022	Laboratoire de Virologie, HUG	Swiss National Reference Centre for Influenza	2020-02-27	LAUBSCHER Florian et al. et al
Switzerland/GR2988/2020	EPI_ISL_415698	Hôpitaux universitaires de Genève Laboratoire de Virologie	Hôpitaux universitaires de Genève Laboratoire de Virologie	2020-02-27	Laubscher F. et al
Switzerland/GR3043/2020	EPI_ISL_415699	Hôpitaux universitaires de Genève Laboratoire de Virologie	Hôpitaux universitaires de Genève Laboratoire de Virologie	2020-02-27	Laubscher F. et al
Switzerland/TI9486/2020	EPI_ISL_413996	Laboratoire de Virologie, HUG	Swiss National Reference Centre for Influenza	2020-02-24	LAUBSCHER Florian et al. et al
Switzerland/VD0503/2020	EPI_ISL_415459	Hôpitaux universitaires de Genève Laboratoire de Virologie	Hôpitaux universitaires de Genève Laboratoire de Virologie	2020-02-29	Laubscher F. et al
Taiwan/108/2020	EPI_ISL_429883	Centers for Disease Control, R.O.C. (Taiwan)	Centers for Disease Control, R.O.C. (Taiwan)	2020-03-23	Ji-Rong Yang et al A
Taiwan/128/2020	EPI_ISL_420083	Centers for Disease Control, R.O.C. (Taiwan)	Centers for Disease Control, R.O.C. (Taiwan)	2020-03-18	Ji-Rong Yang et al A
Taiwan/144/2020	EPI_ISL_421641	Centers for Disease Control, R.O.C. (Taiwan)	Centers for Disease Control, R.O.C. (Taiwan)	2020-03-19	Ji-Rong Yang et al A
Taiwan/170/2020	EPI_ISL_420084	Centers for Disease Control, R.O.C. (Taiwan)	Centers for Disease Control, R.O.C. (Taiwan)	2020-03-21	Ji-Rong Yang et al A
Taiwan/2/2020	EPI_ISL_406031	Centers for Disease Control, R.O.C. (Taiwan)	Centers for Disease Control, R.O.C. (Taiwan)	2020-01-23	Ji-Rong Yang et al C (https://dx.doi.org/10.1099/acmi.0.000133)
Taiwan/225/2020	EPI_ISL_421651	Centers for Disease Control, R.O.C. (Taiwan)	Centers for Disease Control, R.O.C. (Taiwan)	2020-03-22	Ji-Rong Yang et al A
Taiwan/3/2020	EPI_ISL_411926	Taiwan Centers for Disease Control	Taiwan Centers for Disease Control	2020-01-24	Ji-Rong Yang et al A
Taiwan/4/2020	EPI_ISL_411927	Taiwan Centers for Disease Control	Taiwan Centers for Disease Control	2020-01-28	Ji-Rong Yang et al B (https://dx.doi.org/10.2807/1560-7917.ES.2020.25.13.2000305)
Taiwan/5/2020	EPI_ISL_428489	Centers for Disease Control, R.O.C. (Taiwan)	Centers for Disease Control, R.O.C. (Taiwan)	2020-01-31	Ji-Rong Yang et al A
Taiwan/8/2020	EPI_ISL_428488	Centers for Disease Control, R.O.C. (Taiwan)	Centers for Disease Control, R.O.C. (Taiwan)	2020-01-31	Ji-Rong Yang et al A
Taiwan/CGMH-CGU-01/2020	EPI_ISL_411915	Laboratory Medicine	Department of Laboratory Medicine, Lin-Kou Chang Gung Memorial Hospital, Taoyuan, Taiwan.	2020-01-25	Kuo-Chien Tsao et al
Taiwan/CGMH-CGU-02/2020	EPI_ISL_417518	Laboratory Medicine	Department of Laboratory Medicine, Lin-Kou Chang Gung Memorial Hospital, Taoyuan, Taiwan	2020-02-04	Kuo-Chien Tsao et al
Taiwan/CGMH-CGU-03/2020	EPI_ISL_415741	Laboratory Medicine	Department of Laboratory Medicine, Lin-Kou Chang Gung Memorial Hospital, Taoyuan, Taiwan	2020-02-26	Kuo-Chien Tsao et al
Taiwan/CGMH-CGU-04/2020	EPI_ISL_415742	Laboratory Medicine	Department of Laboratory Medicine, Lin-Kou Chang Gung Memorial Hospital, Taoyuan, Taiwan	2020-02-27	Kuo-Chien Tsao et al
Taiwan/CGMH-CGU-05/2020	EPI_ISL_415743	Laboratory Medicine	Department of Laboratory Medicine, Lin-Kou Chang Gung Memorial Hospital, Taoyuan, Taiwan	2020-02-27	Kuo-Chien Tsao et al
Taiwan/CGMH-CGU-07/2020	EPI_ISL_417520	Laboratory Medicine	Department of Laboratory Medicine, Lin-Kou Chang Gung Memorial Hospital, Taoyuan, Taiwan	2020-03-09	Kuo-Chien Tsao et al
Taiwan/CGMH-CGU-08/2020	EPI_ISL_417521	Laboratory Medicine	Department of Laboratory Medicine, Lin-Kou Chang Gung Memorial Hospital, Taoyuan, Taiwan	2020-03-10	Kuo-Chien Tsao et al
Taiwan/CGMH-CGU-14/2020	EPI_ISL_424970	Laboratory Medicine	Department of Laboratory Medicine, Lin-Kou Chang Gung Memorial Hospital, Taoyuan, Taiwan	2020-03-17	Kuo-Chien Tsao et al
Taiwan/CGMH-CGU-16/2020	EPI_ISL_424972	Laboratory Medicine	Department of Laboratory Medicine, Lin-Kou Chang Gung Memorial Hospital, Taoyuan, Taiwan	2020-03-16	Kuo-Chien Tsao et al
Taiwan/CGMH-CGU-19/2020	EPI_ISL_424975	Laboratory Medicine	Department of Laboratory Medicine, Lin-Kou Chang Gung Memorial Hospital, Taoyuan, Taiwan	2020-03-18	Kuo-Chien Tsao et al
Taiwan/CGMH-CGU-21/2020	EPI_ISL_444274	Laboratory Medicine	Department of Laboratory Medicine, Lin-Kou Chang Gung Memorial Hospital, Taoyuan, Taiwan	2020-02-29	Kuo-Chien Tsao et al
Taiwan/CGMH-CGU-23/2020	EPI_ISL_444276	Laboratory Medicine	Department of Laboratory Medicine, Lin-Kou Chang Gung Memorial Hospital, Taoyuan, Taiwan	2020-03-21	Kuo-Chien Tsao et al
Taiwan/CGMH-CGU-25/2020	EPI_ISL_444278	Laboratory Medicine	Department of Laboratory Medicine, Lin-Kou Chang Gung Memorial Hospital, Taoyuan, Taiwan	2020-03-20	Kuo-Chien Tsao et al
Taiwan/CGMH-CGU-26/2020	EPI_ISL_452178	Laboratory Medicine	Department of Laboratory Medicine, Lin-Kou Chang Gung Memorial Hospital, Taoyuan, Taiwan	2020-04-19	Kuo-Chien Tsao et al
Taiwan/CGMH-CGU-27/2020	EPI_ISL_452179	Laboratory Medicine	Department of Laboratory Medicine, Lin-Kou Chang Gung Memorial Hospital, Taoyuan, Taiwan	2020-04-19	Kuo-Chien Tsao et al
Taiwan/CGMH-CGU-30/2020	EPI_ISL_464094	Laboratory Medicine	Department of Laboratory Medicine, Lin-Kou Chang Gung Memorial Hospital, Taoyuan, Taiwan	2020-04-11	Kuo-Chien Tsao et al
Taiwan/NTU01/2020	EPI_ISL_408489	Department of Laboratory Medicine, National Taiwan University Hospital	Microbial Genomics Core Lab, National Taiwan University Centers of Genomic and Precision Medicine	2020-01-31	Shiou-Hwei Yeh et al B
Taiwan/NTU02/2020	EPI_ISL_410218	Department of Laboratory Medicine, National Taiwan University Hospital	Microbial Genomics Core Lab, National Taiwan University Centers of Genomic and Precision Medicine	2020-02-05	Shiou-Hwei Yeh et al A (http://biorxiv.org/lookup/doi/10.1101/2020.04.01.019463)
Taiwan/NTU04/2020	EPI_ISL_422407	Department of Laboratory Medicine, National Taiwan University Hospital	Microbial Genomics Core Lab, National Taiwan University Centers of Genomic and Precision Medicine	2020-03-04	Shiou-Hwei Yeh et al B
Taiwan/NTU07/2020	EPI_ISL_422410	Department of Laboratory Medicine, National Taiwan University Hospital	Microbial Genomics Core Lab, National Taiwan University Centers of Genomic and Precision Medicine	2020-03-13	Shiou-Hwei Yeh et al B
Taiwan/NTU22/2020	EPI_ISL_447616	Department of Laboratory Medicine, National Taiwan University Hospital	Microbial Genomics Core Lab, National Taiwan University Centers of Genomic and Precision Medicine	2020-03-21	Shiou-Hwei Yeh et al B
Taiwan/NTU23/2020	EPI_ISL_447617	Department of Laboratory Medicine, National Taiwan University Hospital	Microbial Genomics Core Lab, National Taiwan University Centers of Genomic and Precision Medicine	2020-03-25	Shiou-Hwei Yeh et al B
Taiwan/NTU24/2020	EPI_ISL_447618	Department of Laboratory Medicine, National Taiwan University Hospital	Microbial Genomics Core Lab, National Taiwan University Centers of Genomic and Precision Medicine	2020-03-25	Shiou-Hwei Yeh et al B
Taiwan/NTU26/2020	EPI_ISL_447620	Department of Laboratory Medicine, National Taiwan University Hospital	Microbial Genomics Core Lab, National Taiwan University Centers of Genomic and Precision Medicine	2020-03-30	Shiou-Hwei Yeh et al B
Taiwan/NTU27/2020	EPI_ISL_447621	Department of Laboratory Medicine, National Taiwan University Hospital	Microbial Genomics Core Lab, National Taiwan University Centers of Genomic and Precision Medicine	2020-04-13	Shiou-Hwei Yeh et al B
Taiwan/NTU28/2020	EPI_ISL_447622	Department of Laboratory Medicine, National Taiwan University Hospital	Microbial Genomics Core Lab, National Taiwan University Centers of Genomic and Precision Medicine	2020-04-27	Shiou-Hwei Yeh et al B
Taiwan/NTU29/2020	EPI_ISL_463007	Department of Laboratory Medicine, National Taiwan University Hospital	Microbial Genomics Core Lab, National Taiwan University Centers of Genomic and Precision Medicine	2020-04-12	Shiou-Hwei Yeh et al B
Taiwan/TSGH-04/2020	EPI_ISL_426632	TSGH-CP molecular lab	TSGH-CP molecular lab	2020-03-17	Cherng-Lih Perng et al
Taiwan/TSGH-05/2020	EPI_ISL_427392	TSGH-CP molecular lab	TSGH-CP molecular lab	2020-03-14	Cherng-Lih Perng et al
Taiwan/TSGH-07/2020	EPI_ISL_427394	TSGH-CP molecular lab	TSGH-CP molecular lab	2020-03-22	Cherng-Lih Perng et al
Taiwan/TSGH-08/2020	EPI_ISL_427395	TSGH-CP molecular lab	TSGH-CP molecular lab	2020-03-23	Cherng-Lih Perng et al
Taiwan/TSGH-14/2020	EPI_ISL_428231	TSGH-CP molecular lab	TSGH-CP molecular lab	2020-03-24	Cherng-Lih Perng et al
Taiwan/TSGH-16/2020	EPI_ISL_436101	TSGH-CP molecular lab	TSGH-CP molecular lab	2020-03-19	Cherng-Lih Perng et al
Taiwan/TSGH-17/2020	EPI_ISL_436102	TSGH-CP molecular lab	TSGH-CP molecular lab	2020-03-19	Cherng-Lih Perng et al
Taiwan/TSGH-18/2020	EPI_ISL_436103	TSGH-CP molecular lab	TSGH-CP molecular lab	2020-03-24	Cherng-Lih Perng et al
Taiwan/TSGH-21/2020	EPI_ISL_436106	TSGH-CP molecular lab	TSGH-CP molecular lab	2020-03-30	Cherng-Lih Perng et al
Taiwan/TSGH-22/2020	EPI_ISL_436107	TSGH-CP molecular lab	TSGH-CP molecular lab	2020-03-31	Cherng-Lih Perng et al
Taiwan/TSGH-23/2020	EPI_ISL_436108	TSGH-CP molecular lab	TSGH-CP molecular lab	2020-04-02	Cherng-Lih Perng et al
Taiwan/TSGH-24/2020	EPI_ISL_436099	TSGH-CP molecular lab	TSGH-CP molecular lab	2020-04-02	Cherng-Lih Perng et al
Taiwan/TSGH-26/2020	EPI_ISL_447252	TSGH-CP molecular lab	TSGH-CP molecular lab	2020-02-25	Cherng-Lih Perng et al
Taiwan/TSGH-27/2020	EPI_ISL_447253	TSGH-CP molecular lab	TSGH-CP molecular lab	2020-03-11	Cherng-Lih Perng et al
Taiwan/TSGH-28/2020	EPI_ISL_447254	TSGH-CP molecular lab	TSGH-CP molecular lab	2020-03-16	Cherng-Lih Perng et al
Taiwan/TSGH-34/2020	EPI_ISL_447593	TSGH-CP molecular lab	TSGH-CP molecular lab	2020-04-01	Cherng-Lih Perng et al
Taiwan/TSGH-35/2020	EPI_ISL_457726	TSGH-CP molecular lab	TSGH-CP molecular lab	2020-02-06	Cherng-Lih Perng et al
Taiwan/TSGH-37/2020	EPI_ISL_457733	TSGH-CP molecular lab	TSGH-CP molecular lab	2020-02-08	Cherng-Lih Perng et al
Thailand/61/2020	EPI_ISL_403962	Bamrasnaradura Hospital	1. Department of Medical Sciences, Ministry of Public Health, Thailand 2. Thai Red Cross Emerging Infectious Diseases - Health Science Centre 3. Department of Disease Control, Ministry of Public Health, Thailand	2020-01-08	Pilailuk et al B (https://dx.doi.org/10.2807/1560-7917.ES.2020.25.8.2000097)
Thailand/74/2020	EPI_ISL_403963	Bamrasnaradura Hospital	1. Department of Medical Sciences, Ministry of Public Health, Thailand 2. Thai Red Cross Emerging Infectious Diseases - Health Science Centre 3. Department of Disease Control, Ministry of Public Health, Thailand	2020-01-13	Pilailuk et al B (https://dx.doi.org/10.2807/1560-7917.ES.2020.25.8.2000097)
Thailand/Bangkok_168/2020	EPI_ISL_450196	bumrungrad international hospital	National Institute of Health. Department of medical Sciences, Ministry of Public Health, Thailand	2020-01-23	Pilailuk et al A
Thailand/Bangkok_269/2020	EPI_ISL_447915		National Institute of Health. Department of medical Sciences, Ministry of Public Health, Thailand	2020-01-26	Pilailuk et al A
Thailand/Bangkok_323/2020	EPI_ISL_447916		National Institute of Health. Department of medical Sciences, Ministry of Public Health, Thailand	2020-01-29	Pilailuk et al A
Thailand/Bangkok-0021/2020	EPI_ISL_423042	Ramathibodi Hospital	COVID-19 Network Investigations (CONI) Alliance	2020-03-24	Elizabeth Batty et al B (https://dx.doi.org/10.1101/2020.05.22.20108498)
Thailand/Bangkok-0042/2020	EPI_ISL_429178	Ramathibodi Hospital	COVID-19 Network Investigations (CONI) Alliance	2020-03-28	Elizabeth Batty et al B (https://dx.doi.org/10.1101/2020.05.22.20108498)
Thailand/Bangkok-0046/2020	EPI_ISL_429182	Ramathibodi Hospital	COVID-19 Network Investigations (CONI) Alliance	2020-03-28	Elizabeth Batty et al B (https://dx.doi.org/10.1101/2020.05.22.20108498)
Thailand/Bangkok-0048/2020	EPI_ISL_429184	Ramathibodi Hospital	COVID-19 Network Investigations (CONI) Alliance	2020-03-28	Elizabeth Batty et al B (https://dx.doi.org/10.1101/2020.05.22.20108498)
Thailand/Bangkok-0055/2020	EPI_ISL_446997	Ramathibodi Hospital	COVID-19 Network Investigations (CONI) Alliance	2020-03-28	Elizabeth Batty et al A
Thailand/Bangkok-0056/2020	EPI_ISL_446998	Ramathibodi Hospital	COVID-19 Network Investigations (CONI) Alliance	2020-03-28	Elizabeth Batty et al A
Thailand/Bangkok-0070/2020	EPI_ISL_447012	Ramathibodi Hospital	COVID-19 Network Investigations (CONI) Alliance	2020-03-30	Elizabeth Batty et al A
Thailand/Bangkok-0073/2020	EPI_ISL_447014	Ramathibodi Hospital	COVID-19 Network Investigations (CONI) Alliance	2020-03-03	Elizabeth Batty et al A
Thailand/Bangkok-0077/2020	EPI_ISL_447018	Ramathibodi Hospital	COVID-19 Network Investigations (CONI) Alliance	2020-04-01	Elizabeth Batty et al A
Thailand/Bangkok-0078/2020	EPI_ISL_447019	Ramathibodi Hospital	COVID-19 Network Investigations (CONI) Alliance	2020-04-01	Elizabeth Batty et al A
Thailand/Bangkok-0081/2020	EPI_ISL_447022	Ramathibodi Hospital	COVID-19 Network Investigations (CONI) Alliance	2020-04-02	Elizabeth Batty et al A
Thailand/Bangkok-0082/2020	EPI_ISL_447023	Ramathibodi Hospital	COVID-19 Network Investigations (CONI) Alliance	2020-04-02	Elizabeth Batty et al A
Thailand/Bangkok-0084/2020	EPI_ISL_447025	Ramathibodi Hospital	COVID-19 Network Investigations (CONI) Alliance	2020-04-02	Elizabeth Batty et al A
Thailand/Bangkok-0087/2020	EPI_ISL_447027	Ramathibodi Hospital	COVID-19 Network Investigations (CONI) Alliance	2020-04-06	Elizabeth Batty et al A
Thailand/Bangkok-0088/2020	EPI_ISL_447028	Ramathibodi Hospital	COVID-19 Network Investigations (CONI) Alliance	2020-04-07	Elizabeth Batty et al A
Thailand/Bangkok-CONI-0097/2020	EPI_ISL_458026	Hospital for Tropical Diseases	COVID-19 Network Investigations (CONI) Alliance	2020-04-02	Elizabeth Batty et al A
Thailand/Bangkok-CONI-0098/2020	EPI_ISL_458027	Hospital for Tropical Diseases	COVID-19 Network Investigations (CONI) Alliance	2020-04-03	Elizabeth Batty et al A
Thailand/Bangkok-CONI-0099/2020	EPI_ISL_458028	Hospital for Tropical Diseases	COVID-19 Network Investigations (CONI) Alliance	2020-04-04	Elizabeth Batty et al A
Thailand/Bangkok-CONI-0117/2020	EPI_ISL_455919	Ramathibodi Hospital	COVID-19 Network Investigations (CONI) Alliance	2020-03-12	Elizabeth Batty et al A
Thailand/Bangkok-CONI-0127/2020	EPI_ISL_455921	Ramathibodi Hospital	COVID-19 Network Investigations (CONI) Alliance	2020-03-12	Elizabeth Batty et al A
Thailand/Bangkok-CONI-0149/2020	EPI_ISL_455943	Ramathibodi Hospital	COVID-19 Network Investigations (CONI) Alliance	2020-03-15	Elizabeth Batty et al A
Thailand/NIH-15/2020	EPI_ISL_434692	Bamrasnaradura hospital	National Institute of Health. Department of medical Sciences, Ministry of Public Health, Thailand	2020-01-05	Pilailuk et al A
Thailand/NIH-162/2020	EPI_ISL_434694	Bamrasnaradura hospital	National Institute of Health. Department of medical Sciences, Ministry of Public Health, Thailand	2020-01-22	Pilailuk et al A
Thailand/NIH-190/2020	EPI_ISL_434695		National Institute of Health. Department of medical Sciences, Ministry of Public Health, Thailand	2020-01-22	Pilailuk et al A
Thailand/NIH-2294/2020	EPI_ISL_434698	Praram 9 Hospital	National Institute of Health. Department of medical Sciences, Ministry of Public Health, Thailand	2020-03-13	Pilailuk et al A
Thailand/NIH-59/2020	EPI_ISL_434693	Bamrasnaradura hospital	National Institute of Health. Department of medical Sciences, Ministry of Public Health, Thailand	2020-01-16	Pilailuk et al A
Thailand/Nonthaburi_3472/2020	EPI_ISL_455593		National Institute of Health. Department of medical Sciences, Ministry of Public Health, Thailand	2020-03-20	Pilailuk et al A
Thailand/Nonthaburi_363/2020	EPI_ISL_447917		National Institute of Health. Department of medical Sciences, Ministry of Public Health, Thailand	2020-01-29	Pilailuk et al A
Thailand/Nonthaburi_59/2020	EPI_ISL_447909		National Institute of Health. Department of medical Sciences, Ministry of Public Health, Thailand	2020-01-13	Pilailuk et al A
Thailand/Nonthaburi_68/2020	EPI_ISL_447910		National Institute of Health. Department of medical Sciences, Ministry of Public Health, Thailand	2020-01-15	Pilailuk et al A
Thailand/Phuket_247/2020	EPI_ISL_447914		National Institute of Health. Department of medical Sciences, Ministry of Public Health, Thailand	2020-01-25	Pilailuk et al A
Thailand/Samutprakarn_840/2020	EPI_ISL_447919		National Institute of Health. Department of medical Sciences, Ministry of Public Health, Thailand	2020-02-04	Pilailuk et al A
Thailand/SI200040-NT/2020	EPI_ISL_437623		Faculty of Medicine	2020-01-08	Rodpan et al
Thailand/SI200383-NT/2020	EPI_ISL_437622		Faculty of Medicine	2020-01-23	Rodpan et al

Thailand/SI200433-NT/2020	EPI_ISL_437621		Faculty of Medicine	2020-01-25	Rodpan et al
Thailand/SI200478-NST/2020	EPI_ISL_437619		Faculty of Medicine	2020-01-26	Rodpan et al
Thailand/SI200615-NT/2020	EPI_ISL_437618		Faculty of Medicine	2020-01-29	Rodpan et al
Thailand/SI200893-NT/2020	EPI_ISL_437624		Faculty of Medicine	2020-02-01	Rodpan et al
Thailand/SI201712-NT/2020	EPI_ISL_437617		Faculty of Medicine	2020-02-03	Rodpan et al
Timor-Leste/TL05/2020	EPI_ISL_456597	National Health Laboratory, Timor-Leste	Microbiological Diagnostic Unit Public Health Laboratory, The Peter Doherty Institute for Infection and Immunity	2020-04-11	Soares da Silva et al
Timor-Leste/TL06/2020	EPI_ISL_456598	National Health Laboratory, Timor-Leste	Microbiological Diagnostic Unit Public Health Laboratory, The Peter Doherty Institute for Infection and Immunity	2020-04-11	Soares da Silva et al
Timor-Leste/TL07/2020	EPI_ISL_456606	National Health Laboratory, Timor-Leste	Microbiological Diagnostic Unit Public Health Laboratory, The Peter Doherty Institute for Infection and Immunity	2020-04-11	Soares da Silva et al
Timor-Leste/TL14/2020	EPI_ISL_456601	National Health Laboratory, Timor-Leste	Microbiological Diagnostic Unit Public Health Laboratory, The Peter Doherty Institute for Infection and Immunity	2020-04-12	Soares da Silva et al
Timor-Leste/TL15/2020	EPI_ISL_456602	National Health Laboratory, Timor-Leste	Microbiological Diagnostic Unit Public Health Laboratory, The Peter Doherty Institute for Infection and Immunity	2020-04-12	Soares da Silva et al
Timor-Leste/TL16/2020	EPI_ISL_456604	National Health Laboratory, Timor-Leste	Microbiological Diagnostic Unit Public Health Laboratory, The Peter Doherty Institute for Infection and Immunity	2020-04-12	Soares da Silva et al
Timor-Leste/TL17/2020	EPI_ISL_456605	National Health Laboratory, Timor-Leste	Microbiological Diagnostic Unit Public Health Laboratory, The Peter Doherty Institute for Infection and Immunity	2020-04-12	Soares da Silva et al
Timor-Leste/TL18/2020	EPI_ISL_456608	National Health Laboratory, Timor-Leste	Microbiological Diagnostic Unit Public Health Laboratory, The Peter Doherty Institute for Infection and Immunity	2020-04-12	Soares da Silva et al
Timor-Leste/TL19/2020	EPI_ISL_456610	National Health Laboratory, Timor-Leste	Microbiological Diagnostic Unit Public Health Laboratory, The Peter Doherty Institute for Infection and Immunity	2020-04-12	Soares da Silva et al
Timor-Leste/TL20/2020	EPI_ISL_456599	National Health Laboratory, Timor-Leste	Microbiological Diagnostic Unit Public Health Laboratory, The Peter Doherty Institute for Infection and Immunity	2020-04-13	Soares da Silva et al
Timor-Leste/TL21/2020	EPI_ISL_456607	National Health Laboratory, Timor-Leste	Microbiological Diagnostic Unit Public Health Laboratory, The Peter Doherty Institute for Infection and Immunity	2020-04-14	Soares da Silva et al
Timor-Leste/TL22/2020	EPI_ISL_456612	National Health Laboratory, Timor-Leste	Microbiological Diagnostic Unit Public Health Laboratory, The Peter Doherty Institute for Infection and Immunity	2020-04-17	Soares da Silva et al
Timor-Leste/TL23/2020	EPI_ISL_456611	National Health Laboratory, Timor-Leste	Microbiological Diagnostic Unit Public Health Laboratory, The Peter Doherty Institute for Infection and Immunity	2020-04-18	Soares da Silva et al
Tunisia/COV0010-12/2020	EPI_ISL_463001		Clinical virology	2020-03-18	Fares et al (https://dx.doi.org/10.22541/au.159137642.26983355)
Tunisia/COV0425/2020	EPI_ISL_463005		Clinical virology	2020-03-27	Fares et al (https://dx.doi.org/10.22541/au.159137642.26983355)
Tunisia/COV0880/2020	EPI_ISL_463002		Clinical virology	2020-03-28	Fares et al (https://dx.doi.org/10.22541/au.159137642.26983355)
Tunisia/COV1339/2020	EPI_ISL_463003		Clinical virology	2020-03-30	Fares et al (https://dx.doi.org/10.22541/au.159137642.26983355)
Tunisia/MHT_1/2020	EPI_ISL_458285		Bundeswehr Institute of Microbiology	2020-03-21	Handrick et al
Tunisia/MHT_2/2020	EPI_ISL_458286		Bundeswehr Institute of Microbiology	2020-03-24	Handrick et al
Turkey/ERAGEM-001/2020	EPI_ISL_424366	Vaccine Research, Development and Application Center, Erciyes University	Gen Era Diagnostics Inc.	2020-03-17	Shaikh Terkis Islam Pavel et al (https://dx.doi.org/10.1101/2020.05.15.095794)
Turkey/GLAB-CoV008/2020	EPI_ISL_427391	Genomic Laboratory (GLAB) (Conjoint lab of Health Directorate of Istanbul and Istanbul Technical University)	Genomic Laboratory (GLAB), Istanbul Technical University	2020-04-13	Ilker Karacan et al (https://dx.doi.org/10.1101/2020.05.15.095794)
Turkey/GLAB-CoV012/2020	EPI_ISL_428346	Genomic Laboratory (GLAB) (Conjoint lab of Health Directorate of Istanbul and Istanbul Technical University)	Genomic Laboratory (GLAB), Istanbul Technical University	2020-04-17	Ilker Karacan et al (https://dx.doi.org/10.1101/2020.05.15.095794)
Turkey/GLAB-CoV013/2020	EPI_ISL_428368	Genomic Laboratory (GLAB) (Conjoint lab of Health Directorate of Istanbul and Istanbul Technical University)	Genomic Laboratory (GLAB), Istanbul Technical University	2020-04-16	Ilker Karacan et al (https://dx.doi.org/10.1101/2020.05.15.095794)
Turkey/HSGM-1004/2020	EPI_ISL_437316	Ministry of Health Turkey	Ministry of Health Turkey	2020-03-25	Fatma Bayrakdar et al B
Turkey/HSGM-10241/2020	EPI_ISL_429871	Ministry of Health Turkey	Ministry of Health Turkey	2020-03-23	Fatma Bayrakdar et al A (https://dx.doi.org/10.1101/2020.05.15.095794)
Turkey/HSGM-1027/2020	EPI_ISL_437317	Ministry of Health Turkey	Ministry of Health Turkey	2020-03-27	Fatma Bayrakdar et al B
Turkey/HSGM-1081/2020	EPI_ISL_437310	Ministry of Health Turkey	Ministry of Health Turkey	2020-03-27	Fatma Bayrakdar et al B
Turkey/HSGM-11640/2020	EPI_ISL_437333	Ministry of Health Turkey	Ministry of Health Turkey	2020-03-25	Fatma Bayrakdar et al B
Turkey/HSGM-12059/2020	EPI_ISL_437335	Ministry of Health Turkey	Ministry of Health Turkey	2020-03-25	Fatma Bayrakdar et al B
Turkey/HSGM-1418/2020	EPI_ISL_437305	Ministry of Health Turkey	Ministry of Health Turkey	2020-03-27	Fatma Bayrakdar et al B
Turkey/HSGM-1428/2020	EPI_ISL_437311	Ministry of Health Turkey	Ministry of Health Turkey	2020-03-27	Fatma Bayrakdar et al B
Turkey/HSGM-1444/2020	EPI_ISL_437318	Ministry of Health Turkey	Ministry of Health Turkey	2020-03-19	Fatma Bayrakdar et al B
Turkey/HSGM-1458/2020	EPI_ISL_437327	Ministry of Health Turkey	Ministry of Health Turkey	2020-03-19	Fatma Bayrakdar et al B
Turkey/HSGM-1476/2020	EPI_ISL_437324	Ministry of Health Turkey	Ministry of Health Turkey	2020-03-19	Fatma Bayrakdar et al B
Turkey/HSGM-1481/2020	EPI_ISL_437323	Ministry of Health Turkey	Ministry of Health Turkey	2020-03-19	Fatma Bayrakdar et al B
Turkey/HSGM-1490/2020	EPI_ISL_437319	Ministry of Health Turkey	Ministry of Health Turkey	2020-03-19	Fatma Bayrakdar et al B
Turkey/HSGM-302/2020	EPI_ISL_437313	Ministry of Health Turkey	Ministry of Health Turkey	2020-03-27	Fatma Bayrakdar et al B
Turkey/HSGM-439/2020	EPI_ISL_437307	Ministry of Health Turkey	Ministry of Health Turkey	2020-03-25	Fatma Bayrakdar et al B
Turkey/HSGM-4598/2020	EPI_ISL_429868	Ministry of Health Turkey	Ministry of Health Turkey	2020-03-17	Fatma Bayrakdar et al A (https://dx.doi.org/10.1101/2020.05.15.095794)
Turkey/HSGM-4701/2020	EPI_ISL_429869	Ministry of Health Turkey	Ministry of Health Turkey	2020-03-17	Fatma Bayrakdar et al A (https://dx.doi.org/10.1101/2020.05.15.095794)
Turkey/HSGM-4706/2020	EPI_ISL_428712	Ministry of Health Turkey	Ministry of Health Turkey	2020-03-17	Fatma Bayrakdar et al A (https://dx.doi.org/10.1101/2020.05.15.095794)
Turkey/HSGM-515/2020	EPI_ISL_437308	Ministry of Health Turkey	Ministry of Health Turkey	2020-03-25	Fatma Bayrakdar et al B
Turkey/HSGM-5516/2020	EPI_ISL_428713	Ministry of Health Turkey	Ministry of Health Turkey	2020-03-18	Fatma Bayrakdar et al A (https://dx.doi.org/10.1101/2020.05.15.095794)
Turkey/HSGM-5624/2020	EPI_ISL_428715	Ministry of Health Turkey	Ministry of Health Turkey	2020-03-18	Fatma Bayrakdar et al A (https://dx.doi.org/10.1101/2020.05.15.095794)
Turkey/HSGM-5769/2020	EPI_ISL_428717	Ministry of Health Turkey	Ministry of Health Turkey	2020-03-19	Fatma Bayrakdar et al A (https://dx.doi.org/10.1101/2020.05.15.095794)
Turkey/HSGM-5770/2020	EPI_ISL_428718	Ministry of Health Turkey	Ministry of Health Turkey	2020-03-19	Fatma Bayrakdar et al A (https://dx.doi.org/10.1101/2020.05.15.095794)
Turkey/HSGM-674/2020	EPI_ISL_437304	Ministry of Health Turkey	Ministry of Health Turkey	2020-03-26	Fatma Bayrakdar et al B
Turkey/HSGM-7718/2020	EPI_ISL_428719	Ministry of Health Turkey	Ministry of Health Turkey	2020-03-21	Fatma Bayrakdar et al A (https://dx.doi.org/10.1101/2020.05.15.095794)
Turkey/HSGM-8010/2020	EPI_ISL_428722	Ministry of Health Turkey	Ministry of Health Turkey	2020-03-22	Fatma Bayrakdar et al A (https://dx.doi.org/10.1101/2020.05.15.095794)
Turkey/HSGM-8964/2020	EPI_ISL_428723	Ministry of Health Turkey	Ministry of Health Turkey	2020-03-22	Fatma Bayrakdar et al A (https://dx.doi.org/10.1101/2020.05.15.095794)
Turkey/HSGM-8975/2020	EPI_ISL_429863	Ministry of Health Turkey	Ministry of Health Turkey	2020-03-22	Fatma Bayrakdar et al A (https://dx.doi.org/10.1101/2020.05.15.095794)
Turkey/IMU-SP-01/2020	EPI_ISL_460618		Physiology	2020-04-15	Pence et al
Turkey/IMU-SP-02/2020	EPI_ISL_460617		Physiology	2020-04-16	Pence et al
Turkey/IMU-SP-03/2020	EPI_ISL_468066		Physiology, Istanbul Medeniyet University	2020-04-16	Pence et al
Turkey/IMU-SP-04/2020	EPI_ISL_460619		Physiology	2020-04-16	Pence et al
Turkey/Istanbul-BezmCoV1/2020	EPI_ISL_457824	Bezmialem Vakif University, Dept Microbiology, Medical School, Fatih, Istanbul, Turkey	Bezmialem Vakif University, Medical School & Beykoz Institute of Life Sciences & Biotechnology	2020-03-24	Mehmet Z. Doymaz et al
Turkey/VETAL1902_1/2020	EPI_ISL_435057	T.C. Sağlık Bakanlığı Adıyaman İl Sağlık Müdürlüğü Adıyaman Eğitim Ve Araştırma Hastanesi	VETAL Animal Health Products Company, BSL3+ Production Laboratuary, Turkey	2020-04-09	Fatma Nilay Tutak et al
Turkey/VETAL1902_2/2020	EPI_ISL_455719	T.C. Sağlık Bakanlığı Adıyaman İl Sağlık Müdürlüğü Adıyaman Eğitim Ve Araştırma Hastanesi	VETAL Animal Health Products Company, BSL3+ Production Laboratuary /Turkey	2020-04-09	Mehmet Turgut et al
Uganda/UG001/2020	EPI_ISL_451183	Uganda Virus Research Institute	MRC/UVRI & LSHTM Uganda Research Unit	2020-03-25	Dan Lule Bugembe et al
Uganda/UG002/2020	EPI_ISL_451184	Uganda Virus Research Institute	MRC/UVRI & LSHTM Uganda Research Unit	2020-03-26	Dan Lule Bugembe et al
Uganda/UG003/2020	EPI_ISL_451185	Uganda Virus Research Institute	MRC/UVRI & LSHTM Uganda Research Unit	2020-03-27	Dan Lule Bugembe et al
Uganda/UG004/2020	EPI_ISL_451186	Uganda Virus Research Institute	MRC/UVRI & LSHTM Uganda Research Unit	2020-03-27	Dan Lule Bugembe et al
Uganda/UG005/2020	EPI_ISL_451187	Uganda Virus Research Institute	MRC/UVRI & LSHTM Uganda Research Unit	2020-03-27	Dan Lule Bugembe et al
Uganda/UG006/2020	EPI_ISL_451188	Uganda Virus Research Institute	MRC/UVRI & LSHTM Uganda Research Unit	2020-03-30	Dan Lule Bugembe et al
Uganda/UG007/2020	EPI_ISL_451189	Uganda Virus Research Institute	MRC/UVRI & LSHTM Uganda Research Unit	2020-03-30	Dan Lule Bugembe et al
Uganda/UG008/2020	EPI_ISL_451190	Uganda Virus Research Institute	MRC/UVRI & LSHTM Uganda Research Unit	2020-03-30	Dan Lule Bugembe et al
Uganda/UG009/2020	EPI_ISL_451191	Uganda Virus Research Institute	MRC/UVRI & LSHTM Uganda Research Unit	2020-03-30	Dan Lule Bugembe et al
Uganda/UG010/2020	EPI_ISL_451192	Uganda Virus Research Institute	MRC/UVRI & LSHTM Uganda Research Unit	2020-03-30	Dan Lule Bugembe et al
Uganda/UG011/2020	EPI_ISL_451193	Uganda Virus Research Institute	MRC/UVRI & LSHTM Uganda Research Unit	2020-03-30	Dan Lule Bugembe et al
Uganda/UG012/2020	EPI_ISL_451194	Uganda Virus Research Institute	MRC/UVRI & LSHTM Uganda Research Unit	2020-03-22	Dan Lule Bugembe et al
Uganda/UG013/2020	EPI_ISL_451195	Uganda Virus Research Institute	MRC/UVRI & LSHTM Uganda Research Unit	2020-03-22	Dan Lule Bugembe et al
Uganda/UG014/2020	EPI_ISL_451196	Uganda Virus Research Institute	MRC/UVRI & LSHTM Uganda Research Unit	2020-03-25	Dan Lule Bugembe et al
Uganda/UG015/2020	EPI_ISL_451197	Uganda Virus Research Institute	MRC/UVRI & LSHTM Uganda Research Unit	2020-04-27	Dan Lule Bugembe et al
Uganda/UG016/2020	EPI_ISL_451198	Uganda Virus Research Institute	MRC/UVRI & LSHTM Uganda Research Unit	2020-04-27	Dan Lule Bugembe et al
Uganda/UG017/2020	EPI_ISL_451199	Uganda Virus Research Institute	MRC/UVRI & LSHTM Uganda Research Unit	2020-04-20	Dan Lule Bugembe et al
Uganda/UG019/2020	EPI_ISL_451201	Uganda Virus Research Institute	MRC/UVRI & LSHTM Uganda Research Unit	2020-04-30	Dan Lule Bugembe et al
Uganda/UG020/2020	EPI_ISL_451202	Uganda Virus Research Institute	MRC/UVRI & LSHTM Uganda Research Unit	2020-05-01	Dan Lule Bugembe et al
UnitedArabEmirates/L0/2020	EPI_ISL_435119	Mohammed Bin Rashid University of Medicine and Health Sciences	Al Jalila Children's Hospital	2020-03-12	Ahmad Abou Tayoun et al B
UnitedArabEmirates/L0184/2020	EPI_ISL_435121	Mohammed Bin Rashid University of Medicine and Health Sciences	Al Jalila Genomics Center	2020-02-25	Ahmad Abou Tayoun et al A (https://dx.doi.org/10.1101/2020.05.06.080606)
UnitedArabEmirates/L0231/2020	EPI_ISL_435122	Mohammed Bin Rashid University of Medicine and Health Sciences	Al Jalila Genomics Center	2020-03-14	Ahmad Abou Tayoun et al A (https://dx.doi.org/10.1101/2020.05.06.080606)
UnitedArabEmirates/L0287/2020	EPI_ISL_469276	Mohammed Bin Rashid University of Medicine and Health Sciences	Al Jalila Genomics Center	2020-03-27	Ahmad Abou Tayoun et al B
UnitedArabEmirates/L0484/2020	EPI_ISL_435123	Mohammed Bin Rashid University of Medicine and Health Sciences	Al Jalila Genomics Center	2020-03-12	Ahmad Abou Tayoun et al A (https://dx.doi.org/10.1101/2020.05.06.080606)
UnitedArabEmirates/L068/2020	EPI_ISL_435120	Mohammed Bin Rashid University of Medicine and Health Sciences	Al Jalila Genomics Center	2020-03-14	Ahmad Abou Tayoun et al A (https://dx.doi.org/10.1101/2020.05.06.080606)
UnitedArabEmirates/L0879/2020	EPI_ISL_435124	Mohammed Bin Rashid University of Medicine and Health Sciences	Al Jalila Genomics Center	2020-03-16	Ahmad Abou Tayoun et al A (https://dx.doi.org/10.1101/2020.05.06.080606)
UnitedArabEmirates/L0881/2020	EPI_ISL_435125	Mohammed Bin Rashid University of Medicine and Health Sciences	Al Jalila Genomics Center	2020-03-16	Ahmad Abou Tayoun et al A (https://dx.doi.org/10.1101/2020.05.06.080606)
UnitedArabEmirates/L0904/2020	EPI_ISL_435126	Mohammed Bin Rashid University of Medicine and Health Sciences	Al Jalila Genomics Center	2020-02-25	Ahmad Abou Tayoun et al A (https://dx.doi.org/10.1101/2020.05.06.080606)
UnitedArabEmirates/L1014/2020	EPI_ISL_435127	Mohammed Bin Rashid University of Medicine and Health Sciences	Al Jalila Genomics Center	2020-03-15	Ahmad Abou Tayoun et al A (https://dx.doi.org/10.1101/2020.05.06.080606)
UnitedArabEmirates/L1076/2020	EPI_ISL_435128	Mohammed Bin Rashid University of Medicine and Health Sciences	Al Jalila Genomics Center	2020-03-15	Ahmad Abou Tayoun et al A (https://dx.doi.org/10.1101/2020.05.06.080606)
UnitedArabEmirates/L1189/2020	EPI_ISL_469277	Mohammed Bin Rashid University of Medicine and Health Sciences	Al Jalila Genomics Center	2020-05-04	Ahmad Abou Tayoun et al B
UnitedArabEmirates/L1758/2020	EPI_ISL_435129	Mohammed Bin Rashid University of Medicine and Health Sciences	Al Jalila Genomics Center	2020-03-10	Ahmad Abou Tayoun et al A (https://dx.doi.org/10.1101/2020.05.06.080606)
UnitedArabEmirates/L2185/2020	EPI_ISL_435130	Mohammed Bin Rashid University of Medicine and Health Sciences	Al Jalila Genomics Center	2020-03-12	Ahmad Abou Tayoun et al A (https://dx.doi.org/10.1101/2020.05.06.080606)
UnitedArabEmirates/L2409/2020	EPI_ISL_435131	Mohammed Bin Rashid University of Medicine and Health Sciences	Al Jalila Genomics Center	2020-02-25	Ahmad Abou Tayoun et al A (https://dx.doi.org/10.1101/2020.05.06.080606)
UnitedArabEmirates/L3779/2020	EPI_ISL_435132	Mohammed Bin Rashid University of Medicine and Health Sciences	Al Jalila Genomics Center	2020-03-15	Ahmad Abou Tayoun et al A (https://dx.doi.org/10.1101/2020.05.06.080606)
UnitedArabEmirates/L4184/2020	EPI_ISL_435133	Mohammed Bin Rashid University of Medicine and Health Sciences	Al Jalila Genomics Center	2020-03-10	Ahmad Abou Tayoun et al A (https://dx.doi.org/10.1101/2020.05.06.080606)
UnitedArabEmirates/L4280/2020	EPI_ISL_435134	Mohammed Bin Rashid University of Medicine and Health Sciences	Al Jalila Genomics Center	2020-02-08	Ahmad Abou Tayoun et al A (https://dx.doi.org/10.1101/2020.05.06.080606)
UnitedArabEmirates/L4682/2020	EPI_ISL_435135	Mohammed Bin Rashid University of Medicine and Health Sciences	Al Jalila Genomics Center	2020-02-25	Ahmad Abou Tayoun et al A (https://dx.doi.org/10.1101/2020.05.06.080606)
UnitedArabEmirates/L4711/2020	EPI_ISL_469278	Mohammed Bin Rashid University of Medicine and Health Sciences	Al Jalila Genomics Center	2020-03-24	Ahmad Abou Tayoun et al B
UnitedArabEmirates/L5621/2020	EPI_ISL_435136	Mohammed Bin Rashid University of Medicine and Health Sciences	Al Jalila Genomics Center	2020-03-11	Ahmad Abou Tayoun et al A (https://dx.doi.org/10.1101/2020.05.06.080606)
UnitedArabEmirates/L5857/2020	EPI_ISL_469279	Mohammed Bin Rashid University of Medicine and Health Sciences	Al Jalila Genomics Center	2020-03-31	Ahmad Abou Tayoun et al B
UnitedArabEmirates/L6599/2020	EPI_ISL_435138	Mohammed Bin Rashid University of Medicine and Health Sciences	Al Jalila Genomics Center	2020-02-25	Ahmad Abou Tayoun et al A (https://dx.doi.org/10.1101/2020.05.06.080606)
UnitedArabEmirates/L6627/2020	EPI_ISL_435139	Mohammed Bin Rashid University of Medicine and Health Sciences	Al Jalila Genomics Center	2020-02-25	Ahmad Abou Tayoun et al A (https://dx.doi.org/10.1101/2020.05.06.080606)
UnitedArabEmirates/L6841/2020	EPI_ISL_469280	Mohammed Bin Rashid University of Medicine and Health Sciences	Al Jalila Genomics Center	2020-03-27	Ahmad Abou Tayoun et al B
UnitedArabEmirates/L7356/2020	EPI_ISL_435140	Mohammed Bin Rashid University of Medicine and Health Sciences	Al Jalila Genomics Center	2020-03-12	Ahmad Abou Tayoun et al A (https://dx.doi.org/10.1101/2020.05.06.080606)
UnitedArabEmirates/L9119/2020	EPI_ISL_469281	Mohammed Bin Rashid University of Medicine and Health Sciences	Al Jalila Genomics Center	2020-03-28	Ahmad Abou Tayoun et al B
UnitedArabEmirates/L9440/2020	EPI_ISL_435141	Mohammed Bin Rashid University of Medicine and Health Sciences	Al Jalila Genomics Center	2020-03-12	Ahmad Abou Tayoun et al A (https://dx.doi.org/10.1101/2020.05.06.080606)

UnitedArabEmirates/L9766/2020	EPI_ISL_435142	Mohammed Bin Rashid University of Medicine and Health Sciences	Al Jalila Genomics Center	2020-03-14	Ahmad Abou Tayoun et al A (https://dx.doi.org/10.1101/2020.05.06.080606)
UnitedArabEmirates/L9768/2020	EPI_ISL_435143	Mohammed Bin Rashid University of Medicine and Health Sciences	Al Jalila Genomics Center	2020-03-18	Ahmad Abou Tayoun et al A (https://dx.doi.org/10.1101/2020.05.06.080606)
Uruguay/Mdeo-1/2020	EPI_ISL_444493	Departamento de Laboratorios de Salud Publica (DLSP, Division Epidemiologia, Ministerio de Salud Publica)	Facultad de Ciencias (Sección Genética Evolutiva, Sección Virología).	2020-03-13	Panzera et al
Uruguay/UY-1/2020	EPI_ISL_426476	Microbial Genomics Laboratory, Institut Pasteur Montevideo	Microbial Genomics Laboratory, Institut Pasteur Montevideo, Uruguay	2020-03-16	Cecilia Salazar et al (https://dx.doi.org/10.1101/2020.05.09.086223)
Uruguay/UY-10/2020	EPI_ISL_429257	Microbial Genomics Laboratory, Institut Pasteur Montevideo	Microbial Genomics Laboratory, Institut Pasteur Montevideo	2020-03-19	Cecilia Salazar et al (https://dx.doi.org/10.1101/2020.05.09.086223)
Uruguay/UY-2/2020	EPI_ISL_426477	Microbial Genomics Laboratory, Institut Pasteur Montevideo	Microbial Genomics Laboratory, Institut Pasteur Montevideo, Uruguay	2020-03-16	Cecilia Salazar et al (https://dx.doi.org/10.1101/2020.05.09.086223)
Uruguay/UY-4/2020	EPI_ISL_426479	Microbial Genomics Laboratory, Institut Pasteur Montevideo	Microbial Genomics Laboratory, Institut Pasteur Montevideo	2020-03-17	Cecilia Salazar et al (https://dx.doi.org/10.1101/2020.05.09.086223)
Uruguay/UY-5/2020	EPI_ISL_426480	Microbial Genomics Laboratory, Institut Pasteur Montevideo	Microbial Genomics Laboratory, Institut Pasteur Montevideo	2020-03-17	Cecilia Salazar et al (https://dx.doi.org/10.1101/2020.05.09.086223)
Uruguay/UY-6/2020	EPI_ISL_426481	Microbial Genomics Laboratory, Institut Pasteur Montevideo, Uruguay	Microbial Genomics Laboratory, Institut Pasteur Montevideo	2020-03-17	Cecilia Salazar et al (https://dx.doi.org/10.1101/2020.05.09.086223)
Uruguay/UY-7/2020	EPI_ISL_426482	Microbial Genomics Laboratory, Institut Pasteur Montevideo, Uruguay	Microbial Genomics Laboratory, Institut Pasteur Montevideo	2020-03-17	Cecilia Salazar et al (https://dx.doi.org/10.1101/2020.05.09.086223)
Uruguay/UY-8/2020	EPI_ISL_426583	Microbial Genomics Laboratory, Institut Pasteur Monteivdeo	Microbial Genomics Laboratory, Institut Pasteur Montevideo, Uruguay	2020-03-17	Cecilia Salazar et al (https://dx.doi.org/10.1101/2020.05.09.086223)
Uruguay/UY-9/2020	EPI_ISL_426584	Microbial Genomics Laboratory, Institut Pasteur Montevideo, Uruguay	Microbial Genomics Laboratory, Institut Pasteur Montevideo, Uruguay	2020-03-19	Cecilia Salazar et al (https://dx.doi.org/10.1101/2020.05.09.086223)
Uruguay/UY-NYUMC844/2020	EPI_ISL_457940	Laboratorio de Biología Molecular Asociación Española Primera en Salud	Departments of Pathology and Medicine, New York University School of Medicine	2020-03-17	Maria Victoria Elizondo et al
Uruguay/UY-NYUMC845/2020	EPI_ISL_457941	Laboratorio de Biología Molecular Asociación Española Primera en Salud	Departments of Pathology and Medicine, New York University School of Medicine	2020-03-17	Maria Victoria Elizondo et al
Uruguay/UY-NYUMC846/2020	EPI_ISL_457942	Laboratorio de Biología Molecular Asociación Española Primera en Salud	Departments of Pathology and Medicine, New York University School of Medicine	2020-03-17	Maria Victoria Elizondo et al
Uruguay/UY-NYUMC847/2020	EPI_ISL_457943	Laboratorio de Biología Molecular Asociación Española Primera en Salud	Departments of Pathology and Medicine, New York University School of Medicine	2020-03-17	Maria Victoria Elizondo et al
Uruguay/UY-NYUMC848/2020	EPI_ISL_457944	Laboratorio de Biología Molecular Asociación Española Primera en Salud	Departments of Pathology and Medicine, New York University School of Medicine	2020-03-17	Maria Victoria Elizondo et al
Uruguay/UY-NYUMC850/2020	EPI_ISL_457946	Laboratorio de Biología Molecular Asociación Española Primera en Salud	Departments of Pathology and Medicine, New York University School of Medicine	2020-03-17	Maria Victoria Elizondo et al
Uruguay/UY-NYUMC852/2020	EPI_ISL_457948	Laboratorio de Biología Molecular Asociación Española Primera en Salud	Departments of Pathology and Medicine, New York University School of Medicine	2020-03-17	Maria Victoria Elizondo et al
Uruguay/UY-NYUMC855/2020	EPI_ISL_457951	Laboratorio de Biología Molecular Asociación Española Primera en Salud	Departments of Pathology and Medicine, New York University School of Medicine	2020-03-21	Maria Victoria Elizondo et al
Uruguay/UY-NYUMC856/2020	EPI_ISL_457952	Laboratorio de Biología Molecular Asociación Española Primera en Salud	Departments of Pathology and Medicine, New York University School of Medicine	2020-03-21	Maria Victoria Elizondo et al
Uruguay/UY-NYUMC857/2020	EPI_ISL_457953	Laboratorio de Biología Molecular Asociación Española Primera en Salud	Departments of Pathology and Medicine, New York University School of Medicine	2020-03-23	Maria Victoria Elizondo et al
Uruguay/UY-NYUMC858/2020	EPI_ISL_457954	Laboratorio de Biología Molecular Asociación Española Primera en Salud	Departments of Pathology and Medicine, New York University School of Medicine	2020-03-23	Maria Victoria Elizondo et al
Uruguay/UY-NYUMC860/2020	EPI_ISL_457956	Laboratorio de Biología Molecular Asociación Española Primera en Salud	Departments of Pathology and Medicine, New York University School of Medicine	2020-03-25	Maria Victoria Elizondo et al
Uruguay/UY-NYUMC861/2020	EPI_ISL_457957	Laboratorio de Biología Molecular Asociación Española Primera en Salud	Departments of Pathology and Medicine, New York University School of Medicine	2020-03-25	Maria Victoria Elizondo et al
Uruguay/UY-NYUMC862/2020	EPI_ISL_457958	Laboratorio de Biología Molecular Asociación Española Primera en Salud	Departments of Pathology and Medicine, New York University School of Medicine	2020-03-25	Maria Victoria Elizondo et al
Uruguay/UY-NYUMC864/2020	EPI_ISL_457960	Laboratorio de Biología Molecular Asociación Española Primera en Salud	Departments of Pathology and Medicine, New York University School of Medicine	2020-03-28	Maria Victoria Elizondo et al
Uruguay/UY-NYUMC866/2020	EPI_ISL_457962	Laboratorio de Biología Molecular Asociación Española Primera en Salud	Departments of Pathology and Medicine, New York University School of Medicine	2020-03-30	Maria Victoria Elizondo et al
Uruguay/UY-NYUMC867/2020	EPI_ISL_457963	Laboratorio de Biología Molecular Asociación Española Primera en Salud	Departments of Pathology and Medicine, New York University School of Medicine	2020-03-28	Maria Victoria Elizondo et al
Uruguay/UY-NYUMC868/2020	EPI_ISL_457964	Laboratorio de Biología Molecular Asociación Española Primera en Salud	Departments of Pathology and Medicine, New York University School of Medicine	2020-04-01	Maria Victoria Elizondo et al
Uruguay/UY-NYUMC869/2020	EPI_ISL_457965	Laboratorio de Biología Molecular Asociación Española Primera en Salud	Departments of Pathology and Medicine, New York University School of Medicine	2020-04-03	Maria Victoria Elizondo et al
Uruguay/UY-NYUMC871/2020	EPI_ISL_457967	Laboratorio de Biología Molecular Asociación Española Primera en Salud	Departments of Pathology and Medicine, New York University School of Medicine	2020-04-09	Maria Victoria Elizondo et al
Uruguay/UY-NYUMC872/2020	EPI_ISL_457968	Laboratorio de Biología Molecular Asociación Española Primera en Salud	Departments of Pathology and Medicine, New York University School of Medicine	2020-04-11	Maria Victoria Elizondo et al
Uruguay/UY-NYUMC873/2020	EPI_ISL_457969	Laboratorio de Biología Molecular Asociación Española Primera en Salud	Departments of Pathology and Medicine, New York University School of Medicine	2020-04-14	Maria Victoria Elizondo et al
Uruguay/UY-NYUMC874/2020	EPI_ISL_457970	Laboratorio de Biología Molecular Asociación Española Primera en Salud	Departments of Pathology and Medicine, New York University School of Medicine	2020-04-14	Maria Victoria Elizondo et al
Uruguay/UY-NYUMC875/2020	EPI_ISL_457971	Laboratorio de Biología Molecular Asociación Española Primera en Salud	Departments of Pathology and Medicine, New York University School of Medicine	2020-04-19	Maria Victoria Elizondo et al
Uruguay/UY-NYUMC876/2020	EPI_ISL_457972	Laboratorio de Biología Molecular Asociación Española Primera en Salud	Departments of Pathology and Medicine, New York University School of Medicine	2020-04-21	Maria Victoria Elizondo et al
Uruguay/UY-NYUMC877/2020	EPI_ISL_457973	Laboratorio de Biología Molecular Asociación Española Primera en Salud	Departments of Pathology and Medicine, New York University School of Medicine	2020-04-22	Maria Victoria Elizondo et al
USA/CA_2602/2020	EPI_ISL_419554	California Department of Public Health	Pathogen Discovery, Respiratory Viruses Branch, Division of Viral Diseases, Centers for Disease Control and Prevention	2020-02-26	Ying Tao et al B
USA/CA-CDPH-UC1/2020	EPI_ISL_413557	California Department of Public Health	Chiu Laboratory, University of California, San Francisco	2020-02-26	Xianding Deng et al A (https://dx.doi.org/10.1126/science.abb9263)
USA/CA-CDPH-UC3/2020	EPI_ISL_413559	California Department of Public Health	Chiu Laboratory, University of California, San Francisco	2020-02-27	Xianding Deng et al A (https://dx.doi.org/10.1126/science.abb9263)
USA/CA-CZB-1095/2020	EPI_ISL_444053	UCSF Clinical Microbiology Laboratory	Chan-Zuckerberg Biohub	2020-04-27	CZB Cliahub Consortium et al
USA/CA-CZB-1140/2020	EPI_ISL_447891	University of California, Davis	Chan-Zuckerberg Biohub	2020-04-01	CZB Cliahub Consortium et al
USA/CA-CZB-1219/2020	EPI_ISL_450468	Stanford clinical virology lab	Chan-Zuckerberg Biohub	2020-03-20	Benjamin Pinksy et al
USA/CA-CZB-1400/2020	EPI_ISL_468380	Alameda County Public Health Lab	Chan-Zuckerberg Biohub	2020-04-19	CZB Cliahub Consortium et al
USA/CA-CZB-1484/2020	EPI_ISL_468435	County of San Luis Obispo Public Health Laboratory	Chan-Zuckerberg Biohub	2020-05-01	CZB Cliahub Consortium et al
USA/CA-CZB-1622/2020	EPI_ISL_468551	San Joaquin County Public Health Lab	Chan-Zuckerberg Biohub	2020-05-07	CZB Cliahub Consortium et al
USA/CA-SCCPHD-UC104/2020	EPI_ISL_435583	Santa Clara County Public Health Department	Chiu Laboratory, University of California, San Francisco	2020-02-29	Xianding Deng et al B
USA/CA-SCCPHD-UC106/2020	EPI_ISL_435585	Santa Clara County Public Health Department	Chiu Laboratory, University of California, San Francisco	2020-02-29	Xianding Deng et al B
USA/CA-SR0106/2020	EPI_ISL_445102	UC San Diego Center for Advanced Laboratory Medicine	Andersen lab at Scripps Research	2020-03-14	SEARCH Alliance San Diego with David Pride et al
USA/CA2/2020	EPI_ISL_406036	California Department of Public Health	Pathogen Discovery, Respiratory Viruses Branch, Division of Viral Diseases, Centers for Disease Control and Prevention	2020-01-22	Anna Uehara et al (https://dx.doi.org/10.1101/2020.03.09.20032896)
USA/CA5/2020	EPI_ISL_408010	California Department of Health	Pathogen Discovery, Respiratory Viruses Branch, Division of Viral Diseases, Centers for Disease Control and Prevention	2020-01-29	Ying Tao et al A (https://dx.doi.org/10.1101/2020.03.09.20032896)
USA/CA6/2020	EPI_ISL_410044	California Department of Public Health	Pathogen Discovery, Respiratory Viruses Branch, Division of Viral Diseases, Centers for Disease Control and Prevention	2020-01-27	Jing Zhang et al (https://dx.doi.org/10.1101/2020.03.09.20032896)
USA/CT-Yale-034/2020	EPI_ISL_419524	Yale Clinical Virology Laboratory	Grubaugh Lab - Yale School of Public Health	2020-03-15	Joseph Fauver et al
USA/CT-Yale-076/2020	EPI_ISL_428384	Yale COVID-19 Biorepository	Grubaugh Lab - Yale School of Public Health	2020-04-04	Joseph Fauver et al
USA/FL-BPHL-0063/2020	EPI_ISL_445006	Florida Bureau of Public Health Laboratories	Florida Bureau of Public Health Laboratories	2020-04-16	Sarah Schmedes et al
USA/IL-UW-360/2020	EPI_ISL_450714	University of Wisconsin-Madison AIDS Vaccine Research Laboratories	University of Wisconsin-Madison AIDS Vaccine Research Laboratories	2020-04-18	Gage Moreno et al
USA/IL1/2020	EPI_ISL_404253	IL Department of Public Health Chicago Laboratory	Pathogen Discovery, Respiratory Viruses Branch, Division of Viral Diseases, Centers for Disease Control and Prevention	2020-01-21	Ying Tao et al A (https://dx.doi.org/10.1101/2020.03.09.20032896)
USA/LA-EVTL095/2020	EPI_ISL_451256	LSUHS Emerging Viral Threat Laboratory	Microbial Genome Sequencing Center	2020-05-06	Jeremy P. Kamil et al
USA/LA-SR0292/2020	EPI_ISL_445147	Robert Garry lab	Andersen lab at Scripps Research	2020-04-07	Allison Smither et al
USA/LA-SR0299/2020	EPI_ISL_445154	Robert Garry lab	Andersen lab at Scripps Research	2020-04-07	Allison Smither et al
USA/MA_1355/2020	EPI_ISL_424913	MA State Public Health Laboratory	Pathogen Discovery, Respiratory Viruses Branch, Division of Viral Diseases, Centers for Disease Control and Prevention	2020-03-05	Ying Tao et al B
USA/MA_3626/2020	EPI_ISL_424918	MA State Public Health Laboratory	Pathogen Discovery, Respiratory Viruses Branch, Division of Viral Diseases, Centers for Disease Control and Prevention	2020-03-06	Ying Tao et al B
USA/MA-MGH-00264/2020	EPI_ISL_460160	Massachusetts General Hospital	Infectious Disease Program, Broad Institute of Harvard and MIT	2020-04-01	Lemieux et al (https://virological.org/t/introduction-and-spread-of-sars-cov-2-in-the-greater-boston-area/503)
USA/MA-MGH-00446/2020	EPI_ISL_460352	Massachusetts General Hospital	Infectious Disease Program, Broad Institute of Harvard and MIT	2020-04-01	Lemieux et al (https://virological.org/t/introduction-and-spread-of-sars-cov-2-in-the-greater-boston-area/503)
USA/MA-MGH-00664/2020	EPI_ISL_460307	Massachusetts General Hospital	Infectious Disease Program, Broad Institute of Harvard and MIT	2020-04-04	Lemieux et al (https://virological.org/t/introduction-and-spread-of-sars-cov-2-in-the-greater-boston-area/503)
USA/MI-MDHHS-SC20289/2020	EPI_ISL_452263	Michigan Department of Health and Human Services, Bureau of Laboratories	Michigan Department of Health and Human Services, Bureau of Laboratories	2020-05-01	Blankenship HM et al
USA/MI-MDHHS-SC20348/2020	EPI_ISL_450627	Michigan Department of Health and Human Services, Bureau of Laboratories	Michigan Department of Health and Human Services, Bureau of Laboratories	2020-03-29	Blankenship HM et al
USA/MI-MDHHS-SC20364/2020	EPI_ISL_450575	Michigan Department of Health and Human Services, Bureau of Laboratories	Michigan Department of Health and Human Services, Bureau of Laboratories	2020-03-14	Blankenship HM et al
USA/MI-MDHHS-SC20400/2020	EPI_ISL_452274	Michigan Department of Health and Human Services, Bureau of Laboratories	Michigan Department of Health and Human Services, Bureau of Laboratories	2020-03-24	Blankenship HM et al
USA/MI-MDHHS-SC20638/2020	EPI_ISL_462720	Michigan Department of Health and Human Services, Bureau of Laboratories	Michigan Department of Health and Human Services, Bureau of Laboratories	2020-04-26	Blankenship HM et al
USA/MI-QDX-70/2020	EPI_ISL_467872	Quest Diagnostics	Quest Diagnostics	2020-03-15	Anderson et al
USA/MN-MDH-1007/2020	EPI_ISL_462852	Minnesota Department of Health, Public Health Laboratory	Minnesota Department of Health, Public Health Laboratory	2020-04-30	Matt Plumb et al
USA/MN-MDH-1028/2020	EPI_ISL_462873	Minnesota Department of Health, Public Health Laboratory	Minnesota Department of Health, Public Health Laboratory	2020-05-07	Matt Plumb et al
USA/MN-MDH-1040/2020	EPI_ISL_462885	Minnesota Department of Health, Public Health Laboratory	Minnesota Department of Health, Public Health Laboratory	2020-05-07	Matt Plumb et al
USA/MN-MDH-1042/2020	EPI_ISL_462887	Minnesota Department of Health, Public Health Laboratory	Minnesota Department of Health, Public Health Laboratory	2020-05-07	Matt Plumb et al
USA/MN-MDH-1070/2020	EPI_ISL_470750	Minnesota Department of Health, Public Health Laboratory	Minnesota Department of Health, Public Health Laboratory	2020-06-03	Matt Plumb et al
USA/MN-MDH-1071/2020	EPI_ISL_470751	Minnesota Department of Health, Public Health Laboratory	Minnesota Department of Health, Public Health Laboratory	2020-06-03	Matt Plumb et al
USA/MN-MDH-1073/2020	EPI_ISL_470753	Minnesota Department of Health, Public Health Laboratory	Minnesota Department of Health, Public Health Laboratory	2020-06-03	Matt Plumb et al
USA/MN-MDH-1074/2020	EPI_ISL_470754	Minnesota Department of Health, Public Health Laboratory	Minnesota Department of Health, Public Health Laboratory	2020-06-03	Matt Plumb et al
USA/MN-MDH-1075/2020	EPI_ISL_470755	Minnesota Department of Health, Public Health Laboratory	Minnesota Department of Health, Public Health Laboratory	2020-06-03	Matt Plumb et al
USA/MN-MDH-1076/2020	EPI_ISL_470756	Minnesota Department of Health, Public Health Laboratory	Minnesota Department of Health, Public Health Laboratory	2020-06-03	Matt Plumb et al
USA/MN-MDH-1078/2020	EPI_ISL_470758	Minnesota Department of Health, Public Health Laboratory	Minnesota Department of Health, Public Health Laboratory	2020-06-03	Matt Plumb et al
USA/MN-MDH-1079/2020	EPI_ISL_470759	Minnesota Department of Health, Public Health Laboratory	Minnesota Department of Health, Public Health Laboratory	2020-06-03	Matt Plumb et al
USA/MN-MDH-1082/2020	EPI_ISL_470762	Minnesota Department of Health, Public Health Laboratory	Minnesota Department of Health, Public Health Laboratory	2020-06-03	Matt Plumb et al
USA/MN-MDH-1084/2020	EPI_ISL_470764	Minnesota Department of Health, Public Health Laboratory	Minnesota Department of Health, Public Health Laboratory	2020-06-03	Matt Plumb et al
USA/MN-MDH-1085/2020	EPI_ISL_470765	Minnesota Department of Health, Public Health Laboratory	Minnesota Department of Health, Public Health Laboratory	2020-06-03	Matt Plumb et al
USA/MN-MDH-1088/2020	EPI_ISL_470768	Minnesota Department of Health, Public Health Laboratory	Minnesota Department of Health, Public Health Laboratory	2020-06-03	Matt Plumb et al
USA/MN-MDH-1089/2020	EPI_ISL_470769	Minnesota Department of Health, Public Health Laboratory	Minnesota Department of Health, Public Health Laboratory	2020-06-03	Matt Plumb et al
USA/MN-MDH-1090/2020	EPI_ISL_470770	Minnesota Department of Health, Public Health Laboratory	Minnesota Department of Health, Public Health Laboratory	2020-06-03	Matt Plumb et al
USA/MN-MDH-1091/2020	EPI_ISL_470771	Minnesota Department of Health, Public Health Laboratory	Minnesota Department of Health, Public Health Laboratory	2020-06-03	Matt Plumb et al
USA/MN-MDH-1092/2020	EPI_ISL_470772	Minnesota Department of Health, Public Health Laboratory	Minnesota Department of Health, Public Health Laboratory	2020-06-03	Matt Plumb et al
USA/MN-MDH-1093/2020	EPI_ISL_470773	Minnesota Department of Health, Public Health Laboratory	Minnesota Department of Health, Public Health Laboratory	2020-06-03	Matt Plumb et al
USA/MN-MDH-1094/2020	EPI_ISL_470774	Minnesota Department of Health, Public Health Laboratory	Minnesota Department of Health, Public Health Laboratory	2020-06-03	Matt Plumb et al
USA/MN-MDH-1095/2020	EPI_ISL_470775	Minnesota Department of Health, Public Health Laboratory	Minnesota Department of Health, Public Health Laboratory	2020-06-03	Matt Plumb et al
USA/MN-MDH-1098/2020	EPI_ISL_470778	Minnesota Department of Health, Public Health Laboratory	Minnesota Department of Health, Public Health Laboratory	2020-06-03	Matt Plumb et al
USA/MN-MDH-1099/2020	EPI_ISL_470779	Minnesota Department of Health, Public Health Laboratory	Minnesota Department of Health, Public Health Laboratory	2020-06-03	Matt Plumb et al
USA/MN-MDH-1100/2020	EPI_ISL_470780	Minnesota Department of Health, Public Health Laboratory	Minnesota Department of Health, Public Health Laboratory	2020-06-03	Matt Plumb et al
USA/MN-MDH-1102/2020	EPI_ISL_470782	Minnesota Department of Health, Public Health Laboratory	Minnesota Department of Health, Public Health Laboratory	2020-06-03	Matt Plumb et al
USA/MN-MDH-1103/2020	EPI_ISL_470783	Minnesota Department of Health, Public Health Laboratory	Minnesota Department of Health, Public Health Laboratory	2020-06-03	Matt Plumb et al
USA/MN-MDH-1104/2020	EPI_ISL_470784	Minnesota Department of Health, Public Health Laboratory	Minnesota Department of Health, Public Health Laboratory	2020-06-03	Matt Plumb et al
USA/MN-MDH-1105/2020	EPI_ISL_470785	Minnesota Department of Health, Public Health Laboratory	Minnesota Department of Health, Public Health Laboratory	2020-06-03	Matt Plumb et al
USA/MN-MDH-1107/2020	EPI_ISL_470787	Minnesota Department of Health, Public Health Laboratory	Minnesota Department of Health, Public Health Laboratory	2020-06-03	Matt Plumb et al
USA/MN-MDH-1108/2020	EPI_ISL_470788	Minnesota Department of Health, Public Health Laboratory	Minnesota Department of Health, Public Health Laboratory	2020-06-03	Matt Plumb et al
USA/MN4-MDH4/2020	EPI_ISL_417189	Minnesota Department of Health, Public Health Laboratory	Minnesota Department of Health, Public Health Laboratory	2020-03-09	Matt Plumb et al
USA/MO-WUSTL035/2020	EPI_ISL_467302	Washington University in St. Louis	Washington University in St. Louis	2020-06-08	David Wang et al
USA/MO-WUSTL055/2020	EPI_ISL_467304	Washington University in St. Louis	Washington University in St. Louis	2020-06-08	David Wang et al
USA/NE-10933/2020	EPI_ISL_466669	Nebraska Public Health Laboratory	UNMC COVID-19 Response Team	2020-05-04	UNMC COVID-19 Response Team et al

USA/NE-10971/2020	EPI_ISL_466659	Nebraska Public Health Laboratory	UNMC COVID-19 Response Team	2020-05-04	UNMC COVID-19 Response Team et al
USA/NE-11120/2020	EPI_ISL_466672	Nebraska Public Health Laboratory	UNMC COVID-19 Response Team	2020-05-04	UNMC COVID-19 Response Team et al
USA/NJ-QDX-74/2020	EPI_ISL_467883	Quest Diagnostics	Quest Diagnostics	2020-03-14	Anderson et al
USA/NM-UNM-00148/2020	EPI_ISL_467569	New Mexico Department of Health Scientific Laboratory Division	Center for Global Health, University of New Mexico Health Sciences Center	2020-04-03	Daryl Domman et al
USA/NY-NYUMC151/2020	EPI_ISL_424958	NYU Langone Health	Departments of Pathology and Medicine, New York University School of Medicine	2020-04-01	Maria Aguero-Rosenfeld et al A (https://dx.doi.org/10.1101/2020.04.15.20064931)
USA/NY-NYUMC192/2020	EPI_ISL_427487	NYU Langone Health	Departments of Pathology and Medicine, New York University School of Medicine	2020-04-01	Maria Aguero-Rosenfeld et al A (https://dx.doi.org/10.1101/2020.04.15.20064931)
USA/NY-NYUMC463/2020	EPI_ISL_435722	NYU Langone Health	Departments of Pathology and Medicine, New York University School of Medicine	2020-04-16	Maria Aguero-Rosenfeld et al B
USA/NY-NYUMC598/2020	EPI_ISL_444715	NYU Langone Health	Departments of Pathology and Medicine, New York University School of Medicine	2020-04-03	Maria Aguero-Rosenfeld et al B
USA/NY-NYUMC641/2020	EPI_ISL_444758	NYU Langone Health	Departments of Pathology and Medicine, New York University School of Medicine	2020-04-03	Maria Aguero-Rosenfeld et al B
USA/NY-WCMP10H01P/2020	EPI_ISL_427580	NewYork-Presbyterian & Mason Lab	Mason Lab	2020-03-15	Daniel J. Butler et al (https://dx.doi.org/10.1101/2020.04.20.048066)
USA/NY-WCMP12C09P/2020	EPI_ISL_427602	NewYork-Presbyterian & Mason Lab	Mason Lab	2020-03-16	Daniel J. Butler et al (https://dx.doi.org/10.1101/2020.04.20.048066)
USA/PA-MGSC35-05/2020	EPI_ISL_436484	UPMC Clinical Laboratory	Microbial Genome Sequencing Center, Microbial Genomic Epidemiological Laboratory	2020-03-27	Dan Snyder et al
USA/PA-MGSC40-05/2020	EPI_ISL_436488	UPMC Clinical Laboratory	Microbial Genome Sequencing Center, Microbial Genomic Epidemiological Laboratory	2020-03-26	Dan Snyder et al
USA/RI_0520/2020	EPI_ISL_419553	RI State Health Laboratories	Pathogen Discovery, Respiratory Viruses Branch, Division of Viral Diseases, Centers for Disease Control and Prevention	2020-02-28	Ying Tao et al B
USA/TX_2039/2020	EPI_ISL_420796	Texas DSHS Lab Services	Pathogen Discovery, Respiratory Viruses Branch, Division of Viral Diseases, Centers for Disease Control and Prevention	2020-02-29	Krista Queen et al A
USA/TX-HMH0146/2020	EPI_ISL_434845	Houston Methodist Hospital	Houston Methodist Hospital	2020-03-22	S. Wesley Long et al (https://dx.doi.org/10.1101/2020.05.01.072652)
USA/TX-HMH050/2020	EPI_ISL_434759	Houston Methodist Hospital	Houston Methodist Hospital	2020-03-16	S. Wesley Long et al (https://dx.doi.org/10.1101/2020.05.01.072652)
USA/TX-HMH059/2020	EPI_ISL_434768	Houston Methodist Hospital	Houston Methodist Hospital	2020-03-17	S. Wesley Long et al (https://dx.doi.org/10.1101/2020.05.01.072652)
USA/UT-014/2020	EPI_ISL_417964	Utah Public Health Laboratory	Utah Public Health Laboratory	2020-03-13	Erin Young et al
USA/UT-03876/2020	EPI_ISL_450562	Utah Public Health Laboratory	Utah Public Health Laboratory	2020-04-13	Erin Young et al
USA/VA-DCLS-0221/2020	EPI_ISL_452799	Virginia DCLS	Virginia DCLS	2020-04-03	Virginia DCLS et al
USA/VA-DCLS-0379/2020	EPI_ISL_463135	Virginia DCLS	Virginia DCLS	2020-05-13	Virginia DCLS et al
USA/WA-NH23/2020	EPI_ISL_418790	WA State Department of Health	Pathogen Discovery, Respiratory Viruses Branch, Division of Viral Diseases, Centers for Disease Control and Prevention	2020-03-13	Ying Tao et al B
USA/WA-S1027/2020	EPI_ISL_463517	Washington State Department of Health	Seattle Flu Study	2020-05-01	Chu et al et al A
USA/WA-S1032/2020	EPI_ISL_463522	Washington State Department of Health	Seattle Flu Study	2020-05-01	Chu et al et al A
USA/WA-S1036/2020	EPI_ISL_463526	Washington State Department of Health	Seattle Flu Study	2020-05-03	Chu et al et al A
USA/WA-S107/2020	EPI_ISL_417160	Washington State Department of Health	Seattle Flu Study	2020-02-29	Chu etl al et al (https://doi.dx.org/10.1101/2020.04.02.20051417)
USA/WA-S1111/2020	EPI_ISL_463601	Washington State Department of Health	Seattle Flu Study	2020-05-04	Chu et al et al A
USA/WA-S1119/2020	EPI_ISL_463609	Washington State Department of Health	Seattle Flu Study	2020-05-04	Chu et al et al A
USA/WA-S1143/2020	EPI_ISL_463633	Washington State Department of Health	Seattle Flu Study	2020-05-05	Chu et al et al A
USA/WA-S1156/2020	EPI_ISL_463646	Washington State Department of Health	Seattle Flu Study	2020-05-04	Chu et al et al A
USA/WA-S1173/2020	EPI_ISL_463663	Washington State Department of Health	Seattle Flu Study	2020-05-04	Chu et al et al A
USA/WA-S1178/2020	EPI_ISL_463668	Washington State Department of Health	Seattle Flu Study	2020-05-06	Chu et al et al A
USA/WA-S119/2020	EPI_ISL_417172	Washington State Department of Health	Seattle Flu Study	2020-02-29	Chu etl al et al (https://doi.dx.org/10.1101/2020.04.02.20051417)
USA/WA-S1190/2020	EPI_ISL_463680	Washington State Department of Health	Seattle Flu Study	2020-05-05	Chu et al et al A
USA/WA-S1191/2020	EPI_ISL_463681	Washington State Department of Health	Seattle Flu Study	2020-05-05	Chu et al et al A
USA/WA-S1215/2020	EPI_ISL_463705	Washington State Department of Health	Seattle Flu Study	2020-05-07	Chu et al et al A
USA/WA-S2/2020	EPI_ISL_413456	Seattle Flu Study	Seattle Flu Study	2020-02-20	Chu et al et al B (https://doi.dx.org/10.1101/2020.04.02.20051417)
USA/WA-S234/2020	EPI_ISL_430272	Washington State Department of Health	Seattle Flu Study	2020-02-28	Chu et al et al A
USA/WA-S257/2020	EPI_ISL_430295	Washington State Department of Health	Seattle Flu Study	2020-02-25	Chu et al et al A
USA/WA-S277/2020	EPI_ISL_430131	Seattle Flu Study	Seattle Flu Study	2020-02-22	Chu et al et al A
USA/WA-S281/2020	EPI_ISL_430135	Seattle Flu Study	Seattle Flu Study	2020-02-29	Chu et al et al A
USA/WA-S30/2020	EPI_ISL_417083	Washington State Department of Health	Seattle Flu Study	2020-02-28	Chu etl al et al (https://doi.dx.org/10.1101/2020.04.02.20051417)
USA/WA-S397/2020	EPI_ISL_434157	Washington State Department of Health	Seattle Flu Study	2020-03-25	Chu et al et al A
USA/WA-S44/2020	EPI_ISL_417097	Washington State Department of Health	Seattle Flu Study	2020-02-28	Chu etl al et al (https://doi.dx.org/10.1101/2020.04.02.20051417)
USA/WA-S5/2020	EPI_ISL_416460	Seattle Flu Study	Seattle Flu Study	2020-02-29	Chu et al et al B (https://doi.dx.org/10.1101/2020.04.02.20051417)
USA/WA-S55/2020	EPI_ISL_417108	Washington State Department of Health	Seattle Flu Study	2020-02-29	Chu etl al et al (https://doi.dx.org/10.1101/2020.04.02.20051417)
USA/WA-S6/2020	EPI_ISL_416461	Seattle Flu Study	Seattle Flu Study	2020-02-29	Chu et al et al B (https://doi.dx.org/10.1101/2020.04.02.20051417)
USA/WA-S65/2020	EPI_ISL_417118	Washington State Department of Health	Seattle Flu Study	2020-03-03	Chu etl al et al (https://doi.dx.org/10.1101/2020.04.02.20051417)
USA/WA-S731/2020	EPI_ISL_449901	Washington State Department of Health	Seattle Flu Study	2020-04-11	Chu et al et al A
USA/WA-S801/2020	EPI_ISL_449971	Washington State Department of Health	Seattle Flu Study	2020-04-16	Chu et al et al A
USA/WA-S84/2020	EPI_ISL_417137	Washington State Department of Health	Seattle Flu Study	2020-02-21	Chu etl al et al (https://doi.dx.org/10.1101/2020.04.02.20051417)
USA/WA-S89/2020	EPI_ISL_417142	Washington State Department of Health	Seattle Flu Study	2020-02-29	Chu etl al et al (https://doi.dx.org/10.1101/2020.04.02.20051417)
USA/WA-S917/2020	EPI_ISL_463407	Washington State Department of Health	Seattle Flu Study	2020-04-27	Chu et al et al A
USA/WA-S92/2020	EPI_ISL_417145	Washington State Department of Health	Seattle Flu Study	2020-02-29	Chu etl al et al (https://doi.dx.org/10.1101/2020.04.02.20051417)
USA/WA-S939/2020	EPI_ISL_463429	Washington State Department of Health	Seattle Flu Study	2020-04-23	Chu et al et al A
USA/WA-S95/2020	EPI_ISL_417148	Washington State Department of Health	Seattle Flu Study	2020-02-28	Chu etl al et al (https://doi.dx.org/10.1101/2020.04.02.20051417)
USA/WA-S954/2020	EPI_ISL_463444	Washington State Department of Health	Seattle Flu Study	2020-05-01	Chu et al et al A
USA/WA-S962/2020	EPI_ISL_463452	Washington State Department of Health	Seattle Flu Study	2020-05-01	Chu et al et al A
USA/WA-S97/2020	EPI_ISL_417150	Washington State Department of Health	Seattle Flu Study	2020-02-28	Chu etl al et al (https://doi.dx.org/10.1101/2020.04.02.20051417)
USA/WA-UW-10107/2020	EPI_ISL_461466	UW Virology Lab	UW Virology Lab	2020-05-29	Pavitra Roychoudhury et al B
USA/WA-UW-10108/2020	EPI_ISL_461467	UW Virology Lab	UW Virology Lab	2020-05-29	Pavitra Roychoudhury et al B
USA/WA-UW-3895/2020	EPI_ISL_427203	UW Virology Lab	UW Virology Lab	2020-03-27	Pavitra Roychoudhury et al B
USA/WA-UW-5189/2020	EPI_ISL_430873	UW Virology Lab	UW Virology Lab	2020-04-01	Pavitra Roychoudhury et al B
USA/WA-UW-5203/2020	EPI_ISL_430899	UW Virology Lab	UW Virology Lab	2020-04-01	Pavitra Roychoudhury et al B
USA/WA-UW-5717/2020	EPI_ISL_430913	UW Virology Lab	UW Virology Lab	2020-04-06	Pavitra Roychoudhury et al B
USA/WA-UW-5947/2020	EPI_ISL_430932	UW Virology Lab	UW Virology Lab	2020-04-07	Pavitra Roychoudhury et al B
USA/WA-UW101/2020	EPI_ISL_416639	UW Virology Lab	UW Virology Lab	2020-03-10	Pavitra Roychoudhury et al A (https://doi.dx.org/10.1101/2020.04.02.20051417)
USA/WA1/2020	EPI_ISL_404895	Providence Regional Medical Center	Division of Viral Diseases, Centers for Disease Control and Prevention	2020-01-19	Queen et al (https://dx.doi.org/10.1101/2020.03.02.972935)
USA/WA4-UW2/2020	EPI_ISL_413455	Washington State Public Health Lab	University of Washington Virology Lab	2020-02-28	Pavitra Roychoudhury et al A (https://doi.dx.org/10.1101/2020.04.02.20051417)
USA/WI-GMF-02274/2020	EPI_ISL_436897	Gundersen Molecular Diagnostics Laboratory	Kabara Cancer Research Institute	2020-05-03	Craig S. Richmond et al (https://www.medrxiv.org/content/10.1101/2020.06.08.20125534v1)
USA/WI-GMF-02381/2020	EPI_ISL_436900	Gundersen Molecular Diagnostics Laboratory	Kabara Cancer Research Institute	2020-05-04	Craig S. Richmond et al (https://www.medrxiv.org/content/10.1101/2020.06.08.20125534v1)
USA/WI-GMF-M00001/2020	EPI_ISL_455573	Gundersen Clinical Microbiology Laboratory	Kabara Cancer Research Institute	2020-05-10	Craig S. Richmond et al (https://www.medrxiv.org/content/10.1101/2020.06.08.20125534v1)
USA/WI-UW-112/2020	EPI_ISL_425169	University of Wisconsin-Madison AIDS Vaccine Research Laboratories	University of Wisconsin-Madison AIDS Vaccine Research Laboratories	2020-03-30	Gage Moreno et al
USA/WI-UW-223/2020	EPI_ISL_428319	University of Wisconsin-Madison AIDS Vaccine Research Laboratories	University of Wisconsin-Madison AIDS Vaccine Research Laboratories	2020-03-26	Gage Moreno et al
USA/WI-UW-329/2020	EPI_ISL_436623	University of Wisconsin-Madison AIDS Vaccine Research Laboratories	University of Wisconsin-Madison AIDS Vaccine Research Laboratories	2020-04-25	Gage Moreno et al
USA/WI1/2020	EPI_ISL_408670	Wisconsin Department of Health Services	Pathogen Discovery, Respiratory Viruses Branch, Division of Viral Diseases, Centers for Disease Control and Prevention	2020-01-31	Jing Zhang et al (https://dx.doi.org/10.1101/2020.03.09.20032896)
Vietnam/19-01S/2020	EPI_ISL_418269		Microbiology and Immunology department	2020-01-22	Cao et al (http://biorxiv.org/lookup/doi/10.1101/2020.04.01.019463)
Vietnam/19-02S/2020	EPI_ISL_418267		Microbiology and Immunology department	2020-01-22	Nguyen et al
Vietnam/39607/2020	EPI_ISL_416428	National Influenza Center, National Institute of Hygiene and Epidemiology (NIHE)	National Influenza Center, National Institute of Hygiene and Epidemiology (NIHE)	2020-03-07	Le Quynh Mai et al
Vietnam/CM296/2020	EPI_ISL_416431	National Influenza Center, National Institute of Hygiene and Epidemiology (NIHE)	National Influenza Center, National Institute of Hygiene and Epidemiology (NIHE)	2020-03-06	Le Quynh Mai et al
Vietnam/HCMC-05004/2020	EPI_ISL_450738	OUCRU/HTD	OUCRU/HTD	2020-03-27	Nguyen Van Vinh Chau et al
Vietnam/HCMC-35016/2020	EPI_ISL_450741	OUCRU/HTD	OUCRU/HTD	2020-03-20	Nguyen Van Vinh Chau et al
Vietnam/HCMC-35018/2020	EPI_ISL_450742	OUCRU/HTD	OUCRU/HTD	2020-03-25	Nguyen Van Vinh Chau et al
Vietnam/HCMC-35019/2020	EPI_ISL_450743	OUCRU/HTD	OUCRU/HTD	2020-03-24	Nguyen Van Vinh Chau et al
Vietnam/HCMC-35020/2020	EPI_ISL_450744	OUCRU/HTD	OUCRU/HTD	2020-03-25	Nguyen Van Vinh Chau et al
Vietnam/HCMC-35021/2020	EPI_ISL_450745	OUCRU/HTD	OUCRU/HTD	2020-03-26	Nguyen Van Vinh Chau et al
Vietnam/IBT-14Mar/2020	EPI_ISL_448222	Pasteur Insitute Ho Chi Minh City	National Key Laboratory of Gene Technology, Institute of Biotechnology, Vietnam Academy of Science and Technology	2020-03-15	Le Tung Lam et al (https://www.biorxiv.org/content/10.1101/2020.06.12.149377v1)
Vietnam/VNHN_022/2020	EPI_ISL_435303	National Hospital of Tropical Diseases	Oxford University Clinical Research Unit, Hanoi, Vietnam	2020-03-07	Nguyen Thi Tam et al
Vietnam/VNHN_026/2020	EPI_ISL_435305	National Hospital of Tropical Diseases	Oxford University Clinical Research Unit, Hanoi, Vietnam	2020-03-07	Nguyen Thi Tam et al
Vietnam/VNHN_0299/2020	EPI_ISL_435308	National Hospital of Tropical Diseases	Oxford University Clinical Research Unit, Hanoi, Vietnam	2020-03-09	Nguyen Thi Tam et al
Vietnam/VNHN_0300/2020	EPI_ISL_435310	National Hospital of Tropical Diseases	Oxford University Clinical Research Unit, Hanoi, Vietnam	2020-03-09	Nguyen Thi Tam et al
Vietnam/VNHN_0418/2020	EPI_ISL_435311	National Hospital of Tropical Diseases	Oxford University Clinical Research Unit, Hanoi, Vietnam	2020-03-10	Nguyen Thi Tam et al
Vietnam/VNHN_0764/2020	EPI_ISL_435314	National Hospital of Tropical Diseases	Oxford University Clinical Research Unit, Hanoi, Vietnam	2020-03-13	Nguyen Thi Tam et al
Vietnam/VNHN_0837/2020	EPI_ISL_435315	National Hospital of Tropical Diseases	Oxford University Clinical Research Unit, Hanoi, Vietnam	2020-03-15	Nguyen Thi Tam et al
Vietnam/VNHN_1072/2020	EPI_ISL_455717	National Hospital of Tropical Diseases	Oxford University Clinical Research Unit, Hanoi, Vietnam	2020-03-17	Nguyen Thi Tam et al
Vietnam/VNHN_1098/2020	EPI_ISL_455694	National Hospital of Tropical Diseases	Oxford University Clinical Research Unit, Hanoi, Vietnam	2020-03-18	Nguyen Thi Tam et al
Vietnam/VNHN_1226/2020	EPI_ISL_455698	National Hospital of Tropical Diseases	Oxford University Clinical Research Unit, Hanoi, Vietnam	2020-03-19	Nguyen Thi Tam et al
Vietnam/VNHN_148/2020	EPI_ISL_455713	National Hospital of Tropical Diseases	Oxford University Clinical Research Unit, Hanoi, Vietnam	2020-03-06	Nguyen Thi Tam et al
Vietnam/VNHN_1492/2020	EPI_ISL_455699	National Hospital of Tropical Diseases	Oxford University Clinical Research Unit, Hanoi, Vietnam	2020-03-21	Nguyen Thi Tam et al
Vietnam/VNHN_1713/2020	EPI_ISL_455701	National Hospital of Tropical Diseases	Oxford University Clinical Research Unit, Hanoi, Vietnam	2020-03-23	Nguyen Thi Tam et al
Vietnam/VNHN_2406/2020	EPI_ISL_455700	National Hospital of Tropical Diseases	Oxford University Clinical Research Unit, Hanoi, Vietnam	2020-03-25	Nguyen Thi Tam et al
Vietnam/VNHN_301/2020	EPI_ISL_455714	National Hospital of Tropical Diseases	Oxford University Clinical Research Unit, Hanoi, Vietnam	2020-03-09	Nguyen Thi Tam et al
Vietnam/VNHN_302/2020	EPI_ISL_455715	National Hospital of Tropical Diseases	Oxford University Clinical Research Unit, Hanoi, Vietnam	2020-03-09	Nguyen Thi Tam et al
Vietnam/VNHN_3085/2020	EPI_ISL_455703	National Hospital of Tropical Diseases	Oxford University Clinical Research Unit, Hanoi, Vietnam	2020-03-27	Nguyen Thi Tam et al
Vietnam/VNHN_3096/2020	EPI_ISL_455702	National Hospital of Tropical Diseases	Oxford University Clinical Research Unit, Hanoi, Vietnam	2020-03-27	Nguyen Thi Tam et al
Vietnam/VNHN_3629/2020	EPI_ISL_455706	National Hospital of Tropical Diseases	Oxford University Clinical Research Unit, Hanoi, Vietnam	2020-03-30	Nguyen Thi Tam et al
Vietnam/VNHN_3916/2020	EPI_ISL_455704	National Hospital of Tropical Diseases	Oxford University Clinical Research Unit, Hanoi, Vietnam	2020-03-31	Nguyen Thi Tam et al
Vietnam/VNHN_4189/2020	EPI_ISL_455707	National Hospital of Tropical Diseases	Oxford University Clinical Research Unit, Hanoi, Vietnam	2020-04-02	Nguyen Thi Tam et al
Vietnam/VNHN_4806/2020	EPI_ISL_455711	National Hospital of Tropical Diseases	Oxford University Clinical Research Unit, Hanoi, Vietnam	2020-04-08	Nguyen Thi Tam et al
Vietnam/VNHN_4851/2020	EPI_ISL_455709	National Hospital of Tropical Diseases	Oxford University Clinical Research Unit, Hanoi, Vietnam	2020-04-09	Nguyen Thi Tam et al
Vietnam/VNHN_4864/2020	EPI_ISL_455708	National Hospital of Tropical Diseases	Oxford University Clinical Research Unit, Hanoi, Vietnam	2020-04-10	Nguyen Thi Tam et al

Vietnam/VNHN_4868/2020	EPI_ISL_455710	National Hospital of Tropical Diseases	Oxford University Clinical Research Unit, Hanoi, Vietnam	2020-04-09	Nguyen Thi Tam et al
Vietnam/VNHN_4875/2020	EPI_ISL_455712	National Hospital of Tropical Diseases	Oxford University Clinical Research Unit, Hanoi, Vietnam	2020-04-10	Nguyen Thi Tam et al
Vietnam/VNHN_4958/2020	EPI_ISL_455718	National Hospital of Tropical Diseases	Oxford University Clinical Research Unit, Hanoi, Vietnam	2020-04-10	Nguyen Thi Tam et al
Wales/PHWC-25E1A/2020	EPI_ISL_422331	Wales Specialist Virology Centre	Public Health Wales Microbiology Cardiff	2020-03-25	Catherine Moore et al (https://virological.org/t/preliminary-analysis-of-sars-cov-2-importation-establishment-of-uk-transmission-lineages/507)
Wales/PHWC-28A8C/2020	EPI_ISL_445394	Wales Specialist Virology Centre	Public Health Wales Microbiology Cardiff	2020-04-03	Catherine Moore et al (https://virological.org/t/preliminary-analysis-of-sars-cov-2-importation-establishment-of-uk-transmission-lineages/507)
Wales/PHWC-3474A/2020	EPI_ISL_446968	Wales Specialist Virology Centre	Public Health Wales Microbiology Cardiff	2020-04-20	Catherine Moore et al (https://virological.org/t/preliminary-analysis-of-sars-cov-2-importation-establishment-of-uk-transmission-lineages/507)
Wuhan/HB-WH1-122/2020	EPI_ISL_454904	Wuhan Chain Medical Labs (CMLabs)	State Key Laboratory of Biotherapy of Sichuan University	2020-03-02	Baowen Du et al
Wuhan/HB-WH1-127/2020	EPI_ISL_454908	Wuhan Chain Medical Labs (CMLabs)	State Key Laboratory of Biotherapy of Sichuan University	2020-03-02	Baowen Du et al
Wuhan/HB-WH1-135/2020	EPI_ISL_454912	Wuhan Chain Medical Labs (CMLabs)	State Key Laboratory of Biotherapy of Sichuan University	2020-02-25	Baowen Du et al
Wuhan/HB-WH1-138/2020	EPI_ISL_454915	Wuhan Chain Medical Labs (CMLabs)	State Key Laboratory of Biotherapy of Sichuan University	2020-02-24	Baowen Du et al
Wuhan/HB-WH2-147/2020	EPI_ISL_455371	Wuhan Chain Medical Labs (CMLabs)	State Key Laboratory of Biotherapy of Sichuan University	2020-03-02	Baowen Du et al
Wuhan/HB-WH2-161/2020	EPI_ISL_454929	Wuhan Chain Medical Labs (CMLabs)	State Key Laboratory of Biotherapy of Sichuan University	2020-02-28	Baowen Du et al
Wuhan/HB-WH2-166/2020	EPI_ISL_454932	Wuhan Chain Medical Labs (CMLabs)	State Key Laboratory of Biotherapy of Sichuan University	2020-02-11	Baowen Du et al
Wuhan/HB-WH3-171/2020	EPI_ISL_454937	Wuhan Chain Medical Labs (CMLabs)	State Key Laboratory of Biotherapy of Sichuan University	2020-02-11	Baowen Du et al
Wuhan/HB-WH3-178/2020	EPI_ISL_455378	Wuhan Chain Medical Labs (CMLabs)	State Key Laboratory of Biotherapy of Sichuan University	2020-03-13	Baowen Du et al
Wuhan/HB-WH3-183/2020	EPI_ISL_454944	Wuhan Chain Medical Labs (CMLabs)	State Key Laboratory of Biotherapy of Sichuan University	2020-03-12	Baowen Du et al
Wuhan/HB-WH3-190/2020	EPI_ISL_455384	Wuhan Chain Medical Labs (CMLabs)	State Key Laboratory of Biotherapy of Sichuan University	2020-03-16	Baowen Du et al
Wuhan/HB-WH4-196/2020	EPI_ISL_454950	Wuhan Chain Medical Labs (CMLabs)	State Key Laboratory of Biotherapy of Sichuan University	2020-03-20	Baowen Du et al
Wuhan/HB-WH4-205/2020	EPI_ISL_454955	Wuhan Chain Medical Labs (CMLabs)	State Key Laboratory of Biotherapy of Sichuan University	2020-03-01	Baowen Du et al
Wuhan/HB-WH4-206/2020	EPI_ISL_454956	Wuhan Chain Medical Labs (CMLabs)	State Key Laboratory of Biotherapy of Sichuan University	2020-03-27	Baowen Du et al
Wuhan/HB-WH5-218/2020	EPI_ISL_455390	Wuhan Chain Medical Labs (CMLabs)	State Key Laboratory of Biotherapy of Sichuan University	2020-03-19	Baowen Du et al
Wuhan/HB-WH5-235/2020	EPI_ISL_455394	Wuhan Chain Medical Labs (CMLabs)	State Key Laboratory of Biotherapy of Sichuan University	2020-03-16	Baowen Du et al
Wuhan/HB-WHCM-108/2020	EPI_ISL_454991	Wuhan Chain Medical Labs (CMLabs)	State Key Laboratory of Biotherapy of Sichuan University	2020-03-02	Baowen Du et al
Wuhan/HB-WHCM-112/2020	EPI_ISL_455405	Wuhan Chain Medical Labs (CMLabs)	State Key Laboratory of Biotherapy of Sichuan University	2020-03-02	Baowen Du et al
Wuhan/HB-WHCM-120/2020	EPI_ISL_454998	Wuhan Chain Medical Labs (CMLabs)	State Key Laboratory of Biotherapy of Sichuan University	2020-02-24	Baowen Du et al
Wuhan/HB-WHCM2-277/2020	EPI_ISL_455411	Wuhan Chain Medical Labs (CMLabs)	State Key Laboratory of Biotherapy of Sichuan University	2020-03-02	Baowen Du et al
Wuhan/HBCDC-HB-01/2019	EPI_ISL_402132	Wuhan Jinyintan Hospital	Hubei Provincial Center for Disease Control and Prevention	2019-12-30	Bin Fang et al (https://dx.doi.org/10.1101/2020.03.04.976662)
Wuhan/HBCDC-HB-02/2019	EPI_ISL_412898	Wuhan Jinyintan Hospital	Hubei Provincial Center for Disease Control and Prevention	2019-12-30	Bin Fang et al (https://dx.doi.org/10.1101/2020.03.04.976662)
Wuhan/HBCDC-HB-03/2019	EPI_ISL_412899	Wuhan Jinyintan Hospital	Hubei Provincial Center for Disease Control and Prevention	2019-12-30	Bin Fang et al (https://dx.doi.org/10.1101/2020.03.04.976662)
Wuhan/HBCDC-HB-03/2020	EPI_ISL_412979	Union Hospital of Tongji Medical College, Huazhong University of Science and Technology	Hubei Provincial Center for Disease Control and Prevention	2020-01-18	Bin Fang et al (https://dx.doi.org/10.1101/2020.03.04.976662)
Wuhan/Hu-1/2019	EPI_ISL_402125		National Institute for Communicable Disease Control and Prevention (ICDC) Chinese Center for Disease Control and Prevention (China CDC)	2019-12-26	Zhang et al (https://dx.doi.org/10.1038/s41586-020-2008-3)
Wuhan/IPBCAMS-WH-01/2019	EPI_ISL_402123	Institute of Pathogen Biology, Chinese Academy of Medical Sciences & Peking Union Medical College	Institute of Pathogen Biology, Chinese Academy of Medical Sciences & Peking Union Medical College	2019-12-24	Lili Ren et al A
Wuhan/IPBCAMS-WH-03/2019	EPI_ISL_403930	Institute of Pathogen Biology, Chinese Academy of Medical Sciences & Peking Union Medical College	Institute of Pathogen Biology, Chinese Academy of Medical Sciences & Peking Union Medical College	2019-12-30	Lili Ren et al B (https://dx.doi.org/10.1093/cid/ciaa203/5780800)
Wuhan/IPBCAMS-WH-04/2019	EPI_ISL_403929	Institute of Pathogen Biology, Chinese Academy of Medical Sciences & Peking Union Medical College	Institute of Pathogen Biology, Chinese Academy of Medical Sciences & Peking Union Medical College	2019-12-30	Lili Ren et al B (https://dx.doi.org/10.1093/cid/ciaa203/5780800)
Wuhan/IVDC-HB-01/2019	EPI_ISL_402119	National Institute for Viral Disease Control and Prevention, China CDC	National Institute for Viral Disease Control and Prevention, China CDC	2019-12-30	Wenjie Tan et al A (https://dx.doi.org/10.1056/NEJMoa2001017)
Wuhan/IVDC-HB-05/2019	EPI_ISL_402121	National Institute for Viral Disease Control and Prevention, China CDC	National Institute for Viral Disease Control and Prevention, China CDC	2019-12-30	Wenjie Tan et al A (https://dx.doi.org/10.1056/NEJMoa2001017)
Wuhan/IVDC-HB-GX02/2019	EPI_ISL_434534	National Institute for Viral Disease Control and Prevention, China CDC	National Institute for Viral Disease Control and Prevention, China CDC, Yunnan Provincial CDC	2019-12-30	Wenjie Tan et al B
Wuhan/WH01/2019	EPI_ISL_406798	General Hospital of Central Theater Command of People's Liberation Army of China	BGI & Institute of Microbiology, Chinese Academy of Sciences & Shandong First Medical University & Shandong Academy of Medical Sciences & General Hospital of Central Theater Command of People's Liberation Army of China	2019-12-26	Weijun Chen et al (https://dx.doi.org/10.1016/S0140-6736(20)30251-8)
Wuhan/WIV02/2019	EPI_ISL_402127	Wuhan Jinyintan Hospital	Wuhan Institute of Virology, Chinese Academy of Sciences	2019-12-30	Peng Zhou et al
Wuhan/WIV04/2019	EPI_ISL_402124	Wuhan Jinyintan Hospital	Wuhan Institute of Virology, Chinese Academy of Sciences	2019-12-30	Peng Zhou et al
Wuhan/WIV05/2019	EPI_ISL_402128	Wuhan Jinyintan Hospital	Wuhan Institute of Virology, Chinese Academy of Sciences	2019-12-30	Peng Zhou et al
Wuhan/WIV06/2019	EPI_ISL_402129	Wuhan Jinyintan Hospital	Wuhan Institute of Virology, Chinese Academy of Sciences	2019-12-30	Peng Zhou et al
Wuhan/WIV07/2019	EPI_ISL_402130	Wuhan Jinyintan Hospital	Wuhan Institute of Virology, Chinese Academy of Sciences	2019-12-30	Peng Zhou et al
Wuhan/YB012504/2020	EPI_ISL_450489		CAS Key Laboratory of Special Pathogens and Biosafety and Center for Emerging Infectious Diseases	2020-01-25	Si et al
Zhejiang/CN1/2020	EPI_ISL_455683		Department of Microbiology	2020-01-22	Gao et al
Zhejiang/CN3/2020	EPI_ISL_455686		Department of Microbiology	2020-01-26	Gao et al
Zhejiang/OS3/2020	EPI_ISL_455691		Department of Microbiology	2020-03-24	Gao et al

Supplementary Table S4. Accession numbers of sequences used for the phylogeny shown in figure 2.

Strain	gisaid_epi_isl	Originating Lab	Submitting Lab	Collection Data	Author
Austria/CeMM0003/2020	EPI_ISL_419656	Center for Virology, Medical University of Vienna	Bergthaler laboratory, CeMM Research Center for Molecular Medicine of the Austrian Academy of Sciences	2020-02-26	Alexandra Popa et al
Austria/CeMM0004/2020	EPI_ISL_419657	Center for Virology, Medical University of Vienna	Bergthaler laboratory, CeMM Research Center for Molecular Medicine of the Austrian Academy of Sciences	2020-03-03	Alexandra Popa et al
Austria/CeMM0005/2020	EPI_ISL_419658	Center for Virology, Medical University of Vienna	Bergthaler laboratory, CeMM Research Center for Molecular Medicine of the Austrian Academy of Sciences	2020-03-06	Alexandra Popa et al
Austria/CeMM0006/2020	EPI_ISL_419659	Center for Virology, Medical University of Vienna	Bergthaler laboratory, CeMM Research Center for Molecular Medicine of the Austrian Academy of Sciences	2020-03-10	Alexandra Popa et al
Austria/CeMM0007/2020	EPI_ISL_419660	Center for Virology, Medical University of Vienna	Bergthaler laboratory, CeMM Research Center for Molecular Medicine of the Austrian Academy of Sciences	2020-03-13	Alexandra Popa et al
Austria/CeMM0008/2020	EPI_ISL_419661	Center for Virology, Medical University of Vienna	Bergthaler laboratory, CeMM Research Center for Molecular Medicine of the Austrian Academy of Sciences	2020-03-10	Alexandra Popa et al
Austria/CeMM0010/2020	EPI_ISL_419663	Center for Virology, Medical University of Vienna	Bergthaler laboratory, CeMM Research Center for Molecular Medicine of the Austrian Academy of Sciences	2020-03-15	Alexandra Popa et al
Austria/CeMM0011/2020	EPI_ISL_419664	Center for Virology, Medical University of Vienna	Bergthaler laboratory, CeMM Research Center for Molecular Medicine of the Austrian Academy of Sciences	2020-03-23	Alexandra Popa et al
Austria/CeMM0012/2020	EPI_ISL_419665	Center for Virology, Medical University of Vienna	Bergthaler laboratory, CeMM Research Center for Molecular Medicine of the Austrian Academy of Sciences	2020-03-10	Alexandra Popa et al
Austria/CeMM0013/2020	EPI_ISL_419666	Center for Virology, Medical University of Vienna	Bergthaler laboratory, CeMM Research Center for Molecular Medicine of the Austrian Academy of Sciences	2020-03-11	Alexandra Popa et al
Austria/CeMM0014/2020	EPI_ISL_419667	Center for Virology, Medical University of Vienna	Bergthaler laboratory, CeMM Research Center for Molecular Medicine of the Austrian Academy of Sciences	2020-03-13	Alexandra Popa et al
Austria/CeMM0015/2020	EPI_ISL_419668	Center for Virology, Medical University of Vienna	Bergthaler laboratory, CeMM Research Center for Molecular Medicine of the Austrian Academy of Sciences	2020-03-13	Alexandra Popa et al
Austria/CeMM0016/2020	EPI_ISL_419669	Center for Virology, Medical University of Vienna	Bergthaler laboratory, CeMM Research Center for Molecular Medicine of the Austrian Academy of Sciences	2020-03-16	Alexandra Popa et al
Austria/CeMM0017/2020	EPI_ISL_419670	Center for Virology, Medical University of Vienna	Bergthaler laboratory, CeMM Research Center for Molecular Medicine of the Austrian Academy of Sciences	2020-03-16	Alexandra Popa et al
Austria/CeMM0018/2020	EPI_ISL_419671	Center for Virology, Medical University of Vienna	Bergthaler laboratory, CeMM Research Center for Molecular Medicine of the Austrian Academy of Sciences	2020-03-19	Alexandra Popa et al
Austria/CeMM0019/2020	EPI_ISL_419672	Center for Virology, Medical University of Vienna	Bergthaler laboratory, CeMM Research Center for Molecular Medicine of the Austrian Academy of Sciences	2020-03-19	Alexandra Popa et al
Austria/CeMM0020/2020	EPI_ISL_419673	Center for Virology, Medical University of Vienna	Bergthaler laboratory, CeMM Research Center for Molecular Medicine of the Austrian Academy of Sciences	2020-03-22	Alexandra Popa et al
Austria/CeMM0021/2020	EPI_ISL_419674	Center for Virology, Medical University of Vienna	Bergthaler laboratory, CeMM Research Center for Molecular Medicine of the Austrian Academy of Sciences	2020-03-24	Alexandra Popa et al
Austria/CeMM0023/2020	EPI_ISL_437913	Institut für Virologie am Department für Hygiene, Mikrobiologie und Public Health	Bergthaler laboratory, CeMM Research Center for Molecular Medicine of the Austrian Academy of Sciences	2020-03-09	Alexandra Popa et al
Austria/CeMM0024/2020	EPI_ISL_437914	Institut für Virologie am Department für Hygiene, Mikrobiologie und Public Health	Bergthaler laboratory, CeMM Research Center for Molecular Medicine of the Austrian Academy of Sciences	2020-03-09	Alexandra Popa et al
Austria/CeMM0025/2020	EPI_ISL_437915	Institut für Virologie am Department für Hygiene, Mikrobiologie und Public Health	Bergthaler laboratory, CeMM Research Center for Molecular Medicine of the Austrian Academy of Sciences	2020-03-09	Alexandra Popa et al
Austria/CeMM0026/2020	EPI_ISL_437916	Institut für Virologie am Department für Hygiene, Mikrobiologie und Public Health	Bergthaler laboratory, CeMM Research Center for Molecular Medicine of the Austrian Academy of Sciences	2020-03-19	Alexandra Popa et al
Austria/CeMM0028/2020	EPI_ISL_437917	Institut für Virologie am Department für Hygiene, Mikrobiologie und Public Health	Bergthaler laboratory, CeMM Research Center for Molecular Medicine of the Austrian Academy of Sciences	2020-03-09	Alexandra Popa et al
Austria/CeMM0029/2020	EPI_ISL_437918	Institut für Virologie am Department für Hygiene, Mikrobiologie und Public Health	Bergthaler laboratory, CeMM Research Center for Molecular Medicine of the Austrian Academy of Sciences	2020-03-09	Alexandra Popa et al
Austria/CeMM0030/2020	EPI_ISL_437919	Institut für Virologie am Department für Hygiene, Mikrobiologie und Public Health	Bergthaler laboratory, CeMM Research Center for Molecular Medicine of the Austrian Academy of Sciences	2020-03-09	Alexandra Popa et al
Austria/CeMM0031/2020	EPI_ISL_437920	Institut für Virologie am Department für Hygiene, Mikrobiologie und Public Health	Bergthaler laboratory, CeMM Research Center for Molecular Medicine of the Austrian Academy of Sciences	2020-03-09	Alexandra Popa et al
Austria/CeMM0032/2020	EPI_ISL_437921	Institut für Virologie am Department für Hygiene, Mikrobiologie und Public Health	Bergthaler laboratory, CeMM Research Center for Molecular Medicine of the Austrian Academy of Sciences	2020-03-12	Alexandra Popa et al
Austria/CeMM0033/2020	EPI_ISL_437922	Institut für Virologie am Department für Hygiene, Mikrobiologie und Public Health	Bergthaler laboratory, CeMM Research Center for Molecular Medicine of the Austrian Academy of Sciences	2020-03-12	Alexandra Popa et al
Austria/CeMM0034/2020	EPI_ISL_437923	Institut für Virologie am Department für Hygiene, Mikrobiologie und Public Health	Bergthaler laboratory, CeMM Research Center for Molecular Medicine of the Austrian Academy of Sciences	2020-03-10	Alexandra Popa et al
Austria/CeMM0035/2020	EPI_ISL_437924	Institut für Virologie am Department für Hygiene, Mikrobiologie und Public Health	Bergthaler laboratory, CeMM Research Center for Molecular Medicine of the Austrian Academy of Sciences	2020-03-07	Alexandra Popa et al
Austria/CeMM0036/2020	EPI_ISL_437925	Institut für Virologie am Department für Hygiene, Mikrobiologie und Public Health	Bergthaler laboratory, CeMM Research Center for Molecular Medicine of the Austrian Academy of Sciences	2020-03-09	Alexandra Popa et al
Austria/CeMM0037/2020	EPI_ISL_437926	Institut für Virologie am Department für Hygiene, Mikrobiologie und Public Health	Bergthaler laboratory, CeMM Research Center for Molecular Medicine of the Austrian Academy of Sciences	2020-03-09	Alexandra Popa et al
Austria/CeMM0038/2020	EPI_ISL_437927	Institut für Virologie am Department für Hygiene, Mikrobiologie und Public Health	Bergthaler laboratory, CeMM Research Center for Molecular Medicine of the Austrian Academy of Sciences	2020-03-09	Alexandra Popa et al
Austria/CeMM0039/2020	EPI_ISL_437928	Institut für Virologie am Department für Hygiene, Mikrobiologie und Public Health	Bergthaler laboratory, CeMM Research Center for Molecular Medicine of the Austrian Academy of Sciences	2020-03-10	Alexandra Popa et al
Austria/CeMM0041/2020	EPI_ISL_437929	Institut für Virologie am Department für Hygiene, Mikrobiologie und Public Health	Bergthaler laboratory, CeMM Research Center for Molecular Medicine of the Austrian Academy of Sciences	2020-03-05	Alexandra Popa et al
Austria/CeMM0042/2020	EPI_ISL_437930	Institut für Virologie am Department für Hygiene, Mikrobiologie und Public Health	Bergthaler laboratory, CeMM Research Center for Molecular Medicine of the Austrian Academy of Sciences	2020-03-09	Alexandra Popa et al
Austria/CeMM0045/2020	EPI_ISL_437932	Institut für Virologie am Department für Hygiene, Mikrobiologie und Public Health	Bergthaler laboratory, CeMM Research Center for Molecular Medicine of the Austrian Academy of Sciences	2020-02-24	Alexandra Popa et al
Austria/CeMM0047/2020	EPI_ISL_437933	Universitaetsklinik für Innere Medizin II Innsbruck	Bergthaler laboratory, CeMM Research Center for Molecular Medicine of the Austrian Academy of Sciences	2020-03-25	Alexandra Popa et al
Austria/CeMM0048/2020	EPI_ISL_437934	Universitaetsklinik für Innere Medizin II Innsbruck	Bergthaler laboratory, CeMM Research Center for Molecular Medicine of the Austrian Academy of Sciences	2020-03-25	Alexandra Popa et al
Austria/CeMM0052/2020	EPI_ISL_437935	Universitaetsklinik für Innere Medizin II Innsbruck	Bergthaler laboratory, CeMM Research Center for Molecular Medicine of the Austrian Academy of Sciences	2020-03-30	Alexandra Popa et al
Austria/CeMM0053/2020	EPI_ISL_437936	Universitaetsklinik für Innere Medizin II Innsbruck	Bergthaler laboratory, CeMM Research Center for Molecular Medicine of the Austrian Academy of Sciences	2020-03-30	Alexandra Popa et al
Austria/CeMM0058/2020	EPI_ISL_437938	Universitaetsklinik für Innere Medizin II Innsbruck	Bergthaler laboratory, CeMM Research Center for Molecular Medicine of the Austrian Academy of Sciences	2020-04-03	Alexandra Popa et al
Austria/CeMM0067/2020	EPI_ISL_437939	Universitaetsklinik für Innere Medizin II Innsbruck	Bergthaler laboratory, CeMM Research Center for Molecular Medicine of the Austrian Academy of Sciences	2020-04-06	Alexandra Popa et al
Austria/CeMM0068/2020	EPI_ISL_437940	Universitaetsklinik für Innere Medizin II Innsbruck	Bergthaler laboratory, CeMM Research Center for Molecular Medicine of the Austrian Academy of Sciences	2020-03-12	Alexandra Popa et al
Austria/CeMM0071/2020	EPI_ISL_437941	Universitaetsklinik für Innere Medizin II Innsbruck	Bergthaler laboratory, CeMM Research Center for Molecular Medicine of the Austrian Academy of Sciences	2020-03-11	Alexandra Popa et al
Austria/CeMM0073/2020	EPI_ISL_437942	Universitaetsklinik für Innere Medizin II Innsbruck	Bergthaler laboratory, CeMM Research Center for Molecular Medicine of the Austrian Academy of Sciences	2020-03-11	Alexandra Popa et al

Austria/CeMM0074/2020	EPI_ISL_437943	Universitaetsklinik für Innere Medizin II Innsbruck	Bergthaler laboratory, CeMM Research Center for Molecular Medicine of the Austrian Academy of Sciences	2020-03-17	Alexandra Popa et al
Austria/CeMM0076/2020	EPI_ISL_437944	Universitaetsklinik für Innere Medizin II Innsbruck	Bergthaler laboratory, CeMM Research Center for Molecular Medicine of the Austrian Academy of Sciences	2020-03-17	Alexandra Popa et al
Austria/CeMM0077/2020	EPI_ISL_437945	Universitaetsklinik für Innere Medizin II Innsbruck	Bergthaler laboratory, CeMM Research Center for Molecular Medicine of the Austrian Academy of Sciences	2020-03-17	Alexandra Popa et al
Austria/CeMM0078/2020	EPI_ISL_437946	Universitaetsklinik für Innere Medizin II Innsbruck	Bergthaler laboratory, CeMM Research Center for Molecular Medicine of the Austrian Academy of Sciences	2020-03-24	Alexandra Popa et al
Austria/CeMM0083/2020	EPI_ISL_437948	Universitaetsklinik für Innere Medizin II Innsbruck	Bergthaler laboratory, CeMM Research Center for Molecular Medicine of the Austrian Academy of Sciences	2020-03-24	Alexandra Popa et al
Austria/CeMM0084/2020	EPI_ISL_437949	Universitaetsklinik für Innere Medizin II Innsbruck	Bergthaler laboratory, CeMM Research Center for Molecular Medicine of the Austrian Academy of Sciences	2020-03-24	Alexandra Popa et al
Austria/CeMM0086/2020	EPI_ISL_437950	Universitaetsklinik für Innere Medizin II Innsbruck	Bergthaler laboratory, CeMM Research Center for Molecular Medicine of the Austrian Academy of Sciences	2020-03-24	Alexandra Popa et al
Austria/CeMM0088/2020	EPI_ISL_437951	Universitaetsklinik für Innere Medizin II Innsbruck	Bergthaler laboratory, CeMM Research Center for Molecular Medicine of the Austrian Academy of Sciences	2020-03-24	Alexandra Popa et al
Austria/CeMM0089/2020	EPI_ISL_437952	Universitaetsklinik für Innere Medizin II Innsbruck	Bergthaler laboratory, CeMM Research Center for Molecular Medicine of the Austrian Academy of Sciences	2020-03-24	Alexandra Popa et al
Austria/CeMM0090/2020	EPI_ISL_437953	Universitaetsklinik für Innere Medizin II Innsbruck	Bergthaler laboratory, CeMM Research Center for Molecular Medicine of the Austrian Academy of Sciences	2020-03-25	Alexandra Popa et al
Austria/CeMM0091/2020	EPI_ISL_437954	Universitaetsklinik für Innere Medizin II Innsbruck	Bergthaler laboratory, CeMM Research Center for Molecular Medicine of the Austrian Academy of Sciences	2020-03-25	Alexandra Popa et al
Austria/CeMM0094/2020	EPI_ISL_437955	Universitaetsklinik für Innere Medizin II Innsbruck	Bergthaler laboratory, CeMM Research Center for Molecular Medicine of the Austrian Academy of Sciences	2020-03-23	Alexandra Popa et al
Austria/CeMM0095/2020	EPI_ISL_437956	Universitaetsklinik für Innere Medizin II Innsbruck	Bergthaler laboratory, CeMM Research Center for Molecular Medicine of the Austrian Academy of Sciences	2020-03-23	Alexandra Popa et al
Austria/CeMM0096/2020	EPI_ISL_437957	Universitaetsklinik für Innere Medizin II Innsbruck	Bergthaler laboratory, CeMM Research Center for Molecular Medicine of the Austrian Academy of Sciences	2020-03-23	Alexandra Popa et al
Austria/CeMM0098/2020	EPI_ISL_437959	Universitaetsklinik für Innere Medizin II Innsbruck	Bergthaler laboratory, CeMM Research Center for Molecular Medicine of the Austrian Academy of Sciences	2020-03-20	Alexandra Popa et al
Austria/CeMM0099/2020	EPI_ISL_437960	Universitaetsklinik für Innere Medizin II Innsbruck	Bergthaler laboratory, CeMM Research Center for Molecular Medicine of the Austrian Academy of Sciences	2020-03-20	Alexandra Popa et al
Austria/CeMM0100/2020	EPI_ISL_437961	Universitaetsklinik für Innere Medizin II Innsbruck	Bergthaler laboratory, CeMM Research Center for Molecular Medicine of the Austrian Academy of Sciences	2020-03-20	Alexandra Popa et al
Austria/CeMM0101/2020	EPI_ISL_437962	Universitaetsklinik für Innere Medizin II Innsbruck	Bergthaler laboratory, CeMM Research Center for Molecular Medicine of the Austrian Academy of Sciences	2020-03-20	Alexandra Popa et al
Austria/CeMM0102/2020	EPI_ISL_437963	Universitaetsklinik für Innere Medizin II Innsbruck	Bergthaler laboratory, CeMM Research Center for Molecular Medicine of the Austrian Academy of Sciences	2020-03-19	Alexandra Popa et al
Austria/CeMM0103/2020	EPI_ISL_437964	Universitaetsklinik für Innere Medizin II Innsbruck	Bergthaler laboratory, CeMM Research Center for Molecular Medicine of the Austrian Academy of Sciences	2020-03-19	Alexandra Popa et al
Austria/CeMM0104/2020	EPI_ISL_437965	Universitaetsklinik für Innere Medizin II Innsbruck	Bergthaler laboratory, CeMM Research Center for Molecular Medicine of the Austrian Academy of Sciences	2020-03-19	Alexandra Popa et al
Austria/CeMM0106/2020	EPI_ISL_437966	Universitaetsklinik für Innere Medizin II Innsbruck	Bergthaler laboratory, CeMM Research Center for Molecular Medicine of the Austrian Academy of Sciences	2020-03-18	Alexandra Popa et al
Austria/CeMM0108/2020	EPI_ISL_437967	Universitaetsklinik für Innere Medizin II Innsbruck	Bergthaler laboratory, CeMM Research Center for Molecular Medicine of the Austrian Academy of Sciences	2020-03-13	Alexandra Popa et al
Austria/CeMM0109/2020	EPI_ISL_437968	Universitaetsklinik für Innere Medizin II Innsbruck	Bergthaler laboratory, CeMM Research Center for Molecular Medicine of the Austrian Academy of Sciences	2020-03-12	Alexandra Popa et al
Austria/CeMM0111/2020	EPI_ISL_437969	Universitaetsklinik für Innere Medizin II Innsbruck	Bergthaler laboratory, CeMM Research Center for Molecular Medicine of the Austrian Academy of Sciences	2020-03-12	Alexandra Popa et al
Austria/CeMM0112/2020	EPI_ISL_437970	Universitaetsklinik für Innere Medizin II Innsbruck	Bergthaler laboratory, CeMM Research Center for Molecular Medicine of the Austrian Academy of Sciences	2020-03-17	Alexandra Popa et al
Austria/CeMM0113/2020	EPI_ISL_437971	Universitaetsklinik für Innere Medizin II Innsbruck	Bergthaler laboratory, CeMM Research Center for Molecular Medicine of the Austrian Academy of Sciences	2020-03-18	Alexandra Popa et al
Austria/CeMM0115/2020	EPI_ISL_437973	Universitaetsklinik für Innere Medizin II Innsbruck	Bergthaler laboratory, CeMM Research Center for Molecular Medicine of the Austrian Academy of Sciences	2020-03-18	Alexandra Popa et al
Austria/CeMM0116/2020	EPI_ISL_437974	Institut für Virologie am Department für Hygiene, Mikrobiologie und Public Health	Bergthaler laboratory, CeMM Research Center for Molecular Medicine of the Austrian Academy of Sciences	2020-03-10	Alexandra Popa et al
Austria/CeMM0117/2020	EPI_ISL_437975	Institut für Virologie am Department für Hygiene, Mikrobiologie und Public Health	Bergthaler laboratory, CeMM Research Center for Molecular Medicine of the Austrian Academy of Sciences	2020-03-13	Alexandra Popa et al
Austria/CeMM0120/2020	EPI_ISL_437977	Institut für Virologie am Department für Hygiene, Mikrobiologie und Public Health	Bergthaler laboratory, CeMM Research Center for Molecular Medicine of the Austrian Academy of Sciences	2020-03-15	Alexandra Popa et al
Austria/CeMM0121/2020	EPI_ISL_437978	Institut für Virologie am Department für Hygiene, Mikrobiologie und Public Health	Bergthaler laboratory, CeMM Research Center for Molecular Medicine of the Austrian Academy of Sciences	2020-03-11	Alexandra Popa et al
Austria/CeMM0123/2020	EPI_ISL_437979	Institut für Virologie am Department für Hygiene, Mikrobiologie und Public Health	Bergthaler laboratory, CeMM Research Center for Molecular Medicine of the Austrian Academy of Sciences	2020-03-11	Alexandra Popa et al
Austria/CeMM0124/2020	EPI_ISL_437980	Institut für Virologie am Department für Hygiene, Mikrobiologie und Public Health	Bergthaler laboratory, CeMM Research Center for Molecular Medicine of the Austrian Academy of Sciences	2020-03-11	Alexandra Popa et al
Austria/CeMM0125/2020	EPI_ISL_437981	Institut für Virologie am Department für Hygiene, Mikrobiologie und Public Health	Bergthaler laboratory, CeMM Research Center for Molecular Medicine of the Austrian Academy of Sciences	2020-03-11	Alexandra Popa et al
Austria/CeMM0126/2020	EPI_ISL_437982	Institut für Virologie am Department für Hygiene, Mikrobiologie und Public Health	Bergthaler laboratory, CeMM Research Center for Molecular Medicine of the Austrian Academy of Sciences	2020-03-11	Alexandra Popa et al
Austria/CeMM0127/2020	EPI_ISL_437983	Institut für Virologie am Department für Hygiene, Mikrobiologie und Public Health	Bergthaler laboratory, CeMM Research Center for Molecular Medicine of the Austrian Academy of Sciences	2020-03-11	Alexandra Popa et al
Austria/CeMM0128/2020	EPI_ISL_437984	Institut für Virologie am Department für Hygiene, Mikrobiologie und Public Health	Bergthaler laboratory, CeMM Research Center for Molecular Medicine of the Austrian Academy of Sciences	2020-03-11	Alexandra Popa et al
Austria/CeMM0132/2020	EPI_ISL_437985	Institut für Virologie am Department für Hygiene, Mikrobiologie und Public Health	Bergthaler laboratory, CeMM Research Center for Molecular Medicine of the Austrian Academy of Sciences	2020-03-13	Alexandra Popa et al
Austria/CeMM0133/2020	EPI_ISL_437986	Institut für Virologie am Department für Hygiene, Mikrobiologie und Public Health	Bergthaler laboratory, CeMM Research Center for Molecular Medicine of the Austrian Academy of Sciences	2020-03-13	Alexandra Popa et al
Austria/CeMM0134/2020	EPI_ISL_437987	Institut für Virologie am Department für Hygiene, Mikrobiologie und Public Health	Bergthaler laboratory, CeMM Research Center for Molecular Medicine of the Austrian Academy of Sciences	2020-03-13	Alexandra Popa et al
Austria/CeMM0135/2020	EPI_ISL_437988	Institut für Virologie am Department für Hygiene, Mikrobiologie und Public Health	Bergthaler laboratory, CeMM Research Center for Molecular Medicine of the Austrian Academy of Sciences	2020-03-13	Alexandra Popa et al
Austria/CeMM0136/2020	EPI_ISL_437989	Institut für Virologie am Department für Hygiene, Mikrobiologie und Public Health	Bergthaler laboratory, CeMM Research Center for Molecular Medicine of the Austrian Academy of Sciences	2020-03-13	Alexandra Popa et al
Austria/CeMM0137/2020	EPI_ISL_437990	Institut für Virologie am Department für Hygiene, Mikrobiologie und Public Health	Bergthaler laboratory, CeMM Research Center for Molecular Medicine of the Austrian Academy of Sciences	2020-03-13	Alexandra Popa et al
Austria/CeMM0139/2020	EPI_ISL_437991	Institut für Virologie am Department für Hygiene, Mikrobiologie und Public Health	Bergthaler laboratory, CeMM Research Center for Molecular Medicine of the Austrian Academy of Sciences	2020-03-13	Alexandra Popa et al
Austria/CeMM0140/2020	EPI_ISL_437992	Institut für Virologie am Department für Hygiene, Mikrobiologie und Public Health	Bergthaler laboratory, CeMM Research Center for Molecular Medicine of the Austrian Academy of Sciences	2020-03-13	Alexandra Popa et al
Austria/CeMM0143/2020	EPI_ISL_437993	Center for Virology, Medical University of Vienna	Bergthaler laboratory, CeMM Research Center for Molecular Medicine of the Austrian Academy of Sciences	2020-02-27	Alexandra Popa et al
Austria/CeMM0146/2020	EPI_ISL_437994	Center for Virology, Medical University of Vienna	Bergthaler laboratory, CeMM Research Center for Molecular Medicine of the Austrian Academy of Sciences	2020-02-28	Alexandra Popa et al
Austria/CeMM0147/2020	EPI_ISL_437995	Center for Virology, Medical University of Vienna	Bergthaler laboratory, CeMM Research Center for Molecular Medicine of the Austrian Academy of Sciences	2020-02-28	Alexandra Popa et al
Austria/CeMM0148/2020	EPI_ISL_437996	Center for Virology, Medical University of Vienna	Bergthaler laboratory, CeMM Research Center for Molecular Medicine of the Austrian Academy of Sciences	2020-03-02	Alexandra Popa et al
Austria/CeMM0149/2020	EPI_ISL_437997	Center for Virology, Medical University of Vienna	Bergthaler laboratory, CeMM Research Center for Molecular Medicine of the Austrian Academy of Sciences	2020-03-03	Alexandra Popa et al
Austria/CeMM0150/2020	EPI_ISL_437998	Center for Virology, Medical University of Vienna	Bergthaler laboratory, CeMM Research Center for Molecular Medicine of the Austrian Academy of Sciences	2020-03-03	Alexandra Popa et al
Austria/CeMM0153/2020	EPI_ISL_437999	Center for Virology, Medical University of Vienna	Bergthaler laboratory, CeMM Research Center for Molecular Medicine of the Austrian Academy of Sciences	2020-03-03	Alexandra Popa et al
Austria/CeMM0154/2020	EPI_ISL_438000	Center for Virology, Medical University of Vienna	Bergthaler laboratory, CeMM Research Center for Molecular Medicine of the Austrian Academy of Sciences	2020-03-03	Alexandra Popa et al
Austria/CeMM0155/2020	EPI_ISL_438001	Center for Virology, Medical University of Vienna	Bergthaler laboratory, CeMM Research Center for Molecular Medicine of the Austrian Academy of Sciences	2020-03-03	Alexandra Popa et al
Austria/CeMM0156/2020	EPI_ISL_438002	Center for Virology, Medical University of Vienna	Bergthaler laboratory, CeMM Research Center for Molecular Medicine of the Austrian Academy of Sciences	2020-03-03	Alexandra Popa et al
Austria/CeMM0157/2020	EPI_ISL_438003	Center for Virology, Medical University of Vienna	Bergthaler laboratory, CeMM Research Center for Molecular Medicine of the Austrian Academy of Sciences	2020-03-04	Alexandra Popa et al
Austria/CeMM0158/2020	EPI_ISL_438004	Center for Virology, Medical University of Vienna	Bergthaler laboratory, CeMM Research Center for Molecular Medicine of the Austrian Academy of Sciences	2020-03-04	Alexandra Popa et al
Austria/CeMM0159/2020	EPI_ISL_438005	Center for Virology, Medical University of Vienna	Bergthaler laboratory, CeMM Research Center for Molecular Medicine of the Austrian Academy of Sciences	2020-03-04	Alexandra Popa et al
Austria/CeMM0160/2020	EPI_ISL_438006	Center for Virology, Medical University of Vienna	Bergthaler laboratory, CeMM Research Center for Molecular Medicine of the Austrian Academy of Sciences	2020-03-04	Alexandra Popa et al
Austria/CeMM0161/2020	EPI_ISL_438007	Center for Virology, Medical University of Vienna	Bergthaler laboratory, CeMM Research Center for Molecular Medicine of the Austrian Academy of Sciences	2020-03-05	Alexandra Popa et al
Austria/CeMM0162/2020	EPI_ISL_438008	Center for Virology, Medical University of Vienna	Bergthaler laboratory, CeMM Research Center for Molecular Medicine of the Austrian Academy of Sciences	2020-03-05	Alexandra Popa et al
Austria/CeMM0163/2020	EPI_ISL_438009	Center for Virology, Medical University of Vienna	Bergthaler laboratory, CeMM Research Center for Molecular Medicine of the Austrian Academy of Sciences	2020-03-06	Alexandra Popa et al
Austria/CeMM0165/2020	EPI_ISL_438010	Center for Virology, Medical University of Vienna	Bergthaler laboratory, CeMM Research Center for Molecular Medicine of the Austrian Academy of Sciences	2020-03-06	Alexandra Popa et al
Austria/CeMM0166/2020	EPI_ISL_438011	Center for Virology, Medical University of Vienna	Bergthaler laboratory, CeMM Research Center for Molecular Medicine of the Austrian Academy of Sciences	2020-03-06	Alexandra Popa et al
Austria/CeMM0167/2020	EPI_ISL_438012	Center for Virology, Medical University of Vienna	Bergthaler laboratory, CeMM Research Center for Molecular Medicine of the Austrian Academy of Sciences	2020-03-06	Alexandra Popa et al
Austria/CeMM0168/2020	EPI_ISL_438013	Center for Virology, Medical University of Vienna	Bergthaler laboratory, CeMM Research Center for Molecular Medicine of the Austrian Academy of Sciences	2020-03-07	Alexandra Popa et al
Austria/CeMM0169/2020	EPI_ISL_438014	Center for Virology, Medical University of Vienna	Bergthaler laboratory, CeMM Research Center for Molecular Medicine of the Austrian Academy of Sciences	2020-03-07	Alexandra Popa et al
Austria/CeMM0170/2020	EPI_ISL_438015	Center for Virology, Medical University of Vienna	Bergthaler laboratory, CeMM Research Center for Molecular Medicine of the Austrian Academy of Sciences	2020-03-07	Alexandra Popa et al
Austria/CeMM0171/2020	EPI_ISL_438016	Center for Virology, Medical University of Vienna	Bergthaler laboratory, CeMM Research Center for Molecular Medicine of the Austrian Academy of Sciences	2020-03-07	Alexandra Popa et al
Austria/CeMM0172/2020	EPI_ISL_438017	Center for Virology, Medical University of Vienna	Bergthaler laboratory, CeMM Research Center for Molecular Medicine of the Austrian Academy of Sciences	2020-03-07	Alexandra Popa et al
Austria/CeMM0175/2020	EPI_ISL_438018	Center for Virology, Medical University of Vienna	Bergthaler laboratory, CeMM Research Center for Molecular Medicine of the Austrian Academy of Sciences	2020-03-08	Alexandra Popa et al
Austria/CeMM0176/2020	EPI_ISL_438019	Center for Virology, Medical University of Vienna	Bergthaler laboratory, CeMM Research Center for Molecular Medicine of the Austrian Academy of Sciences	2020-03-08	Alexandra Popa et al
Austria/CeMM0177/2020	EPI_ISL_438020	Center for Virology, Medical University of Vienna	Bergthaler laboratory, CeMM Research Center for Molecular Medicine of the Austrian Academy of Sciences	2020-03-08	Alexandra Popa et al
Austria/CeMM0178/2020	EPI_ISL_438021	Center for Virology, Medical University of Vienna	Bergthaler laboratory, CeMM Research Center for Molecular Medicine of the Austrian Academy of Sciences	2020-03-08	Alexandra Popa et al
Austria/CeMM0180/2020	EPI_ISL_438022	Center for Virology, Medical University of Vienna	Bergthaler laboratory, CeMM Research Center for Molecular Medicine of the Austrian Academy of Sciences	2020-03-09	Alexandra Popa et al
Austria/CeMM0181/2020	EPI_ISL_438023	Center for Virology, Medical University of Vienna	Bergthaler laboratory, CeMM Research Center for Molecular Medicine of the Austrian Academy of Sciences	2020-03-09	Alexandra Popa et al
Austria/CeMM0182/2020	EPI_ISL_438024	Center for Virology, Medical University of Vienna	Bergthaler laboratory, CeMM Research Center for Molecular Medicine of the Austrian Academy of Sciences	2020-03-09	Alexandra Popa et al
Austria/CeMM0183/2020	EPI_ISL_438025	Center for Virology, Medical University of Vienna	Bergthaler laboratory, CeMM Research Center for Molecular Medicine of the Austrian Academy of Sciences	2020-03-10	Alexandra Popa et al
Austria/CeMM0184/2020	EPI_ISL_438026	Center for Virology, Medical University of Vienna	Bergthaler laboratory, CeMM Research Center for Molecular Medicine of the Austrian Academy of Sciences	2020-03-11	Alexandra Popa et al
Austria/CeMM0185/2020	EPI_ISL_438027	Center for Virology, Medical University of Vienna	Bergthaler laboratory, CeMM Research Center for Molecular Medicine of the Austrian Academy of Sciences	2020-03-11	Alexandra Popa et al
Austria/CeMM0186/2020	EPI_ISL_438028	Center for Virology, Medical University of Vienna	Bergthaler laboratory, CeMM Research Center for Molecular Medicine of the Austrian Academy of Sciences	2020-03-11	Alexandra Popa et al
Austria/CeMM0188/2020	EPI_ISL_438029	Center for Virology, Medical University of Vienna	Bergthaler laboratory, CeMM Research Center for Molecular Medicine of the Austrian Academy of Sciences	2020-03-13	Alexandra Popa et al
Austria/CeMM0189/2020	EPI_ISL_438030	Center for Virology, Medical University of Vienna	Bergthaler laboratory, CeMM Research Center for Molecular Medicine of the Austrian Academy of Sciences	2020-03-13	Alexandra Popa et al
Austria/CeMM0190/2020	EPI_ISL_438031	Center for Virology, Medical University of Vienna	Bergthaler laboratory, CeMM Research Center for Molecular Medicine of the Austrian Academy of Sciences	2020-03-13	Alexandra Popa et al
Austria/CeMM0193/2020	EPI_ISL_438034	Center for Virology, Medical University of Vienna	Bergthaler laboratory, CeMM Research Center for Molecular Medicine of the Austrian Academy of Sciences	2020-03-14	Alexandra Popa et al
Austria/CeMM0194/2020	EPI_ISL_438035	Center for Virology, Medical University of Vienna	Bergthaler laboratory, CeMM Research Center for Molecular Medicine of the Austrian Academy of Sciences	2020-03-14	Alexandra Popa et al
Austria/CeMM0195/2020	EPI_ISL_438036	Center for Virology, Medical University of Vienna	Bergthaler laboratory, CeMM Research Center for Molecular Medicine of the Austrian Academy of Sciences	2020-03-14	Alexandra Popa et al
Austria/CeMM0196/2020	EPI_ISL_438037	Center for Virology, Medical University of Vienna	Bergthaler laboratory, CeMM Research Center for Molecular Medicine of the Austrian Academy of Sciences	2020-03-14	Alexandra Popa et al
Austria/CeMM0197/2020	EPI_ISL_438038	Center for Virology, Medical University of Vienna	Bergthaler laboratory, CeMM Research Center for Molecular Medicine of the Austrian Academy of Sciences	2020-03-14	Alexandra Popa et al
Austria/CeMM0198/2020	EPI_ISL_438039	Center for Virology, Medical University of Vienna	Bergthaler laboratory, CeMM Research Center for Molecular Medicine of the Austrian Academy of Sciences	2020-03-14	Alexandra Popa et al
Austria/CeMM0199/2020	EPI_ISL_438040	Center for Virology, Medical University of Vienna	Bergthaler laboratory, CeMM Research Center for Molecular Medicine of the Austrian Academy of Sciences	2020-03-15	Alexandra Popa et al
Austria/CeMM0200/2020	EPI_ISL_438041	Center for Virology, Medical University of Vienna	Bergthaler laboratory, CeMM Research Center for Molecular Medicine of the Austrian Academy of Sciences	2020-03-15	Alexandra Popa et al
Austria/CeMM0201/2020	EPI_ISL_438042	Center for Virology, Medical University of Vienna	Bergthaler laboratory, CeMM Research Center for Molecular Medicine of the Austrian Academy of Sciences	2020-03-15	Alexandra Popa et al
Austria/CeMM0204/2020	EPI_ISL_438043	Center for Virology, Medical University of Vienna	Bergthaler laboratory, CeMM Research Center for Molecular Medicine of the Austrian Academy of Sciences	2020-03-15	Alexandra Popa et al
Austria/CeMM0205/2020	EPI_ISL_438044	Center for Virology, Medical University of Vienna	Bergthaler laboratory, CeMM Research Center for Molecular Medicine of the Austrian Academy of Sciences	2020-03-15	Alexandra Popa et al
Austria/CeMM0206/2020	EPI_ISL_438045	Center for Virology, Medical University of Vienna	Bergthaler laboratory, CeMM Research Center for Molecular Medicine of the Austrian Academy of Sciences	2020-03-15	Alexandra Popa et al
Austria/CeMM0209/2020	EPI_ISL_438046	Center for Virology, Medical University of Vienna	Bergthaler laboratory, CeMM Research Center for Molecular Medicine of the Austrian Academy of Sciences	2020-03-16	Alexandra Popa et al
Austria/CeMM0210/2020	EPI_ISL_438047	Center for Virology, Medical University of Vienna	Bergthaler laboratory, CeMM Research Center for Molecular Medicine of the Austrian Academy of Sciences	2020-03-16	Alexandra Popa et al
Austria/CeMM0212/2020	EPI_ISL_438048	Center for Virology, Medical University of Vienna	Bergthaler laboratory, CeMM Research Center for Molecular Medicine of the Austrian Academy of Sciences	2020-03-16	Alexandra Popa et al
Austria/CeMM0213/2020	EPI_ISL_438049	Center for Virology, Medical University of Vienna	Bergthaler laboratory, CeMM Research Center for Molecular Medicine of the Austrian Academy of Sciences	2020-03-16	Alexandra Popa et al

Austria/CeMM0216/2020	EPI_ISL_438050	Center for Virology, Medical University of Vienna	Bergthaler laboratory, CeMM Research Center for Molecular Medicine of the Austrian Academy of Sciences	2020-03-16	Alexandra Popa et al
Austria/CeMM0217/2020	EPI_ISL_438051	Center for Virology, Medical University of Vienna	Bergthaler laboratory, CeMM Research Center for Molecular Medicine of the Austrian Academy of Sciences	2020-03-16	Alexandra Popa et al
Austria/CeMM0218/2020	EPI_ISL_438052	Center for Virology, Medical University of Vienna	Bergthaler laboratory, CeMM Research Center for Molecular Medicine of the Austrian Academy of Sciences	2020-03-16	Alexandra Popa et al
Austria/CeMM0219/2020	EPI_ISL_438053	Center for Virology, Medical University of Vienna	Bergthaler laboratory, CeMM Research Center for Molecular Medicine of the Austrian Academy of Sciences	2020-03-16	Alexandra Popa et al
Austria/CeMM0220/2020	EPI_ISL_438054	Center for Virology, Medical University of Vienna	Bergthaler laboratory, CeMM Research Center for Molecular Medicine of the Austrian Academy of Sciences	2020-03-16	Alexandra Popa et al
Austria/CeMM0221/2020	EPI_ISL_438055	Center for Virology, Medical University of Vienna	Bergthaler laboratory, CeMM Research Center for Molecular Medicine of the Austrian Academy of Sciences	2020-03-16	Alexandra Popa et al
Austria/CeMM0222/2020	EPI_ISL_438056	Center for Virology, Medical University of Vienna	Bergthaler laboratory, CeMM Research Center for Molecular Medicine of the Austrian Academy of Sciences	2020-03-17	Alexandra Popa et al
Austria/CeMM0223/2020	EPI_ISL_438057	Center for Virology, Medical University of Vienna	Bergthaler laboratory, CeMM Research Center for Molecular Medicine of the Austrian Academy of Sciences	2020-03-17	Alexandra Popa et al
Austria/CeMM0224/2020	EPI_ISL_438058	Center for Virology, Medical University of Vienna	Bergthaler laboratory, CeMM Research Center for Molecular Medicine of the Austrian Academy of Sciences	2020-03-17	Alexandra Popa et al
Austria/CeMM0225/2020	EPI_ISL_438059	Center for Virology, Medical University of Vienna	Bergthaler laboratory, CeMM Research Center for Molecular Medicine of the Austrian Academy of Sciences	2020-03-17	Alexandra Popa et al
Austria/CeMM0226/2020	EPI_ISL_438060	Center for Virology, Medical University of Vienna	Bergthaler laboratory, CeMM Research Center for Molecular Medicine of the Austrian Academy of Sciences	2020-03-17	Alexandra Popa et al
Austria/CeMM0228/2020	EPI_ISL_438061	Center for Virology, Medical University of Vienna	Bergthaler laboratory, CeMM Research Center for Molecular Medicine of the Austrian Academy of Sciences	2020-03-18	Alexandra Popa et al
Austria/CeMM0229/2020	EPI_ISL_438062	Center for Virology, Medical University of Vienna	Bergthaler laboratory, CeMM Research Center for Molecular Medicine of the Austrian Academy of Sciences	2020-03-18	Alexandra Popa et al
Austria/CeMM0230/2020	EPI_ISL_438063	Center for Virology, Medical University of Vienna	Bergthaler laboratory, CeMM Research Center for Molecular Medicine of the Austrian Academy of Sciences	2020-03-18	Alexandra Popa et al
Austria/CeMM0232/2020	EPI_ISL_438064	Center for Virology, Medical University of Vienna	Bergthaler laboratory, CeMM Research Center for Molecular Medicine of the Austrian Academy of Sciences	2020-03-18	Alexandra Popa et al
Austria/CeMM0233/2020	EPI_ISL_438065	Center for Virology, Medical University of Vienna	Bergthaler laboratory, CeMM Research Center for Molecular Medicine of the Austrian Academy of Sciences	2020-03-18	Alexandra Popa et al
Austria/CeMM0235/2020	EPI_ISL_438066	Center for Virology, Medical University of Vienna	Bergthaler laboratory, CeMM Research Center for Molecular Medicine of the Austrian Academy of Sciences	2020-03-19	Alexandra Popa et al
Austria/CeMM0237/2020	EPI_ISL_438067	Center for Virology, Medical University of Vienna	Bergthaler laboratory, CeMM Research Center for Molecular Medicine of the Austrian Academy of Sciences	2020-03-19	Alexandra Popa et al
Austria/CeMM0238/2020	EPI_ISL_438068	Center for Virology, Medical University of Vienna	Bergthaler laboratory, CeMM Research Center for Molecular Medicine of the Austrian Academy of Sciences	2020-03-19	Alexandra Popa et al
Austria/CeMM0239/2020	EPI_ISL_438069	Center for Virology, Medical University of Vienna	Bergthaler laboratory, CeMM Research Center for Molecular Medicine of the Austrian Academy of Sciences	2020-03-19	Alexandra Popa et al
Austria/CeMM0240/2020	EPI_ISL_438070	Center for Virology, Medical University of Vienna	Bergthaler laboratory, CeMM Research Center for Molecular Medicine of the Austrian Academy of Sciences	2020-03-20	Alexandra Popa et al
Austria/CeMM0241/2020	EPI_ISL_438071	Center for Virology, Medical University of Vienna	Bergthaler laboratory, CeMM Research Center for Molecular Medicine of the Austrian Academy of Sciences	2020-03-20	Alexandra Popa et al
Austria/CeMM0242/2020	EPI_ISL_438072	Center for Virology, Medical University of Vienna	Bergthaler laboratory, CeMM Research Center for Molecular Medicine of the Austrian Academy of Sciences	2020-03-20	Alexandra Popa et al
Austria/CeMM0243/2020	EPI_ISL_438073	Center for Virology, Medical University of Vienna	Bergthaler laboratory, CeMM Research Center for Molecular Medicine of the Austrian Academy of Sciences	2020-03-20	Alexandra Popa et al
Austria/CeMM0244/2020	EPI_ISL_438074	Center for Virology, Medical University of Vienna	Bergthaler laboratory, CeMM Research Center for Molecular Medicine of the Austrian Academy of Sciences	2020-03-20	Alexandra Popa et al
Austria/CeMM0245/2020	EPI_ISL_438075	Center for Virology, Medical University of Vienna	Bergthaler laboratory, CeMM Research Center for Molecular Medicine of the Austrian Academy of Sciences	2020-03-20	Alexandra Popa et al
Austria/CeMM0246/2020	EPI_ISL_438076	Center for Virology, Medical University of Vienna	Bergthaler laboratory, CeMM Research Center for Molecular Medicine of the Austrian Academy of Sciences	2020-03-21	Alexandra Popa et al
Austria/CeMM0247/2020	EPI_ISL_438077	Center for Virology, Medical University of Vienna	Bergthaler laboratory, CeMM Research Center for Molecular Medicine of the Austrian Academy of Sciences	2020-03-21	Alexandra Popa et al
Austria/CeMM0249/2020	EPI_ISL_438079	Center for Virology, Medical University of Vienna	Bergthaler laboratory, CeMM Research Center for Molecular Medicine of the Austrian Academy of Sciences	2020-03-21	Alexandra Popa et al
Austria/CeMM0250/2020	EPI_ISL_438080	Center for Virology, Medical University of Vienna	Bergthaler laboratory, CeMM Research Center for Molecular Medicine of the Austrian Academy of Sciences	2020-03-21	Alexandra Popa et al
Austria/CeMM0251/2020	EPI_ISL_438081	Center for Virology, Medical University of Vienna	Bergthaler laboratory, CeMM Research Center for Molecular Medicine of the Austrian Academy of Sciences	2020-03-21	Alexandra Popa et al
Austria/CeMM0252/2020	EPI_ISL_438082	Center for Virology, Medical University of Vienna	Bergthaler laboratory, CeMM Research Center for Molecular Medicine of the Austrian Academy of Sciences	2020-03-21	Alexandra Popa et al
Austria/CeMM0254/2020	EPI_ISL_438083	Center for Virology, Medical University of Vienna	Bergthaler laboratory, CeMM Research Center for Molecular Medicine of the Austrian Academy of Sciences	2020-03-22	Alexandra Popa et al
Austria/CeMM0255/2020	EPI_ISL_438084	Center for Virology, Medical University of Vienna	Bergthaler laboratory, CeMM Research Center for Molecular Medicine of the Austrian Academy of Sciences	2020-03-22	Alexandra Popa et al
Austria/CeMM0256/2020	EPI_ISL_438085	Center for Virology, Medical University of Vienna	Bergthaler laboratory, CeMM Research Center for Molecular Medicine of the Austrian Academy of Sciences	2020-03-22	Alexandra Popa et al
Austria/CeMM0257/2020	EPI_ISL_438086	Center for Virology, Medical University of Vienna	Bergthaler laboratory, CeMM Research Center for Molecular Medicine of the Austrian Academy of Sciences	2020-03-22	Alexandra Popa et al
Austria/CeMM0258/2020	EPI_ISL_438087	Center for Virology, Medical University of Vienna	Bergthaler laboratory, CeMM Research Center for Molecular Medicine of the Austrian Academy of Sciences	2020-03-22	Alexandra Popa et al
Austria/CeMM0259/2020	EPI_ISL_438088	Center for Virology, Medical University of Vienna	Bergthaler laboratory, CeMM Research Center for Molecular Medicine of the Austrian Academy of Sciences	2020-03-23	Alexandra Popa et al
Austria/CeMM0260/2020	EPI_ISL_438089	Center for Virology, Medical University of Vienna	Bergthaler laboratory, CeMM Research Center for Molecular Medicine of the Austrian Academy of Sciences	2020-03-23	Alexandra Popa et al
Austria/CeMM0261/2020	EPI_ISL_438090	Center for Virology, Medical University of Vienna	Bergthaler laboratory, CeMM Research Center for Molecular Medicine of the Austrian Academy of Sciences	2020-03-23	Alexandra Popa et al
Austria/CeMM0262/2020	EPI_ISL_438091	Center for Virology, Medical University of Vienna	Bergthaler laboratory, CeMM Research Center for Molecular Medicine of the Austrian Academy of Sciences	2020-03-23	Alexandra Popa et al
Austria/CeMM0263/2020	EPI_ISL_438092	Center for Virology, Medical University of Vienna	Bergthaler laboratory, CeMM Research Center for Molecular Medicine of the Austrian Academy of Sciences	2020-03-23	Alexandra Popa et al
Austria/CeMM0264/2020	EPI_ISL_438093	Center for Virology, Medical University of Vienna	Bergthaler laboratory, CeMM Research Center for Molecular Medicine of the Austrian Academy of Sciences	2020-03-23	Alexandra Popa et al
Austria/CeMM0265/2020	EPI_ISL_438094	Center for Virology, Medical University of Vienna	Bergthaler laboratory, CeMM Research Center for Molecular Medicine of the Austrian Academy of Sciences	2020-03-23	Alexandra Popa et al
Austria/CeMM0267/2020	EPI_ISL_438096	Center for Virology, Medical University of Vienna	Bergthaler laboratory, CeMM Research Center for Molecular Medicine of the Austrian Academy of Sciences	2020-03-24	Alexandra Popa et al
Austria/CeMM0269/2020	EPI_ISL_438097	Center for Virology, Medical University of Vienna	Bergthaler laboratory, CeMM Research Center for Molecular Medicine of the Austrian Academy of Sciences	2020-03-24	Alexandra Popa et al
Austria/CeMM0271/2020	EPI_ISL_438098	Center for Virology, Medical University of Vienna	Bergthaler laboratory, CeMM Research Center for Molecular Medicine of the Austrian Academy of Sciences	2020-03-24	Alexandra Popa et al
Austria/CeMM0272/2020	EPI_ISL_438099	Center for Virology, Medical University of Vienna	Bergthaler laboratory, CeMM Research Center for Molecular Medicine of the Austrian Academy of Sciences	2020-03-24	Alexandra Popa et al
Austria/CeMM0273/2020	EPI_ISL_438100	Center for Virology, Medical University of Vienna	Bergthaler laboratory, CeMM Research Center for Molecular Medicine of the Austrian Academy of Sciences	2020-03-24	Alexandra Popa et al
Austria/CeMM0274/2020	EPI_ISL_438101	Center for Virology, Medical University of Vienna	Bergthaler laboratory, CeMM Research Center for Molecular Medicine of the Austrian Academy of Sciences	2020-03-24	Alexandra Popa et al
Austria/CeMM0275/2020	EPI_ISL_438102	Center for Virology, Medical University of Vienna	Bergthaler laboratory, CeMM Research Center for Molecular Medicine of the Austrian Academy of Sciences	2020-03-24	Alexandra Popa et al
Austria/CeMM0276/2020	EPI_ISL_438103	Center for Virology, Medical University of Vienna	Bergthaler laboratory, CeMM Research Center for Molecular Medicine of the Austrian Academy of Sciences	2020-03-25	Alexandra Popa et al
Austria/CeMM0277/2020	EPI_ISL_438104	Center for Virology, Medical University of Vienna	Bergthaler laboratory, CeMM Research Center for Molecular Medicine of the Austrian Academy of Sciences	2020-03-25	Alexandra Popa et al
Austria/CeMM0278/2020	EPI_ISL_438105	Center for Virology, Medical University of Vienna	Bergthaler laboratory, CeMM Research Center for Molecular Medicine of the Austrian Academy of Sciences	2020-03-25	Alexandra Popa et al
Austria/CeMM0279/2020	EPI_ISL_438106	Center for Virology, Medical University of Vienna	Bergthaler laboratory, CeMM Research Center for Molecular Medicine of the Austrian Academy of Sciences	2020-03-25	Alexandra Popa et al
Austria/CeMM0280/2020	EPI_ISL_438107	Center for Virology, Medical University of Vienna	Bergthaler laboratory, CeMM Research Center for Molecular Medicine of the Austrian Academy of Sciences	2020-03-25	Alexandra Popa et al
Austria/CeMM0281/2020	EPI_ISL_438108	Center for Virology, Medical University of Vienna	Bergthaler laboratory, CeMM Research Center for Molecular Medicine of the Austrian Academy of Sciences	2020-03-25	Alexandra Popa et al
Austria/CeMM0283/2020	EPI_ISL_438109	Center for Virology, Medical University of Vienna	Bergthaler laboratory, CeMM Research Center for Molecular Medicine of the Austrian Academy of Sciences	2020-03-25	Alexandra Popa et al
Austria/CeMM0284/2020	EPI_ISL_438110	Center for Virology, Medical University of Vienna	Bergthaler laboratory, CeMM Research Center for Molecular Medicine of the Austrian Academy of Sciences	2020-03-26	Alexandra Popa et al
Austria/CeMM0285/2020	EPI_ISL_438111	Center for Virology, Medical University of Vienna	Bergthaler laboratory, CeMM Research Center for Molecular Medicine of the Austrian Academy of Sciences	2020-03-26	Alexandra Popa et al
Austria/CeMM0287/2020	EPI_ISL_438112	Center for Virology, Medical University of Vienna	Bergthaler laboratory, CeMM Research Center for Molecular Medicine of the Austrian Academy of Sciences	2020-03-26	Alexandra Popa et al
Austria/CeMM0288/2020	EPI_ISL_438113	Center for Virology, Medical University of Vienna	Bergthaler laboratory, CeMM Research Center for Molecular Medicine of the Austrian Academy of Sciences	2020-03-26	Alexandra Popa et al
Austria/CeMM0290/2020	EPI_ISL_438114	Center for Virology, Medical University of Vienna	Bergthaler laboratory, CeMM Research Center for Molecular Medicine of the Austrian Academy of Sciences	2020-03-27	Alexandra Popa et al
Austria/CeMM0291/2020	EPI_ISL_438115	Center for Virology, Medical University of Vienna	Bergthaler laboratory, CeMM Research Center for Molecular Medicine of the Austrian Academy of Sciences	2020-03-27	Alexandra Popa et al
Austria/CeMM0292/2020	EPI_ISL_438116	Center for Virology, Medical University of Vienna	Bergthaler laboratory, CeMM Research Center for Molecular Medicine of the Austrian Academy of Sciences	2020-03-27	Alexandra Popa et al
Austria/CeMM0294/2020	EPI_ISL_438117	Center for Virology, Medical University of Vienna	Bergthaler laboratory, CeMM Research Center for Molecular Medicine of the Austrian Academy of Sciences	2020-03-27	Alexandra Popa et al
Austria/CeMM0295/2020	EPI_ISL_438118	Center for Virology, Medical University of Vienna	Bergthaler laboratory, CeMM Research Center for Molecular Medicine of the Austrian Academy of Sciences	2020-03-27	Alexandra Popa et al
Austria/CeMM0296/2020	EPI_ISL_438119	Center for Virology, Medical University of Vienna	Bergthaler laboratory, CeMM Research Center for Molecular Medicine of the Austrian Academy of Sciences	2020-03-27	Alexandra Popa et al
Austria/CeMM0357/2020	EPI_ISL_438120	Center for Virology, Medical University of Vienna	Bergthaler laboratory, CeMM Research Center for Molecular Medicine of the Austrian Academy of Sciences	2020-03-19	Alexandra Popa et al
Austria/CeMM0358/2020	EPI_ISL_438121	Center for Virology, Medical University of Vienna	Bergthaler laboratory, CeMM Research Center for Molecular Medicine of the Austrian Academy of Sciences	2020-03-31	Alexandra Popa et al
Austria/CeMM0359/2020	EPI_ISL_438122	Center for Virology, Medical University of Vienna	Bergthaler laboratory, CeMM Research Center for Molecular Medicine of the Austrian Academy of Sciences	2020-04-02	Alexandra Popa et al
Austria/CeMM0360/2020	EPI_ISL_438123	Center for Virology, Medical University of Vienna	Bergthaler laboratory, CeMM Research Center for Molecular Medicine of the Austrian Academy of Sciences	2020-04-05	Alexandra Popa et al
Austria/CeMM0361/2020	EPI_ISL_438124	Center for Virology, Medical University of Vienna	Bergthaler laboratory, CeMM Research Center for Molecular Medicine of the Austrian Academy of Sciences	2020-03-22	Alexandra Popa et al
Austria/CeMM0362/2020	EPI_ISL_438125	Center for Virology, Medical University of Vienna	Bergthaler laboratory, CeMM Research Center for Molecular Medicine of the Austrian Academy of Sciences	2020-03-30	Alexandra Popa et al
Austria/CeMM0363/2020	EPI_ISL_438126	Center for Virology, Medical University of Vienna	Bergthaler laboratory, CeMM Research Center for Molecular Medicine of the Austrian Academy of Sciences	2020-04-02	Alexandra Popa et al
Austria/CeMM0364/2020	EPI_ISL_438127	Center for Virology, Medical University of Vienna	Bergthaler laboratory, CeMM Research Center for Molecular Medicine of the Austrian Academy of Sciences	2020-04-05	Alexandra Popa et al
Austria/CeMM0365/2020	EPI_ISL_438128	Center for Virology, Medical University of Vienna	Bergthaler laboratory, CeMM Research Center for Molecular Medicine of the Austrian Academy of Sciences	2020-04-07	Alexandra Popa et al
Austria/Graz-MUG16/2020	EPI_ISL_437302	Diagnostic- and Research Institute of Pathology, Medical University of Graz	Diagnostic- and Research Institute of Pathology, Medical University of Graz	2020-04-17	Karl Kashofer et al
Austria/Graz-MUG17/2020	EPI_ISL_437303	Diagnostic- and Research Institute of Pathology, Medical University of Graz	Diagnostic- and Research Institute of Pathology, Medical University of Graz	2020-04-17	Karl Kashofer et al
Austria/Graz-MUG3/2020	EPI_ISL_437199	Diagnostic- and Research Institute of Pathology, Medical University of Graz	Diagnostic- and Research Institute of Pathology, Medical University of Graz	2020-03-31	Karl Kashofer et al
Austria/Graz-MUG4/2020	EPI_ISL_437200	Diagnostic- and Research Institute of Pathology, Medical University of Graz	Diagnostic- and Research Institute of Pathology, Medical University of Graz	2020-03-31	Karl Kashofer et al
Austria/Graz-MUG6/2020	EPI_ISL_437202	Diagnostic- and Research Institute of Pathology, Medical University of Graz	Diagnostic- and Research Institute of Pathology, Medical University of Graz	2020-04-03	Karl Kashofer et al
Austria/Graz-MUG8/2020	EPI_ISL_437298	Diagnostic- and Research Institute of Pathology, Medical University of Graz	Diagnostic- and Research Institute of Pathology, Medical University of Graz	2020-04-08	Karl Kashofer et al
Austria/Graz-MUG9/2020	EPI_ISL_437299	Diagnostic- and Research Institute of Pathology, Medical University of Graz	Diagnostic- and Research Institute of Pathology, Medical University of Graz	2020-04-09	Karl Kashofer et al
cat/France/53/2020	EPI_ISL_437349	Ecole nationale vétérinaire d'Alfort-laboratoire de santé animale Anses UMR 1161 de virologie ENVA-Anses-INRAE	Institut Pasteur CIBU-ERI	2020-04-17	Sophie Le Poder et al
France/211_80/2020	EPI_ISL_469282	Service de Virologie Hôpital Saint-Louis	Laboratory of Cell Biology of viral infection, Unit INSERM-U944	2020-03-11	Laurent Meertens et al
France/220_95/2020	EPI_ISL_469284	Service de Virologie Hôpital Saint-Louis	Laboratory Cell Biology of Viral Infection-INSERM unit 944	2020-03-18	Laurent Meertens et al
France/221_61/2020	EPI_ISL_469283	Service de Virologie Hôpital Saint-Louis	Laboratory Cell Biology of Viral Infection-INSERM unit 944	2020-03-18	Laurent Meertens et al
France/ARA094100/2020	EPI_ISL_418412	Centre Hospitalier des Vals d'Ardeche	CNR Virus des Infections Respiratoires - France SUD	2020-03-15	Antonin Bal et al
France/ARA09428/2020	EPI_ISL_418413	Centre Hospitalier de Macon	CNR Virus des Infections Respiratoires - France SUD	2020-03-15	Antonin Bal et al
France/ARA09434/2020	EPI_ISL_418414	Centre Hospitalier de Valence	CNR Virus des Infections Respiratoires - France SUD	2020-03-15	Antonin Bal et al
France/ARA09451/2020	EPI_ISL_418415	Centre Hospitalier de Valence	CNR Virus des Infections Respiratoires - France SUD	2020-03-15	Antonin Bal et al
France/ARA09588/2020	EPI_ISL_418416	GH Les Portes du Sud	CNR Virus des Infections Respiratoires - France SUD	2020-03-16	Antonin Bal et al
France/ARA09686/2020	EPI_ISL_418417	Centre Hospitalier de Valence	CNR Virus des Infections Respiratoires - France SUD	2020-03-16	Antonin Bal et al
France/ARA10163/2020	EPI_ISL_418418	Centre Hospitalier Saint Joseph Saint Luc	CNR Virus des Infections Respiratoires - France SUD	2020-03-16	Antonin Bal et al
France/ARA10165/2020	EPI_ISL_418419	Centre Hospitalier Saint Joseph Saint Luc	CNR Virus des Infections Respiratoires - France SUD	2020-03-16	Antonin Bal et al
France/ARA10170/2020	EPI_ISL_418420	Institut des Agents Infectieux (IAI), Hospices Civils de Lyon	CNR Virus des Infections Respiratoires - France SUD	2020-03-17	Antonin Bal et al
France/ARA10172/2020	EPI_ISL_418421	Institut des Agents Infectieux (IAI), Hospices Civils de Lyon	CNR Virus des Infections Respiratoires - France SUD	2020-03-17	Antonin Bal et al
France/ARA10184/2020	EPI_ISL_418422	Institut des Agents Infectieux (IAI), Hospices Civils de Lyon	CNR Virus des Infections Respiratoires - France SUD	2020-03-17	Antonin Bal et al
France/ARA10188/2020	EPI_ISL_418423	Institut des Agents Infectieux (IAI), Hospices Civils de Lyon	CNR Virus des Infections Respiratoires - France SUD	2020-03-17	Antonin Bal et al
France/ARA10189/2020	EPI_ISL_418424	Institut des Agents Infectieux (IAI), Hospices Civils de Lyon	CNR Virus des Infections Respiratoires - France SUD	2020-03-17	Antonin Bal et al
France/ARA10192/2020	EPI_ISL_418425	Institut des Agents Infectieux (IAI), Hospices Civils de Lyon	CNR Virus des Infections Respiratoires - France SUD	2020-03-17	Antonin Bal et al
France/ARA10251/2020	EPI_ISL_418426	Centre Hospitalier de Bourg en Bresse	CNR Virus des Infections Respiratoires - France SUD	2020-03-17	Antonin Bal et al

France/ARA10257/2020	EPI_ISL_418427	Hopital Privé de l'Est Lyonnais	CNR Virus des Infections Respiratoires - France SUD	2020-03-17	Antonin Bal et al
France/ARA10282/2020	EPI_ISL_418428	Centre Hospitalier Lucien Hussel	CNR Virus des Infections Respiratoires - France SUD	2020-03-17	Antonin Bal et al
France/ARA10552/2020	EPI_ISL_419168	Centre Hospitalier de Valence	CNR Virus des Infections Respiratoires - France SUD	2020-03-17	Antonin Bal et al
France/ARA10876/2020	EPI_ISL_418429	Institut des Agents Infectieux (IAI), Hospices Civils de Lyon	CNR Virus des Infections Respiratoires - France SUD	2020-03-18	Antonin Bal et al
France/ARA10910/2020	EPI_ISL_418430	Institut des Agents Infectieux (IAI), Hospices Civils de Lyon	CNR Virus des Infections Respiratoires - France SUD	2020-03-18	Antonin Bal et al
France/ARA10968/2020	EPI_ISL_418431	Institut des Agents Infectieux (IAI), Hospices Civils de Lyon	CNR Virus des Infections Respiratoires - France SUD	2020-03-18	Antonin Bal et al
France/ARA11036/2020	EPI_ISL_418432	Institut des Agents Infectieux (IAI), Hospices Civils de Lyon	CNR Virus des Infections Respiratoires - France SUD	2020-03-18	Antonin Bal et al
France/ARA11939/2020	EPI_ISL_419169	Institut des Agents Infectieux (IAI), Hospices Civils de Lyon	CNR Virus des Infections Respiratoires - France SUD	2020-03-21	Antonin Bal et al
France/ARA11943/2020	EPI_ISL_419170	Institut des Agents Infectieux (IAI), Hospices Civils de Lyon	CNR Virus des Infections Respiratoires - France SUD	2020-03-21	Antonin Bal et al
France/ARA11949/2020	EPI_ISL_419171	Institut des Agents Infectieux (IAI), Hospices Civils de Lyon	CNR Virus des Infections Respiratoires - France SUD	2020-03-21	Antonin Bal et al
France/ARA11950/2020	EPI_ISL_419172	Institut des Agents Infectieux (IAI), Hospices Civils de Lyon	CNR Virus des Infections Respiratoires - France SUD	2020-03-21	Antonin Bal et al
France/ARA11952/2020	EPI_ISL_419173	Institut des Agents Infectieux (IAI), Hospices Civils de Lyon	CNR Virus des Infections Respiratoires - France SUD	2020-03-21	Antonin Bal et al
France/ARA11980/2020	EPI_ISL_419174	Centre Hospitalier de Macon	CNR Virus des Infections Respiratoires - France SUD	2020-03-20	Antonin Bal et al
France/ARA11995/2020	EPI_ISL_419175	Centre Hospitalier de Macon	CNR Virus des Infections Respiratoires - France SUD	2020-03-21	Antonin Bal et al
France/ARA11997/2020	EPI_ISL_419176	Centre Hospitalier de Macon	CNR Virus des Infections Respiratoires - France SUD	2020-03-21	Antonin Bal et al
France/ARA12125/2020	EPI_ISL_419177	Institut des Agents Infectieux (IAI), Hospices Civils de Lyon	CNR Virus des Infections Respiratoires - France SUD	2020-03-22	Antonin Bal et al
France/ARA12217/2020	EPI_ISL_419178	Institut des Agents Infectieux (IAI), Hospices Civils de Lyon	CNR Virus des Infections Respiratoires - France SUD	2020-03-22	Antonin Bal et al
France/ARA12222/2020	EPI_ISL_419179	Institut des Agents Infectieux (IAI), Hospices Civils de Lyon	CNR Virus des Infections Respiratoires - France SUD	2020-03-22	Antonin Bal et al
France/ARA12238/2020	EPI_ISL_419180	Institut des Agents Infectieux (IAI), Hospices Civils de Lyon	CNR Virus des Infections Respiratoires - France SUD	2020-03-22	Antonin Bal et al
France/ARA12249/2020	EPI_ISL_419181	Institut des Agents Infectieux (IAI), Hospices Civils de Lyon	CNR Virus des Infections Respiratoires - France SUD	2020-03-22	Antonin Bal et al
France/ARA12250/2020	EPI_ISL_419182	Institut des Agents Infectieux (IAI), Hospices Civils de Lyon	CNR Virus des Infections Respiratoires - France SUD	2020-03-22	Antonin Bal et al
France/ARA12253/2020	EPI_ISL_419183	Centre Hospitalier de Bourg en Bresse	CNR Virus des Infections Respiratoires - France SUD	2020-03-22	Antonin Bal et al
France/ARA12260/2020	EPI_ISL_419184	Institut des Agents Infectieux (IAI), Hospices Civils de Lyon	CNR Virus des Infections Respiratoires - France SUD	2020-03-22	Antonin Bal et al
France/ARA12264/2020	EPI_ISL_419185	Centre Hospitalier de Bourg en Bresse	CNR Virus des Infections Respiratoires - France SUD	2020-03-22	Antonin Bal et al
France/ARA12265/2020	EPI_ISL_419186	Centre Hospitalier de Bourg en Bresse	CNR Virus des Infections Respiratoires - France SUD	2020-03-22	Antonin Bal et al
France/ARA12269/2020	EPI_ISL_419187	Centre Hospitalier de Macon	CNR Virus des Infections Respiratoires - France SUD	2020-03-22	Antonin Bal et al
France/ARA12270/2020	EPI_ISL_419188	Centre Hospitalier de Macon	CNR Virus des Infections Respiratoires - France SUD	2020-03-22	Antonin Bal et al
France/ARA12371/2020	EPI_ISL_420604	Institut des Agents Infectieux (IAI), Hospices Civils de Lyon	CNR Virus des Infections Respiratoires - France SUD	2020-03-23	Antonin Bal et al
France/ARA12384/2020	EPI_ISL_420605	Institut des Agents Infectieux (IAI), Hospices Civils de Lyon	CNR Virus des Infections Respiratoires - France SUD	2020-03-22	Antonin Bal et al
France/ARA12388/2020	EPI_ISL_420606	Institut des Agents Infectieux (IAI), Hospices Civils de Lyon	CNR Virus des Infections Respiratoires - France SUD	2020-03-22	Antonin Bal et al
France/ARA12485/2020	EPI_ISL_420607	Institut des Agents Infectieux (IAI), Hospices Civils de Lyon	CNR Virus des Infections Respiratoires - France SUD	2020-03-23	Antonin Bal et al
France/ARA12499/2020	EPI_ISL_420608	Institut des Agents Infectieux (IAI), Hospices Civils de Lyon	CNR Virus des Infections Respiratoires - France SUD	2020-03-23	Antonin Bal et al
France/ARA12524/2020	EPI_ISL_420609	Institut des Agents Infectieux (IAI), Hospices Civils de Lyon	CNR Virus des Infections Respiratoires - France SUD	2020-03-23	Antonin Bal et al
France/ARA12558/2020	EPI_ISL_420610	Institut des Agents Infectieux (IAI), Hospices Civils de Lyon	CNR Virus des Infections Respiratoires - France SUD	2020-03-23	Antonin Bal et al
France/ARA12576/2020	EPI_ISL_420611	Institut des Agents Infectieux (IAI), Hospices Civils de Lyon	CNR Virus des Infections Respiratoires - France SUD	2020-03-23	Antonin Bal et al
France/ARA12626/2020	EPI_ISL_420612	Centre Hospitalier de Macon	CNR Virus des Infections Respiratoires - France SUD	2020-03-23	Antonin Bal et al
France/ARA12630/2020	EPI_ISL_420613	Centre Hospitalier de Macon	CNR Virus des Infections Respiratoires - France SUD	2020-03-23	Antonin Bal et al
France/ARA12632/2020	EPI_ISL_420614	Centre Hospitalier de Macon	CNR Virus des Infections Respiratoires - France SUD	2020-03-23	Antonin Bal et al
France/ARA12759/2020	EPI_ISL_420615	Institut des Agents Infectieux (IAI), Hospices Civils de Lyon	CNR Virus des Infections Respiratoires - France SUD	2020-03-23	Antonin Bal et al
France/ARA1284/2020	EPI_ISL_420616	Institut des Agents Infectieux (IAI), Hospices Civils de Lyon	CNR Virus des Infections Respiratoires - France SUD	2020-03-23	Antonin Bal et al
France/ARA12877/2020	EPI_ISL_420617	Centre Hospitalier Saint Joseph Saint Luc	CNR Virus des Infections Respiratoires - France SUD	2020-03-23	Antonin Bal et al
France/ARA12915/2020	EPI_ISL_420618	Institut des Agents Infectieux (IAI), Hospices Civils de Lyon	CNR Virus des Infections Respiratoires - France SUD	2020-03-23	Antonin Bal et al
France/ARA12973/2020	EPI_ISL_420619	Institut des Agents Infectieux (IAI), Hospices Civils de Lyon	CNR Virus des Infections Respiratoires - France SUD	2020-03-23	Antonin Bal et al
France/ARA12996/2020	EPI_ISL_420620	Centre Hospitalier de Bourg en Bresse	CNR Virus des Infections Respiratoires - France SUD	2020-03-23	Antonin Bal et al
France/ARA1307/2020	EPI_ISL_420621	Institut des Agents Infectieux (IAI), Hospices Civils de Lyon	CNR Virus des Infections Respiratoires - France SUD	2020-03-24	Antonin Bal et al
France/ARA13074/2020	EPI_ISL_420622	Institut des Agents Infectieux (IAI), Hospices Civils de Lyon	CNR Virus des Infections Respiratoires - France SUD	2020-03-24	Antonin Bal et al
France/ARA13095/2020	EPI_ISL_420623	Institut des Agents Infectieux (IAI), Hospices Civils de Lyon	CNR Virus des Infections Respiratoires - France SUD	2020-03-24	Antonin Bal et al
France/ARA13160/2020	EPI_ISL_420624	Institut des Agents Infectieux (IAI), Hospices Civils de Lyon	CNR Virus des Infections Respiratoires - France SUD	2020-03-24	Antonin Bal et al
France/ARA1322/2020	EPI_ISL_420625	Institut des Agents Infectieux (IAI), Hospices Civils de Lyon	CNR Virus des Infections Respiratoires - France SUD	2020-03-24	Antonin Bal et al
France/B1623/2020	EPI_ISL_418219	CHU - Hôpital Cavale Blanche - Labo. de Virologie	National Reference Center for Viruses of Respiratory Infections, Institut Pasteur, Paris	2020-02-26	Mélanie Albert et al A (https://dx.doi.org/10.1101/2020.04.24.059576)
France/B2330/2020	EPI_ISL_416502	CHRU Pontchaillou - Laboratoire de Virologie	National Reference Center for Viruses of Respiratory Infections, Institut Pasteur, Paris	2020-02-26	Mélnie Albert et al B (https://dx.doi.org/10.1101/2020.04.24.059576)
France/B2334/2020	EPI_ISL_416503	CHRU Pontchaillou - Laboratoire de Virologie	National Reference Center for Viruses of Respiratory Infections, Institut Pasteur, Paris	2020-03-01	Mélnie Albert et al B (https://dx.doi.org/10.1101/2020.04.24.059576)
France/B2335/2020	EPI_ISL_416504	CHRU Pontchaillou - Laboratoire de Virologie	National Reference Center for Viruses of Respiratory Infections, Institut Pasteur, Paris	2020-03-02	Mélnie Albert et al B (https://dx.doi.org/10.1101/2020.04.24.059576)
France/B2336/2020	EPI_ISL_416505	CHRU Pontchaillou - Laboratoire de Virologie	National Reference Center for Viruses of Respiratory Infections, Institut Pasteur, Paris	2020-03-02	Mélnie Albert et al B (https://dx.doi.org/10.1101/2020.04.24.059576)
France/B2337/2020	EPI_ISL_416506	CHRU Pontchaillou - Laboratoire de Virologie	National Reference Center for Viruses of Respiratory Infections, Institut Pasteur, Paris	2020-03-03	Mélnie Albert et al B (https://dx.doi.org/10.1101/2020.04.24.059576)
France/B2340/2020	EPI_ISL_416507	CHRU Pontchaillou - Laboratoire de Virologie	National Reference Center for Viruses of Respiratory Infections, Institut Pasteur, Paris	2020-03-05	Mélnie Albert et al B (https://dx.doi.org/10.1101/2020.04.24.059576)
France/B2343/2020	EPI_ISL_416508	CHRU Pontchaillou - Laboratoire de Virologie	National Reference Center for Viruses of Respiratory Infections, Institut Pasteur, Paris	2020-03-06	Mélnie Albert et al B (https://dx.doi.org/10.1101/2020.04.24.059576)
France/B2344/2020	EPI_ISL_416509	CHRU Pontchaillou - Laboratoire de Virologie	National Reference Center for Viruses of Respiratory Infections, Institut Pasteur, Paris	2020-03-06	Mélnie Albert et al B (https://dx.doi.org/10.1101/2020.04.24.059576)
France/B2346/2020	EPI_ISL_416510	CHRU Pontchaillou - Laboratoire de Virologie	National Reference Center for Viruses of Respiratory Infections, Institut Pasteur, Paris	2020-03-06	Mélnie Albert et al B (https://dx.doi.org/10.1101/2020.04.24.059576)
France/B2348/2020	EPI_ISL_416511	CHRU Pontchaillou - Laboratoire de Virologie	National Reference Center for Viruses of Respiratory Infections, Institut Pasteur, Paris	2020-03-07	Mélnie Albert et al B (https://dx.doi.org/10.1101/2020.04.24.059576)
France/B2349/2020	EPI_ISL_416512	CHRU Pontchaillou - Laboratoire de Virologie	National Reference Center for Viruses of Respiratory Infections, Institut Pasteur, Paris	2020-03-07	Mélnie Albert et al B (https://dx.doi.org/10.1101/2020.04.24.059576)
France/B2351/2020	EPI_ISL_416513	CHRU Pontchaillou - Laboratoire de Virologie	National Reference Center for Viruses of Respiratory Infections, Institut Pasteur, Paris	2020-03-07	Mélnie Albert et al B (https://dx.doi.org/10.1101/2020.04.24.059576)
France/B5310/2020	EPI_ISL_443265	CHU - Hôpital Cavale Blanche - Labo. de Virologie	National Reference Center for Viruses of Respiratory Infections, Institut Pasteur, Paris	2020-04-03	Mélanie Albert et al C
France/B5322/2020	EPI_ISL_443266	CHU - Hôpital Cavale Blanche - Labo. de Virologie	National Reference Center for Viruses of Respiratory Infections, Institut Pasteur, Paris	2020-03-16	Mélanie Albert et al C
France/B5325/2020	EPI_ISL_443267	CHU - Hôpital Cavale Blanche - Labo. de Virologie	National Reference Center for Viruses of Respiratory Infections, Institut Pasteur, Paris	2020-03-18	Mélanie Albert et al C
France/B5336/2020	EPI_ISL_443268	CHU - Hôpital Cavale Blanche - Labo. de Virologie	National Reference Center for Viruses of Respiratory Infections, Institut Pasteur, Paris	2020-03-18	Mélanie Albert et al C
France/B5344/2020	EPI_ISL_443269	CHU - Hôpital Cavale Blanche - Labo. de Virologie	National Reference Center for Viruses of Respiratory Infections, Institut Pasteur, Paris	2020-03-21	Mélanie Albert et al C
France/B5353/2020	EPI_ISL_443270	CHU - Hôpital Cavale Blanche - Labo. de Virologie	National Reference Center for Viruses of Respiratory Infections, Institut Pasteur, Paris	2020-03-23	Mélanie Albert et al C
France/B5355/2020	EPI_ISL_443271	CHU - Hôpital Cavale Blanche - Labo. de Virologie	National Reference Center for Viruses of Respiratory Infections, Institut Pasteur, Paris	2020-03-24	Mélanie Albert et al C
France/B5359/2020	EPI_ISL_443272	CHU - Hôpital Cavale Blanche - Labo. de Virologie	National Reference Center for Viruses of Respiratory Infections, Institut Pasteur, Paris	2020-03-24	Mélanie Albert et al C
France/B5363/2020	EPI_ISL_443273	CHU - Hôpital Cavale Blanche - Labo. de Virologie	National Reference Center for Viruses of Respiratory Infections, Institut Pasteur, Paris	2020-03-24	Mélanie Albert et al C
France/B5374/2020	EPI_ISL_443274	CHU - Hôpital Cavale Blanche - Labo. de Virologie	National Reference Center for Viruses of Respiratory Infections, Institut Pasteur, Paris	2020-03-25	Mélanie Albert et al C
France/B5397/2020	EPI_ISL_443275	CHU - Hôpital Cavale Blanche - Labo. de Virologie	National Reference Center for Viruses of Respiratory Infections, Institut Pasteur, Paris	2020-03-27	Mélanie Albert et al C
France/B5400/2020	EPI_ISL_443276	CHU - Hôpital Cavale Blanche - Labo. de Virologie	National Reference Center for Viruses of Respiratory Infections, Institut Pasteur, Paris	2020-03-27	Mélanie Albert et al C
France/B5413/2020	EPI_ISL_443277	CHU - Hôpital Cavale Blanche - Labo. de Virologie	National Reference Center for Viruses of Respiratory Infections, Institut Pasteur, Paris	2020-03-28	Mélanie Albert et al C
France/B5415/2020	EPI_ISL_443278	CHU - Hôpital Cavale Blanche - Labo. de Virologie	National Reference Center for Viruses of Respiratory Infections, Institut Pasteur, Paris	2020-03-29	Mélanie Albert et al C
France/B5434/2020	EPI_ISL_443279	CHU - Hôpital Cavale Blanche - Labo. de Virologie	National Reference Center for Viruses of Respiratory Infections, Institut Pasteur, Paris	2020-04-01	Mélanie Albert et al C
France/B5447/2020	EPI_ISL_443280	CHU - Hôpital Cavale Blanche - Labo. de Virologie	National Reference Center for Viruses of Respiratory Infections, Institut Pasteur, Paris	2020-04-01	Mélanie Albert et al C
France/B5456/2020	EPI_ISL_443281	CHU - Hôpital Cavale Blanche - Labo. de Virologie	National Reference Center for Viruses of Respiratory Infections, Institut Pasteur, Paris	2020-04-03	Mélanie Albert et al C
France/B5458/2020	EPI_ISL_443282	CHU - Hôpital Cavale Blanche - Labo. de Virologie	National Reference Center for Viruses of Respiratory Infections, Institut Pasteur, Paris	2020-03-22	Mélanie Albert et al C
France/B5465/2020	EPI_ISL_443283	CHU - Hôpital Cavale Blanche - Labo. de Virologie	National Reference Center for Viruses of Respiratory Infections, Institut Pasteur, Paris	2020-03-23	Mélanie Albert et al C
France/B5678/2020	EPI_ISL_443289	CHRU Pontchaillou - Laboratoire de Virologie	National Reference Center for Viruses of Respiratory Infections, Institut Pasteur, Paris	2020-03-27	Mélanie Albert et al C
France/B5681/2020	EPI_ISL_443290	CHRU Pontchaillou - Laboratoire de Virologie	National Reference Center for Viruses of Respiratory Infections, Institut Pasteur, Paris	2020-03-31	Mélanie Albert et al C
France/B5685/2020	EPI_ISL_443291	CHRU Pontchaillou - Laboratoire de Virologie	National Reference Center for Viruses of Respiratory Infections, Institut Pasteur, Paris	2020-04-02	Mélanie Albert et al C
France/B5687/2020	EPI_ISL_443292	CHRU Pontchaillou - Laboratoire de Virologie	National Reference Center for Viruses of Respiratory Infections, Institut Pasteur, Paris	2020-04-03	Mélanie Albert et al C
France/B5688/2020	EPI_ISL_443293	CHRU Pontchaillou - Laboratoire de Virologie	National Reference Center for Viruses of Respiratory Infections, Institut Pasteur, Paris	2020-04-03	Mélanie Albert et al C
France/B5689/2020	EPI_ISL_443294	CHRU Pontchaillou - Laboratoire de Virologie	National Reference Center for Viruses of Respiratory Infections, Institut Pasteur, Paris	2020-04-06	Mélanie Albert et al C
France/B6041/2020	EPI_ISL_443313	Cabinet Médical	National Reference Center for Viruses of Respiratory Infections, Institut Pasteur, Paris	2020-03-05	Mélanie Albert et al C
France/BFC2094/2020	EPI_ISL_415651		National Reference Center for Viruses of Respiratory Infections, Institut Pasteur, Paris	2020-03-05	Mélnie Albert et al B (https://dx.doi.org/10.1101/2020.04.24.059576)
France/BFC2147/2020	EPI_ISL_415652		National Reference Center for Viruses of Respiratory Infections, Institut Pasteur, Paris	2020-03-05	Mélnie Albert et al B (https://dx.doi.org/10.1101/2020.04.24.059576)
France/BFC2709/2020	EPI_ISL_420038	Sentinelles network	National Reference Center for Viruses of Respiratory Infections, Institut Pasteur, Paris	2020-03-17	Mélanie Albert et al A (https://dx.doi.org/10.1101/2020.04.24.059576)
France/BFC5011/2020	EPI_ISL_443261	CHU de Dijon - Laboratoire de Virologie	National Reference Center for Viruses of Respiratory Infections, Institut Pasteur, Paris	2020-04-03	Mélanie Albert et al C
France/BFC5012/2020	EPI_ISL_443262	CHU de Dijon - Laboratoire de Virologie	National Reference Center for Viruses of Respiratory Infections, Institut Pasteur, Paris	2020-04-02	Mélanie Albert et al C
France/BFC5017/2020	EPI_ISL_443263	CHU de Dijon - Laboratoire de Virologie	National Reference Center for Viruses of Respiratory Infections, Institut Pasteur, Paris	2020-04-02	Mélanie Albert et al C
France/BFC5019/2020	EPI_ISL_443264	CHU de Dijon - Laboratoire de Virologie	National Reference Center for Viruses of Respiratory Infections, Institut Pasteur, Paris	2020-04-02	Mélanie Albert et al C
France/Bourg-en-Bresse_06678/2020	EPI_ISL_416757	Centre Hospitalier de Bourg en Bresse	CNR Virus des Infections Respiratoires - France SUD	2020-03-07	Bal et al B
France/Bourg-en-Bresse_06813/2020	EPI_ISL_417340	Centre Hospitalier de Bourg en Bresse	CNR Virus des Infections Respiratoires - France SUD	2020-03-07	Antonin Bal et al
France/CIBU200107/2020	EPI_ISL_437689	Laboratory for Urgent Response to Biological Threats	Institut Pasteur CIBU / ERI	2020-02-27	V. Caro et al
France/CIBU200132/2020	EPI_ISL_437690	Laboratory for Urgent Response to Biological Threats	Institut Pasteur CIBU /ERI	2020-02-29	V. Caro et al
France/Clermont-Ferrand_650/2020	EPI_ISL_416752	CHU Gabriel Montpied	CNR Virus des Infections Respiratoires - France SUD	2020-03-04	Bal et al B
France/Clermont-Ferrand_651/2020	EPI_ISL_416751	CHU Gabriel Montpied	CNR Virus des Infections Respiratoires - France SUD	2020-03-05	Bal et al B
France/CVL2000/2020	EPI_ISL_418222	CHRU Bretonneau - Serv. Bacterio-Virol.	National Reference Center for Viruses of Respiratory Infections, Institut Pasteur, Paris	2020-03-04	Mélanie Albert et al A (https://dx.doi.org/10.1101/2020.04.24.059576)
France/CVL3365/2020	EPI_ISL_421514	Sentinelles network	National Reference Center for Viruses of Respiratory Infections, Institut Pasteur, Paris	2020-03-20	Mélanie Albert et al A (https://dx.doi.org/10.1101/2020.04.24.059576)
France/CVL4430/2020	EPI_ISL_443312	Cabinet Médical	National Reference Center for Viruses of Respiratory Infections, Institut Pasteur, Paris	2020-03-24	Mélanie Albert et al C
France/GE1583/2020	EPI_ISL_414623	Laboratoire de Virologie Institut de Virologie - INSERM U 1109 Hôpitaux Universitaires de Strasbourg	National Reference Center for Viruses of Respiratory Infections, Institut Pasteur, Paris	2020-02-25	Mélnie Albert et al A
France/GE1973/2020	EPI_ISL_414631	Hôpital Robert Debré Laboratoire de Virologie	National Reference Center for Viruses of Respiratory Infections, Institut Pasteur, Paris	2020-03-04	Mélnie Albert et al B (https://dx.doi.org/10.1101/2020.04.24.059576)
France/GE1977/2020	EPI_ISL_414632	Hôpital Robert Debré Laboratoire de Virologie	National Reference Center for Viruses of Respiratory Infections, Institut Pasteur, Paris	2020-03-04	Mélnie Albert et al B (https://dx.doi.org/10.1101/2020.04.24.059576)
France/GE2720/2020	EPI_ISL_420039	L'Air du Temps	National Reference Center for Viruses of Respiratory Infections, Institut Pasteur, Paris	2020-03-12	Mélanie Albert et al A (https://dx.doi.org/10.1101/2020.04.24.059576)

France/GE2722/2020	EPI_ISL_420040	L'Air du Temps	National Reference Center for Viruses of Respiratory Infections, Institut Pasteur, Paris	2020-03-12	Mélanie Albert et al A (https://dx.doi.org/10.1101/2020.04.24.059576)
France/GE2843/2020	EPI_ISL_420045	Sentinelles network	National Reference Center for Viruses of Respiratory Infections, Institut Pasteur, Paris	2020-03-17	Mélanie Albert et al A (https://dx.doi.org/10.1101/2020.04.24.059576)
France/GE3067/2020	EPI_ISL_420055	Sentinelles network	National Reference Center for Viruses of Respiratory Infections, Institut Pasteur, Paris	2020-03-17	Mélanie Albert et al A (https://dx.doi.org/10.1101/2020.04.24.059576)
France/GE3372/2020	EPI_ISL_428348	Maison de Santé du Val d'Ormois	National Reference Center for Viruses of Respiratory Infections, Institut Pasteur, Paris	2020-03-20	Mélanie Albert et al C
France/GE4420/2020	EPI_ISL_443310	Centre de santé Filieris	National Reference Center for Viruses of Respiratory Infections, Institut Pasteur, Paris	2020-03-20	Mélanie Albert et al C
France/GE6058/2020	EPI_ISL_443308	Plaisance	National Reference Center for Viruses of Respiratory Infections, Institut Pasteur, Paris	2020-04-15	Mélanie Albert et al C
France/HF1463/2020	EPI_ISL_429968	Centre Hospitalier Compiègne Laboratoire de Biologie	National Reference Center for Viruses of Respiratory Infections, Institut Pasteur, Paris	2020-02-21	Mélanie Albert et al A (https://dx.doi.org/10.1101/2020.04.24.059576)
France/HF1465/2020	EPI_ISL_418218	Centre Hospitalier Compiègne Laboratoire de Biologie	National Reference Center for Viruses of Respiratory Infections, Institut Pasteur, Paris	2020-02-21	Mélanie Albert et al C
France/HF1645/2020	EPI_ISL_418220	Centre Hospitalier Compiègne Laboratoire de Biologie	National Reference Center for Viruses of Respiratory Infections, Institut Pasteur, Paris	2020-02-28	Mélanie Albert et al A (https://dx.doi.org/10.1101/2020.04.24.059576)
France/HF1684/2020	EPI_ISL_414626		National Reference Center for Viruses of Respiratory Infections, Institut Pasteur, Paris	2020-02-29	Mélnie Albert et al B (https://dx.doi.org/10.1101/2020.04.24.059576)
France/HF1795/2020	EPI_ISL_414627	Centre Hospitalier Compiègne Laboratoire de Biologie	National Reference Center for Viruses of Respiratory Infections, Institut Pasteur, Paris	2020-03-02	Mélnie Albert et al B (https://dx.doi.org/10.1101/2020.04.24.059576)
France/HF1813/2020	EPI_ISL_418221	Centre Hospitalier Compiègne Laboratoire de Biologie	National Reference Center for Viruses of Respiratory Infections, Institut Pasteur, Paris	2020-03-02	Mélanie Albert et al A (https://dx.doi.org/10.1101/2020.04.24.059576)
France/HF1870/2020	EPI_ISL_414629	Centre Hospitalier Compiègne Laboratoire de Biologie	National Reference Center for Viruses of Respiratory Infections, Institut Pasteur, Paris	2020-03-03	Mélnie Albert et al B (https://dx.doi.org/10.1101/2020.04.24.059576)
France/HF1871/2020	EPI_ISL_414630	Centre Hospitalier Compiègne Laboratoire de Biologie	National Reference Center for Viruses of Respiratory Infections, Institut Pasteur, Paris	2020-03-03	Mélnie Albert et al B (https://dx.doi.org/10.1101/2020.04.24.059576)
France/HF1986/2020	EPI_ISL_414634	Centre Hospitalier Compiègne Laboratoire de Biologie	National Reference Center for Viruses of Respiratory Infections, Institut Pasteur, Paris	2020-03-04	Mélnie Albert et al B (https://dx.doi.org/10.1101/2020.04.24.059576)
France/HF1988/2020	EPI_ISL_414635	Centre Hospitalier Compiègne Laboratoire de Biologie	National Reference Center for Viruses of Respiratory Infections, Institut Pasteur, Paris	2020-03-04	Mélnie Albert et al B (https://dx.doi.org/10.1101/2020.04.24.059576)
France/HF1989/2020	EPI_ISL_414636	Centre Hospitalier Compiègne Laboratoire de Biologie	National Reference Center for Viruses of Respiratory Infections, Institut Pasteur, Paris	2020-03-04	Mélnie Albert et al B (https://dx.doi.org/10.1101/2020.04.24.059576)
France/HF1993/2020	EPI_ISL_414637	Centre Hospitalier Compiègne Laboratoire de Biologie	National Reference Center for Viruses of Respiratory Infections, Institut Pasteur, Paris	2020-03-04	Mélnie Albert et al B (https://dx.doi.org/10.1101/2020.04.24.059576)
France/HF1995/2020	EPI_ISL_414638	Centre Hospitalier Compiègne Laboratoire de Biologie	National Reference Center for Viruses of Respiratory Infections, Institut Pasteur, Paris	2020-03-04	Mélnie Albert et al B (https://dx.doi.org/10.1101/2020.04.24.059576)
France/HF2039/2020	EPI_ISL_415649		National Reference Center for Viruses of Respiratory Infections, Institut Pasteur, Paris	2020-03-05	Mélnie Albert et al B (https://dx.doi.org/10.1101/2020.04.24.059576)
France/HF2060/2020	EPI_ISL_418223	Centre Hospitalier Compiègne Laboratoire de Biologie	National Reference Center for Viruses of Respiratory Infections, Institut Pasteur, Paris	2020-03-05	Mélanie Albert et al A (https://dx.doi.org/10.1101/2020.04.24.059576)
France/HF2150/2020	EPI_ISL_418224	Centre Hospitalier Compiègne Laboratoire de Biologie	National Reference Center for Viruses of Respiratory Infections, Institut Pasteur, Paris	2020-03-08	Mélanie Albert et al A (https://dx.doi.org/10.1101/2020.04.24.059576)
France/HF2151/2020	EPI_ISL_415653	Centre Hospitalier Compiègne Laboratoire de Biologie	National Reference Center for Viruses of Respiratory Infections, Institut Pasteur, Paris	2020-03-08	Mélnie Albert et al B (https://dx.doi.org/10.1101/2020.04.24.059576)
France/HF2155/2020	EPI_ISL_418225	Centre Hospitalier Compiègne Laboratoire de Biologie	National Reference Center for Viruses of Respiratory Infections, Institut Pasteur, Paris	2020-03-08	Mélanie Albert et al A (https://dx.doi.org/10.1101/2020.04.24.059576)
France/HF2174/2020	EPI_ISL_415654	Centre Hospitalier Compiègne Laboratoire de Biologie	National Reference Center for Viruses of Respiratory Infections, Institut Pasteur, Paris	2020-03-09	Mélnie Albert et al B (https://dx.doi.org/10.1101/2020.04.24.059576)
France/HF2196/2020	EPI_ISL_416493	CH Jean de Navarre Laboratoire de Biologie	National Reference Center for Viruses of Respiratory Infections, Institut Pasteur, Paris	2020-03-08	Mélnie Albert et al B (https://dx.doi.org/10.1101/2020.04.24.059576)
France/HF2234/2020	EPI_ISL_416495	Centre Hospitalier Compiègne Laboratoire de Biologie	National Reference Center for Viruses of Respiratory Infections, Institut Pasteur, Paris	2020-03-10	Mélnie Albert et al B (https://dx.doi.org/10.1101/2020.04.24.059576)
France/HF2237/2020	EPI_ISL_416496	Centre Hospitalier Compiègne Laboratoire de Biologie	National Reference Center for Viruses of Respiratory Infections, Institut Pasteur, Paris	2020-03-10	Mélnie Albert et al B (https://dx.doi.org/10.1101/2020.04.24.059576)
France/HF2239/2020	EPI_ISL_416497	Centre Hospitalier Compiègne Laboratoire de Biologie	National Reference Center for Viruses of Respiratory Infections, Institut Pasteur, Paris	2020-03-10	Mélnie Albert et al B (https://dx.doi.org/10.1101/2020.04.24.059576)
France/HF2306/2020	EPI_ISL_421500	CH Compiègne Laboratoire de Biologie	National Reference Center for Viruses of Respiratory Infections, Institut Pasteur, Paris	2020-03-11	Mélanie Albert et al C
France/HF2381/2020	EPI_ISL_418226	EHPAD - Résidences les Cèdres	National Reference Center for Viruses of Respiratory Infections, Institut Pasteur, Paris	2020-03-09	Mélanie Albert et al A (https://dx.doi.org/10.1101/2020.04.24.059576)
France/HF2393/2020	EPI_ISL_418227	Centre Hospitalier Compiègne Laboratoire de Biologie	National Reference Center for Viruses of Respiratory Infections, Institut Pasteur, Paris	2020-03-12	Mélanie Albert et al A (https://dx.doi.org/10.1101/2020.04.24.059576)
France/HF2405/2020	EPI_ISL_418228	Centre Hospitalier Compiègne Laboratoire de Biologie	National Reference Center for Viruses of Respiratory Infections, Institut Pasteur, Paris	2020-03-12	Mélanie Albert et al A (https://dx.doi.org/10.1101/2020.04.24.059576)
France/HF2496/2020	EPI_ISL_418231	Centre Hospitalier Compiègne Laboratoire de Biologie	National Reference Center for Viruses of Respiratory Infections, Institut Pasteur, Paris	2020-03-15	Mélanie Albert et al A (https://dx.doi.org/10.1101/2020.04.24.059576)
France/HF2586/2020	EPI_ISL_418236	Centre Hospitalier Compiègne Laboratoire de Biologie	National Reference Center for Viruses of Respiratory Infections, Institut Pasteur, Paris	2020-03-16	Mélanie Albert et al A (https://dx.doi.org/10.1101/2020.04.24.059576)
France/HF2595/2020	EPI_ISL_418237	Centre Hospitalier Compiègne Laboratoire de Biologie	National Reference Center for Viruses of Respiratory Infections, Institut Pasteur, Paris	2020-03-16	Mélanie Albert et al A (https://dx.doi.org/10.1101/2020.04.24.059576)
France/HF2597/2020	EPI_ISL_418238	Centre Hospitalier Compiègne Laboratoire de Biologie	National Reference Center for Viruses of Respiratory Infections, Institut Pasteur, Paris	2020-03-16	Mélanie Albert et al A (https://dx.doi.org/10.1101/2020.04.24.059576)
France/HF2601/2020	EPI_ISL_418239	Centre Hospitalier Compiègne Laboratoire de Biologie	National Reference Center for Viruses of Respiratory Infections, Institut Pasteur, Paris	2020-03-16	Mélanie Albert et al A (https://dx.doi.org/10.1101/2020.04.24.059576)
France/HF2748/2020	EPI_ISL_420041	CH Compiègne Laboratoire de Biologie	National Reference Center for Viruses of Respiratory Infections, Institut Pasteur, Paris	2020-03-17	Mélanie Albert et al A (https://dx.doi.org/10.1101/2020.04.24.059576)
France/HF2797/2020	EPI_ISL_420044	CH Jean de Navarre Laboratoire de Biologie	National Reference Center for Viruses of Respiratory Infections, Institut Pasteur, Paris	2020-03-18	Mélanie Albert et al A (https://dx.doi.org/10.1101/2020.04.24.059576)
France/HF2946/2020	EPI_ISL_420049	CH Compiègne Laboratoire de Biologie	National Reference Center for Viruses of Respiratory Infections, Institut Pasteur, Paris	2020-03-19	Mélanie Albert et al A (https://dx.doi.org/10.1101/2020.04.24.059576)
France/HF2948/2020	EPI_ISL_420050	CH Compiègne Laboratoire de Biologie	National Reference Center for Viruses of Respiratory Infections, Institut Pasteur, Paris	2020-03-19	Mélanie Albert et al A (https://dx.doi.org/10.1101/2020.04.24.059576)
France/HF3036/2020	EPI_ISL_420053	CH Jean de Navarre Laboratoire de Biologie	National Reference Center for Viruses of Respiratory Infections, Institut Pasteur, Paris	2020-03-19	Mélanie Albert et al A (https://dx.doi.org/10.1101/2020.04.24.059576)
France/HF3138/2020	EPI_ISL_420056	CH Compiègne Laboratoire de Biologie	National Reference Center for Viruses of Respiratory Infections, Institut Pasteur, Paris	2020-03-22	Mélanie Albert et al A (https://dx.doi.org/10.1101/2020.04.24.059576)
France/HF3141/2020	EPI_ISL_420057	CH Compiègne Laboratoire de Biologie	National Reference Center for Viruses of Respiratory Infections, Institut Pasteur, Paris	2020-03-22	Mélanie Albert et al A (https://dx.doi.org/10.1101/2020.04.24.059576)
France/HF3290/2020	EPI_ISL_421509	CH Compiègne Laboratoire de Biologie	National Reference Center for Viruses of Respiratory Infections, Institut Pasteur, Paris	2020-03-23	Mélanie Albert et al C
France/HF3293/2020	EPI_ISL_421510	CH Compiègne Laboratoire de Biologie	National Reference Center for Viruses of Respiratory Infections, Institut Pasteur, Paris	2020-03-23	Mélanie Albert et al C
France/HF3295/2020	EPI_ISL_421511	CH Compiègne Laboratoire de Biologie	National Reference Center for Viruses of Respiratory Infections, Institut Pasteur, Paris	2020-03-23	Mélanie Albert et al C
France/HF3419/2020	EPI_ISL_428350	CH Jean de Navarre Laboratoire de Biologie	National Reference Center for Viruses of Respiratory Infections, Institut Pasteur, Paris	2020-03-24	Mélanie Albert et al C
France/HF3534/2020	EPI_ISL_428353	CH Compiègne Laboratoire de Biologie	National Reference Center for Viruses of Respiratory Infections, Institut Pasteur, Paris	2020-03-24	Mélanie Albert et al C
France/HF3598/2020	EPI_ISL_428358	CH Jeanne de Navarre Laboratoire de Biologie	National Reference Center for Viruses of Respiratory Infections, Institut Pasteur, Paris	2020-03-24	Mélanie Albert et al C
France/HF3668/2020	EPI_ISL_443316	CH Compiègne Laboratoire de Biologie	National Reference Center for Viruses of Respiratory Infections, Institut Pasteur, Paris	2020-03-25	Mélanie Albert et al C
France/HF3669/2020	EPI_ISL_443309	CH Compiègne Laboratoire de Biologie	National Reference Center for Viruses of Respiratory Infections, Institut Pasteur, Paris	2020-03-25	Mélanie Albert et al C
France/HF3677/2020	EPI_ISL_428359	CH Compiègne Laboratoire de Biologie	National Reference Center for Viruses of Respiratory Infections, Institut Pasteur, Paris	2020-03-23	Mélanie Albert et al C
France/HF3678/2020	EPI_ISL_428360	CH Compiègne Laboratoire de Biologie	National Reference Center for Viruses of Respiratory Infections, Institut Pasteur, Paris	2020-03-25	Mélanie Albert et al C
France/HF4220/2020	EPI_ISL_428366	CH Jeanne de Navarre Laboratoire de Biologie	National Reference Center for Viruses of Respiratory Infections, Institut Pasteur, Paris	2020-03-30	Mélanie Albert et al C
France/HF6466/2020	EPI_ISL_443315	Château de la Source	National Reference Center for Viruses of Respiratory Infections, Institut Pasteur, Paris	2020-04-02	Mélanie Albert et al C
France/IDF0372/2020	EPI_ISL_406596	Department of Infectious and Tropical Diseases, Bichat Claude Bernard Hospital, Paris	National Reference Center for Viruses of Respiratory Infections, Institut Pasteur, Paris	2020-01-23	Mélanie Albert et al A (https://dx.doi.org/10.1101/2020.04.24.059576)
France/IDF0373/2020	EPI_ISL_406597	Department of Infectious and Tropical Diseases, Bichat Claude Bernard Hospital, Paris	National Reference Center for Viruses of Respiratory Infections, Institut Pasteur, Paris	2020-01-23	Mélanie Albert et al A (https://dx.doi.org/10.1101/2020.04.24.059576)
France/IDF0386-islP1/2020	EPI_ISL_411219	Department of Infectious and Tropical Diseases, Bichat Claude Bernard Hospital, Paris	Laboratoire Virpath, CIRI U111, UCBL1, INSERM, CNRS, ENS Lyon	2020-01-28	Olivier Terrier et al (https://dx.doi.org/10.2807/1560-7917.ES.2020.25.13.2000305)
France/IDF0515/2020	EPI_ISL_408430	Department of Infectious and Tropical Diseases, Bichat Claude Bernard Hospital, Paris	National Reference Center for Viruses of Respiratory Infections, Institut Pasteur, Paris	2020-01-29	Mélanie Albert et al A (https://dx.doi.org/10.1101/2020.04.24.059576)
France/IDF1980/2020	EPI_ISL_414633	Centre Hospitalier René Dubois Laboratoire de Microbiologie - Bât A	National Reference Center for Viruses of Respiratory Infections, Institut Pasteur, Paris	2020-03-04	Mélnie Albert et al B (https://dx.doi.org/10.1101/2020.04.24.059576)
France/IDF2075/2020	EPI_ISL_415650	Hôpital Instruction des Armées - BEGIN	National Reference Center for Viruses of Respiratory Infections, Institut Pasteur, Paris	2020-03-02	Mélnie Albert et al B (https://dx.doi.org/10.1101/2020.04.24.059576)
France/IDF2251/2020	EPI_ISL_428355	Institut Médico légal- Hop R. Poincaré	National Reference Center for Viruses of Respiratory Infections, Institut Pasteur, Paris	2020-03-11	Mélanie Albert et al C
France/IDF2252/2020	EPI_ISL_428356	Institut Médico légal- Hop R. Poincaré	National Reference Center for Viruses of Respiratory Infections, Institut Pasteur, Paris	2020-03-11	Mélanie Albert et al C
France/IDF2253/2020	EPI_ISL_428357	Institut Médico légal- Hop R. Poincaré	National Reference Center for Viruses of Respiratory Infections, Institut Pasteur, Paris	2020-03-11	Mélanie Albert et al C
France/IDF2256/2020	EPI_ISL_416498	Institut Médico légal- Hop R. Poincaré	National Reference Center for Viruses of Respiratory Infections, Institut Pasteur, Paris	2020-03-11	Mélnie Albert et al B (https://dx.doi.org/10.1101/2020.04.24.059576)
France/IDF2278/2020	EPI_ISL_416499	LABM GH nord Essonne	National Reference Center for Viruses of Respiratory Infections, Institut Pasteur, Paris	2020-03-11	Mélnie Albert et al B (https://dx.doi.org/10.1101/2020.04.24.059576)
France/IDF2279/2020	EPI_ISL_416500	LABM GH nord Essonne	National Reference Center for Viruses of Respiratory Infections, Institut Pasteur, Paris	2020-03-11	Mélnie Albert et al B (https://dx.doi.org/10.1101/2020.04.24.059576)
France/IDF2284/2020	EPI_ISL_416501	Hopital franco britannique - Service des Urgences	National Reference Center for Viruses of Respiratory Infections, Institut Pasteur, Paris	2020-03-10	Mélnie Albert et al B (https://dx.doi.org/10.1101/2020.04.24.059576)
France/IDF2359/2020	EPI_ISL_421501	Service de Biologie Médicale - BP 125	National Reference Center for Viruses of Respiratory Infections, Institut Pasteur, Paris	2020-03-12	Mélanie Albert et al C
France/IDF2410/2020	EPI_ISL_418229	Hopital franco britannique - Laboratoire	National Reference Center for Viruses of Respiratory Infections, Institut Pasteur, Paris	2020-03-12	Mélanie Albert et al A (https://dx.doi.org/10.1101/2020.04.24.059576)
France/IDF2412/2020	EPI_ISL_421502	Parc des Dames	National Reference Center for Viruses of Respiratory Infections, Institut Pasteur, Paris	2020-03-12	Mélanie Albert et al C
France/IDF2414/2020	EPI_ISL_421503	Parc des Dames	National Reference Center for Viruses of Respiratory Infections, Institut Pasteur, Paris	2020-03-12	Mélanie Albert et al C
France/IDF2420/2020	EPI_ISL_418230	Clinique AVERAY LA BROUSTE, Med. Polyvalente	National Reference Center for Viruses of Respiratory Infections, Institut Pasteur, Paris	2020-03-13	Mélanie Albert et al A (https://dx.doi.org/10.1101/2020.04.24.059576)
France/IDF2532/2020	EPI_ISL_418232	Service des Urgences	National Reference Center for Viruses of Respiratory Infections, Institut Pasteur, Paris	2020-03-15	Mélanie Albert et al A (https://dx.doi.org/10.1101/2020.04.24.059576)
France/IDF2533/2020	EPI_ISL_418233	Service des Urgences	National Reference Center for Viruses of Respiratory Infections, Institut Pasteur, Paris	2020-03-15	Mélanie Albert et al A (https://dx.doi.org/10.1101/2020.04.24.059576)
France/IDF2534/2020	EPI_ISL_418234	LABM GH nord Essonne	National Reference Center for Viruses of Respiratory Infections, Institut Pasteur, Paris	2020-03-14	Mélanie Albert et al A (https://dx.doi.org/10.1101/2020.04.24.059576)
France/IDF2536/2020	EPI_ISL_421504	Service de Biologie Médicale - BP 125	National Reference Center for Viruses of Respiratory Infections, Institut Pasteur, Paris	2020-03-14	Mélanie Albert et al C
France/IDF2548/2020	EPI_ISL_421505	Service de Biologie Médicale - BP 125	National Reference Center for Viruses of Respiratory Infections, Institut Pasteur, Paris	2020-03-15	Mélanie Albert et al C
France/IDF2561/2020	EPI_ISL_418235	Cabinet médical	National Reference Center for Viruses of Respiratory Infections, Institut Pasteur, Paris	2020-03-16	Mélanie Albert et al A (https://dx.doi.org/10.1101/2020.04.24.059576)
France/IDF2684/2020	EPI_ISL_418240	LABM GH nord Essonne	National Reference Center for Viruses of Respiratory Infections, Institut Pasteur, Paris	2020-03-16	Mélanie Albert et al A (https://dx.doi.org/10.1101/2020.04.24.059576)
France/IDF2768/2020	EPI_ISL_420042	Service de Biologie clinique	National Reference Center for Viruses of Respiratory Infections, Institut Pasteur, Paris	2020-03-17	Mélanie Albert et al A (https://dx.doi.org/10.1101/2020.04.24.059576)
France/IDF2792/2020	EPI_ISL_420043	CMIP	National Reference Center for Viruses of Respiratory Infections, Institut Pasteur, Paris	2020-03-18	Mélanie Albert et al A (https://dx.doi.org/10.1101/2020.04.24.059576)
France/IDF2848/2020	EPI_ISL_420046	Résidence Villa Caroline	National Reference Center for Viruses of Respiratory Infections, Institut Pasteur, Paris	2020-03-17	Mélanie Albert et al A (https://dx.doi.org/10.1101/2020.04.24.059576)
France/IDF2849/2020	EPI_ISL_420047	Résidence Villa Caroline	National Reference Center for Viruses of Respiratory Infections, Institut Pasteur, Paris	2020-03-17	Mélanie Albert et al A (https://dx.doi.org/10.1101/2020.04.24.059576)
France/IDF2936/2020	EPI_ISL_420048	Service de Biologie Médicale - BP 125	National Reference Center for Viruses of Respiratory Infections, Institut Pasteur, Paris	2020-03-18	Mélanie Albert et al A (https://dx.doi.org/10.1101/2020.04.24.059576)
France/IDF2989/2020	EPI_ISL_420051	Résidence Eleusis	National Reference Center for Viruses of Respiratory Infections, Institut Pasteur, Paris	2020-03-19	Mélanie Albert et al A (https://dx.doi.org/10.1101/2020.04.24.059576)
France/IDF3029/2020	EPI_ISL_420052	Résidence les Marines	National Reference Center for Viruses of Respiratory Infections, Institut Pasteur, Paris	2020-03-20	Mélanie Albert et al A (https://dx.doi.org/10.1101/2020.04.24.059576)
France/IDF3040/2020	EPI_ISL_420054	Résidence de maintenon	National Reference Center for Viruses of Respiratory Infections, Institut Pasteur, Paris	2020-03-20	Mélanie Albert et al A (https://dx.doi.org/10.1101/2020.04.24.059576)
France/IDF3081/2020	EPI_ISL_443300	Cabinet Médical	National Reference Center for Viruses of Respiratory Infections, Institut Pasteur, Paris	2020-03-17	Mélanie Albert et al C
France/IDF3163/2020	EPI_ISL_420058	Service de Biologie Médicale - BP 125	National Reference Center for Viruses of Respiratory Infections, Institut Pasteur, Paris	2020-03-20	Mélanie Albert et al A (https://dx.doi.org/10.1101/2020.04.24.059576)
France/IDF3165/2020	EPI_ISL_420059	Service de Biologie Médicale - BP 125	National Reference Center for Viruses of Respiratory Infections, Institut Pasteur, Paris	2020-03-21	Mélanie Albert et al A (https://dx.doi.org/10.1101/2020.04.24.059576)
France/IDF3170/2020	EPI_ISL_420060	Service de Biologie Médicale - BP 125	National Reference Center for Viruses of Respiratory Infections, Institut Pasteur, Paris	2020-03-20	Mélanie Albert et al A (https://dx.doi.org/10.1101/2020.04.24.059576)
France/IDF3212/2020	EPI_ISL_420061	CMIP	National Reference Center for Viruses of Respiratory Infections, Institut Pasteur, Paris	2020-03-23	Mélanie Albert et al A (https://dx.doi.org/10.1101/2020.04.24.059576)
France/IDF3218/2020	EPI_ISL_420062	Service de Biologie Médicale - BP 125	National Reference Center for Viruses of Respiratory Infections, Institut Pasteur, Paris	2020-03-22	Mélanie Albert et al A (https://dx.doi.org/10.1101/2020.04.24.059576)
France/IDF3230/2020	EPI_ISL_420063	Labo BM - Site de Juvisy - Hopital Général	National Reference Center for Viruses of Respiratory Infections, Institut Pasteur, Paris	2020-03-22	Mélanie Albert et al A (https://dx.doi.org/10.1101/2020.04.24.059576)
France/IDF3235/2020	EPI_ISL_420064	Service de Biologie Médicale - BP 125	National Reference Center for Viruses of Respiratory Infections, Institut Pasteur, Paris	2020-03-23	Mélanie Albert et al A (https://dx.doi.org/10.1101/2020.04.24.059576)
France/IDF3236/2020	EPI_ISL_421506	Service de Biologie Médicale - BP 125	National Reference Center for Viruses of Respiratory Infections, Institut Pasteur, Paris	2020-03-21	Mélanie Albert et al C
France/IDF3274/2020	EPI_ISL_421507	Le Château de Seine-Port	National Reference Center for Viruses of Respiratory Infections, Institut Pasteur, Paris	2020-03-23	Mélanie Albert et al C

France/IDF3276/2020	EPI_ISL_421508	Le Château de Seine-Port	National Reference Center for Viruses of Respiratory Infections, Institut Pasteur, Paris	2020-03-23	Mélanie Albert et al C
France/IDF3324/2020	EPI_ISL_421512	Service de Biologie Médicale - BP 125	National Reference Center for Viruses of Respiratory Infections, Institut Pasteur, Paris	2020-03-23	Mélanie Albert et al C
France/IDF3345/2020	EPI_ISL_421513	Service de Biologie clinique	National Reference Center for Viruses of Respiratory Infections, Institut Pasteur, Paris	2020-03-23	Mélanie Albert et al C
France/IDF3359/2020	EPI_ISL_443301	Cabinet Médical	National Reference Center for Viruses of Respiratory Infections, Institut Pasteur, Paris	2020-03-23	Mélanie Albert et al C
France/IDF3368/2020	EPI_ISL_443302	Cabinet Médical	National Reference Center for Viruses of Respiratory Infections, Institut Pasteur, Paris	2020-03-23	Mélanie Albert et al C
France/IDF3384/2020	EPI_ISL_428347	Service de Biologie Médicale - BP 125	National Reference Center for Viruses of Respiratory Infections, Institut Pasteur, Paris	2020-03-23	Mélanie Albert et al C
France/IDF3386/2020	EPI_ISL_428349	Service de Biologie Médicale - BP 125	National Reference Center for Viruses of Respiratory Infections, Institut Pasteur, Paris	2020-03-23	Mélanie Albert et al C
France/IDF3509/2020	EPI_ISL_428351	GH Nord Essonne Service de Biologie clinique	National Reference Center for Viruses of Respiratory Infections, Institut Pasteur, Paris	2020-03-24	Mélanie Albert et al C
France/IDF3518/2020	EPI_ISL_428352	GH Nord Essonne Service de Biologie clinique	National Reference Center for Viruses of Respiratory Infections, Institut Pasteur, Paris	2020-03-23	Mélanie Albert et al C
France/IDF3577/2020	EPI_ISL_428354	LABM GH nord Essonne de Longjumeau - BP 125	National Reference Center for Viruses of Respiratory Infections, Institut Pasteur, Paris	2020-03-25	Mélanie Albert et al C
France/IDF3703/2020	EPI_ISL_428361	LABM GH nord Essonne de Longjumeau - BP 125	National Reference Center for Viruses of Respiratory Infections, Institut Pasteur, Paris	2020-03-25	Mélanie Albert et al C
France/IDF3709/2020	EPI_ISL_428362	LABM GH nord Essonne de Longjumeau - BP 125	National Reference Center for Viruses of Respiratory Infections, Institut Pasteur, Paris	2020-03-25	Mélanie Albert et al C
France/IDF3745/2020	EPI_ISL_428363	GH Nord Essonne Service de Biologie clinique	National Reference Center for Viruses of Respiratory Infections, Institut Pasteur, Paris	2020-03-26	Mélanie Albert et al C
France/IDF3831/2020	EPI_ISL_428364	Cabinet Médical	National Reference Center for Viruses of Respiratory Infections, Institut Pasteur, Paris	2020-03-23	Mélanie Albert et al C
France/IDF3930/2020	EPI_ISL_428365	LABM GH nord Essonne de Longjumeau - BP 125	National Reference Center for Viruses of Respiratory Infections, Institut Pasteur, Paris	2020-03-26	Mélanie Albert et al C
France/IDF4423/2020	EPI_ISL_443311	Cabinet Médical	National Reference Center for Viruses of Respiratory Infections, Institut Pasteur, Paris	2020-03-23	Mélanie Albert et al C
France/IDF4432/2020	EPI_ISL_443317	Cabinet Médical	National Reference Center for Viruses of Respiratory Infections, Institut Pasteur, Paris	2020-03-23	Mélanie Albert et al C
France/IDF4791/2020	EPI_ISL_443258	Résidence Ornano	National Reference Center for Viruses of Respiratory Infections, Institut Pasteur, Paris	2020-04-02	Mélanie Albert et al C
France/IDF4793/2020	EPI_ISL_443259	Résidence Ornano	National Reference Center for Viruses of Respiratory Infections, Institut Pasteur, Paris	2020-04-02	Mélanie Albert et al C
France/IDF4843/2020	EPI_ISL_443260	LABM GH nord Essonne de Longjumeau - BP 125	National Reference Center for Viruses of Respiratory Infections, Institut Pasteur, Paris	2020-04-02	Mélanie Albert et al C
France/IDF5577/2020	EPI_ISL_443303	Résidence Les Marines	National Reference Center for Viruses of Respiratory Infections, Institut Pasteur, Paris	2020-04-09	Mélanie Albert et al C
France/IDF5650/2020	EPI_ISL_443284	Laboratoire de Microbiologie - Bât A - CH René Dubois	National Reference Center for Viruses of Respiratory Infections, Institut Pasteur, Paris	2020-04-02	Mélanie Albert et al C
France/IDF5655/2020	EPI_ISL_443285	Laboratoire de Microbiologie - Bât A - CH René Dubois	National Reference Center for Viruses of Respiratory Infections, Institut Pasteur, Paris	2020-04-02	Mélanie Albert et al C
France/IDF5657/2020	EPI_ISL_443286	Laboratoire de Microbiologie - Bât A - CH René Dubois	National Reference Center for Viruses of Respiratory Infections, Institut Pasteur, Paris	2020-04-02	Mélanie Albert et al C
France/IDF5662/2020	EPI_ISL_443287	Laboratoire de Microbiologie - Bât A - CH René Dubois	National Reference Center for Viruses of Respiratory Infections, Institut Pasteur, Paris	2020-04-03	Mélanie Albert et al C
France/IDF5666/2020	EPI_ISL_443288	Laboratoire de Microbiologie - Bât A - CH René Dubois	National Reference Center for Viruses of Respiratory Infections, Institut Pasteur, Paris	2020-04-03	Mélanie Albert et al C
France/IDF5778/2020	EPI_ISL_443304	Résidence Esterel	National Reference Center for Viruses of Respiratory Infections, Institut Pasteur, Paris	2020-04-01	Mélanie Albert et al C
France/IDF5847/2020	EPI_ISL_443295	Hôpital Necker - Enfants - Malades Laboratoire de Virologie	National Reference Center for Viruses of Respiratory Infections, Institut Pasteur, Paris	2020-03-19	Mélanie Albert et al C
France/IDF5848/2020	EPI_ISL_443296	Hôpital Necker - Enfants - Malades Laboratoire de Virologie	National Reference Center for Viruses of Respiratory Infections, Institut Pasteur, Paris	2020-03-19	Mélanie Albert et al C
France/IDF5850/2020	EPI_ISL_443297	Hôpital Necker - Enfants - Malades Laboratoire de Virologie	National Reference Center for Viruses of Respiratory Infections, Institut Pasteur, Paris	2020-03-26	Mélanie Albert et al C
France/IDF5851/2020	EPI_ISL_443298	Hôpital Necker - Enfants - Malades Laboratoire de Virologie	National Reference Center for Viruses of Respiratory Infections, Institut Pasteur, Paris	2020-03-25	Mélanie Albert et al C
France/IDF5853/2020	EPI_ISL_443299	Hôpital Necker - Enfants - Malades Laboratoire de Virologie	National Reference Center for Viruses of Respiratory Infections, Institut Pasteur, Paris	2020-03-25	Mélanie Albert et al C
France/IDF5986/2020	EPI_ISL_443305	LABM GH nord Essonne de Longjumeau - BP 125	National Reference Center for Viruses of Respiratory Infections, Institut Pasteur, Paris	2020-04-13	Mélanie Albert et al C
France/IDF6037/2020	EPI_ISL_443306	Cabinet Médical	National Reference Center for Viruses of Respiratory Infections, Institut Pasteur, Paris	2020-04-02	Mélanie Albert et al C
France/IDF6073/2020	EPI_ISL_443314	LABM GH nord Essonne de Longjumeau - BP 125	National Reference Center for Viruses of Respiratory Infections, Institut Pasteur, Paris	2020-04-14	Mélanie Albert et al C
France/Lyon_06042/2020	EPI_ISL_417333	Institut des Agents Infectieux (IAI), Hospices Civils de Lyon	CNR Virus des Infections Respiratoires - France SUD	2020-03-04	Antonin Bal et al
France/Lyon_06056/2020	EPI_ISL_417334	Institut des Agents Infectieux (IAI), Hospices Civils de Lyon	CNR Virus des Infections Respiratoires - France SUD	2020-03-04	Antonin Bal et al
France/Lyon_06464/2020	EPI_ISL_416753	Institut des Agents Infectieux (IAI) Hospices Civils de Lyon	CNR Virus des Infections Respiratoires - France SUD	2020-03-06	Bal et al B
France/Lyon_06487/2020	EPI_ISL_416754	Institut des Agents Infectieux (IAI) Hospices Civils de Lyon	CNR Virus des Infections Respiratoires - France SUD	2020-03-06	Bal et al B
France/Lyon_06531/2020	EPI_ISL_416756	Institut des Agents Infectieux (IAI) Hospices Civils de Lyon	CNR Virus des Infections Respiratoires - France SUD	2020-03-06	Bal et al B
France/Lyon_06625/2020	EPI_ISL_417337	Institut des Agents Infectieux (IAI), Hospices Civils de Lyon	CNR Virus des Infections Respiratoires - France SUD	2020-03-07	Antonin Bal et al
France/Lyon_0668/2020	EPI_ISL_417336	Institut des Agents Infectieux (IAI), Hospices Civils de Lyon	CNR Virus des Infections Respiratoires - France SUD	2020-03-06	Antonin Bal et al
France/Lyon_06820/2020	EPI_ISL_417339	Institut des Agents Infectieux (IAI), Hospices Civils de Lyon	CNR Virus des Infections Respiratoires - France SUD	2020-03-08	Antonin Bal et al
France/Lyon_0693/2020	EPI_ISL_416758	Institut des Agents Infectieux (IAI) Hospices Civils de Lyon	CNR Virus des Infections Respiratoires - France SUD	2020-03-08	Bal et al B
France/Lyon_487/2020	EPI_ISL_416747	Institut des Agents Infectieux (IAI) Hospices Civils de Lyon	CNR Virus des Infections Respiratoires - France SUD	2020-03-04	Bal et al B
France/Lyon_508/2020	EPI_ISL_416748	Institut des Agents Infectieux (IAI) Hospices Civils de Lyon	CNR Virus des Infections Respiratoires - France SUD	2020-03-04	Bal et al B
France/Lyon_683/2020	EPI_ISL_416750	Institut des Agents Infectieux (IAI) Hospices Civils de Lyon	CNR Virus des Infections Respiratoires - France SUD	2020-03-06	Bal et al B
France/Macon_06756/2020	EPI_ISL_417338	Centre Hospitalier de Macon	CNR Virus des Infections Respiratoires - France SUD	2020-03-07	Antonin Bal et al
France/N1620/2020	EPI_ISL_414624	Centre Hositalier Universitaire de Rouen Laboratoire de Virologie	National Reference Center for Viruses of Respiratory Infections, Institut Pasteur, Paris	2020-02-26	Mélnie Albert et al B (https://dx.doi.org/10.1101/2020.04.24.059576)
France/N2223/2020	EPI_ISL_416494	Centre Hositalier Universitaire de Rouen Laboratoire de Virologie	National Reference Center for Viruses of Respiratory Infections, Institut Pasteur, Paris	2020-03-04	Mélnie Albert et al B (https://dx.doi.org/10.1101/2020.04.24.059576)
France/N4427/2020	EPI_ISL_428367	Cabinet Médical	National Reference Center for Viruses of Respiratory Infections, Institut Pasteur, Paris	2020-03-23	Mélanie Albert et al C
France/OCC-11/2020	EPI_ISL_434626	CHU Purpan - Laboratoire de Virologie - Institut Fédératif de Biologie	Laboratoire de virologie - École Nationale Vétérinaire de Toulouse	2020-03-26	Guillaume Croville et al
France/OCC-12/2020	EPI_ISL_434627	CHU Purpan - Laboratoire de Virologie - Institut Fédératif de Biologie	Laboratoire de virologie - École Nationale Vétérinaire de Toulouse	2020-03-26	Guillaume Croville et al
France/OCC-13/2020	EPI_ISL_434628	CHU Purpan - Laboratoire de Virologie - Institut Fédératif de Biologie	Laboratoire de virologie - École Nationale Vétérinaire de Toulouse	2020-04-07	Guillaume Croville et al
France/OCC-14/2020	EPI_ISL_434629	CHU Purpan - Laboratoire de Virologie - Institut Fédératif de Biologie	Laboratoire de virologie - École Nationale Vétérinaire de Toulouse	2020-04-07	Guillaume Croville et al
France/OCC-15/2020	EPI_ISL_434630	CHU Purpan - Laboratoire de Virologie - Institut Fédératif de Biologie	Laboratoire de virologie - École Nationale Vétérinaire de Toulouse	2020-04-08	Guillaume Croville et al
France/OCC-16/2020	EPI_ISL_434631	CHU Purpan - Laboratoire de Virologie - Institut Fédératif de Biologie	Laboratoire de virologie - École Nationale Vétérinaire de Toulouse	2020-04-08	Guillaume Croville et al
France/OCC-17/2020	EPI_ISL_434632	CHU Purpan - Laboratoire de Virologie - Institut Fédératif de Biologie	Laboratoire de virologie - École Nationale Vétérinaire de Toulouse	2020-04-09	Guillaume Croville et al
France/OCC-18/2020	EPI_ISL_434633	CHU Purpan - Laboratoire de Virologie - Institut Fédératif de Biologie	Laboratoire de virologie - École Nationale Vétérinaire de Toulouse	2020-04-09	Guillaume Croville et al
France/OCC-19/2020	EPI_ISL_434634	CHU Purpan - Laboratoire de Virologie - Institut Fédératif de Biologie	Laboratoire de virologie - École Nationale Vétérinaire de Toulouse	2020-04-11	Guillaume Croville et al
France/OCC-20/2020	EPI_ISL_434635	CHU Purpan - Laboratoire de Virologie - Institut Fédératif de Biologie	Laboratoire de virologie - École Nationale Vétérinaire de Toulouse	2020-04-11	Guillaume Croville et al
France/PL1643/2020	EPI_ISL_414625	Centre Hospitalier Régional Universitaire de Nantes Laboratoire de Virologie	National Reference Center for Viruses of Respiratory Infections, Institut Pasteur, Paris	2020-02-26	Mélnie Albert et al B (https://dx.doi.org/10.1101/2020.04.24.059576)
France/Pollionay_1733/2020	EPI_ISL_416745	CNR Virus des Infections Respiratoires - France SUD	CNR Virus des Infections Respiratoires - France SUD	2020-03-10	Bal et al B
France/Valence_425/2020	EPI_ISL_416746	CNR Virus des Infections Respiratoires - France SUD	CNR Virus des Infections Respiratoires - France SUD	2020-03-03	Bal et al B
France/Valence_532/2020	EPI_ISL_416749	Centre Hospitalier de Valence	CNR Virus des Infections Respiratoires - France SUD	2020-03-04	Bal et al B
Germany/Baden-Wuerttemberg-1/2020	EPI_ISL_412912	State Health Office Baden-Wuerttemberg	Charité Universitätsmedizin Berlin, Institute of Virology	2020-02-25	Victor M Corman et al (https://dx.doi.org/10.2807/1560-7917.ES.2020.25.13.2000305)
Germany/BAV-MVP0002/2020	EPI_ISL_437204	Max von Pettenkofer Institute, Virology, National Reference Center for Retroviruses, LMU München	Laboratory for Functional Genome Analysis, Dept. Genomics, Gene Center of the LMU Munich	2020-03-05	Max Muenchhoff et al
Germany/BAV-MVP0003/2020	EPI_ISL_437205	Max von Pettenkofer Institute, Virology, National Reference Center for Retroviruses, LMU München	Laboratory for Functional Genome Analysis, Dept. Genomics, Gene Center of the LMU Munich	2020-03-16	Max Muenchhoff et al
Germany/BAV-MVP0005/2020	EPI_ISL_437206	Max von Pettenkofer Institute, Virology, National Reference Center for Retroviruses, LMU München	Laboratory for Functional Genome Analysis, Dept. Genomics, Gene Center of the LMU Munich	2020-03-18	Max Muenchhoff et al
Germany/BAV-MVP0006/2020	EPI_ISL_437207	Max von Pettenkofer Institute, Virology, National Reference Center for Retroviruses, LMU München	Laboratory for Functional Genome Analysis, Dept. Genomics, Gene Center of the LMU Munich	2020-04-09	Max Muenchhoff et al
Germany/BAV-MVP0007/2020	EPI_ISL_437208	Max von Pettenkofer Institute, Virology, National Reference Center for Retroviruses, LMU München	Laboratory for Functional Genome Analysis, Dept. Genomics, Gene Center of the LMU Munich	2020-04-11	Max Muenchhoff et al
Germany/BAV-MVP0008/2020	EPI_ISL_437209	Max von Pettenkofer Institute, Virology, National Reference Center for Retroviruses, LMU München	Laboratory for Functional Genome Analysis, Dept. Genomics, Gene Center of the LMU Munich	2020-04-13	Max Muenchhoff et al
Germany/BAV-MVP0009/2020	EPI_ISL_437210	Max von Pettenkofer Institute, Virology, National Reference Center for Retroviruses, LMU München	Laboratory for Functional Genome Analysis, Dept. Genomics, Gene Center of the LMU Munich	2020-03-07	Max Muenchhoff et al
Germany/BAV-MVP0010/2020	EPI_ISL_437211	Max von Pettenkofer Institute, Virology, National Reference Center for Retroviruses, LMU München	Laboratory for Functional Genome Analysis, Dept. Genomics, Gene Center of the LMU Munich	2020-03-12	Max Muenchhoff et al
Germany/BAV-MVP0011/2020	EPI_ISL_437212	Max von Pettenkofer Institute, Virology, National Reference Center for Retroviruses, LMU München	Laboratory for Functional Genome Analysis, Dept. Genomics, Gene Center of the LMU Munich	2020-03-22	Max Muenchhoff et al
Germany/BAV-MVP0012/2020	EPI_ISL_437213	Max von Pettenkofer Institute, Virology, National Reference Center for Retroviruses, LMU München	Laboratory for Functional Genome Analysis, Dept. Genomics, Gene Center of the LMU Munich	2020-04-05	Max Muenchhoff et al
Germany/BAV-MVP0013/2020	EPI_ISL_437214	Max von Pettenkofer Institute, Virology, National Reference Center for Retroviruses, LMU München	Laboratory for Functional Genome Analysis, Dept. Genomics, Gene Center of the LMU Munich	2020-04-07	Max Muenchhoff et al
Germany/BAV-MVP0014/2020	EPI_ISL_437215	Max von Pettenkofer Institute, Virology, National Reference Center for Retroviruses, LMU München	Laboratory for Functional Genome Analysis, Dept. Genomics, Gene Center of the LMU Munich	2020-04-08	Max Muenchhoff et al
Germany/BAV-MVP0015/2020	EPI_ISL_437216	Max von Pettenkofer Institute, Virology, National Reference Center for Retroviruses, LMU München	Laboratory for Functional Genome Analysis, Dept. Genomics, Gene Center of the LMU Munich	2020-04-09	Max Muenchhoff et al
Germany/BAV-MVP0016/2020	EPI_ISL_437217	Max von Pettenkofer Institute, Virology, National Reference Center for Retroviruses, LMU München	Laboratory for Functional Genome Analysis, Dept. Genomics, Gene Center of the LMU Munich	2020-04-11	Max Muenchhoff et al
Germany/BAV-MVP0017/2020	EPI_ISL_437218	Max von Pettenkofer Institute, Virology, National Reference Center for Retroviruses, LMU München	Laboratory for Functional Genome Analysis, Dept. Genomics, Gene Center of the LMU Munich	2020-03-12	Max Muenchhoff et al
Germany/BAV-MVP0019/2020	EPI_ISL_437219	Max von Pettenkofer Institute, Virology, National Reference Center for Retroviruses, LMU München	Laboratory for Functional Genome Analysis, Dept. Genomics, Gene Center of the LMU Munich	2020-04-02	Max Muenchhoff et al
Germany/BAV-MVP0020/2020	EPI_ISL_437220	Max von Pettenkofer Institute, Virology, National Reference Center for Retroviruses, LMU München	Laboratory for Functional Genome Analysis, Dept. Genomics, Gene Center of the LMU Munich	2020-04-06	Max Muenchhoff et al
Germany/BAV-MVP0021/2020	EPI_ISL_437221	Max von Pettenkofer Institute, Virology, National Reference Center for Retroviruses, LMU München	Laboratory for Functional Genome Analysis, Dept. Genomics, Gene Center of the LMU Munich	2020-04-06	Max Muenchhoff et al
Germany/BAV-MVP0022/2020	EPI_ISL_437222	Max von Pettenkofer Institute, Virology, National Reference Center for Retroviruses, LMU München	Laboratory for Functional Genome Analysis, Dept. Genomics, Gene Center of the LMU Munich	2020-04-11	Max Muenchhoff et al
Germany/BAV-MVP0023/2020	EPI_ISL_437223	Max von Pettenkofer Institute, Virology, National Reference Center for Retroviruses, LMU München	Laboratory for Functional Genome Analysis, Dept. Genomics, Gene Center of the LMU Munich	2020-03-12	Max Muenchhoff et al
Germany/BAV-MVP0025/2020	EPI_ISL_437224	Max von Pettenkofer Institute, Virology, National Reference Center for Retroviruses, LMU München	Laboratory for Functional Genome Analysis, Dept. Genomics, Gene Center of the LMU Munich	2020-03-29	Max Muenchhoff et al
Germany/BAV-MVP0026/2020	EPI_ISL_437225	Max von Pettenkofer Institute, Virology, National Reference Center for Retroviruses, LMU München	Laboratory for Functional Genome Analysis, Dept. Genomics, Gene Center of the LMU Munich	2020-04-01	Max Muenchhoff et al
Germany/BAV-MVP0027/2020	EPI_ISL_437226	Max von Pettenkofer Institute, Virology, National Reference Center for Retroviruses, LMU München	Laboratory for Functional Genome Analysis, Dept. Genomics, Gene Center of the LMU Munich	2020-04-10	Max Muenchhoff et al
Germany/BAV-MVP0028/2020	EPI_ISL_437227	Max von Pettenkofer Institute, Virology, National Reference Center for Retroviruses, LMU München	Laboratory for Functional Genome Analysis, Dept. Genomics, Gene Center of the LMU Munich	2020-04-13	Max Muenchhoff et al
Germany/BAV-MVP0029/2020	EPI_ISL_437228	Max von Pettenkofer Institute, Virology, National Reference Center for Retroviruses, LMU München	Laboratory for Functional Genome Analysis, Dept. Genomics, Gene Center of the LMU Munich	2020-03-11	Max Muenchhoff et al
Germany/BAV-MVP0030/2020	EPI_ISL_437229	Max von Pettenkofer Institute, Virology, National Reference Center for Retroviruses, LMU München	Laboratory for Functional Genome Analysis, Dept. Genomics, Gene Center of the LMU Munich	2020-03-14	Max Muenchhoff et al
Germany/BAV-MVP0031/2020	EPI_ISL_437230	Max von Pettenkofer Institute, Virology, National Reference Center for Retroviruses, LMU München	Laboratory for Functional Genome Analysis, Dept. Genomics, Gene Center of the LMU Munich	2020-03-22	Max Muenchhoff et al
Germany/BAV-MVP0032/2020	EPI_ISL_437231	Max von Pettenkofer Institute, Virology, National Reference Center for Retroviruses, LMU München	Laboratory for Functional Genome Analysis, Dept. Genomics, Gene Center of the LMU Munich	2020-03-25	Max Muenchhoff et al
Germany/BAV-MVP0033/2020	EPI_ISL_437232	Max von Pettenkofer Institute, Virology, National Reference Center for Retroviruses, LMU München	Laboratory for Functional Genome Analysis, Dept. Genomics, Gene Center of the LMU Munich	2020-03-31	Max Muenchhoff et al
Germany/BAV-MVP0034/2020	EPI_ISL_437233	Max von Pettenkofer Institute, Virology, National Reference Center for Retroviruses, LMU München	Laboratory for Functional Genome Analysis, Dept. Genomics, Gene Center of the LMU Munich	2020-03-13	Max Muenchhoff et al
Germany/BAV-MVP0035/2020	EPI_ISL_437234	Max von Pettenkofer Institute, Virology, National Reference Center for Retroviruses, LMU München	Laboratory for Functional Genome Analysis, Dept. Genomics, Gene Center of the LMU Munich	2020-03-15	Max Muenchhoff et al
Germany/BAV-MVP0036/2020	EPI_ISL_437235	Max von Pettenkofer Institute, Virology, National Reference Center for Retroviruses, LMU München	Laboratory for Functional Genome Analysis, Dept. Genomics, Gene Center of the LMU Munich	2020-03-21	Max Muenchhoff et al
Germany/BAV-MVP0037/2020	EPI_ISL_437236	Max von Pettenkofer Institute, Virology, National Reference Center for Retroviruses, LMU München	Laboratory for Functional Genome Analysis, Dept. Genomics, Gene Center of the LMU Munich	2020-03-22	Max Muenchhoff et al
Germany/BAV-MVP0038/2020	EPI_ISL_437237	Max von Pettenkofer Institute, Virology, National Reference Center for Retroviruses, LMU München	Laboratory for Functional Genome Analysis, Dept. Genomics, Gene Center of the LMU Munich	2020-03-23	Max Muenchhoff et al
Germany/BAV-MVP0039/2020	EPI_ISL_437238	Max von Pettenkofer Institute, Virology, National Reference Center for Retroviruses, LMU München	Laboratory for Functional Genome Analysis, Dept. Genomics, Gene Center of the LMU Munich	2020-03-24	Max Muenchhoff et al
Germany/BAV-MVP0040/2020	EPI_ISL_437239	Max von Pettenkofer Institute, Virology, National Reference Center for Retroviruses, LMU München	Laboratory for Functional Genome Analysis, Dept. Genomics, Gene Center of the LMU Munich	2020-04-02	Max Muenchhoff et al
Germany/BAV-MVP0041/2020	EPI_ISL_437240	Max von Pettenkofer Institute, Virology, National Reference Center for Retroviruses, LMU München	Laboratory for Functional Genome Analysis, Dept. Genomics, Gene Center of the LMU Munich	2020-04-06	Max Muenchhoff et al
Germany/BAV-MVP0042/2020	EPI_ISL_437241	Max von Pettenkofer Institute, Virology, National Reference Center for Retroviruses, LMU München	Laboratory for Functional Genome Analysis, Dept. Genomics, Gene Center of the LMU Munich	2020-04-07	Max Muenchhoff et al

Germany/BAV-MVP0043/2020	EPI_ISL_437242	Max von Pettenkofer Institute, Virology, National Reference Center for Retroviruses, LMU München	Laboratory for Functional Genome Analysis, Dept. Genomics, Gene Center of the LMU Munich	2020-04-09	Max Muenchhoff et al
Germany/BAV-MVP0044/2020	EPI_ISL_437243	Max von Pettenkofer Institute, Virology, National Reference Center for Retroviruses, LMU München	Laboratory for Functional Genome Analysis, Dept. Genomics, Gene Center of the LMU Munich	2020-04-11	Max Muenchhoff et al
Germany/BAV-MVP0045/2020	EPI_ISL_437244	Max von Pettenkofer Institute, Virology, National Reference Center for Retroviruses, LMU München	Laboratory for Functional Genome Analysis, Dept. Genomics, Gene Center of the LMU Munich	2020-03-08	Max Muenchhoff et al
Germany/BAV-MVP0047/2020	EPI_ISL_437246	Max von Pettenkofer Institute, Virology, National Reference Center for Retroviruses, LMU München	Laboratory for Functional Genome Analysis, Dept. Genomics, Gene Center of the LMU Munich	2020-03-11	Max Muenchhoff et al
Germany/BAV-MVP0048/2020	EPI_ISL_437247	Max von Pettenkofer Institute, Virology, National Reference Center for Retroviruses, LMU München	Laboratory for Functional Genome Analysis, Dept. Genomics, Gene Center of the LMU Munich	2020-03-11	Max Muenchhoff et al
Germany/BAV-MVP0049/2020	EPI_ISL_437248	Max von Pettenkofer Institute, Virology, National Reference Center for Retroviruses, LMU München	Laboratory for Functional Genome Analysis, Dept. Genomics, Gene Center of the LMU Munich	2020-03-11	Max Muenchhoff et al
Germany/BAV-MVP0050/2020	EPI_ISL_437249	Max von Pettenkofer Institute, Virology, National Reference Center for Retroviruses, LMU München	Laboratory for Functional Genome Analysis, Dept. Genomics, Gene Center of the LMU Munich	2020-03-12	Max Muenchhoff et al
Germany/BAV-MVP0051/2020	EPI_ISL_437250	Max von Pettenkofer Institute, Virology, National Reference Center for Retroviruses, LMU München	Laboratory for Functional Genome Analysis, Dept. Genomics, Gene Center of the LMU Munich	2020-03-12	Max Muenchhoff et al
Germany/BAV-MVP0052/2020	EPI_ISL_437251	Max von Pettenkofer Institute, Virology, National Reference Center for Retroviruses, LMU München	Laboratory for Functional Genome Analysis, Dept. Genomics, Gene Center of the LMU Munich	2020-03-14	Max Muenchhoff et al
Germany/BAV-MVP0053/2020	EPI_ISL_437252	Max von Pettenkofer Institute, Virology, National Reference Center for Retroviruses, LMU München	Laboratory for Functional Genome Analysis, Dept. Genomics, Gene Center of the LMU Munich	2020-03-16	Max Muenchhoff et al
Germany/BAV-MVP0054/2020	EPI_ISL_437253	Max von Pettenkofer Institute, Virology, National Reference Center for Retroviruses, LMU München	Laboratory for Functional Genome Analysis, Dept. Genomics, Gene Center of the LMU Munich	2020-03-17	Max Muenchhoff et al
Germany/BAV-MVP0055/2020	EPI_ISL_437254	Max von Pettenkofer Institute, Virology, National Reference Center for Retroviruses, LMU München	Laboratory for Functional Genome Analysis, Dept. Genomics, Gene Center of the LMU Munich	2020-03-17	Max Muenchhoff et al
Germany/BAV-MVP0056/2020	EPI_ISL_437255	Max von Pettenkofer Institute, Virology, National Reference Center for Retroviruses, LMU München	Laboratory for Functional Genome Analysis, Dept. Genomics, Gene Center of the LMU Munich	2020-03-19	Max Muenchhoff et al
Germany/BAV-MVP0057/2020	EPI_ISL_437256	Max von Pettenkofer Institute, Virology, National Reference Center for Retroviruses, LMU München	Laboratory for Functional Genome Analysis, Dept. Genomics, Gene Center of the LMU Munich	2020-03-20	Max Muenchhoff et al
Germany/BAV-MVP0058/2020	EPI_ISL_437257	Max von Pettenkofer Institute, Virology, National Reference Center for Retroviruses, LMU München	Laboratory for Functional Genome Analysis, Dept. Genomics, Gene Center of the LMU Munich	2020-03-20	Max Muenchhoff et al
Germany/BAV-MVP0059/2020	EPI_ISL_437258	Max von Pettenkofer Institute, Virology, National Reference Center for Retroviruses, LMU München	Laboratory for Functional Genome Analysis, Dept. Genomics, Gene Center of the LMU Munich	2020-03-20	Max Muenchhoff et al
Germany/BAV-MVP0060/2020	EPI_ISL_437259	Max von Pettenkofer Institute, Virology, National Reference Center for Retroviruses, LMU München	Laboratory for Functional Genome Analysis, Dept. Genomics, Gene Center of the LMU Munich	2020-03-21	Max Muenchhoff et al
Germany/BAV-MVP0061/2020	EPI_ISL_437260	Max von Pettenkofer Institute, Virology, National Reference Center for Retroviruses, LMU München	Laboratory for Functional Genome Analysis, Dept. Genomics, Gene Center of the LMU Munich	2020-03-22	Max Muenchhoff et al
Germany/BAV-MVP0062/2020	EPI_ISL_437261	Max von Pettenkofer Institute, Virology, National Reference Center for Retroviruses, LMU München	Laboratory for Functional Genome Analysis, Dept. Genomics, Gene Center of the LMU Munich	2020-03-22	Max Muenchhoff et al
Germany/BAV-MVP0063/2020	EPI_ISL_437262	Max von Pettenkofer Institute, Virology, National Reference Center for Retroviruses, LMU München	Laboratory for Functional Genome Analysis, Dept. Genomics, Gene Center of the LMU Munich	2020-03-22	Max Muenchhoff et al
Germany/BAV-MVP0064/2020	EPI_ISL_437263	Max von Pettenkofer Institute, Virology, National Reference Center for Retroviruses, LMU München	Laboratory for Functional Genome Analysis, Dept. Genomics, Gene Center of the LMU Munich	2020-03-24	Max Muenchhoff et al
Germany/BAV-MVP0067/2020	EPI_ISL_437264	Max von Pettenkofer Institute, Virology, National Reference Center for Retroviruses, LMU München	Laboratory for Functional Genome Analysis, Dept. Genomics, Gene Center of the LMU Munich	2020-03-25	Max Muenchhoff et al
Germany/BAV-MVP0069/2020	EPI_ISL_437265	Max von Pettenkofer Institute, Virology, National Reference Center for Retroviruses, LMU München	Laboratory for Functional Genome Analysis, Dept. Genomics, Gene Center of the LMU Munich	2020-03-25	Max Muenchhoff et al
Germany/BAV-MVP0071/2020	EPI_ISL_437267	Max von Pettenkofer Institute, Virology, National Reference Center for Retroviruses, LMU München	Laboratory for Functional Genome Analysis, Dept. Genomics, Gene Center of the LMU Munich	2020-03-27	Max Muenchhoff et al
Germany/BAV-MVP0072/2020	EPI_ISL_437268	Max von Pettenkofer Institute, Virology, National Reference Center for Retroviruses, LMU München	Laboratory for Functional Genome Analysis, Dept. Genomics, Gene Center of the LMU Munich	2020-03-28	Max Muenchhoff et al
Germany/BAV-MVP0073/2020	EPI_ISL_437269	Max von Pettenkofer Institute, Virology, National Reference Center for Retroviruses, LMU München	Laboratory for Functional Genome Analysis, Dept. Genomics, Gene Center of the LMU Munich	2020-03-28	Max Muenchhoff et al
Germany/BAV-MVP0075/2020	EPI_ISL_437270	Max von Pettenkofer Institute, Virology, National Reference Center for Retroviruses, LMU München	Laboratory for Functional Genome Analysis, Dept. Genomics, Gene Center of the LMU Munich	2020-03-29	Max Muenchhoff et al
Germany/BAV-MVP0076/2020	EPI_ISL_437271	Max von Pettenkofer Institute, Virology, National Reference Center for Retroviruses, LMU München	Laboratory for Functional Genome Analysis, Dept. Genomics, Gene Center of the LMU Munich	2020-03-30	Max Muenchhoff et al
Germany/BAV-MVP0077/2020	EPI_ISL_437272	Max von Pettenkofer Institute, Virology, National Reference Center for Retroviruses, LMU München	Laboratory for Functional Genome Analysis, Dept. Genomics, Gene Center of the LMU Munich	2020-03-30	Max Muenchhoff et al
Germany/BAV-MVP0082/2020	EPI_ISL_437276	Max von Pettenkofer Institute, Virology, National Reference Center for Retroviruses, LMU München	Laboratory for Functional Genome Analysis, Dept. Genomics, Gene Center of the LMU Munich	2020-03-30	Max Muenchhoff et al
Germany/BAV-MVP0083/2020	EPI_ISL_437277	Max von Pettenkofer Institute, Virology, National Reference Center for Retroviruses, LMU München	Laboratory for Functional Genome Analysis, Dept. Genomics, Gene Center of the LMU Munich	2020-03-31	Max Muenchhoff et al
Germany/BAV-MVP0084/2020	EPI_ISL_437278	Max von Pettenkofer Institute, Virology, National Reference Center for Retroviruses, LMU München	Laboratory for Functional Genome Analysis, Dept. Genomics, Gene Center of the LMU Munich	2020-04-01	Max Muenchhoff et al
Germany/BAV-MVP0085/2020	EPI_ISL_437279	Max von Pettenkofer Institute, Virology, National Reference Center for Retroviruses, LMU München	Laboratory for Functional Genome Analysis, Dept. Genomics, Gene Center of the LMU Munich	2020-04-01	Max Muenchhoff et al
Germany/BAV-MVP0086/2020	EPI_ISL_437280	Max von Pettenkofer Institute, Virology, National Reference Center for Retroviruses, LMU München	Laboratory for Functional Genome Analysis, Dept. Genomics, Gene Center of the LMU Munich	2020-04-01	Max Muenchhoff et al
Germany/BAV-MVP0087/2020	EPI_ISL_437281	Max von Pettenkofer Institute, Virology, National Reference Center for Retroviruses, LMU München	Laboratory for Functional Genome Analysis, Dept. Genomics, Gene Center of the LMU Munich	2020-04-01	Max Muenchhoff et al
Germany/BAV-MVP0088/2020	EPI_ISL_437282	Max von Pettenkofer Institute, Virology, National Reference Center for Retroviruses, LMU München	Laboratory for Functional Genome Analysis, Dept. Genomics, Gene Center of the LMU Munich	2020-04-01	Max Muenchhoff et al
Germany/BAV-MVP0089/2020	EPI_ISL_437283	Max von Pettenkofer Institute, Virology, National Reference Center for Retroviruses, LMU München	Laboratory for Functional Genome Analysis, Dept. Genomics, Gene Center of the LMU Munich	2020-04-02	Max Muenchhoff et al
Germany/BAV-MVP0090/2020	EPI_ISL_437284	Max von Pettenkofer Institute, Virology, National Reference Center for Retroviruses, LMU München	Laboratory for Functional Genome Analysis, Dept. Genomics, Gene Center of the LMU Munich	2020-04-02	Max Muenchhoff et al
Germany/BAV-MVP0091/2020	EPI_ISL_437285	Max von Pettenkofer Institute, Virology, National Reference Center for Retroviruses, LMU München	Laboratory for Functional Genome Analysis, Dept. Genomics, Gene Center of the LMU Munich	2020-04-02	Max Muenchhoff et al
Germany/BAV-MVP0092/2020	EPI_ISL_437286	Max von Pettenkofer Institute, Virology, National Reference Center for Retroviruses, LMU München	Laboratory for Functional Genome Analysis, Dept. Genomics, Gene Center of the LMU Munich	2020-04-02	Max Muenchhoff et al
Germany/BAV-MVP0095/2020	EPI_ISL_437287	Max von Pettenkofer Institute, Virology, National Reference Center for Retroviruses, LMU München	Laboratory for Functional Genome Analysis, Dept. Genomics, Gene Center of the LMU Munich	2020-04-03	Max Muenchhoff et al
Germany/BAV-MVP0096/2020	EPI_ISL_437288	Max von Pettenkofer Institute, Virology, National Reference Center for Retroviruses, LMU München	Laboratory for Functional Genome Analysis, Dept. Genomics, Gene Center of the LMU Munich	2020-04-03	Max Muenchhoff et al
Germany/BAV-MVP0097/2020	EPI_ISL_437289	Max von Pettenkofer Institute, Virology, National Reference Center for Retroviruses, LMU München	Laboratory for Functional Genome Analysis, Dept. Genomics, Gene Center of the LMU Munich	2020-04-03	Max Muenchhoff et al
Germany/BAV-MVP0098/2020	EPI_ISL_452104	Max von Pettenkofer Institute, Virology, National Reference Center for Retroviruses, LMU München	Laboratory for Functional Genome Analysis, Dept. Genomics, Gene Center of the LMU Munich	2020-04-04	Max Muenchhoff et al
Germany/BAV-MVP0099/2020	EPI_ISL_437290	Max von Pettenkofer Institute, Virology, National Reference Center for Retroviruses, LMU München	Laboratory for Functional Genome Analysis, Dept. Genomics, Gene Center of the LMU Munich	2020-04-05	Max Muenchhoff et al
Germany/BAV-MVP0100/2020	EPI_ISL_437291	Max von Pettenkofer Institute, Virology, National Reference Center for Retroviruses, LMU München	Laboratory for Functional Genome Analysis, Dept. Genomics, Gene Center of the LMU Munich	2020-04-06	Max Muenchhoff et al
Germany/BAV-MVP0101/2020	EPI_ISL_437292	Max von Pettenkofer Institute, Virology, National Reference Center for Retroviruses, LMU München	Laboratory for Functional Genome Analysis, Dept. Genomics, Gene Center of the LMU Munich	2020-04-07	Max Muenchhoff et al
Germany/BAV-MVP0103/2020	EPI_ISL_437293	Max von Pettenkofer Institute, Virology, National Reference Center for Retroviruses, LMU München	Laboratory for Functional Genome Analysis, Dept. Genomics, Gene Center of the LMU Munich	2020-04-09	Max Muenchhoff et al
Germany/BAV-MVP0105/2020	EPI_ISL_437295	Max von Pettenkofer Institute, Virology, National Reference Center for Retroviruses, LMU München	Laboratory for Functional Genome Analysis, Dept. Genomics, Gene Center of the LMU Munich	2020-04-12	Max Muenchhoff et al
Germany/BAV-MVP0106/2020	EPI_ISL_437296	Max von Pettenkofer Institute, Virology, National Reference Center for Retroviruses, LMU München	Laboratory for Functional Genome Analysis, Dept. Genomics, Gene Center of the LMU Munich	2020-04-13	Max Muenchhoff et al
Germany/BAV-MVP0107/2020	EPI_ISL_437297	Max von Pettenkofer Institute, Virology, National Reference Center for Retroviruses, LMU München	Laboratory for Functional Genome Analysis, Dept. Genomics, Gene Center of the LMU Munich	2020-04-13	Max Muenchhoff et al
Germany/BAV-MVP0108/2020				2020-04-13	
Germany/BAV-MVP0109/2020				2020-03-11	
Germany/BAV-MVP0111/2020				2020-03-14	
Germany/BAV-MVP0113/2020	EPI_ISL_451937	Max von Pettenkofer Institute, Virology, National Reference Center for Retroviruses, LMU München	Laboratory for Functional Genome Analysis, Dept. Genomics, Gene Center of the LMU Munich	2020-03-17	Max Muenchhoff et al
Germany/BAV-MVP0119/2020	EPI_ISL_451939	Max von Pettenkofer Institute, Virology, National Reference Center for Retroviruses, LMU München	Laboratory for Functional Genome Analysis, Dept. Genomics, Gene Center of the LMU Munich	2020-03-20	Max Muenchhoff et al
Germany/BAV-MVP0123/2020	EPI_ISL_451940	Max von Pettenkofer Institute, Virology, National Reference Center for Retroviruses, LMU München	Laboratory for Functional Genome Analysis, Dept. Genomics, Gene Center of the LMU Munich	2020-03-23	Max Muenchhoff et al
Germany/BAV-MVP0124/2020	EPI_ISL_451941	Max von Pettenkofer Institute, Virology, National Reference Center for Retroviruses, LMU München	Laboratory for Functional Genome Analysis, Dept. Genomics, Gene Center of the LMU Munich	2020-03-23	Max Muenchhoff et al
Germany/BAV-MVP0149/2020	EPI_ISL_451942	Max von Pettenkofer Institute, Virology, National Reference Center for Retroviruses, LMU München	Laboratory for Functional Genome Analysis, Dept. Genomics, Gene Center of the LMU Munich	2020-03-30	Max Muenchhoff et al
Germany/BAV-MVP0151/2020	EPI_ISL_451943	Max von Pettenkofer Institute, Virology, National Reference Center for Retroviruses, LMU München	Laboratory for Functional Genome Analysis, Dept. Genomics, Gene Center of the LMU Munich	2020-03-30	Max Muenchhoff et al
Germany/BAV-MVP0153/2020	EPI_ISL_451944	Max von Pettenkofer Institute, Virology, National Reference Center for Retroviruses, LMU München	Laboratory for Functional Genome Analysis, Dept. Genomics, Gene Center of the LMU Munich	2020-03-31	Max Muenchhoff et al
Germany/BAV-MVP0155/2020	EPI_ISL_451945	Max von Pettenkofer Institute, Virology, National Reference Center for Retroviruses, LMU München	Laboratory for Functional Genome Analysis, Dept. Genomics, Gene Center of the LMU Munich	2020-04-01	Max Muenchhoff et al
Germany/BAV-MVP0157/2020	EPI_ISL_451946	Max von Pettenkofer Institute, Virology, National Reference Center for Retroviruses, LMU München	Laboratory for Functional Genome Analysis, Dept. Genomics, Gene Center of the LMU Munich	2020-04-02	Max Muenchhoff et al
Germany/BAV-MVP0173/2020	EPI_ISL_451947	Max von Pettenkofer Institute, Virology, National Reference Center for Retroviruses, LMU München	Laboratory for Functional Genome Analysis, Dept. Genomics, Gene Center of the LMU Munich	2020-04-08	Max Muenchhoff et al
Germany/BAV-MVP0179/2020				2020-04-12	
Germany/BAV-MVP0182/2020	EPI_ISL_466875	Max von Pettenkofer Institute, Virology, National Reference Center for Retroviruses, LMU München	Laboratory for Functional Genome Analysis, Dept. Genomics, Gene Center of the LMU Munich	2020-04-13	Max Muenchhoff et al
Germany/BAV-MVP0183/2020	EPI_ISL_466876	Max von Pettenkofer Institute, Virology, National Reference Center for Retroviruses, LMU München	Laboratory for Functional Genome Analysis, Dept. Genomics, Gene Center of the LMU Munich	2020-04-13	Max Muenchhoff et al
Germany/BAV-MVP0184/2020	EPI_ISL_466877	Max von Pettenkofer Institute, Virology, National Reference Center for Retroviruses, LMU München	Laboratory for Functional Genome Analysis, Dept. Genomics, Gene Center of the LMU Munich	2020-04-13	Max Muenchhoff et al
Germany/BAV-MVP0185/2020	EPI_ISL_466878	Max von Pettenkofer Institute, Virology, National Reference Center for Retroviruses, LMU München	Laboratory for Functional Genome Analysis, Dept. Genomics, Gene Center of the LMU Munich	2020-04-13	Max Muenchhoff et al
Germany/BAV-MVP0186/2020	EPI_ISL_466879	Max von Pettenkofer Institute, Virology, National Reference Center for Retroviruses, LMU München	Laboratory for Functional Genome Analysis, Dept. Genomics, Gene Center of the LMU Munich	2020-04-13	Max Muenchhoff et al
Germany/BAV-MVP0187/2020	EPI_ISL_466880	Max von Pettenkofer Institute, Virology, National Reference Center for Retroviruses, LMU München	Laboratory for Functional Genome Analysis, Dept. Genomics, Gene Center of the LMU Munich	2020-04-13	Max Muenchhoff et al
Germany/BAV-MVP0188/2020				2020-04-14	
Germany/BAV-MVP0190/2020	EPI_ISL_466881	Max von Pettenkofer Institute, Virology, National Reference Center for Retroviruses, LMU München	Laboratory for Functional Genome Analysis, Dept. Genomics, Gene Center of the LMU Munich	2020-04-14	Max Muenchhoff et al
Germany/BAV-MVP0192/2020	EPI_ISL_466882	Max von Pettenkofer Institute, Virology, National Reference Center for Retroviruses, LMU München	Laboratory for Functional Genome Analysis, Dept. Genomics, Gene Center of the LMU Munich	2020-04-15	Max Muenchhoff et al
Germany/BAV-MVP0193/2020	EPI_ISL_466883	Max von Pettenkofer Institute, Virology, National Reference Center for Retroviruses, LMU München	Laboratory for Functional Genome Analysis, Dept. Genomics, Gene Center of the LMU Munich	2020-04-15	Max Muenchhoff et al
Germany/BAV-MVP0196/2020	EPI_ISL_466884	Max von Pettenkofer Institute, Virology, National Reference Center for Retroviruses, LMU München	Laboratory for Functional Genome Analysis, Dept. Genomics, Gene Center of the LMU Munich	2020-04-16	Max Muenchhoff et al
Germany/BAV-MVP0200/2020	EPI_ISL_466885	Max von Pettenkofer Institute, Virology, National Reference Center for Retroviruses, LMU München	Laboratory for Functional Genome Analysis, Dept. Genomics, Gene Center of the LMU Munich	2020-04-16	Max Muenchhoff et al
Germany/BAV-MVP0201/2020	EPI_ISL_466886	Max von Pettenkofer Institute, Virology, National Reference Center for Retroviruses, LMU München	Laboratory for Functional Genome Analysis, Dept. Genomics, Gene Center of the LMU Munich	2020-04-17	Max Muenchhoff et al
Germany/BAV-MVP0203/2020	EPI_ISL_466887	Max von Pettenkofer Institute, Virology, National Reference Center for Retroviruses, LMU München	Laboratory for Functional Genome Analysis, Dept. Genomics, Gene Center of the LMU Munich	2020-04-17	Max Muenchhoff et al
Germany/BAV-MVP0204/2020				2020-04-17	
Germany/BAV-MVP0207/2020	EPI_ISL_466888	Max von Pettenkofer Institute, Virology, National Reference Center for Retroviruses, LMU München	Laboratory for Functional Genome Analysis, Dept. Genomics, Gene Center of the LMU Munich	2020-04-20	Max Muenchhoff et al
Germany/BAV-MVP0208/2020	EPI_ISL_466889	Max von Pettenkofer Institute, Virology, National Reference Center for Retroviruses, LMU München	Laboratory for Functional Genome Analysis, Dept. Genomics, Gene Center of the LMU Munich	2020-04-19	Max Muenchhoff et al
Germany/BAV-MVP0210/2020	EPI_ISL_466890	Max von Pettenkofer Institute, Virology, National Reference Center for Retroviruses, LMU München	Laboratory for Functional Genome Analysis, Dept. Genomics, Gene Center of the LMU Munich	2020-04-20	Max Muenchhoff et al
Germany/BAV-MVP0214/2020				2020-04-21	
Germany/BAV-MVP0218/2020	EPI_ISL_466891	Max von Pettenkofer Institute, Virology, National Reference Center for Retroviruses, LMU München	Laboratory for Functional Genome Analysis, Dept. Genomics, Gene Center of the LMU Munich	2020-04-22	Max Muenchhoff et al
Germany/BAV-MVP0219/2020	EPI_ISL_466892	Max von Pettenkofer Institute, Virology, National Reference Center for Retroviruses, LMU München	Laboratory for Functional Genome Analysis, Dept. Genomics, Gene Center of the LMU Munich	2020-04-22	Max Muenchhoff et al
Germany/BAV-MVP0220/2020	EPI_ISL_466893	Max von Pettenkofer Institute, Virology, National Reference Center for Retroviruses, LMU München	Laboratory for Functional Genome Analysis, Dept. Genomics, Gene Center of the LMU Munich	2020-04-22	Max Muenchhoff et al
Germany/BAV-MVP0221/2020	EPI_ISL_466894	Max von Pettenkofer Institute, Virology, National Reference Center for Retroviruses, LMU München	Laboratory for Functional Genome Analysis, Dept. Genomics, Gene Center of the LMU Munich	2020-04-22	Max Muenchhoff et al
Germany/BAV-MVP0225/2020	EPI_ISL_466895	Max von Pettenkofer Institute, Virology, National Reference Center for Retroviruses, LMU München	Laboratory for Functional Genome Analysis, Dept. Genomics, Gene Center of the LMU Munich	2020-04-23	Max Muenchhoff et al
Germany/BAV-MVP0226/2020	EPI_ISL_466896	Max von Pettenkofer Institute, Virology, National Reference Center for Retroviruses, LMU München	Laboratory for Functional Genome Analysis, Dept. Genomics, Gene Center of the LMU Munich	2020-04-23	Max Muenchhoff et al
Germany/BAV-MVP0229/2020	EPI_ISL_466897	Max von Pettenkofer Institute, Virology, National Reference Center for Retroviruses, LMU München	Laboratory for Functional Genome Analysis, Dept. Genomics, Gene Center of the LMU Munich	2020-04-24	Max Muenchhoff et al
Germany/BAV-MVP0237/2020	EPI_ISL_466898	Max von Pettenkofer Institute, Virology, National Reference Center for Retroviruses, LMU München	Laboratory for Functional Genome Analysis, Dept. Genomics, Gene Center of the LMU Munich	2020-04-28	Max Muenchhoff et al
Germany/BAV-MVP0240/2020	EPI_ISL_466899	Max von Pettenkofer Institute, Virology, National Reference Center for Retroviruses, LMU München	Laboratory for Functional Genome Analysis, Dept. Genomics, Gene Center of the LMU Munich	2020-04-28	Max Muenchhoff et al
Germany/BAV-MVP0244/2020	EPI_ISL_466900	Max von Pettenkofer Institute, Virology, National Reference Center for Retroviruses, LMU München	Laboratory for Functional Genome Analysis, Dept. Genomics, Gene Center of the LMU Munich	2020-04-30	Max Muenchhoff et al
Germany/BAV-MVP0246/2020	EPI_ISL_466901	Max von Pettenkofer Institute, Virology, National Reference Center for Retroviruses, LMU München	Laboratory for Functional Genome Analysis, Dept. Genomics, Gene Center of the LMU Munich	2020-04-30	Max Muenchhoff et al
Germany/BAV-MVP0247/2020	EPI_ISL_466902	Max von Pettenkofer Institute, Virology, National Reference Center for Retroviruses, LMU München	Laboratory for Functional Genome Analysis, Dept. Genomics, Gene Center of the LMU Munich	2020-04-30	Max Muenchhoff et al
Germany/BAV-MVP0249/2020	EPI_ISL_466903	Max von Pettenkofer Institute, Virology, National Reference Center for Retroviruses, LMU München	Laboratory for Functional Genome Analysis, Dept. Genomics, Gene Center of the LMU Munich	2020-04-30	Max Muenchhoff et al
Germany/BAV-MVP0250/2020	EPI_ISL_466904	Max von Pettenkofer Institute, Virology, National Reference Center for Retroviruses, LMU München	Laboratory for Functional Genome Analysis, Dept. Genomics, Gene Center of the LMU Munich	2020-04-30	Max Muenchhoff et al
Germany/BAV-MVP0253/2020	EPI_ISL_466906	Max von Pettenkofer Institute, Virology, National Reference Center for Retroviruses, LMU München	Laboratory for Functional Genome Analysis, Dept. Genomics, Gene Center of the LMU Munich	2020-05-04	Max Muenchhoff et al
Germany/BAV-MVP0254/2020	EPI_ISL_466907	Max von Pettenkofer Institute, Virology, National Reference Center for Retroviruses, LMU München	Laboratory for Functional Genome Analysis, Dept. Genomics, Gene Center of the LMU Munich	2020-05-04	Max Muenchhoff et al
Germany/BAV-MVP0259/2020	EPI_ISL_466874	Max von Pettenkofer Institute, Virology, National Reference Center for Retroviruses, LMU München	Laboratory for Functional Genome Analysis, Dept. Genomics, Gene Center of the LMU Munich	2020-04-06	Max Muenchhoff et al
Germany/BAV-MVP0264/2020				2020-05-12	

Germany/BAV-MVP0268/2020	EPI_ISL_466910	Max von Pettenkofer Institute, Virology, National Reference Center for Retroviruses, LMU München	Laboratory for Functional Genome Analysis, Dept. Genomics, Gene Center of the LMU Munich	2020-05-17	Max Muenchhoff et al
Germany/BAV-MVP0269/2020	EPI_ISL_466911	Max von Pettenkofer Institute, Virology, National Reference Center for Retroviruses, LMU München	Laboratory for Functional Genome Analysis, Dept. Genomics, Gene Center of the LMU Munich	2020-05-18	Max Muenchhoff et al
Germany/BAV-MVP0270/2020	EPI_ISL_466912	Max von Pettenkofer Institute, Virology, National Reference Center for Retroviruses, LMU München	Laboratory for Functional Genome Analysis, Dept. Genomics, Gene Center of the LMU Munich	2020-05-19	Max Muenchhoff et al
Germany/BAV-MVP0271/2020	EPI_ISL_466914	Max von Pettenkofer Institute, Virology, National Reference Center for Retroviruses, LMU München	Laboratory for Functional Genome Analysis, Dept. Genomics, Gene Center of the LMU Munich	2020-05-20	Max Muenchhoff et al
Germany/BAV-MVP0272/2020	EPI_ISL_466918	Max von Pettenkofer Institute, Virology, National Reference Center for Retroviruses, LMU München	Laboratory for Functional Genome Analysis, Dept. Genomics, Gene Center of the LMU Munich	2020-05-23	Max Muenchhoff et al
Germany/BAV-MVP0273/2020	EPI_ISL_466913	Max von Pettenkofer Institute, Virology, National Reference Center for Retroviruses, LMU München	Laboratory for Functional Genome Analysis, Dept. Genomics, Gene Center of the LMU Munich	2020-05-20	Max Muenchhoff et al
Germany/BAV-MVP0274/2020	EPI_ISL_466915	Max von Pettenkofer Institute, Virology, National Reference Center for Retroviruses, LMU München	Laboratory for Functional Genome Analysis, Dept. Genomics, Gene Center of the LMU Munich	2020-05-22	Max Muenchhoff et al
Germany/BAV-MVP0276/2020	EPI_ISL_466919	Max von Pettenkofer Institute, Virology, National Reference Center for Retroviruses, LMU München	Laboratory for Functional Genome Analysis, Dept. Genomics, Gene Center of the LMU Munich	2020-05-25	Max Muenchhoff et al
Germany/BAV-MVP0277/2020	EPI_ISL_466920	Max von Pettenkofer Institute, Virology, National Reference Center for Retroviruses, LMU München	Laboratory for Functional Genome Analysis, Dept. Genomics, Gene Center of the LMU Munich	2020-05-25	Max Muenchhoff et al
Germany/BAV-MVP0283/2020	EPI_ISL_466909	Max von Pettenkofer Institute, Virology, National Reference Center for Retroviruses, LMU München	Laboratory for Functional Genome Analysis, Dept. Genomics, Gene Center of the LMU Munich	2020-05-11	Max Muenchhoff et al
Germany/BAV-MVP0284/2020	EPI_ISL_466917	Max von Pettenkofer Institute, Virology, National Reference Center for Retroviruses, LMU München	Laboratory for Functional Genome Analysis, Dept. Genomics, Gene Center of the LMU Munich	2020-05-23	Max Muenchhoff et al
Germany/BAV-MVP0287/2020	EPI_ISL_466921	Max von Pettenkofer Institute, Virology, National Reference Center for Retroviruses, LMU München	Laboratory for Functional Genome Analysis, Dept. Genomics, Gene Center of the LMU Munich	2020-05-25	Max Muenchhoff et al
Germany/BAV-MVP0288/2020	EPI_ISL_466916	Max von Pettenkofer Institute, Virology, National Reference Center for Retroviruses, LMU München	Laboratory for Functional Genome Analysis, Dept. Genomics, Gene Center of the LMU Munich	2020-05-22	Max Muenchhoff et al
Germany/BAV-MVP0289/2020	EPI_ISL_466922	Max von Pettenkofer Institute, Virology, National Reference Center for Retroviruses, LMU München	Laboratory for Functional Genome Analysis, Dept. Genomics, Gene Center of the LMU Munich	2020-05-25	Max Muenchhoff et al
Germany/BAV-MVP0291/2020	EPI_ISL_466923	Max von Pettenkofer Institute, Virology, National Reference Center for Retroviruses, LMU München	Laboratory for Functional Genome Analysis, Dept. Genomics, Gene Center of the LMU Munich	2020-05-25	Max Muenchhoff et al
Germany/BAV-MVP0292/2020				2020-05-25	
Germany/BAV-MVP0294/2020	EPI_ISL_466924	Max von Pettenkofer Institute, Virology, National Reference Center for Retroviruses, LMU München	Laboratory for Functional Genome Analysis, Dept. Genomics, Gene Center of the LMU Munich	2020-05-26	Max Muenchhoff et al
Germany/BAV-MVP0295/2020				2020-05-26	
Germany/BAV-MVP0296/2020	EPI_ISL_466925	Max von Pettenkofer Institute, Virology, National Reference Center for Retroviruses, LMU München	Laboratory for Functional Genome Analysis, Dept. Genomics, Gene Center of the LMU Munich	2020-05-26	Max Muenchhoff et al
Germany/BAV-MVP0298/2020				2020-05-22	
Germany/BAV-V2011395/2020	EPI_ISL_420903	Max von Pettenkofer Institute, Virology, National Reference Center for Retroviruses, LMU Munich	Laboratory for Functional Genome Analysis, Dept. Genomics, Gene Center of the LMU Munich	2020-03-14	Max Muenchhoff et al
Germany/BAV-V2011672/2020	EPI_ISL_420905	Max von Pettenkofer Institute, Virology, National Reference Center for Retroviruses, LMU Munich	Laboratory for Functional Genome Analysis, Dept. Genomics, Gene Center of the LMU Munich	2020-03-16	Max Muenchhoff et al
Germany/BAV-V2011673/2020	EPI_ISL_420906	Max von Pettenkofer Institute, Virology, National Reference Center for Retroviruses, LMU Munich	Laboratory for Functional Genome Analysis, Dept. Genomics, Gene Center of the LMU Munich	2020-03-16	Max Muenchhoff et al
Germany/BAV-V2011674/2020				2020-03-16	
Germany/BAV-V2011675/2020	EPI_ISL_420907	Max von Pettenkofer Institute, Virology, National Reference Center for Retroviruses, LMU Munich	Laboratory for Functional Genome Analysis, Dept. Genomics, Gene Center of the LMU Munich	2020-03-16	Max Muenchhoff et al
Germany/BAV-V2012620/2020	EPI_ISL_420909	Max von Pettenkofer Institute, Virology, National Reference Center for Retroviruses, LMU Munich	Laboratory for Functional Genome Analysis, Dept. Genomics, Gene Center of the LMU Munich	2020-03-19	Max Muenchhoff et al
Germany/BAV-V2012856/2020	EPI_ISL_420912	Max von Pettenkofer Institute, Virology, National Reference Center for Retroviruses, LMU Munich	Laboratory for Functional Genome Analysis, Dept. Genomics, Gene Center of the LMU Munich	2020-03-21	Max Muenchhoff et al
Germany/BavPat2/2020	EPI_ISL_414520	Bundeswehr Institute of Microbiology	Bundeswehr Institute of Microbiology	2020-03-02	Mathias C Walter et al
Germany/BavPat3/2020	EPI_ISL_414521	Bundeswehr Institute of Microbiology	Bundeswehr Institute of Microbiology	2020-03-02	Mathias C Walter et al
Germany/Cento-70001814/2020	EPI_ISL_459962	Centogene AG	Centogene AG	2020-04-01	Prof. Dr. Peter Bauer et al
Germany/Cento-70002372/2020	EPI_ISL_459964	Centogene AG	Centogene AG	2020-03-28	Prof. Dr. Peter Bauer et al
Germany/Cento-70004280/2020	EPI_ISL_459963	Centogene AG	Centogene AG	2020-04-01	Prof. Dr. Peter Bauer et al
Germany/COV7/2020-03-26	EPI_ISL_457750	Centogene AG	Centogene AG	2020-03-26	Prof. Dr. Peter Bauer et al
Germany/FrankfurtFFM1/2020	EPI_ISL_452218	University Hospital Frankfurt	Widera/Toptan	2020-03-02	Tuna Toptan1 et al
Germany/FrankfurtFFM3/2020	EPI_ISL_452219	University Hospital Frankfurt	Widera/Toptan	2020-03-02	Tuna Toptan1 et al
Germany/FrankfurtFFM4/2020	EPI_ISL_452220	University Hospital Frankfurt	Widera/Toptan	2020-03-02	Tuna Toptan1 et al
Germany/FrankfurtFFM6/2020	EPI_ISL_452222	University Hospital Frankfurt	Widera/Toptan	2020-03-02	Tuna Toptan1 et al
Germany/FrankfurtFFM7/2020	EPI_ISL_452223	University Hospital Frankfurt	Widera/Toptan	2020-03-02	Tuna Toptan1 et al
Germany/HH-1/2020	EPI_ISL_450413		Center for Diagnostics	2020-03-19	Huang et al
Germany/Muenster_FI1103201/2020	EPI_ISL_463008	Inst. of Molecular Virology	Inst. Hygiene	2020-03-11	Angeles Mecate Zambrano et al
Germany/NRW-011/2020	EPI_ISL_414507	Center of Medical Microbiology, Virology, and Hospital Hygiene, University of Duesseldorf	Center of Medical Microbiology, Virology, and Hospital Hygiene, University of Duesseldorf	2020-03-03	Ortwin Adams et al B
Germany/NRW-12/2020	EPI_ISL_417457	Center of Medical Microbiology, Virology, and Hospital Hygiene, University of Duesseldorf	Center of Medical Microbiology, Virology, and Hospital Hygiene, University of Duesseldorf	2020-03-10	Ortwin Adams et al A (https://dx.doi.org/10.1101/2020.04.25.20079517)
Germany/NRW-13/2020	EPI_ISL_417458	Center of Medical Microbiology, Virology, and Hospital Hygiene, University of Duesseldorf	Center of Medical Microbiology, Virology, and Hospital Hygiene, University of Duesseldorf	2020-03-11	Ortwin Adams et al A (https://dx.doi.org/10.1101/2020.04.25.20079517)
Germany/NRW-18/2020	EPI_ISL_417463	Center of Medical Microbiology, Virology, and Hospital Hygiene, University of Duesseldorf	Center of Medical Microbiology, Virology, and Hospital Hygiene, University of Duesseldorf	2020-03-13	Ortwin Adams et al A (https://dx.doi.org/10.1101/2020.04.25.20079517)
Germany/NRW-19/2020	EPI_ISL_417464	Center of Medical Microbiology, Virology, and Hospital Hygiene, University of Duesseldorf	Center of Medical Microbiology, Virology, and Hospital Hygiene, University of Duesseldorf	2020-03-14	Ortwin Adams et al A (https://dx.doi.org/10.1101/2020.04.25.20079517)
Germany/NRW-20/2020	EPI_ISL_417465	Center of Medical Microbiology, Virology, and Hospital Hygiene, University of Duesseldorf	Center of Medical Microbiology, Virology, and Hospital Hygiene, University of Duesseldorf	2020-03-14	Ortwin Adams et al A (https://dx.doi.org/10.1101/2020.04.25.20079517)
Germany/NRW-21/2020	EPI_ISL_417466	Center of Medical Microbiology, Virology, and Hospital Hygiene, University of Duesseldorf	Center of Medical Microbiology, Virology, and Hospital Hygiene, University of Duesseldorf	2020-03-14	Ortwin Adams et al A (https://dx.doi.org/10.1101/2020.04.25.20079517)
Germany/NRW-22/2020	EPI_ISL_417467	Center of Medical Microbiology, Virology, and Hospital Hygiene, University of Duesseldorf	Center of Medical Microbiology, Virology, and Hospital Hygiene, University of Duesseldorf	2020-03-15	Ortwin Adams et al A (https://dx.doi.org/10.1101/2020.04.25.20079517)
Germany/NRW-23/2020	EPI_ISL_417468	Center of Medical Microbiology, Virology, and Hospital Hygiene, University of Duesseldorf	Center of Medical Microbiology, Virology, and Hospital Hygiene, University of Duesseldorf	2020-03-16	Ortwin Adams et al A (https://dx.doi.org/10.1101/2020.04.25.20079517)
Germany/NRW-24/2020	EPI_ISL_419541	Center of Medical Microbiology, Virology, and Hospital Hygiene, University of Duesseldorf	Center of Medical Microbiology, Virology, and Hospital Hygiene, University of Duesseldorf	2020-03-14	Ortwin Adams et al A (https://dx.doi.org/10.1101/2020.04.25.20079517)
Germany/NRW-25/2020	EPI_ISL_419542	Center of Medical Microbiology, Virology, and Hospital Hygiene, University of Duesseldorf	Center of Medical Microbiology, Virology, and Hospital Hygiene, University of Duesseldorf	2020-03-15	Ortwin Adams et al A (https://dx.doi.org/10.1101/2020.04.25.20079517)
Germany/NRW-26/2020	EPI_ISL_419543	Center of Medical Microbiology, Virology, and Hospital Hygiene, University of Duesseldorf	Center of Medical Microbiology, Virology, and Hospital Hygiene, University of Duesseldorf	2020-03-15	Ortwin Adams et al A (https://dx.doi.org/10.1101/2020.04.25.20079517)
Germany/NRW-27/2020	EPI_ISL_419544	Center of Medical Microbiology, Virology, and Hospital Hygiene, University of Duesseldorf	Center of Medical Microbiology, Virology, and Hospital Hygiene, University of Duesseldorf	2020-03-15	Ortwin Adams et al A (https://dx.doi.org/10.1101/2020.04.25.20079517)
Germany/NRW-28/2020	EPI_ISL_419545	Center of Medical Microbiology, Virology, and Hospital Hygiene, University of Duesseldorf	Center of Medical Microbiology, Virology, and Hospital Hygiene, University of Duesseldorf	2020-03-15	Ortwin Adams et al A (https://dx.doi.org/10.1101/2020.04.25.20079517)
Germany/NRW-29/2020	EPI_ISL_419546	Center of Medical Microbiology, Virology, and Hospital Hygiene, University of Duesseldorf	Center of Medical Microbiology, Virology, and Hospital Hygiene, University of Duesseldorf	2020-03-15	Ortwin Adams et al A (https://dx.doi.org/10.1101/2020.04.25.20079517)
Germany/NRW-30/2020	EPI_ISL_419547	Center of Medical Microbiology, Virology, and Hospital Hygiene, University of Duesseldorf	Center of Medical Microbiology, Virology, and Hospital Hygiene, University of Duesseldorf	2020-03-15	Ortwin Adams et al A (https://dx.doi.org/10.1101/2020.04.25.20079517)
Germany/NRW-32/2020	EPI_ISL_419549	Center of Medical Microbiology, Virology, and Hospital Hygiene, University of Duesseldorf	Center of Medical Microbiology, Virology, and Hospital Hygiene, University of Duesseldorf	2020-03-15	Ortwin Adams et al A (https://dx.doi.org/10.1101/2020.04.25.20079517)
Germany/NRW-33/2020	EPI_ISL_419550	Center of Medical Microbiology, Virology, and Hospital Hygiene, University of Duesseldorf	Center of Medical Microbiology, Virology, and Hospital Hygiene, University of Duesseldorf	2020-03-16	Ortwin Adams et al A (https://dx.doi.org/10.1101/2020.04.25.20079517)
Germany/NRW-34/2020	EPI_ISL_419551	Center of Medical Microbiology, Virology, and Hospital Hygiene, University of Duesseldorf	Center of Medical Microbiology, Virology, and Hospital Hygiene, University of Duesseldorf	2020-03-16	Ortwin Adams et al A (https://dx.doi.org/10.1101/2020.04.25.20079517)
Germany/NRW-35/2020	EPI_ISL_419552	Center of Medical Microbiology, Virology, and Hospital Hygiene, University of Duesseldorf	Center of Medical Microbiology, Virology, and Hospital Hygiene, University of Duesseldorf	2020-03-16	Ortwin Adams et al A (https://dx.doi.org/10.1101/2020.04.25.20079517)
Germany/NRW-36/2020	EPI_ISL_425120	Center of Medical Microbiology, Virology, and Hospital Hygiene, University of Duesseldorf	Center of Medical Microbiology, Virology, and Hospital Hygiene, University of Duesseldorf	2020-03-16	Ortwin Adams et al A (https://dx.doi.org/10.1101/2020.04.25.20079517)
Germany/NRW-37/2020	EPI_ISL_425121	Center of Medical Microbiology, Virology, and Hospital Hygiene, University of Duesseldorf	Center of Medical Microbiology, Virology, and Hospital Hygiene, University of Duesseldorf	2020-03-16	Ortwin Adams et al A (https://dx.doi.org/10.1101/2020.04.25.20079517)
Germany/NRW-38/2020	EPI_ISL_425122	Center of Medical Microbiology, Virology, and Hospital Hygiene, University of Duesseldorf	Center of Medical Microbiology, Virology, and Hospital Hygiene, University of Duesseldorf	2020-03-16	Ortwin Adams et al A (https://dx.doi.org/10.1101/2020.04.25.20079517)
Germany/NRW-39/2020	EPI_ISL_425123	Center of Medical Microbiology, Virology, and Hospital Hygiene, University of Duesseldorf	Center of Medical Microbiology, Virology, and Hospital Hygiene, University of Duesseldorf	2020-03-17	Ortwin Adams et al A (https://dx.doi.org/10.1101/2020.04.25.20079517)
Germany/NRW-40/2020	EPI_ISL_425124	Center of Medical Microbiology, Virology, and Hospital Hygiene, University of Duesseldorf	Center of Medical Microbiology, Virology, and Hospital Hygiene, University of Duesseldorf	2020-03-17	Ortwin Adams et al A (https://dx.doi.org/10.1101/2020.04.25.20079517)
Germany/NRW-41/2020	EPI_ISL_425125	Center of Medical Microbiology, Virology, and Hospital Hygiene, University of Duesseldorf	Center of Medical Microbiology, Virology, and Hospital Hygiene, University of Duesseldorf	2020-03-18	Ortwin Adams et al A (https://dx.doi.org/10.1101/2020.04.25.20079517)
Germany/NRW-42/2020	EPI_ISL_425126	Center of Medical Microbiology, Virology, and Hospital Hygiene, University of Duesseldorf	Center of Medical Microbiology, Virology, and Hospital Hygiene, University of Duesseldorf	2020-03-18	Ortwin Adams et al A (https://dx.doi.org/10.1101/2020.04.25.20079517)
Germany/NRW-43/2020	EPI_ISL_425138	Center of Medical Microbiology, Virology, and Hospital Hygiene, University of Duesseldorf	Center of Medical Microbiology, Virology, and Hospital Hygiene, University of Duesseldorf	2020-03-19	Ortwin Adams et al A (https://dx.doi.org/10.1101/2020.04.25.20079517)
Germany/NRW-44/2020	EPI_ISL_425139	Center of Medical Microbiology, Virology, and Hospital Hygiene, University of Duesseldorf	Center of Medical Microbiology, Virology, and Hospital Hygiene, University of Duesseldorf	2020-03-19	Ortwin Adams et al A (https://dx.doi.org/10.1101/2020.04.25.20079517)
Germany/NRW-45/2020	EPI_ISL_425129	Center of Medical Microbiology, Virology, and Hospital Hygiene, University of Duesseldorf	Center of Medical Microbiology, Virology, and Hospital Hygiene, University of Duesseldorf	2020-03-20	Ortwin Adams et al A (https://dx.doi.org/10.1101/2020.04.25.20079517)
Germany/NRW-46/2020	EPI_ISL_425130	Center of Medical Microbiology, Virology, and Hospital Hygiene, University of Duesseldorf	Center of Medical Microbiology, Virology, and Hospital Hygiene, University of Duesseldorf	2020-03-21	Ortwin Adams et al A (https://dx.doi.org/10.1101/2020.04.25.20079517)
Germany/NRW-47/2020	EPI_ISL_425131	Center of Medical Microbiology, Virology, and Hospital Hygiene, University of Duesseldorf	Center of Medical Microbiology, Virology, and Hospital Hygiene, University of Duesseldorf	2020-03-20	Ortwin Adams et al A (https://dx.doi.org/10.1101/2020.04.25.20079517)
Germany/NRW-49/2020	EPI_ISL_425133	Center of Medical Microbiology, Virology, and Hospital Hygiene, University of Duesseldorf	Center of Medical Microbiology, Virology, and Hospital Hygiene, University of Duesseldorf	2020-03-10	Ortwin Adams et al A (https://dx.doi.org/10.1101/2020.04.25.20079517)
Germany/NRW-51/2020	EPI_ISL_425135	Center of Medical Microbiology, Virology, and Hospital Hygiene, University of Duesseldorf	Center of Medical Microbiology, Virology, and Hospital Hygiene, University of Duesseldorf	2020-03-13	Ortwin Adams et al A (https://dx.doi.org/10.1101/2020.04.25.20079517)
Germany/NRW-52/2020	EPI_ISL_425136	Center of Medical Microbiology, Virology, and Hospital Hygiene, University of Duesseldorf	Center of Medical Microbiology, Virology, and Hospital Hygiene, University of Duesseldorf	2020-03-14	Ortwin Adams et al A (https://dx.doi.org/10.1101/2020.04.25.20079517)
Germany/NRW-53/2020	EPI_ISL_425137	Center of Medical Microbiology, Virology, and Hospital Hygiene, University of Duesseldorf	Center of Medical Microbiology, Virology, and Hospital Hygiene, University of Duesseldorf	2020-03-14	Ortwin Adams et al A (https://dx.doi.org/10.1101/2020.04.25.20079517)
Germany/NRW-55/2020	EPI_ISL_425140	Center of Medical Microbiology, Virology, and Hospital Hygiene, University of Duesseldorf	Center of Medical Microbiology, Virology, and Hospital Hygiene, University of Duesseldorf	2020-03-20	Ortwin Adams et al A (https://dx.doi.org/10.1101/2020.04.25.20079517)
Italy/206/2020	EPI_ISL_457699	Department of Infectious Diseases, Istituto Superiore di Sanità, Roma , Italy	Army Medical and Veterinary Research Center	2020-02-22	Paola Stefanelli et al B
Italy/2389/2020	EPI_ISL_457728	Department of Infectious Diseases, Istituto Superiore di Sanità, Roma , Italy	Army Medical and Veterinary Research Center	2020-03-04	Paola Stefanelli et al B
Italy/2629/2020	EPI_ISL_457700	Department of Infectious Diseases, Istituto Superiore di Sanità, Roma , Italy	Army Medical and Veterinary Research Center	2020-03-06	Paola Stefanelli et al B
Italy/4411/2020	EPI_ISL_457721	Department of Infectious Diseases, Istituto Superiore di Sanità, Roma , Italy	Army Medical and Veterinary Research Center	2020-03-12	Paola Stefanelli et al B
Italy/484/2020	EPI_ISL_457749	Department of Infectious Diseases, Istituto Superiore di Sanità, Roma , Italy	Army Medical and Veterinary Research Center	2020-02-27	Paola Stefanelli et al B
Italy/4870/2020	EPI_ISL_457732	Department of Infectious Diseases, Istituto Superiore di Sanità, Roma , Italy	Army Medical and Veterinary Research Center	2020-03-14	Paola Stefanelli et al B
Italy/4875/2020	EPI_ISL_457724	Department of Infectious Diseases, Istituto Superiore di Sanità, Roma , Italy	Army Medical and Veterinary Research Center	2020-03-15	Paola Stefanelli et al B
Italy/5062/2020	EPI_ISL_457736	Department of Infectious Diseases, Istituto Superiore di Sanità, Roma , Italy	Army Medical and Veterinary Research Center	2020-03-24	Paola Stefanelli et al B
Italy/6193/2020	EPI_ISL_420568	Ospedale Civile Giuseppe Mazzini	Istituto Zooprofilattico Sperimentale dell'Abruzzo e Molise "G. Caporale"	2020-03-23	Lorusso A et al

Italy/AMC1-4634/2020	EPI_ISL_457825	Army Medical Research Center - Scientific Department	Army Medical and Veterinary Research Center	2020-03-25	Silvia Fillo et al
Italy/AMC2-1245/2020	EPI_ISL_457826	Army Medical Center - Scientific Department	Army Medical and Veterinary Research Center	2020-03-08	Silvia Fillo et al
Italy/BG-11639-28Feb/2020	EPI_ISL_460080	Molecular Virology Unit, Fondazione IRCCS Policlinico San Matteo , Pavia	Laboratory of Virology, INMI Lazzaro Spallanzani IRCCS	2020-02-28	Antonio Piralla et al
Italy/BG-5825-22Feb/2020	EPI_ISL_460079	Molecular Virology Unit, Fondazione IRCCS Policlinico San Matteo , Pavia	Laboratory of Virology, INMI Lazzaro Spallanzani IRCCS	2020-02-22	Barbara Bartolini et al
Italy/BG-7158-24Feb/2020	EPI_ISL_460081	Molecular Virology Unit, Fondazione IRCCS Policlinico San Matteo , Pavia	Laboratory of Virology, INMI Lazzaro Spallanzani IRCCS	2020-02-24	Fausto Baldanti et al
Italy/BG-9209-26Feb/2020	EPI_ISL_460082	Molecular Virology Unit, Fondazione IRCCS Policlinico San Matteo , Pavia	Laboratory of Virology, INMI Lazzaro Spallanzani IRCCS	2020-02-26	Martina Rueca et al
Italy/BG-9225-26Feb/2020	EPI_ISL_460083	Molecular Virology Unit, Fondazione IRCCS Policlinico San Matteo , Pavia	Laboratory of Virology, INMI Lazzaro Spallanzani IRCCS	2020-02-26	Martina Rueca et al
Italy/BS-12256-29Feb/2020	EPI_ISL_460084	Molecular Virology Unit, Fondazione IRCCS Policlinico San Matteo , Pavia	Laboratory of Virology, INMI Lazzaro Spallanzani IRCCS	2020-02-29	Fausto Baldanti et al
Italy/CDG1/2020	EPI_ISL_412973	Department of Infectious Diseases, Istituto Superiore di Sanità, Roma , Italy	Virology Laboratory, Scientific Department, Army Medical Center	2020-02-20	Paola Stefanelli et al A (https://dx.doi.org/10.2807/1560-7917.ES.2020.25.13.2000305)
Italy/CR-13221-1Mar/2020	EPI_ISL_460085	Molecular Virology Unit, Fondazione IRCCS Policlinico San Matteo , Pavia	Laboratory of Virology, INMI Lazzaro Spallanzani IRCCS	2020-03-01	Cesare E.M. Gruber et al A
Italy/CR-7070-24Feb/2020	EPI_ISL_460086	Molecular Virology Unit, Fondazione IRCCS Policlinico San Matteo , Pavia	Laboratory of Virology, INMI Lazzaro Spallanzani IRCCS	2020-02-24	Maria R. Capobianchi et al B
Italy/CR-9206-26Feb/2020	EPI_ISL_460087	Molecular Virology Unit, Fondazione IRCCS Policlinico San Matteo , Pavia	Laboratory of Virology, INMI Lazzaro Spallanzani IRCCS	2020-02-26	Cesare E.M. Gruber et al A
Italy/CR-9214-26Feb/2020	EPI_ISL_460088	Molecular Virology Unit, Fondazione IRCCS Policlinico San Matteo , Pavia	Laboratory of Virology, INMI Lazzaro Spallanzani IRCCS	2020-02-26	Martina Rueca et al
Italy/CR-9491-26Feb/2020	EPI_ISL_460089	Molecular Virology Unit, Fondazione IRCCS Policlinico San Matteo , Pavia	Laboratory of Virology, INMI Lazzaro Spallanzani IRCCS	2020-02-26	Antonino Di Caro et al
Italy/FVG-ICGEB_S1/2020	EPI_ISL_417418	Laboratory of Molecular Virology International Center fro Genetic Engineering and Biotechnology (ICGEB)	ARGO Open Lab Platform for Genome sequencing	2020-03-01	Licastro D et al A (https://dx.doi.org/10.1128/JVI.00543-20)
Italy/FVG-ICGEB_S14/2020	EPI_ISL_428853	Laboratory of Molecular Virology International Center for Genetic Engineering and Biotechnology (ICGEB)	ARGO Open Lab Platform for Genome Sequencing	2020-03-30	Licastro D et al B
Italy/FVG-ICGEB_S18/2020	EPI_ISL_428854	Laboratory of Molecular Virology International Center for Genetic Engineering and Biotechnology (ICGEB)	ARGO Open Lab Platform for Genome sequencing	2020-03-30	Licastro D et al B
Italy/FVG-ICGEB_S5/2020	EPI_ISL_417419	Laboratory of Molecular Virology International Center for Genetic Engineering and Biotechnology (ICGEB)	ARGO Open Lab Platform for Genome sequencing	2020-03-01	Licastro D et al A (https://dx.doi.org/10.1128/JVI.00543-20)
Italy/FVG-ICGEB_S8/2020	EPI_ISL_417421	Laboratory of Molecular Virology International Center for Genetic Engineering and Biotechnology (ICGEB)	ARGO Open Lab Platform for Genome sequencing	2020-03-01	Licastro D et al A (https://dx.doi.org/10.1128/JVI.00543-20)
Italy/FVG-ICGEB_S9/2020	EPI_ISL_417423	Laboratory of Molecular Virology International Center for Genetic Engineering and Biotechnology (ICGEB)	ARGO Open Lab Platform for Genome sequencing	2020-03-01	Licastro D et al A (https://dx.doi.org/10.1128/JVI.00543-20)
Italy/INMI1-B1/2020	EPI_ISL_451298	Laboratory of Virology, INMI Lazzaro Spallanzani IRCCS	Laboratory of Virology, INMI Lazzaro Spallanzani IRCCS	2020-02-12	Cesare E.M. Gruber et al A
Italy/INMI1-B2/2020	EPI_ISL_451299	Laboratory of Virology, INMI Lazzaro Spallanzani IRCCS	Laboratory of Virology, INMI Lazzaro Spallanzani IRCCS	2020-02-04	Martina Rueca et al
Italy/INMI1-cs/2020	EPI_ISL_410546	INMI Lazzaro Spallanzani IRCCS	Laboratory of Virology, INMI Lazzaro Spallanzani IRCCS	2020-01-29	Maria R. Capobianchi et al A (https://dx.doi.org/10.1002/jmv.25699)
Italy/INMI1-N/2020	EPI_ISL_451300	Laboratory of Virology, INMI Lazzaro Spallanzani IRCCS	Laboratory of Virology, INMI Lazzaro Spallanzani IRCCS	2020-02-03	Cesare E.M. Gruber et al A
Italy/INMI10/2020	EPI_ISL_424344	INMI Lazzaro Spallanzani IRCCS	Laboratory of Virology, INMI Lazzaro Spallanzani IRCCS	2020-03-04	Eleonora Lalle et al
Italy/INMI11-B/2020	EPI_ISL_451304	Laboratory of Virology, INMI Lazzaro Spallanzani IRCCS	Laboratory of Virology, INMI Lazzaro Spallanzani IRCCS	2020-03-23	Cesare E.M. Gruber et al A
Italy/INMI11-N/2020	EPI_ISL_451305	Laboratory of Virology, INMI Lazzaro Spallanzani IRCCS	Laboratory of Virology, INMI Lazzaro Spallanzani IRCCS	2020-03-23	Martina Rueca et al
Italy/INMI2-N/2020	EPI_ISL_451302	Laboratory of Virology, INMI Lazzaro Spallanzani IRCCS	Laboratory of Virology, INMI Lazzaro Spallanzani IRCCS	2020-01-30	Cesare E.M. Gruber et al B (https://dx.doi.org/10.1002/jmv.25699)
Italy/INMI3/2020	EPI_ISL_417921	INMI Lazzaro Spallanzani IRCCS	Laboratory of Virology, INMI Lazzaro Spallanzani IRCCS	2020-03-01	Martina Rueca et al
Italy/INMI4/2020	EPI_ISL_417922	INMI Lazzaro Spallanzani IRCCS	Laboratory of Virology, INMI Lazzaro Spallanzani IRCCS	2020-02-28	Cesare E. M. Gruber et al
Italy/INMI5-N/2020	EPI_ISL_451303	Laboratory of Virology, INMI Lazzaro Spallanzani IRCCS	Laboratory of Virology, INMI Lazzaro Spallanzani IRCCS	2020-03-04	Martina Rueca et al
Italy/INMI5/2020	EPI_ISL_417923	INMI Lazzaro Spallanzani IRCCS	Laboratory of Virology, INMI Lazzaro Spallanzani IRCCS	2020-03-04	Francesco Messina et al
Italy/INMI6/2020	EPI_ISL_419254	INMI Lazzaro Spallanzani IRCCS	Laboratory of Virology, INMI Lazzaro Spallanzani IRCCS	2020-03-23	Barbara Bartolini et al
Italy/INMI7/2020	EPI_ISL_419255	INMI Lazzaro Spallanzani IRCCS	INMI Lazzaro Spallanzani IRCCS	2020-03-23	Antonino Di Caro et al
Italy/INMI8/2020	EPI_ISL_424342	INMI Lazzaro Spallanzani IRCCS	Laboratory of Virology, INMI Lazzaro Spallanzani IRCCS	2020-03-07	Concetta Castilletti et al
Italy/INMI9/2020	EPI_ISL_424343	INMI Lazzaro Spallanzani IRCCS	Laboratory of Virology, INMI Lazzaro Spallanzani IRCCS	2020-03-23	Fabrizio Carletti et al
Italy/IZSPB_1259/2020	EPI_ISL_451962	Istituto Zooprofilattico Sperimentale Puglia e Basilicata; Dipartimento di Bioscienze, Biotecnologie e Biofarmaceutica dell’Università degli Studi di Bari “A.Moro”; Istituto di Biomembrane. Bioenergetica e Biotecnologie Molecolari del Consiglio Nazionale delle Ricerche di Bari	Beaconlab (Bioinformatics, Evolution and Comparative Genomics lab), Dept of Biosciences, University on Milan	2020-03-20	Parisi A. et al
Italy/IZSPB_703/2020	EPI_ISL_468914	Istituto Zooprofilattico Sperimentale Puglia e Basilicata; Dipartimento di Bioscienze, Biotecnologie e Biofarmaceutica dell’Università degli Studi di Bari “A.Moro”; Istituto di Biomembrane. Bioenergetica e Biotecnologie Molecolari del Consiglio Nazionale delle Ricerche di Bari	Beaconlab (Bioinformatics, Evolution and Comparative Genomics lab), Dept of Biosciences, University on Milan	2020-03-18	Parisi A. et al
Italy/IZSPB_706/2020	EPI_ISL_469016	Istituto Zooprofilattico Sperimentale Puglia e Basilicata; Dipartimento di Bioscienze, Biotecnologie e Biofarmaceutica dell’Università degli Studi di Bari “A.Moro”; Istituto di Biomembrane. Bioenergetica e Biotecnologie Molecolari del Consiglio Nazionale delle Ricerche di Bari	Beaconlab (Bioinformatics, Evolution and Comparative Genomics lab), Dept of Biosciences, University on Milan	2020-03-18	Parisi A. et al
Italy/IZSPB_802/2020	EPI_ISL_469018	Istituto Zooprofilattico Sperimentale Puglia e Basilicata; Dipartimento di Bioscienze, Biotecnologie e Biofarmaceutica dell’Università degli Studi di Bari “A.Moro”; Istituto di Biomembrane. Bioenergetica e Biotecnologie Molecolari del Consiglio Nazionale delle Ricerche di Bari	Beaconlab (Bioinformatics, Evolution and Comparative Genomics lab), Dept of Biosciences, University on Milan	2020-03-19	Parisi A. et al
Italy/IZSPB_804/2020	EPI_ISL_469019	Istituto Zooprofilattico Sperimentale Puglia e Basilicata; Dipartimento di Bioscienze, Biotecnologie e Biofarmaceutica dell’Università degli Studi di Bari “A.Moro”; Istituto di Biomembrane. Bioenergetica e Biotecnologie Molecolari del Consiglio Nazionale delle Ricerche di Bari	Beaconlab (Bioinformatics, Evolution and Comparative Genomics lab), Dept of Biosciences, University on Milan	2020-03-20	Parisi A. et al
Italy/IZSPB_809/2020	EPI_ISL_469020	Istituto Zooprofilattico Sperimentale Puglia e Basilicata; Dipartimento di Bioscienze, Biotecnologie e Biofarmaceutica dell’Università degli Studi di Bari “A.Moro”; Istituto di Biomembrane. Bioenergetica e Biotecnologie Molecolari del Consiglio Nazionale delle Ricerche di Bari	Beaconlab (Bioinformatics, Evolution and Comparative Genomics lab), Dept of Biosciences, University on Milan	2020-03-19	Parisi A. et al
Italy/IZSPB_813/2020	EPI_ISL_469021	Istituto Zooprofilattico Sperimentale Puglia e Basilicata; Dipartimento di Bioscienze, Biotecnologie e Biofarmaceutica dell’Università degli Studi di Bari “A.Moro”; Istituto di Biomembrane. Bioenergetica e Biotecnologie Molecolari del Consiglio Nazionale delle Ricerche di Bari	Beaconlab (Bioinformatics, Evolution and Comparative Genomics lab), Dept of Biosciences, University on Milan	2020-03-20	Parisi A. et al
Italy/IZSPB_847/2020	EPI_ISL_469022	Istituto Zooprofilattico Sperimentale Puglia e Basilicata; Dipartimento di Bioscienze, Biotecnologie e Biofarmaceutica dell’Università degli Studi di Bari “A.Moro”; Istituto di Biomembrane. Bioenergetica e Biotecnologie Molecolari del Consiglio Nazionale delle Ricerche di Bari	Beaconlab (Bioinformatics, Evolution and Comparative Genomics lab), Dept of Biosciences, University on Milan	2020-03-20	Parisi A. et al
Italy/IZSPB_857/2020	EPI_ISL_469023	Istituto Zooprofilattico Sperimentale Puglia e Basilicata; Dipartimento di Bioscienze, Biotecnologie e Biofarmaceutica dell’Università degli Studi di Bari “A.Moro”; Istituto di Biomembrane. Bioenergetica e Biotecnologie Molecolari del Consiglio Nazionale delle Ricerche di Bari	Beaconlab (Bioinformatics, Evolution and Comparative Genomics lab), Dept of Biosciences, University on Milan	2020-03-20	Parisi A. et al
Italy/IZSPB_CL1/2020	EPI_ISL_451961	Istituto Zooprofilattico Sperimentale Puglia e Basilicata; Dipartimento di Bioscienze, Biotecnologie e Biofarmaceutica dell’Università degli Studi di Bari “A.Moro”; Istituto di Biomembrane. Bioenergetica e Biotecnologie Molecolari del Consiglio Nazionale delle Ricerche di Bari	Beaconlab (Bioinformatics Evolution and Comparative Genomics lab), Dept of Biosciences, University of Milan	2020-03-14	Parisi A. et al
Italy/LO-11550-28Feb/2020	EPI_ISL_460090	Molecular Virology Unit, Fondazione IRCCS Policlinico San Matteo , Pavia	Laboratory of Virology, INMI Lazzaro Spallanzani IRCCS	2020-02-28	Antonio Piralla et al
Italy/LO-13075-B/2020	EPI_ISL_451308	Molecular Virology Unit, Fondazione IRCCS Policlinico San Matteo , Pavia	Laboratory of Virology, INMI Lazzaro Spallanzani IRCCS	2020-03-01	Antonio Piralla et al
Italy/LO-13075-N/2020	EPI_ISL_451309	Molecular Virology Unit, Fondazione IRCCS Policlinico San Matteo , Pavia	Laboratory of Virology, INMI Lazzaro Spallanzani IRCCS	2020-03-01	Fausto Baldanti et al
Italy/MI-5925-22Feb/2020	EPI_ISL_460091	Molecular Virology Unit, Fondazione IRCCS Policlinico San Matteo , Pavia	Laboratory of Virology, INMI Lazzaro Spallanzani IRCCS	2020-02-22	Antonino Di Caro et al
Italy/PV-11951-28Feb/2020	EPI_ISL_460092	Molecular Virology Unit, Fondazione IRCCS Policlinico San Matteo , Pavia	Laboratory of Virology, INMI Lazzaro Spallanzani IRCCS	2020-02-28	Cesare E.M. Gruber et al A
Italy/PV-12578-29Feb/2020	EPI_ISL_460093	Molecular Virology Unit, Fondazione IRCCS Policlinico San Matteo , Pavia	Laboratory of Virology, INMI Lazzaro Spallanzani IRCCS	2020-02-29	Maria R. Capobianchi et al B
Italy/PV-5314-B/2020	EPI_ISL_451306	Molecular Virology Unit, Fondazione IRCCS Policlinico San Matteo , Pavia	Laboratory of Virology, INMI Lazzaro Spallanzani IRCCS	2020-02-21	Antonio Piralla et al
Italy/PV-5314-N/2020	EPI_ISL_451307	Molecular Virology Unit, Fondazione IRCCS Policlinico San Matteo , Pavia	Laboratory of Virology, INMI Lazzaro Spallanzani IRCCS	2020-02-21	Fausto Baldanti et al
Italy/PV-8058-25Feb/2020	EPI_ISL_460094	Molecular Virology Unit, Fondazione IRCCS Policlinico San Matteo , Pavia	Laboratory of Virology, INMI Lazzaro Spallanzani IRCCS	2020-02-25	Barbara Bartolini et al
Italy/PV-9675-26Feb/2020	EPI_ISL_460095	Molecular Virology Unit, Fondazione IRCCS Policlinico San Matteo , Pavia	Laboratory of Virology, INMI Lazzaro Spallanzani IRCCS	2020-02-26	Barbara Bartolini et al
ITALY/SardiniaNuoro04039046/2020	EPI_ISL_458084	Laboratorio Biologia Molecolare Sars Cov2 - UOC Laboratorio Analisi - Servizio Medicina di Laboratorio, Ospedale "San Francesco" - ATS-ASSL Nuoro	Laboratorio specialistico UOC Ematologia - Ospedale "San Francesco" - ATS-ASSL Nuoro	2020-04-03	Piras Giovanna et al
ITALY/SardiniaNuoroCOV28/2020	EPI_ISL_458085	Laboratorio Biologia Molecolare Sars Cov2 - UOC Laboratorio Analisi - Servizio Medicina di Laboratorio , Ospedale "San Francesco" - ATS- ASSL Nuoro	Laboratorio specialistico UOC Ematologia - Ospedale "San Francesco"- ATS-ASSL Nuoro	2020-04-12	Piras Giovanna et al
Italy/Siena-1/2020	EPI_ISL_454733		Department of Medical	2020-03-01	Cusi et al
Italy/SPL1/2020	EPI_ISL_412974	Department of Infectious Diseases, Istituto Superiore di Sanità, Rome, Italy	Virology Laboratory, Scientific Department, Army Medical Center	2020-01-29	Paola Stefanelli et al A (https://dx.doi.org/10.2807/1560-7917.ES.2020.25.13.2000305)
Italy/TE12337/2020	EPI_ISL_435146	Villa Serena del Dr. Leonardo Petruzzi	Istituto Zooprofilattico Sperimentale dell’Abruzzo e Molise “G. Caporale”	2020-04-07	Lorusso A et al
Italy/TE12759/2020	EPI_ISL_435147	Villa Serena del Dr. Leonardo Petruzzi	Istituto Zooprofilattico Sperimentale dell’Abruzzo e Molise “G. Caporale”	2020-04-08	Lorusso A et al
Italy/TE12774/2020	EPI_ISL_435150	Ospedale SS Annunziata	Istituto Zooprofilattico Sperimentale dell’Abruzzo e Molise “G. Caporale”	2020-04-08	Lorusso A et al
Italy/TE13457/2020	EPI_ISL_435149	SERVIZIO DI IGIENE E SANITÀ PUBBLICA ASL Teramo	Istituto Zooprofilattico Sperimentale dell’Abruzzo e Molise “G. Caporale”	2020-04-08	Lorusso A et al
Italy/TE13484/2020	EPI_ISL_435151	Ospedale SS Annunziata	Istituto Zooprofilattico Sperimentale dell’Abruzzo e Molise “G. Caporale”	2020-04-08	Lorusso A et al
Italy/TE13491/2020	EPI_ISL_435148	Ospedale SS Annunziata	Istituto Zooprofilattico Sperimentale dell’Abruzzo e Molise “G. Caporale”	2020-04-08	Lorusso A et al
Italy/TE13858/2020	EPI_ISL_435152	Servizio di Igiene, Epidemiologia e Sanità Pubblica (SIESP) Avezzano	Istituto Zooprofilattico Sperimentale dell’Abruzzo e Molise “G. Caporale”	2020-04-09	Lorusso A et al
Italy/TE14162/2020	EPI_ISL_435154	SERVIZIO DI IGIENE E SANITÀ PUBBLICA ASL Teramo	Istituto Zooprofilattico Sperimentale dell’Abruzzo e Molise “G. Caporale”	2020-04-09	Lorusso A et al
Italy/TE14168/2020	EPI_ISL_435155	SERVIZIO DI IGIENE E SANITÀ PUBBLICA ASL Teramo	Istituto Zooprofilattico Sperimentale dell’Abruzzo e Molise “G. Caporale”	2020-04-09	Lorusso A et al
Italy/TE14297/2020	EPI_ISL_435153	SERVIZIO DI IGIENE E SANITÀ PUBBLICA ASL Teramo	Istituto Zooprofilattico Sperimentale dell’Abruzzo e Molise “G. Caporale”	2020-04-09	Lorusso A et al
Italy/TE26423/2020	EPI_ISL_436731	Ospedale Civile S. Liberatore di Atri	Istituto Zooprofilattico Sperimentale dell’Abruzzo e Molise “G. Caporale”	2020-04-26	Lorusso A et al
Italy/TE26425/2020	EPI_ISL_436732	Ospedale Civile S. Liberatore di Atri	Istituto Zooprofilattico Sperimentale dell’Abruzzo e Molise “G. Caporale”	2020-04-27	Lorusso A et al
Italy/TE26533/2020	EPI_ISL_436726	SERVIZIO DI IGIENE E SANITÀ PUBBLICA ASL Teramo	Istituto Zooprofilattico Sperimentale dell’Abruzzo e Molise “G. Caporale”	2020-04-27	Lorusso A et al
Italy/TE26539/2020	EPI_ISL_436727	SERVIZIO DI IGIENE E SANITÀ PUBBLICA ASL Teramo	Istituto Zooprofilattico Sperimentale dell’Abruzzo e Molise “G. Caporale”	2020-04-27	Lorusso A et al
Italy/TE26540/2020	EPI_ISL_436728	SERVIZIO DI IGIENE E SANITÀ PUBBLICA ASL Teramo	Istituto Zooprofilattico Sperimentale dell’Abruzzo e Molise “G. Caporale”	2020-04-27	Lorusso A et al
Italy/TE26643/2020	EPI_ISL_436729	SERVIZIO DI IGIENE E SANITÀ PUBBLICA ASL Teramo	Istituto Zooprofilattico Sperimentale dell’Abruzzo e Molise “G. Caporale”	2020-04-27	Lorusso A et al
Italy/TE27020/2020	EPI_ISL_436725	RSA/RP Villa San Giovanni - Gruppo Edos	Istituto Zooprofilattico Sperimentale dell’Abruzzo e Molise “G. Caporale”	2020-04-27	Lorusso A et al
Italy/TE4836/2020	EPI_ISL_418260	Ospedale Civile Giuseppe Mazzini	Istituto Zooprofilattico Sperimentale dell'Abruzzo e Molise "G. Caporale"	2020-03-16	Lorusso A et al
Italy/TE4880/2020	EPI_ISL_418256	Ospedale “San Liberatore” di Atri	Istituto Zooprofilattico Sperimentale dell'Abruzzo e Molise "G. Caporale"	2020-03-14	Lorusso A et al
Italy/TE4925/2020	EPI_ISL_418255	Presidio Ospedaliero "S. Spirito" - PESCARA	Istituto Zooprofilattico Sperimentale dell'Abruzzo e Molise "G. Caporale"	2020-03-14	Lorusso A et al
Italy/TE4953/2020	EPI_ISL_418258	Presidio ospedaliero "Santo Spirito"	Istituto Zooprofilattico Sperimentale dell'Abruzzo e Molise "G. Caporale"	2020-03-14	Lorusso A et al
Italy/TE5052/2020	EPI_ISL_418261	Ospedale Civile Giuseppe Mazzini	Istituto Zooprofilattico Sperimentale dell'Abruzzo e Molise "G. Caporale"	2020-03-17	Lorusso A et al
Italy/TE5056/2020	EPI_ISL_418257	Ospedale Civile Giuseppe Mazzini, Teramo	Istituto Zooprofilattico Sperimentale dell'Abruzzo e Molise "G.Caporale"	2020-03-17	Lorusso A et al
Italy/TE5166/2020	EPI_ISL_420563	Ospedale Civile Giuseppe Mazzini	Istituto Zooprofilattico Sperimentale dell'Abruzzo e Molise "G. Caporale"	2020-03-18	Lorusso A et al
Italy/TE5472/2020	EPI_ISL_420564	Ospedale Civile Castel Di Sangro	Istituto Zooprofilattico Sperimentale dell’Abruzzo e Molise “G. Caporale”	2020-03-19	Lorusso A et al
Italy/TE5473/2020	EPI_ISL_436718	Ospedale Regionale San Salvatore	Istituto Zooprofilattico Sperimentale dell’Abruzzo e Molise “G. Caporale”	2020-03-19	Lorusso A et al
Italy/TE5476/2020	EPI_ISL_420565	Ospedale Civile Giuseppe Mazzini	Istituto Zooprofilattico Sperimentale dell’Abruzzo e Molise “G. Caporale”	2020-03-19	Lorusso A et al
Italy/TE5512/2020	EPI_ISL_420566	Ospedale Regionale San Salvatore	Istituto Zooprofilattico Sperimentale dell’Abruzzo e Molise “G. Caporale”	2020-03-19	Lorusso A et al
Italy/TE5541/2020	EPI_ISL_436719	Ospedale Civile S. Liberatore di Atri	Istituto Zooprofilattico Sperimentale dell’Abruzzo e Molise “G. Caporale”	2020-03-20	Lorusso A et al
Italy/TE5543/2020	EPI_ISL_436720	Ospedale Civile S. Liberatore di Atri	Istituto Zooprofilattico Sperimentale dell’Abruzzo e Molise “G. Caporale”	2020-03-20	Lorusso A et al
Italy/TE5545/2020	EPI_ISL_436721	Ospedale Civile S. Liberatore di Atri	Istituto Zooprofilattico Sperimentale dell’Abruzzo e Molise “G. Caporale”	2020-03-20	Lorusso A et al
Italy/TE5547/2020	EPI_ISL_436722	Ospedale Civile S. Liberatore di Atri	Istituto Zooprofilattico Sperimentale dell’Abruzzo e Molise “G. Caporale”	2020-03-20	Lorusso A et al
Italy/TE5551/2020	EPI_ISL_429226	Presidio Ospedaliero Santo Spirito	Istituto Zooprofilattico Sperimentale dell'Abruzzo e Molise "G. Caporale"	2020-03-17	Lorusso A et al
Italy/TE5553/2020	EPI_ISL_429227	Presidio Ospedaliero Santo Spirito	Istituto Zooprofilattico Sperimentale dell'Abruzzo e Molise "G. Caporale"	2020-03-17	Lorusso A et al
Italy/TE5674/2020	EPI_ISL_429228	Ospedale Civile Giuseppe Mazzini	Istituto Zooprofilattico Sperimentale dell'Abruzzo e Molise "G. Caporale"	2020-03-20	Lorusso A et al
Italy/TE5687/2020	EPI_ISL_436723	Ospedale Civile Giuseppe Mazzini	Istituto Zooprofilattico Sperimentale dell’Abruzzo e Molise “G. Caporale”	2020-03-20	Lorusso A et al
Italy/TE5780/2020	EPI_ISL_420567	Ospedale Regionale San Salvatore	Istituto Zooprofilattico Sperimentale dell’Abruzzo e Molise “G. Caporale”	2020-03-21	Lorusso A et al
Italy/TE5827/2020	EPI_ISL_436724	Ospedale Civile S. Liberatore di Atri	Istituto Zooprofilattico Sperimentale dell’Abruzzo e Molise “G. Caporale”	2020-03-21	Lorusso A et al
Italy/TE5835/2020	EPI_ISL_429229	Ospedale Regionale San Salvatore	Istituto Zooprofilattico Sperimentale dell'Abruzzo e Molise "G. Caporale"	2020-03-21	Lorusso A et al
Italy/TE6195/2020	EPI_ISL_420569	Ospedale Civile Giuseppe Mazzini	Istituto Zooprofilattico Sperimentale dell’Abruzzo e Molise “G. Caporale”	2020-03-23	Lorusso A et al

Italy/TE6222/2020	EPI_ISL_420583	Ospedale Civile Giuseppe Mazzini	Istituto Zooprofilattico Sperimentale dell'Abruzzo e Molise "G. Caporale"	2020-03-23	Lorusso A et al
Italy/TE6225/2020	EPI_ISL_420592	Ospedale Civile Giuseppe Mazzini	Istituto Zooprofilattico Sperimentale dell'Abruzzo e Molise “G. Caporale”	2020-03-23	Lorusso A et al
Italy/TE6571/2020	EPI_ISL_429231	Ospedale Civile Giuseppe Mazzini	Istituto Zooprofilattico Sperimentale dell'Abruzzo e Molise "G. Caporale"	2020-03-24	Lorusso A et al
Italy/TE6572/2020	EPI_ISL_429232	Ospedale Civile Giuseppe Mazzini	Istituto Zooprofilattico Sperimentale dell'Abruzzo e Molise "G. Caporale"	2020-03-24	Lorusso A et al
Italy/TE6575/2020	EPI_ISL_429233	Ospedale Civile Giuseppe Mazzini	Istituto Zooprofilattico Sperimentale dell'Abruzzo e Molise "G. Caporale"	2020-03-24	Lorusso A et al
Italy/TE6577/2020	EPI_ISL_429234	Ospedale Civile Giuseppe Mazzini	Istituto Zooprofilattico Sperimentale dell'Abruzzo e Molise "G. Caporale"	2020-03-24	Lorusso A et al
Italy/TE6580/2020	EPI_ISL_429235	Ospedale Civile Giuseppe Mazzini	Istituto Zooprofilattico Sperimentale dell'Abruzzo e Molise "G. Caporale"	2020-03-24	Lorusso A et al
Italy/TE6644/2020	EPI_ISL_435145	Ospedale Civile Giuseppe Mazzini	Istituto Zooprofilattico Sperimentale dell'Abruzzo e Molise “G. Caporale”	2020-03-24	Lorusso A et al
Italy/TE7859/2020	EPI_ISL_429236	Ospedale Civile S. Liberatore di Atri	Istituto Zooprofilattico Sperimentale dell'Abruzzo e Molise "G. Caporale"	2020-03-28	Lorusso A et al
Italy/UniMI03/2020	EPI_ISL_417447	Laboratory of Infectious Diseases, Department of Biomedical and Clinical Sciences L. Sacco, University of Milan	Laboratory of Infectious Diseases, Department of Biomedical and Clinical Sciences L. Sacco, University of Milan	2020-02-24	Gianguglielmo Zehender et al
Italy/UniSR1/2020	EPI_ISL_413489	Laboratorio di Microbiologia e Virologia, Università Vita-Salute San Raffaele, Milano	Laboratorio di Microbiologia e Virologia, Università Vita-Salute San Raffaele, Milano	2020-03-03	R.A Diotti et al
Italy/UnivPM1/2020	EPI_ISL_417491	Virology Laboratory, Department of Biomedical Sciences and Public Health, University Politecnica delle Marche	Virology and Legal Medicine Laboratories, Department of Biomedical Sciences and Public Health, University Politecnica delle Marche	2020-03-03	Bagnarelli et al
Italy/VR_20COV10-41/2020	EPI_ISL_452181	ULSS9 Distretto di Bussolengo	Istituto Zooprofilattico Sperimentale delle Venezie	2020-03-22	Adelaide Milani et al
Italy/VR_20COV11-181/2020	EPI_ISL_452182	ULSS9 Distretto di Bussolengo	Istituto Zooprofilattico Sperimentale delle Venezie	2020-03-23	Adelaide Milani et al
Italy/VR_20COV17-11/2020	EPI_ISL_452183	ULSS9 Distretto di Bussolengo	Istituto Zooprofilattico Sperimentale delle Venezie	2020-03-24	Adelaide Milani et al
Italy/VR_20COV18-48/2020	EPI_ISL_452184	ULSS9 Distretto di Bussolengo	Istituto Zooprofilattico Sperimentale delle Venezie	2020-03-24	Adelaide Milani et al
Italy/VR_20COV21-26/2020	EPI_ISL_422437	ULSS9 Distretto di Bussolengo	Istituto Zooprofilattico Sperimentale delle Venezie	2020-03-25	Adelaide Milani et al
Italy/VR_20COV21-37/2020	EPI_ISL_422438	ULSS9 Distretto di Bussolengo	Istituto Zooprofilattico Sperimentale delle Venezie	2020-03-25	Adelaide Milani et al
Italy/VR_20COV23-50/2020	EPI_ISL_452185	ULSS9 Distretto di Bussolengo	Istituto Zooprofilattico Sperimentale delle Venezie	2020-03-26	Adelaide Milani et al
Italy/VR_20COV25-80/2020	EPI_ISL_452186	ULSS9 Distretto di Bussolengo	Istituto Zooprofilattico Sperimentale delle Venezie	2020-03-28	Adelaide Milani et al
Italy/VR_20COV31-18/2020	EPI_ISL_452187	ULSS9 Distretto di Bussolengo	Istituto Zooprofilattico Sperimentale delle Venezie	2020-03-31	Adelaide Milani et al
Italy/VR_20COV31-19/2020	EPI_ISL_452188	ULSS9 Distretto di Bussolengo	Istituto Zooprofilattico Sperimentale delle Venezie	2020-03-31	Adelaide Milani et al
Italy/VR_20COV33-126/2020	EPI_ISL_452189	ULSS9 Distretto di Bussolengo	Istituto Zooprofilattico Sperimentale delle Venezie	2020-04-01	Adelaide Milani et al
Italy/VR_20COV46-23/2020	EPI_ISL_452191	ULSS9 Distretto di San Bonifacio	Istituto Zooprofilattico Sperimentale delle Venezie	2020-04-03	Adelaide Milani et al
Italy/VR_20COV46-8/2020	EPI_ISL_452190	ULSS9 Distretto di San Bonifacio	Istituto Zooprofilattico Sperimentale delle Venezie	2020-04-03	Adelaide Milani et al
Switzerland/100002/2020	EPI_ISL_466927	Viollier AG	Department of Biosystems Science and Engineering, ETH Zürich	2020-03-05	Christian Beisel et al
Switzerland/100003/2020	EPI_ISL_466928	Viollier AG	Department of Biosystems Science and Engineering, ETH Zürich	2020-03-04	Christian Beisel et al
Switzerland/100004/2020	EPI_ISL_466929	Viollier AG	Department of Biosystems Science and Engineering, ETH Zürich	2020-03-06	Christian Beisel et al
Switzerland/100005/2020	EPI_ISL_466930	Viollier AG	Department of Biosystems Science and Engineering, ETH Zürich	2020-03-06	Christian Beisel et al
Switzerland/100006/2020	EPI_ISL_466931	Viollier AG	Department of Biosystems Science and Engineering, ETH Zürich	2020-03-06	Christian Beisel et al
Switzerland/100007/2020	EPI_ISL_466932	Viollier AG	Department of Biosystems Science and Engineering, ETH Zürich	2020-03-05	Christian Beisel et al
Switzerland/100009/2020	EPI_ISL_466934	Viollier AG	Department of Biosystems Science and Engineering, ETH Zürich	2020-03-05	Christian Beisel et al
Switzerland/100010/2020	EPI_ISL_466935	Viollier AG	Department of Biosystems Science and Engineering, ETH Zürich	2020-03-05	Christian Beisel et al
Switzerland/100011/2020	EPI_ISL_466936	Viollier AG	Department of Biosystems Science and Engineering, ETH Zürich	2020-03-06	Christian Beisel et al
Switzerland/100012/2020	EPI_ISL_466937	Viollier AG	Department of Biosystems Science and Engineering, ETH Zürich	2020-03-06	Christian Beisel et al
Switzerland/100013/2020	EPI_ISL_466938	Viollier AG	Department of Biosystems Science and Engineering, ETH Zürich	2020-03-05	Christian Beisel et al
Switzerland/100014/2020	EPI_ISL_466939	Viollier AG	Department of Biosystems Science and Engineering, ETH Zürich	2020-03-08	Christian Beisel et al
Switzerland/100016/2020	EPI_ISL_466940	Viollier AG	Department of Biosystems Science and Engineering, ETH Zürich	2020-03-08	Christian Beisel et al
Switzerland/100017/2020	EPI_ISL_466941	Viollier AG	Department of Biosystems Science and Engineering, ETH Zürich	2020-03-08	Christian Beisel et al
Switzerland/100018/2020	EPI_ISL_466942	Viollier AG	Department of Biosystems Science and Engineering, ETH Zürich	2020-03-08	Christian Beisel et al
Switzerland/100019/2020	EPI_ISL_466943	Viollier AG	Department of Biosystems Science and Engineering, ETH Zürich	2020-03-09	Christian Beisel et al
Switzerland/100020/2020	EPI_ISL_466944	Viollier AG	Department of Biosystems Science and Engineering, ETH Zürich	2020-03-09	Christian Beisel et al
Switzerland/100021/2020	EPI_ISL_466945	Viollier AG	Department of Biosystems Science and Engineering, ETH Zürich	2020-03-09	Christian Beisel et al
Switzerland/100022/2020	EPI_ISL_466946	Viollier AG	Department of Biosystems Science and Engineering, ETH Zürich	2020-03-10	Christian Beisel et al
Switzerland/100023/2020	EPI_ISL_466947	Viollier AG	Department of Biosystems Science and Engineering, ETH Zürich	2020-03-10	Christian Beisel et al
Switzerland/100024/2020	EPI_ISL_466948	Viollier AG	Department of Biosystems Science and Engineering, ETH Zürich	2020-03-10	Christian Beisel et al
Switzerland/100025/2020	EPI_ISL_466949	Viollier AG	Department of Biosystems Science and Engineering, ETH Zürich	2020-03-10	Christian Beisel et al
Switzerland/100026/2020	EPI_ISL_466950	Viollier AG	Department of Biosystems Science and Engineering, ETH Zürich	2020-03-09	Christian Beisel et al
Switzerland/100027/2020	EPI_ISL_466951	Viollier AG	Department of Biosystems Science and Engineering, ETH Zürich	2020-03-10	Christian Beisel et al
Switzerland/100028/2020	EPI_ISL_466952	Viollier AG	Department of Biosystems Science and Engineering, ETH Zürich	2020-03-10	Christian Beisel et al
Switzerland/100029/2020	EPI_ISL_466953	Viollier AG	Department of Biosystems Science and Engineering, ETH Zürich	2020-03-10	Christian Beisel et al
Switzerland/100030/2020	EPI_ISL_466954	Viollier AG	Department of Biosystems Science and Engineering, ETH Zürich	2020-03-10	Christian Beisel et al
Switzerland/100031/2020	EPI_ISL_466955	Viollier AG	Department of Biosystems Science and Engineering, ETH Zürich	2020-03-10	Christian Beisel et al
Switzerland/100032/2020	EPI_ISL_466956	Viollier AG	Department of Biosystems Science and Engineering, ETH Zürich	2020-03-10	Christian Beisel et al
Switzerland/100033/2020	EPI_ISL_466957	Viollier AG	Department of Biosystems Science and Engineering, ETH Zürich	2020-03-10	Christian Beisel et al
Switzerland/100034/2020	EPI_ISL_466958	Viollier AG	Department of Biosystems Science and Engineering, ETH Zürich	2020-03-11	Christian Beisel et al
Switzerland/100035/2020	EPI_ISL_466959	Viollier AG	Department of Biosystems Science and Engineering, ETH Zürich	2020-03-11	Christian Beisel et al
Switzerland/100036/2020	EPI_ISL_466960	Viollier AG	Department of Biosystems Science and Engineering, ETH Zürich	2020-03-11	Christian Beisel et al
Switzerland/100037/2020	EPI_ISL_466961	Viollier AG	Department of Biosystems Science and Engineering, ETH Zürich	2020-03-11	Christian Beisel et al
Switzerland/100039/2020	EPI_ISL_466962	Viollier AG	Department of Biosystems Science and Engineering, ETH Zürich	2020-03-11	Christian Beisel et al
Switzerland/100040/2020	EPI_ISL_466963	Viollier AG	Department of Biosystems Science and Engineering, ETH Zürich	2020-03-11	Christian Beisel et al
Switzerland/100041/2020	EPI_ISL_466964	Viollier AG	Department of Biosystems Science and Engineering, ETH Zürich	2020-03-11	Christian Beisel et al
Switzerland/100042/2020	EPI_ISL_466965	Viollier AG	Department of Biosystems Science and Engineering, ETH Zürich	2020-03-11	Christian Beisel et al
Switzerland/100043/2020	EPI_ISL_466966	Viollier AG	Department of Biosystems Science and Engineering, ETH Zürich	2020-03-11	Christian Beisel et al
Switzerland/100044/2020	EPI_ISL_466967	Viollier AG	Department of Biosystems Science and Engineering, ETH Zürich	2020-03-11	Christian Beisel et al
Switzerland/100045/2020	EPI_ISL_466968	Viollier AG	Department of Biosystems Science and Engineering, ETH Zürich	2020-03-11	Christian Beisel et al
Switzerland/100047/2020	EPI_ISL_466969	Viollier AG	Department of Biosystems Science and Engineering, ETH Zürich	2020-03-11	Christian Beisel et al
Switzerland/1000477102/2020	EPI_ISL_413019	Department of Internal Medicine, Triemli Hospital	Institute of Medical Virology, University of Zurich	2020-02-26	Stefan Schmutz et al A
Switzerland/1000477377/2020	EPI_ISL_413020	Department of Internal Medicine, Triemli Hospital	Institute of Medical Virology, University of Zurich	2020-02-27	Stefan Schmutz et al C (https://virological.org/t/phylodynamic-analyses-based-on-11-genomes-from-the-italian-outbreak/426)
Switzerland/1000477757/2020	EPI_ISL_413021	Klinik Hirslanden Zurich	Institute of Medical Virology, University of Zurich	2020-02-29	Stefan Schmutz et al B (https://dx.doi.org/10.1002/jmv.25773)
Switzerland/1000477796/2020	EPI_ISL_413022	Division of Infectious Diseases, University Hospital Zurich	Institute of Medical Virology, University of Zurich	2020-02-29	Stefan Schmutz et al B (https://dx.doi.org/10.1002/jmv.25773)
Switzerland/1000477797/2020	EPI_ISL_413023	Division of Infectious Diseases, University Hospital Zurich	Institute of Medical Virology, University of Zurich	2020-02-29	Stefan Schmutz et al B (https://dx.doi.org/10.1002/jmv.25773)
Switzerland/1000477806/2020	EPI_ISL_413024	Division of Infectious Diseases, University Hospital Zurich	Institute of Medical Virology, University of Zurich	2020-02-29	Stefan Schmutz et al B (https://dx.doi.org/10.1002/jmv.25773)
Switzerland/100048/2020	EPI_ISL_466970	Viollier AG	Department of Biosystems Science and Engineering, ETH Zürich	2020-03-11	Christian Beisel et al
Switzerland/100049/2020	EPI_ISL_466971	Viollier AG	Department of Biosystems Science and Engineering, ETH Zürich	2020-03-11	Christian Beisel et al
Switzerland/100050/2020	EPI_ISL_466972	Viollier AG	Department of Biosystems Science and Engineering, ETH Zürich	2020-03-11	Christian Beisel et al
Switzerland/100051/2020	EPI_ISL_466973	Viollier AG	Department of Biosystems Science and Engineering, ETH Zürich	2020-03-11	Christian Beisel et al
Switzerland/100052/2020	EPI_ISL_466974	Viollier AG	Department of Biosystems Science and Engineering, ETH Zürich	2020-03-12	Christian Beisel et al
Switzerland/100053/2020	EPI_ISL_466975	Viollier AG	Department of Biosystems Science and Engineering, ETH Zürich	2020-03-12	Christian Beisel et al
Switzerland/100054/2020	EPI_ISL_466976	Viollier AG	Department of Biosystems Science and Engineering, ETH Zürich	2020-03-12	Christian Beisel et al
Switzerland/100055/2020	EPI_ISL_466977	Viollier AG	Department of Biosystems Science and Engineering, ETH Zürich	2020-03-12	Christian Beisel et al
Switzerland/100057/2020	EPI_ISL_466979	Viollier AG	Department of Biosystems Science and Engineering, ETH Zürich	2020-03-12	Christian Beisel et al
Switzerland/100058/2020	EPI_ISL_466980	Viollier AG	Department of Biosystems Science and Engineering, ETH Zürich	2020-03-12	Christian Beisel et al
Switzerland/100059/2020	EPI_ISL_466981	Viollier AG	Department of Biosystems Science and Engineering, ETH Zürich	2020-03-12	Christian Beisel et al
Switzerland/100060/2020	EPI_ISL_466982	Viollier AG	Department of Biosystems Science and Engineering, ETH Zürich	2020-03-12	Christian Beisel et al
Switzerland/100062/2020	EPI_ISL_466984	Viollier AG	Department of Biosystems Science and Engineering, ETH Zürich	2020-03-12	Christian Beisel et al
Switzerland/100063/2020	EPI_ISL_466985	Viollier AG	Department of Biosystems Science and Engineering, ETH Zürich	2020-03-12	Christian Beisel et al
Switzerland/100064/2020	EPI_ISL_466986	Viollier AG	Department of Biosystems Science and Engineering, ETH Zürich	2020-03-12	Christian Beisel et al
Switzerland/100065/2020	EPI_ISL_466987	Viollier AG	Department of Biosystems Science and Engineering, ETH Zürich	2020-03-12	Christian Beisel et al
Switzerland/100066/2020	EPI_ISL_466988	Viollier AG	Department of Biosystems Science and Engineering, ETH Zürich	2020-03-12	Christian Beisel et al
Switzerland/100067/2020	EPI_ISL_466989	Viollier AG	Department of Biosystems Science and Engineering, ETH Zürich	2020-03-12	Christian Beisel et al
Switzerland/100068/2020	EPI_ISL_466990	Viollier AG	Department of Biosystems Science and Engineering, ETH Zürich	2020-03-12	Christian Beisel et al
Switzerland/100069/2020	EPI_ISL_466991	Viollier AG	Department of Biosystems Science and Engineering, ETH Zürich	2020-03-12	Christian Beisel et al
Switzerland/100070/2020	EPI_ISL_466992	Viollier AG	Department of Biosystems Science and Engineering, ETH Zürich	2020-03-12	Christian Beisel et al
Switzerland/100071/2020	EPI_ISL_466993	Viollier AG	Department of Biosystems Science and Engineering, ETH Zürich	2020-03-12	Christian Beisel et al
Switzerland/100072/2020	EPI_ISL_466994	Viollier AG	Department of Biosystems Science and Engineering, ETH Zürich	2020-03-12	Christian Beisel et al
Switzerland/100073/2020	EPI_ISL_466995	Viollier AG	Department of Biosystems Science and Engineering, ETH Zürich	2020-03-12	Christian Beisel et al
Switzerland/100074/2020	EPI_ISL_466996	Viollier AG	Department of Biosystems Science and Engineering, ETH Zürich	2020-03-12	Christian Beisel et al
Switzerland/100075/2020	EPI_ISL_451668	Viollier AG	Department of Biosystems Science and Engineering, ETH Zürich	2020-03-12	Christian Beisel et al
Switzerland/100076/2020	EPI_ISL_451669	Viollier AG	Department of Biosystems Science and Engineering, ETH Zürich	2020-03-12	Christian Beisel et al
Switzerland/100077/2020	EPI_ISL_451670	Viollier AG	Department of Biosystems Science and Engineering, ETH Zürich	2020-03-12	Christian Beisel et al
Switzerland/100079/2020	EPI_ISL_451671	Viollier AG	Department of Biosystems Science and Engineering, ETH Zürich	2020-03-12	Christian Beisel et al
Switzerland/100080/2020	EPI_ISL_451672	Viollier AG	Department of Biosystems Science and Engineering, ETH Zürich	2020-03-13	Christian Beisel et al
Switzerland/100081/2020	EPI_ISL_451673	Viollier AG	Department of Biosystems Science and Engineering, ETH Zürich	2020-03-13	Christian Beisel et al
Switzerland/100082/2020	EPI_ISL_451674	Viollier AG	Department of Biosystems Science and Engineering, ETH Zürich	2020-03-13	Christian Beisel et al
Switzerland/100083/2020	EPI_ISL_451675	Viollier AG	Department of Biosystems Science and Engineering, ETH Zürich	2020-03-13	Christian Beisel et al
Switzerland/100084/2020	EPI_ISL_451676	Viollier AG	Department of Biosystems Science and Engineering, ETH Zürich	2020-03-13	Christian Beisel et al
Switzerland/100085/2020	EPI_ISL_451677	Viollier AG	Department of Biosystems Science and Engineering, ETH Zürich	2020-03-13	Christian Beisel et al
Switzerland/100086/2020	EPI_ISL_451678	Viollier AG	Department of Biosystems Science and Engineering, ETH Zürich	2020-03-13	Christian Beisel et al
Switzerland/100087/2020	EPI_ISL_451679	Viollier AG	Department of Biosystems Science and Engineering, ETH Zürich	2020-03-13	Christian Beisel et al
Switzerland/100089/2020	EPI_ISL_451681	Viollier AG	Department of Biosystems Science and Engineering, ETH Zürich	2020-03-12	Christian Beisel et al
Switzerland/100090/2020	EPI_ISL_451682	Viollier AG	Department of Biosystems Science and Engineering, ETH Zürich	2020-03-13	Christian Beisel et al
Switzerland/100091/2020	EPI_ISL_451683	Viollier AG	Department of Biosystems Science and Engineering, ETH Zürich	2020-03-13	Christian Beisel et al

Switzerland/100092/2020	EPI_ISL_451684	Viollier AG	Department of Biosystems Science and Engineering, ETH Zürich	2020-03-13	Christian Beisel et al
Switzerland/100093/2020	EPI_ISL_451685	Viollier AG	Department of Biosystems Science and Engineering, ETH Zürich	2020-03-13	Christian Beisel et al
Switzerland/100094/2020	EPI_ISL_451686	Viollier AG	Department of Biosystems Science and Engineering, ETH Zürich	2020-03-13	Christian Beisel et al
Switzerland/100095/2020	EPI_ISL_451687	Viollier AG	Department of Biosystems Science and Engineering, ETH Zürich	2020-03-13	Christian Beisel et al
Switzerland/100096/2020	EPI_ISL_451688	Viollier AG	Department of Biosystems Science and Engineering, ETH Zürich	2020-03-12	Christian Beisel et al
Switzerland/100097/2020	EPI_ISL_451689	Viollier AG	Department of Biosystems Science and Engineering, ETH Zürich	2020-03-13	Christian Beisel et al
Switzerland/100098/2020	EPI_ISL_451690	Viollier AG	Department of Biosystems Science and Engineering, ETH Zürich	2020-03-13	Christian Beisel et al
Switzerland/100099/2020	EPI_ISL_451691	Viollier AG	Department of Biosystems Science and Engineering, ETH Zürich	2020-03-13	Christian Beisel et al
Switzerland/100108/2020	EPI_ISL_451692	Viollier AG	Department of Biosystems Science and Engineering, ETH Zürich	2020-03-14	Christian Beisel et al
Switzerland/100109/2020	EPI_ISL_451693	Viollier AG	Department of Biosystems Science and Engineering, ETH Zürich	2020-03-14	Christian Beisel et al
Switzerland/100111/2020	EPI_ISL_451695	Viollier AG	Department of Biosystems Science and Engineering, ETH Zürich	2020-03-16	Christian Beisel et al
Switzerland/100112/2020	EPI_ISL_451696	Viollier AG	Department of Biosystems Science and Engineering, ETH Zürich	2020-03-16	Christian Beisel et al
Switzerland/100113/2020	EPI_ISL_451697	Viollier AG	Department of Biosystems Science and Engineering, ETH Zürich	2020-03-16	Christian Beisel et al
Switzerland/100114/2020	EPI_ISL_451698	Viollier AG	Department of Biosystems Science and Engineering, ETH Zürich	2020-03-16	Christian Beisel et al
Switzerland/100115/2020	EPI_ISL_451699	Viollier AG	Department of Biosystems Science and Engineering, ETH Zürich	2020-03-16	Christian Beisel et al
Switzerland/100116/2020	EPI_ISL_451700	Viollier AG	Department of Biosystems Science and Engineering, ETH Zürich	2020-03-16	Christian Beisel et al
Switzerland/100117/2020	EPI_ISL_451701	Viollier AG	Department of Biosystems Science and Engineering, ETH Zürich	2020-03-16	Christian Beisel et al
Switzerland/100118/2020	EPI_ISL_451702	Viollier AG	Department of Biosystems Science and Engineering, ETH Zürich	2020-03-16	Christian Beisel et al
Switzerland/100119/2020	EPI_ISL_451703	Viollier AG	Department of Biosystems Science and Engineering, ETH Zürich	2020-03-16	Christian Beisel et al
Switzerland/100120/2020	EPI_ISL_451704	Viollier AG	Department of Biosystems Science and Engineering, ETH Zürich	2020-03-16	Christian Beisel et al
Switzerland/100121/2020	EPI_ISL_451705	Viollier AG	Department of Biosystems Science and Engineering, ETH Zürich	2020-03-16	Christian Beisel et al
Switzerland/100122/2020	EPI_ISL_451706	Viollier AG	Department of Biosystems Science and Engineering, ETH Zürich	2020-03-16	Christian Beisel et al
Switzerland/100123/2020	EPI_ISL_451707	Viollier AG	Department of Biosystems Science and Engineering, ETH Zürich	2020-03-16	Christian Beisel et al
Switzerland/100124/2020	EPI_ISL_451708	Viollier AG	Department of Biosystems Science and Engineering, ETH Zürich	2020-03-16	Christian Beisel et al
Switzerland/100125/2020	EPI_ISL_451709	Viollier AG	Department of Biosystems Science and Engineering, ETH Zürich	2020-03-16	Christian Beisel et al
Switzerland/100126/2020	EPI_ISL_451710	Viollier AG	Department of Biosystems Science and Engineering, ETH Zürich	2020-03-16	Christian Beisel et al
Switzerland/100127/2020	EPI_ISL_451711	Viollier AG	Department of Biosystems Science and Engineering, ETH Zürich	2020-03-16	Christian Beisel et al
Switzerland/100128/2020	EPI_ISL_451712	Viollier AG	Department of Biosystems Science and Engineering, ETH Zürich	2020-03-16	Christian Beisel et al
Switzerland/100129/2020	EPI_ISL_451713	Viollier AG	Department of Biosystems Science and Engineering, ETH Zürich	2020-03-16	Christian Beisel et al
Switzerland/100130/2020	EPI_ISL_451714	Viollier AG	Department of Biosystems Science and Engineering, ETH Zürich	2020-03-16	Christian Beisel et al
Switzerland/100132/2020	EPI_ISL_451716	Viollier AG	Department of Biosystems Science and Engineering, ETH Zürich	2020-03-16	Christian Beisel et al
Switzerland/100133/2020	EPI_ISL_451717	Viollier AG	Department of Biosystems Science and Engineering, ETH Zürich	2020-03-16	Christian Beisel et al
Switzerland/100135/2020	EPI_ISL_451719	Viollier AG	Department of Biosystems Science and Engineering, ETH Zürich	2020-03-16	Christian Beisel et al
Switzerland/100136/2020	EPI_ISL_451720	Viollier AG	Department of Biosystems Science and Engineering, ETH Zürich	2020-03-16	Christian Beisel et al
Switzerland/100137/2020	EPI_ISL_451721	Viollier AG	Department of Biosystems Science and Engineering, ETH Zürich	2020-03-16	Christian Beisel et al
Switzerland/100138/2020	EPI_ISL_451722	Viollier AG	Department of Biosystems Science and Engineering, ETH Zürich	2020-03-15	Christian Beisel et al
Switzerland/100139/2020	EPI_ISL_451723	Viollier AG	Department of Biosystems Science and Engineering, ETH Zürich	2020-03-16	Christian Beisel et al
Switzerland/100140/2020	EPI_ISL_451724	Viollier AG	Department of Biosystems Science and Engineering, ETH Zürich	2020-03-16	Christian Beisel et al
Switzerland/100141/2020	EPI_ISL_451725	Viollier AG	Department of Biosystems Science and Engineering, ETH Zürich	2020-03-15	Christian Beisel et al
Switzerland/100142/2020	EPI_ISL_451726	Viollier AG	Department of Biosystems Science and Engineering, ETH Zürich	2020-03-16	Christian Beisel et al
Switzerland/100143/2020	EPI_ISL_451727	Viollier AG	Department of Biosystems Science and Engineering, ETH Zürich	2020-03-16	Christian Beisel et al
Switzerland/100144/2020	EPI_ISL_451728	Viollier AG	Department of Biosystems Science and Engineering, ETH Zürich	2020-03-16	Christian Beisel et al
Switzerland/100145/2020	EPI_ISL_451729	Viollier AG	Department of Biosystems Science and Engineering, ETH Zürich	2020-03-16	Christian Beisel et al
Switzerland/100146/2020	EPI_ISL_451730	Viollier AG	Department of Biosystems Science and Engineering, ETH Zürich	2020-03-16	Christian Beisel et al
Switzerland/100147/2020	EPI_ISL_451731	Viollier AG	Department of Biosystems Science and Engineering, ETH Zürich	2020-03-16	Christian Beisel et al
Switzerland/100148/2020	EPI_ISL_451732	Viollier AG	Department of Biosystems Science and Engineering, ETH Zürich	2020-03-16	Christian Beisel et al
Switzerland/100150/2020	EPI_ISL_451734	Viollier AG	Department of Biosystems Science and Engineering, ETH Zürich	2020-03-16	Christian Beisel et al
Switzerland/100152/2020	EPI_ISL_451736	Viollier AG	Department of Biosystems Science and Engineering, ETH Zürich	2020-03-16	Christian Beisel et al
Switzerland/100153/2020	EPI_ISL_451737	Viollier AG	Department of Biosystems Science and Engineering, ETH Zürich	2020-03-16	Christian Beisel et al
Switzerland/100154/2020	EPI_ISL_451738	Viollier AG	Department of Biosystems Science and Engineering, ETH Zürich	2020-03-16	Christian Beisel et al
Switzerland/100155/2020	EPI_ISL_451739	Viollier AG	Department of Biosystems Science and Engineering, ETH Zürich	2020-03-16	Christian Beisel et al
Switzerland/100790/2020	EPI_ISL_451740	Viollier AG	Department of Biosystems Science and Engineering, ETH Zürich	2020-03-25	Christian Beisel et al
Switzerland/100791/2020	EPI_ISL_451741	Viollier AG	Department of Biosystems Science and Engineering, ETH Zürich	2020-03-25	Christian Beisel et al
Switzerland/100792/2020	EPI_ISL_451742	Viollier AG	Department of Biosystems Science and Engineering, ETH Zürich	2020-03-25	Christian Beisel et al
Switzerland/100794/2020	EPI_ISL_451744	Viollier AG	Department of Biosystems Science and Engineering, ETH Zürich	2020-03-25	Christian Beisel et al
Switzerland/100795/2020	EPI_ISL_451745	Viollier AG	Department of Biosystems Science and Engineering, ETH Zürich	2020-03-25	Christian Beisel et al
Switzerland/100796/2020	EPI_ISL_451746	Viollier AG	Department of Biosystems Science and Engineering, ETH Zürich	2020-03-25	Christian Beisel et al
Switzerland/100797/2020	EPI_ISL_451747	Viollier AG	Department of Biosystems Science and Engineering, ETH Zürich	2020-03-25	Christian Beisel et al
Switzerland/100798/2020	EPI_ISL_451748	Viollier AG	Department of Biosystems Science and Engineering, ETH Zürich	2020-03-25	Christian Beisel et al
Switzerland/100799/2020	EPI_ISL_451749	Viollier AG	Department of Biosystems Science and Engineering, ETH Zürich	2020-03-25	Christian Beisel et al
Switzerland/100800/2020	EPI_ISL_451750	Viollier AG	Department of Biosystems Science and Engineering, ETH Zürich	2020-03-25	Christian Beisel et al
Switzerland/100801/2020	EPI_ISL_451751	Viollier AG	Department of Biosystems Science and Engineering, ETH Zürich	2020-03-25	Christian Beisel et al
Switzerland/100802/2020	EPI_ISL_451752	Viollier AG	Department of Biosystems Science and Engineering, ETH Zürich	2020-03-25	Christian Beisel et al
Switzerland/100803/2020	EPI_ISL_451753	Viollier AG	Department of Biosystems Science and Engineering, ETH Zürich	2020-03-25	Christian Beisel et al
Switzerland/100804/2020	EPI_ISL_451754	Viollier AG	Department of Biosystems Science and Engineering, ETH Zürich	2020-03-25	Christian Beisel et al
Switzerland/100805/2020	EPI_ISL_451755	Viollier AG	Department of Biosystems Science and Engineering, ETH Zürich	2020-03-25	Christian Beisel et al
Switzerland/100806/2020	EPI_ISL_451756	Viollier AG	Department of Biosystems Science and Engineering, ETH Zürich	2020-03-25	Christian Beisel et al
Switzerland/100807/2020	EPI_ISL_451757	Viollier AG	Department of Biosystems Science and Engineering, ETH Zürich	2020-03-25	Christian Beisel et al
Switzerland/100808/2020	EPI_ISL_451758	Viollier AG	Department of Biosystems Science and Engineering, ETH Zürich	2020-03-26	Christian Beisel et al
Switzerland/100809/2020	EPI_ISL_451759	Viollier AG	Department of Biosystems Science and Engineering, ETH Zürich	2020-03-26	Christian Beisel et al
Switzerland/100810/2020	EPI_ISL_451760	Viollier AG	Department of Biosystems Science and Engineering, ETH Zürich	2020-03-26	Christian Beisel et al
Switzerland/100811/2020	EPI_ISL_451761	Viollier AG	Department of Biosystems Science and Engineering, ETH Zürich	2020-03-26	Christian Beisel et al
Switzerland/100812/2020	EPI_ISL_451762	Viollier AG	Department of Biosystems Science and Engineering, ETH Zürich	2020-03-26	Christian Beisel et al
Switzerland/100813/2020	EPI_ISL_451763	Viollier AG	Department of Biosystems Science and Engineering, ETH Zürich	2020-03-26	Christian Beisel et al
Switzerland/100814/2020	EPI_ISL_451764	Viollier AG	Department of Biosystems Science and Engineering, ETH Zürich	2020-03-26	Christian Beisel et al
Switzerland/100815/2020	EPI_ISL_451765	Viollier AG	Department of Biosystems Science and Engineering, ETH Zürich	2020-03-26	Christian Beisel et al
Switzerland/100816/2020	EPI_ISL_451766	Viollier AG	Department of Biosystems Science and Engineering, ETH Zürich	2020-03-26	Christian Beisel et al
Switzerland/100817/2020	EPI_ISL_451767	Viollier AG	Department of Biosystems Science and Engineering, ETH Zürich	2020-03-26	Christian Beisel et al
Switzerland/100820/2020	EPI_ISL_451769	Viollier AG	Department of Biosystems Science and Engineering, ETH Zürich	2020-03-26	Christian Beisel et al
Switzerland/100821/2020	EPI_ISL_451770	Viollier AG	Department of Biosystems Science and Engineering, ETH Zürich	2020-03-26	Christian Beisel et al
Switzerland/100822/2020	EPI_ISL_451771	Viollier AG	Department of Biosystems Science and Engineering, ETH Zürich	2020-03-26	Christian Beisel et al
Switzerland/100823/2020	EPI_ISL_451772	Viollier AG	Department of Biosystems Science and Engineering, ETH Zürich	2020-03-26	Christian Beisel et al
Switzerland/100824/2020	EPI_ISL_451773	Viollier AG	Department of Biosystems Science and Engineering, ETH Zürich	2020-03-26	Christian Beisel et al
Switzerland/100826/2020	EPI_ISL_451775	Viollier AG	Department of Biosystems Science and Engineering, ETH Zürich	2020-03-26	Christian Beisel et al
Switzerland/100827/2020	EPI_ISL_451776	Viollier AG	Department of Biosystems Science and Engineering, ETH Zürich	2020-03-26	Christian Beisel et al
Switzerland/100828/2020	EPI_ISL_451777	Viollier AG	Department of Biosystems Science and Engineering, ETH Zürich	2020-03-26	Christian Beisel et al
Switzerland/100830/2020	EPI_ISL_451778	Viollier AG	Department of Biosystems Science and Engineering, ETH Zürich	2020-03-26	Christian Beisel et al
Switzerland/100831/2020	EPI_ISL_451779	Viollier AG	Department of Biosystems Science and Engineering, ETH Zürich	2020-03-26	Christian Beisel et al
Switzerland/100832/2020	EPI_ISL_451780	Viollier AG	Department of Biosystems Science and Engineering, ETH Zürich	2020-03-25	Christian Beisel et al
Switzerland/100833/2020	EPI_ISL_451781	Viollier AG	Department of Biosystems Science and Engineering, ETH Zürich	2020-03-25	Christian Beisel et al
Switzerland/100834/2020	EPI_ISL_451782	Viollier AG	Department of Biosystems Science and Engineering, ETH Zürich	2020-03-25	Christian Beisel et al
Switzerland/100835/2020	EPI_ISL_451783	Viollier AG	Department of Biosystems Science and Engineering, ETH Zürich	2020-03-25	Christian Beisel et al
Switzerland/100836/2020	EPI_ISL_451784	Viollier AG	Department of Biosystems Science and Engineering, ETH Zürich	2020-03-25	Christian Beisel et al
Switzerland/100837/2020	EPI_ISL_451785	Viollier AG	Department of Biosystems Science and Engineering, ETH Zürich	2020-03-25	Christian Beisel et al
Switzerland/100838/2020	EPI_ISL_451786	Viollier AG	Department of Biosystems Science and Engineering, ETH Zürich	2020-03-25	Christian Beisel et al
Switzerland/100839/2020	EPI_ISL_451787	Viollier AG	Department of Biosystems Science and Engineering, ETH Zürich	2020-03-25	Christian Beisel et al
Switzerland/100840/2020	EPI_ISL_451788	Viollier AG	Department of Biosystems Science and Engineering, ETH Zürich	2020-03-25	Christian Beisel et al
Switzerland/100842/2020	EPI_ISL_451789	Viollier AG	Department of Biosystems Science and Engineering, ETH Zürich	2020-03-25	Christian Beisel et al
Switzerland/100843/2020	EPI_ISL_451790	Viollier AG	Department of Biosystems Science and Engineering, ETH Zürich	2020-03-25	Christian Beisel et al
Switzerland/100844/2020	EPI_ISL_451791	Viollier AG	Department of Biosystems Science and Engineering, ETH Zürich	2020-03-25	Christian Beisel et al
Switzerland/100845/2020	EPI_ISL_451792	Viollier AG	Department of Biosystems Science and Engineering, ETH Zürich	2020-03-25	Christian Beisel et al
Switzerland/100846/2020	EPI_ISL_451793	Viollier AG	Department of Biosystems Science and Engineering, ETH Zürich	2020-03-25	Christian Beisel et al
Switzerland/100848/2020	EPI_ISL_451794	Viollier AG	Department of Biosystems Science and Engineering, ETH Zürich	2020-03-25	Christian Beisel et al
Switzerland/101127/2020	EPI_ISL_451795	Viollier AG	Department of Biosystems Science and Engineering, ETH Zürich	2020-03-30	Christian Beisel et al
Switzerland/101129/2020	EPI_ISL_451796	Viollier AG	Department of Biosystems Science and Engineering, ETH Zürich	2020-03-30	Christian Beisel et al
Switzerland/101130/2020	EPI_ISL_451797	Viollier AG	Department of Biosystems Science and Engineering, ETH Zürich	2020-03-30	Christian Beisel et al
Switzerland/101131/2020	EPI_ISL_451798	Viollier AG	Department of Biosystems Science and Engineering, ETH Zürich	2020-03-30	Christian Beisel et al
Switzerland/101132/2020	EPI_ISL_451799	Viollier AG	Department of Biosystems Science and Engineering, ETH Zürich	2020-03-30	Christian Beisel et al
Switzerland/101133/2020	EPI_ISL_451800	Viollier AG	Department of Biosystems Science and Engineering, ETH Zürich	2020-03-30	Christian Beisel et al
Switzerland/101134/2020	EPI_ISL_451801	Viollier AG	Department of Biosystems Science and Engineering, ETH Zürich	2020-03-30	Christian Beisel et al
Switzerland/101135/2020	EPI_ISL_451802	Viollier AG	Department of Biosystems Science and Engineering, ETH Zürich	2020-03-30	Christian Beisel et al
Switzerland/101136/2020	EPI_ISL_451803	Viollier AG	Department of Biosystems Science and Engineering, ETH Zürich	2020-03-30	Christian Beisel et al
Switzerland/101137/2020	EPI_ISL_451804	Viollier AG	Department of Biosystems Science and Engineering, ETH Zürich	2020-03-30	Christian Beisel et al
Switzerland/101138/2020	EPI_ISL_451805	Viollier AG	Department of Biosystems Science and Engineering, ETH Zürich	2020-03-31	Christian Beisel et al
Switzerland/101139/2020	EPI_ISL_451806	Viollier AG	Department of Biosystems Science and Engineering, ETH Zürich	2020-03-31	Christian Beisel et al
Switzerland/101140/2020	EPI_ISL_451807	Viollier AG	Department of Biosystems Science and Engineering, ETH Zürich	2020-03-31	Christian Beisel et al

Switzerland/101141/2020	EPI_ISL_451808	Viollier AG	Department of Biosystems Science and Engineering, ETH Zürich	2020-03-31	Christian Beisel et al
Switzerland/101142/2020	EPI_ISL_451809	Viollier AG	Department of Biosystems Science and Engineering, ETH Zürich	2020-03-31	Christian Beisel et al
Switzerland/101143/2020	EPI_ISL_451810	Viollier AG	Department of Biosystems Science and Engineering, ETH Zürich	2020-03-31	Christian Beisel et al
Switzerland/101144/2020	EPI_ISL_451811	Viollier AG	Department of Biosystems Science and Engineering, ETH Zürich	2020-03-31	Christian Beisel et al
Switzerland/101145/2020	EPI_ISL_451812	Viollier AG	Department of Biosystems Science and Engineering, ETH Zürich	2020-03-31	Christian Beisel et al
Switzerland/101146/2020	EPI_ISL_451813	Viollier AG	Department of Biosystems Science and Engineering, ETH Zürich	2020-03-31	Christian Beisel et al
Switzerland/101147/2020	EPI_ISL_451814	Viollier AG	Department of Biosystems Science and Engineering, ETH Zürich	2020-03-31	Christian Beisel et al
Switzerland/101148/2020	EPI_ISL_451815	Viollier AG	Department of Biosystems Science and Engineering, ETH Zürich	2020-03-31	Christian Beisel et al
Switzerland/101149/2020	EPI_ISL_451816	Viollier AG	Department of Biosystems Science and Engineering, ETH Zürich	2020-03-31	Christian Beisel et al
Switzerland/101150/2020	EPI_ISL_451817	Viollier AG	Department of Biosystems Science and Engineering, ETH Zürich	2020-03-31	Christian Beisel et al
Switzerland/101151/2020	EPI_ISL_451818	Viollier AG	Department of Biosystems Science and Engineering, ETH Zürich	2020-03-31	Christian Beisel et al
Switzerland/101152/2020	EPI_ISL_451819	Viollier AG	Department of Biosystems Science and Engineering, ETH Zürich	2020-03-31	Christian Beisel et al
Switzerland/101155/2020	EPI_ISL_451821	Viollier AG	Department of Biosystems Science and Engineering, ETH Zürich	2020-03-31	Christian Beisel et al
Switzerland/101156/2020	EPI_ISL_451822	Viollier AG	Department of Biosystems Science and Engineering, ETH Zürich	2020-03-31	Christian Beisel et al
Switzerland/101157/2020	EPI_ISL_451823	Viollier AG	Department of Biosystems Science and Engineering, ETH Zürich	2020-03-31	Christian Beisel et al
Switzerland/101158/2020	EPI_ISL_451824	Viollier AG	Department of Biosystems Science and Engineering, ETH Zürich	2020-03-31	Christian Beisel et al
Switzerland/101160/2020	EPI_ISL_451826	Viollier AG	Department of Biosystems Science and Engineering, ETH Zürich	2020-03-31	Christian Beisel et al
Switzerland/101161/2020	EPI_ISL_451827	Viollier AG	Department of Biosystems Science and Engineering, ETH Zürich	2020-03-31	Christian Beisel et al
Switzerland/101165/2020	EPI_ISL_451829	Viollier AG	Department of Biosystems Science and Engineering, ETH Zürich	2020-03-31	Christian Beisel et al
Switzerland/101166/2020	EPI_ISL_451830	Viollier AG	Department of Biosystems Science and Engineering, ETH Zürich	2020-03-31	Christian Beisel et al
Switzerland/101167/2020	EPI_ISL_451831	Viollier AG	Department of Biosystems Science and Engineering, ETH Zürich	2020-03-31	Christian Beisel et al
Switzerland/101168/2020	EPI_ISL_451832	Viollier AG	Department of Biosystems Science and Engineering, ETH Zürich	2020-03-31	Christian Beisel et al
Switzerland/101170/2020	EPI_ISL_451833	Viollier AG	Department of Biosystems Science and Engineering, ETH Zürich	2020-03-31	Christian Beisel et al
Switzerland/101171/2020	EPI_ISL_451834	Viollier AG	Department of Biosystems Science and Engineering, ETH Zürich	2020-03-31	Christian Beisel et al
Switzerland/101172/2020	EPI_ISL_451835	Viollier AG	Department of Biosystems Science and Engineering, ETH Zürich	2020-03-31	Christian Beisel et al
Switzerland/101173/2020	EPI_ISL_451836	Viollier AG	Department of Biosystems Science and Engineering, ETH Zürich	2020-03-31	Christian Beisel et al
Switzerland/101174/2020	EPI_ISL_451837	Viollier AG	Department of Biosystems Science and Engineering, ETH Zürich	2020-03-31	Christian Beisel et al
Switzerland/101175/2020	EPI_ISL_451838	Viollier AG	Department of Biosystems Science and Engineering, ETH Zürich	2020-03-31	Christian Beisel et al
Switzerland/101177/2020	EPI_ISL_451839	Viollier AG	Department of Biosystems Science and Engineering, ETH Zürich	2020-03-31	Christian Beisel et al
Switzerland/101178/2020	EPI_ISL_451840	Viollier AG	Department of Biosystems Science and Engineering, ETH Zürich	2020-03-31	Christian Beisel et al
Switzerland/101179/2020	EPI_ISL_451841	Viollier AG	Department of Biosystems Science and Engineering, ETH Zürich	2020-03-31	Christian Beisel et al
Switzerland/101180/2020	EPI_ISL_451842	Viollier AG	Department of Biosystems Science and Engineering, ETH Zürich	2020-03-31	Christian Beisel et al
Switzerland/101181/2020	EPI_ISL_451843	Viollier AG	Department of Biosystems Science and Engineering, ETH Zürich	2020-03-31	Christian Beisel et al
Switzerland/101182/2020	EPI_ISL_451844	Viollier AG	Department of Biosystems Science and Engineering, ETH Zürich	2020-03-31	Christian Beisel et al
Switzerland/101183/2020	EPI_ISL_451845	Viollier AG	Department of Biosystems Science and Engineering, ETH Zürich	2020-03-30	Christian Beisel et al
Switzerland/101184/2020	EPI_ISL_451846	Viollier AG	Department of Biosystems Science and Engineering, ETH Zürich	2020-03-31	Christian Beisel et al
Switzerland/101186/2020	EPI_ISL_451848	Viollier AG	Department of Biosystems Science and Engineering, ETH Zürich	2020-03-31	Christian Beisel et al
Switzerland/101187/2020	EPI_ISL_451849	Viollier AG	Department of Biosystems Science and Engineering, ETH Zürich	2020-03-31	Christian Beisel et al
Switzerland/101188/2020	EPI_ISL_451850	Viollier AG	Department of Biosystems Science and Engineering, ETH Zürich	2020-03-31	Christian Beisel et al
Switzerland/101189/2020	EPI_ISL_451851	Viollier AG	Department of Biosystems Science and Engineering, ETH Zürich	2020-03-31	Christian Beisel et al
Switzerland/101190/2020	EPI_ISL_451852	Viollier AG	Department of Biosystems Science and Engineering, ETH Zürich	2020-03-31	Christian Beisel et al
Switzerland/101191/2020	EPI_ISL_451853	Viollier AG	Department of Biosystems Science and Engineering, ETH Zürich	2020-03-31	Christian Beisel et al
Switzerland/101192/2020	EPI_ISL_451854	Viollier AG	Department of Biosystems Science and Engineering, ETH Zürich	2020-03-31	Christian Beisel et al
Switzerland/101193/2020	EPI_ISL_451855	Viollier AG	Department of Biosystems Science and Engineering, ETH Zürich	2020-03-31	Christian Beisel et al
Switzerland/110426/2020	EPI_ISL_451856	Viollier AG	Department of Biosystems Science and Engineering, ETH Zürich	2020-04-06	Christian Beisel et al
Switzerland/110427/2020	EPI_ISL_451857	Viollier AG	Department of Biosystems Science and Engineering, ETH Zürich	2020-04-07	Christian Beisel et al
Switzerland/110429/2020	EPI_ISL_451858	Viollier AG	Department of Biosystems Science and Engineering, ETH Zürich	2020-04-07	Christian Beisel et al
Switzerland/110430/2020	EPI_ISL_451859	Viollier AG	Department of Biosystems Science and Engineering, ETH Zürich	2020-04-07	Christian Beisel et al
Switzerland/110431/2020	EPI_ISL_451860	Viollier AG	Department of Biosystems Science and Engineering, ETH Zürich	2020-04-07	Christian Beisel et al
Switzerland/110435/2020	EPI_ISL_451861	Viollier AG	Department of Biosystems Science and Engineering, ETH Zürich	2020-04-07	Christian Beisel et al
Switzerland/110436/2020	EPI_ISL_451862	Viollier AG	Department of Biosystems Science and Engineering, ETH Zürich	2020-04-07	Christian Beisel et al
Switzerland/110437/2020	EPI_ISL_451863	Viollier AG	Department of Biosystems Science and Engineering, ETH Zürich	2020-04-07	Christian Beisel et al
Switzerland/110439/2020	EPI_ISL_451864	Viollier AG	Department of Biosystems Science and Engineering, ETH Zürich	2020-04-07	Christian Beisel et al
Switzerland/110440/2020	EPI_ISL_451865	Viollier AG	Department of Biosystems Science and Engineering, ETH Zürich	2020-04-07	Christian Beisel et al
Switzerland/110442/2020	EPI_ISL_451866	Viollier AG	Department of Biosystems Science and Engineering, ETH Zürich	2020-04-07	Christian Beisel et al
Switzerland/110444/2020	EPI_ISL_451867	Viollier AG	Department of Biosystems Science and Engineering, ETH Zürich	2020-04-07	Christian Beisel et al
Switzerland/110445/2020	EPI_ISL_451868	Viollier AG	Department of Biosystems Science and Engineering, ETH Zürich	2020-04-07	Christian Beisel et al
Switzerland/110448/2020	EPI_ISL_451869	Viollier AG	Department of Biosystems Science and Engineering, ETH Zürich	2020-04-07	Christian Beisel et al
Switzerland/110450/2020	EPI_ISL_451870	Viollier AG	Department of Biosystems Science and Engineering, ETH Zürich	2020-04-07	Christian Beisel et al
Switzerland/110451/2020	EPI_ISL_451871	Viollier AG	Department of Biosystems Science and Engineering, ETH Zürich	2020-04-07	Christian Beisel et al
Switzerland/110452/2020	EPI_ISL_451872	Viollier AG	Department of Biosystems Science and Engineering, ETH Zürich	2020-04-07	Christian Beisel et al
Switzerland/110453/2020	EPI_ISL_451873	Viollier AG	Department of Biosystems Science and Engineering, ETH Zürich	2020-04-07	Christian Beisel et al
Switzerland/110454/2020	EPI_ISL_451874	Viollier AG	Department of Biosystems Science and Engineering, ETH Zürich	2020-04-07	Christian Beisel et al
Switzerland/110455/2020	EPI_ISL_451875	Viollier AG	Department of Biosystems Science and Engineering, ETH Zürich	2020-04-07	Christian Beisel et al
Switzerland/110457/2020	EPI_ISL_451876	Viollier AG	Department of Biosystems Science and Engineering, ETH Zürich	2020-04-07	Christian Beisel et al
Switzerland/110458/2020	EPI_ISL_451877	Viollier AG	Department of Biosystems Science and Engineering, ETH Zürich	2020-04-07	Christian Beisel et al
Switzerland/110459/2020	EPI_ISL_451878	Viollier AG	Department of Biosystems Science and Engineering, ETH Zürich	2020-04-07	Christian Beisel et al
Switzerland/110460/2020	EPI_ISL_451879	Viollier AG	Department of Biosystems Science and Engineering, ETH Zürich	2020-04-07	Christian Beisel et al
Switzerland/110461/2020	EPI_ISL_451880	Viollier AG	Department of Biosystems Science and Engineering, ETH Zürich	2020-04-07	Christian Beisel et al
Switzerland/110464/2020	EPI_ISL_451882	Viollier AG	Department of Biosystems Science and Engineering, ETH Zürich	2020-04-07	Christian Beisel et al
Switzerland/110465/2020	EPI_ISL_451883	Viollier AG	Department of Biosystems Science and Engineering, ETH Zürich	2020-04-07	Christian Beisel et al
Switzerland/110466/2020	EPI_ISL_451884	Viollier AG	Department of Biosystems Science and Engineering, ETH Zürich	2020-04-06	Christian Beisel et al
Switzerland/110467/2020	EPI_ISL_451885	Viollier AG	Department of Biosystems Science and Engineering, ETH Zürich	2020-04-06	Christian Beisel et al
Switzerland/110468/2020	EPI_ISL_451886	Viollier AG	Department of Biosystems Science and Engineering, ETH Zürich	2020-04-06	Christian Beisel et al
Switzerland/110469/2020	EPI_ISL_451887	Viollier AG	Department of Biosystems Science and Engineering, ETH Zürich	2020-04-06	Christian Beisel et al
Switzerland/110470/2020	EPI_ISL_451888	Viollier AG	Department of Biosystems Science and Engineering, ETH Zürich	2020-04-06	Christian Beisel et al
Switzerland/110474/2020	EPI_ISL_451889	Viollier AG	Department of Biosystems Science and Engineering, ETH Zürich	2020-04-06	Christian Beisel et al
Switzerland/110478/2020	EPI_ISL_451890	Viollier AG	Department of Biosystems Science and Engineering, ETH Zürich	2020-04-07	Christian Beisel et al
Switzerland/110479/2020	EPI_ISL_451891	Viollier AG	Department of Biosystems Science and Engineering, ETH Zürich	2020-04-06	Christian Beisel et al
Switzerland/110480/2020	EPI_ISL_451892	Viollier AG	Department of Biosystems Science and Engineering, ETH Zürich	2020-04-07	Christian Beisel et al
Switzerland/110481/2020	EPI_ISL_451893	Viollier AG	Department of Biosystems Science and Engineering, ETH Zürich	2020-04-07	Christian Beisel et al
Switzerland/110482/2020	EPI_ISL_451894	Viollier AG	Department of Biosystems Science and Engineering, ETH Zürich	2020-04-07	Christian Beisel et al
Switzerland/110486/2020	EPI_ISL_451898	Viollier AG	Department of Biosystems Science and Engineering, ETH Zürich	2020-04-07	Christian Beisel et al
Switzerland/110489/2020	EPI_ISL_451900	Viollier AG	Department of Biosystems Science and Engineering, ETH Zürich	2020-04-07	Christian Beisel et al
Switzerland/110491/2020	EPI_ISL_451902	Viollier AG	Department of Biosystems Science and Engineering, ETH Zürich	2020-04-07	Christian Beisel et al
Switzerland/110492/2020	EPI_ISL_451903	Viollier AG	Department of Biosystems Science and Engineering, ETH Zürich	2020-04-07	Christian Beisel et al
Switzerland/110497/2020	EPI_ISL_451905	Viollier AG	Department of Biosystems Science and Engineering, ETH Zürich	2020-04-07	Christian Beisel et al
Switzerland/110498/2020	EPI_ISL_451906	Viollier AG	Department of Biosystems Science and Engineering, ETH Zürich	2020-04-07	Christian Beisel et al
Switzerland/110499/2020	EPI_ISL_451907	Viollier AG	Department of Biosystems Science and Engineering, ETH Zürich	2020-04-07	Christian Beisel et al
Switzerland/110500/2020	EPI_ISL_451908	Viollier AG	Department of Biosystems Science and Engineering, ETH Zürich	2020-04-07	Christian Beisel et al
Switzerland/110503/2020	EPI_ISL_451909	Viollier AG	Department of Biosystems Science and Engineering, ETH Zürich	2020-04-07	Christian Beisel et al
Switzerland/110504/2020	EPI_ISL_451910	Viollier AG	Department of Biosystems Science and Engineering, ETH Zürich	2020-04-07	Christian Beisel et al
Switzerland/110505/2020	EPI_ISL_451911	Viollier AG	Department of Biosystems Science and Engineering, ETH Zürich	2020-04-07	Christian Beisel et al
Switzerland/110506/2020	EPI_ISL_451912	Viollier AG	Department of Biosystems Science and Engineering, ETH Zürich	2020-04-07	Christian Beisel et al
Switzerland/110507/2020	EPI_ISL_451913	Viollier AG	Department of Biosystems Science and Engineering, ETH Zürich	2020-04-07	Christian Beisel et al
Switzerland/110508/2020	EPI_ISL_451914	Viollier AG	Department of Biosystems Science and Engineering, ETH Zürich	2020-04-07	Christian Beisel et al
Switzerland/110509/2020	EPI_ISL_451915	Viollier AG	Department of Biosystems Science and Engineering, ETH Zürich	2020-04-07	Christian Beisel et al
Switzerland/110510/2020	EPI_ISL_451916	Viollier AG	Department of Biosystems Science and Engineering, ETH Zürich	2020-04-07	Christian Beisel et al
Switzerland/120001/2020	EPI_ISL_468202	Viollier AG	Department of Biosystems Science and Engineering, ETH Zürich	2020-04-17	Christian Beisel et al
Switzerland/120002/2020	EPI_ISL_468203	Viollier AG	Department of Biosystems Science and Engineering, ETH Zürich	2020-04-17	Christian Beisel et al
Switzerland/120003/2020	EPI_ISL_468204	Viollier AG	Department of Biosystems Science and Engineering, ETH Zürich	2020-04-17	Christian Beisel et al
Switzerland/120004/2020	EPI_ISL_468205	Viollier AG	Department of Biosystems Science and Engineering, ETH Zürich	2020-04-17	Christian Beisel et al
Switzerland/120006/2020	EPI_ISL_468207	Viollier AG	Department of Biosystems Science and Engineering, ETH Zürich	2020-04-17	Christian Beisel et al
Switzerland/120007/2020	EPI_ISL_468208	Viollier AG	Department of Biosystems Science and Engineering, ETH Zürich	2020-04-17	Christian Beisel et al
Switzerland/120009/2020	EPI_ISL_468209	Viollier AG	Department of Biosystems Science and Engineering, ETH Zürich	2020-04-17	Christian Beisel et al
Switzerland/120010/2020	EPI_ISL_468210	Viollier AG	Department of Biosystems Science and Engineering, ETH Zürich	2020-04-17	Christian Beisel et al
Switzerland/120011/2020	EPI_ISL_468211	Viollier AG	Department of Biosystems Science and Engineering, ETH Zürich	2020-04-17	Christian Beisel et al
Switzerland/120013/2020	EPI_ISL_468212	Viollier AG	Department of Biosystems Science and Engineering, ETH Zürich	2020-04-17	Christian Beisel et al
Switzerland/120014/2020	EPI_ISL_468213	Viollier AG	Department of Biosystems Science and Engineering, ETH Zürich	2020-04-17	Christian Beisel et al
Switzerland/120016/2020	EPI_ISL_468214	Viollier AG	Department of Biosystems Science and Engineering, ETH Zürich	2020-04-17	Christian Beisel et al
Switzerland/120017/2020	EPI_ISL_468215	Viollier AG	Department of Biosystems Science and Engineering, ETH Zürich	2020-04-17	Christian Beisel et al
Switzerland/120019/2020	EPI_ISL_468216	Viollier AG	Department of Biosystems Science and Engineering, ETH Zürich	2020-04-17	Christian Beisel et al
Switzerland/120021/2020	EPI_ISL_468218	Viollier AG	Department of Biosystems Science and Engineering, ETH Zürich	2020-04-17	Christian Beisel et al
Switzerland/120022/2020	EPI_ISL_468219	Viollier AG	Department of Biosystems Science and Engineering, ETH Zürich	2020-04-17	Christian Beisel et al
Switzerland/120025/2020	EPI_ISL_468220	Viollier AG	Department of Biosystems Science and Engineering, ETH Zürich	2020-04-17	Christian Beisel et al
Switzerland/120029/2020	EPI_ISL_468221	Viollier AG	Department of Biosystems Science and Engineering, ETH Zürich	2020-04-18	Christian Beisel et al

Switzerland/120030/2020	EPI_ISL_468222	Viollier AG	Department of Biosystems Science and Engineering, ETH Zürich	2020-04-18	Christian Beisel et al
Switzerland/120031/2020	EPI_ISL_468223	Viollier AG	Department of Biosystems Science and Engineering, ETH Zürich	2020-04-18	Christian Beisel et al
Switzerland/120033/2020	EPI_ISL_468224	Viollier AG	Department of Biosystems Science and Engineering, ETH Zürich	2020-04-18	Christian Beisel et al
Switzerland/120035/2020	EPI_ISL_468225	Viollier AG	Department of Biosystems Science and Engineering, ETH Zürich	2020-04-19	Christian Beisel et al
Switzerland/120036/2020	EPI_ISL_468226	Viollier AG	Department of Biosystems Science and Engineering, ETH Zürich	2020-04-20	Christian Beisel et al
Switzerland/120038/2020	EPI_ISL_468227	Viollier AG	Department of Biosystems Science and Engineering, ETH Zürich	2020-04-20	Christian Beisel et al
Switzerland/120039/2020	EPI_ISL_468228	Viollier AG	Department of Biosystems Science and Engineering, ETH Zürich	2020-04-20	Christian Beisel et al
Switzerland/120040/2020	EPI_ISL_468229	Viollier AG	Department of Biosystems Science and Engineering, ETH Zürich	2020-04-20	Christian Beisel et al
Switzerland/120041/2020	EPI_ISL_468230	Viollier AG	Department of Biosystems Science and Engineering, ETH Zürich	2020-04-20	Christian Beisel et al
Switzerland/120043/2020	EPI_ISL_468231	Viollier AG	Department of Biosystems Science and Engineering, ETH Zürich	2020-04-20	Christian Beisel et al
Switzerland/120044/2020	EPI_ISL_468232	Viollier AG	Department of Biosystems Science and Engineering, ETH Zürich	2020-04-20	Christian Beisel et al
Switzerland/120045/2020	EPI_ISL_468233	Viollier AG	Department of Biosystems Science and Engineering, ETH Zürich	2020-04-20	Christian Beisel et al
Switzerland/120047/2020	EPI_ISL_468234	Viollier AG	Department of Biosystems Science and Engineering, ETH Zürich	2020-04-20	Christian Beisel et al
Switzerland/120050/2020	EPI_ISL_468235	Viollier AG	Department of Biosystems Science and Engineering, ETH Zürich	2020-04-20	Christian Beisel et al
Switzerland/120060/2020	EPI_ISL_468237	Viollier AG	Department of Biosystems Science and Engineering, ETH Zürich	2020-04-20	Christian Beisel et al
Switzerland/120064/2020	EPI_ISL_468238	Viollier AG	Department of Biosystems Science and Engineering, ETH Zürich	2020-04-20	Christian Beisel et al
Switzerland/120067/2020	EPI_ISL_468239	Viollier AG	Department of Biosystems Science and Engineering, ETH Zürich	2020-04-20	Christian Beisel et al
Switzerland/120068/2020	EPI_ISL_468240	Viollier AG	Department of Biosystems Science and Engineering, ETH Zürich	2020-04-20	Christian Beisel et al
Switzerland/120070/2020	EPI_ISL_468241	Viollier AG	Department of Biosystems Science and Engineering, ETH Zürich	2020-04-20	Christian Beisel et al
Switzerland/120072/2020	EPI_ISL_468242	Viollier AG	Department of Biosystems Science and Engineering, ETH Zürich	2020-04-21	Christian Beisel et al
Switzerland/120164/2020	EPI_ISL_468243	Viollier AG	Department of Biosystems Science and Engineering, ETH Zürich	2020-04-25	Christian Beisel et al
Switzerland/120166/2020	EPI_ISL_468244	Viollier AG	Department of Biosystems Science and Engineering, ETH Zürich	2020-04-26	Christian Beisel et al
Switzerland/120167/2020	EPI_ISL_468245	Viollier AG	Department of Biosystems Science and Engineering, ETH Zürich	2020-04-26	Christian Beisel et al
Switzerland/120168/2020	EPI_ISL_468246	Viollier AG	Department of Biosystems Science and Engineering, ETH Zürich	2020-04-26	Christian Beisel et al
Switzerland/120169/2020	EPI_ISL_468247	Viollier AG	Department of Biosystems Science and Engineering, ETH Zürich	2020-04-26	Christian Beisel et al
Switzerland/120170/2020	EPI_ISL_468248	Viollier AG	Department of Biosystems Science and Engineering, ETH Zürich	2020-04-26	Christian Beisel et al
Switzerland/120171/2020	EPI_ISL_468249	Viollier AG	Department of Biosystems Science and Engineering, ETH Zürich	2020-04-26	Christian Beisel et al
Switzerland/120172/2020	EPI_ISL_468250	Viollier AG	Department of Biosystems Science and Engineering, ETH Zürich	2020-04-27	Christian Beisel et al
Switzerland/120174/2020	EPI_ISL_468251	Viollier AG	Department of Biosystems Science and Engineering, ETH Zürich	2020-04-27	Christian Beisel et al
Switzerland/120175/2020	EPI_ISL_468252	Viollier AG	Department of Biosystems Science and Engineering, ETH Zürich	2020-04-27	Christian Beisel et al
Switzerland/120177/2020	EPI_ISL_468253	Viollier AG	Department of Biosystems Science and Engineering, ETH Zürich	2020-04-27	Christian Beisel et al
Switzerland/120178/2020	EPI_ISL_468254	Viollier AG	Department of Biosystems Science and Engineering, ETH Zürich	2020-04-27	Christian Beisel et al
Switzerland/120179/2020	EPI_ISL_468255	Viollier AG	Department of Biosystems Science and Engineering, ETH Zürich	2020-04-27	Christian Beisel et al
Switzerland/120182/2020	EPI_ISL_468257	Viollier AG	Department of Biosystems Science and Engineering, ETH Zürich	2020-04-27	Christian Beisel et al
Switzerland/120183/2020	EPI_ISL_468258	Viollier AG	Department of Biosystems Science and Engineering, ETH Zürich	2020-04-27	Christian Beisel et al
Switzerland/120184/2020	EPI_ISL_468259	Viollier AG	Department of Biosystems Science and Engineering, ETH Zürich	2020-04-27	Christian Beisel et al
Switzerland/120185/2020	EPI_ISL_468260	Viollier AG	Department of Biosystems Science and Engineering, ETH Zürich	2020-04-27	Christian Beisel et al
Switzerland/120188/2020	EPI_ISL_468263	Viollier AG	Department of Biosystems Science and Engineering, ETH Zürich	2020-04-27	Christian Beisel et al
Switzerland/120189/2020	EPI_ISL_468264	Viollier AG	Department of Biosystems Science and Engineering, ETH Zürich	2020-04-27	Christian Beisel et al
Switzerland/120190/2020	EPI_ISL_468265	Viollier AG	Department of Biosystems Science and Engineering, ETH Zürich	2020-04-27	Christian Beisel et al
Switzerland/120191/2020	EPI_ISL_468266	Viollier AG	Department of Biosystems Science and Engineering, ETH Zürich	2020-04-27	Christian Beisel et al
Switzerland/120192/2020	EPI_ISL_468267	Viollier AG	Department of Biosystems Science and Engineering, ETH Zürich	2020-04-27	Christian Beisel et al
Switzerland/120194/2020	EPI_ISL_468268	Viollier AG	Department of Biosystems Science and Engineering, ETH Zürich	2020-04-27	Christian Beisel et al
Switzerland/120195/2020	EPI_ISL_468269	Viollier AG	Department of Biosystems Science and Engineering, ETH Zürich	2020-04-27	Christian Beisel et al
Switzerland/120196/2020	EPI_ISL_468270	Viollier AG	Department of Biosystems Science and Engineering, ETH Zürich	2020-04-28	Christian Beisel et al
Switzerland/120198/2020	EPI_ISL_468272	Viollier AG	Department of Biosystems Science and Engineering, ETH Zürich	2020-04-28	Christian Beisel et al
Switzerland/120200/2020	EPI_ISL_468273	Viollier AG	Department of Biosystems Science and Engineering, ETH Zürich	2020-04-28	Christian Beisel et al
Switzerland/120202/2020	EPI_ISL_468275	Viollier AG	Department of Biosystems Science and Engineering, ETH Zürich	2020-04-28	Christian Beisel et al
Switzerland/120203/2020	EPI_ISL_468276	Viollier AG	Department of Biosystems Science and Engineering, ETH Zürich	2020-04-28	Christian Beisel et al
Switzerland/120204/2020	EPI_ISL_468277	Viollier AG	Department of Biosystems Science and Engineering, ETH Zürich	2020-04-28	Christian Beisel et al
Switzerland/120205/2020	EPI_ISL_468278	Viollier AG	Department of Biosystems Science and Engineering, ETH Zürich	2020-04-28	Christian Beisel et al
Switzerland/120206/2020	EPI_ISL_468279	Viollier AG	Department of Biosystems Science and Engineering, ETH Zürich	2020-04-27	Christian Beisel et al
Switzerland/120207/2020	EPI_ISL_468280	Viollier AG	Department of Biosystems Science and Engineering, ETH Zürich	2020-04-28	Christian Beisel et al
Switzerland/120208/2020	EPI_ISL_468281	Viollier AG	Department of Biosystems Science and Engineering, ETH Zürich	2020-04-28	Christian Beisel et al
Switzerland/120219/2020	EPI_ISL_468282	Viollier AG	Department of Biosystems Science and Engineering, ETH Zürich	2020-04-29	Christian Beisel et al
Switzerland/120221/2020	EPI_ISL_468284	Viollier AG	Department of Biosystems Science and Engineering, ETH Zürich	2020-04-30	Christian Beisel et al
Switzerland/120223/2020	EPI_ISL_468285	Viollier AG	Department of Biosystems Science and Engineering, ETH Zürich	2020-04-30	Christian Beisel et al
Switzerland/120224/2020	EPI_ISL_468286	Viollier AG	Department of Biosystems Science and Engineering, ETH Zürich	2020-04-30	Christian Beisel et al
Switzerland/120227/2020	EPI_ISL_468288	Viollier AG	Department of Biosystems Science and Engineering, ETH Zürich	2020-05-01	Christian Beisel et al
Switzerland/120228/2020	EPI_ISL_468289	Viollier AG	Department of Biosystems Science and Engineering, ETH Zürich	2020-05-01	Christian Beisel et al
Switzerland/120229/2020	EPI_ISL_468290	Viollier AG	Department of Biosystems Science and Engineering, ETH Zürich	2020-05-01	Christian Beisel et al
Switzerland/120230/2020	EPI_ISL_468291	Viollier AG	Department of Biosystems Science and Engineering, ETH Zürich	2020-05-01	Christian Beisel et al
Switzerland/120232/2020	EPI_ISL_468293	Viollier AG	Department of Biosystems Science and Engineering, ETH Zürich	2020-05-01	Christian Beisel et al
Switzerland/120233/2020	EPI_ISL_468294	Viollier AG	Department of Biosystems Science and Engineering, ETH Zürich	2020-05-01	Christian Beisel et al
Switzerland/120234/2020	EPI_ISL_468295	Viollier AG	Department of Biosystems Science and Engineering, ETH Zürich	2020-05-01	Christian Beisel et al
Switzerland/120235/2020	EPI_ISL_468296	Viollier AG	Department of Biosystems Science and Engineering, ETH Zürich	2020-05-01	Christian Beisel et al
Switzerland/120236/2020	EPI_ISL_468297	Viollier AG	Department of Biosystems Science and Engineering, ETH Zürich	2020-05-01	Christian Beisel et al
Switzerland/120237/2020	EPI_ISL_468298	Viollier AG	Department of Biosystems Science and Engineering, ETH Zürich	2020-05-01	Christian Beisel et al
Switzerland/120238/2020	EPI_ISL_468299	Viollier AG	Department of Biosystems Science and Engineering, ETH Zürich	2020-05-01	Christian Beisel et al
Switzerland/120241/2020	EPI_ISL_468301	Viollier AG	Department of Biosystems Science and Engineering, ETH Zürich	2020-05-04	Christian Beisel et al
Switzerland/120242/2020	EPI_ISL_468302	Viollier AG	Department of Biosystems Science and Engineering, ETH Zürich	2020-05-04	Christian Beisel et al
Switzerland/120244/2020	EPI_ISL_468303	Viollier AG	Department of Biosystems Science and Engineering, ETH Zürich	2020-05-04	Christian Beisel et al
Switzerland/130001/2020	EPI_ISL_466997	Viollier AG	Department of Biosystems Science and Engineering, ETH Zürich	2020-05-05	Christian Beisel et al
Switzerland/130003/2020	EPI_ISL_466999	Viollier AG	Department of Biosystems Science and Engineering, ETH Zürich	2020-05-05	Christian Beisel et al
Switzerland/130004/2020	EPI_ISL_467000	Viollier AG	Department of Biosystems Science and Engineering, ETH Zürich	2020-05-05	Christian Beisel et al
Switzerland/130005/2020	EPI_ISL_467001	Viollier AG	Department of Biosystems Science and Engineering, ETH Zürich	2020-05-06	Christian Beisel et al
Switzerland/130006/2020	EPI_ISL_467002	Viollier AG	Department of Biosystems Science and Engineering, ETH Zürich	2020-05-06	Christian Beisel et al
Switzerland/130007/2020	EPI_ISL_467003	Viollier AG	Department of Biosystems Science and Engineering, ETH Zürich	2020-05-06	Christian Beisel et al
Switzerland/130008/2020	EPI_ISL_467004	Viollier AG	Department of Biosystems Science and Engineering, ETH Zürich	2020-05-06	Christian Beisel et al
Switzerland/130012/2020	EPI_ISL_467006	Viollier AG	Department of Biosystems Science and Engineering, ETH Zürich	2020-05-07	Christian Beisel et al
Switzerland/130013/2020	EPI_ISL_467007	Viollier AG	Department of Biosystems Science and Engineering, ETH Zürich	2020-05-07	Christian Beisel et al
Switzerland/130014/2020	EPI_ISL_467008	Viollier AG	Department of Biosystems Science and Engineering, ETH Zürich	2020-05-07	Christian Beisel et al
Switzerland/130015/2020	EPI_ISL_467009	Viollier AG	Department of Biosystems Science and Engineering, ETH Zürich	2020-05-07	Christian Beisel et al
Switzerland/130018/2020	EPI_ISL_467010	Viollier AG	Department of Biosystems Science and Engineering, ETH Zürich	2020-05-08	Christian Beisel et al
Switzerland/130020/2020	EPI_ISL_467012	Viollier AG	Department of Biosystems Science and Engineering, ETH Zürich	2020-05-08	Christian Beisel et al
Switzerland/130021/2020	EPI_ISL_467013	Viollier AG	Department of Biosystems Science and Engineering, ETH Zürich	2020-05-11	Christian Beisel et al
Switzerland/130026/2020	EPI_ISL_467014	Viollier AG	Department of Biosystems Science and Engineering, ETH Zürich	2020-05-12	Christian Beisel et al
Switzerland/130028/2020	EPI_ISL_467015	Viollier AG	Department of Biosystems Science and Engineering, ETH Zürich	2020-05-13	Christian Beisel et al
Switzerland/130032/2020	EPI_ISL_467016	Viollier AG	Department of Biosystems Science and Engineering, ETH Zürich	2020-05-13	Christian Beisel et al
Switzerland/42169171/2020	EPI_ISL_418271	University Hospital Basel, Clinical Virology	University Hospital Basel, Labormedizin	2020-02-27	Hirsch et al
Switzerland/42169310/2020	EPI_ISL_418275	University Hospital Basel, Clinical Virology	University Hospital Basel, Clinical Bacteriology	2020-02-27	Hirsch et al
Switzerland/42169471/2020	EPI_ISL_418273	University Hospital Basel, Clinical Virology	University Hospital Basel, Labormedizin	2020-02-28	Hirsch et al
Switzerland/42170345/2020	EPI_ISL_418274	University Hospital Basel, Clinical Virology	University Hospital Basel, Clinical Bacteriology	2020-02-28	Hirsch et al
Switzerland/42174724/2020	EPI_ISL_418277	University Hospital Basel, Clinical Virology	University Hospital Basel, Clinical Bacteriology	2020-03-03	Hirsch et al
Switzerland/42175075/2020	EPI_ISL_418278	University Hospital Basel, Clinical Virology	University Hospital Basel, Clinical Bacteriology	2020-03-03	Hirsch et al
Switzerland/42175213/2020	EPI_ISL_418279	University Hospital Basel, Clinical Virology	University Hospital Basel, Clinical Bacteriology	2020-03-03	Hirsch et al
Switzerland/42175220/2020	EPI_ISL_418280	University Hospital Basel, Clinical Virology	University Hospital Basel, Clinical Bacteriology	2020-03-03	Hirsch et al
Switzerland/42176216/2020	EPI_ISL_418281	University Hospital Basel, Clinical Virology	University Hospital Basel, Clinical Bacteriology	2020-03-04	Hirsch et al
Switzerland/42176229/2020	EPI_ISL_418282	University Hospital Basel, Clinical Virology	University Hospital Basel, Clinical Bacteriology	2020-03-04	Hirsch et al
Switzerland/42176560/2020	EPI_ISL_418283	University Hospital Basel, Clinical Virology	University Hospital Basel, Clinical Bacteriology	2020-03-04	Hirsch et al
Switzerland/42176753/2020	EPI_ISL_418284	University Hospital Basel, Clinical Virology	University Hospital Basel, Clinical Bacteriology	2020-03-04	Hirsch et al
Switzerland/42176771/2020	EPI_ISL_418285	University Hospital Basel, Clinical Virology	University Hospital Basel, Clinical Bacteriology	2020-03-04	Hirsch et al
Switzerland/42177236/2020	EPI_ISL_418433	University Hospital Basel, Clinical Virology	University Hospital Basel, Clinical Bacteriology	2020-03-05	Hirsch et al
Switzerland/42177430/2020	EPI_ISL_418434	University Hospital Basel, Clinical Virology	University Hospital Basel, Clinical Bacteriology	2020-03-05	Hirsch et al
Switzerland/42177434/2020	EPI_ISL_418435	University Hospital Basel, Clinical Virology	University Hospital Basel, Clinical Bacteriology	2020-03-05	Hirsch et al
Switzerland/42177472/2020	EPI_ISL_418436	University Hospital Basel, Clinical Virology	University Hospital Basel, Clinical Virology	2020-03-05	Hirsch et al
Switzerland/42178712/2020	EPI_ISL_418437	University Hospital Basel, Clinical Virology	University Hospital Basel, Clinical Bacteriology	2020-03-06	Hirsch et al
Switzerland/42202619/2020	EPI_ISL_418439	University Hospital Basel, Clinical Virology	University Hospital Basel, Clinical Bacteriology	2020-03-22	Hirsch et al
Switzerland/42202622/2020	EPI_ISL_418438	University Hospital Basel, Clinical Virology	University Hospital Basel, Clinical Bacteriology	2020-03-22	Hirsch et al
Switzerland/42202805/2020	EPI_ISL_418440	University Hospital Basel, Clinical Virology	University Hospital Basel, Clinical Bacteriology	2020-03-20	Hirsch et al
Switzerland/AG0361/2020	EPI_ISL_413999	Laboratoire de Virologie, HUG	Swiss National Reference Centre for Influenza	2020-02-27	LAUBSCHER Florian et al. et al
Switzerland/AG7120/2020	EPI_ISL_415457	Hôpitaux universitaires de Genève Laboratoire de Virologie	Hôpitaux universitaires de Genève Laboratoire de Virologie	2020-02-29	Laubscher F. et al
Switzerland/BE2536/2020	EPI_ISL_415704	Hôpitaux universitaires de Genève Laboratoire de Virologie	Hôpitaux universitaires de Genève Laboratoire de Virologie	2020-03-04	Laubscher F. et al
Switzerland/BE6651/2020	EPI_ISL_415456	Hôpitaux universitaires de Genève Laboratoire de Virologie	Hôpitaux universitaires de Genève Laboratoire de Virologie	2020-02-29	Laubscher F. et al
Switzerland/BL0902/2020	EPI_ISL_414021	Laboratoire de Virologie, HUG	Swiss National Reference Centre for Influenza	2020-02-27	LAUBSCHER Florian et al. et al
Switzerland/BS0914/2020	EPI_ISL_415701	Hôpitaux universitaires de Genève Laboratoire de Virologie	Hôpitaux universitaires de Genève Laboratoire de Virologie	2020-03-02	Laubscher F. et al

Switzerland/GE0056/2020	EPI_ISL_429212	University Hospitals of Geneva Laboratory of Virology	University Hospitals of Geneva Laboratory of Virology	2020-03-24	Laubscher F. et al
Switzerland/GE0199-2/2020	EPI_ISL_429197	University Hospitals of Geneva Laboratory of Virology	University Hospitals of Geneva Laboratory of Virology	2020-03-21	Laubscher F. et al
Switzerland/GE0199/2020	EPI_ISL_415455	Hôpitaux universitaires de Genève Laboratoire de Virologie	Hôpitaux universitaires de Genève Laboratoire de Virologie	2020-02-28	Laubscher F. et al
Switzerland/GE0304/2020	EPI_ISL_429218	University Hospitals of Geneva Laboratory of Virology	University Hospitals of Geneva Laboratory of Virology	2020-04-02	Laubscher F. et al
Switzerland/GE06207/2020	EPI_ISL_415706	Hôpitaux universitaires de Genève Laboratoire de Virologie	Hôpitaux universitaires de Genève Laboratoire de Virologie	2020-03-06	Laubscher F. et al
Switzerland/GE0636/2020	EPI_ISL_429199	University Hospitals of Geneva Laboratory of Virology	University Hospitals of Geneva Laboratory of Virology	2020-03-22	Laubscher F. et al
Switzerland/GE1184/2020	EPI_ISL_429207	University Hospitals of Geneva Laboratory of Virology	University Hospitals of Geneva Laboratory of Virology	2020-04-03	Laubscher F. et al
Switzerland/GE1402/2020	EPI_ISL_415700	Hôpitaux universitaires de Genève Laboratoire de Virologie	Hôpitaux universitaires de Genève Laboratoire de Virologie	2020-02-28	Laubscher F. et al
Switzerland/GE1422/2020	EPI_ISL_415454	Hôpitaux universitaires de Genève Laboratoire de Virologie	Hôpitaux universitaires de Genève Laboratoire de Virologie	2020-02-28	Laubscher F. et al
Switzerland/GE1736/2020	EPI_ISL_429203	University Hospitals of Geneva Laboratory of Virology	University Hospitals of Geneva Laboratory of Virology	2020-03-21	Laubscher F. et al
Switzerland/GE2164/2020	EPI_ISL_429208	University Hospitals of Geneva Laboratory of Virology	University Hospitals of Geneva Laboratory of Virology	2020-03-17	Laubscher F. et al
Switzerland/GE2297/2020	EPI_ISL_429201	University Hospitals of Geneva Laboratory of Virology	University Hospitals of Geneva Laboratory of Virology	2020-03-29	Laubscher F. et al
Switzerland/GE2453/2020	EPI_ISL_429219	University Hospitals of Geneva Laboratory of Virology	University Hospitals of Geneva Laboratory of Virology	2020-04-03	Laubscher F. et al
Switzerland/GE2759/2020	EPI_ISL_429214	University Hospitals of Geneva Laboratory of Virology	University Hospitals of Geneva Laboratory of Virology	2020-03-23	Laubscher F. et al
Switzerland/GE3121/2020	EPI_ISL_414019	Laboratoire de Virologie, HUG	Swiss National Reference Centre for Influenza	2020-02-27	LAUBSCHER Florian et al. et al
Switzerland/GE3144/2020	EPI_ISL_429196	University Hospitals of Geneva Laboratory of Virology	University Hospitals of Geneva Laboratory of Virology	2020-03-18	Laubscher F. et al
Switzerland/GE3650/2020	EPI_ISL_429209	University Hospitals of Geneva Laboratory of Virology	University Hospitals of Geneva Laboratory of Virology	2020-03-16	Laubscher F. et al
Switzerland/GE3655/2020	EPI_ISL_429222	University Hospitals of Geneva Laboratory of Virology	University Hospitals of Geneva Laboratory of Virology	2020-04-07	Laubscher F. et al
Switzerland/GE3895/2020	EPI_ISL_413997	Laboratoire de Virologie, HUG	Swiss National Reference Centre for Influenza	2020-02-26	LAUBSCHER Florian et al. et al
Switzerland/GE4135/2020	EPI_ISL_415705	Hôpitaux universitaires de Genève Laboratoire de Virologie	Hôpitaux universitaires de Genève Laboratoire de Virologie	2020-03-06	Laubscher F. et al
Switzerland/GE4644/2020	EPI_ISL_429223	University Hospitals of Geneva Laboratory of Virology	University Hospitals of Geneva Laboratory of Virology	2020-04-07	Laubscher F. et al
Switzerland/GE4807/2020	EPI_ISL_429215	University Hospitals of Geneva Laboratory of Virology	University Hospitals of Geneva Laboratory of Virology	2020-03-23	Laubscher F. et al
Switzerland/GE4984/2020	EPI_ISL_415708	Hôpitaux universitaires de Genève Laboratoire de Virologie	Hôpitaux universitaires de Genève Laboratoire de Virologie	2020-03-07	Laubscher F. et al
Switzerland/GE5007/2020	EPI_ISL_429206	University Hospitals of Geneva Laboratory of Virology	University Hospitals of Geneva Laboratory of Virology	2020-04-07	Laubscher F. et al
Switzerland/GE5373/2020	EPI_ISL_414020	Laboratoire de Virologie, HUG	Swiss National Reference Centre for Influenza	2020-02-27	LAUBSCHER Florian et al. et al
Switzerland/GE5708/2020	EPI_ISL_429211	University Hospitals of Geneva Laboratory of Virology	University Hospitals of Geneva Laboratory of Virology	2020-03-24	Laubscher F. et al
Switzerland/GE5737/2020	EPI_ISL_429200	University Hospitals of Geneva Laboratory of Virology	University Hospitals of Geneva Laboratory of Virology	2020-03-19	Laubscher F. et al
Switzerland/GE6065/2020	EPI_ISL_429220	University Hospitals of Geneva Laboratory of Virology	University Hospitals of Geneva Laboratory of Virology	2020-04-06	Laubscher F. et al
Switzerland/GE6099/2020	EPI_ISL_429221	University Hospitals of Geneva Laboratory of Virology	University Hospitals of Geneva Laboratory of Virology	2020-04-06	Laubscher F. et al
Switzerland/GE6414/2020	EPI_ISL_429217	University Hospitals of Geneva Laboratory of Virology	University Hospitals of Geneva Laboratory of Virology	2020-04-06	Laubscher F. et al
Switzerland/GE6679/2020	EPI_ISL_415707	Hôpitaux universitaires de Genève Laboratoire de Virologie	Hôpitaux universitaires de Genève Laboratoire de Virologie	2020-03-08	Laubscher F. et al
Switzerland/GE7617/2020	EPI_ISL_429204	University Hospitals of Geneva Laboratory of Virology	University Hospitals of Geneva Laboratory of Virology	2020-04-03	Laubscher F. et al
Switzerland/GE8086/2020	EPI_ISL_429213	University Hospitals of Geneva Laboratory of Virology	University Hospitals of Geneva Laboratory of Virology	2020-03-16	Laubscher F. et al
Switzerland/GE8102/2020	EPI_ISL_415458	Hôpitaux universitaires de Genève Laboratoire de Virologie	Hôpitaux universitaires de Genève Laboratoire de Virologie	2020-03-01	Laubscher F. et al
Switzerland/GE8147/2020	EPI_ISL_429210	University Hospitals of Geneva Laboratory of Virology	University Hospitals of Geneva Laboratory of Virology	2020-03-16	Laubscher F. et al
Switzerland/GE9586/2020	EPI_ISL_414022	Laboratoire de Virologie, HUG	Swiss National Reference Centre for Influenza	2020-02-27	LAUBSCHER Florian et al. et al
Switzerland/GR2988/2020	EPI_ISL_415698	Hôpitaux universitaires de Genève Laboratoire de Virologie	Hôpitaux universitaires de Genève Laboratoire de Virologie	2020-02-27	Laubscher F. et al
Switzerland/GR3043/2020	EPI_ISL_415699	Hôpitaux universitaires de Genève Laboratoire de Virologie	Hôpitaux universitaires de Genève Laboratoire de Virologie	2020-02-27	Laubscher F. et al
Switzerland/SZ1417/2020	EPI_ISL_415702	Hôpitaux universitaires de Genève Laboratoire de Virologie	Hôpitaux universitaires de Genève Laboratoire de Virologie	2020-03-02	Laubscher F. et al
Switzerland/TI2045/2020	EPI_ISL_415703	Hôpitaux universitaires de Genève Laboratoire de Virologie	Hôpitaux universitaires de Genève Laboratoire de Virologie	2020-03-01	Laubscher F. et al
Switzerland/TI9486/2020	EPI_ISL_413996	Laboratoire de Virologie, HUG	Swiss National Reference Centre for Influenza	2020-02-24	LAUBSCHER Florian et al. et al
Switzerland/VD0503/2020	EPI_ISL_415459	Hôpitaux universitaires de Genève Laboratoire de Virologie	Hôpitaux universitaires de Genève Laboratoire de Virologie	2020-02-29	Laubscher F. et al
Switzerland/VD5615/2020	EPI_ISL_414023	Laboratoire de Virologie, HUG	Swiss National Reference Centre for Influenza	2020-03-01	LAUBSCHER Florian et al. et al
Wuhan/WH01/2019	EPI_ISL_406798	General Hospital of Central Theater Command of People's Liberation Army of China	BGI & Institute of Microbiology, Chinese Academy of Sciences & Shandong First Medical University & Shandong Academy of Medical Sciences & General Hospital of Central Theater Command of People's Liberation Army of China	2019-12-26	Weijun Chen et al (https://dx.doi.org/10.1016/S0140-6736(20)30251-8)
